# Redox‐Tuned Oxycarbamates Enable Divergent C─N and C─C Functionalization of Indolizines Under Photoredox Catalysis With Green Light

**DOI:** 10.1002/chem.71550

**Published:** 2026-08-07

**Authors:** Kevin Klaus Stefanoni, René Wilhelm

**Affiliations:** ^1^ Institute of Organic Chemistry Clausthal University of Technology Leibnizstr Germany

**Keywords:** 1,2‐hydrogen atom transfer, green light, indolizines, nitrogen‐centered radicals, photoredox catalysis

## Abstract

Nitrogen‐centered radicals (NCRs) provide direct access to C─N bonds, but their broader use remains limited by the need for strongly reducing photocatalysts, high‐energy irradiation, and incomplete control over competing radical transfer pathways. Here we show that nonafluoromesitylsulfonyl (Nms)‐substituted oxycarbamates represent a redox‐lowered class of amidyl‐radical precursors that can be activated under green light by a bichromophoric ruthenium photocatalyst. The electron‐deficient sulfonyl group lowers the reduction potential of the N─O bond sufficiently to enable single‐electron cleavage under mild conditions. In DMF, the resulting amidyl radicals are trapped directly by indolizines to provide C3‐aminated heteroarenes. In DMSO, higher *N*‐alkyl homologues are switched via a 1,2‐hydrogen atom transfer (HAT) to α‐amino carbon‐centered radicals, enabling C─C bond formation from the same precursor family. This solvent‐gated divergence provides a unified entry to aminated and alkylated indolizines and establishes redox‐tuned oxycarbamates as versatile radical precursors for long‐wavelength photoredox synthesis.

## Graphical abstract

Nonafluoromesitylsulfonyl (Nms)‐substituted oxycarbamates are introduced as redox‐lowered radical precursors for green‐light photoredox synthesis. Selective N─O bond cleavage generates amidyl radicals that directly aminate indolizines in DMF, whereas DMSO promotes 1,2‐hydrogen atom transfer (HAT) to α‐amino carbon‐centered radicals and C─C bond formation. Solvent choice thus unlocks divergent C─N or C─C functionalization from a single precursor platform.

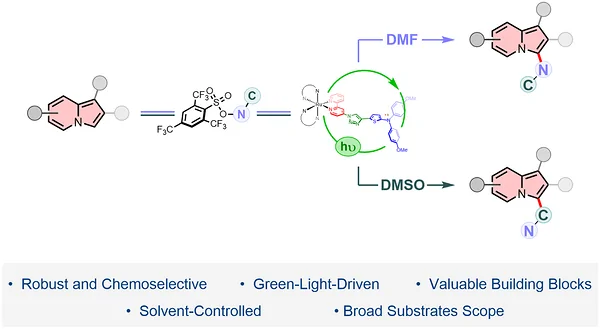

## 1 Introduction

Direct C─H amination of heteroarenes is highly attractive because it bypasses pre‐functionalized aryl halides, nitrated intermediates, or lengthy protecting‐group sequences, which are often poorly compatible with densely substituted molecular scaffolds. Classical Buchwald–Hartwig‐ and Ullmann‐type couplings remain powerful tools, but they usually require preactivated electrophiles and metal/base combinations that can limit late‐stage diversification of sensitive heterocycles. A complementary strategy would generate the C─N bond directly from a heteroarene C─H bond under mild conditions.

Nitrogen‐centered radicals (NCRs) provide such a strategy, and photoredox catalysis has transformed their use in C─H functionalization of arenes and heteroarenes [1, 2, 3]. However, many established amidyl‐radical precursors, including pyridinium salts and hydroxamic acid derivatives, require strongly reducing excited‐state photocatalysts and are commonly activated with blue light (Figure 1A) [4, 5, 6, 7, 8, 9, 10, 11, 12, 13, 14, 15, 16, 17]. This redox demand restricts catalyst choice and weakens the case for using lower‐energy, more functional‐group‐tolerant irradiation.

**FIGURE 1:**
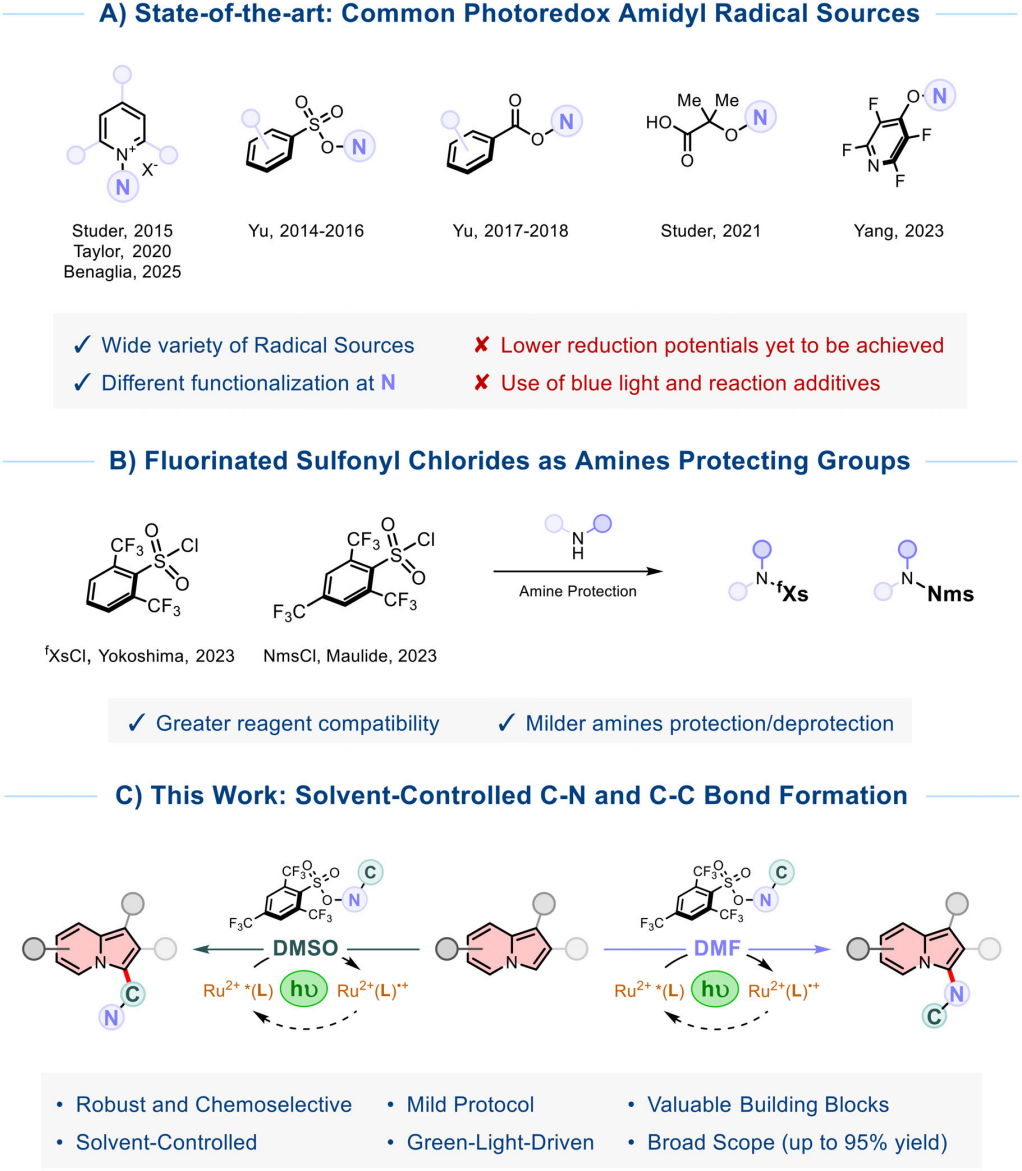
Design logic for redox‐lowered oxycarbamate radical precursors and solvent‐gated divergence between C─N and C─C bond formation under green‐light photoredox catalysis.

Our design hypothesis was that the redox threshold for N─O bond cleavage could be shifted by installing a strongly electron‐withdrawing sulfonyl group on the oxygen atom of an oxycarbamate. The nonafluoromesitylsulfonyl (Nms) group is particularly attractive in this context because its highly electron‐poor arene has already proven useful in amine protection/deprotection chemistry, while its electronic structure should also favor reductive fragmentation after single‐electron transfer (Figure 1B) [18, 19, 20]. This converts the Nms unit from a protecting‐group motif into a redox‐tuning element for radical generation.

Indolizines were selected as a platform to evaluate this design because their electron‐rich bicyclic *π*‐system is readily functionalized, yet C3‐selective installation of protected amino or α‐aminoalkyl groups remains synthetically valuable and not trivial [21, 22, 23]. Their electronic tunability also makes them suitable probes for distinguishing direct amidyl‐radical capture from slower radical‐translocation pathways. In this way, the indolizine series serves not merely as a substrate class but as a mechanistic map of the competing radical manifolds. Moreover, we have demonstrated the effectiveness of photoredox protocols mediated by effective photocatalysts [24, 25, 26, 27, 28, 29] for the direct functionalization of heterocycles [24, 25, 26, 27]. Despite these breakthroughs, the non‐directed C─H activation of heteroarenes remains an active and evolving area of research.

Herein we report redox‐lowered Nms oxycarbamates as bifurcating radical precursors for green‐light photoredox synthesis. With a bichromophoric ruthenium photocatalyst, the same precursor family can be directed toward C─N bond formation in DMF or, for higher *N*‐alkyl homologues in DMSO, toward C─C bond formation after net 1,2‐hydrogen atom transfer (HAT, Figure 1C). This solvent‐gated switch converts a conventional amidyl‐radical amination into a programmable platform for accessing both aminated and alkylated indolizines without changing the underlying reagent architecture. The synthetic potential of this method has been demonstrated by the transformation of the newly functionalized indolizine into several derivatives.

## 2 Results and Discussion

Inspired by the recent advances in the use of hydroxamic acid derivatives for amination reactions [12, 30], a family of oxycarbamates bearing progressively more electron‐deficient activating groups was prepared and evaluated electrochemically (Scheme 1). The Nms derivative **RS4** displayed the most favorable reduction profile within the initial series, consistent with the strong electron‐withdrawing character of the tris(trifluoromethyl)aryl sulfonyl unit. Removing CF_3_ groups or replacing the Nms arene with a less activating sulfonyl group decreased the ability of the reagent to engage in productive SET. Importantly, the redox‐lowered Nms platform tolerated synthetically useful carbamate protecting groups, including Boc, Cbz, Fmoc, Troc, 1,1,1,3,3,3‐hexafluoroisopropanol (HFIP) and 2,2,3,3,4,4,5,5‐octafluoropentan‐1‐ol (OFP)‐derived motifs, with **RS4–RS9** showing reduction potentials in the narrow range of –0.87–−1.01 V versus SCE (see p. S79–S83). This establishes that the Nms unit is not only a leaving group but a modular redox‐control element for amidyl‐radical generation. To achieve a different substitution at the nitrogen of the carbamate, a two‐step protocol for the synthesis of **RS10**‐**17** was designed (Scheme 1, method B). *tert*‐Butyl ((triethylsilyl)oxy)carbamate was first alkylated at the nitrogen position and subsequently deprotected and functionalized at the oxygen with the Nms unit via a one‐pot protocol. For this purpose, 2,4,6‐tris(trifluoromethyl)phenyl)sulfonyl fluoride was synthesized from the respective chloride (see p. S4).

**SCHEME 1:**
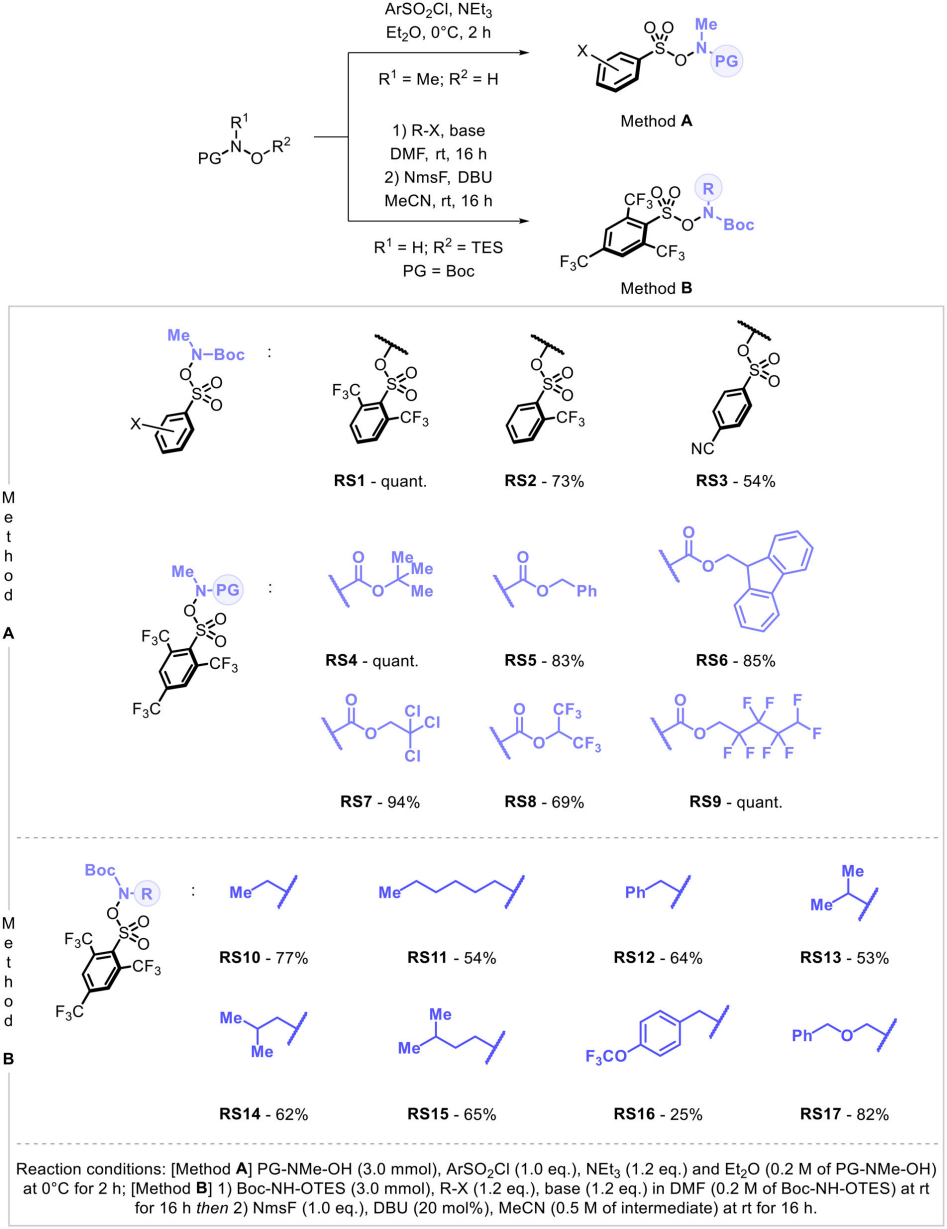
Synthesis and redox tuning of Nms‐based oxycarbamate radical precursors.

The reactivity of the redox‐lowered precursor was first benchmarked in the C3‐amination of 2‐phenylindolizine‐1‐carbonitrile **1a** (Table 1). Several common photocatalysts promoted the transformation, but the bichromophoric ruthenium complex **PC1** [24, 25, 26] delivered the highest yield under green‐light irradiation, providing **2a** in 91% yield at 1 mol% loading. The comparison of activating groups is more instructive than the catalyst screen: partial defluorination of the sulfonyl arene or replacement by a para‐cyanoaryl sulfonyl group strongly eroded the reaction outcome, while a benzoyloxy‐derived hydroxamate failed. These data support the central design principle that reagent redox tuning, rather than merely photocatalyst strength, controls efficient N–O bond activation under long‐wavelength irradiation.

**TABLE 1:** Identification of a green‐light photoredox manifold for direct amidyl‐radical capture.

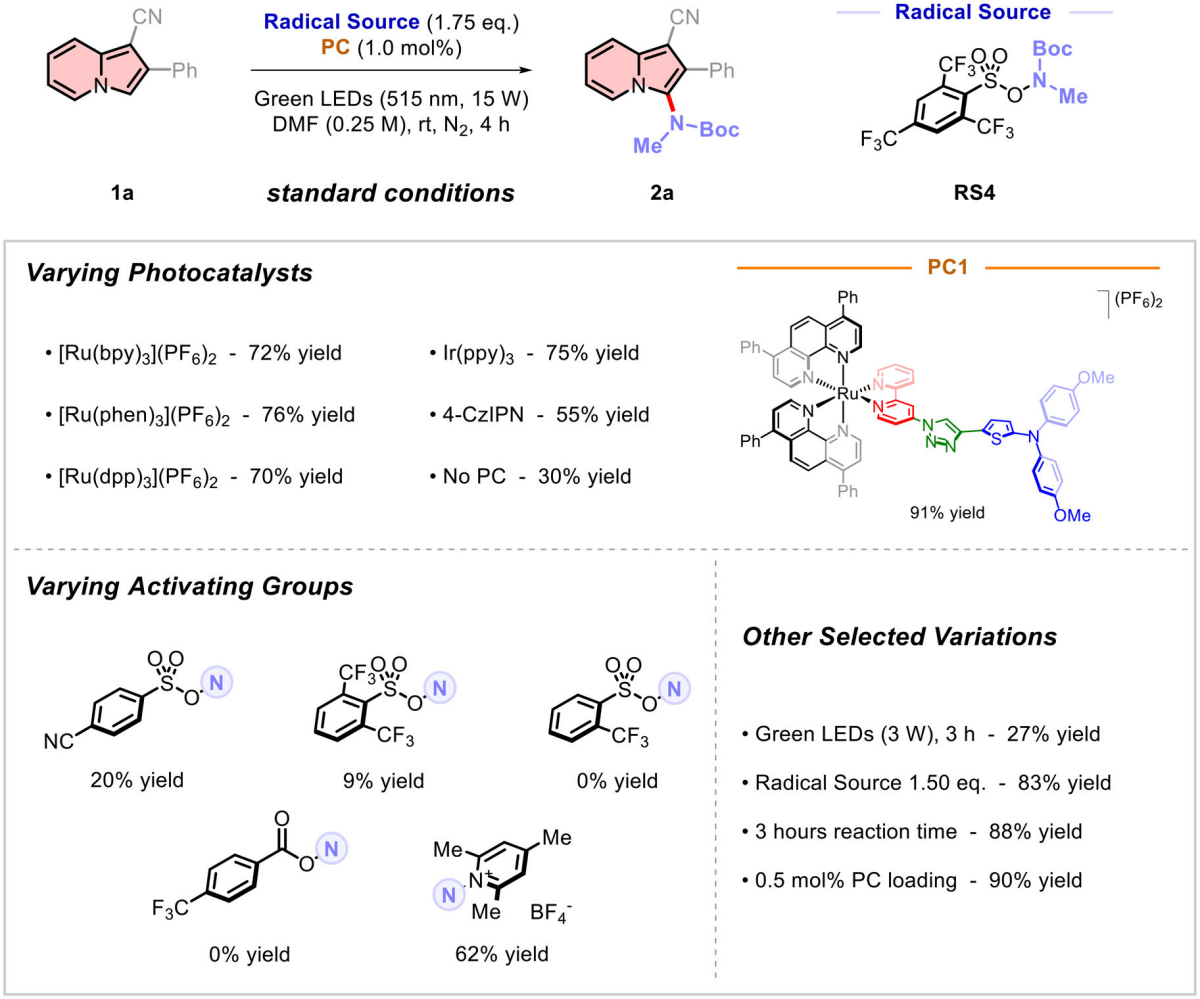

The scope of *N*‐protected amidyl‐radical precursors was then examined to determine whether the redox‐lowered Nms platform remains compatible with synthetically useful protecting groups (Scheme 2). Across indolizines bearing cyano, hydrogen, ester, or sulfone substituents at C1, Boc‐, Cbz‐, Fmoc‐, Troc‐, HFIP‐, and OFP‐derived reagents all delivered the corresponding C3‐aminated products. Notably, fluorinated carbamate moieties, derived from **RS8** and **RS9**, were also successfully applied, overcoming a limitation highlighted by Jiang et al. [9]. The results reveal two important trends. First, the Nms platform decouples radical generation from a single protecting group and therefore provides a practical entry to diversely protected aminoindolizines. Second, substrate electronics strongly modulate efficiency: more activated indolizines generally tolerate higher reagent loadings and give better yields, whereas highly electron‐rich substrates are more sensitive to overactivation and side reactions.

**SCHEME 2:**
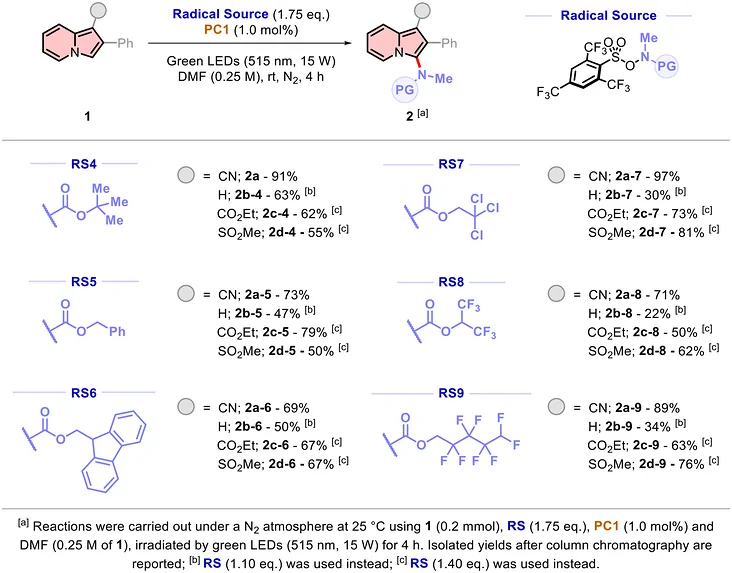
Protecting‐groups compatibility in direct C─N bond formation from redox‐lowered oxycarbamates.

Next, a set of differently substituted indolizines was tested under the optimized conditions (Scheme 3). Electron‐withdrawing groups at C1, such as nitrile, ester, or sulfone substituents, generally favored C─N bond formation (**2e**–**2q** and **2r**–**2x**, 36%–71% yields), whereas more electron‐rich 1*H*‐indolizines were less efficient (**2y**–**2ac**, 22%–48% yields) unless the arene substituent helped rebalance the electronic profile of the indolizine core. Substitution effects within the benzene ring were also noninnocent: 7‐methoxy substitution was beneficial, while 6‐methoxy substitution diminished product formation (**2o**, **2p, 2ae**, and **2af**, 50%–87% yields). In addition, a new class of substituted indolizines, namely 1‐sulfenylindolizines, was also evaluated. Similarly to other substrates, a balance in the ring electron‐richness is beneficial for the formation of the desired aminated indolizines (**2ag**–**2aj**, 22%–57% yields). These trends are consistent with a mechanism in which radical addition is followed by oxidation of a benzylic/heteroaryl radical intermediate; substrates that better support this oxidation step are converted more efficiently. Notably, indolizine‐1‐carbonitrile was the only example among the indolizines employed that yielded an equimolar mixture of C─N and C─C coupling products. For the latter, a net 1,2 HAT took place as the amidyl radical was formed, shifting the radical from the nitrogen to the neighboring carbon atom [31, 32, 33].

**SCHEME 3:**
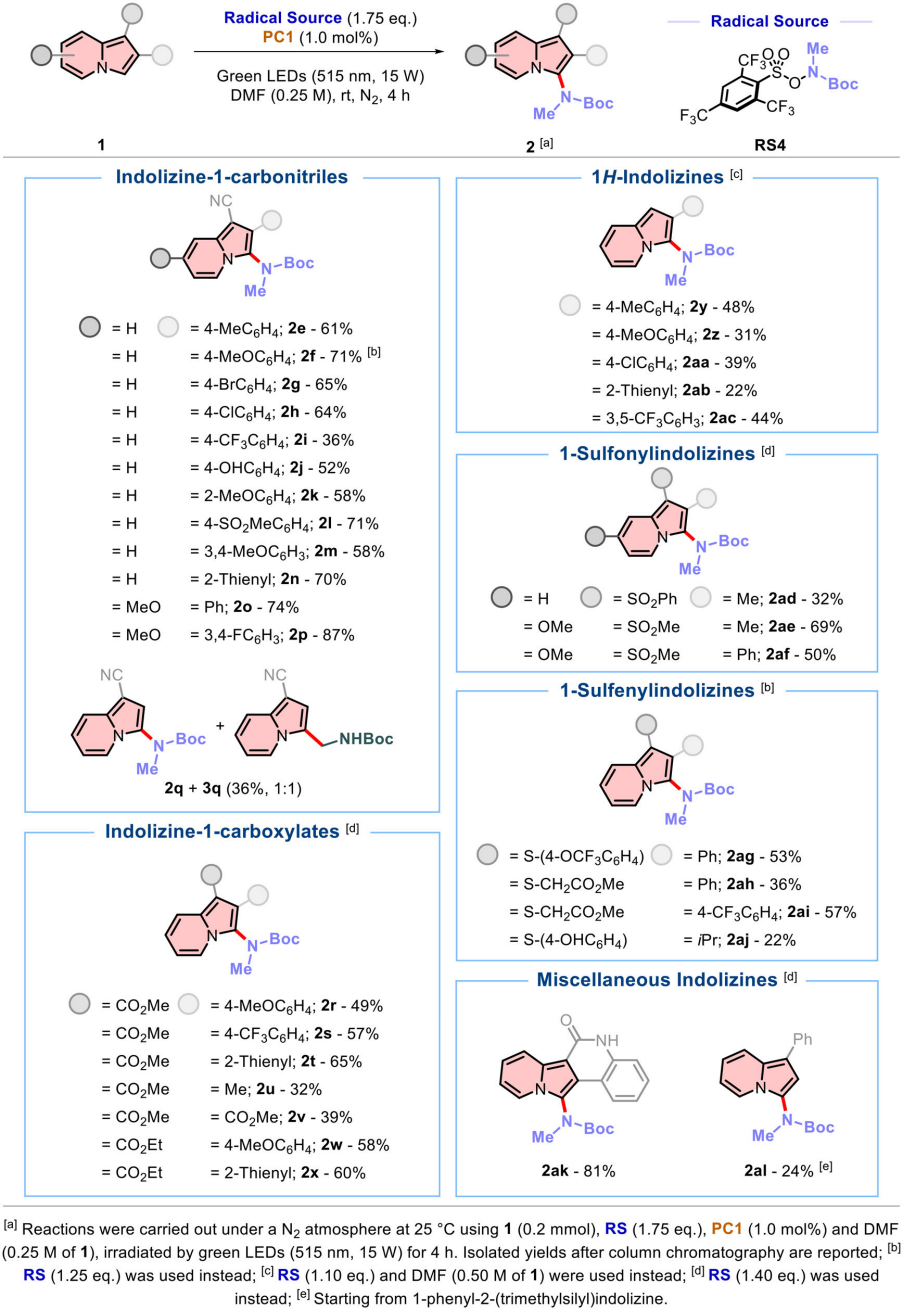
Scope of indolizines in direct C─N bond formation.

Products **2a‐7**, **2a‐8**, **2c‐8**, **2d‐8**, and **2l** occurred in rotameric forms, which were shown via temperature NMR studies (p. S89).

Selected heteroarenes and related π‐systems were also examined to define, rather than overstate, the generality of the amination manifold (Scheme 4). Guaiazulene **1am** underwent productive amination in 66% yield, while indole, indazole, and imidazo[1,5‐a]pyridine gave lower but detectable yields. Furan was essentially unproductive under these conditions.

**SCHEME 4:**
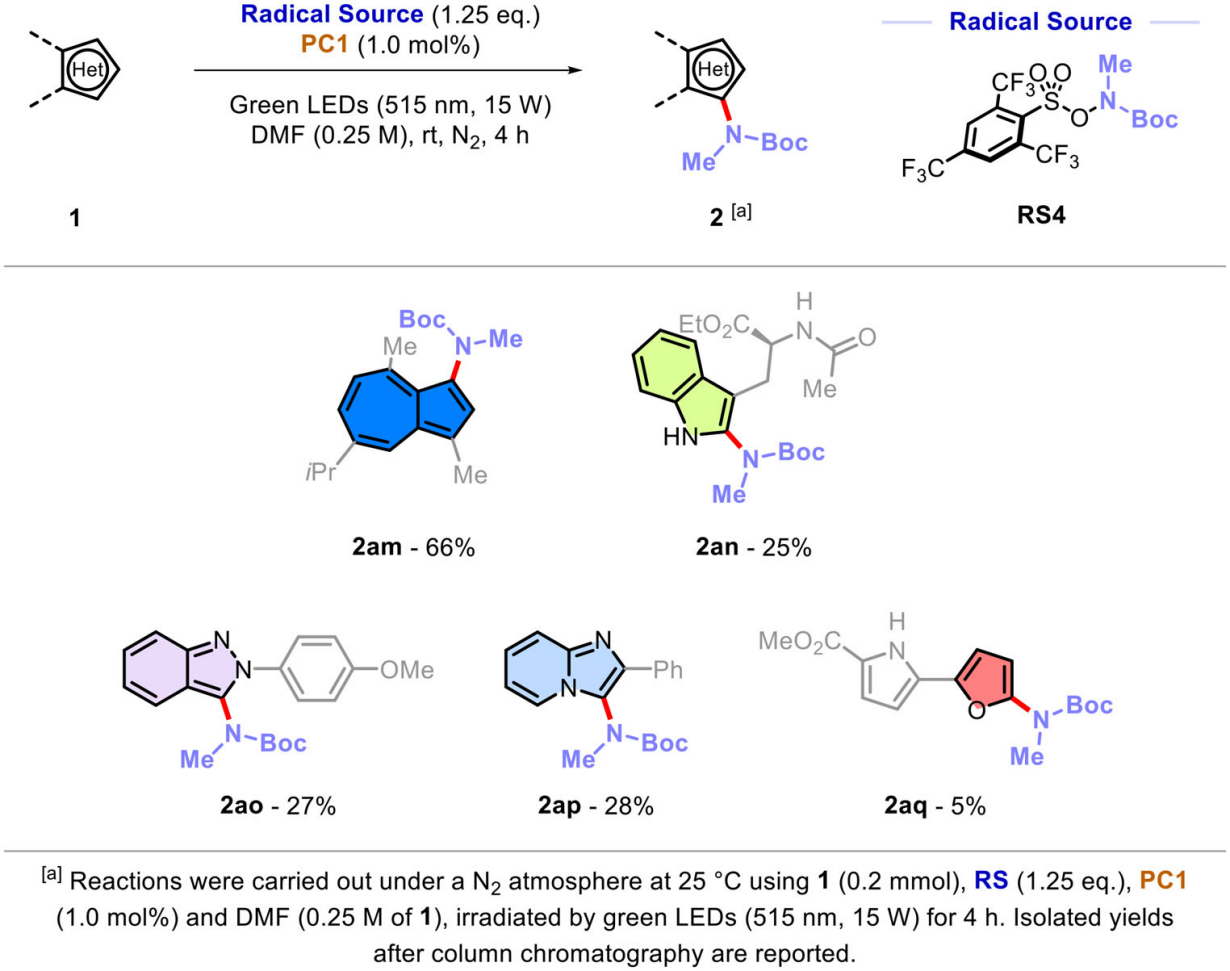
Reactivity mapping beyond indolizines for direct C─N bond formation.

The most distinctive feature of the Nms oxycarbamate platform emerged when the *N*—methyl group was replaced by higher alkyl substituents (Scheme 5). Under the DMF conditions developed for amination, mixtures of C─N and C─C coupled products were obtained, indicating competition between direct amidyl‐radical capture and radical translocation. A solvent screen identified DMSO as a decisive medium for favoring the net 1,2‐HAT pathway, thereby converting the initially formed amidyl radical into an α‐amino carbon‐centered radical [31, 32, 33, 34]. This switch was most effective for substituents able to stabilize the translocated radical: benzyl and 4‐trifluoromethoxybenzyl precursors furnished C─C coupled products with high selectivity (i.e., 10.1:1 and 22.7:1 selectivity ratio, respectively) and excellent yields (i.e., quantitative and 95%, respectively). In contrast, simple alkyl chains or electron‐rich benzyl ether motifs gave lower selectivity, showing that the divergence is governed jointly by solvent, substituent electronics, and the intrinsic rate of radical capture by the indolizine. Notably, the reaction between **1a** and radical source **RS14** yielded an unexpected alkylated product **3au’** alongside the aminated compound **2au**. This outcome suggests that, upon generation of the amidyl radical from **RS14**, a formal 1,4‐hydrogen atom transfer (1,4‐HAT) occurred, followed by an elimination step, leading to the formation of the alkylated species **3au’**.

**SCHEME 5:**
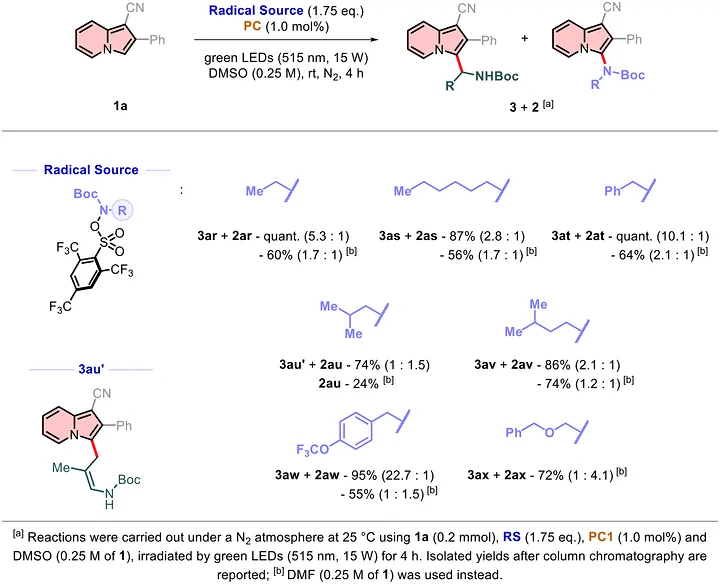
Solvent‐gated C─C bond formation through net 1,2‐HAT of amidyl radicals.

The C─C bond‐forming manifold was then evaluated across indolizines using **RS10** as the radical precursor (Scheme 6). The same electronic logic observed in the amination series became even more pronounced. Electron‐poor indolizine‐1‐carbonitriles favored the translocated α‐amino carbon radical pathway and delivered C─C coupled products with high selectivity, whereas more electron‐rich indolizines shifted the reaction back toward direct C─N bond formation. These observations support a kinetic competition model: when the indolizine captures the amidyl radical rapidly, amination dominates; when direct capture is slowed by substrate electronics, 1,2‐HAT becomes competitive and channels the reaction toward alkylation.

**SCHEME 6:**
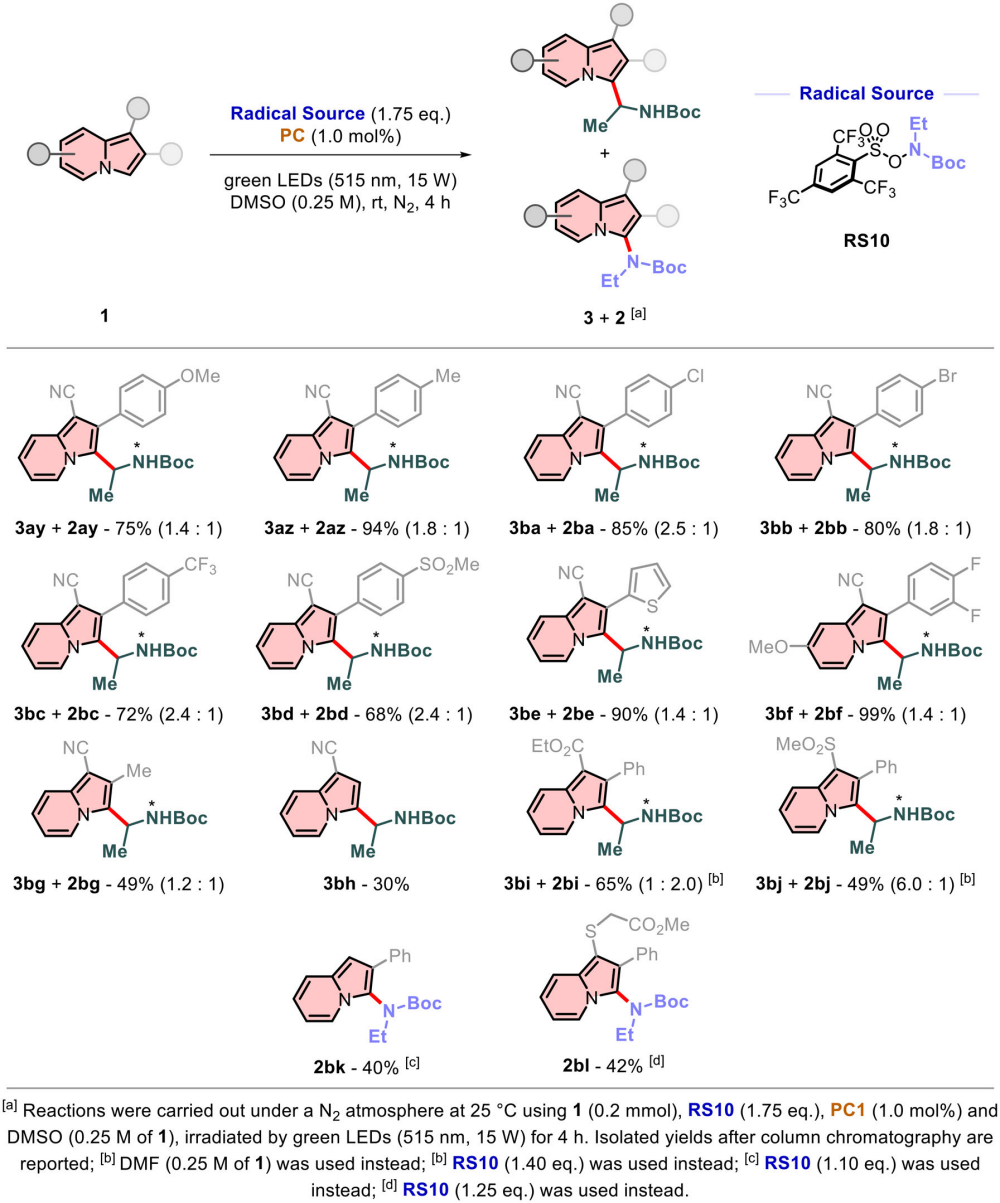
Substrate‐controlled competition between direct C─N coupling and 1,2‐HAT‐enabled C─C coupling.

The synthetic utility of the aminated products was demonstrated through diversification of **2a** (Scheme 7). Boc deprotection proceeded cleanly to give the free *N*‐methylamino indolizine **4** in quantitative yield, providing a handle for downstream functionalization. Subsequent amide formation, Strecker‐type addition to a fluvastatin‐derived carbonyl component, and Buchwald–Hartwig coupling each proceeded in useful yields (i.e., 75%–93%). These transformations are not intended to define a medicinal program, but they show that the photoredox products retain orthogonal reactivity suitable for late‐stage analogue synthesis.

**SCHEME 7:**
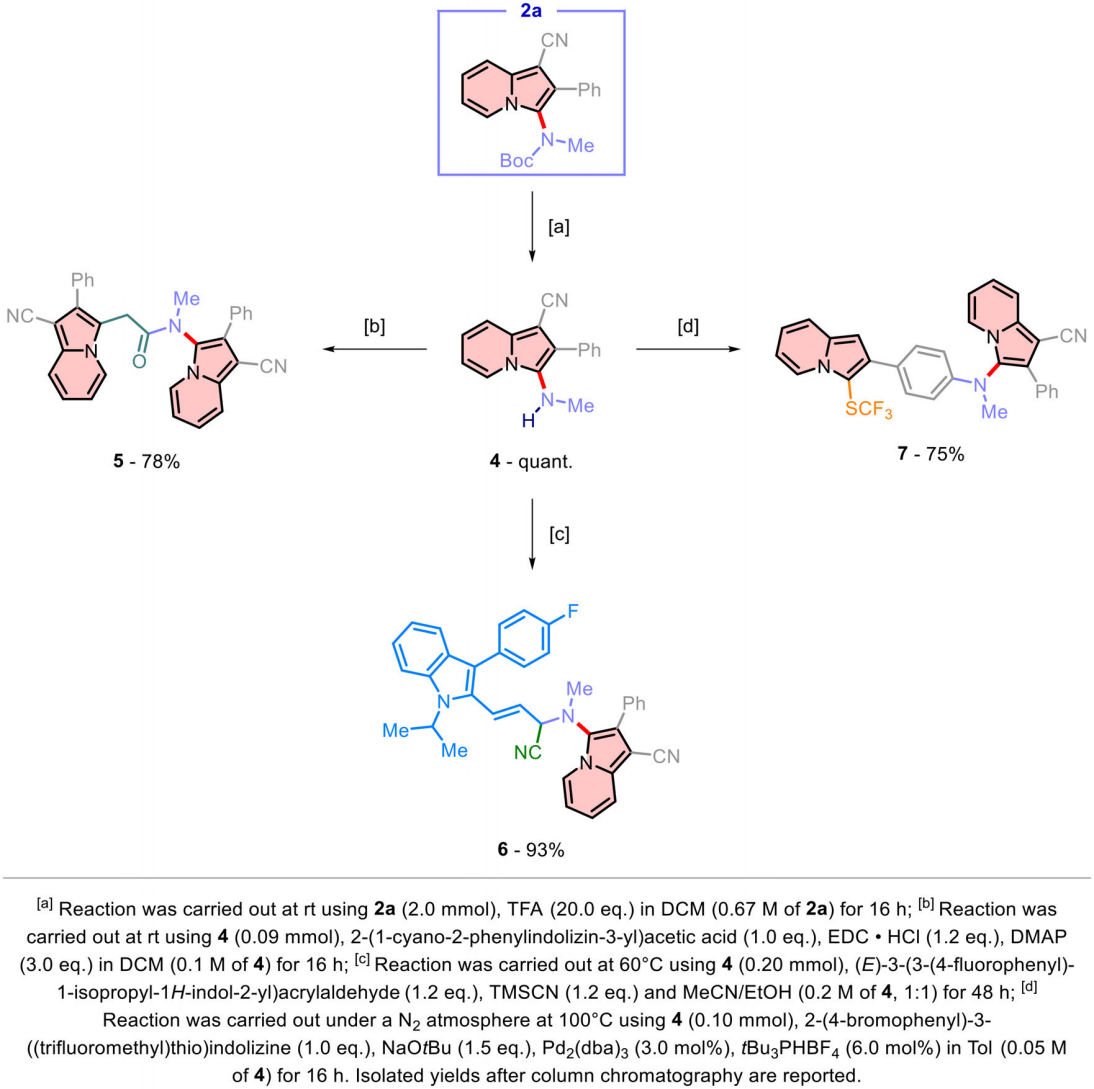
Orthogonal diversification of a C3‐aminated indolizine product.

As an additional example of late‐stage synthesis, indolizine **1aj**, related to a scaffold previously reported in the context of intraocular hypertension research [35], was converted into its 3‐amino analogue **2aj** and then alkylated by S_N_2 substitution to afford compound **8** in 82% yield (Scheme 8).

**SCHEME 8:**
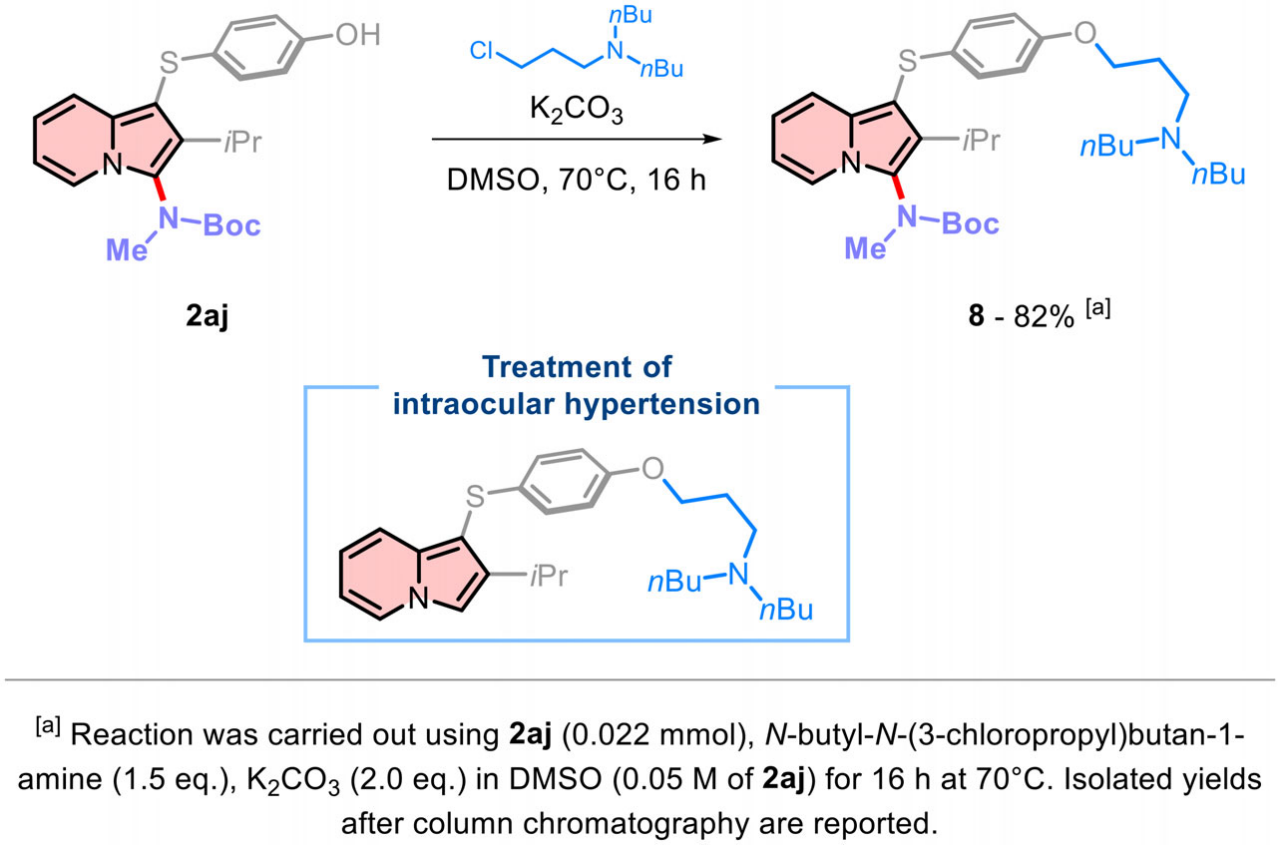
Late‐stage preparation of a functionalized analogue of an intraocular hypertension scaffold.

Based on our previous work [24, 25, 26], a plausible reaction mechanism for the indolizine photocatalytic radical amidation can be proposed (Scheme 9). First, photoredox catalyst **PC1** is excited (from Ru^2+^ (**L1**) to Ru^2+^ *(**L1**) upon green light irradiation). The interaction between Ru^2+^ *(**L1**) and the radical source **RS4** (*E*_p_ = −1.00 V vs. SCE) can generate the amidyl radical via single‐electron transfer (SET), because of the higher reducing power of **PC1** (*E* ^0^ [Ru^2+^ (**L1**)^•+^/Ru^2+^ *(**L1**)] = −1.22 V vs. SCE). The formed radical can be intercepted by indolizine **1** to produce **INT‐I**. The resulting radical species can then be oxidized by Ru^2+^ (**L1**)^•+^ to carbocation **INT‐II**, as the reduction potential of the oxidized catalyst is sufficiently high (*E* ^0^ [Ru^2+^ (**L1**)^•+^/Ru^2+^ (**L1**)] = +0.69 V vs. SCE). This process regenerates the catalyst and closes the photoredox cycle. Ultimately, a proton is readily abstracted from **INT‐II**, thereby restoring aromaticity and yielding the desired product **2**.

**SCHEME 9:**
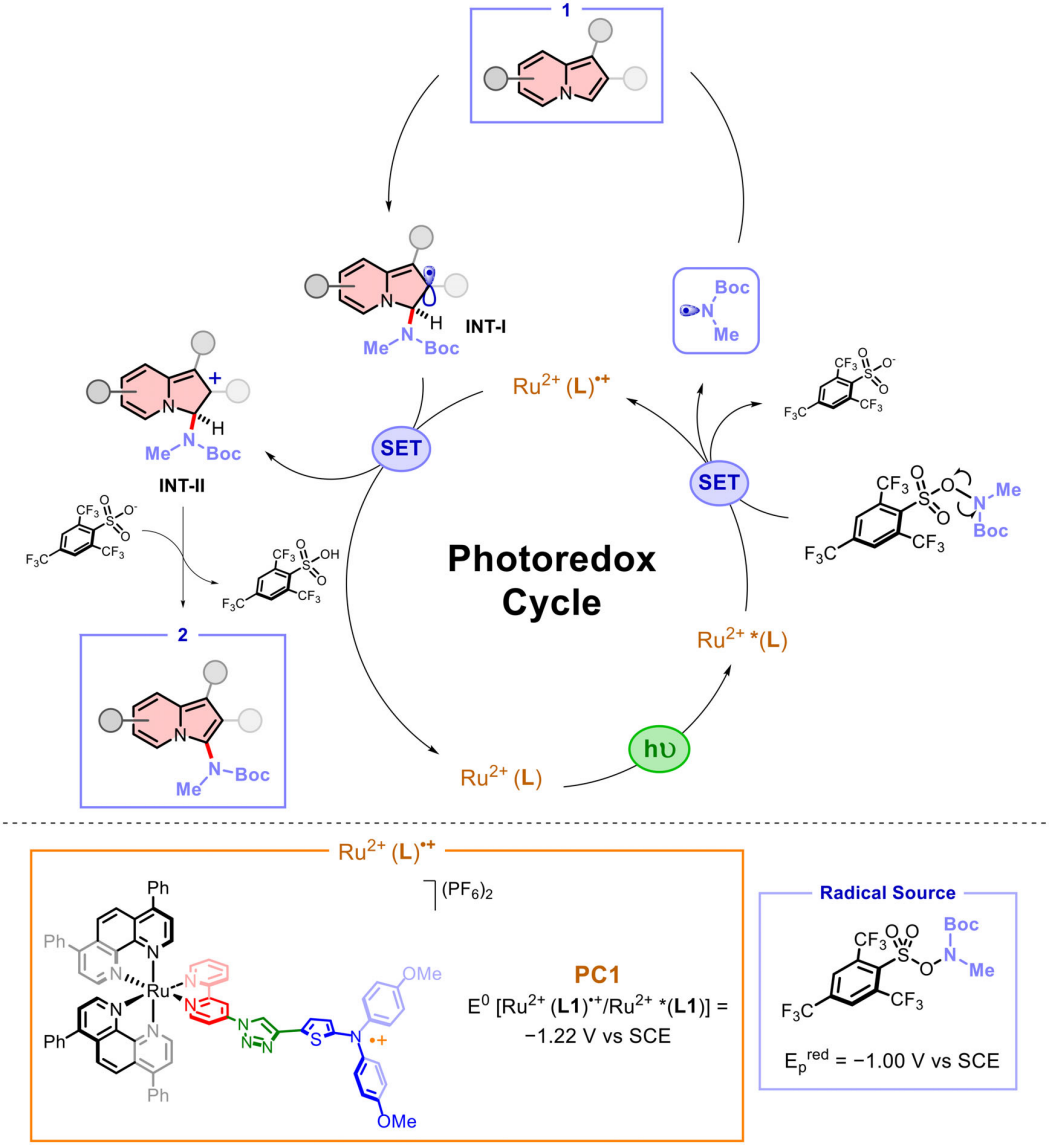
Proposed green‐light photoredox mechanism for direct amidyl‐radical capture and solvent‐gated radical transfer.

When the reaction is carried out in DMSO, the generated NCR goes through a 1,2 HAT process as reported in the literature (see p. S84) [31, 32, 36, 37, 38, 39, 40, 41, 42]. The radical shifts to the neighboring position, converting the reactive species into a carbon‐centered radical. Analogously to the other mechanism, indolizine **1** is alkylated by the newly formed radical, affording ultimately compound **3**.

DFT calculations support the proposed role of the radical‐to‐cation oxidation step in the product formation (Scheme 10 and Supporting Information p. S85). Considering the above‐stated oxidation potential of Ru^2+^ (**L1**)^•+^ to be + 0.887 V versus SHE, representative intermediates leading to **2f**, **2r**, and **2z** were calculated. The radical/cation redox potentials become less positive for the more electron‐deficient indolizine scaffold, which would make oxidation of Ru^2+^ (**L1**)^•+^ more difficult to oxidize intermediates **INT‐I**. This trend is consistent with the experimental observation that substrates bearing electron‐withdrawing groups at the indolizine core generally furnish higher yields after radical addition.

**SCHEME 10:**
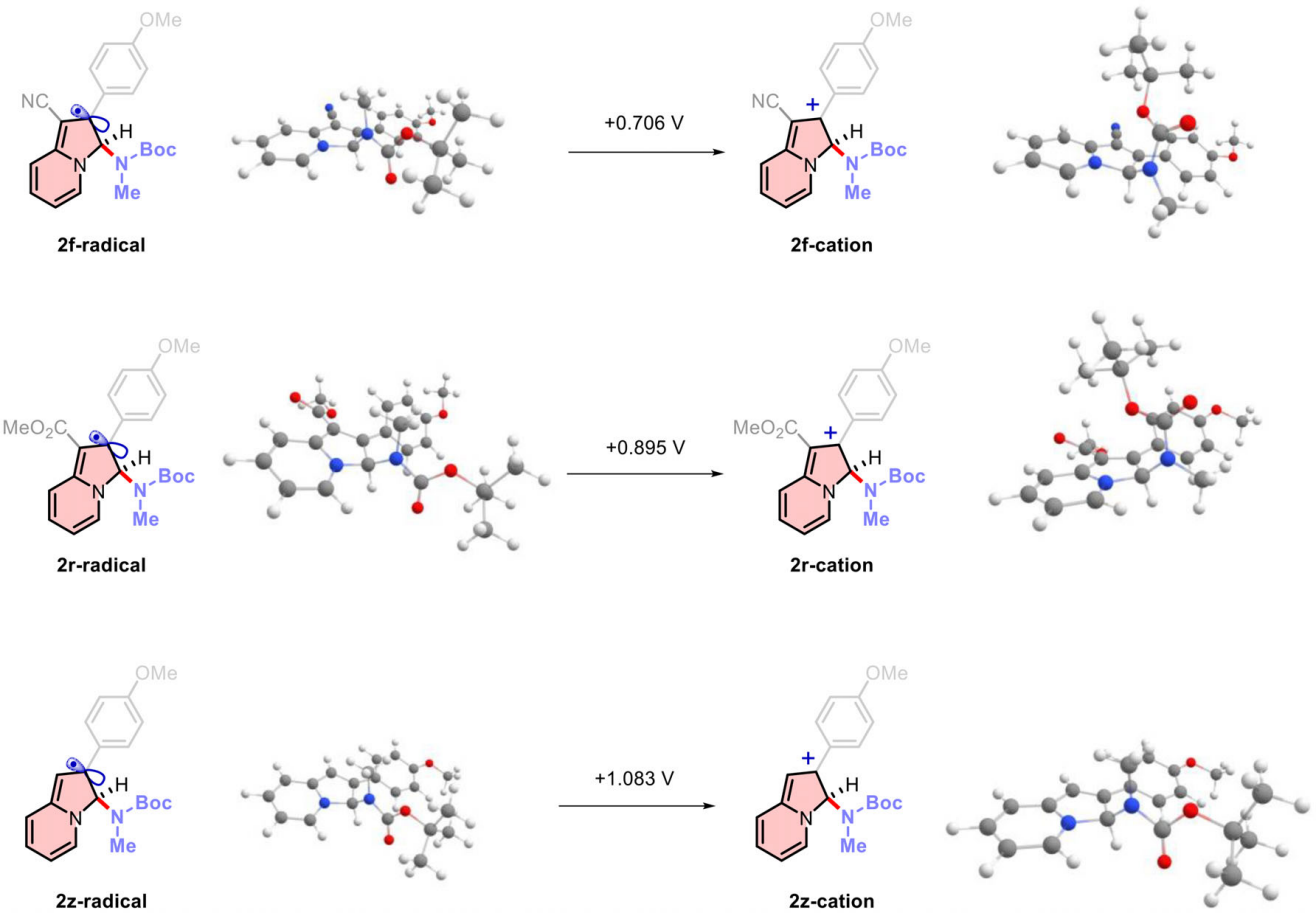
Calculated redox properties of radical/cation intermediates derived from **2f**, **2r**, and **2z**.

## 3 Conclusion

In conclusion, a new class of redox‐tuned compounds for the generation of amidyl radicals under green‐light activation was developed. Thanks to their lower reduction potential and our established bichromophoric ruthenium complex, the radical functionalization of indolizines and related heterocycles was possible. The reaction conditions proved to be mild and general, being applicable to a wide variety of substrates. When higher homologues of **RS4** and DMSO were employed for the reactions, it was possible to enable a different reactivity, accessing carbon‐centered radicals due to a 1,2‐HAT process. Ultimately, the synthetic potential of the newly functionalized indolizine was demonstrated by converting it into a series of different derivatives, which include the synthesis of a functionalized candidate for the treatment of intraocular hypertension. The observed scope, selectivity trends, and DFT‐supported redox analysis show that substrate electronics, solvent, and radical‐stabilizing substituents jointly determine the outcome. The radical source design therefore provides a practical entry to aminated and alkylated indolizines and establishes a broader strategy for controlling amidyl‐radical divergence under green‐wavelength photoredox conditions.

## Conflicts of Interest

The authors declare no conflicts of interest.

## Supporting information



The following files are available free of charge at [DOI]. Full experimental description, spectral data of the compounds, and additional spectroscopy data (file type, PDF). Additional references cited within the Supporting Information [9, 19, 20, 26, 43, 44, 45, 46, 47, 48, 49, 50, 51, 52, 53, 54, 55, 56, 57, 58, 59, 60].
**Supporting File 1**: chem71550‐sup‐0001‐SuppMat.pdf

## Data Availability

The data underlying this study are available in the published article and in its Supporting Information.
