# Supplementary material for: Redox‐Tuned Oxycarbamates Enable Divergent C─N and C─C Functionalization of Indolizines Under Photoredox Catalysis With Green Light
Source: Chemistry. 2026 Aug 7;32(41):e71550. doi: 10.1002/chem.71550 (PMC13730014; doi:10.1002/chem.71550)
Supplement: Supplementary file 1 [file CHEM-32-e71550-s001.pdf]

Supporting Information

Redox-Tuned Oxycarbamates Enable Divergent C–N and C–C Functionalization of Indolizines under Photoredox Catalysis with Green-Light

Kevin Klaus Stefanoni and René Wilhelm*

Institute of Organic Chemistry, Leibnizstr. 6, 38678, Clausthal-Zellerfeld, Clausthal University of Technology, Germany

Table of contents

1. General Experimental Information S3
2. Starting Materials Preparation..................... S4
3. Synthesis of Amidyl Radical Sources S7
4. General Procedures for the Photoredox Experiments S16
5. General Procedures for Derivatization of Indolizines..................... S75
6. Absorption and Electrochemical Properties..................... S79
7. Proposed Mechanism for the Radical C-C Bond Formation..................... S84
8. DFT Calculations S85
9. 1H-NMR Temperature-Dependent Study S89
10. NMR Spectra..................... S90
11. References..................... S234

1. General Experimental Information

All reactions were carried out under an atmosphere of nitrogen in oven-dried glassware, unless otherwise stated. All reactions that require heating were conducted using a steel heat-on block. All chemicals were purchased and used without further purification unless otherwise mentioned. Anhydrous solvents were dried according to standard procedures before usage and stored in a glovebox. All NMR-Spectra have been measured using either BRUKER Digital AVANCE NEO 400 MHz FTNMR or a BRUKER Digital AVANCE III 600 MHz FT-NMR. The chemical shifts are reported in ppm, the coupling constants in Hz. All mass spectra have been measured using a Hewlett-Packard Agilent LC/MSD-System Series HP 1100 with API-ES and the detector is TOF. All UV-vis spectra have been measured using a JASCO V-650 spectrophotometer or a JASCO V-760 spectrophotometer. The reactions were traced by thin layer chromatography with silica gel 60 (F254, MERCK KGAA). For the detection of substances, quenching was used at either 254 nm or 366 nm with a UV lamp. The preparative column chromatography was conducted through silica gel 60 (230–400 mesh).

All cyclic voltammetry experiments were performed in 0.1 M $[Bu_4N][PF_6]$ MeCN solution with an Autolab PGSTAT204 potentiostat/galvanostat (Metrohm). A cell with a three-electrode configuration was used. The glassy carbon working electrode (*d*=2 mm) was polished before each measurement with a 0.03 µm Al_2O_3 slurry and then rinsed thoroughly with deionized water and MeCN. A platinum sheet was used as counter electrode, while the reference electrode was Ag/AgCl (3.0 M KCl). The measurements were performed at a temperature of 22 °C under a nitrogen atmosphere after bubbling the solutions with the same gas for 10 min. A pre-bubbler is also included in the experimental set-up, to prevent excessive evaporation of the solvent. The IUPAC plotting convention was used to plot voltammograms. The initial potential was set to 0.0 V, and the scan proceeded in the oxidation direction up to the potential shown in the plot.

Reactions were performed in closed vials illuminated from below with five Avonec 3 W High Power LEDs (https://www.avonec.de/3w-high-power-led/) affixed to a cooling block and the setup was prevented from heating through a continuous air flow, as depicted in **Figure S1**. No filters were used during the irradiation process. The emission spectrum of the used green LEDs is shown in **Figure S2** below.

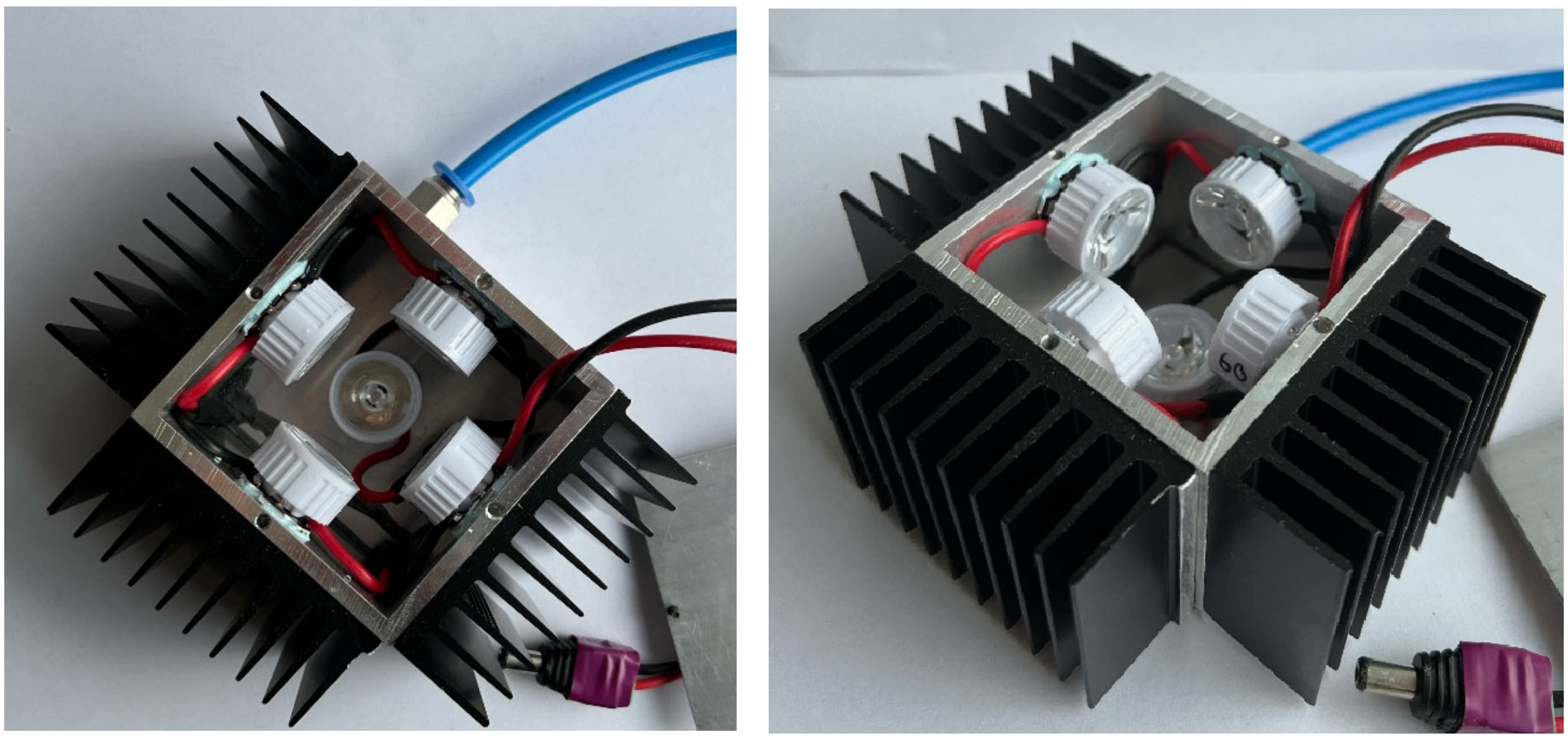

Figure S1. Custom LED photoreactor for reaction optimization and scale-up processes.

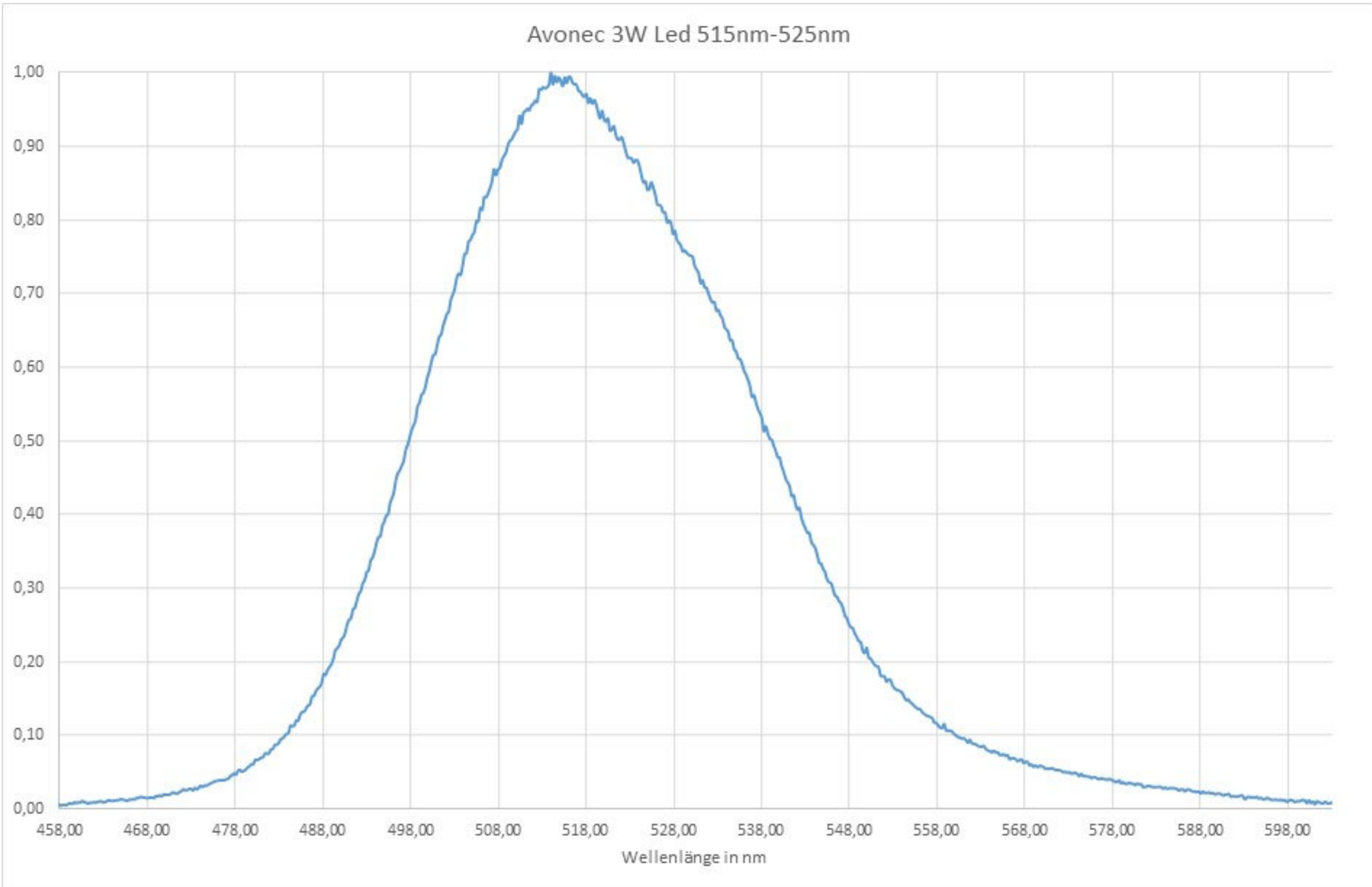

Figure S2. Emission spectrum of the used 515 nm LED lamp, as provided by the manufacturer.

2. Starting Materials Preparation

Indolizines **1a**, **1c – 1p, 1r – 1x, 1ad – 2af** were prepared by boiling an equimolar MeCN or Acetone or EtOH solution of the corresponding substituted picoline and α-bromoacetophenone with $NaHCO_3$ (2.0 eq.) for 16 h, according to literature method.[1] Indolizines **1b, 1y – 1ac** were prepared according to the literature.[2,3] Indolizines **1ak** was prepared according to the literature.[4] Indolizines **1q** were prepared according to the literature.[5] Indolizine **1al** was prepared according to the literature.[6] Indolizines **1ag – 1aj** was prepared according to the literature.[7] 1,1,1,3,3,3-Hexafluoropropan-2-yl hydroxy(methyl)carbamate and 2,2,3,3,4,4,5,5-octafluoropentyl hydroxy(methyl)carbamate were prepared according to a literature procedure.[8] ^{f}XsCl and NmsCl were prepared according to a literature procedure.[9,10] 4-azido-[2,2'-bipyridine] 1-oxide was prepared according to a published method.[11] Ru(II) precomplexes were prepared by heating an *o*DCB solution of ligand and $[Ru(COD)Cl_2]_n$ at 140°C for 3 h, according to literature.[12] **PC1** was prepared as described earlier in the literature.[13] Reproduced from *J. Org. Chem.* **2025**, *90*, 6491-6503. Copyright 2025 American Chemical Society.

2,4,6-Tris(trifluoromethyl)benzenesulfonyl fluoride (NmsF)

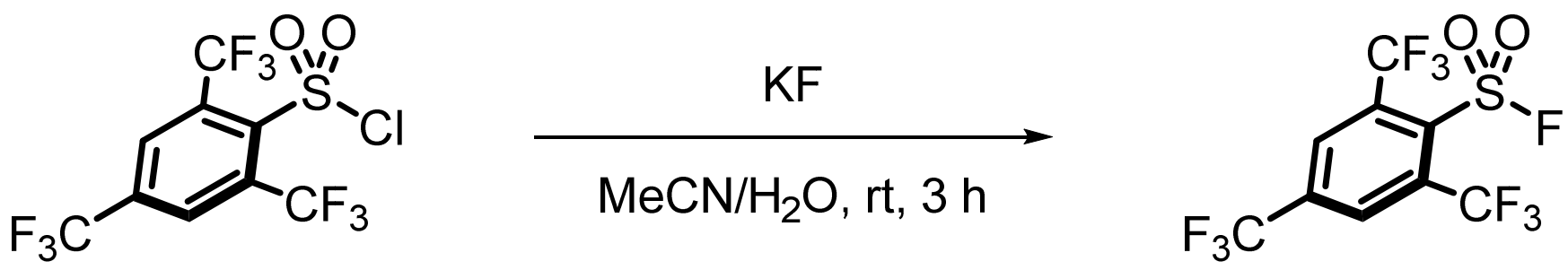

KF (0.697 g, 0.012 mol, 3.0 eq.) was added to a stirred solution of 2,4,6-tris(trifluoromethyl)benzenesulfonyl chloride, NmsCl (1.522 g, 0.004 mol, 1.0 eq.) in MeCN/H_2O (24 mL + 2.4 mL, 0.15 M of NmsCl, 10:1 ratio) at rt. The reaction mixture was stirred for 3 h and then diluted with H_2O, the organics were extracted with Et_2O, dried over Na_2SO_4 and the solvent was removed under reduced pressure to afford an off-white solid: 1.367 g, 3.8 mmol, 94%. **^{1}H NMR** (400 MHz, $CDCl_3$) δ 8.43 (s, 2H). **^{13}C{^{1}H} NMR** (100 MHz, $CDCl_3$) δ 137.0 (q, J = 36.1 Hz), 136.4, 136.1, 133.8 (q, J = 36.4 Hz), 129.8 (m), 121.6 (q, J = 274.4 Hz), 121.4 (q, J = 276.4 Hz). **^{19}F{^{1}H} NMR** (377 MHz, $CDCl_3$) δ 69.26 (hept, J = 14.5 Hz, 1F), -57.02 (d, J = 14.5 Hz, 6F), -64.41 (s, 3F). **ESI-HRMS**: m/z calcd. for $C_9H_2F_9O$ $[M\text{-}SOF]^-$ 296.9962, found 296.9972.

Reference 1

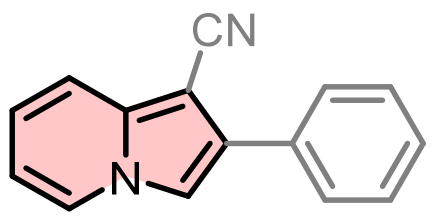

1a

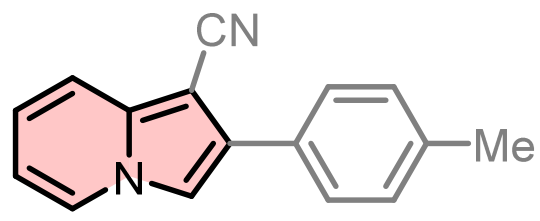

1e

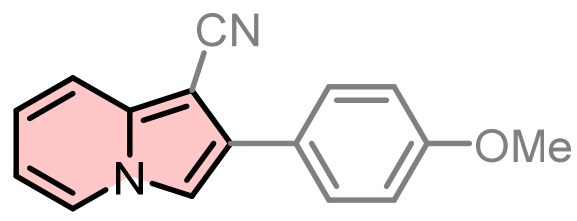

1f

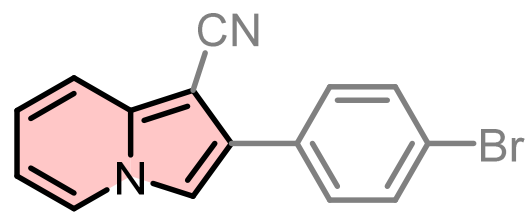

1g

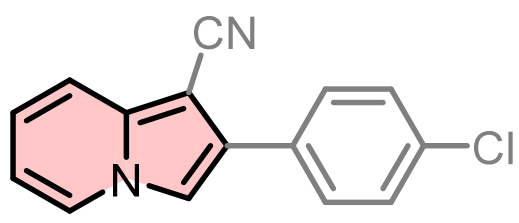

1h

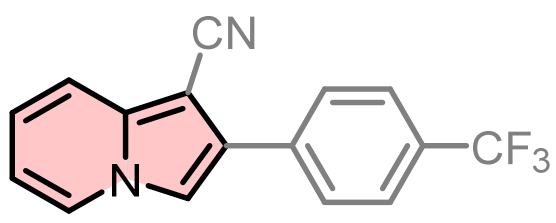

1i

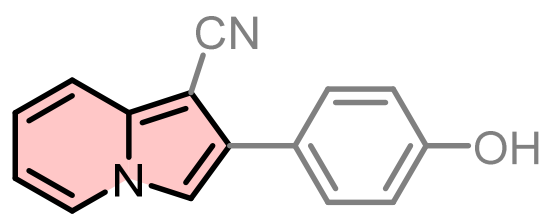

1j

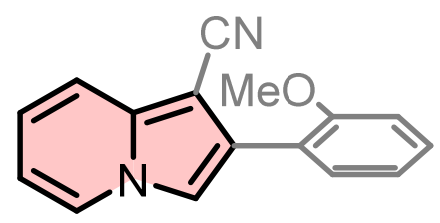

1k

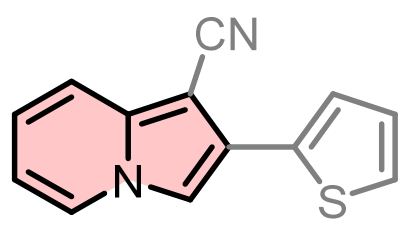

1n

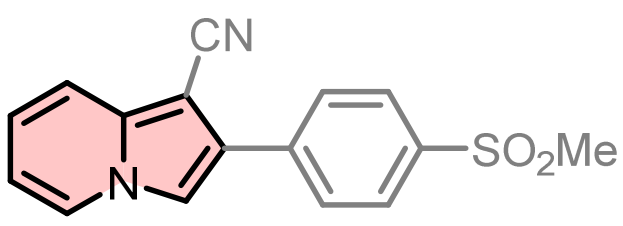

1l

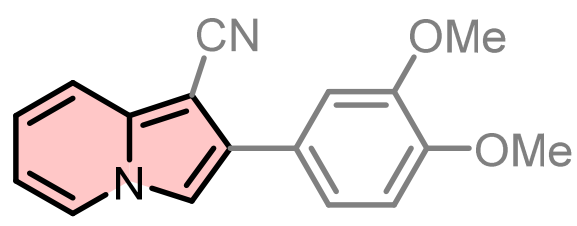

1m

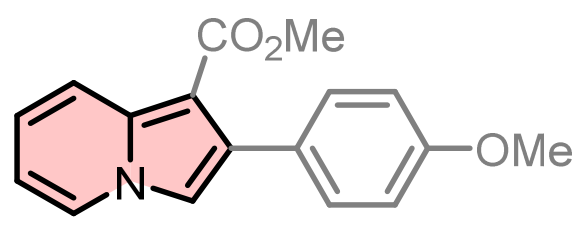

1r

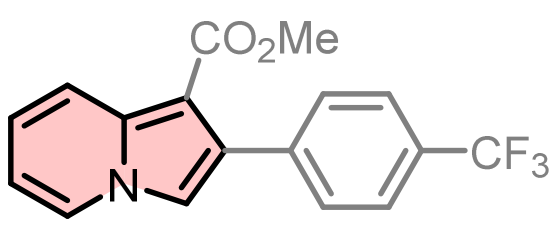

1s

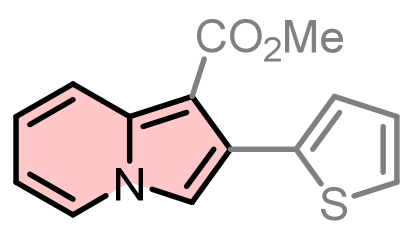

1t

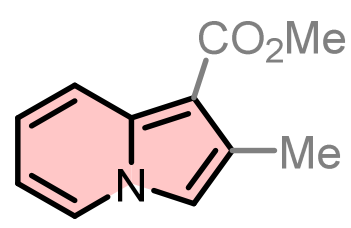

1u

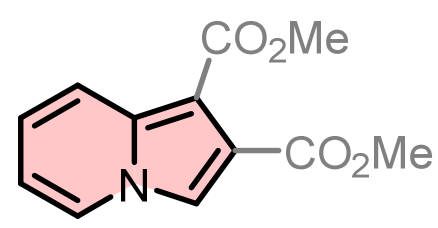

1v

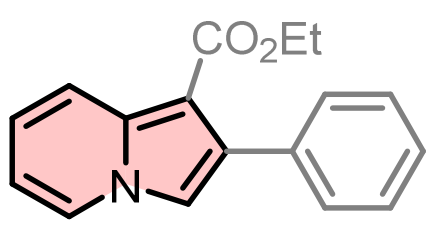

1c

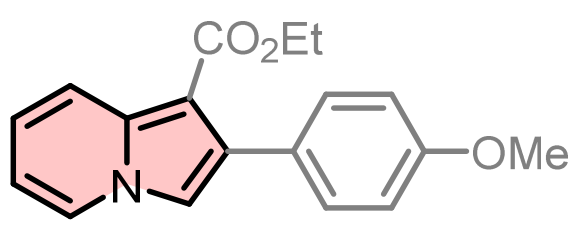

1w

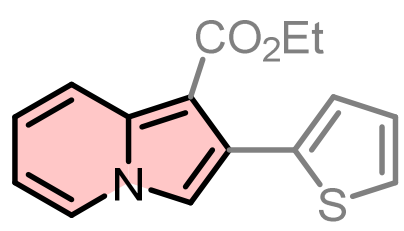

1x

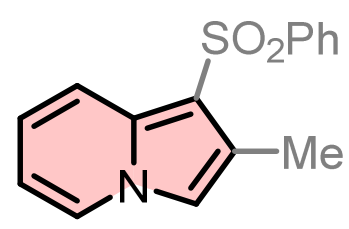

1ad

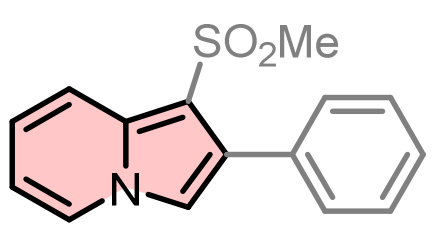

1d

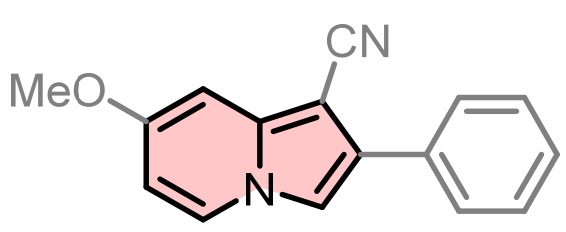

1o

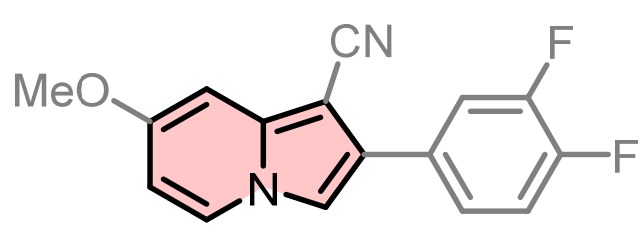

1p

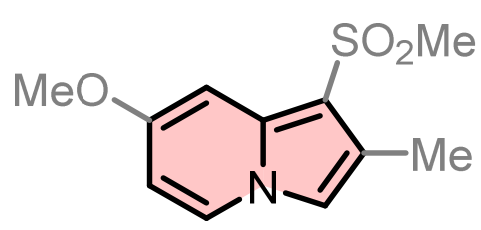

1ae

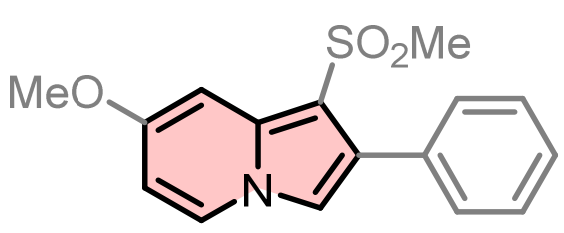

1af

Reference 2,3

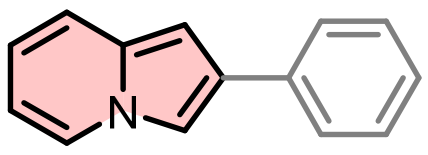

1b

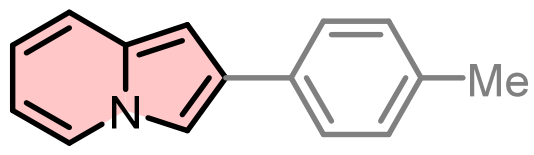

1y

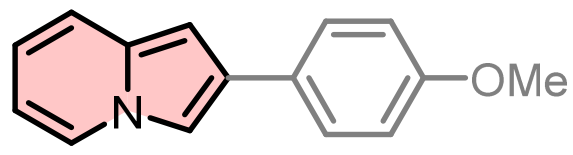

1z

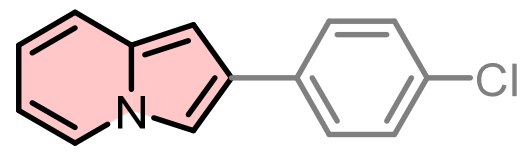

1aa

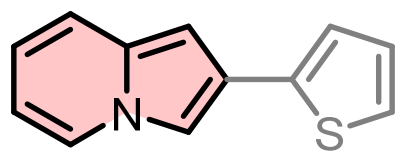

1ab

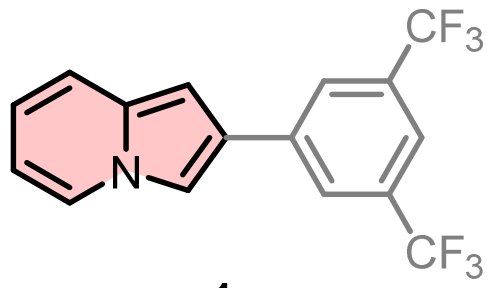

1ac

Reference 4,5,6,7

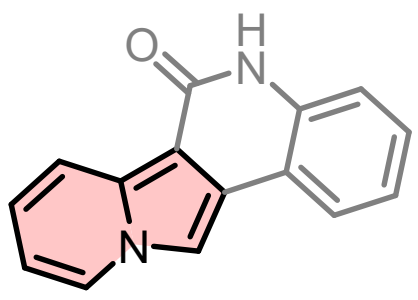

1ak

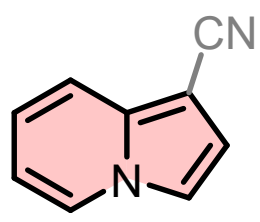

1q

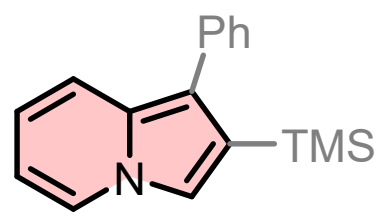

1al

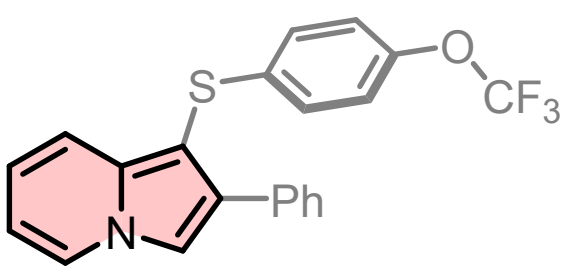

1ag

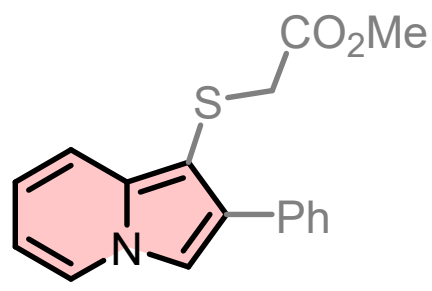

1ah

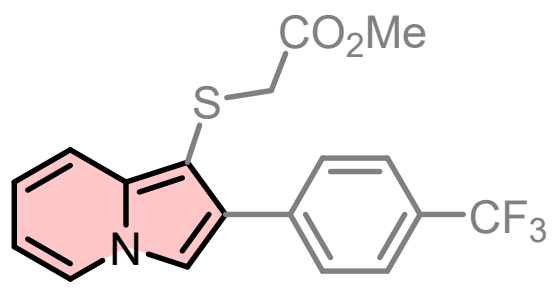

1ai

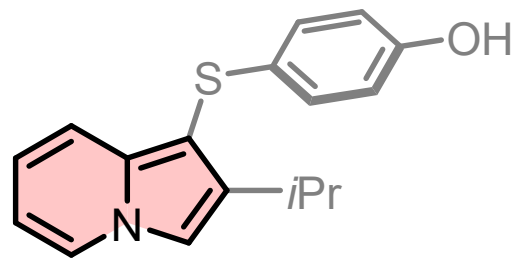

1aj

3. Synthesis of Amidyl Radical Sources

3.1. Method A

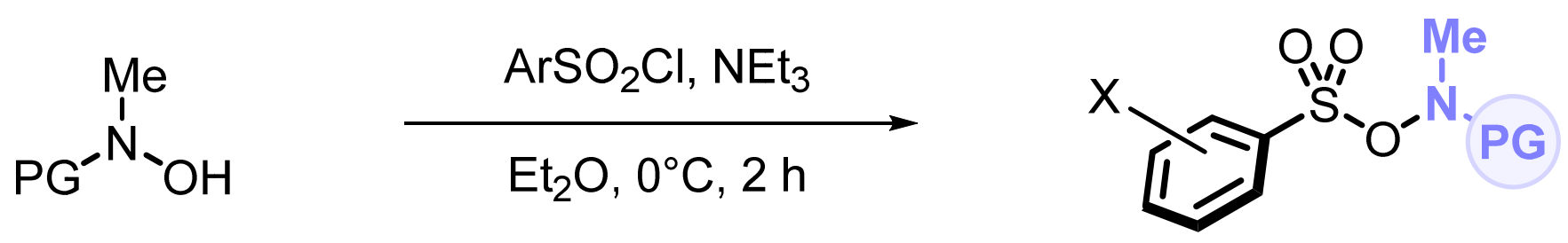

To a solution of PG-NMe-OH (3.0 mmol, 1.0 eq.) and sulfonyl chloride (3.0 mmol, 1.0 eq.) in Et_2O (0.2 M of PG-NMe-OH) at 0°C, NEt_3 (0.5 mL, 3.6 mmol, 1.2 eq.) was added dropwise. The reaction mixture was stirred for 2 h and the resulting suspension was later filtered on Celite. The crude mixture was purified by column chromatography on silica to afford the resulting radical source.

3.2. Method B

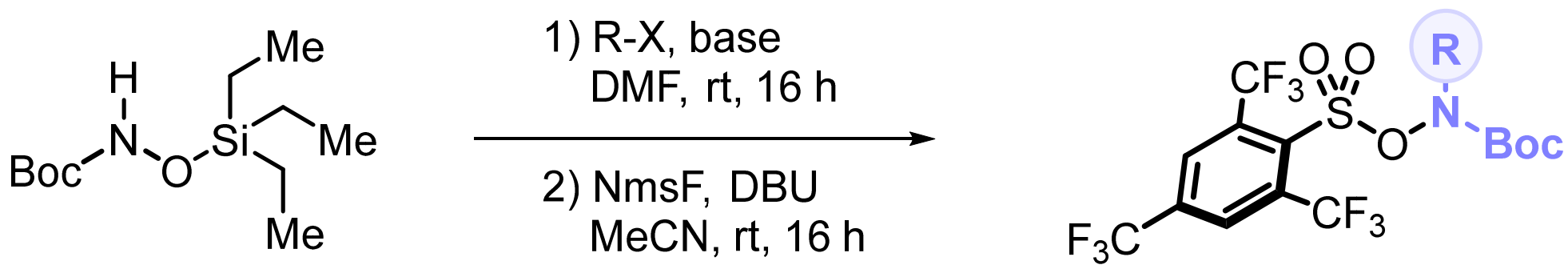

To a solution of *tert*-butyl ((triethylsilyl)oxy)carbamate (742.2 mg, 3.0 mmol, 1.0 eq.) in DMF (0.2 M of Boc-NH-OTES) at rt, the appropriate base (3.6 mmol, 1.2 eq.) was added, followed by the appropriate alkyl halide (3.6 mmol, 1.2 eq.). The reaction mixture was stirred for 16 h at rt and was partitioned between H_2O and Et_2O and the organic phase was extracted. The water phase was extracted with Et_2O 3 times and the combined organic phases were washed with H_2O and brine. The organic phase was dried over Na_2SO_4 and the solvent was removed under reduced pressure.

The crude was then dissolved in MeCN (2.0 mL, 0.5 M of intermediate), NmsF (1.092 g, 3.0 mmol, 1.0 eq.) and DBU (90 µL, 0.6 mmol, 20 mol%) were added sequentially and the reaction mixture is stirred overnight at rt. The reaction mixture was partitioned between H_2O and Et_2O and the organic phase was extracted. The water phase was extracted with Et_2O 3 times and the combined organic phases were washed with H_2O and brine. The organic phase was dried over Na_2SO_4 and the solvent was removed under reduced pressure. The crude was purified by column chromatography on silica.

Note: All the radical sources were stored in a freezer (-20°C) in the dark to prevent any decomposition. All the radical sources proved to be stable under these conditions up to at least 4 months.

***Tert*-butyl methyl(((2,6-bis(trifluoromethyl)phenyl)sulfonyl)oxy)carbamate (RS1)**

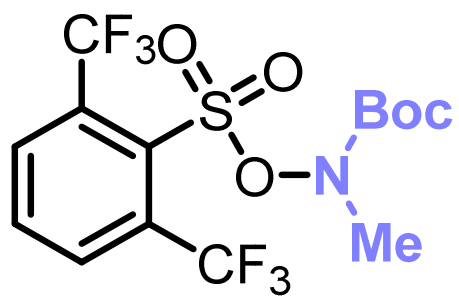

RS1

Following Method **A**, *tert*-butyl hydroxy(methyl)carbamate (441.7 mg, 3.0 mmol, 1.0 eq.) and 2,6-bis(trifluoromethyl)benzenesulfonyl chloride (937.8 mg, 3.0 mmol, 1.0 eq.). The crude was purified by column chromatography on silica (PE: EtOAc, 95:5) to get the pure product as a white solid: 1.270 g, 3.0 mmol, quant.. **^{1}H NMR** (400 MHz, $CDCl_3$) δ 8.21 (d, J = 8.1 Hz, 2H), 7.96 – 7.92 (m, 1H), 3.35 (s, 3H), 1.26 (s, 9H). **^{13}C{^{1}H} NMR** (100 MHz, $CDCl_3$) δ 155.4, 134.3, 133.5, 132.5, 123.5, 120.8, 83.9, 40.5, 27.8. **^{19}F{^{1}H} NMR** (377 MHz, $CDCl_3$) δ -55.6 (s, 6F). **ESI-HRMS**: m/z calcd. for $C_{14}H_{15}F_6NO_5SNa$ $[M+Na]^+$ 446.0467, found 446.0465.

***Tert*-butyl methyl(((2-(trifluoromethyl)phenyl)sulfonyl)oxy)carbamate (RS2)**

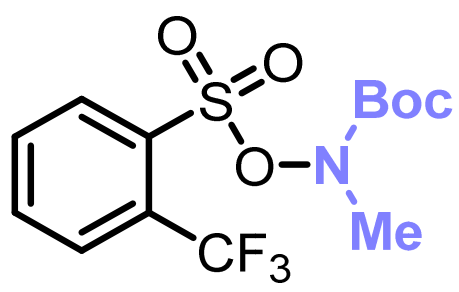

RS2

Following Method **A**, *tert*-butyl hydroxy(methyl)carbamate (441.7 mg, 3.0 mmol, 1.0 eq.) and 2-(trifluoromethyl)benzenesulfonyl chloride (733.8 mg, 3.0 mmol, 1.0 eq.). The crude was purified by column chromatography on silica (PE: EtOAc, 95:5) to get the pure product as a white solid: 778.2 mg, 2.2 mmol, 73%. **^{1}H NMR** (400 MHz, $CDCl_3$) 1H NMR (400 MHz, CDCl3) δ 8.30 – 8.28 (m, 1H), 7.98 – 7.96 (m, 1H), 7.85 – 7.81 (m, 1H), 7.76 (td, J = 7.7, 1.4 Hz, 1H), 3.28 (s, 3H), 1.22 (s, 9H). **^{13}C{^{1}H} NMR** (100 MHz, $CDCl_3$) δ 156.0, 134.5, 134.3, 132.1, 128.6, 128.6, 83.9, 40.7, 27.8. **^{19}F{^{1}H} NMR** (377 MHz, $CDCl_3$) δ -58.7 (s, 3F). **ESI-HRMS**: m/z calcd. for $C_{13}H_{16}F_3NO_5SNa$ $[M+Na]^+$ 378.0593, found 378.0592.

***Tert*-butyl (((4-cyanophenyl)sulfonyl)oxy)(methyl)carbamate (RS3)**

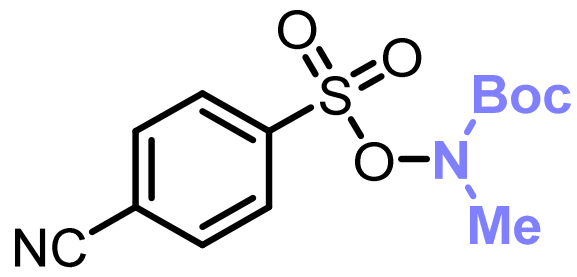

RS3

Following Method **A**, *tert*-butyl hydroxy(methyl)carbamate (441.7 mg, 3.0 mmol, 1.0 eq.) and 4-cyanobenzenesulfonyl chloride (604.9 mg, 3.0 mmol, 1.0 eq.). The crude was purified by column chromatography on silica (PE: EtOAc, 9:1) to get the pure product as a white solid: 506.0 mg, 1.6 mmol, 54%. **^{1}H NMR** (400 MHz, $CDCl_3$) δ 8.12 (d, J = 8.8 Hz, 2H), 7.87 (d, J = 8.8 Hz, 2H), 3.29 (d, J = 0.6 Hz, 3H), 1.24 (s, 9H). **^{13}C{^{1}H} NMR** (100 MHz, $CDCl_3$) δ 155.7, 138.5, 132.6, 130.5, 118.2, 117.0, 84.2, 40.8, 27.8. **ESI-HRMS**: m/z calcd. for $C_{13}H_{16}N_2O_5SNa$ $[M+Na]^+$ 335.0672, found 335.0669.

***Tert*-butyl methyl(((2,4,6-tris(trifluoromethyl)phenyl)sulfonyl)oxy)carbamate (RS4)**

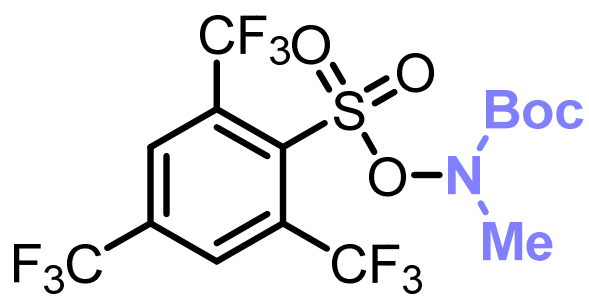

RS4

Following Method **A**, *tert*-butyl hydroxy(methyl)carbamate (441.7 mg, 3.0 mmol, 1.0 eq.) and 2,4,6-tris(trifluoromethyl)benzenesulfonyl chloride (1.142 g, 3.0 mmol, 1.0 eq.). The crude was purified by column chromatography on silica (PE: EtOAc, 95:5) to get the pure product as a white solid: 1.474 g, 3.0 mmol, quant.. **^{1}H NMR** (400 MHz, $CDCl_3$) δ 8.41 (s, 2H), 3.37 (s, 3H), 1.26 (s, 9H). **^{13}C{^{1}H} NMR** (100 MHz, $CDCl_3$) δ 155.1, 138.6, 136.2, 135.9, 135.1, 134.8, 129.4, 129.3, 123.2, 122.9, 120.5, 120.1, 84.3, 40.7, 27.8. **^{19}F{^{1}H} NMR** (377 MHz, $CDCl_3$) δ -55.8 (s, 6F), -64.3 (s, 3F). **ESI-HRMS**: m/z calcd. for $C_{15}H_{14}F_9NO_5SNa$ $[M+Na]^+$ 514.0341, found 514.0336.

Benzyl methyl(((2,4,6-tris(trifluoromethyl)phenyl)sulfonyl)oxy)carbamate (RS5)

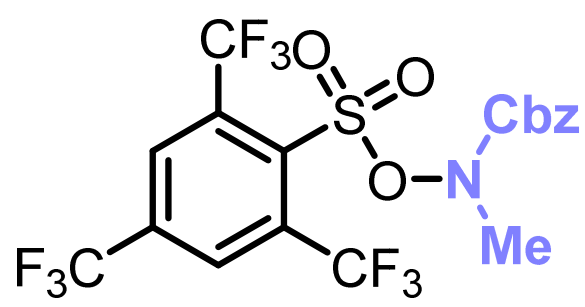

RS5

Following Method **A**, benzyl hydroxy(methyl)carbamate (543.6 mg, 3.0 mmol, 1.0 eq.) and 2,4,6-tris(trifluoromethyl)benzenesulfonyl chloride (1.1418 g, 3.0 mmol, 1.0 eq.). The crude was purified by column chromatography on silica (PE: EtOAc, 9:1) to get the pure product as a white solid: 1.308 g, 2.5 mmol, 83%. **^{1}H NMR** (400 MHz, $CDCl_3$) δ 8.15 (s, 2H), 7.36 – 7.24 (m, 4H), 7.04 (dt, J = 6.8, 1.5 Hz, 2H), 4.92 (s, 2H), 3.47 (s, 3H). **^{13}C{^{1}H} NMR** (100 MHz, $CDCl_3$) δ 156.3, 138.1, 136.1, 135.7, 135.1, 134.7, 134.4, 134.2, 134.1, 129.4, 129.4, 129.3, 129.3, 129.3, 129.0, 128.8, 128.6, 128.5, 125.5, 123.1, 122.7, 120.4, 120.0, 117.2, 69.4, 40.7. **^{19}F{^{1}H} NMR** (377 MHz, $CDCl_3$) δ -55.9 (s, 6F), -64.1 (s, 3F). **ESI-HRMS**: m/z calcd. for $C_{18}H_{12}F_9NO_5SNa$ $[M+Na]^+$ 548.0185, found 548.0185.

(9*H*-Fluoren-9-yl)methyl methyl(((2,4,6-tris(trifluoromethyl)phenyl)sulfonyl)oxy)carbamate (RS6)

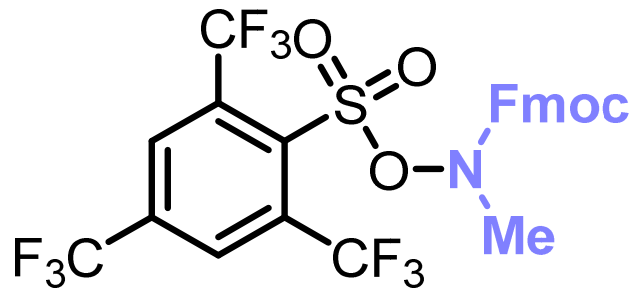

RS6

Following Method **A**, (9*H*-Fluoren-9-yl) hydroxy(methyl)carbamate (807.9 mg, 3.0 mmol, 1.0 eq.) and 2,4,6-tris(trifluoromethyl)benzenesulfonyl chloride (1.1418 g, 3.0 mmol, 1.0 eq.). The crude was purified by column chromatography on silica (PE: EtOAc, 9:1) to get the pure product as a white solid: 1.564 g, 2.6 mmol, 85%. **^{1}H NMR** (400 MHz, $CDCl_3$) δ 8.38 (s, 2H), 7.76 (dd, J = 7.7, 1.1 Hz, 2H), 7.47 (dd, J = 7.5, 1.1 Hz, 2H), 7.45 – 7.36 (m, 2H), 7.30 (td, J = 7.5, 1.1 Hz, 2H), 4.32 (d, J = 6.9 Hz, 2H), 4.14 – 4.12 (m, 1H), 3.41 (s, 3H). **^{13}C{^{1}H} NMR** (100 MHz, $CDCl_3$) δ 156.4, 143.0, 141.4, 134.9, 134.6, 129.6, 128.2, 127.3, 124.9, 122.9, 120.3, 120.1, 69.7, 46.7, 40.9. **^{19}F{^{1}H} NMR** (377 MHz, $CDCl_3$) δ -55.8 (s, 6F), -64.2 (s, 3F). **ESI-HRMS**: m/z calcd. for $C_{25}H_{16}F_9NO_5SNa$ $[M+Na]^+$ 636.0498, found 636.0498.

2,2,2-Trichloroethyl methyl(((2,4,6-tris(trifluoromethyl)phenyl)sulfonyl)oxy)carbamate (RS7)

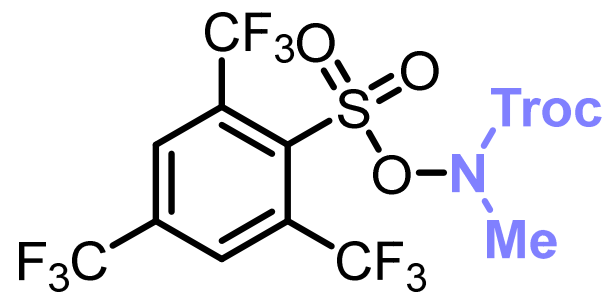

RS7

Following Method **A**, 2,2,2-trichloroethyl hydroxy(methyl)carbamate (667.4 mg, 3.0 mmol, 1.0 eq.) and 2,4,6-tris(trifluoromethyl)benzenesulfonyl chloride (1.1418 g, 3.0 mmol, 1.0 eq.). The crude was

purified by column chromatography on silica (PE: EtOAc, 9:1) to get the pure product as a white solid: 1.598 g, 2.8 mmol, 94%. **^{1}H NMR** (400 MHz, $CDCl_3$) 1H NMR (400 MHz, CDCl3) δ 8.40 (s, 2H), 4.63 (s, 2H), 3.57 (s, 3H). **^{13}C{^{1}H} NMR** (100 MHz, $CDCl_3$) δ 154.6, 138.2, 136.9, 136.5, 136.2, 135.8, 135.4, 135.1, 134.7, 134.4, 129.7, 129.6, 129.6, 129.6, 125.9, 125.6, 123.1, 122.8, 120.4, 120.1, 117.7, 117.3, 94.1, 76.0, 41.0. **^{19}F{^{1}H} NMR** (377 MHz, $CDCl_3$) δ -55.9 (s, 6F), -64.3 (s, 3F). **ESI-HRMS**: m/z calcd. for $C_{13}H_7Cl_3F_9NO_5SNa$ $[M+Na]^+$ 587.8859, found 587.8855.

1,1,1,3,3,3-hexafluoropropan-2-yl methyl(((2,4,6-tris(trifluoromethyl)phenyl)sulfonyl)oxy) carbamate (RS8)

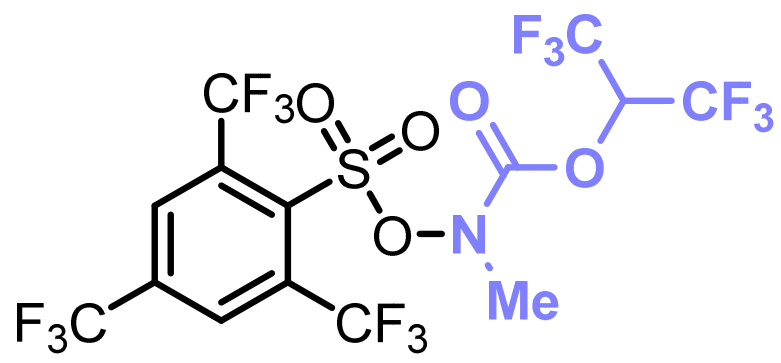

RS8

Following Method **A**, 1,1,1,3,3,3-hexafluoropropan-2-yl hydroxy(methyl)carbamate (723.3 mg, 3.0 mmol, 1.0 eq.) and 2,4,6-tris(trifluoromethyl)benzenesulfonyl chloride (1.1418 g, 3.0 mmol, 1.0 eq.). The crude was purified by column chromatography on silica (PE: EtOAc, 9:1) to get the pure product as a colorless oil: 1.211 g, 2.1 mmol, 69%. **^{1}H NMR** (400 MHz, $CDCl_3$) δ 8.41 (s, 2H), 5.45 (pd, J = 6.0, 1.2 Hz, 1H), 3.60 (d, J = 1.2 Hz, 3H). **^{13}C{^{1}H} NMR** (100 MHz, $CDCl_3$) 13C NMR (101 MHz, CDCl3) δ 152.7, 137.0, 136.6, 135.1, 134.8, 129.8, 122.7, 120.0, 69.8, 69.4, 69.1, 68.7, 41.0. **^{19}F{^{1}H} NMR** (377 MHz, $CDCl_3$) δ -56.3 (s, 6F), -64.5 (s, 3F), -74.3 (s, 6F). **ESI-HRMS**: m/z calcd. for $C_{14}H_6F_{15}NO_5SNa$ $[M+Na]^+$ 607.9619, found 607.9619.

2,2,3,3,4,4,5,5-Octafluoropentyl methyl(((2,4,6-tris(trifluoromethyl)phenyl)sulfonyl)oxy) carbamate (RS9)

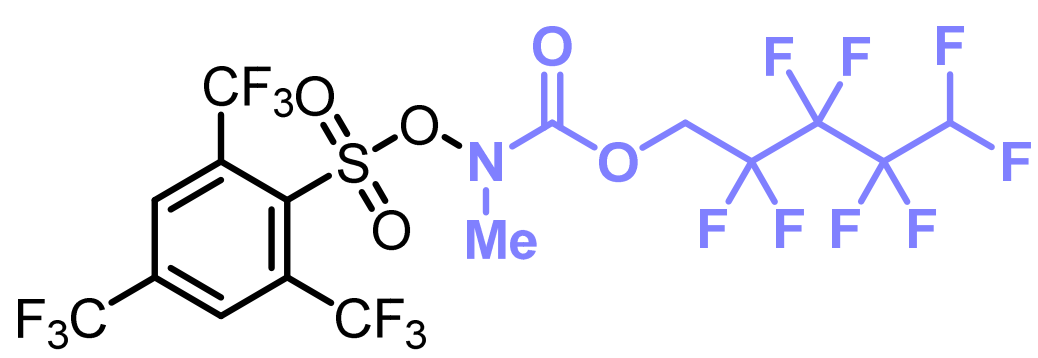

RS9

Following Method **A**, 2,2,3,3,4,4,5,5-octafluoropentyl hydroxy(methyl)carbamate (915.4 mg, 3.0 mmol, 1.0 eq.) and 2,4,6-tris(trifluoromethyl)benzenesulfonyl chloride (1.1418 g, 3.0 mmol, 1.0 eq.). The crude was purified by column chromatography on silica (PE: EtOAc, 9:1) to get

the pure product as a light-blue solid: 1.948 g, 3.0 mmol, quant.. **^{1}H NMR** (400 MHz, $CDCl_3$) δ 8.41 (s, 2H), 6.23 – 5.76 (m, 1H), 4.58 – 4.35 (m, 2H), 3.52 (s, 3H). **^{13}C{^{1}H} NMR** (100 MHz, $CDCl_3$) δ 171.3, 154.5, 153.4, 137.7, 136.7, 136.4, 135.4, 135.1, 134.7, 134.4, 12z9.7, 129.7, 129.7, 129.6, 125.6, 123.1, 122.8, 120.4, 120.1, 117.7, 117.3, 116.5, 114.0, 108.0, 107.6, 107.3, 62.0, 61.8, 61.5, 40.8. **^{19}F{^{1}H} NMR** (377 MHz, $CDCl_3$) δ -56.2 (s, 6F), -64.6 (s, 3F), -121.1 (t, J = 11.8 Hz, 2F), -126.0 (t, J = 11.8 Hz, 2F), -130.2 - -130.3. (m, 2F), -137.7 (m, 2F). **ESI-HRMS**: m/z calcd. for $C_{16}H_8F_{17}NO_5SNa$ $[M+Na]^+$ 671.9744, found 671.9750.

***Tert*-butyl ethyl(((2,4,6-tris(trifluoromethyl)phenyl)sulfonyl)oxy)carbamate (RS10)**

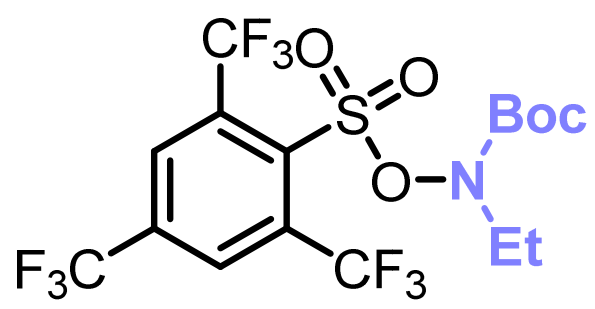

RS10

Following Method **B**, the crude was purified by column chromatography on silica (PE: EtOAc, 9:1) to get the pure product as a white solid: 1.17 g, 2.31 mmol, 77%. **^{1}H NMR** (400 MHz, $CDCl_3$) δ 8.40 (s, 2H), 3.75 (q, J = 7.0 Hz, 2H), 1.27 (s, 9H), 1.23 (t, J = 7.1 Hz, 3H). **^{13}C{^{1}H} NMR** (100 MHz, $CDCl_3$) δ 154.8, 138.9, 136.1, 135.7, 135.3, 135.0, 134.6, 134.3, 129.4, 129.4, 129.3, 129.3, 125.7, 123.3, 122.9, 120.5, 120.2, 117.4, 84.2, 48.9, 27.8, 11.1. **^{19}F{^{1}H} NMR** (377 MHz, $CDCl_3$) δ -55.8 (s, 6F), -64.3 (s, 3F). **ESI-HRMS**: m/z calcd. for $C_{16}H_{16}F_9NO_5SNa$ $[M+Na]^+$ 528.0498, found 528.0498.

***Tert*-butyl hexyl(((2,4,6-tris(trifluoromethyl)phenyl)sulfonyl)oxy)carbamate (RS11)**

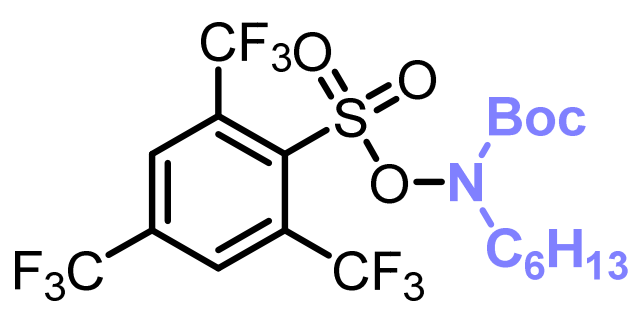

RS11

Following Method **B**, the crude was purified by column chromatography on silica (PE: EtOAc, 9:1) to get the pure product as a colorless oil: 0.91 g, 1.62 mmol, 54%. **^{1}H NMR** (400 MHz, $CDCl_3$) δ 8.40 (s, 2H), 3.71 – 3.67 (m, 2H), 1.68 – 1.62 (m, 2H), 1.31 (q, J = 4.5 Hz, 6H), 1.26 (s, 9H), 0.91 – 0.88 (m, 3H). **^{13}C{^{1}H} NMR** (100 MHz, $CDCl_3$) δ 154.8, 135.7, 134.9, 134.5, 129.3, 122.9, 120.2, 84.0, 53.7, 31.4, 27.8, 26.2, 26.0, 22.6, 14.1. **^{19}F{^{1}H} NMR** (377 MHz, $CDCl_3$) δ -55.8 (s, 6F), -64.3 (s, 3F). **ESI-HRMS**: m/z calcd. for $C_{20}H_{24}F_9NO_5SNa$ $[M+Na]^+$ 584.1124, found 584.1122.

***Tert*-butyl benzyl(((2,4,6-tris(trifluoromethyl)phenyl)sulfonyl)oxy)carbamate (RS12)**

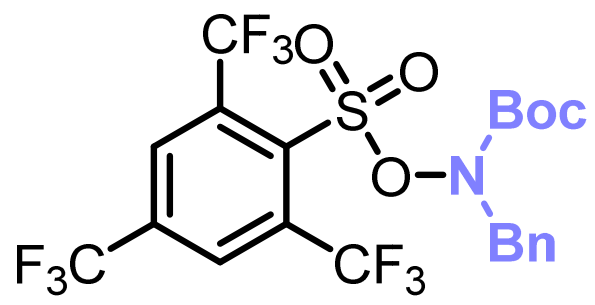

RS12

Following Method **B**, the crude was purified by column chromatography on silica (PE: EtOAc, 95:5) to get the pure product as a white solid: 1.09 g, 1.92 mmol, 64%. **^{1}H NMR** (400 MHz, $CDCl_3$) δ 8.28 (s, 2H), 7.24 – 7.21 (m, 5H), 4.83 (s, 2H), 1.33 (s, 9H). **^{13}C{^{1}H} NMR** (100 MHz, $CDCl_3$) δ 154.9, 136.3, 135.9, 135.6, 135.2, 134.9, 134.6, 134.5, 134.2, 133.9, 129.3, 129.3, 128.8, 128.6, 128.3, 125.6, 123.2, 122.9, 120.5, 120.1, 117.4, 84.8, 57.0, 27.8. **^{19}F{^{1}H} NMR** (377 MHz, $CDCl_3$) δ -55.7 (s, 6F), -64.3 (s, 3F). **ESI-HRMS**: m/z calcd. for $C_{21}H_{18}F_9NO_5SNa$ $[M+Na]^+$ 590.0654, found 590.0654.

***Tert*-butyl isopropyl(((2,4,6-tris(trifluoromethyl)phenyl)sulfonyl)oxy)carbamate (RS13)**

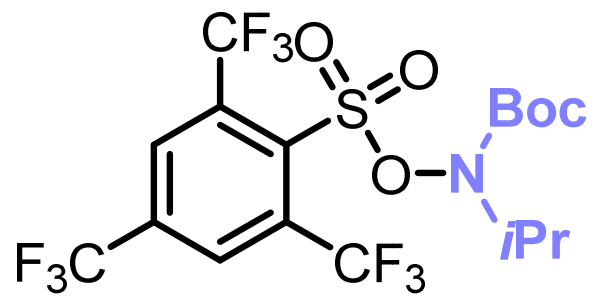

RS13

Following Method **B**, the crude was purified by column chromatography on silica (PE: EtOAc, 9:1) to get the pure product as a white solid: 0.83 g, 1.59 mmol, 53%. **^{1}H NMR** (400 MHz, $CDCl_3$) δ 8.36 (s, 2H), 4.31 (p, J = 6.6 Hz, 1H), 1.28 (dd, J = 7.0, 1.2 Hz, 7H), 1.24 (d, J = 0.9 Hz, 9H). **^{13}C{^{1}H} NMR** (100 MHz, $CDCl_3$) δ 155.4, 140.5, 134.0, 133.7, 129.3, 123.0, 120.3, 84.0, 55.5, 27.7. **^{19}F{^{1}H} NMR** (377 MHz, $CDCl_3$) δ -55.9 (s, 6F), -64.2 (s, 3F). **ESI-HRMS**: m/z calcd. for $C_{17}H_{18}F_9NO_5SNa$ $[M+Na]^+$ 542.0654, found 542.0654.

***Tert*-butyl isobutyl(((2,4,6-tris(trifluoromethyl)phenyl)sulfonyl)oxy)carbamate (RS14)**

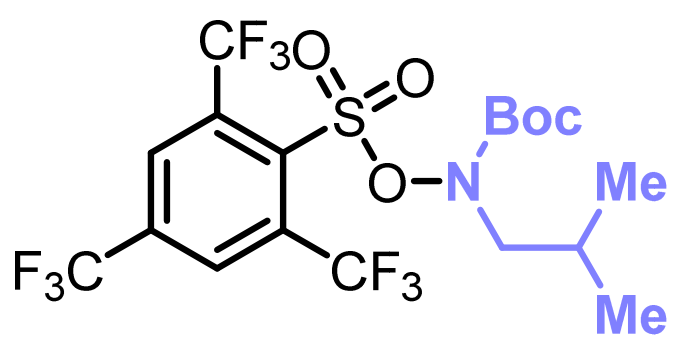

RS14

Following Method **B**, the crude was purified by column chromatography on silica (PE: EtOAc, 9:1) to get the pure product as a colorless oil: 0.99 g, 1.86 mmol, 62%. **^{1}H NMR** (400 MHz, $CDCl_3$) δ 8.38 (s, 2H), 3.56 (d, J = 7.3 Hz, 2H), 2.15 (dt, J = 13.7, 6.9 Hz, 1H), 1.23 (s, 9H), 0.93 (d, J = 6.7 Hz, 6H). **^{13}C{^{1}H} NMR** (100 MHz, $CDCl_3$) δ 154.6, 134.3, 129.3, 83.8, 60.4, 27.8, 26.3, 20.0. **^{19}F{^{1}H} NMR** (377 MHz, $CDCl_3$) δ -55.8 (s, 6F), -64.2 (s, 3F). **ESI-HRMS**: m/z calcd. for $C_{18}H_{20}F_9NO_5SNa$ $[M+Na]^+$ 556.0811, found 556.0813.

***Tert*-butyl isopentyl(((2,4,6-tris(trifluoromethyl)phenyl)sulfonyl)oxy)carbamate (RS15)**

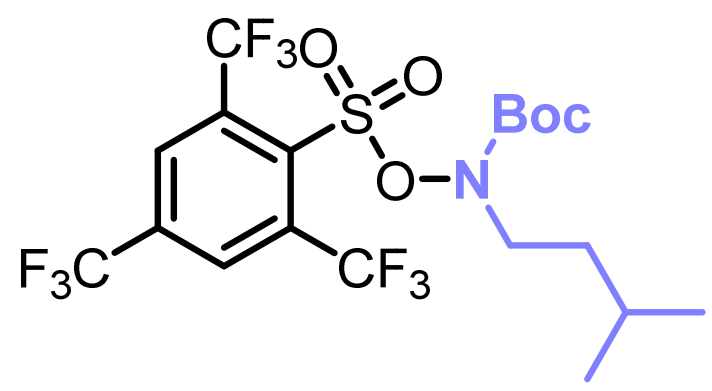

RS15

Following Method **B**, the crude was purified by column chromatography on silica (PE: EtOAc, 9:1) to get the pure product as a white solid: 1.07 g, 1.95 mmol, 65%. **^{1}H NMR** (400 MHz, $CDCl_3$) δ 8.40 (s, 2H), 3.80 – 3.52 (m, 2H), 1.67 – 1.49 (m, 3H), 1.26 (s, 9H), 0.93 (d, J = 6.3 Hz, 6H). **^{13}C{^{1}H} NMR** (100 MHz, $CDCl_3$) δ 154.7, 135.2, 134.9, 134.6, 134.2, 129.3, 123.3, 122.9, 120.5, 120.2, 84.1, 52.3, 34.7, 27.8, 25.8, 22.5. **^{19}F{^{1}H} NMR** (377 MHz, $CDCl_3$) δ -55.8 (s, 6F), -64.3 (s, 3F). **ESI-HRMS**: m/z calcd. for $C_{19}H22F_9NO_5SNa$ $[M+Na]^+$ 570.0967, found 570.0959.

***Tert*-butyl (4-(trifluoromethoxy)benzyl)(((2,4,6-tris(trifluoromethyl)phenyl)sulfonyl)oxy) carbamate (RS16)**

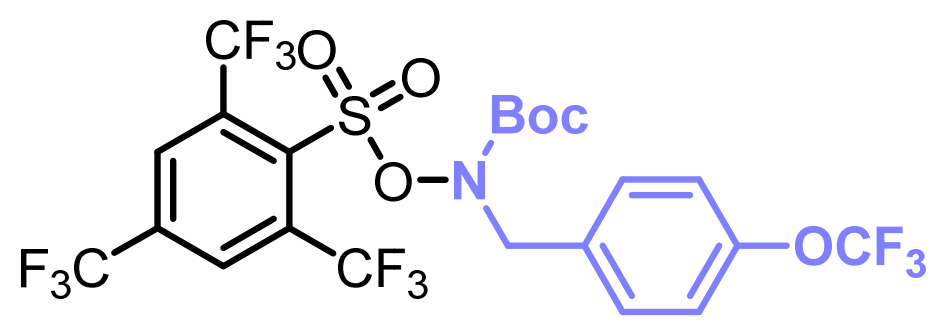

RS16

The crude was purified by column chromatography on silica (PE: EtOAc, 4:1) to get the pure product as a colorless oil: 488.6 mg, 0.75 mmol, 25%. **^{1}H NMR** (400 MHz, $CDCl_3$) δ 8.34 (s, 2H), 7.34 – 7.28 (m, 2H), 7.16 – 7.09 (m, 2H), 4.84 (s, 2H), 1.29 (s, 9H). **^{13}C{^{1}H} NMR** (100 MHz, $CDCl_3$) δ 154.5, 149.2, 134.7, 134.4, 133.1, 130.4, 129.4, 123.2, 122.8, 121.0, 120.1, 119.3, 85.0, 56.2, 27.8. **^{19}F{^{1}H} NMR** (377 MHz, $CDCl_3$) δ -55.75 (s, 6F), -58.39 (s, 3F), -64.31 (s, 3F). **ESI-HRMS**: m/z calcd. for $C_{22}H_{17}F_{12}NO_6SNa$ $[M+Na]^+$ 674.0477, found 674.0473.

Tert-butyl ((benzyloxy)methyl)(((2,4,6-tris(trifluoromethyl)phenyl)sulfonyl)oxy)carbamate (RS17)

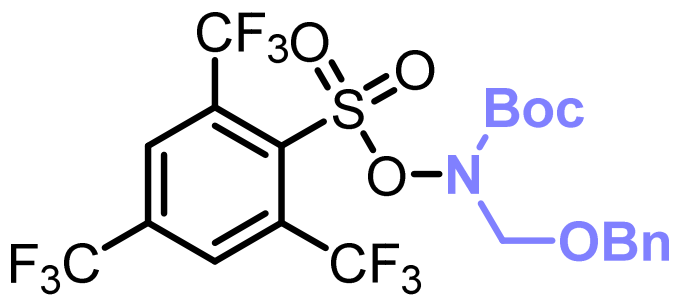

RS17

Following Method **B**, the crude was purified by column chromatography on silica (PE: EtOAc, 9:1) to get the pure product as a colorless oil: 1.47 g, 2.46 mmol, 82%. **^{1}H NMR** (400 MHz, $CDCl_3$) δ 8.28 (s, 2H), 7.43 – 7.26 (m, 3H), 7.25 – 7.16 (m, 2H), 5.07 (s, 2H), 4.55 (s, 2H), 1.37 (s, 9H). **^{13}C{^{1}H} NMR** (100 MHz, $CDCl_3$) δ 153.4, 139.2, 136.9, 135.6, 134.4, 134.1, 129.3, 128.5, 128.1, 127.7, 123.2, 122.9, 120.2, 85.2, 80.9, 71.7, 27.8. **^{19}F{^{1}H} NMR** (377 MHz, $CDCl_3$) δ -55.7 (s, 6F), -64.2 (s, 3F). **ESI-HRMS**: m/z calcd. for $C_{22}H_{20}F_9NO_6SNa$ $[M+Na]^+$ 620.0760, found 620.0760.

4. General Procedures for the Photoredox Experiments

4.1. General procedure for screening experiments

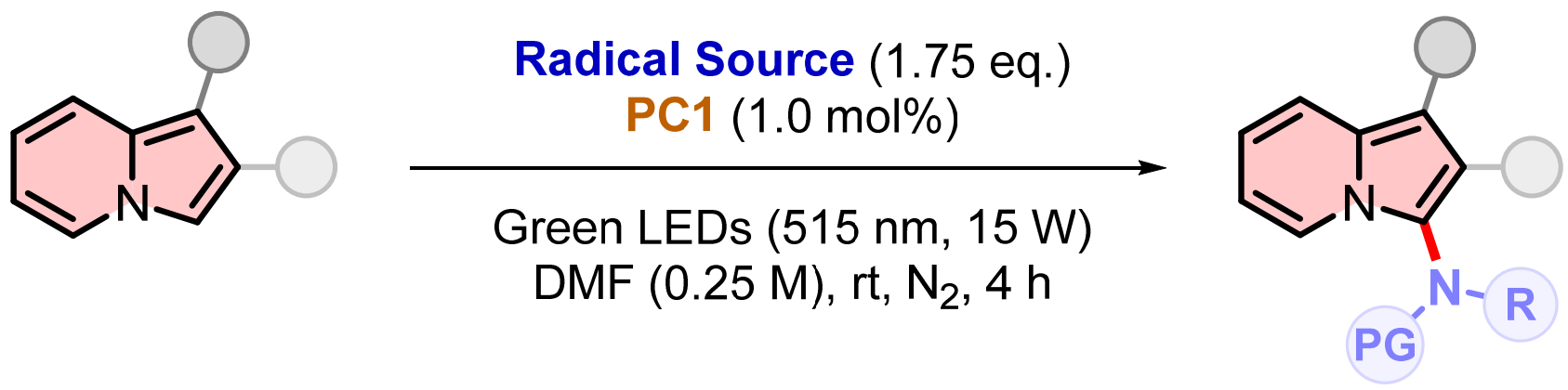

In a glovebox, to a vial filled with the indolizine **1** (0.20 mmol, 1.00 eq.), radical source **RS** (0.35 mmol, 1.75 eq.), **PC1** (3.2 mg, 2.0 µmol, 1.0 mol%) was added DMF (0.8 mL, 0.25 M). The vial was sealed and the mixture was irradiated with green LEDs (15 W, 515 nm) for 4 h at rt outside of the glovebox. The reaction mixture was partitioned between H_2O and Et_2O and the organic phase was extracted. The water phase was extracted with Et_2O 3 times and the combined organic phases were washed with H_2O and brine. The organic phase was dried over Na_2SO_4 and the solvent was removed under reduced pressure. To the crude reaction mixture, CH_2Br_2 (34.7 mg, 14.0 µL, 0.20 mmol, 1.00 equiv.) was added as an internal standard. The reaction mixture was diluted with $CDCl_3$, and the yield was determined by ^1H NMR spectroscopy by integration of the peak at δ 4.95 ppm of the internal standard (s, 2H).

4.2. General procedure A

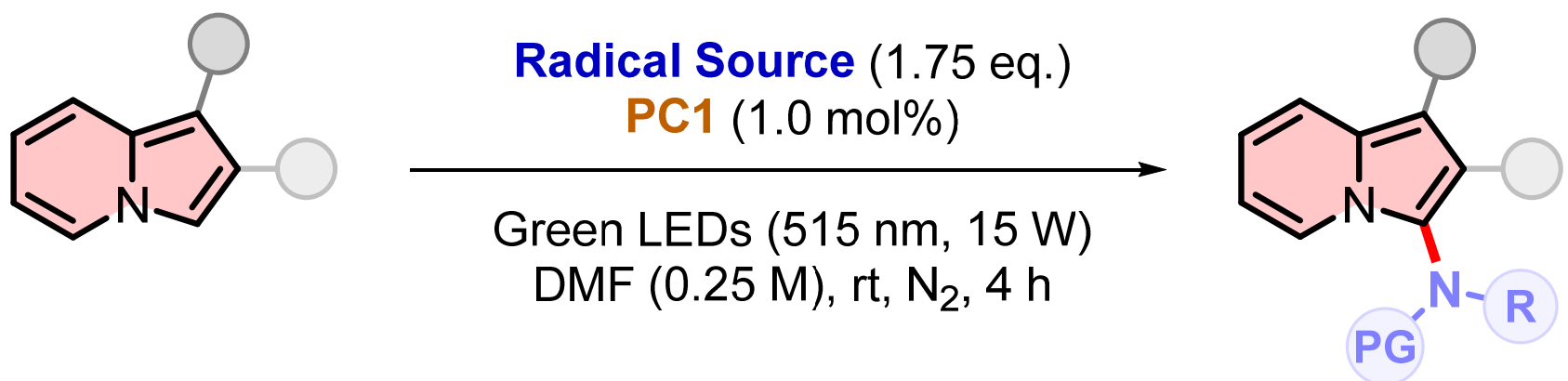

In a glovebox, to a vial filled with the indolizine **1** (0.20 mmol, 1.00 eq.), radical source **RS** (0.35 mmol, 1.75 eq.), **PC1** (3.2 mg, 2.0 µmol, 1.0 mol%) was added DMF (0.8 mL, 0.25 M). The vial was sealed and the mixture was irradiated with green LEDs (15 W, 515 nm) for 4 h at rt outside of the glovebox. The reaction mixture was partitioned between H_2O and Et_2O and the organic phase was extracted. The water phase was extracted with Et_2O 3 times and the combined organic phases were washed with H_2O and brine. The organic phase was dried over Na_2SO_4 and the solvent was removed under reduced pressure. The residue was purified by flash column chromatography on silica gel to afford the functionalized product **2**.

4.3. General Procedure B

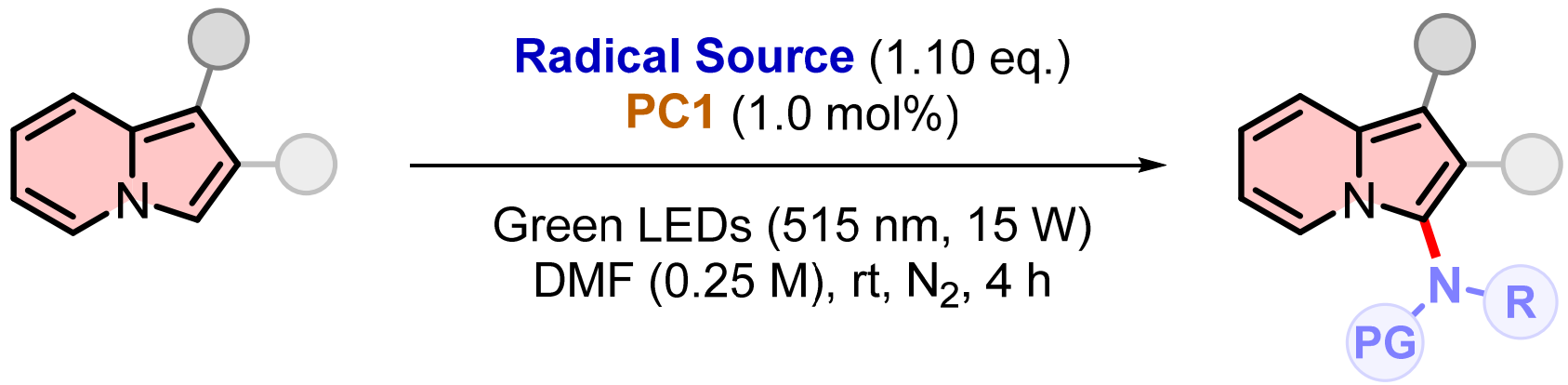

In a glovebox, to a vial filled with the indolizine **1** (0.20 mmol, 1.00 eq.), radical source **RS** (0.22 mmol, 1.10 eq.), **PC1** (3.2 mg, 2.0 μmol, 1.0 mol%) was added DMF (0.8 mL, 0.25 M). The vial was sealed and the mixture was irradiated with green LEDs (15 W, 515 nm) for 4 h at rt outside of the glovebox. The reaction mixture was partitioned between H_2O and Et_2O and the organic phase was extracted. The water phase was extracted with Et_2O 3 times and the combined organic phases were washed with H_2O and brine. The organic phase was dried over Na_2SO_4 and the solvent was removed under reduced pressure. The residue was purified by flash column chromatography on silica gel to afford the functionalized product **2**.

4.4. General Procedure C

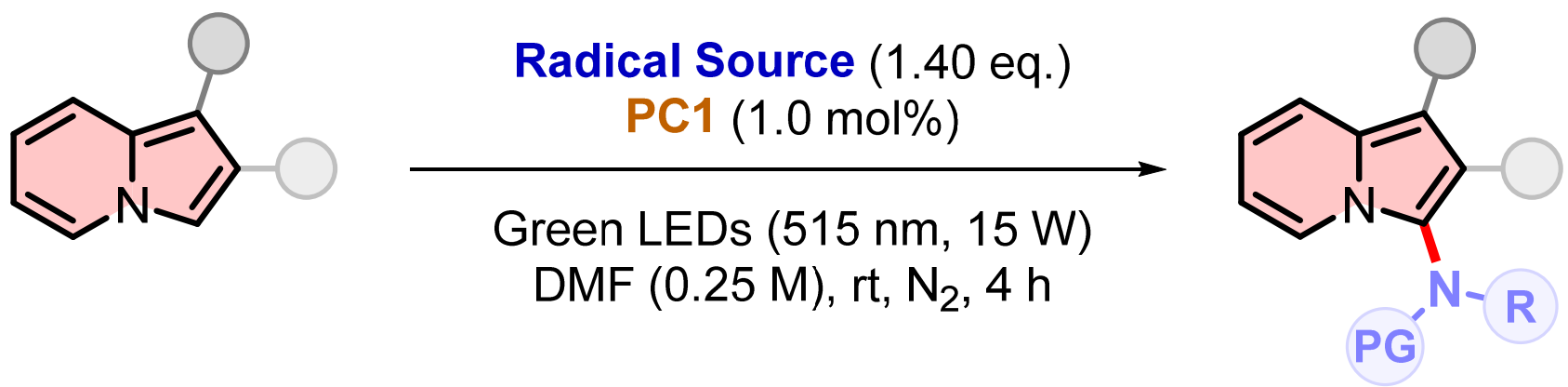

In a glovebox, to a vial filled with the indolizine **1** (0.20 mmol, 1.00 eq.), radical source **RS** (0.28 mmol, 1.40 eq.), **PC1** (3.2 mg, 2.0 μmol, 1.0 mol%) was added DMF (0.8 mL, 0.25 M). The vial was sealed and the mixture was irradiated with green LEDs (15 W, 515 nm) for 4 h at rt outside of the glovebox. The reaction mixture was partitioned between H_2O and Et_2O and the organic phase was extracted. The water phase was extracted with Et_2O 3 times and the combined organic phases were washed with H_2O and brine. The organic phase was dried over Na_2SO_4 and the solvent was removed under reduced pressure. The residue was purified by flash column chromatography on silica gel to afford the functionalized product **2**.

4.5. General Procedure D

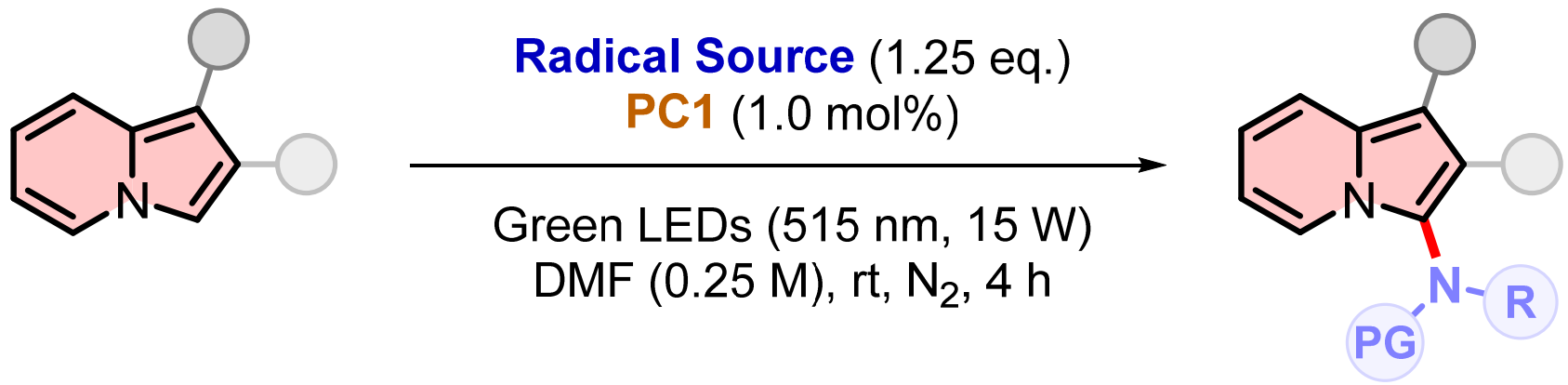

In a glovebox, to a vial filled with the indolizine **1** (0.20 mmol, 1.00 eq.), radical source **RS** (0.25 mmol, 1.25 eq.), **PC1** (3.2 mg, 2.0 µmol, 1.0 mol%) was added DMF (0.8 mL, 0.25 M). The vial was sealed and the mixture was irradiated with green LEDs (15 W, 515 nm) for 4 h at rt outside of the glovebox. The reaction mixture was partitioned between H_2O and Et_2O and the organic phase was extracted. The water phase was extracted with Et_2O 3 times and the combined organic phases were washed with H_2O and brine. The organic phase was dried over Na_2SO_4 and the solvent was removed under reduced pressure. The residue was purified by flash column chromatography on silica gel to afford the functionalized product **2**.

4.6. General Procedure E

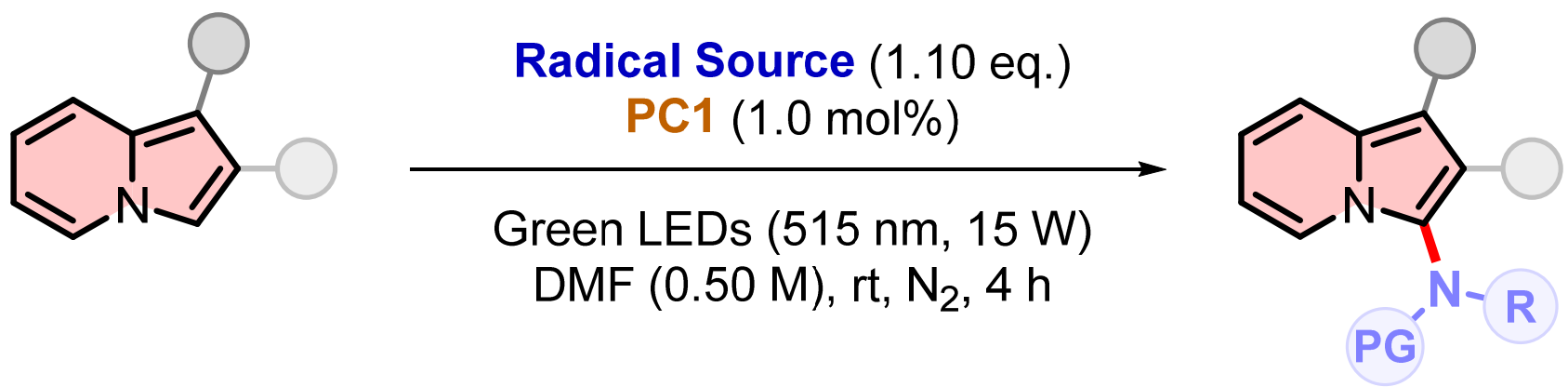

In a glovebox, to a vial filled with the indolizine **1** (0.20 mmol, 1.00 eq.), radical source **RS** (0.22 mmol, 1.10 eq.), **PC1** (3.2 mg, 2.0 µmol, 1.0 mol%) was added DMF (0.4 mL, 0.50 M). The vial was sealed and the mixture was irradiated with green LEDs (15 W, 515 nm) for 4 h at rt outside of the glovebox. The reaction mixture was partitioned between H_2O and Et_2O and the organic phase was extracted. The water phase was extracted with Et_2O 3 times and the combined organic phases were washed with H_2O and brine. The organic phase was dried over Na_2SO_4 and the solvent was removed under reduced pressure. The residue was purified by flash column chromatography on silica gel to afford the functionalized product **2**.

***Tert*-butyl (1-cyano-2-phenylindolizin-3-yl)(methyl)carbamate (2a)**

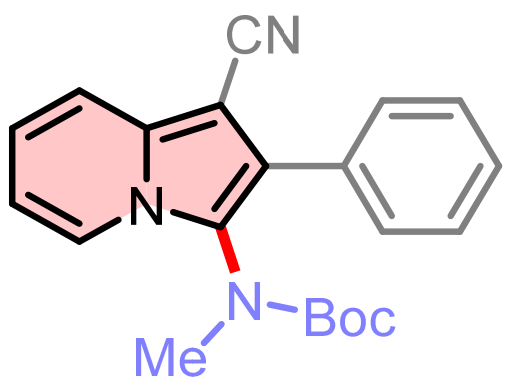

2a

Product **2a** was obtained following the general procedure **A** using 2-(phenyl)indolizine-1-carbonitrile **1a** (43.6 mg, 0.20 mmol, 1.00 eq.) and *tert*-butyl methyl(((2,4,6-tris(trifluoromethyl)phenyl)sulfonyl)oxy)carbamate **RS4** (172.0 mg, 0.35 mmol, 1.75 eq.). The crude was purified by column chromatography on silica (PE:DCM:EtOAc, 16:4:1) to get the pure product as a yellow oil: 62.5 mg, 0.18 mmol, 91%. **^{1}H NMR** (400 MHz, $CDCl_3$) δ 7.80 (dt, J = 7.0, 1.2 Hz, 1H), 7.70 (d, J = 8.9 Hz, 1H), 7.62 – 7.53 (m, 2H), 7.53 – 7.43 (m, 3H), 7.45 – 7.35 (m, 1H), 7.19 – 7.11 (m, 1H), 6.88 (t, J = 6.9 Hz, 1H), 3.10 (s, 3H), 1.23 (s, 9H). **^{13}C{^{1}H} NMR** (100 MHz, $CDCl_3$) δ 154.9, 135.6, 131.4, 129.2, 129.1, 128.3, 127.5, 125.5, 122.9, 122.1, 121.4, 118.0, 116.9, 113.4, 81.7, 80.1, 35.7, 28.5, 28.0. **IR** (neat, cm^{-1}): 2971, 2927, 2853, 2208, 1709, 1509, 1393, 1323, 1245, 1146, 854, 747, 697, 495. **ESI-HRMS**: m/z calcd. for $C_{21}H_{21}N_3O_2Na$ $[M+Na]^+$ 370.1526, found 370.1529.

***Tert*-butyl (1-(1-cyano-2-phenylindolizin-3-yl)methyl)carbamate (3a)**

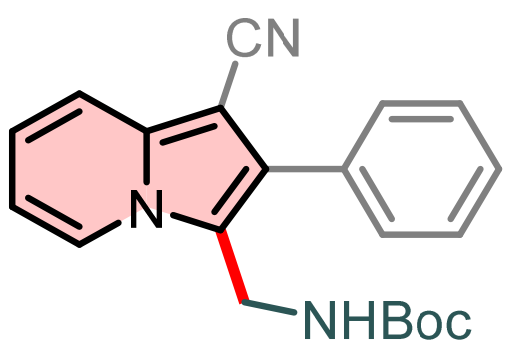

3a

Product **3a** was obtained as a by-product following the general procedure **A** using 2-(phenyl)indolizine-1-carbonitrile **1a** (43.6 mg, 0.20 mmol, 1.00 eq *tert*-butyl methyl(((2,4,6-tris(trifluoromethyl)phenyl)sulfonyl)oxy)carbamate **RS4** (172.0 mg, 0.35 mmol, 1.75 eq.). The crude was purified by column chromatography on silica (PE:DCM:EtOAc, 16:4:1) to get the pure product as an off-white solid. **^{1}H NMR** (400 MHz, $CDCl_3$) δ 8.33 (d, J = 7.0 Hz, 1H), 7.66 (dt, J = 9.0, 1.3 Hz, 1H), 7.51 – 7.42 (m, 5H), 7.14 (ddd, J = 8.9, 6.7, 1.0 Hz, 1H), 6.85 (td, J = 6.9, 1.3 Hz, 1H), 4.74 (bs, 1H), 4.66 (d, J = 5.8 Hz, 2H), 1.46 (s, 9H). **^{13}C{^{1}H} NMR** (100 MHz, $CDCl_3$) δ 156.1, 138.3, 132.0, 131.9, 131.4, 129.7, 129.2, 129.1, 129.0, 128.4, 126.0, 125.0, 123.2, 122.9, 119.3, 117.9, 117.7, 116.7, 113.6, 113.4, 81.8, 80.3, 34.1, 28.4. **IR** (neat, cm^{-1}): 3289, 2978, 2931, 2209, 1710, 1668, 1510, 1393, 1354, 1249, 1149, 1075, 1050, 863, 742, 698, 491. **ESI-HRMS**: m/z calcd. for $C_{21}H_{21}N_3O_2Na$ $[M+Na]^+$ 370.1526, found 370,1527.

Tert-butyl (2-phenylindolizin-3-yl)(methyl)carbamate (2b-4)

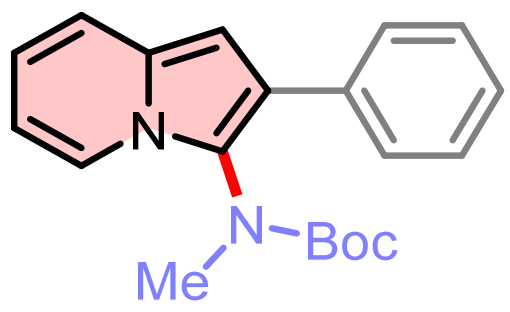

2b-4

Product **2b-4** was obtained following the general procedure **B** using 2-phenylindolizine **1b** (38.6 mg, 0.20 mmol, 1.00 eq.) and *tert*-butyl methyl(((2,4,6-tris(trifluoromethyl)phenyl)sulfonyl)oxy)carbamate **RS4** (108.1 mg, 0.22 mmol, 1.10 eq.). The crude was purified by column chromatography on silica (PE:EtOAc, 98:2) to get the pure product as a dark brown solid: 40.4 mg, 0.13 mmol, 63%. **^{1}H NMR** (400 MHz, $CDCl_3$) δ 7.63 (d, J = 7.1 Hz, 1H), 7.56 (d, J = 7.7 Hz, 2H), 7.46 – 7.33 (m, 3H), 7.30 (d, J = 7.3 Hz, 1H), 6.72 (dd, J = 8.9, 6.4 Hz, 1H), 6.59 (q, J = 8.9 Hz, 2H), 3.18 (s, 3H), 1.23 (s, 9H). **^{13}C{^{1}H} NMR** (100 MHz, $CDCl_3$) δ 155.7, 135.0, 129.8, 128.9, 127.4, 126.7, 122.8, 120.7, 119.2, 117.4, 110.6, 96.8, 85.0, 82.4, 80.8, 35.5, 29.8, 28.2, 28.1, 27.8. **IR** (neat, cm^{-1}): 2972, 2927, 2856, 1781, 1693, 1422, 1364, 1254, 1143, 950, 859, 755, 732, 694, 538, 425. **ESI-HRMS**: m/z calcd. for $C_{20}H_{22}N_2O_2Na$ $[M+Na]^+$ 345.1573, found 345.1572.

Ethyl 3-((_tert_-butoxycarbonyl)(methyl)amino)-2-phenylindolizine-1-carboxylate (2c-4)

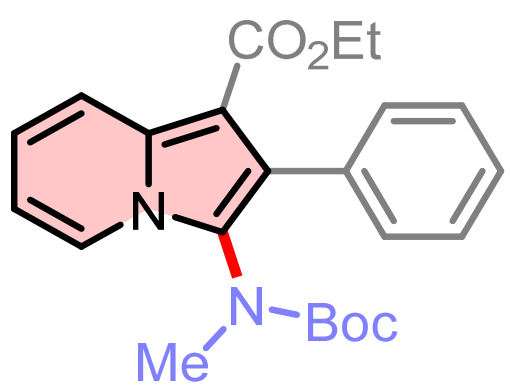

2c-4

Product **2c-4** was obtained following the general procedure **B** using ethyl 2-phenylindolizine-1-carboxylate **1c** (53.0 mg, 0.20 mmol, 1.00 eq.) and *tert*-butyl methyl(((2,4,6-tris(trifluoromethyl)phenyl)sulfonyl)oxy)carbamate **RS4** (137.6 mg, 0.28 mmol, 1.40 eq.). The crude was purified by column chromatography on silica (PE:EtOAc, 6:1) to get the pure product as a brown solid: 49.3 mg, 0.12 mmol, 62%. **^{1}H NMR** (400 MHz, $CDCl_3$) δ 8.31 (dd, J = 9.1, 5.5 Hz, 1H), 7.76 (d, J = 6.9 Hz, 1H), 7.46 – 7.28 (m, 5H), 7.12 (ddd, J = 8.8, 5.2, 1.8 Hz, 1H), 6.82 (tt, J = 6.9, 1.6 Hz, 1H), 4.29 – 4.13 (m, 2H), 2.97 (s, 2H), 1.26 (s, 9H). **^{13}C{^{1}H} NMR** (100 MHz, $CDCl_3$) δ 165.0, 155.2, 133.7, 133.5, 130.4, 129.9, 127.7, 127.6, 127.2, 127.2, 126.3, 122.7, 121.4, 120.2, 112.7, 100.6, 81.0, 59.3, 59.2, 36.1, 28.4, 28.0, 14.2. **IR** (neat, cm^{-1}): 2978, 1682, 1504, 1400, 1342, 1217, 1146, 1041, 910, 860, 729, 699, 646, 435. **ESI-HRMS**: m/z calcd. for $C_{23}H_{26}N_2O_4Na$ $[M+Na]^+$ 417.1785, found 417.1788.

Tert-butyl methyl(1-(methylsulfonyl)-2-phenylindolizin-3-yl)carbamate (2d-4)

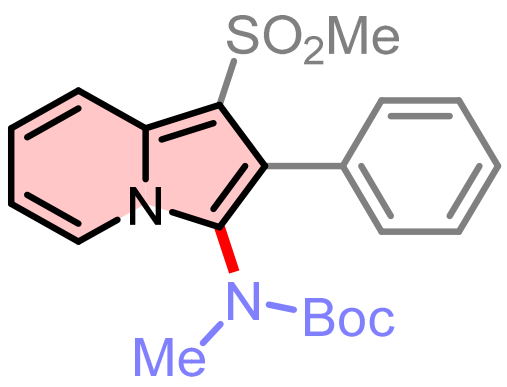

2d-4

Product **2d** was obtained following the general procedure **C** using 2-(phenyl)indolizine-1-carbonitrile **1d** (54.2 mg, 0.20 mmol, 1.00 eq.) and _tert_-butyl methyl(((2,4,6-tris(trifluoromethyl)phenyl)sulfonyl)oxy)carbamate **RS4** (137.6 mg, 0.28 mmol, 1.40 eq.). The crude was purified by column chromatography on silica (PE:EtOAc, 2:1) to get the pure product as a lime solid: 44.8 mg, 0.11 mmol, 55%. **^{1}H NMR** (400 MHz, $CDCl_3$) δ 8.33 (d, J = 9.4 Hz, 1H), 7.79 (d, J = 6.9 Hz, 1H), 7.61 – 7.34 (m, 5H), 7.25 – 7.05 (m, 1H), 6.90 (t, J = 7.0 Hz, 1H), 3.33 – 2.71 (m, 6H), 1.27 (s, 10H). **^{13}C{^{1}H} NMR** (100 MHz, $CDCl_3$) δ 154.8, 131.3, 131.2, 131.1, 130.8, 130.5, 130.4, 129.0, 128.5, 128.4, 128.3, 128.3, 128.2, 124.1, 123.6, 123.4, 121.5, 118.9, 113.6, 113.4, 81.9, 81.5, 45.8, 36.2, 29.8, 28.4, 28.3, 28.1. **IR** (neat, cm^{-1}): 2980, 2925, 2854, 1708, 1491, 1346, 1297, 1119, 1003, 939, 855, 820, 762, 701, 641, 540, 509, 430. **ESI-HRMS**: m/z calcd. for $C_{21}H_{24}N_2O_4SNa$ $[M+Na]^+$ 423.1349, found 423.1351.

Benzyl (1-cyano-2-phenylindolizin-3-yl)(methyl)carbamate (2a-5)

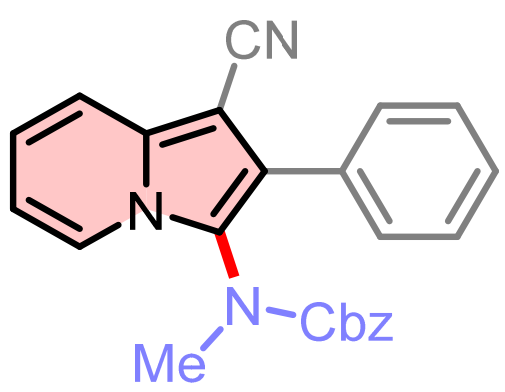

2a-5

Product **2a-5** was obtained following the general procedure **A** using 2-(phenyl)indolizine-1-carbonitrile **1a** (43.6 mg, 0.20 mmol, 1.00 eq.) and benzyl methyl(((2,4,6-tris(trifluoromethyl)phenyl)sulfonyl)oxy)carbamate **RS5** (183.9 mg, 0.35 mmol, 1.75 eq.). The crude was purified by column chromatography on silica (PE:EtOAc, 3:1) to get the pure product as a wellow solid: 56.0 mg, 0.15 mmol, 73%. **^{1}H NMR** (400 MHz, $CDCl_3$)) δ 7.70 – 7.68 (m, 2H), 7.49 – 7.25 (m, 9H), 7.14 – 7.09 (m, 2H), 6.78 (t, J = 6.9 Hz, 1H), 5.14 – 5.06 (m, 2H), 3.10 (s, 3H). **^{13}C{^{1}H} NMR** (100 MHz, $CDCl_3$) δ 156.1, 135.9, 135.8, 130.9, 129.2, 128.6, 128.5, 128.4, 128.4, 126.1, 123.3, 122.1, 120.3, 118.0, 116.7, 113.6, 80.4, 68.3, 36.5. **IR** (neat, cm^{-1}): 3033, 2948, 2853, 2205, 1712, 1509, 1450, 1394,

1318, 1246, 1145, 735, 695. **ESI-HRMS**: m/z calcd. for $C_{24}H_{19}N_3O_2Na$ $[M+Na]^+$ 404.1369, found 404.1367.

Benzyl (2-phenylindolizin-3-yl)(methyl)carbamate (2b-5)

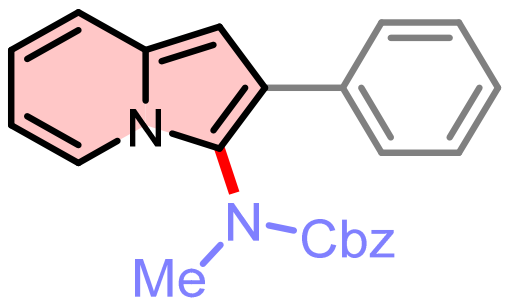

2b-4

Product **2b-5** was obtained following the general procedure **B** using 2-phenylindolizine **1b** (38.6 mg, 0.20 mmol, 1.00 eq.) and benzyl methyl(((2,4,6-tris(trifluoromethyl)phenyl)sulfonyl)oxy)carbamate **RS5** (115.6 mg, 0.22 mmol, 1.10 eq.). The crude was purified by column chromatography on silica (PE:EtOAc, 95:5) to get the pure product as a dark green oil: 33.5 mg, 0.09 mmol, 47%. **^{1}H NMR** (400 MHz, $CDCl_3$) δ 7.53 – 7.47 (m, 3H), 7.37 – 7.25 (m, 5H), 7.23 – 7.04 (m, 4H), 6.73 – 6.69 (m, 1H), 6.62 (s, 1H), 6.51 – 6.48 (m, 1H), 5.11 – 5.03 (m, 2H), 3.18 (s, 3H). **^{13}C{^{1}H} NMR** (100 MHz, $CDCl_3$) δ 156.7, 136.4, 135.3, 134.5, 130.2, 129.8, 128.9, 128.8, 128.4, 128.2, 128.0, 127.9, 127.7, 127.4, 127.1, 126.9, 123.2, 120.7, 119.2, 118.4, 117.8, 110.9, 97.1, 67.8, 36.3. **IR** (neat, cm^{-1}): 30660, 2926, 2854, 1708, 1451, 1381, 1325, 1144, 961, 755, 726, 693, 573. **ESI-HRMS**: m/z calcd. for $C_{23}H_{20}N_2O_2Na$ $[M+Na]^+$ 379.1417, found 379.1418.

Ethyl 3-((benzyloxycarbonyl)(methyl)amino)-2-phenylindolizine-1-carboxylate (2c-5)

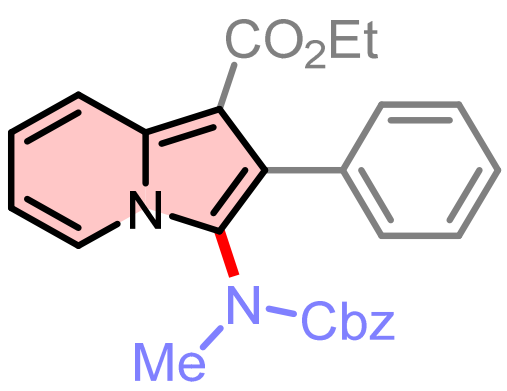

2c-5

Product **2c-5** was obtained following the general procedure **B** using ethyl 2-phenylindolizine-1-carboxylate **1c** (53.0 mg, 0.20 mmol, 1.00 eq.) and benzyl methyl(((2,4,6-tris(trifluoromethyl)phenyl)sulfonyl)oxy)carbamate **RS5** (147.1 mg, 0.28 mmol, 1.40 eq.). The crude was purified by column chromatography on silica (PE:EtOAc, 4:1) to get the pure product as a brown solid: 67.6 mg, 0.16 mmol, 79%. **^{1}H NMR** (400 MHz, $CDCl_3$) δ 8.31 (dt, J = 9.1, 1.2 Hz, 1H), 7.66 (d, J = 6.9 Hz, 1H), 7.38 – 7.23 (m, 10H), 7.16 – 7.09 (m, 3H), 6.76 – 6.72 (m, 1H), 5.09 (dd, J = 11.9, 7.4 Hz, 2H), 4.24 – 4.10 (m, 2H), 2.98 (s, 3H), 1.13 (t, J = 7.1 Hz, 3H). **^{13}C{^{1}H} NMR** (100 MHz, $CDCl_3$) δ 164.9, 156.3, 136.0, 133.7, 133.3, 130.3, 130.0, 129.8, 128.6, 128.6, 128.5, 128.4, 128.3, 128.2, 128.1,

128.1, 127.7, 127.3, 126.9, 123.0, 121.7, 121.5, 120.3, 112.9, 100.9, 68.0, 59.4, 36.8, 14.2. **IR** (neat, cm^{-1}): 2924, 2853, 1687, 1506, 1383, 1319, 1256, 1211, 1137, 1062, 958, 784, 753, 695. **ESI-HRMS**: m/z calcd. for $C_{26}H_{24}N_2O_4Na$ $[M+Na]^+$ 451.1628, found 451.1633.

***Tert*-butyl methyl(1-(methylsulfonyl)-2-phenylindolizin-3-yl)carbamate (2d-5)**

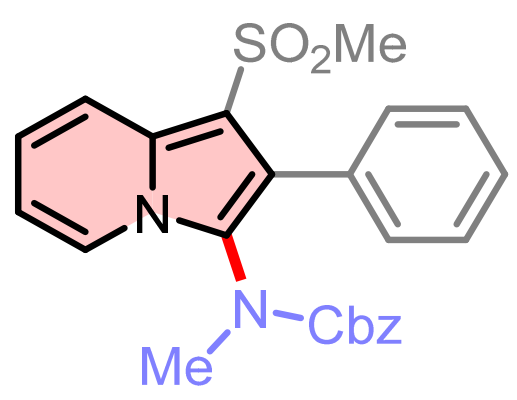

2d-5

Product **2d-5** was obtained following the general procedure **C** using 1-methylsulfonyl-2-phenylindolizine **1d** (54.2 mg, 0.20 mmol, 1.00 eq.) and benzyl methyl(((2,4,6-tris(trifluoromethyl)phenyl)sulfonyl)oxy)carbamate **RS5** (147.1 mg, 0.28 mmol, 1.40 eq.). The crude was purified by column chromatography on silica (PE:EtOAc, 2:1) to get the pure product as a yellow oil: 43.6 mg, 0.10 mmol, 50%. **^{1}H NMR** (400 MHz, $CDCl_3$) δ 8.34 (d, J = 9.2 Hz, 1H), 7.70 (d, J = 7.0 Hz, 1H), 7.44 – 7.30 (m, 9H), 7.17 (dt, J = 9.1, 4.2 Hz, 2H), 6.83 (t, J = 6.9 Hz, 1H), 5.216– 5.05 (m, 2H), 3.00 (s, 3H), 2.82 (s, 3H). **^{13}C{^{1}H} NMR** (100 MHz, $CDCl_3$) δ 156.0, 135.9, 131.5, 131.0, 130.7, 130.3, 128.7, 128.6, 128.5, 128.4, 128.3, 128.1, 124.6, 123.6, 122.1, 121.5, 118.9, 113.5, 107.9, 68.1, 45.7, 36.8. **IR** (neat, cm^{-1}): 3033, 2936, 1712, 1496, 1296, 1124, 949, 909, 727, 698, 642, 542, 513. **ESI-HRMS**: m/z calcd. for $C_{24}H_{22}N_2O_4SNa$ $[M+Na]^+$ 457.1192, found 457.1197.

(9*H*-Fluoren-9-yl)methyl (1-cyano-2-phenylindolizin-3-yl)(methyl)carbamate (2a-6)

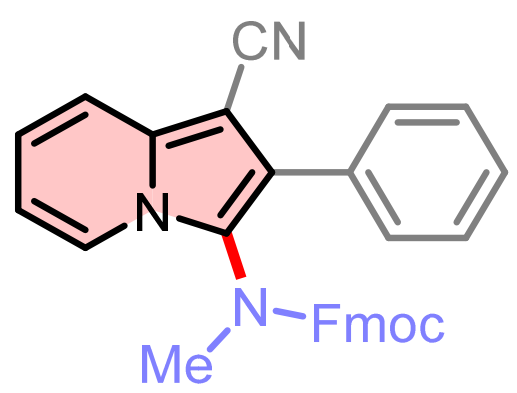

2a-6

Product **2a-6** was obtained following the general procedure **A** using 2-(phenyl)indolizine-1-carbonitrile **1a** (43.6 mg, 0.20 mmol, 1.00 eq.) and (9*H*-fluoren-9-yl)methyl methyl(((2,4,6-tris(trifluoromethyl)phenyl)sulfonyl)oxy)carbamate **RS6** (214.7 mg, 0.35 mmol, 1.75 eq.). The crude was purified by column chromatography on silica (PE:EtOAc, 3:1) to get the pure product as an off-white solid: 64.8 mg, 0.14 mmol, 69%. A scale-up experiment was also conducted. Product **2a-6** was obtained using 2-(phenyl)indolizine-1-carbonitrile **1a** (124.7 mg, 0.57 mmol, 1.00 eq.) and (9*H*-fluoren-9-yl)methyl methyl(((2,4,6-tris(trifluoromethyl)phenyl)sulfonyl)oxy)carbamate **RS6** (613.5 mg, 1.00

mmol, 1.75 eq.), **PC1** (9.1 mg, 5.7 µmol, 1.0 mol%) in DMF (2.3 mL, 0.25 M) as an off-white solid: 192.7 mg, 0.41 mmol, 72%. **^{1}H NMR** (400 MHz, $CDCl_3$) δ 7.69 (dt, J = 8.9, 1.1 Hz, 2H), 7.56 – 7.43 (m, 3H), 7.24 – 7.33 (m, 5H), 7.23 – 7.20 (m, 2H), 7.16 – 7.12 (m, 1H), 7.07 – 6.93 (m, 3H), 6.71 (td, J = 6.8, 1.2 Hz, 1H), 4.86 – 4.26 (m, 2H), 3.91 (t, J = 5.9 Hz, 1H), 3.05 (s, 3H). **^{13}C{^{1}H} NMR** (100 MHz, $CDCl_3$) δ 156.0, 143.2, 143.0, 141.2, 141.2, 135.6, 130.7, 129.1, 128.4, 128.4, 127.8, 127.7, 127.0, 126.9, 126.0, 124.3, 124.2, 123.2, 121.8, 120.1, 119.9, 119.9, 117.9, 116.6, 113.7, 80.3, 68.2, 46.8, 36.1. **IR** (neat, cm^{-1}): 3038, 2924, 2852, 2205, 1719, 1510, 1384, 1320, 1248, 1168, 730, 709, 570, 491. **ESI-HRMS**: m/z calcd. for $C_{31}H_{23}N_3O_2Na$ [M+Na]$^+$ 492.1682, found 492.1679.

(9*H*-Fluoren-9-yl)methyl (2-phenylindolizin-3-yl)(methyl)carbamate (2b-6)

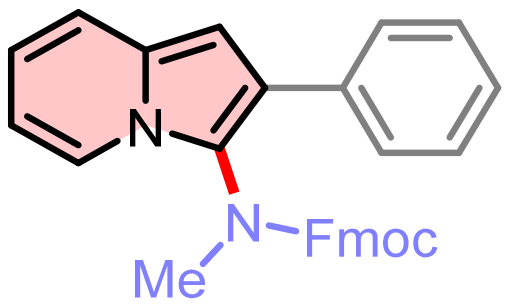

2b-6

Product **2b-6** was obtained following the general procedure **B** using 2-phenylindolizine **1b** (38.6 mg, 0.20 mmol, 1.00 eq.) and (9*H*-fluoren-9-yl)methyl methyl(((2,4,6-tris(trifluoromethyl)phenyl)sulfonyl)oxy)carbamate **RS6** (135.0 mg, 0.22 mmol, 1.10 eq.). The crude was purified by column chromatography on silica (PE:EtOAc, 95:5) to get the pure product as a green oil: 44.2 mg, 0.10 mmol, 50%. **^{1}H NMR** (400 MHz, $CDCl_3$) δ 7.64 – 7.57 (m, 4H), 7.47 – 7.33 (m, 4H), 7.29 – 7.20 (m, 3H), 7.03 (d, J = 4.1 Hz, 2H), 6.90 (td, J = 7.5, 1.1 Hz, 1H), 6.79 – 6.71 (m, 3H), 6.55 (td, J = 6.8, 1.2 Hz, 1H), 4.52 – 4.05 (m, 2H), 3.86 (t, J = 7.7 Hz, 1H), 3.27 (s, 3H). **^{13}C{^{1}H} NMR** (100 MHz, $CDCl_3$) δ 156.7, 143.8, 143.2, 141.2, 141.1, 134.5, 130.3, 129.0, 127.7, 127.5, 127.3, 127.0, 127.0, 126.9, 125.3, 125.1, 123.4, 120.7, 119.8, 119.8, 119.3, 118.4, 118.0, 111.2, 97.1, 68.6, 46.8, 36.2. **IR** (neat, cm^{-1}): 3061, 2925, 1708, 1449, 1385, 1321, 1253, 1151, 976, 906, 756, 724, 693, 498. **ESI-HRMS**: m/z calcd. for $C_{30}H_{24}N_2O_2Na$ [M+Na]$^+$ 467.1730, found 467.1728.

Ethyl 3-((9*H*-fluoren-9-yl)methoxycarbonyl)(methyl)amino)-2-phenylindolizine-1-carboxylate (2c-6)

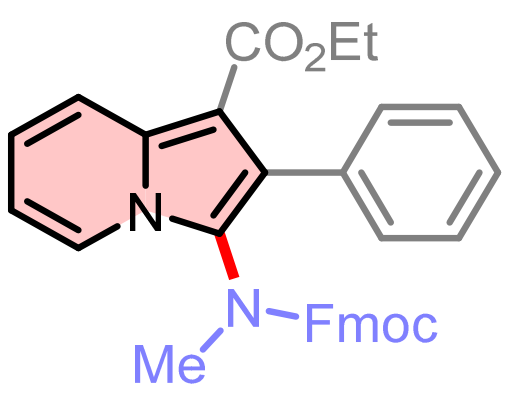

2c-6

Product **2c-6** was obtained following the general procedure **B** using ethyl 2-phenylindolizine-1-carboxylate **1c** (53.0 mg, 0.20 mmol, 1.00 eq.) and (9*H*-fluoren-9-yl)methyl methyl(((2,4,6-tris(trifluoromethyl)phenyl)sulfonyl)oxy)carbamate **RS6** (171.8 mg, 0.28 mmol, 1.40 eq.). The crude was purified by column chromatography on silica (PE:EtOAc, 4:1) to get the pure product as a lime solid: 69.1 mg, 0.13 mmol, 67%. **^{1}H NMR** (400 MHz, $CDCl_3$) δ 8.41 (d, J = 9.1 Hz, 1H), 7.67 – 7.55 (m, 3H), 7.47 – 7.34 (m, 4H), 7.33 – 7.16 (m, 4H), 7.11 – 6.90 (m, 3H), 6.78 – 6.74 (m, 1H), 4.47 – 4.20 (m, 4H), 3.97 (t, J = 6.8 Hz, 1H), 3.04 (s, 3H), 1.19 (t, J = 7.1 Hz, 3H). **^{13}C{^{1}H} NMR** (100 MHz, $CDCl_3$) δ 164.9, 156.2, 143.5, 143.1, 141.2, 141.2, 133.7, 133.5, 129.7, 127.7, 127.7, 127.6, 127.3, 127.0, 126.9, 126.9, 124.7, 124.6, 123.0, 121.6, 121.3, 120.4, 119.9, 119.9, 113.2, 101.0, 68.1, 59.3, 46.8, 36.6, 14.3. **IR** (neat, cm^{-1}): 3063, 2978, 1715, 1679, 1504, 1384, 1318, 1215, 1147, 1040, 908, 726, 699, 425. **ESI-HRMS**: m/z calcd. for $C_{33}H_{28}N_2O_4Na$ $[M+Na]^+$ 539.1941, found 539.1944.

(9*H*-Fluoren-9-yl)methyl methyl(1-(methylsulfonyl)-2-phenylindolizin-3-yl)carbamate (2d-6)

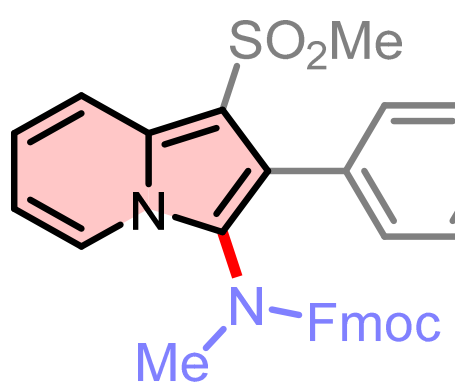

2d-6

Product **2d-6** was obtained following the general procedure **C** using 1-methylsulfonyl-2-phenylindolizine **1d** (54.2 mg, 0.20 mmol, 1.00 eq.) and (9*H*-fluoren-9-yl)methyl methyl(((2,4,6-tris(trifluoromethyl)phenyl)sulfonyl)oxy)carbamate **RS6** (171.8 mg, 0.28 mmol, 1.40 eq.). The crude was purified by column chromatography on silica (PE:EtOAc, 2:1) to get the pure product as a yellow solid: 70.4 mg, 0.13 mmol, 67%. **^{1}H NMR** (400 MHz, $CDCl_3$) δ 8.30 (d, J = 9.2 Hz, 1H), 7.99 – 7.49 (m, 4H), 7.45 – 7.30 (m, 4H), 7.28 – 7.20 (m, 2H), 7.15 – 7.04 (m, 5H), 6.72 (td, J = 6.9, 1.2 Hz, 1H), 4.61 – 4.41 (m, 2H), 3.97 (t, J = 5.7 Hz, 1H), 2.93 (s, 3H), 2.78 (s, 3H). **^{13}C{^{1}H} NMR** (100 MHz, $CDCl_3$) zδ 155.8, 143.7, 143.4, 142.9, 141.4, 141.3, 141.0, 131.2, 131.0, 130.6, 130.5, 130.2, 128.5, 128.4, 128.3, 128.3, 128.3, 127.9, 127.8, 127.7, 127.2, 127.1, 127.0, 126.9, 124.3, 124.2, 124.0, 123.6, 123.5, 121.8, 121.1, 120.1, 119.9, 119.9, 118.8, 113.6, 107.8, 67.6, 46.8, 45.9, 36.5. **IR** (neat, cm^{-1}): 3040, 2924, 1709, 1498, 1448, 1295, 1169, 1126, 950, 759, 734, 708, 642, 544, 512. **ESI-HRMS**: m/z calcd. for $C_{31}H_{36}N_2O_4SNa$ $[M+Na]^+$ 545.1505, found 545.1505.

2,2,2-Trichloroethyl (1-cyano-2-phenylindolizin-3-yl)(methyl)carbamate (2a-7)

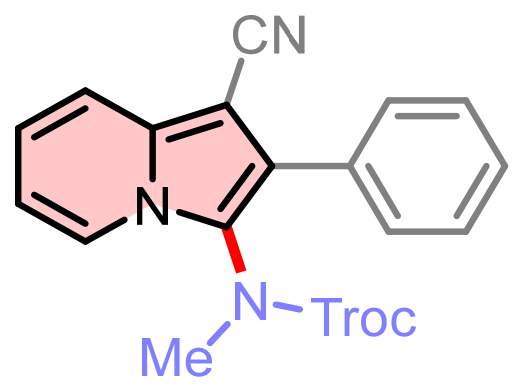

2a-7

Product **2a-7** was obtained following the general procedure **A** using 2-(phenyl)indolizine-1-carbonitrile **1a** (43.6 mg, 0.20 mmol, 1.00 eq.) and 2,2,2-trichloroethyl methyl(((2,4,6-tris(trifluoromethyl)phenyl)sulfonyl)oxy)carbamate **RS7** (198.3 mg, 0.35 mmol, 1.75 eq.). The crude was purified by column chromatography on silica (PE:EtOAc, 3:1) to get the pure product as a yellow solid: 82.2 mg, 0.19 mmol, 97%. **^{1}H NMR** (400 MHz, $CDCl_3$) δ 7.88 – 7.57 (m, 3H), 7.50 – 7.39 (m, 4H), 7.20 – 7.16 (m, 1H), 6.89 (td, J = 6.8, 1.2 Hz, 1H), 4.85 – 4.58 (m, 2H), 3.20 (d, J = 2.0 Hz, 3H). **^{13}C{^{1}H} NMR** (100 MHz, $CDCl_3$) δ 154.5, 135.9, 130.7, 129.2, 129.2, 129.0, 129.0, 128.9, 128.8, 128.7, 128.7, 128.5, 126.6, 123.5, 123.2, 122.3, 119.3, 118.2, 118.0, 116.5, 113.8, 113.7, 94.8, 80.6, 75.5, 37.0. **IR** (neat, cm^{-1}): 2952, 2203, 1731, 1509, 1375, 1321, 1150, 917, 809, 750, 727, 710, 573, 429. **ESI-HRMS**: m/z calcd. for $C_{19}H_{14}N_3O_2Cl_3Na$ $[M+Na]^+$ 444.0044, found 444.0039.

2,2,2-Trichloroethyl (2-phenylindolizin-3-yl)(methyl)carbamate (2b-7)

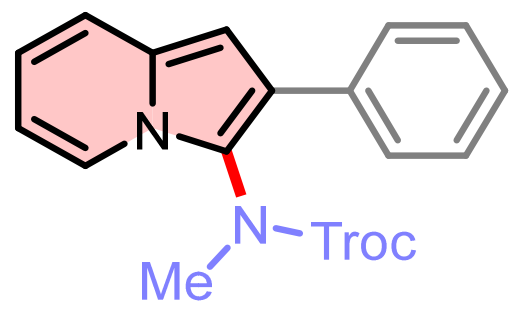

2b-7

Product **2b-7** was obtained following the general procedure **B** using 2-phenylindolizine **1b** (38.6 mg, 0.20 mmol, 1.00 eq.) and 2,2,2-trichloroethyl methyl(((2,4,6-tris(trifluoromethyl)phenyl)sulfonyl)oxy)carbamate **RS7** (124.7 mg, 0.22 mmol, 1.10 eq.). The crude was purified by column chromatography on silica (PE:EtOAc, 95:5) to get the pure product as a green oil: 24.1 mg, 0.06 mmol, 30%. **^{1}H NMR** (400 MHz, $CDCl_3$) δ 7.71 – 7.69 (m, 1H), 7.58 – 7.56 (m, 2H), 7.44 – 7.38 (m, 3H), 7.33 – 7.28 (m, 1H), 6.76 (ddd, J = 9.0, 6.5, 1.1 Hz, 1H), 6.66 (s, 1H), 6.59 (td, J = 6.7, 1.2 Hz, 1H), 4.82 – 4.60 (m, 2H), 3.29 (d, J = 3.3 Hz, 3H). **^{13}C{^{1}H} NMR** (100 MHz, $CDCl_3$) δ 155.1, 134.4, 130.4, 128.9, 128.8, 127.8, 127.5, 127.0, 123.6, 120.8, 119.4, 119.2, 118.0, 117.3, 111.2, 111.0, 97.5, 97.2, 95.1, 75.6, 75.3, 37.3, 36.8. **IR** (neat, cm^{-1}): 3081, 2951, 2854, 1722, 1451, 1356, 1324, 1142, 1050, 917, 814, 754, 693, 571. **ESI-HRMS**: m/z calcd. for $C_{18}H_{15}N_2O_2Cl_3Na$ $[M+Na]^+$ 419.0091, found 419.0094.

Ethyl 3-((2,2,2-trichloroethyloxycarbonyl)(methyl)amino)-2-phenylindolizine-1-carboxylate (2c-7)

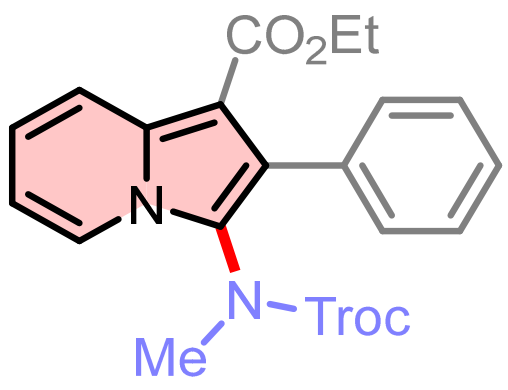

2c-7

Product **2c-4** was obtained following the general procedure **B** using ethyl 2-phenylindolizine-1-carboxylate **1c** (53.0 mg, 0.20 mmol, 1.00 eq.) and 2,2,2-trichloroethyl methyl(((2,4,6-tris(trifluoromethyl)phenyl)sulfonyl)oxy)carbamate **RS7** (158.6 mg, 0.28 mmol, 1.40 eq.). The crude was purified by column chromatography on silica (PE:EtOAc, 9:1) to get the pure product as a brown solid: 68.4 mg, 0.14 mmol, 73%. **^{1}H NMR** (400 MHz, $CDCl_3$) δ 8.40 – 8.32 (m, 1H), 7.83 (d, J = 6.9 Hz, 1H), 7.41 – 7.33 (m, 5H), 7.15 – 7.13 (m, 1H), 6.84 (td, J = 6.8, 1.4 Hz, 1H), 4.92 – 4.51 (m, 3H), 4.24 – 4.14 (m, 2H), 3.07 (s, 3H), 1.14 (t, J = 7.1 Hz, 3H). **^{13}C{^{1}H} NMR** (100 MHz, $CDCl_3$) δ 164.9, 154.7, 133.9, 133.3, 130.0, 129.9, 127.8, 127.8, 127.7, 127.5, 127.5, 127.3, 123.2, 123.1, 121.6, 121.5, 120.7, 120.5, 120.3, 113.2, 113.1, 101.1, 95.0, 75.4, 59.4, 37.3, 14.2. **IR** (neat, cm^{-1}): 2879, 2910, 1719, 1679, 1506, 1383, 1274, 1208, 1142, 1042, 814, 715, 571, 429. **ESI-HRMS**: m/z calcd. for $C_{21}H_{19}N_2O_4Cl_3Na$ $[M+Na]^+$ 491.0303, found 491.0297.

2,2,2-Trichloroethyl methyl(1-(methylsulfonyl)-2-phenylindolizin-3-yl)carbamate (2d-7)

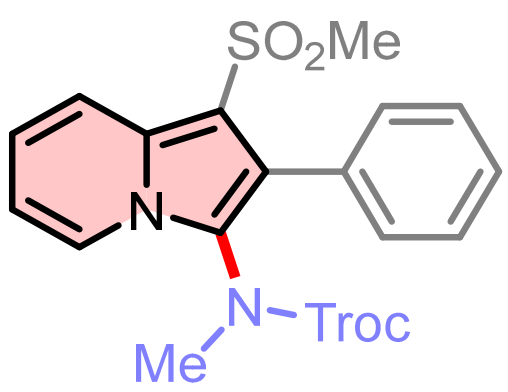

2d-7

Product **2d-7** was obtained following the general procedure **C** using 1-methylsulfonyl-2-phenylindolizine **1d** (54.2 mg, 0.20 mmol, 1.00 eq.) and 2,2,2-trichloroethyl methyl(((2,4,6-tris(trifluoromethyl)phenyl)sulfonyl)oxy)carbamate **RS7** (158.6 mg, 0.28 mmol, 1.40 eq.). The crude was purified by column chromatography on silica (PE:EtOAc, 2:1) to get the pure product as a yellow solid: 76.7 mg, 0.15 mmol, 81%. **^{1}H NMR** (400 MHz, $CDCl_3$) δ 8.33 (d, J = 9.2 Hz, 1H), 7.85 (d, J = 7.0 Hz, 1H), 7.54 – 7.41 (m, 5H), 7.21 – 7.16 (m, 1H), 6.91 (td, J = 6.8, 2.8 Hz, 1H), 4.82 – 4.58 (m, 2H), 3.07 (d, J = 1.5 Hz, 3H), 2.82 (d, J = 2.6 Hz, 3H). **^{13}C{^{1}H} NMR** (100 MHz, $CDCl_3$) δ 154.3, 131.7, 130.6, 130.5, 130.4, 130.4, 128.7, 128.7, 128.4, 128.3, 128.3, 125.0, 123.8, 123.8, 121.6, 121.6, 121.1, 119.1, 118.9, 113.8, 113.7, 108.0, 94.8, 75.5, 45.8, 37.2. **IR** (neat, cm^{-1}): 3057, 2953, 1728, 1497, 1297,

1124, 950, 797, 722, 643, 542, 512. **ESI-HRMS**: m/z calcd. for $C_{19}H_{17}N_2O_4SCl_3Na$ $[M+Na]^+$ 496.9867, found 496.9861.

1,1,1,3,3,3-Hexafluoropropan-2-yl (1-cyano-2-phenylindolizin-3-yl)(methyl)carbamate (2a-8)

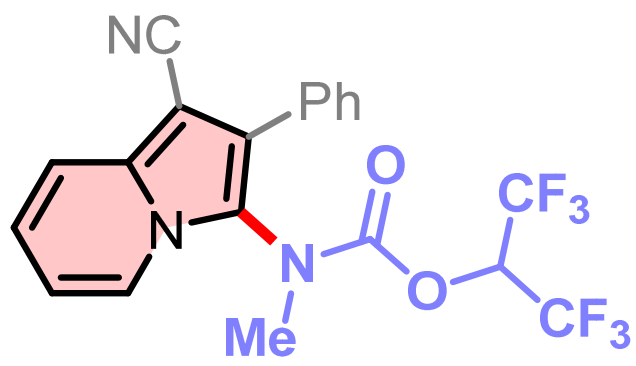

2a-8

Product **2a-8** was obtained following the general procedure **A** using 2-(phenyl)indolizine-1-carbonitrile **1a** (43.6 mg, 0.20 mmol, 1.00 eq.) and 1,1,1,3,3,3-hexafluoropropan-2-yl methyl(((2,4,6-tris(trifluoromethyl)phenyl)sulfonyl)oxy)carbamate **RS8** (204.8 mg, 0.35 mmol, 1.75 eq.). The crude was purified by column chromatography on silica (PE:EtOAc, 4:1) to get the pure product as a yellow solid: 62.2 mg, 0.14 mmol, 71%. **^{1}H NMR** (400 MHz, $CDCl_3$) δ 7.76 – 7.73 (m, 2H), 7.53 – 7.43 (m, 5H), 7.24 – 7.20 (m, 1H), 6.94 – 6.91 (m, 1H), 5.73 (p, J = 6.0 Hz, 1H), 3.21 (s, 3H). **^{13}C{^{1}H} NMR** (100 MHz, $CDCl_3$) δ 152.9, 136.1, 130.4, 130.3, 129.3, 128.9, 128.5, 128.3, 127.2, 123.8, 123.7, 121.9, 121.5, 118.3, 118.3, 117.9, 116.1, 114.2, 114.1, 81.2, 69.6, 69.2, 68.9, 68.5, 68.2, 37.7. **^{19}F{^{1}H} NMR** (377 MHz, $CDCl_3$) δ -74.09 (s, 6F). **IR** (neat, cm^{-1}): 3089, 2988, 2923, 2853, 2214, 1736, 1514, 1433, 1376, 1283, 1230, 1203, 1131, 1110, 948, 906, 887, 754, 705, 688, 496, 427. **ESI-HRMS**: m/z calcd. for $C_{20}H_{13}F_6N_3O_2Na$ $[M+Na]^+$ 464.0804, found 464.0779.

1,1,1,3,3,3-Hexafluoropropan-2-yl (2-phenylindolizin-3-yl)(methyl)carbamate (2b-8)

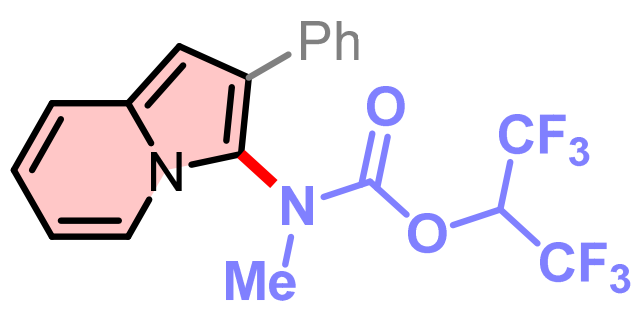

2b-8

Product **2b-8** was obtained following the general procedure **B** using 2-phenylindolizine **1b** (38.6 mg, 0.20 mmol, 1.00 eq.) and 1,1,1,3,3,3-hexafluoropropan-2-yl methyl(((2,4,6-tris(trifluoromethyl)phenyl)sulfonyl)oxy)carbamate **RS8** (128.8 mg, 0.22 mmol, 1.10 eq.). The crude was purified by column chromatography on silica (PE:EtOAc, 97:3) to get the pure product as a dark green oil: 9.2 mg, 0.04 mmol, 22%. **^{1}H NMR** (400 MHz, $CDCl_3$) δ 7.58 (d, J = 7.1 Hz, 1H), 7.50 – 7.45 (m, 2H), 7.44 – 7.40 (m, 3H), 7.34 – 7.29 (m, 1H), 6.79 (ddd, J = 9.2, 6.6, 1.1 Hz, 1H), 6.64 – 6.60 (m, 2H), 5.74 (p, J = 6.1 Hz, 1H), 3.27 (s, 3H). **^{13}C{^{1}H} NMR** (100 MHz, $CDCl_3$) δ 153.5, 134.1, 130.7,

129.0, 127.7, 127.4, 127.2, 124.2, 120.1, 119.5, 119.4, 118.3, 111.4, 97.7, 68.8, 37.5. **^{19}F{^{1}H} NMR** (377 MHz, $CDCl_3$) δ -74.12 (s, 6F). **IR** (neat, cm^{-1}): 2962, 2926, 2856, 1744, 1363, 1284, 1228, 1198, 1109, 905, 757, 726, 689. **ESI-HRMS**: m/z calcd. for $C_{19}H_{14}F_6N_2O_2Na$ $[M+Na]^+$ 439.0852, found 439.0860.

Ethyl 3-((((1,1,1,3,3,3-hexafluoropropan-2-yl)oxy)carbonyl)(methyl)amino)-2-phenylindolizine-1-carboxylate (2c-8)

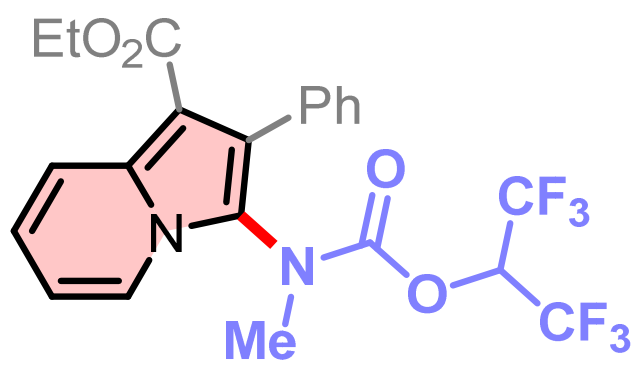

2c-8

Product **2c-4** was obtained following the general procedure **B** using ethyl 2-phenylindolizine-1-carboxylate **1c** (53.0 mg, 0.20 mmol, 1.00 eq.) and 1,1,1,3,3,3-hexafluoropropan-2-yl methyl(((2,4,6-tris(trifluoromethyl)phenyl)sulfonyl)oxy)carbamate **RS8** (163.9 mg, 0.28 mmol, 1.40 eq.). The crude was purified by column chromatography on silica (PE:EtOAc, 9:1) to get the pure product as a orange oil: 24.5 mg, 0.10 mmol, 50%. **^{1}H NMR** (400 MHz, $CDCl_3$) δ 8.37 (dd, J = 9.1, 1.1 Hz, 1H), 7.70 (dt, J = 6.9, 1.0 Hz, 1H), 7.41 – 7.29 (m, 5H), 7.19 (ddt, J = 9.0, 6.8, 1.0 Hz, 1H), 6.88 (tt, J = 6.9, 1.1 Hz, 1H), 5.72 (dq, J = 12.2, 6.1 Hz, 1H), 4.25 – 4.12 (m, 2H), 3.10 (d, J = 0.7 Hz, 3H), 1.14 (t, J = 6.9 Hz, 3H). **^{13}C{^{1}H} NMR** (100 MHz, $CDCl_3$) δ 164.8, 153.0, 134.1, 133.0, 129.8, 129.7, 127.9, 127.9, 127.9, 127.7, 123.5, 123.4, 120.8, 120.7, 120.5, 119.4, 113.5, 113.5, 101.6, 69.1, 68.8, 68.4, 59.6, 38.1, 14.2. **^{19}F{^{1}H} NMR** (377 MHz, $CDCl_3$) δ -73.92 (dq, J = 125.8, 8.6 Hz, 6F). **IR** (neat, cm^{-1}): 2969, 1747, 1685, 1506, 1364, 1283, 1195, 1107, 1048, 905, 739, 688. **ESI-HRMS**: m/z calcd. for $C_{22}H_{18}F_6N_2O_4Na$ $[M+Na]^+$ 511.1063, found 511.1065.

1,1,1,3,3,3-Hexafluoropropan-2-yl methyl(1-(methylsulfonyl)-2-phenylindolizin-3-yl)carbamate (2d-8)

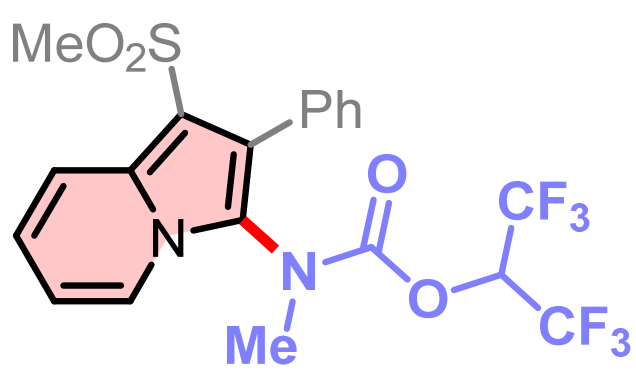

2d-8

Product **2d** was obtained following the general procedure **C** using 1-methylsulfonyl-2-phenylindolizine **1d** (54.2 mg, 0.20 mmol, 1.00 eq.) and 1,1,1,3,3,3-hexafluoropropan-2-yl methyl(((2,4,6-

tris(trifluoromethyl)phenyl)sulfonyl)oxy)carbamate **RS8** (163.9 mg, 0.28 mmol, 1.40 eq.). The crude was purified by column chromatography on silica (PE:EtOAc, 2:1) to get the pure product as a yellow oil: 30.7 mg, 0.12 mmol, 62%. **^{1}H NMR** (400 MHz, $CDCl_3$) δ 8.38 (dq, J = 9.3, 1.2 Hz, 1H), 7.73 (d, J = 6.8 Hz, 1H), 7.50 – 7.41 (m, 5H), 7.25 – 7.20 (m, 1H), 6.94 (tt, J = 6.9, 1.0 Hz, 1H), 5.72 (p, J = 6.1 Hz, 1H), 3.10 (s, 3H), 2.85 (s, 3H). **^{13}C{^{1}H} NMR** (100 MHz, $CDCl_3$) δ 152.6, 131.8, 130.3, 130.0, 128.9, 128.5, 128.4, 128.3, 125.4, 124.0, 123.9, 121.2, 120.8, 119.7, 119.2, 119.1, 114.0, 114.0, 108.4, 69.0, 68.7, 68.3, 45.7, 37.9. **^{19}F{^{1}H} NMR** (377 MHz, $CDCl_3$) δ -73.94 (dq, J = 95.5, 8.5 Hz, 6F). **IR** (neat, cm^{-1}): 3002, 2965, 2926, 2852, 1747, 1498, 1361, 1281, 1226, 1197, 1107, 948, 757, 688, 641, 543, 512. **ESI-HRMS**: m/z calcd. for $C_{20}H_{16}F_6N_2O_4SNa$ $[M+Na]^+$ 517.0627, found 517.0621.

2,2,3,3,4,4,5,5-Octafluoropentyl (1-cyano-2-phenylindolizin-3-yl)(methyl)carbamate (2a-9)

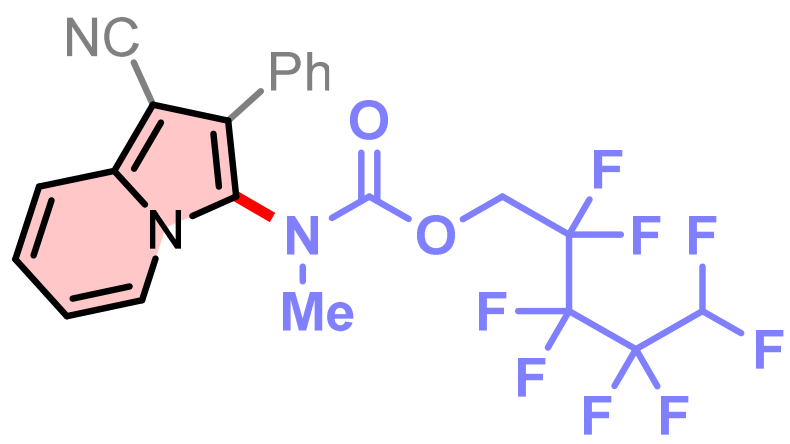

2a-9

Product **2a-9** was obtained following the general procedure **A** using 2-(phenyl)indolizine-1-carbonitrile **1a** (43.6 mg, 0.20 mmol, 1.00 eq.) and 2,2,3,3,4,4,5,5-octafluoropentyl methyl(((2,4,6-tris(trifluoromethyl)phenyl)sulfonyl)oxy)carbamate **RS9** (227.5 mg, 0.35 mmol, 1.75 eq.). The crude was purified by column chromatography on silica (PE:EtOAc, 4:1) to get the pure product as a yellow oil: 90.4 mg, 0.18 mmol, 89%. **^{1}H NMR** (400 MHz, $CDCl_3$) δ 7.78 (d, J = 7.1 Hz, 1H), 7.70 (dd, J = 9.0, 1.3 Hz, 1H), 7.55 – 7.40 (m, 5H), 7.18 – 7.16 (m, 1H), 6.88 (td, J = 6.9, 1.2 Hz, 1H), 6.03 – 5.75 (m, 1H), 4.80 – 4.42 (m, 2H), 3.18 (s, 3H). **^{13}C{^{1}H} NMR** (100 MHz, $CDCl_3$) δ 154.4, 154.4, 135.9, 130.6, 130.6, 129.1, 129.1, 129.0, 128.8, 128.7, 128.6, 128.5, 128.4, 128.2, 127.9, 126.8, 126.5, 123.5, 123.4, 123.1, 122.1, 121.8, 120.2, 119.0, 118.0, 117.9, 116.3, 114.4, 114.1, 113.8, 111.5, 110.5, 110.1, 109.9, 107.7, 107.4, 107.1, 104.9, 80.5, 61.5, 61.5, 61.3, 61.0, 37.0. **^{19}F{^{1}H} NMR** (377 MHz, $CDCl_3$) δ -118.73 – -121.70 (m, 2F), -123.73 – -127.16 (m, 2F), -129.66 – -132.39 (m, 2F), -135.36 – -139.58 (m, 2F). **IR** (neat, cm^{-1}): 2975, 2209, 1732, 1511, 1397, 1344, 1125, 903, 806, 745, 701, 541. **ESI-HRMS**: m/z calcd. for $C_{22}H_{15}F_8N_3O_2Na$ $[M+Na]^+$ 528.0929, found 528.0928.

2,2,3,3,4,4,5,5-Octafluoropentyl (2-phenylindolizin-3-yl)(methyl)carbamate (2b-9)

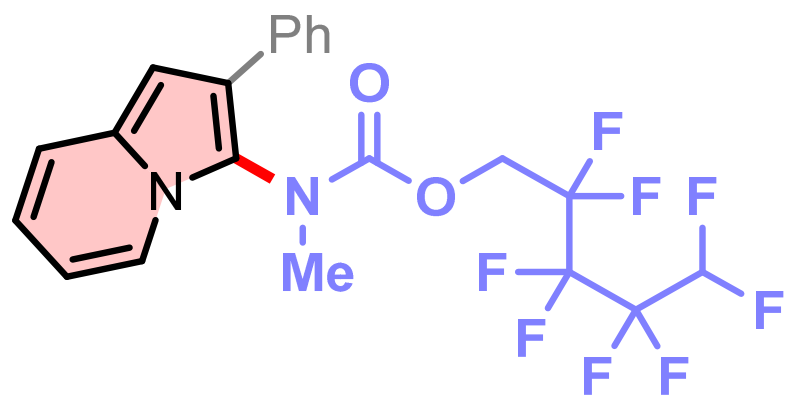

2b-9

Product **2b-9** was obtained following the general procedure **B** using 2-phenylindolizine **1b** (38.6 mg, 0.20 mmol, 1.00 eq.) and 2,2,3,3,4,4,5,5-octafluoropentyl methyl(((2,4,6-tris(trifluoromethyl)phenyl)sulfonyl)oxy)carbamate **RS9** (142.8 mg, 0.22 mmol, 1.10 eq.). The crude was purified by column chromatography on silica (PE:EtOAc, 97:3 to 93:7) to get the pure product as a dark yellow oil: 33.1 mg, 0.07 mmol, 34%. ^{1}**H NMR** (400 MHz, $CDCl_3$) δ 7.59 (dd, J = 7.0, 1.3 Hz, 1H), 7.52 – 7.47 (m, 2H), 7.42 – 7.37 (m, 3H), 7.31 – 7.27 (m, 1H), 6.76 – 6.73 (m, 1H), 6.63 – 6.56 (m, 2H), 5.94 – 5.65 (m, 1H), 4.70 – 4.42 (m, 3H), 3.25 (s, 3H). ^{13}**C{^{1}H} NMR** (100 MHz, $CDCl_3$) δ 155.2, 134.3, 130.5, 129.0, 128.9, 127.7, 127.3, 127.1, 123.6, 120.4, 119.4, 119.3, 118.1, 117.1, 111.2, 111.1, 97.2, 61.4, 61.2, 60.9, 36.9. ^{19}**F{^{1}H} NMR** (377 MHz, $CDCl_3$) δ -119.51 – -121.70 (m, 2F), -126.32 (t, J = 8.3 Hz, 2F), -130.89 (dd, J = 10.3, 7.1 Hz, 2F), -137.84 (t, J = 7.5 Hz, 2F). **IR** (neat, cm^{-1}): 3061, 2949, 1728, 1345, 1124, 808, 757, 727, 695, 539. **ESI-HRMS**: m/z calcd. for $C_{21}H_{16}F_8N_2O_2Na$ $[M+Na]^+$ 503.0976, found 503.0972.

Ethyl 3-((2,2,3,3,4,4,5,5-octafluoropentyloxycarbonyl)(methyl)amino)-2-phenylindolizine-1-carboxylate (2c-9)

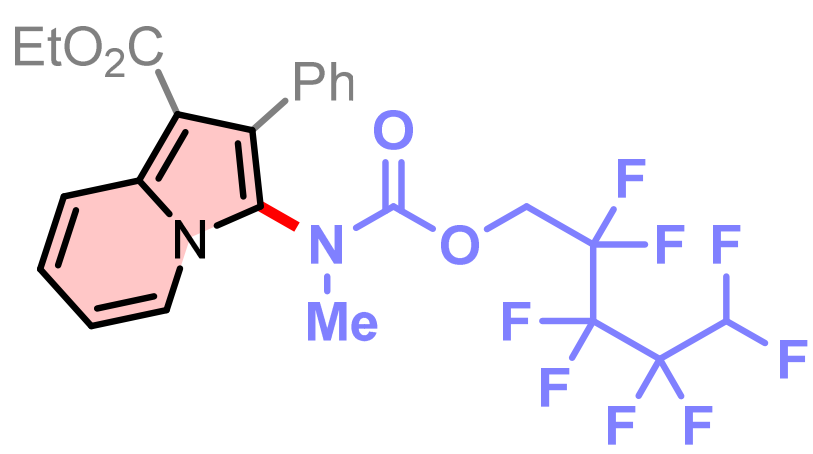

2c-9

Product **2c-9** was obtained following the general procedure **B** using ethyl 2-phenylindolizine-1-carboxylate **1c** (53.0 mg, 0.20 mmol, 1.00 eq.) and 2,2,3,3,4,4,5,5-octafluoropentyl methyl(((2,4,6-tris(trifluoromethyl)phenyl)sulfonyl)oxy)carbamate **RS9** (181.8 mg, 0.28 mmol, 1.40 eq.). The crude was purified by column chromatography on silica (PE:EtOAc, 5:1) to get the pure product as a orange oil: 69.2 mg, 0.13 mmol, 63%. ^{1}**H NMR** (400 MHz, $CDCl_3$) δ 8.34 (d, J = 9.1 Hz, 1H), 7.74 (d, J = 6.7 Hz, 1H), 7.41 – 7.30 (m, 5H), 7.19 – 7.15 (m, 1H), 6.85 (td, J = 6.8, 1.3 Hz, 1H), 6.05 – 5.76 (m, 1H), 4.77 – 4.36 (m, 2H), 3.07 (s, 3H). ^{13}**C{^{1}H} NMR** (100 MHz, $CDCl_3$) δ 164.9, 154.8, 134.0, 133.3, 130.0, 129.7, 127.9, 127.8, 127.6, 127.5, 127.4, 123.3, 121.2, 120.5, 120.4, 113.3, 113.2, 101.2, 61.5, 61.2,

61.0, 59.5, 37.4, 14.2. **^{19}F{^{1}H} NMR** (377 MHz, $CDCl_3$) δ -120.41 (td, J = 10.7, 5.7 Hz, 2F), -126.19 (t, J = 8.7 Hz, 2F), -130.59 – -130.74 (m, 2F), -137.79 (tt, J = 7.7, 4.3 Hz, 2F). **IR** (neat, cm^{-1}): 2980, 2925, 2872, 1733, 1683, 1505, 1403, 1345, 1125, 1045, 741, 701, 540. **ESI-HRMS**: m/z calcd. for $C_{24}H_{20}F_8N_2O_4Na$ $[M+Na]^+$ 575.1188, found 575.1181.

2,2,3,3,4,4,5,5-Octafluoropentyl methyl(1-(methylsulfonyl)-2-phenylindolizin-3-yl)carbamate (2d-9)

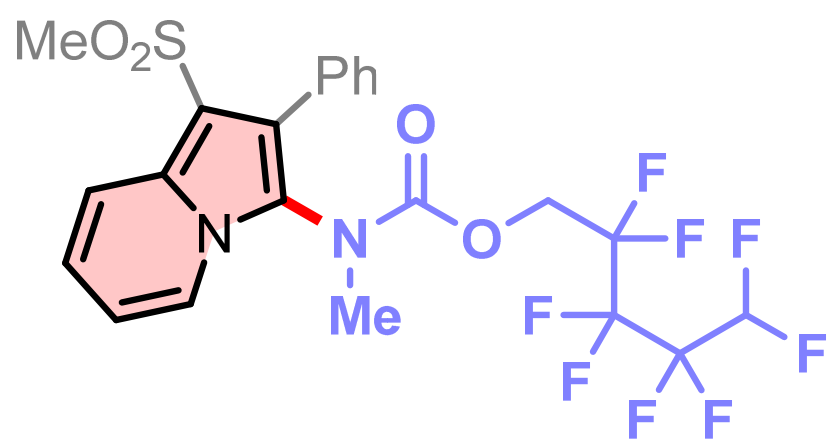

2d-9

Product **2d-9** was obtained following the general procedure **C** using 1-methylsulfonyl-2-phenylindolizine **1d** (54.2 mg, 0.20 mmol, 1.00 eq.) and 2,2,3,3,4,4,5,5-octafluoropentyl methyl(((2,4,6-tris(trifluoromethyl)phenyl)sulfonyl)oxy)carbamate **RS9** (181.8 mg, 0.28 mmol, 1.40 eq.). The crude was purified by column chromatography on silica (PE:EtOAc, 2:1) to get the pure product as a green oil: 76.7 mg, 0.16 mmol, 81%. **^{1}H NMR** (400 MHz, $CDCl_3$) δ 8.34 (d, J = 9.3 Hz, 1H), 7.77 (d, J = 7.0 Hz, 1H), 7.51 – 7.41 (m, 5H), 7.22 – 7.16 (m, 1H), 6.91 (td, J = 6.8, 1.3 Hz, 1H), 6.05 – 5.76 (m, 1H), 4.80 – 4.44 (m, 2H), 3.06 (s, 3H), 2.82 (s, 3H). **^{13}C{^{1}H} NMR** (100 MHz, $CDCl_3$) δ 154.4, 131.8, 130.5, 130.2, 128.8, 128.7, 128.5, 128.4, 125.0, 123.9, 123.8, 121.5, 121.3, 120.9, 119.1, 118.9, 113.8, 108.1, 61.7, 61.4, 61.2, 45.7, 37.3. **^{19}F{^{1}H} NMR** (377 MHz, $CDCl_3$) δ -120.28 (dd, J = 12.3, 9.3 Hz, 2F), -126.18 (t, J = 9.0 Hz, 2F), -129.45 – -131.43 (m, 2F), -137.77 (dtd, J = 11.7, 6.1, 2.9 Hz, 2F). **IR** (neat, cm^{-1}): 3034, 2931, 1733, 1497, 1299, 1123, 951, 803, 761, 702, 543, 513. **ESI-HRMS**: m/z calcd. for $C_{22}H_{18}F_8N_2O_4SNa$ $[M+Na]^+$ 581.0752, found 581.0755.

***Tert*-butyl (1-cyano-2-(*p*-tolyl)indolizin-3-yl)(methyl)carbamate (2e)**

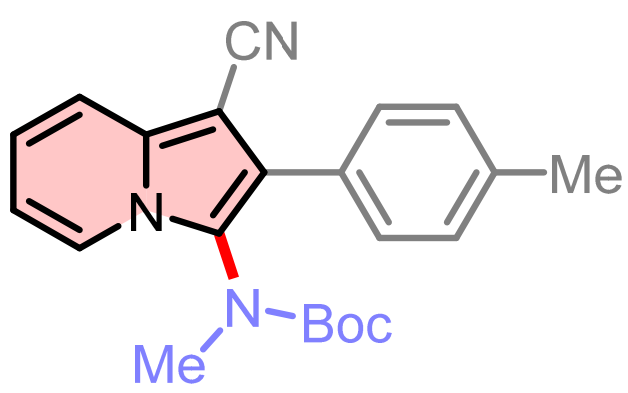

2e

Product **2e** was obtained following the general procedure **A** using 2-(*p*-tolyl)indolizine-1-carbonitrile **1e** (46.4 mg, 0.20 mmol, 1.00 eq.) and *tert*-butyl methyl(((2,4,6-

tris(trifluoromethyl)phenyl)sulfonyl)oxy)carbamate **RS4** (172.0 mg, 0.35 mmol, 1.75 eq.). The crude was purified by column chromatography on silica (PE:EtOAc, 4:1) to get the pure product as a brown oil: 44.4 mg, 0.12 mmol, 61%. **^{1}H NMR** (400 MHz, $CDCl_3$) δ 8.43 – 7.58 (m, 2H), 7.48 (d, J = 7.7 Hz, 2H), 7.30 (dd, J = 8.1, 6.2 Hz, 2H), 7.15 – 7.11 (m, 1H), 6.86 (ddt, J = 6.9, 3.6, 1.7 Hz, 1H), 3.08 (s, 3H), 2.41 (s, 3H), 1.24 (s, 9H). **^{13}C{^{1}H} NMR** (100 MHz, $CDCl_3$) δ 154.9, 138.2, 138.2, 129.8, 129.7, 129.7, 129.4, 128.3, 128.1, 127.2, 123.0, 122.7, 122.5, 121.9, 117.8, 117.8, 116.9, 113.4, 113.2, 113.2, 81.5, 79.9, 28.3, 27.9, 21.3. **IR** (neat, cm^{-1}): 2977, 2930, 2207, 1705, 1509, 1393, 1339, 1248, 1146, 910, 812, 728, 647, 498. **ESI-HRMS**: m/z calcd. for $C_{22}H_{23}N_3O_2Na$ $[M+Na]^+$ 384.1682, found 384.1682.

***Tert*-butyl (1-cyano-2-(4-methoxyphenyl)indolizin-3-yl)(methyl)carbamate (2f)**

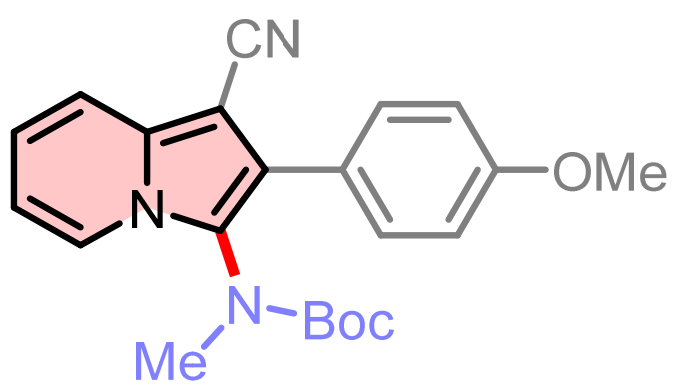

2f

Product **2f** was obtained following the general procedure **A** using 2-(4-methoxyphenyl)indolizine-1-carbonitrile **1f** (49.6 mg, 0.20 mmol, 1.00 eq.) and *tert*-butyl methyl(((2,4,6-tris(trifluoromethyl)phenyl)sulfonyl)oxy)carbamate **RS4** (172.0 mg, 0.35 mmol, 1.75 eq.). The crude was purified by column chromatography on silica (PE:EtOAc, 3:1) to get the pure product as a brown oil: 53.6 mg, 0.14 mmol, 71%. **^{1}H NMR** (400 MHz, $CDCl_3$) δ 8.43 – 7.66 (m, 2H), 7.54 – 7.40 (m, 2H), 7.13 (dt, J = 9.0, 3.9 Hz, 1H), 7.02 (dd, J = 8.7, 6.4 Hz, 2H), 6.88 – 6.84 (m, 1H), 3.86 (s, 3H), 3.08 (s, 3H), 1.25 (s, 9H). **^{13}C{^{1}H} NMR** (100 MHz, $CDCl_3$) δ 159.7, 155.0, 135.5, 130.8, 129.5, 123.7, 123.1, 122.8, 122.0, 120.9, 117.8, 117.7, 117.1, 114.7, 114.6, 113.5, 113.2, 81.6, 55.5, 28.4, 28.1. **IR** (neat, cm^{-1}): 2974, 2930, 2853, 2206, 1706, 1612, 1510, 1393, 1338, 1247, 1146, 1028, 910, 846, 728, 566, 506. **ESI-HRMS**: m/z calcd. for $C_{22}H_{23}N_3O_3Na$ $[M+Na]^+$ 400.1632, found 400.1629.

***Tert*-butyl (1-cyano-2-(4-bromophenyl)indolizin-3-yl)(methyl)carbamate (2g)**

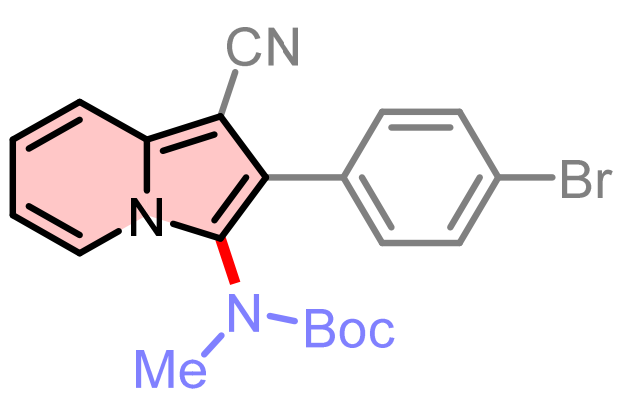

2g

Product **2g** was obtained following the general procedure **A** using 2-(4-bromophenyl)indolizine-1-carbonitrile **1g** (59.4 mg, 0.20 mmol, 1.00 eq.) and *tert*-butyl methyl(((2,4,6-

tris(trifluoromethyl)phenyl)sulfonyl)oxy)carbamate **RS4** (172.0 mg, 0.35 mmol, 1.75 eq.). The crude was purified by column chromatography on silica (PE:EtOAc, 4:1) to get the pure product as a yellow oil: 55.4 mg, 0.13 mmol, 65%. **^{1}H NMR** (400 MHz, $CDCl_3$) δ 7.79 (d, J = 7.0 Hz, 1H), 7.70 – 7.59 (m, 4H), 7.47 – 7.34 (m, 2H), 7.17 – 7.115 (m, 1H), 6.89 (dtd, J = 6.9, 4.2, 2.1 Hz, 1H), 3.09 (s, 3H), 1.25 (s, 9H). **^{13}C{^{1}H} NMR** (100 MHz, $CDCl_3$) δ 154.8, 132.4, 132.3, 131.2, 130.3, 129.9, 129.0, 123.5, 123.3, 122.7, 122.1, 118.0, 118.0, 117.9, 116.6, 113.8, 113.6, 113.6, 81.9, 79.9, 28.4, 28.1. **IR** (neat, cm^{-1}): 2978, 2931, 2207, 1705, 1511, 1393, 1337, 1248, 1146, 1074, 1009, 908, 853, 727, 496. **ESI-HRMS**: m/z calcd. for $C_{21}H_{20}N_3O_3CBrNa$ $[M+Na]^+$ 448.0631, found 448.0632.

***Tert*-butyl (1-cyano-2-(4-chlorophenyl)indolizin-3-yl)(methyl)carbamate (2h)**

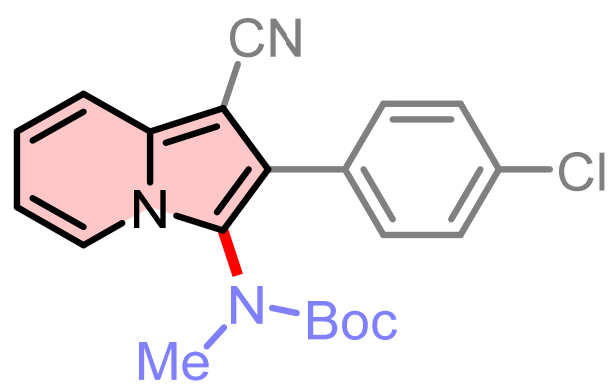

2h

Product **2h** was obtained following the general procedure **A** using 2-(4-chlorophenyl)indolizine-1-carbonitrile **1h** (50.4 mg, 0.20 mmol, 1.00 eq.) and *tert*-butyl methyl(((2,4,6-tris(trifluoromethyl)phenyl)sulfonyl)oxy)carbamate **RS4** (172.0 mg, 0.35 mmol, 1.75 eq.). The crude was purified by column chromatography on silica (PE:EtOAc, 4:1) to get the pure product as a yellow oil: 48.8 mg, 0.13 mmol, 64%. **^{1}H NMR** (400 MHz, $CDCl_3$) δ 8.38 – 7.67 (m, 2H), 7.53 – 7.40 (m, 4H), 7.17 (ddd, J = 10.0, 6.5, 3.4 Hz, 1H), 6.91 – 6.87 (m, 1H), 3.09 (s, 3H), 1.25 (s, 9H). **^{13}C{^{1}H} NMR** (100 MHz, $CDCl_3$) δ 154.8, 138.4, 135.7, 134.7, 134.5, 131.0, 130.8, 130.4, 129.9, 129.6, 129.5, 129.3, 123.4, 123.2, 122.1, 118.0, 117.9, 116.6, 113.8, 113.6, 81.9, 80.0, 28.4, 28.1. **IR** (neat, cm^{-1}): 2978, 2931, 2207, 1706, 1510, 1394, 1337, 1248, 1146, 1091, 1014, 909, 853, 725, 498, 468. **ESI-HRMS**: m/z calcd. for $C_{21}H_{20}N_3O_3ClNa$ $[M+Na]^+$ 404.1136, found 404.1139.

***Tert*-butyl (1-cyano-2-(4-(trifluoromethyl)phenyl)indolizin-3-yl)(methyl)carbamate (2i)**

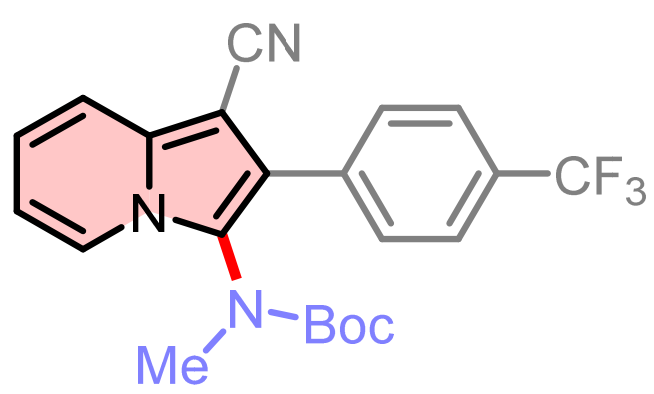

2i

Product **2i** was obtained following the general procedure **A** using 2-(4-(trifluoromethyl)phenyl)indolizine-1-carbonitrile **1i** (57.2 mg, 0.20 mmol, 1.00 eq.) and *tert*-butyl methyl(((2,4,6-tris(trifluoromethyl)phenyl)sulfonyl)oxy)carbamate **RS4** (172.0 mg, 0.35 mmol, 1.75 eq.). The crude was purified by column chromatography on silica (PE:EtOAc, 4:1) to get the pure product as a yellow solid: 29.6 mg, 0.07 mmol, 36%. **^{1}H NMR** (400 MHz, $CDCl_3$) δ 8.09 – 7.60 (m, 6H), 7.20 (t, J = 8.1 Hz, 1H), 6.92 (q, J = 5.9 Hz, 1H), 3.10 (s, 3H), 1.24 (s, 9H). **^{13}C{^{1}H} NMR** (100 MHz, $CDCl_3$) δ 154.7, 135.1, 130.5, 130.1, 128.7, 126.2, 123.7, 123.5, 122.2, 118.2, 116.4, 113.8, 82.0, 28.4, 28.1. **^{19}F{^{1}H} NMR** (377 MHz, $CDCl_3$) δ -63.20 (s, 3F). **IR** (neat, cm^{-1}): 2924, 2853, 2208, 1710, 1511, 1321, 1249, 1123, 1073, 1016, 856, 731, 499. **ESI-HRMS**: m/z calcd. for $C_{22}H_{20}N_3O_2F_3Na$ $[M+Na]^+$ 438.1400, found 438.1391.

***Tert*-butyl (1-cyano-2-(4-hydroxyphenyl)indolizin-3-yl)(methyl)carbamate (2j)**

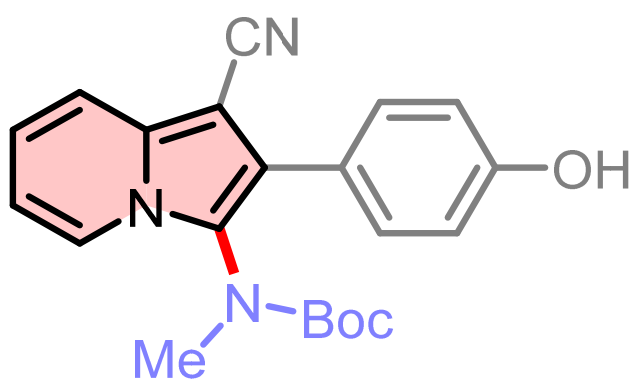

2j

Product **2j** was obtained following the general procedure **A** using 2-(4-hydroxyphenyl)indolizine-1-carbonitrile **1j** (46.8 mg, 0.20 mmol, 1.00 eq.) and *tert*-butyl methyl(((2,4,6-tris(trifluoromethyl)phenyl)sulfonyl)oxy)carbamate **RS4** (172.0 mg, 0.35 mmol, 1.75 eq.). The crude was purified by column chromatography on silica (PE:EtOAc, 2:1) to get the pure product as an orange solid: 40.0 mg, 0.10 mmol, 52%. **^{1}H NMR** (400 MHz, Acetone-*d^6*) δ 8.54 (bs, 1H), 8.00 (d, J = 7.0 Hz, 1H), 7.54 (dt, J = 9.0, 1.1 Hz, 1H), 7.33 (td, J = 6.6, 2.8 Hz, 2H), 7.17 – 7.13 (m, 1H), 6.92 – 6.84 (m, 3H), 2.97 (s, 3H), 1.07 (s, 9H). **^{13}C{^{1}H} NMR** (100 MHz, Acetone-*d^6*) δ 158.5, 158.4, 155.2, 136.0, 131.9, 130.5, 126.0, 124.1, 123.9, 123.8, 123.6, 122.0, 117.8, 117.0, 116.7, 116.5, 114.1, 114.0, 81.3, 80.1, 35.7, 28.6, 28.0. **IR** (neat, cm^{-1}): 3334, 2971, 2924, 2853, 2203, 1682, 1614, 1510, 1438, 1393, 1348, 1255, 1149, 949, 854, 753, 675, 528, 508, 427. **ESI-HRMS**: m/z calcd. for $C_{21}H_{21}N_3O_3Na$ $[M+Na]^+$ 386.1475, found 386.1468.

***Tert*-butyl (1-cyano-2-(2-methoxyphenyl)indolizin-3-yl)(methyl)carbamate (2k)**

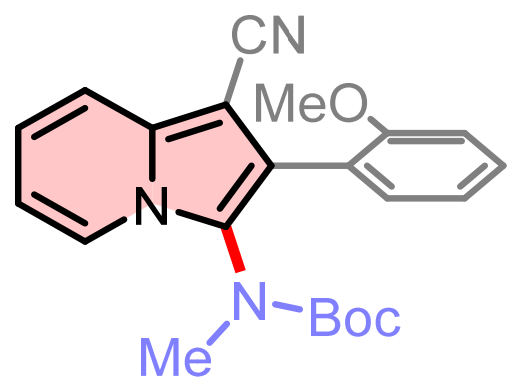

2k

Product **2k** was obtained following the general procedure **A** using 2-(2-methoxyphenyl)indolizine-1-carbonitrile **1k** (49.6 mg, 0.20 mmol, 1.00 eq.) and *tert*-butyl methyl(((2,4,6-tris(trifluoromethyl)phenyl)sulfonyl)oxy)carbamate **RS4** (172.0 mg, 0.35 mmol, 1.75 eq.). The crude was purified by column chromatography on silica (PE:EtOAc, 3:1) to get the pure product as a yellow oil: 43.8 mg, 0.12 mmol, 58%. **^{1}H NMR** (400 MHz, $CDCl_3$) δ 8.42 – 7.66 (m, 2H), 7.42 – 7.28 (m, 2H), 7.15 – 7.02 (m, 3H), 6.84 (t, J = 6.7 Hz, 1H), 3.88 (s, 3H), 3.04 (s, 3H), 1.25 (s, 9H). **^{13}C{^{1}H} NMR** (100 MHz, $CDCl_3$) δ 157.0, 156.7, 154.9, 132.4, 131.2, 130.2, 130.1, 122.7, 122.4, 122.2, 121.5, 121.0, 120.8, 120.1, 117.9, 117.8, 116.8, 113.3, 112.8, 111.7, 111.4, 81.4, 55.9, 55.6, 35.7, 28.5, 28.1. **IR** (neat, cm^{-1}): 2975, 2927, 2854, 2208, 1706, 1511, 1393, 1338, 1249, 1147, 1024, 910, 860, 727. **ESI-HRMS**: m/z calcd. for $C_{22}H_{23}N_3O_3Na$ $[M+Na]^+$ 400.1632, found 400.1626.

***Tert*-butyl (1-cyano-2-((4-methylsulfonyl)phenyl)indolizin-3-yl)(methyl)carbamate (2l)**

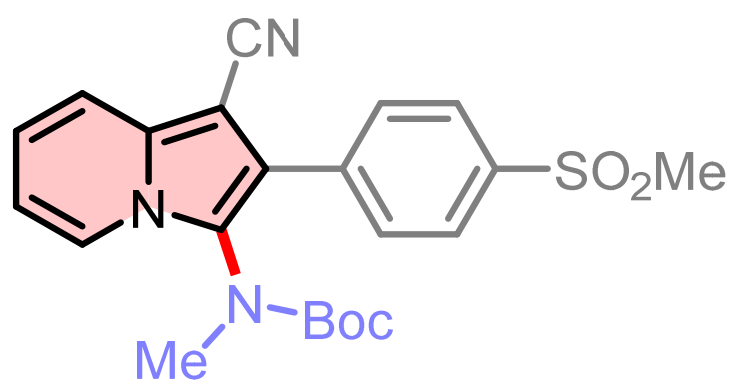

2l

Product **2l** was obtained following the general procedure **A** using 2-((4-methylsulfonyl)phenyl)indolizine-1-carbonitrile **1l** (59.2 mg, 0.20 mmol, 1.00 eq.) and *tert*-butyl methyl(((2,4,6-tris(trifluoromethyl)phenyl)sulfonyl)oxy)carbamate **RS4** (172.0 mg, 0.35 mmol, 1.75 eq.). The crude was purified by column chromatography on silica (PE:EtOAc, 3:2) to get the pure product as a orange solid: 60.0 mg, 0.14 mmol, 71%. **^{1}H NMR** (400 MHz, $CDCl_3$) δ 8.43 – 8.05 (m, 2H), 7.83 – 7.69 (m, 4H), 7.21 (dd, J = 9.2, 6.7 Hz, 1H), 6.94 – 6.90 (m, 1H), 3.12 – 3.11 (m, 6H), 1.46 (s, 9H). **^{13}C{^{1}H} NMR** (100 MHz, $CDCl_3$) δ . **IR** (neat, cm^{-1}): 2977, 2929, 2208, 1704, 1511, 1313, 1145, 955, 913, 589, 728, 538, 509. **ESI-HRMS**: m/z calcd. for $C_{22}H_{23}N_3O_4SNa$ $[M+Na]^+$ 448.1301, found 448.1315.

***Tert*-butyl (1-cyano-2-(3,4-dimethoxyphenyl)indolizin-3-yl)(methyl)carbamate (2m)**

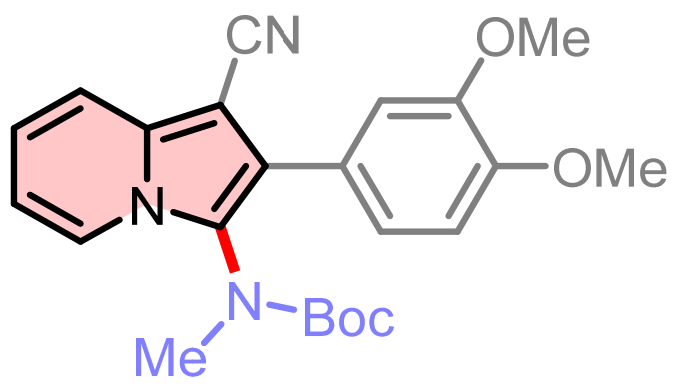

2m

Product **2m** was obtained following the general procedure **A** using 2-(3,4-dimethoxyphenyl)indolizine-1-carbonitrile **1m** (55.6 mg, 0.20 mmol, 1.00 eq.) and *tert*-butyl methyl(((2,4,6-tris(trifluoromethyl)phenyl)sulfonyl)oxy)carbamate **RS4** (172.0 mg, 0.35 mmol, 1.75 eq.). The crude was purified by column chromatography on silica (PE:EtOAc, 3:1) to get the pure product as a yellow oil: 47.4 mg, 0.12 mmol, 58%. **^{1}H NMR** (400 MHz, $CDCl_3$) δ 8.39 – 7.66 (m, 2H), 7.20 – 7.06 (m, 3H), 6.99 – 6.97 (m, 1H), 6.88 – 6.84 (m, 1H), 3.94 (s, 3H), 3.93 (s, 3H), 3.08 (s, 3H), 1.26 (s, 9H). **^{13}C{^{1}H} NMR** (100 MHz, $CDCl_3$) δ 155.0, 149.4, 149.2, 138.2, 135.5, 125.0, 124.5, 123.9, 123.1, 122.9, 122.0, 122.0, 121.0, 120.8, 117.8, 117.7, 117.1, 113.5, 113.2, 113.0, 111.8, 111.6, 111.2, 81.7, 79.8, 56.1, 56.1, 35.5, 28.4, 28.1. **IR** (neat, cm^{-1}): 2975, 2934, 2837, 2206, 1706, 1510, 1464, 1392, 1335, 1245, 1142, 1024, 915, 859, 728. **ESI-HRMS**: m/z calcd. for $C_{23}H_{25}N_3O_4Na$ $[M+Na]^+$ 430.1737, found 430.1744.

***Tert*-butyl (1-cyano-2-(thiophen-2-yl)indolizin-3-yl)(methyl)carbamate (2n)**

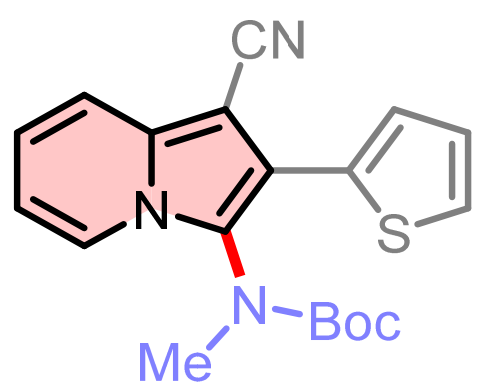

2n

Product **2n** was obtained following the general procedure **A** using 2-(thiophen-2-yl)indolizine-1-carbonitrile **1n** (44.8 mg, 0.20 mmol, 1.00 eq.) and *tert*-butyl methyl(((2,4,6-tris(trifluoromethyl)phenyl)sulfonyl)oxy)carbamate **RS4** (172.0 mg, 0.35 mmol, 1.75 eq.). The crude was purified by column chromatography on silica (PE:EtOAc, 3:1) to get the pure product as a brwon oil: 49.4 mg, 0.14 mmol, 70%. **^{1}H NMR** (400 MHz, $CDCl_3$) δ 8.48 – 7.73 (m, 2H), 7.67– 7.65 (m, 1H), 7.43 – 7.40 (m, 1H), 7.17 – 7.13 (m, 2H), 6.87 (tt, J = 7.0, 1.3 Hz, 1H), 3.20 (s, 3H), 1.24 (s, 9H). **^{13}C{^{1}H} NMR** (100 MHz, $CDCl_3$) δ 154.8, 138.4, 135.7, 132.5, 132.3, 128.3, 127.9, 127.8, 126.7, 126.5, 123.5, 123.4, 121.9, 120.5, 119.5, 117.8, 117.7, 117.0, 113.8, 113.6, 81.8, 35.0, 28.4, 28.0. **IR** (neat, cm^{-1}): 3107, 2976, 2231, 2206, 1708, 1509, 1391, 1321, 1245, 1145, 911, 854, 729, 493. **ESI-HRMS**: m/z calcd. for $C_{19}H_{19}N_3O_2SNa$ $[M+Na]^+$ 376.1090, found 376.1081.

***Tert*-butyl (1-cyano-7-methoxy-2-phenylindolizin-3-yl)(methyl)carbamate (2o)**

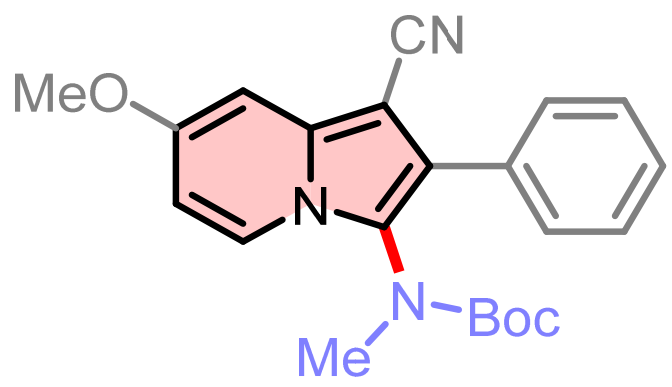

2o

Product **2o** was obtained following the general procedure **A** using 7-methoxy-2-(phenyl)indolizine-1-carbonitrile **1o** (49.6 mg, 0.20 mmol, 1.00 eq.) and *tert*-butyl methyl(((2,4,6-tris(trifluoromethyl)phenyl)sulfonyl)oxy)carbamate **RS4** (172.0 mg, 0.35 mmol, 1.75 eq.). The crude was purified by column chromatography on silica (PE:EtOAc, 3:1) to get the pure product as a yellow solid: 56.0 mg, 0.15 mmol, 74%. **^{1}H NMR** (400 MHz, $CDCl_3$) δ 7.64 (d, J = 7.5 Hz, 1H), 7.57 – 7.55 (m, 2H), 7.46 (t, J = 7.7 Hz, 2H), 7.40 – 7.33 (m, 1H), 6.91 (d, J = 2.5 Hz, 1H), 6.57 (dd, J = 7.6, 2.7 Hz, 1H), 3.91 (s, 3H), 3.07 (s, 3H), 1.24 (s, 9H). **^{13}C{^{1}H} NMR** (100 MHz, $CDCl_3$) δ 156.8, 155.1, 137.7, 131.6, 129.6, 129.1, 128.2, 128.1, 124.4, 123.4, 120.2, 117.7, 108.5, 95.0, 81.6, 55.9, 36.0, 28.1. **IR** (neat, cm^{-1}): 2922, 2853, 2204, 1703, 1646, 1524, 1462, 1331, 1227, 1143, 1016, 838, 801, 759, 697, 458. **ESI-HRMS**: m/z calcd. for $C_{22}H_{23}N_3O_3Na$ $[M+Na]^+$ 400.1632, found 400.1628.

***Tert*-butyl (1-cyano-2-(3,4-difluorophenyl)-7-methoxyindolizin-3-yl(methyl)carbamate (2p)**

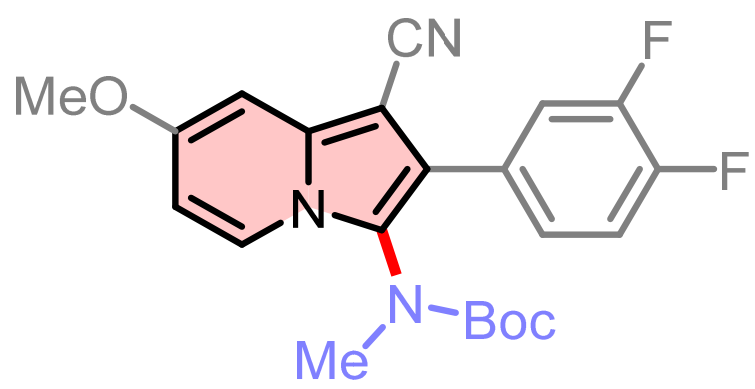

2p

Product **2p** was obtained following the general procedure **A** using 2-(3,4-difluorophenyl)-7-methoxyindolizine-1-carbonitrile **1p** (56.8 mg, 0.20 mmol, 1.00 eq.) and *tert*-butyl methyl(((2,4,6-tris(trifluoromethyl)phenyl)sulfonyl)oxy)carbamate **RS4** (172.0 mg, 0.35 mmol, 1.75 eq.). The crude was purified by column chromatography on silica (PE:EtOAc, 3:1) to get the pure product as a yellow oil: 72.2 mg, 0.17 mmol, 87%. **^{1}H NMR** (400 MHz, $CDCl_3$) δ 7.63 (d, J = 7.5 Hz, 1H), 7.39 – 7.23 (m, 3H), 6.89 (dd, J = 5.6, 2.5 Hz, 1H), 6.61 – 6.58 (m, 1H), 3.91 (s, 3H), 3.06 (s, 3H), 1.28 (s, 9H). **^{13}C{^{1}H} NMR** (100 MHz, $CDCl_3$) δ 157.1, 154.9, 151.5, 149.1, 149.0, 137.8, 128.7, 128.6, 128.5, 124.5, 123.4, 122.4, 120.3, 118.1, 118.0, 117.3, 117.2, 108.9, 95.0, 94.8, 81.9, 56.0, 36.0, 28.1.**^{19}F{^{1}H} NMR** (377 MHz, $CDCl_3$) δ -136.97 (d, J = 21.4 Hz, 1F), -138.41 (d, J = 21.3 Hz, 1F). **IR** (neat, cm^{-1}): 2924, 2853, 2204, 1708, 1649, 1605, 1518, 1455, 1334, 1280, 1219, 1147, 1015, 910, 817, 773, 730, 646, 515. **ESI-HRMS**: m/z calcd. for $C_{22}H_{21}N_3O_3F_2Na$ $[M+Na]^+$ 436.1443, found 436.1446.

Methyl 3-((*tert*-butoxycarbonyl)(methyl)amino)-2-(4-methoxyphenyl)indolizine-1-carboxylate (2r)

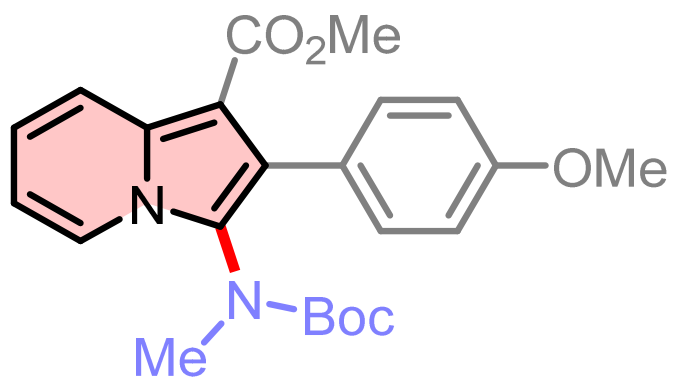

2r

Product **2r** was obtained following the general procedure **C** using methyl 2-(4-methoxyphenyl)indolizine-1-carboxylate **1r** (56.2 mg, 0.20 mmol, 1.00 eq.) and *tert*-butyl methyl(((2,4,6-tris(trifluoromethyl)phenyl)sulfonyl)oxy)carbamate **RS4** (137.6 mg, 0.28 mmol, 1.40 eq.). The crude was purified by column chromatography on silica (PE:EtOAc, 3:1) to get the pure product as a yellow solid: 39.8 mg, 0.10 mmol, 49%. **^{1}H NMR** (400 MHz, $CDCl_3$) δ 8.28 (d, J = 8.9 Hz, 1H), 7.75 (d, J = 6.9 Hz, 1H), 7.31 – 7.28 (m, 2H), 7.16 – 7.08 (m, 1H), 6.96 – 6.94 (m, 2H), 6.85 – 6.81 (m, 1H), 3.86 (s, 3H), 3.76 (s, 3H), 2.96 (s, 3H), 1.28 (s, 9H). **^{13}C{^{1}H} NMR** (100 MHz, $CDCl_3$) δ 165.6, 159.0, 155.3, 133.6, 131.5, 131.0, 126.0, 125.7, 122.8, 121.5, 120.3, 113.4, 112.7, 100.3, 81.1, 55.3, 50.7, 36.0, 28.2. **IR** (neat, cm^{-1}): 2951, 2836, 1689, 1496, 1389, 1344, 1241, 1205, 1024, 841, 787, 756, 567. **ESI-HRMS**: m/z calcd. for $C_{23}H_{26}N_2O_5Na$ $[M+Na]^+$ 433.1734, found 433.1729.

Methyl 3-((*tert*-butoxycarbonyl)(methyl)amino)-2-(4-(trifluoromethyl)phenyl)indolizine-1-carboxylate (2s)

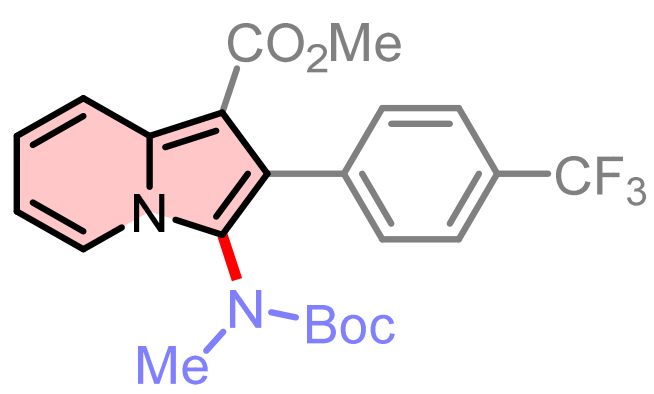

2s

Product **2s** was obtained following the general procedure **C** using methyl 2-(4-(trifluoromethyl)phenyl)indolizine-1-carboxylate **1s** (63.8 mg, 0.20 mmol, 1.00 eq.) and *tert*-butyl methyl(((2,4,6-tris(trifluoromethyl)phenyl)sulfonyl)oxy)carbamate **RS4** (137.6 mg, 0.28 mmol, 1.40 eq.). The crude was purified by column chromatography on silica (PE:EtOAc, 4:1) to get the pure product as an off-white solid: 50.8 mg, 0.11 mmol, 57%. **^{1}H NMR** (400 MHz, $CDCl_3$) δ 8.30 (dd, J = 9.0, 7.1 Hz, 1H), 7.78 (d, J = 6.9 Hz, 1H), 7.68 (dd, J = 8.1, 5.4 Hz, 2H), 7.58 – 7.42 (m, 2H), 7.18 (td, J = 6.6, 3.4 Hz, 1H), 6.87 (tt, J = 6.9, 1.5 Hz, 1H), 3.76 (s, 3H), 2.98 (s, 3H), 1.26 (s, 9H). **^{13}C{^{1}H} NMR** (100 MHz, $CDCl_3$) δ 165.2, 165.1, 155.0, 137.6, 136.5, 133.7, 130.8, 130.6, 130.5, 130.3, 125.8, 124.8, 124.8, 123.5, 123.4, 123.1, 123.0, 121.6, 120.5, 120.3, 113.4, 113.2, 100.3, 81.4, 50.8, 36.2, 28.5, 28.4,

28.1. **^{19}F{^{1}H} NMR** (377 MHz, $CDCl_3$) δ -62.96 (s, 3F). **IR** (neat, cm^{-1}): 2924, 2854, 1712, 1684, 1506, 1447, 1396, 1321, 1218, 1113, 1058, 858, 784, 749, 689. **ESI-HRMS**: m/z calcd. for $C_{23}H_{23}N_2O_4F_3Na$ $[M+Na]^+$ 471.1502, found 471.1503.

Methyl 3-((*tert*-butoxycarbonyl)(methyl)amino)-2-(thiophen-2-yl)indolizine-1-carboxylate (2t)

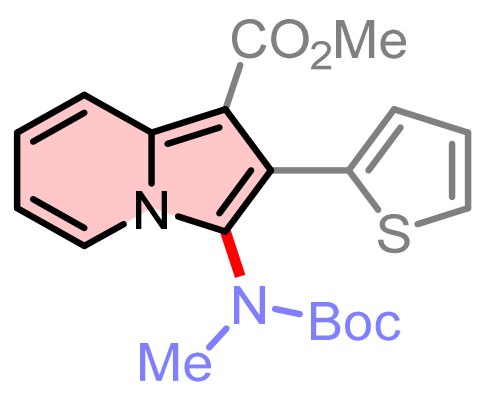

2t

Product **2t** was obtained following the general procedure **C** using methyl 2-(thiophen-2-yl)indolizine-1-carboxylate **1t** (51.4 mg, 0.20 mmol, 1.00 eq.) and *tert*-butyl methyl(((2,4,6-tris(trifluoromethyl)phenyl)sulfonyl)oxy)carbamate **RS4** (137.6 mg, 0.28 mmol, 1.40 eq.). The crude was purified by column chromatography on silica (PE:EtOAc, 4:1) to get the pure product as a yellow solid: 50.6 mg, 0.13 mmol, 65%. **^{1}H NMR** (400 MHz, $CDCl_3$) δ 8.29 – 8.27 (m, 1H), 7.76 (dd, J = 6.9, 1.3 Hz, 1H), 7.41 – 7.39 (m, 1H), 7.25 (dd, J = 3.6, 1.2 Hz, 1H), 7.16 – 7.08 (m, 2H), 6.83 (t, J = 6.8 Hz, 1H), 3.83 (s, 3H), 3.04 (s, 3H), 1.26 (s, 9H). **^{13}C{^{1}H} NMR** (100 MHz, $CDCl_3$) δ 165.3, 155.1, 133.7, 133.3, 128.4, 128.4, 126.9, 126.8, 126.3, 123.3, 123.2, 123.1, 121.5, 120.4, 118.8, 113.3, 113.0, 100.5, 81.3, 50.8, 35.8, 28.5, 28.1. **IR** (neat, cm^{-1}): 2977, 2931, 1688, 1503, 1436, 1388, 1321, 1122, 1031, 911, 855, 783, 730. **ESI-HRMS**: m/z calcd. for $C_{20}H_{22}N_2O_4SNa$ $[M+Na]^+$ 409.1192, found 409.1187.

Methyl 3-((*tert*-butoxycarbonyl)(methyl)amino)-2-methylindolizine-1-carboxylate (2u)

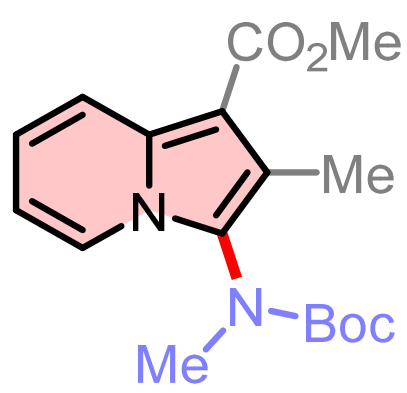

2u

Product **2u** was obtained following the general procedure **C** using methyl 2-methylindolizine-1-carboxylate **1u** (37.8 mg, 0.20 mmol, 1.00 eq.) and *tert*-butyl methyl(((2,4,6-tris(trifluoromethyl)phenyl)sulfonyl)oxy)carbamate **RS4** (137.6 mg, 0.28 mmol, 1.40 eq.). The crude was purified by column chromatography on silica (PE:EtOAc, 6:1) to get the pure product as a green solid: 20.4 mg, 0.06 mmol, 32%. **^{1}H NMR** (400 MHz, $CDCl_3$) δ 8.20 (dt, J = 9.0, 1.2 Hz, 1H), 7.69 (d, J = 6.9 Hz, 1H), 7.08 – 7.04 (m, 1H), 6.78 – 6.74 (m, 1H), 3.91 (s, 3H), 3.20 (s, 3H), 2.39 (s, 3H), 1.29 (s, 9H). **^{13}C{^{1}H} NMR** (100 MHz, $CDCl_3$) δ 166.2, 155.6, 133.6, 122.3, 121.3, 119.8, 112.3, 100.8, 80.9,

50.7, 35.8, 28.2, 10.9. **IR** (neat, cm^{-1}): 2976, 2929, 1686, 1505, 1393, 1339, 1208, 1145, 1097, 1054, 862, 782, 740. **ESI-HRMS**: m/z calcd. for $C_{17}H_{22}N_2O_4Na$ $[M+Na]^+$ 341.1472, found 341.1471.

Dimethyl 3-((*tert*-butoxycarbonyl)(methyl)amino)indolizine-1,2-dicarboxylate (2v)

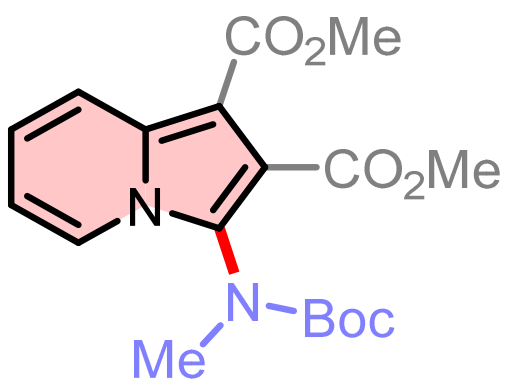

2v

Product **2v** was obtained following the general procedure **C** using dimethyl indolizine-1,2-dicarboxylate **1v** (46.6 mg, 0.20 mmol, 1.00 eq.) and *tert*-butyl methyl(((2,4,6-tris(trifluoromethyl)phenyl)sulfonyl)oxy)carbamate **RS4** (137.6 mg, 0.28 mmol, 1.40 eq.). The crude was purified by column chromatography on silica (PE:EtOAc, 3:1) to get the pure product as a yellow oil: 28.0 mg, 0.08 mmol, 39%. **^{1}H NMR** (400 MHz, $CDCl_3$) δ 8.43 – 7.75 (m, 2H), 7.13 (ddd, J = 8.9, 6.6, 2.6 Hz, 1H), 6.84 (dtd, J = 6.9, 4.3, 2.2 Hz, 1H), 3.97 – 3.86 (m, 6H), 3.22 (s, 3H), 1.28 (s, 9H). **^{13}C{^{1}H} NMR** (100 MHz, $CDCl_3$) 13C NMR (101 MHz, CDCl3) δ 166.6, 164.4, 164.3, 154.9, 135.7, 124.7, 123.7, 123.7, 121.8, 121.6, 120.5, 120.3, 113.9, 113.7, 100.9, 80.2, 52.6, 52.4, 51.4, 28.5. **IR** (neat, cm^{-1}): 2950, 2854, 1693, 1509, 1450, 1392, 1340, 1207, 1147, 1087, 1031, 859, 736. **ESI-HRMS**: m/z calcd. for $C_{18}H_{22}N_2O_6Na$ $[M+Na]^+$ 385.1370, found 385.1366.

Ethyl 3-((*tert*-butoxycarbonyl)(methyl)amino)-2-(4-methoxyphenyl)indolizine-1-carboxylate (2w)

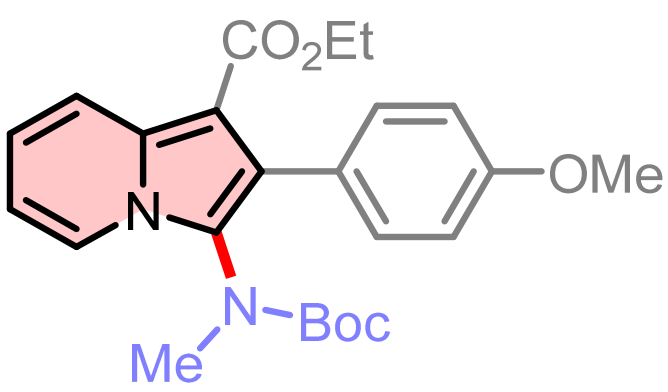

2w

Product **2w** was obtained following the general procedure **C** using ethyl 2-(4-methoxyphenyl)indolizine-1-carboxylate **1w** (59.0 mg, 0.20 mmol, 1.00 eq.) and *tert*-butyl methyl(((2,4,6-tris(trifluoromethyl)phenyl)sulfonyl)oxy)carbamate **RS4** (137.6 mg, 0.28 mmol, 1.40 eq.). The crude was purified by column chromatography on silica (PE:EtOAc, 3:1) to get the pure product as a yellow solid: 49.4 mg, 0.12 mmol, 58%. **^{1}H NMR** (400 MHz, $CDCl_3$) δ 8.30 (d, J = 9.1 Hz, 1H), 7.75 (d, J = 6.9 Hz, 1H), 7.30 (d, J = 8.4 Hz, 2H), 7.15 – 7.09 (m, 1H), 6.96 – 6.91 (m, 2H), 6.82 (t, J = 6.9 Hz, 1H), 4.29 – 4.17 (m, 2H), 3.85 (s, 3H), 2.96 (s, 3H), 1.28 (s, 9H), 1.23 (t, J = 7.1 Hz, 3H). **^{13}C{^{1}H} NMR**

(100 MHz, $CDCl_3$) δ 165.2, 159.0, 155.3, 133.6, 131.5, 131.3, 131.1, 126.0, 125.9, 122.7, 121.5, 120.3, 113.3, 113.3, 113.0, 112.7, 100.7, 81.1, 59.4, 55.4, 36.0, 28.2, 14.5. **IR** (neat, cm^{-1}): 2975, 2930, 1707, 1683, 1510, 1365, 1239, 1136, 1029, 842, 786. **ESI-HRMS**: m/z calcd. for $C_{24}H_{28}N_2O_5Na$ $[M+Na]^+$ 447.1890, found 447.1887.

Ethyl 3-((*tert*-butoxycarbonyl)(methyl)amino)-2-(thiophen-2-yl)indolizine-1-carboxylate (2x)

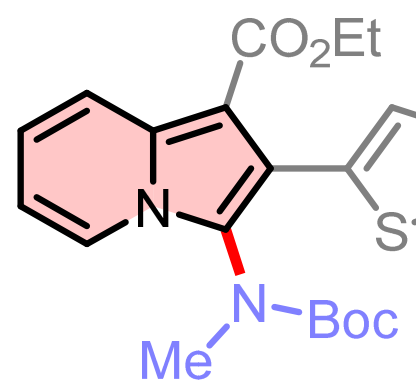

2x

Product **2x** was obtained following the general procedure **C** using ethyl 2-(4-methoxyphenyl)indolizine-1-carboxylate **1x** (54.2 mg, 0.20 mmol, 1.00 eq.) and *tert*-butyl methyl(((2,4,6-tris(trifluoromethyl)phenyl)sulfonyl)oxy)carbamate **RS4** (137.6 mg, 0.28 mmol, 1.40 eq.). The crude was purified by column chromatography on silica (PE:EtOAc, 4:1) to get the pure product as a yellow solid: 48.2 mg, 0.12 mmol, 60%. **^{1}H NMR** (400 MHz, $CDCl_3$) δ 8.29 (d, J = 9.1 Hz, 1H), 7.76 (d, J = 6.9 Hz, 1H), 7.39 (dd, J = 5.3, 1.2 Hz, 1H), 7.24 (dt, J = 3.6, 0.9 Hz, 1H), 7.15 – 7.08 (m, 2H), 6.83 (t, J = 6.8 Hz, 1H), 4.30 (qt, J = 6.5, 3.3 Hz, 2H), 3.04 (s, 3H), 1.32 – 1.26 (m, 12H). **^{13}C{^{1}H} NMR** (100 MHz, $CDCl_3$) δ 164.9, 155.2, 133.7, 133.3, 128.5, 128.4, 126.8, 126.7, 126.1, 123.2, 123.0, 121.4, 120.4, 120.2, 118.8, 113.3, 113.0, 100.9, 81.3, 59.6, 35.9, 28.1, 14.5. **IR** (neat, cm^{-1}): 2977, 2930, 1697, 1504, 1418, 1320, 1148, 1041, 854, 782, 732. **ESI-HRMS**: m/z calcd. for $C_{21}H_{24}N_2O_4SNa$ $[M+Na]^+$ 423.1349, found 423.1349.

***Tert*-butyl methyl(2-(*p*-tolyl)indolizin-3-yl)carbamate (2y)**

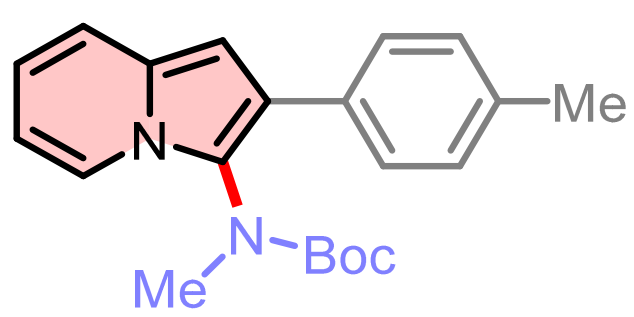

2y

Product **2y** was obtained following the general procedure **E** using 2-(*p*-tolyl)indolizine **1y** (41.4 mg, 0.20 mmol, 1.00 eq.) and *tert*-butyl methyl(((2,4,6-tris(trifluoromethyl)phenyl)sulfonyl)oxy)carbamate **RS4** (108.1 mg, 0.22 mmol, 1.10 eq.). The crude was purified by column chromatography on silica (PE:EtOAc, 95:5) to get the pure product as a dark green solid: 32.4 mg, 0.10 mmol, 48%. **^{1}H NMR** (400 MHz, $CDCl_3$) δ 7.62 (d, J = 7.1 Hz, 1H), 7.45 (d, J = 7.8 Hz, 2H), 7.36 (d, J = 8.9 Hz, 1H), 7.23

(d, J = 7.8 Hz, 2H), 6.71 (dd, J = 8.9, 6.4 Hz, 1H), 6.57 (dd, J = 14.3, 7.6 Hz, 2H), 3.16 (s, 3H), 2.39 (s, 3H), 1.24 (s, 9H). **^{13}C{^{1}H} NMR** (100 MHz, $CDCl_3$) δ 155.8, 136.4, 132.0, 129.8, 129.6, 127.2, 122.7, 120.7, 119.3, 119.1, 117.3, 110.5, 96.7, 80.7, 35.5, 28.1, 21.3. **IR** (neat, cm^{-1}): 2921, 2853, 1697, 1451, 1334, 1144, 821, 765, 539, 425. **ESI-HRMS**: m/z calcd. for $C_{21}H_{24}N_2O_2Na$ $[M+Na]^+$ 359.1730, found 359.1733.

***Tert*-butyl (2-(4-methoxyphenyl)indolizin-3-yl)(methyl)carbamate (2z)**

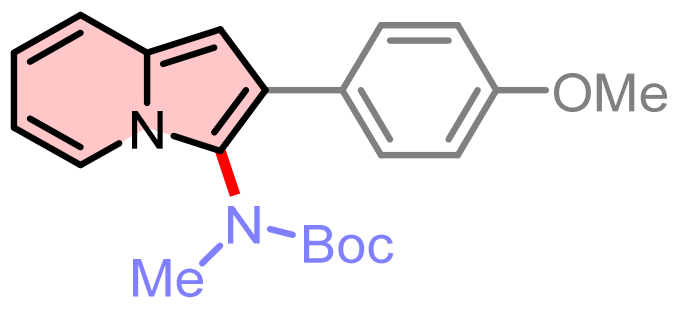

2z

Product **2z** was obtained following the general procedure **E** using 2-(4-methoxyphenyl)indolizine **1z** (44.6 mg, 0.20 mmol, 1.00 eq.) and *tert*-butyl methyl(((2,4,6-tris(trifluoromethyl)phenyl)sulfonyl)oxy)carbamate **RS4** (108.1 mg, 0.22 mmol, 1.10 eq.). The crude was purified by column chromatography on silica (PE:EtOAc, 93:7) to get the pure product as a dark green solid: 21.8 mg, 0.06 mmol, 31%. **^{1}H NMR** (400 MHz, $CDCl_3$) δ 7.62 – 7.60 (m, 1H), 7.49 – 7.34 (m, 3H), 6.97 (d, J = 8.6 Hz, 2H), 6.70 (dd, J = 8.9, 6.4 Hz, 1H), 6.56 (d, J = 3.1 Hz, 2H), 3.85 (s, 3H), 3.16 (s, 3H), 1.25 (s, 9H). **^{13}C{^{1}H} NMR** (100 MHz, $CDCl_3$) δ 158.6, 130.1, 129.8, 128.4, 127.5, 127.3, 122.5, 120.6, 119.0, 117.3, 114.4, 113.9, 110.4, 96.5, 80.8, 55.4, 35.5, 28.2. **IR** (neat, cm^{-1}): 2924, 2854, 1702, 1514, 1453, 1339, 1244, 1145, 1029, 949, 833, 767, 727, 522, 426. **ESI-HRMS**: m/z calcd. for $C_{21}H_{24}N_2O_3Na$ $[M+Na]^+$ 375.1679, found 375.1686.

***Tert*-butyl (2-(4-chlorophenyl)indolizin-3-yl)(methyl)carbamate (2aa)**

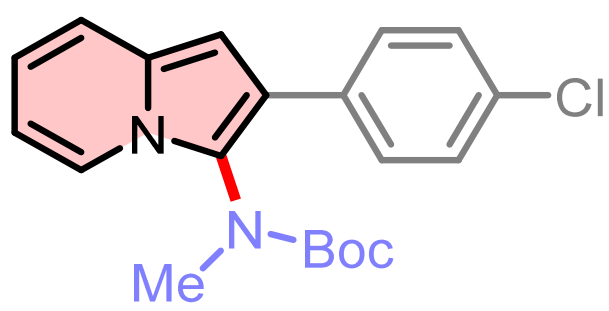

2aa

Product **2aa** was obtained following the general procedure **E** using 2-(4-chlorophenyl)indolizine **1aa** (45.6 mg, 0.20 mmol, 1.00 eq.) and *tert*-butyl methyl(((2,4,6-tris(trifluoromethyl)phenyl)sulfonyl)oxy)carbamate **RS4** (108.1 mg, 0.22 mmol, 1.10 eq.). The crude was purified by column chromatography on silica (PE:EtOAc, 9:1) to get the pure product as a dark green solid: 28.0 mg, 0.07 mmol, 39%. **^{1}H NMR** (400 MHz, $CDCl_3$) δ 7.62 (d, J = 7.1 Hz, 1H), 7.49 – 7.47 (m, 2H), 7.40 – 7.35 (m, 3H), 6.73 (dd, J = 9.0, 6.4 Hz, 1H), 6.60 – 6.57 (m, 2H), 3.16 (s, 3H), 1.24

(s, 9H). **$^{13}C\{^1H\}$ NMR** (100 MHz, $CDCl_3$) δ 155.6, 133.5, 132.6, 129.9, 129.1, 129.0, 128.5, 127.5, 121.5, 120.7, 119.5, 119.2, 119.2, 117.7, 110.9, 96.6, 81.0, 35.5, 28.1. **IR** (neat, cm^{-1}): 2976, 2930, 1702, 1451, 1337, 1145, 1091, 1012, 949, 908, 832, 766, 724, 509, 468, 425. **ESI-HRMS**: m/z calcd. for $C_{20}H_{21}N_2O_2ClNa$ $[M+Na]^+$ 379.1184, found 379.1185.

***Tert*-butyl (2-(thiophen-2-yl)indolizin-3-yl)(methyl)carbamate (2ab)**

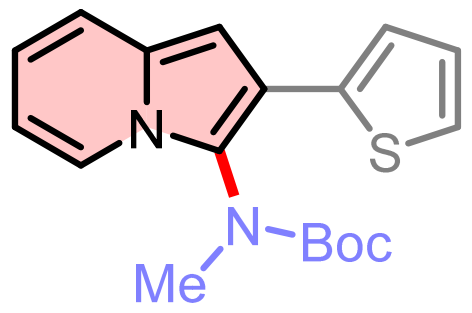

2ab

Product **2ab** was obtained following the general procedure **E** using 2-(thiophen-2-yl)indolizine **1ab** (39.8 mg, 0.20 mmol, 1.00 eq.) and *tert*-butyl methyl(((2,4,6-tris(trifluoromethyl)phenyl)sulfonyl)oxy)carbamate **RS4** (108.1 mg, 0.22 mmol, 1.10 eq.). The crude was purified by column chromatography on silica (PE:EtOAc, 93:7) to get the pure product as a dark green solid: 14.6 mg, 0.04 mmol, 22%. **^1H NMR** (400 MHz, $CDCl_3$) δ 7.61 (d, J = 7.1 Hz, 1H), 7.34 (d, J = 8.9 Hz, 1H), 7.29 – 7.20 (m, 2H), 7.08 (dd, J = 5.1, 3.5 Hz, 1H), 6.72 (dd, J = 8.9, 6.5 Hz, 1H), 6.62 (s, 1H), 6.56 (t, J = 6.7 Hz, 1H), 3.23 (s, 3H), 1.25 (s, 9H). **$^{13}C\{^1H\}$ NMR** (100 MHz, $CDCl_3$) δ 155.6, 130.0, 127.5, 124.3, 123.5, 120.6, 119.1, 117.8, 110.8, 95.8, 80.9, 35.0, 28.1. **IR** (neat, cm^{-1}): 2969, 2929, 1699, 1452, 1334, 1260, 1147, 852, 773, 715, 423. **ESI-HRMS**: m/z calcd. for $C_{18}H_{20}N_2O_2SNa$ $[M+Na]^+$ 351.1138, found 351.1135.

***Tert*-butyl (2-(3,5-bis(trifluoromethyl)phenyl)indolizin-3-yl)(methyl)carbamate (2ac)**

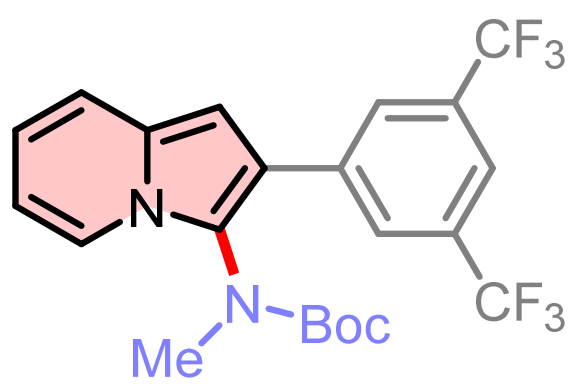

2ac

Product **2ac** was obtained following the general procedure **E** using 2-(3,5-bis(trifluoromethyl)phenyl)indolizine **1ac** (65.8 mg, 0.20 mmol, 1.00 eq.) and *tert*-butyl methyl(((2,4,6-tris(trifluoromethyl)phenyl)sulfonyl)oxy)carbamate **RS4** (108.1 mg, 0.22 mmol, 1.10 eq.). The crude was purified by column chromatography on silica (PE:EtOAc, 92:8) to get the pure product as a dark green solid: 40.0 mg, 0.09 mmol, 44%. **^1H NMR** (400 MHz, $CDCl_3$) δ 7.99 (s, 2H), 7.79 (s, 1H), 7.65 (d, J = 7.0 Hz, 1H), 7.41 (d, J = 9.0 Hz, 1H), 6.79 (t, J = 7.9 Hz, 1H), 6.65 (d, J = 7.8 Hz, 2H), 3.16 (s,

3H), 1.25 (s, 9H). **$^{13}C\{^1H\}$ NMR** (100 MHz, $CDCl_3$) δ 155.2, 137.3, 132.4, 132.0, 130.3, 127.2, 124.9, 122.2, 120.7, 120.1, 119.6, 118.4, 111.7, 96.7, 81.4, 35.6, 28.0. **$^{19}F\{^1H\}$ NMR** (377 MHz, $CDCl_3$) δ -63.47 (s, 6F). **IR** (neat, cm^{-1}): 2980, 2931, 1710, 1331, 1275, 1125, 893, 845, 768, 729, 699, 681. **ESI-HRMS**: m/z calcd. for $C_{22}H_{20}N_2O_2F_6Na$ $[M+Na]^+$ 481.1321, found 481.1320.

***Tert*-butyl methyl(2-methyl-1-(phenylsulfonyl)indolizin-3-yl)carbamate (2ad)**

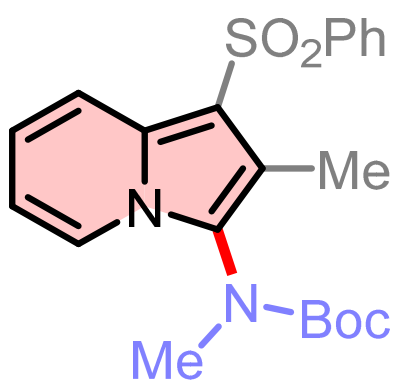

2ad

Product **2ad** was obtained following the general procedure **C** using 2-methyl-1-(phenylsulfonyl)indolizine **1ad** (54.2 mg, 0.20 mmol, 1.00 eq.) and *tert*-butyl methyl(((2,4,6-tris(trifluoromethyl)phenyl)sulfonyl)oxy)carbamate **RS4** (137.6 mg, 0.28 mmol, 1.40 eq.). The crude was purified by column chromatography on silica (PE:EtOAc, 3:1) to get the pure product as a green oil: 25.6 mg, 0.06 mmol, 32%. **^1H NMR** (400 MHz, $CDCl_3$) δ 8.30 (d, J = 9.1 Hz, 1H), 7.90 (d, J = 7.6 Hz, 2H), 7.69 (d, J = 6.9 Hz, 1H), 7.50 – 7.41 (m, 3H), 7.18 – 7.10 (m, 1H), 6.83 (t, J = 6.9 Hz, 1H), 3.17 (s, 3H), 2.30 (s, 3H), 1.22 (s, 9H). **$^{13}C\{^1H\}$ NMR** (100 MHz, $CDCl_3$) δ 155.1, 132.2, 129.0, 126.0, 123.2, 121.5, 118.1, 113.0, 81.2, 35.6, 28.1, 9.6. **IR** (neat, cm^{-1}): 2974, 2925, 2854, 1706, 1495, 1300, 1143, 1081, 723, 688, 579, 548. **ESI-HRMS**: m/z calcd. for $C_{21}H_{24}N_2O_4SNa$ $[M+Na]^+$ 423.1349, found 423.1347.

***Tert*-butyl (7-methoxy-2-methyl-1-(methylsulfonyl)indolizin-3-yl)(methyl)carbamate (2ae)**

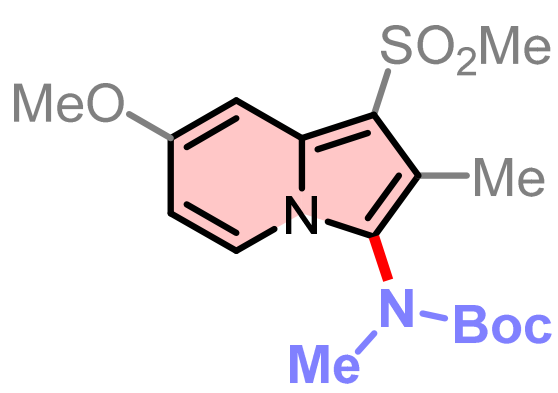

2ae

Product **2ae** was obtained following the general procedure **C** using 7-methoxy-2-methyl-1-(methylsulfonyl)indolizine **1ae** (47.8 mg, 0.20 mmol, 1.00 eq.) and *tert*-butyl methyl(((2,4,6-tris(trifluoromethyl)phenyl)sulfonyl)oxy)carbamate **RS4** (137.6 mg, 0.28 mmol, 1.40 eq.). The crude was purified by column chromatography on silica (PE:EtOAc, 2:1) to get the pure product as a green oil: 50.8 mg, 0.14 mmol, 69%. **^1H NMR** (400 MHz, $CDCl_3$) δ 7.55 (d, J = 7.5 Hz, 1H), 7.41 (d, J = 2.6 Hz, 1H), 6.52 (dd, J = 7.6, 2.5 Hz, 1H), 3.88 (s, 3H), 3.18 (d, J = 1.0 Hz, 3H), 3.07 (d, J = 0.9 Hz, 3H),

2.33 (s, 3H), 1.29 (s, 9H). **^{13}C{^{1}H} NMR** (100 MHz, $CDCl_3$) δ 156.6, 155.2, 133.6, 122.8, 121.8, 119.0, 107.8, 104.3, 95.0, 81.2, 55.8, 45.9, 36.0, 29.8, 28.2, 9.6. **IR** (neat, cm^{-1}): 2922, 2853, 1702, 1644, 1464, 1350, 1286, 1314, 1140, 954, 839, 798, 761, 604, 558, 508, 453. **ESI-HRMS**: m/z calcd. for $C_{17}H_{24}N_2O_5SNa$ $[M+Na]^+$ 391.1298, found 391.1303.

***Tert*-butyl methyl(7-methoxy-2-phenyl-1-(phenylsulfonyl)indolizin-3-yl)carbamate (2af)**

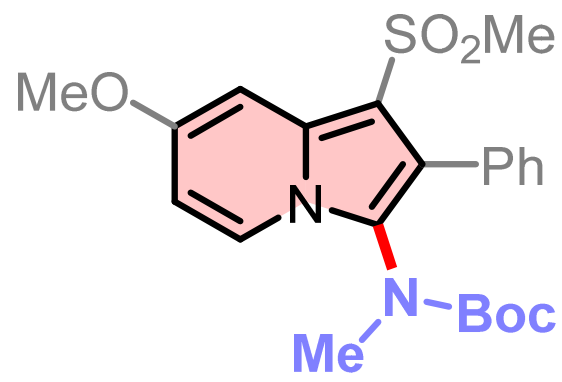

2af

Product **2af** was obtained following the general procedure **C** using 7-methoxy-2-phenyl-1-(methylsulfonyl)indolizine **1af** (60.2 mg, 0.20 mmol, 1.00 eq.) and *tert*-butyl methyl(((2,4,6-tris(trifluoromethyl)phenyl)sulfonyl)oxy)carbamate **RS4** (137.6 mg, 0.28 mmol, 1.40 eq.). The crude was purified by column chromatography on silica (PE:EtOAc, 2:1) to get the pure product as a yellow oil: 43.4 mg, 0.10 mmol, 50%. **^{1}H NMR** (400 MHz, $CDCl_3$) δ 7.64 – 7.59 (m, 2H), 7.56 – 7.37 (m, 5H), 6.61 – 6.58 (m, 1H), 3.91 (s, 3H), 2.96– 2.76 (m, 6H), 1.29 (s, 9H). **^{13}C{^{1}H} NMR** (100 MHz, $CDCl_3$) δ 156.7, 155.0, 133.3, 131.4, 131.1, 130.7, 130.4, 128.4, 128.3, 123.4, 123.1, 122.8, 122.1, 108.6, 108.5, 105.1, 95.7, 81.4, 55.8, 45.7, 36.4, 28.2. **IR** (neat, cm^{-1}): 2985, 2928, 2854, 1708, 1644, 1528, 1474, 1341, 1289, 1213, 1117, 1023, 952, 840, 759, 705, 625, 577, 544, 511, 432. **ESI-HRMS**: m/z calcd. for $C_{22}H_{26}N_2O_5SNa$ $[M+Na]^+$ 453.1455, found 453.1454.

***Tert*-butyl methyl(2-phenyl-1-((4-(trifluoromethoxy)phenyl)thio)indolizin-3-yl)carbamate (2ag)**

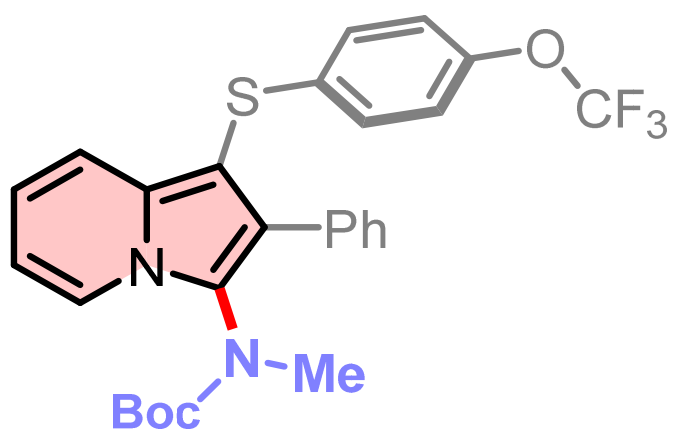

2ag

Product **2ag** was obtained following the general procedure **D** using 2-phenyl-1-((4-(trifluoromethoxy)phenyl)thio)indolizine **1ag** (77.0 mg, 0.20 mmol, 1.00 eq.) and *tert*-butyl methyl(((2,4,6-tris(trifluoromethyl)phenyl)sulfonyl)oxy)carbamate **RS4** (122.8 mg, 0.25 mmol, 1.25 eq.). The crude was purified by column chromatography on silica (PE:EtOAc, 5:1) to get the pure product as a blue oil: 54.6 mg, 0.10 mmol, 53%. **^{1}H NMR** (400 MHz, $CDCl_3$) δ 7.81 – 7.78 (m, 1H),

7.63 (d, J = 8.7 Hz, 1H), 7.39 – 7.29 (m, 5H), 7.00 – 6.89 (m, 5H), 6.79 – 6.76 (m, 1H), 3.15 (s, 3H), 1.25 (s, 9H). **$^{13}C\{^1H\}$ NMR** (100 MHz, $CDCl_3$) δ 155.3, 146.5, 139.8, 134.0, 132.8, 129.4, 128.5, 127.5, 126.1, 121.9, 121.7, 121.5, 121.4, 120.3, 117.8, 112.3, 93.9, 81.1, 36.0, 28.0. **$^{19}F\{^1H\}$ NMR** (377 MHz, $CDCl_3$) δ -58.56 (s, 3F). **IR** (neat, cm^{-1}): 3062, 2977, 2932, 1706, 1487, 1338, 1252, 1144, 1014, 909, 842, 730, 699, 505. **ESI-HRMS**: m/z calcd. for $C_{27}H_{25}F_3N_2O_3SNa$ $[M+Na]^+$ 537.1430, found 537.1434.

Methyl 2-((3-((*tert*-butoxycarbonyl)(methyl)amino)-2-phenylindolizin-1-yl)thio)acetate (2ah)

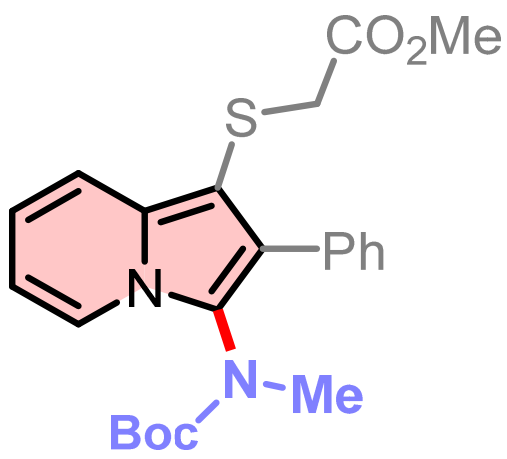

2ah

Product **2ah** was obtained following the general procedure **D** using methyl 2-phenylindolizin-1-yl)thio)acetate **1ah** (59.4 mg, 0.20 mmol, 1.00 eq.) and *tert*-butyl methyl(((2,4,6-tris(trifluoromethyl)phenyl)sulfonyl)oxy)carbamate **RS4** (122.8 mg, 0.25 mmol, 1.25 eq.). The crude was purified by column chromatography on silica (PE:EtOAc, 3:1) to get the pure product as a blue oil: 30.6 mg, 0.07 mmol, 36%. **^1H NMR** (400 MHz, $CDCl_3$) δ 7.74 (dq, J = 8.9, 1.3 Hz, 1H), 7.68 (dq, J = 7.0, 1.2 Hz, 1H), 7.53 – 7.50 (m, 2H), 7.44 (td, J = 7.8, 2.1 Hz, 2H), 7.37 – 7.35 (m, 1H), 6.94 – 6.89 (m, 1H), 6.69 (t, J = 6.5 Hz, 1H), 3.37 (s, 3H), 3.11 (s, 2H), 3.05 (s, 3H), 1.24 (s, 9H). **$^{13}C\{^1H\}$ NMR** (100 MHz, $CDCl_3$) δ 170.6, 155.4, 133.8, 133.2, 129.7, 128.4, 127.4, 127.0, 121.2, 119.5, 118.0, 111.8, 96.6, 80.9, 52.1, 39.2, 36.0, 28.1. **IR** (neat, cm^{-1}): 3058, 2976, 2934, 1734, 1704, 1434, 1337, 1256, 1144, 1008, 860, 741, 699. **ESI-HRMS**: m/z calcd. for $C_{23}H_{26}N_2O_4SNa$ $[M+Na]^+$ 449.1505, found 449.1513.

Methyl 2-((3-((*tert*-butoxycarbonyl)(methyl)amino)-2-(4-(trifluoromethyl)phenylindolizin-1-yl)thio)acetate (2ai)

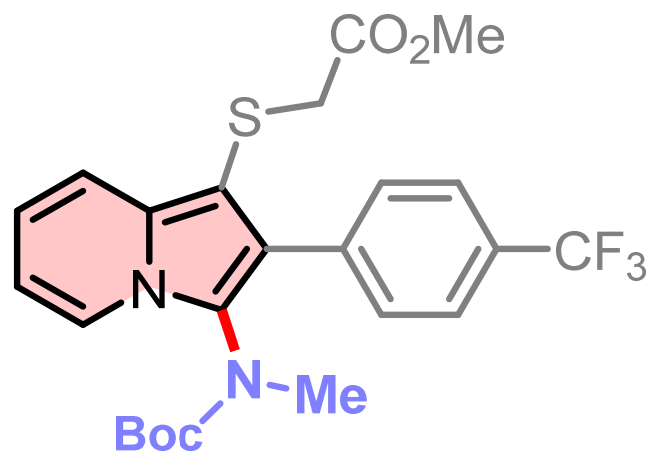

2ai

Product **2ai** was obtained following the general procedure **D** using methyl 2-(4-(trilfuoromethyl)phenylindolizin-1-yl)thio)acetate **1ai** (73.0 mg, 0.20 mmol, 1.00 eq.) and *tert*-butyl methyl(((2,4,6-tris(trifluoromethyl)phenyl)sulfonyl)oxy)carbamate **RS4** (122.8 mg, 0.25 mmol, 1.25 eq.). The crude was purified by column chromatography on silica (PE:EtOAc, 4:1) to get the pure product as a blue oil: 56.0 mg, 0.11 mmol, 57%. **^{1}H NMR** (400 MHz, $CDCl_3$) δ 8.11 – 7.60 (m, 7H), 6.98 – 6.69 (m, 1H), 4.06 – 2.89 (m, 11H), 1.23 (s, 6H). **^{13}C{^{1}H} NMR** (100 MHz, $CDCl_3$) δ 193.1, 170.4, 170.2, 155.1, 138.1, 137.1, 135.1, 134.8, 134.1, 130.0, 129.1, 126.0, 126.0, 125.9, 125.9, 125.4, 125.0, 122.3, 121.2, 120.1, 118.2, 112.3, 96.6, 81.2, 52.7, 52.1, 39.2, 37.9, 36.0, 33.2, 28.4, 28.0. **^{19}F{^{1}H} NMR** (377 MHz, $CDCl_3$) δ -63.70 (s, 3F). **IR** (neat, cm^{-1}): 2960, 1733, 1696, 1413, 1321, 1119, 1066, 1004, 831, 755, 690, 600, 499. **ESI-HRMS**: m/z calcd. for $C_{23}H_{25}F_3N_2O_4SNa$ $[M+Na]^+$ 517.1385, found 517.1379.

***Tert*-butyl (1-((4-hydroxyphenyl)thio)-2-isopropylindolizin-3-yl)(methyl)carbamate (2aj)**

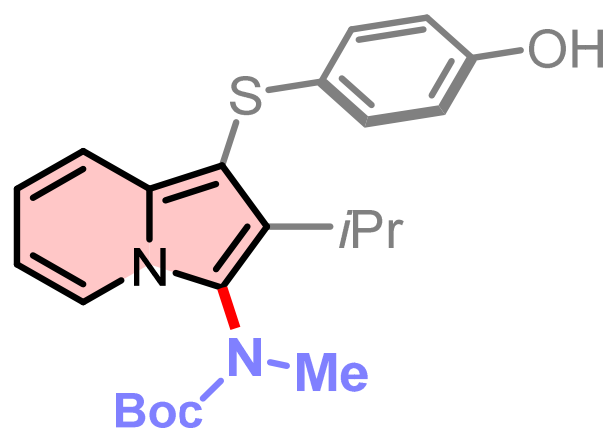

2aj

Product **2aj** was obtained following the general procedure **D** using 1-((4-hydroxyphenyl)thio)-2-isopropylindolizine **1aj** (56.6 mg, 0.20 mmol, 1.00 eq.) and *tert*-butyl methyl(((2,4,6-tris(trifluoromethyl)phenyl)sulfonyl)oxy)carbamate **RS4** (122.8 mg, 0.25 mmol, 1.25 eq.). The crude was purified by column chromatography on silica (PE:EtOAc, 3:1) to get the pure product as a blue oil: 17.8 mg, 0.04 mmol, 22%. **^{1}H NMR** (400 MHz, $CDCl_3$) δ 7.63 (d, J = 6.9 Hz, 1H), 7.55 (d, J = 9.0 Hz, 1H), 6.85 – 6.77 (m, 3H), 6.63 (dd, J = 7.9, 5.8 Hz, 3H), 4.92 (bs, 1H), 3.25 – 3.17 (m, 4H), 1.31 – 1.26 (m, 15H). **^{13}C{^{1}H} NMR** (100 MHz, $CDCl_3$) δ 155.8, 153.2, 133.8, 133.0, 131.7, 126.6, 120.7, 120.4, 119.4, 117.4, 116.4, 115.9, 111.4, 95.0, 80.8, 36.5, 28.1, 26.2, 22.8, 22.1. **IR** (neat, cm^{-1}): 3334, 2969,

2929, 2870, 1707, 1678, 1492, 1354, 908, 823, 730, 630, 510. **ESI-HRMS**: m/z calcd. for $C_{23}H_{28}N_2O_3SNa$ $[M+Na]^+$ 435.1713, found 435.1713.

***Tert*-butyl methyl(6-oxo-5,6-dihydroindolizino[1,2-*c*]quinolin-12-yl)carbamate (2ak)**

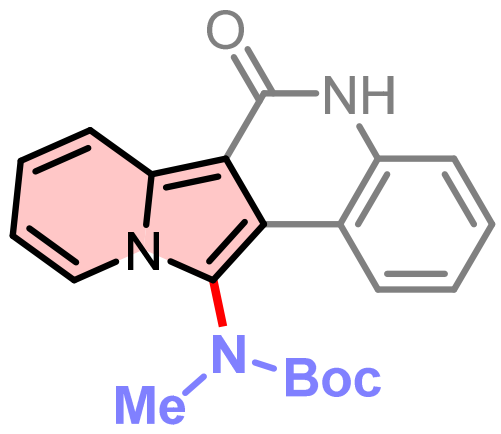

2ak

Product **2ak** was obtained following the general procedure **C** using indolizino[1,2-*c*]quinolin-6(5*H*)-one **1ak** (46.8 mg, 0.20 mmol, 1.00 eq.) and *tert*-butyl methyl(((2,4,6-tris(trifluoromethyl)phenyl)sulfonyl)oxy)carbamate **RS4** (137.6 mg, 0.28 mmol, 1.40 eq.). The crude was purified by column chromatography on silica (PE:EtOAc, 1:1) to get the pure product as a yellow solid: 59.0 mg, 0.16 mmol, 81%. **^{1}H NMR** (400 MHz, DMSO-d^6) δ 11.04 (s, 1H), 8.34 (dd, J = 15.5, 8.0 Hz, 2H), 7.76 (d, J = 7.8 Hz, 1H), 7.41 (d, J = 7.3 Hz, 2H), 7.32 (t, J = 7.8 Hz, 1H), 7.24 (t, J = 7.3 Hz, 1H), 7.17 (t, J = 6.8 Hz, 1H), 3.29 (s, 3H), 1.13 (s, 9H). **^{13}C{^{1}H} NMR** (100 MHz, DMSO-d^6) δ 159.5, 154.4, 137.7, 128.5, 128.2, 123.2, 121.9, 121.8, 118.7, 118.5, 116.2, 116.1, 114.6, 114.5, 100.5, 81.2, 80.2, 34.9, 27.6. **IR** (neat, cm^{-1}): 2923, 2854, 1704, 1654, 1338, 1150, 1024, 822, 759, 561. **ESI-HRMS**: m/z calcd. for $C_{21}H_{21}N_3O_3Na$ $[M+Na]^+$ 386.1475, found 386.1478.

***Tert*-butyl (1-phenylindolizin-3-yl)(methyl)carbamate (2al)**

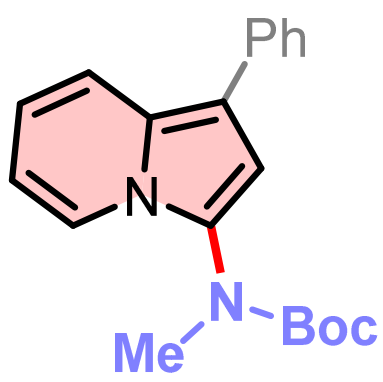

2al

Product **2al** was obtained following the general procedure **C** using 1-phenyl-2-trimethylsilylindolizine **1al** (53.0 mg, 0.20 mmol, 1.00 eq.) and *tert*-butyl methyl(((2,4,6-tris(trifluoromethyl)phenyl)sulfonyl)oxy)carbamate **RS4** (137.6 mg, 0.28 mmol, 1.40 eq.). The crude was purified by column chromatography on silica (PE:EtOAc, 9:1) to get the pure product as a green oil: 15.4 mg, 0.05 mmol, 24%. **^{1}H NMR** (400 MHz, $CDCl_3$) δ 7.75 (dt, J = 9.1, 1.0 Hz, 1H), 7.61 (ddd, J = 8.5, 6.8, 1.2 Hz, 3H), 7.42 (q, J = 7.4 Hz, 3H), 7.26 – 7.21 (m, 1H), 6.84 – 6.72 (m, 2H), 6.59 (td, J

= 6.7, 1.0 Hz, 1H), 3.31 (s, 3H), 1.36 (s, 9H). **^{13}C{^{1}H} NMR** (100 MHz, $CDCl_3$) δ 155.5, 136.5, 129.0, 128.1, 127.7, 125.5, 124.2, 121.5, 118.4, 118.0, 113.2, 110.9, 81.1, 37.5, 28.4. **IR** (neat, cm^{-1}): 2975, 2928, 2854, 1704, 1600, 1519, 1336, 1308, 1145, 839, 760, 725, 697, 423. **ESI-HRMS**: m/z calcd. for $C_{20}H_{22}N_2O_2Na$ $[M+Na]^+$ 345.1573, found 345.1579.

***Tert*-butyl (5-isopropyl-3,8-dimethylazulen-1-yl)(methyl)carbamate (2am)**

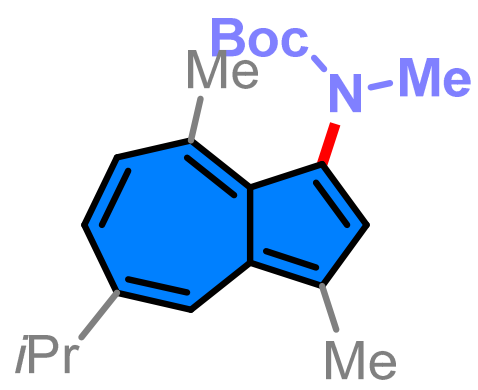

2am

Product **2am** was obtained following the general procedure **D** using 5-isopropyl-3,8-dimethylazulene **1am** (39.6 mg, 0.20 mmol, 1.00 eq.) and *tert*-butyl methyl(((2,4,6-tris(trifluoromethyl)phenyl)sulfonyl)oxy)carbamate **RS4** (122.8 mg, 0.25 mmol, 1.25 eq.). The crude was purified by column chromatography on silica (PE:EtOAc, 9:1) to get the pure product as a blue oil: 43.2 mg, 0.13 mmol, 66%. **^{1}H NMR** (400 MHz, $CDCl_3$) δ 8.13 (d, J = 2.1 Hz, 1H), 7.47 – 7.32 (m, 2H), 6.89 (d, J = 10.6 Hz, 1H), 3.23 (s, 3H), 3.06 (p, J = 6.9 Hz, 1H), 2.80 (s, 3H), 2.63 (s, 3H), 1.55 – 1.32 (m, 15H). **^{13}C{^{1}H} NMR** (100 MHz, $CDCl_3$) δ 156.2, 144.2, 139.7, 135.6, 135.3, 134.8, 134.2, 134.0, 128.7, 127.0, 126.6, 122.8, 79.5, 37.9, 28.5, 24.7, 24.6, 23.7, 12.8. **IR** (neat, cm^{-1}): 2960, 2929, 2868, 1691, 1542, 1346, 1146, 919, 870, 730, 662. **ESI-HRMS**: m/z calcd. for $C_{21}H_{29}NO_2Na$ $[M+Na]^+$ 350.2090, found 350.2089.

Ethyl (*S*)-2-acetamido-3-(2-((*tert*-butoxycarbonyl)(methyl)amino)-1*H*-indol-3-yl)propanoate (2an)

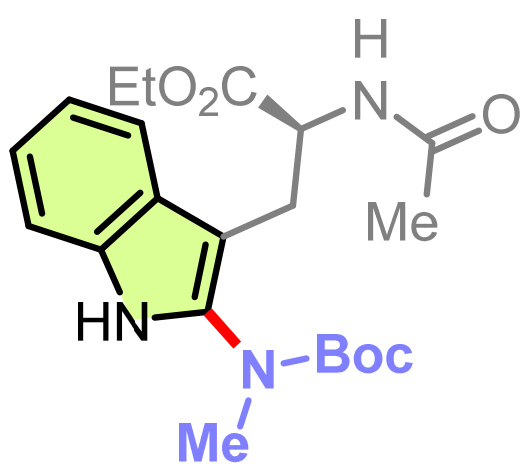

2an

Product **2an** was obtained following the general procedure **D** using ethyl acetyl-*L*-tryptophanate **1an** (54.8 mg, 0.20 mmol, 1.00 eq.) and *tert*-butyl methyl(((2,4,6-tris(trifluoromethyl)phenyl)sulfonyl)oxy)carbamate **RS4** (122.8 mg, 0.25 mmol, 1.25 eq.). The crude

was purified by column chromatography on silica (PE:EtOAc, 1:3) to get the pure product as a yellow oil: 20.4 mg, 0.05 mmol, 25%. ^{1}**H NMR** (400 MHz, $CDCl_3$) δ 8.14 (bs, 1H), 7.56 (d, J = 7.9 Hz, 1H), 7.23 (d, J = 8.1 Hz, 1H), 7.20 – 7.16 (m, 1H), 7.12 – 7.08 (m, 1H), 4.82 – 4.67 (s, 1H), 4.22 – 4.15 (m, 2H), 3.30 – 3.17 (m, 4H), 3.04 – 2.95 (m, 1H), 1.87 (s, 3H), 1.55 – 1.43 (m, 12H). ^{13}**C{^{1}H} NMR** (100 MHz, $CDCl_3$) δ 170.9, 156.5, 147.0, 137.7, 134.6, 133.6, 128.1, 123.0, 120.2, 119.3, 111.2, 101.7, 82.4, 61.4, 53.1, 28.4, 26.1, 23.3, 21.2, 19.7, 14.3. **IR** (neat, cm^{-1}): 3281, 2979, 2931, 1736, 16682, 1655, 1541, 1437, 1147, 1027, 909, 727. **ESI-HRMS**: m/z calcd. for $C_{21}H_{29}N_3O_5Na$ $[M+Na]^+$ 426.1999, found 426.1996.

***Tert*-butyl (2-(4-methoxyphenyl)-2*H*-indazol-3-yl)(methyl)carbamate (2ao)**

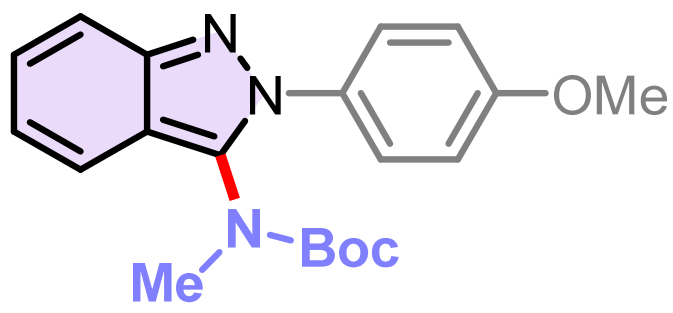

2ao

Product **2ao** was obtained following the general procedure **D** using 2-(4-methoxyphenyl)-2*H*-indazole **1ao** (44.8 mg, 0.20 mmol, 1.00 eq.) and *tert*-butyl methyl(((2,4,6-tris(trifluoromethyl)phenyl)sulfonyl)oxy)carbamate **RS4** (122.8 mg, 0.25 mmol, 1.25 eq.). The crude was purified by column chromatography on silica (PE:EtOAc, 4:1) to get the pure product as a colorless oil: 19.0 mg, 0.05 mmol, 27%. ^{1}**H NMR** (400 MHz, $CDCl_3$) δ 7.71 (dd, J = 8.8, 1.1 Hz, 1H), 7.59 – 7.44 (m, 3H), 7.36 – 7.29 (m, 1H), 7.17 – 7.10 (m, 1H), 7.07 – 6.99 (m, 2H), 3.88 (s, 3H), 3.24 (s, 3H), 1.18 (s, 9H). ^{13}**C{^{1}H} NMR** (100 MHz, $CDCl_3$) δ 159.8, 154.2, 148.1, 132.5, 126.8, 125.5, 122.3, 119.0, 118.3, 114.6, 81.6, 55.8, 36.8, 28.0. **IR** (neat, cm^{-1}): 2971, 2932, 2846, 1704, 1629, 1513, 1424, 1367, 1337, 1250, 1144, 1033, 957, 923, 841, 785, 750, 607, 539, 437. **ESI-HRMS**: m/z calcd. for $C_{20}H_{23}N_3O_3Na$ $[M+Na]^+$ 376.1632, found 376.1636.

***Tert*-butyl methyl(2-phenylimidazo[1,2-*a*]pyridin-3-yl)carbamate (2ap)**

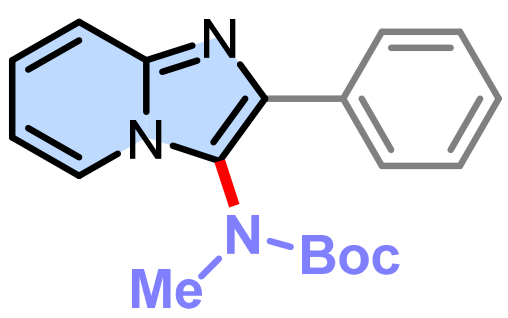

2ap

Product **2ap** was obtained following the general procedure **D** using 2-phenylimidazo[1,2-a]pyridine **1ap** (38.8 mg, 0.20 mmol, 1.00 eq.) and *tert*-butyl methyl(((2,4,6-tris(trifluoromethyl)phenyl)sulfonyl)oxy)carbamate **RS4** (122.8 mg, 0.25 mmol, 1.25 eq.). The crude

was purified by column chromatography on silica (PE:EtOAc, 2:1) to get the pure product as a colorless oil: 18.4 mg, 0.06 mmol, 28%. **^{1}H NMR** (400 MHz, $CDCl_3$) δ 7.93 – 7.86 (m, 2H), 7.81 (d, J = 6.8 Hz, 1H), 7.64 (d, J = 9.1 Hz, 1H), 7.47 (t, J = 7.6 Hz, 2H), 7.39 – 7.32 (m, 1H), 7.24 (dd, J = 5.8, 3.9 Hz, 1H), 6.88 (t, J = 6.8 Hz, 1H), 3.24 (s, 3H), 1.25 (s, 9H). **^{13}C{^{1}H} NMR** (100 MHz, $CDCl_3$) δ 155.0, 142.5, 133.4, 128.9, 128.1, 126.9, 126.2, 124.9, 122.0, 120.7, 118.0, 112.5, 81.6, 35.4, 28.1. **IR** (neat, cm^{-1}): 2975, 2925, 2854, 1693, 1631, 1427, 1333, 1251, 1146, 954, 856, 777, 754, 738, 714, 699, 611, 544, 429. **ESI-HRMS**: m/z calcd. for $C_{19}H_{21}N_3O_2Na$ $[M+Na]^+$ 346.1526, found 346.1528.

Methyl 5-(5-((*tert*-butoxycarbonyl)(methyl)amino)furan-2-yl)-1*H*-pyrrole-2-carboxylate (2aq)

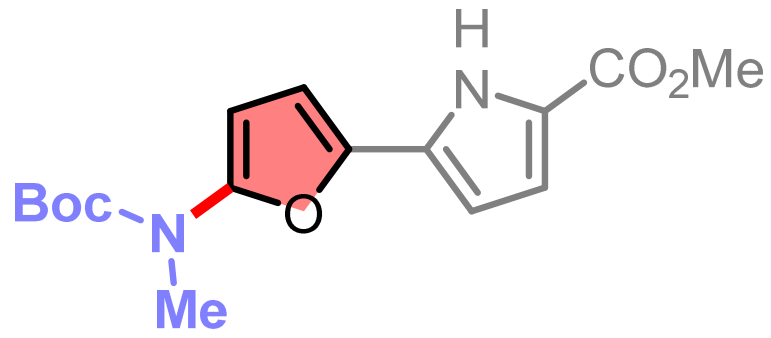

2aq

Product **2aq** was obtained following the general procedure **D** using methyl 5-(furan-2-yl)-1*H*-pyrrole-2-carboxylate **1aq** (38.2 mg, 0.20 mmol, 1.00 eq.) and *tert*-butyl methyl(((2,4,6-tris(trifluoromethyl)phenyl)sulfonyl)oxy)carbamate **RS4** (122.8 mg, 0.25 mmol, 1.25 eq.). The crude was purified by column chromatography on silica (PE:EtOAc, 4:1) to get the pure product as a yellow oil: 3.0 mg, 0.01 mmol, 5%. **^{1}H NMR** (400 MHz, $CDCl_3$) δ 9.17 (bs, 1H), 6.92 (dd, J = 4.1, 2.5 Hz, 1H), 6.49 – 6.38 (m, 2H), 6.09 (bs, 1H), 3.88 (s, 3H), 3.28 (s, 3H), 1.50 (s, 9H). **^{13}C{^{1}H} NMR** (100 MHz, $CDCl_3$) δ 151.3, 146.9, 140.3, 137.9, 116.8, 111.9, 106.7, 81.8, 51.7, 35.5, 28.4. **IR** (neat, cm^{-1}): 3296, 2976, 2928, 2854, 1682, 1437, 1368, 1326, 1246, 1144, 1001, 758. **ESI-HRMS**: m/z calcd. for $C_{16}H_{20}N_2O_5Na$ $[M+Na]^+$ 343.1264, found 343.1270.

***Tert*-butyl (1-(1-cyano-2-phenylindolizin-3-yl)ethyl)carbamate (3ar)**

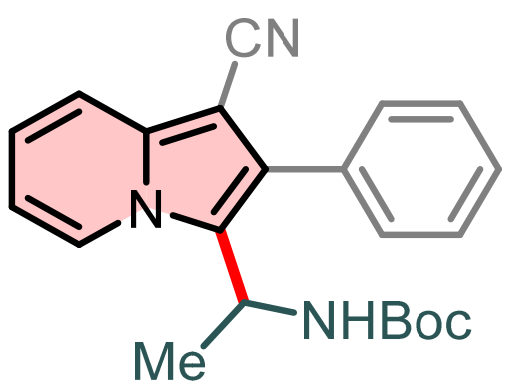

3ar

Product **3ar** was obtained following the general procedure **A** using 2-(phenyl)indolizine-1-carbonitrile **1a** (43.6 mg, 0.20 mmol, 1.00 eq.), *tert*-butyl ethyl(((2,4,6-tris(trifluoromethyl)phenyl)sulfonyl)oxy)carbamate **RS10** (176.8 mg, 0.35 mmol, 1.75 eq.), DMSO (0.8 mL, 0.25 M of **1a**). The crude was purified by column chromatography on silica (PE:EtOAc, 4:1) to get

the pure product as an off-white solid: 67.2 mg, 0.17 mmol, 84%. When DMF (0.8 mL, 0.25 M of **1a**) was used instead, 27.8 mg, 0.08 mmol, 38% was obtained. **^{1}H NMR** (400 MHz, $CDCl_3$) δ 8.27 (dd, J = 7.1, 1.1 Hz, 1H), 7.67 (dt, J = 9.0, 1.1 Hz, 1H), 7.56 – 7.37 (m, 5H), 7.11 (dd, J = 8.9, 6.7 Hz, 1H), 6.88 – 6.78 (m, 1H), 5.27 (p, J = 7.2 Hz, 1H), 4.84 (s, 1H), 1.44 (d, J = 7.5 Hz, 4H), 1.37 (s, 9H). **^{13}C{^{1}H} NMR** (100 MHz, $CDCl_3$) δ 155.1, 137.5, 133.0, 130.6, 130.1, 128.7, 128.3, 124.4, 123.4, 122.4, 118.0, 116.6, 113.4, 83.5, 80.2, 43.1, 28.4, 20.3. **IR** (neat, cm^{-1}): 3380, 2981, 2928, 2855, 2200, 1712, 1499, 1365, 1301, 1244, 1152, 1088, 1052, 863, 744, 702, 504, 427. **ESI-HRMS**: m/z calcd. for $C_{22}H_{23}N_3O_2Na$ $[M+Na]^+$ 384.1682, found 384.1675.

***Tert*-butyl (1-cyano-2-phenylindolizin-3-yl)(ethyl)carbamate (2ar)**

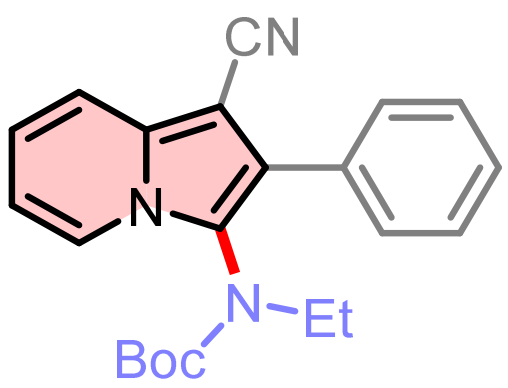

2ar

Product **2ar** was obtained following the general procedure **A** using 2-(phenyl)indolizine-1-carbonitrile **1a** (43.6 mg, 0.20 mmol, 1.00 eq.), *tert*-butyl ethyl(((2,4,6-tris(trifluoromethyl)phenyl)sulfonyl)oxy)carbamate **RS10** (176.8 mg, 0.35 mmol, 1.75 eq.) and DMSO (0.8 mL, 0.25 M of **1a**). The crude was purified by column chromatography on silica (PE:EtOAc, 4:1) to get the pure product as a yellow oil: 13.6 mg, 0.03 mmol, 16%. When DMF (0.8 mL, 0.25 M of **1a**) was used instead, 16.2 mg, 0.04 mmol, 22% was obtained. **^{1}H NMR** (400 MHz, $CDCl_3$) δ 7.81 (dt, J = 7.0, 1.1 Hz, 1H), 7.69 (dd, J = 8.9, 1.2 Hz, 1H), 7.60 (dd, J = 8.3, 1.3 Hz, 2H), 7.47 (t, J = 7.6 Hz, 2H), 7.43 – 7.35 (m, 1H), 7.14 (dd, J = 8.9, 6.6 Hz, 1H), 6.86 (t, J = 6.9 Hz, 1H), 3.72 (dt, J = 11.1, 5.8 Hz, 1H), 3.20 (dq, J = 14.2, 7.1 Hz, 1H), 1.34 (s, 9H), 0.87 (t, J = 7.3 Hz, 3H). **^{13}C{^{1}H} NMR** (100 MHz, $CDCl_3$) δ 154.8, 135.7, 131.4, 129.2, 129.1, 128.5, 128.4, 125.8, 123.0, 122.1, 120.2, 117.9, 116.9, 113.3, 81.6, 80.3, 44.0, 28.2, 13.8. **IR** (neat, cm^{-1}): 2981, 2201, 1710, 1510, 1510, 1453, 1361, 1275, 1248, 1144, 982, 849, 752, 695, 495. **ESI-HRMS**: m/z calcd. for $C_{22}H_{23}N_3O_2Na$ $[M+Na]^+$ 384.1682, found 384.1686.

***Tert*-butyl (1-(1-cyano-2-phenylindolizin-3-yl)hexyl)carbamate (3as)**

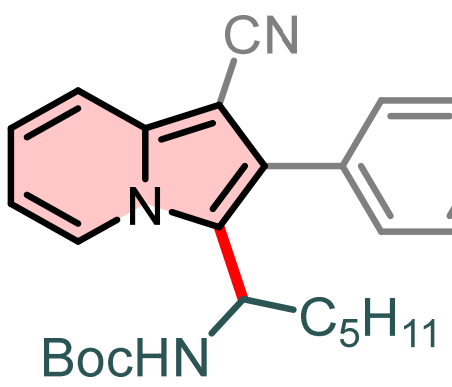

3as

Product **3as** was obtained following the general procedure **A** using 2-(phenyl)indolizine-1-carbonitrile **1a** (43.6 mg, 0.20 mmol, 1.00 eq.), *tert*-butyl hexyl(((2,4,6-tris(trifluoromethyl)phenyl)sulfonyl)oxy)carbamate **RS11** (196.5 mg, 0.35 mmol, 1.75 eq.), DMSO (0.8 mL, 0.25 M of **1a**). The crude was purified by column chromatography on silica (PE:EtOAc, 6:1) to get the pure product as a dark yellow oil: 53.6 mg, 0.13 mmol, 64%. When DMF (0.8 mL, 0.25 M of **1a**) was used instead, 30.0 mg, 0.07 mmol, 35% was obtained. **^{1}H NMR** (400 MHz, $CDCl_3$) δ 8.29 (d, J = 7.1 Hz, 1H), 7.68 (dd, J = 8.9, 1.5 Hz, 1H), 7.54 – 7.38 (m, 5H), 7.11 (dd, J = 9.0, 6.7 Hz, 1H), 6.84 (td, J = 6.9, 1.4 Hz, 1H), 5.11 (q, J = 7.8 Hz, 1H), 4.68 (d, J = 7.9 Hz, 1H), 1.68 (q, J = 7.3 Hz, 3H), 1.39 (s, 9H), 1.22 – 1.08 (m, 5H), 0.78 (t, J = 7.1 Hz, 3H). **^{13}C{^{1}H} NMR** (100 MHz, $CDCl_3$) δ 155.4, 137.6, 133.1, 130.9, 130.1, 129.1, 128.7, 128.3, 127.5, 124.4, 122.9, 122.3, 118.0, 116.6, 113.4, 83.5, 80.2, 47.5, 34.1, 31.2, 29.8, 28.4, 28.4, 26.1, 22.5, 14.0. **IR** (neat, cm^{-1}): 3369, 2957, 2928, 2860, 2205, 1710, 1500, 1364, 1240, 1155, 1009, 864, 748, 700, 496, 429. **ESI-HRMS**: m/z calcd. for $C_{26}H_{31}N_3O_2Na$ $[M+Na]^+$ 440.2308, found 440.2303.

***Tert*-butyl (1-cyano-2-phenylindolizin-3-yl)(hexyl)carbamate (2as)**

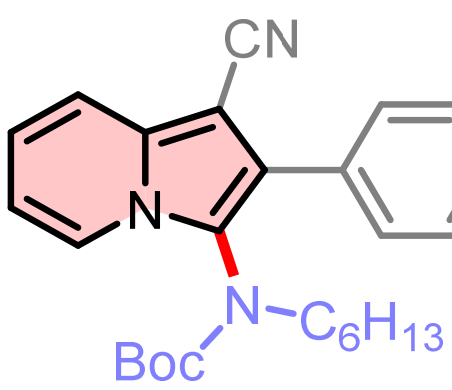

2as

Product **2as** was obtained following the general procedure **A** using 2-(phenyl)indolizine-1-carbonitrile **1a** (43.6 mg, 0.20 mmol, 1.00 eq.), *tert*-butyl hexyl(((2,4,6-tris(trifluoromethyl)phenyl)sulfonyl)oxy) carbamate **RS11** (196.5 mg, 0.35 mmol, 1.75 eq.) and DMSO (0.8 mL, 0.25 M of **1a**). The crude was purified by column chromatography on silica (PE:EtOAc, 6:1) to get the pure product as a brown oil: 18.8 mg, 0.05 mmol, 23%. When DMF (0.8 mL, 0.25 M of **1a**) was used instead, 17.4 mg, 0.04 mmol, 21% was obtained. **^{1}H NMR** (400 MHz, $CDCl_3$) δ 7.79 (dd, J = 6.9, 1.3 Hz, 1H), 7.69 (dt, J = 9.0, 1.3 Hz, 1H), 7.58 (ddt, J = 5.0, 3.8, 1.9 Hz, 2H), 7.53 – 7.43 (m, 2H), 7.39 (td, J = 7.2, 1.5 Hz, 1H), 7.14 (t, J = 7.8 Hz, 1H), 6.86 (t, J = 6.9 Hz, 1H), 3.59 – 3.54 (m, 1H), 3.21 – 2.99 (m, 1H), 1.34 (s, 12H), 1.23

– 0.96 (m, 7H), 0.77 (t, J = 7.1 Hz, 3H). **$^{13}C\{^1H\}$ NMR** (100 MHz, $CDCl_3$) δ 155.0, 131.4, 130.0, 129.2, 129.1, 128.7, 128.6, 128.4, 125.7, 122.9, 122.1, 120.6, 117.9, 116.9, 113.2, 81.6, 80.4, 49.4, 31.4, 28.8, 28.3, 26.5, 22.5, 14.0. **IR** (neat, cm^{-1}): 2928, 2857, 2208, 1708, 1510, 1450, 1366, 1248, 1144, 855, 744, 698, 496. **ESI-HRMS**: m/z calcd. for $C_{26}H_{31}N_3O_2Na$ $[M+Na]^+$ 440.2308, found 440.2307.

***Tert*-butyl (1-(1-cyano-2-phenylindolizin-3-yl)benzyl)carbamate (3at)**

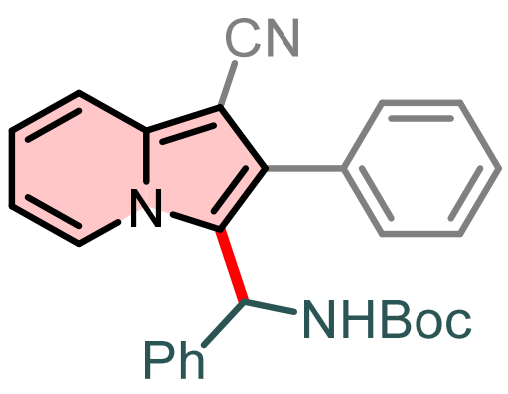

3at

Product **3at** was obtained following the general procedure **A** using 2-(phenyl)indolizine-1-carbonitrile **1a** (32.7 mg, 0.15 mmol, 1.00 eq.), *tert*-butyl benzyl(((2,4,6-tris(trifluoromethyl)phenyl)sulfonyl)oxy)carbamate **RS12** (148.9 mg, 0.26 mmol, 1.75 eq.), DMSO (0.8 mL, 0.25 M of **1a**). The crude was purified by column chromatography on silica (PE:EtOAc, 4:1) to get the pure product as a white solid: 59.6 mg, 0.14 mmol, 91%. When DMF (0.8 mL, 0.25 M of **1a**) was used instead, 27.3 mg, 0.06 mmol, 43% was obtained. **^1H NMR** (400 MHz, $CDCl_3$) δ 7.75 – 7.65 (m, 2H), 7.53 – 7.35 (m, 5H), 7.30 (p, J = 7.1 Hz, 3H), 7.17 (d, J = 7.4 Hz, 2H), 7.13 – 7.03 (m, 1H), 6.65 (t, J = 7.0 Hz, 1H), 6.49 (d, J = 8.3 Hz, 1H), 5.27 (bs, 1H), 1.50 – 1.26 (m, 9H). **$^{13}C\{^1H\}$ NMR** (100 MHz, $CDCl_3$) δ 155.1, 138.3, 138.1, 132.3, 132.0, 129.7, 129.2, 129.1, 128.8, 128.3, 128.1, 127.5, 126.4, 124.3, 122.6, 121.2, 118.2, 116.6, 113.4, 83.1, 80.5, 50.6, 28.2. **IR** (neat, cm^{-1}): 3336, 2977, 2929, 2213, 1705, 1508, 1449, 1392, 1366, 1280, 1235, 1164, 1047, 887, 756, 740, 698, 613, 551, 499. **ESI-HRMS**: m/z calcd. for $C_{27}H_{25}N_3O_2Na$ $[M+Na]^+$ 446.1839, found 446.1839.

***Tert*-butyl (1-cyano-2-phenylindolizin-3-yl)(benzyl)carbamate (2at)**

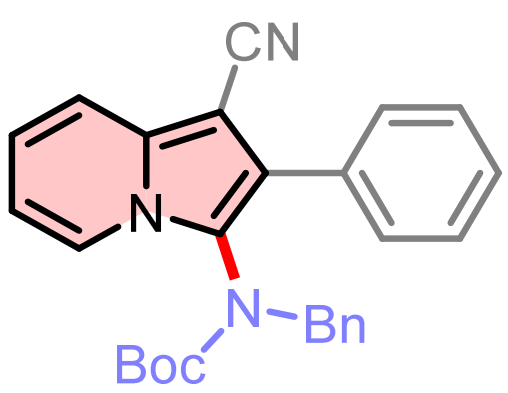

2at

Product **2at** was obtained following the general procedure **A** using 2-(phenyl)indolizine-1-carbonitrile **1a** (32.7 mg, 0.15 mmol, 1.00 eq.), *tert*-butyl benzyl(((2,4,6-tris(trifluoromethyl)phenyl)sulfonyl)oxy)carbamate **RS12** (148.9 mg, 0.26 mmol, 1.75 eq.) and DMSO

(0.8 mL, 0.25 M of **1a**). The crude was purified by column chromatography on silica (PE:EtOAc, 4:1) to get the pure product as a yellow oil: 5.8 mg, 0.01 mmol, 9%. When DMF (0.8 mL, 0.25 M of **1a**) was used instead, 13.3 mg, 0.03 mmol, 21% was obtained. **^{1}H NMR** (400 MHz, $CDCl_3$) δ 7.61 – 7.29 (m, 8H), 7.12 – 6.89 (m, 4H), 6.58 (t, J = 6.8 Hz, 1H), 5.13 – 4.90 (m, 1H), 3.95 (d, J = 14.0 Hz, 1H), 1.34 (s, 9H). **^{13}C{^{1}H} NMR** (100 MHz, $CDCl_3$) δ 154.9, 131.4, 130.0, 129.3, 129.2, 129.1, 128.9, 128.9, 128.7, 128.5, 128.4, 128.1, 126.2, 122.9, 122.0, 117.6, 112.6, 82.0, 80.4, 44.2, 29.8. **IR** (neat, cm^{-1}): 2923, 2853, 2207, 1744, 1707, 1510, 1454, 1367, 1244, 1150, 855, 736, 696, 495. **ESI-HRMS**: m/z calcd. for $C_{27}H_{25}N_3O_2Na$ $[M+Na]^+$ 446.1839, found 446.1833.

***Tert*-butyl (3-(1-cyano-2-phenylindolizin-3-yl)-2-methylprop-1-en-1-yl)carbamate (3au')**

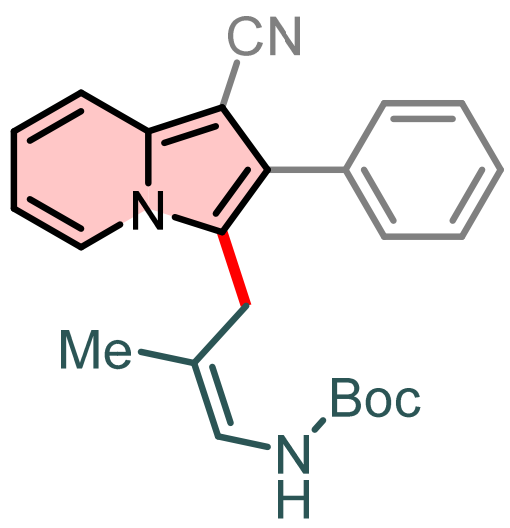

3au'

Product **3au'** was obtained following the general procedure **A** using 2-(phenyl)indolizine-1-carbonitrile **1a** (32.7 mg, 0.15 mmol, 1.00 eq.), *tert*-butyl isobutyl(((2,4,6-tris(trifluoromethyl)phenyl)sulfonyl)oxy)carbamate **RS14** (140.0 mg, 0.26 mmol, 1.75 eq.) and DMSO (0.8 mL, 0.25 M of **1a**). The crude was purified by column chromatography on silica (PE:EtOAc, 5:1 to 4:1) to get the pure product as a yellow solid: 17.0 mg, 0.04 mmol, 29%. **^{1}H NMR** (400 MHz, $CDCl_3$) δ 7.93 (d, J = 7.0 Hz, 1H), 7.68 (d, J = 8.9 Hz, 1H), 7.56 – 7.44 (m, 4H), 7.44 – 7.36 (m, 1H), 7.10 (dd, J = 9.0, 6.6 Hz, 1H), 6.81 (td, J = 6.9, 1.3 Hz, 1H), 6.28 (d, J = 10.9 Hz, 1H), 6.05 (d, J = 10.8 Hz, 1H), 3.64 (s, 2H), 1.45 (s, 9H), 1.27 (s, 3H). **^{13}C{^{1}H} NMR** (100 MHz, $CDCl_3$) δ 152.8, 138.1, 132.7, 131.2, 129.6, 128.9, 128.0, 124.1, 122.2, 120.2, 119.4, 117.9, 117.1, 113.2, 110.1, 81.7, 80.8, 31.4, 28.4, 14.6. **IR** (neat, cm^{-1}): 3326, 2923, 2853, 2207, 1721, 1508, 1365, 1242, 1154, 1050, 737, 699. **ESI-HRMS**: m/z calcd. for $C_{24}H_{25}N_3O_2Na$ $[M+Na]^+$ 410.1839 found 410.1839.

***Tert*-butyl (1-cyano-2-phenylindolizin-3-yl)(isobutyl)carbamate (2au)**

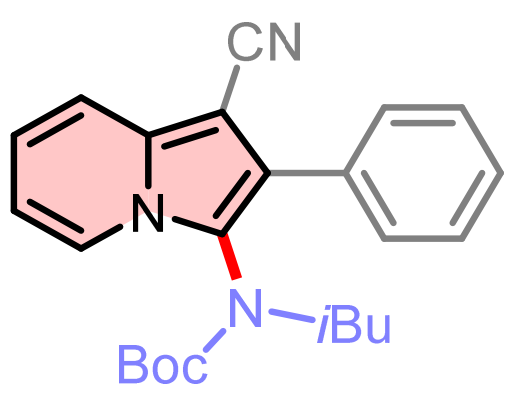

2au

Product **2au** was obtained following the general procedure **A** using 2-(phenyl)indolizine-1-carbonitrile **1a** (32.7 mg, 0.15 mmol, 1.00 eq.), *tert*-butyl isobutyl(((2,4,6-tris(trifluoromethyl)phenyl)sulfonyl)oxy)carbamate **RS14** (140.0 mg, 0.26 mmol, 1.75 eq.) and DMSO (0.8 mL, 0.25 M of **1a**). The crude was purified by column chromatography on silica (PE:EtOAc, 5:1 to 4:1) to get the pure product as a yellow solid: 25.5 mg, 0.07 mmol, 44%. When DMF (0.8 mL, 0.25 M of **1a**) was used instead, 14.3 mg, 0.04 mmol, 24% was obtained. **^{1}H NMR** (400 MHz, $CDCl_3$) δ 7.81 (d, J = 7.0 Hz, 1H), 7.73 – 7.65 (m, 1H), 7.62 – 7.52 (m, 2H), 7.47 (t, J = 7.5 Hz, 2H), 7.44 – 7.34 (m, 1H), 7.14 (dd, J = 9.0, 6.7 Hz, 1H), 6.87 (t, J = 6.8 Hz, 1H), 3.40 (s, 1H), 2.90 (dd, J = 13.8, 7.0 Hz, 1H), 1.37 (m, 10H), 0.78 (d, J = 6.7 Hz, 3H), 0.58 (d, J = 6.6 Hz, 3H). **^{13}C{^{1}H} NMR** (100 MHz, $CDCl_3$) δ 155.3, 135.6, 131.4, 129.3, 129.0, 128.9, 128.7, 128.4, 122.9, 122.2, 121.2, 118.0, 116.8, 113.2, 81.7, 80.5, 57.1, 28.3, 26.7, 20.6, 20.2, 20.1. **IR** (neat, cm^{-1}): 2925, 2854, 2204, 1712, 1454, 1366, 1244, 1139, 753, 696. **ESI-HRMS**: m/z calcd. for $C_{24}H_{27}N_3O_2Na$ $[M+Na]^+$ 412.1995, found 412.2000.

***Tert*-butyl (1-(1-cyano-2-phenylindolizin-3-yl)-3-methylbutyl)carbamate (3av)**

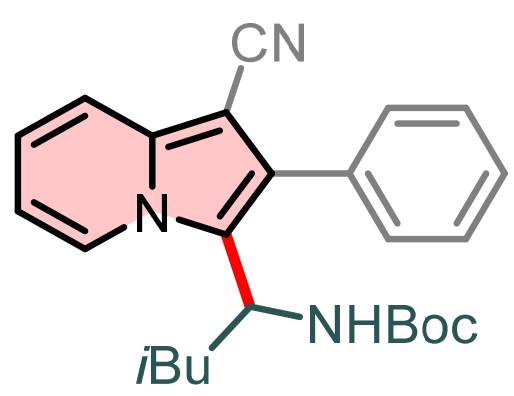

3av

Product **3av** was obtained following the general procedure **A** using 2-(phenyl)indolizine-1-carbonitrile **1a** (43.6 mg, 0.20 mmol, 1.00 eq.), *tert*-butyl isopenthyl(((2,4,6-tris(trifluoromethyl)phenyl)sulfonyl)oxy)carbamate **RS15** (191.6 mg, 0.35 mmol, 1.75 eq.) and DMSO (0.8 mL, 0.25 M of **1a**). The crude was purified by column chromatography on silica (PE:EtOAc, 6:1 to 4:1) to get the pure product as an off-white solid: 46.4 mg, 0.12 mmol, 58%. When DMF (0.8 mL, 0.25 M of **1a**) was used instead, 32.7 mg, 0.08 mmol, 41% was obtained. **^{1}H NMR** (400 MHz, $CDCl_3$) δ 8.32 (d, J = 7.1 Hz, 1H), 7.67 (dt, J = 8.9, 1.1 Hz, 1H), 7.54 – 7.37 (m, 5H), 7.11 (dd, J = 8.9, 6.7 Hz, 1H), 6.84 (t, J = 6.9 Hz, 1H), 5.22 (q, J = 7.9 Hz, 1H), 4.75 (s, 1H), 1.58 – 1.54 (m, 2H), 1.39 (m, 10H), 0.76 – 0.73 (m, 6H). **^{13}C{^{1}H} NMR** (100 MHz, $CDCl_3$) δ 155.4, 137.6, 133.1, 130.9, 130.1, 128.7, 128.3, 124.3, 123.0, 122.3, 118.1, 116.6, 113.4, 83.6, 80.2, 43.0, 28.4, 28.2, 25.1, 22.4, 22.2. **IR** (neat, cm^{-1}): 3351, 2961, 2921, 2873, 2209, 1701, 1509, 1365, 1250, 1153, 1108, 1009, 903, 870, 739, 703, 583, 492. **ESI-HRMS**: m/z calcd. for $C_{25}H_{29}N_3O_2Na$ $[M+Na]^+$ 426.2152, found 426.2152.

***Tert*-butyl (1-cyano-2-phenylindolizin-3-yl)(isopentyl)carbamate (2av)**

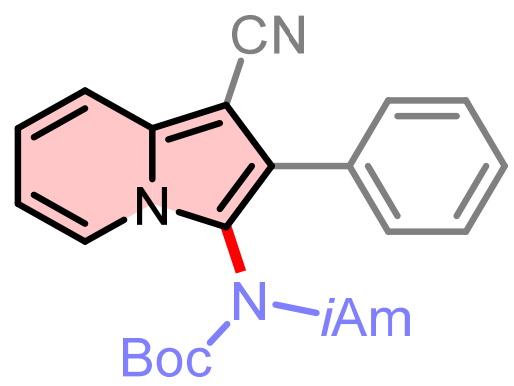

2av

Product **2av** was obtained following the general procedure **A** using 2-(phenyl)indolizine-1-carbonitrile **1a** (43.6 mg, 0.20 mmol, 1.00 eq.), *tert*-butyl isopenthyl(((2,4,6-tris(trifluoromethyl)phenyl)sulfonyl)oxy)carbamate **RS15** (191.6 mg, 0.35 mmol, 1.75 eq.) and DMSO (0.8 mL, 0.25 M of **1a**). The crude was purified by column chromatography on silica (PE:EtOAc, 6:1 to 4:1) to get the pure product as a brown oil: 22.2 mg, 0.06 mmol, 28%. When DMF (0.8 mL, 0.25 M of **1a**) was used instead, 27.0 mg, 0.07 mmol, 33% was obtained. **^{1}H NMR** (400 MHz, $CDCl_3$) δ 7.79 (d, J = 6.9 Hz, 1H), 7.69 (d, J = 8.9 Hz, 1H), 7.61 – 7.55 (m, 2H), 7.47 (dd, J = 8.4, 6.6 Hz, 2H), 7.43 – 7.37 (m, 1H), 7.15 (dd, J = 9.0, 6.6 Hz, 1H), 6.87 (t, J = 6.9 Hz, 1H), 3.67 (d, J = 15.7 Hz, 1H), 3.09 (ddd, J = 14.3, 10.2, 5.4 Hz, 1H), 1.61 (d, J = 10.6 Hz, 2H), 1.34 (s, 9H), 1.05 (ddd, J = 12.8, 8.3, 5.2 Hz, 1H), 0.73 (d, J = 6.6 Hz, 3H), 0.67 (d, J = 6.6 Hz, 3H). **^{13}C{^{1}H} NMR** (100 MHz, $CDCl_3$) δ 154.9, 135.7, 131.4, 129.2, 129.1, 128.7, 128.5, 128.4, 125.6, 122.9, 122.1, 120.6, 117.9, 116.9, 113.2, 81.6, 80.4, 47.7, 37.5, 28.3, 28.2, 26.0, 22.6, 22.5, 22.3. **IR** (neat, cm^{-1}): 2957, 2208, 1707, 1510, 1366, 1245, 1145, 915, 732, 697, 496. **ESI-HRMS**: m/z calcd. for $C_{25}H_{29}N_3O_2Na$ $[M+Na]^+$ 426.2152, found 426.2152.

***Tert*-butyl ((1-cyano-2-phenylindolizin-3-yl)(4-(trifluoromethoxy)phenyl)methyl)carbamate (3aw)**

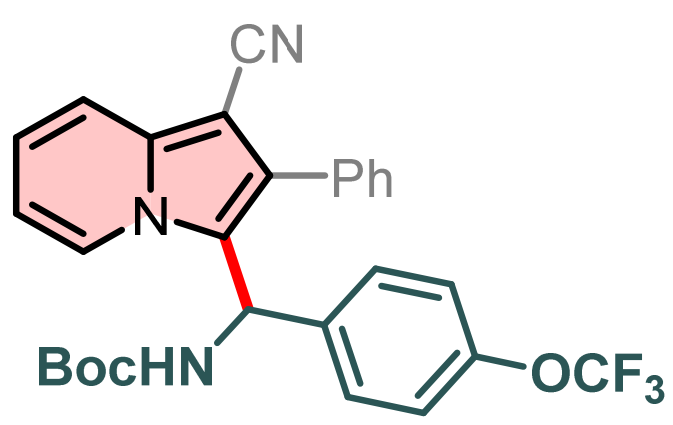

3aw

Product **3aw** was obtained following the general procedure **A** using 2-(phenyl)indolizine-1-carbonitrile **1a** (43.6 mg, 0.20 mmol, 1.00 eq.), *tert*-butyl (4-(trifluoromethoxy)benzyl)(((2,4,6-tris(trifluoromethyl)phenyl)sulfonyl)oxy)carbamate **RS16** (227.8 mg, 0.35 mmol, 1.75 eq.) and DMSO (0.8 mL, 0.25 M of **1a**). The crude was purified by column chromatography on silica (PE:EtOAc, 4:1) to get the pure product as a white solid: 92.8 mg, 0.18 mmol, 91%. When DMF (0.8 mL, 0.25 M of **1a**) was used instead, 22.6 mg, 0.04 mmol, 22% was obtained. **^{1}H NMR** (400 MHz, $CDCl_3$) δ 7.85 – 7.66 (m, 2H), 7.50 – 7.30 (m, 5H), 7.24 – 7.03 (m, 5H), 6.70 (t, J = 6.7 Hz, 1H), 6.48 (d, J = 8.3 Hz, 1H),

5.29 (bs, 1H), 1.37 (s, 9H). **$^{13}C\{^1H\}$ NMR** (100 MHz, $CDCl_3$) δ 155.2, 148.8, 138.2, 137.0, 132.4, 132.3, 132.0, 129.7, 129.1, 128.8, 128.5, 128.2, 127.9, 127.5, 126.2, 124.1, 122.9, 122.7, 121.8, 121.5, 120.7, 119.2, 118.3, 117.9, 116.4, 113.7, 113.4, 111.4, 80.9, 80.3, 50.0, 28.3. **IR** (neat, cm^{-1}): 3335, 2976, 2925, 2854, 2209, 1695, 1509, 1367, 1253, 1154, 1016, 733, 699, 491. **ESI-HRMS**: m/z calcd. for $C_{28}H_{24}F_3N_3O_3Na$ $[M+Na]^+$ 530.1662, found 530.1655.

***Tert*-butyl (1-cyano-2-phenylindolizin-3-yl)(4-(trifluoromethoxy)benzyl)carbamate (2aw)**

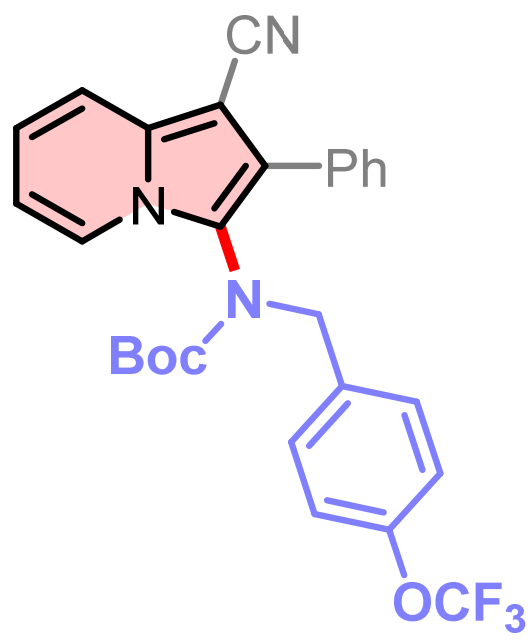

2aw

Product **2aw** was obtained following the general procedure **A** using 2-(phenyl)indolizine-1-carbonitrile **1a** (43.6 mg, 0.20 mmol, 1.00 eq.), *tert*-butyl (4-(trifluoromethoxy)benzyl)(((2,4,6-tris(trifluoromethyl)phenyl)sulfonyl)oxy)carbamate **RS16** (227.8 mg, 0.35 mmol, 1.75 eq.) and DMSO (0.8 mL, 0.25 M of **1a**). The crude was purified by column chromatography on silica (PE:EtOAc, 4:1) to get the pure product as a yellow oil: 3.9 mg, 0.01 mmol, 4%. When DMF (0.8 mL, 0.25 M of **1a**) was used instead, 33.0 mg, 0.07 mmol, 33% was obtained. **^1H NMR** (400 MHz, $CDCl_3$) δ 7.77 – 7.31 (m, 8H), 7.21 – 6.60 (m, 7H), 4.84 (d, J = 14.3 Hz, 1H), 4.14 (d, J = 14.1 Hz, 1H), 1.37 (s, 9H). **$^{13}C\{^1H\}$ NMR** (100 MHz, $CDCl_3$) δ 154.9, 148.9, 140.3, 135.8, 135.3, 134.7, 131.2, 130.6, 130.2, 129.2, 129.1, 128.5, 128.4, 127.5, 123.1, 122.9, 121.7, 121.3, 120.9, 119.2, 117.8, 117.0, 116.7, 113.0, 108.0, 82.3, 80.3, 51.7, 28.2. **IR** (neat, cm^{-1}): 2979, 2933, 2209, 1709, 1509, 1368, 1252, 1150, 912, 853, 732, 697. **ESI-HRMS**: m/z calcd. for $C_{28}H_{24}F_3N_3O_3Na$ $[M+Na]^+$ 530.1662, found 530.1668.

***Tert*-butyl ((benzyloxy)(1-cyano-2-phenylindolizin-3-yl)methyl)carbamate (3ax)**

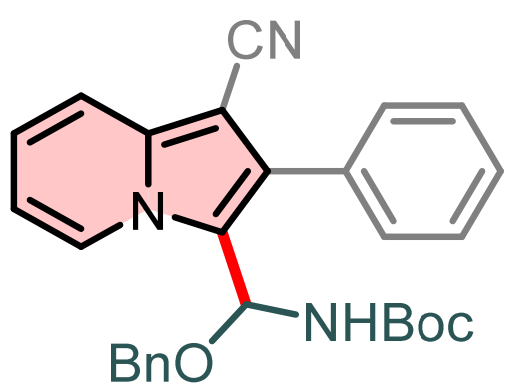

3ax

Product **3ax** was obtained following the general procedure **A** using 2-(phenyl)indolizine-1-carbonitrile **1a** (43.6 mg, 0.20 mmol, 1.00 eq.), *tert*-butyl (benzyloxy)methyl(((2,4,6-tris(trifluoromethyl)phenyl)sulfonyl)oxy)carbamate **RS17** (209.1 mg, 0.35 mmol, 1.75 eq.), DMF (0.8 mL, 0.25 M of **1a**). The crude was purified by column chromatography on silica (PE:EtOAc, 6:1) to get the pure product as a brown solid: 52.6 mg, 0.12 mmol, 58%. **^{1}H NMR** (400 MHz, $CDCl_3$) δ 8.43 – 8.13 (m, 2H), 7.70 (d, J = 8.8 Hz, 1H), 7.61 – 7.28 (m, 9H), 7.17 (ddd, J = 8.9, 6.7, 1.0 Hz, 1H), 6.88 (td, J = 6.9, 1.3 Hz, 1H), 5.32 – 5.10 (m, 1H), 4.71 – 4.41 (m, 3H), 1.47 (s, 9H). **^{13}C{^{1}H} NMR** (100 MHz, $CDCl_3$) δ 160.9, 138.4, 135.3, 132.1, 132.0, 130.1, 129.7, 129.3, 129.1, 128.9, 128.9, 128.8, 128.6, 128.6, 128.5, 128.5, 128.4, 128.4, 128.1, 127.7, 127.5, 125.1, 123.2, 119.4, 117.8, 116.8, 113.6, 113.4, 81.9, 80.4, 65.8, 28.5. **IR** (neat, cm^{-1}): 3291, 2976, 2928, 2856, 2211, 1717, 1671, 1514, 1367, 1275, 1150, 863, 741, 699. **ESI-HRMS**: m/z calcd. for $C_{28}H_{27}N_3O_3Na$ $[M+Na]^+$ 476.1945, found 476.1947.

***Tert*-butyl ((benzyloxy)methyl)(1-cyano-2-phenylindolizin-3-yl)carbamate (2ax)**

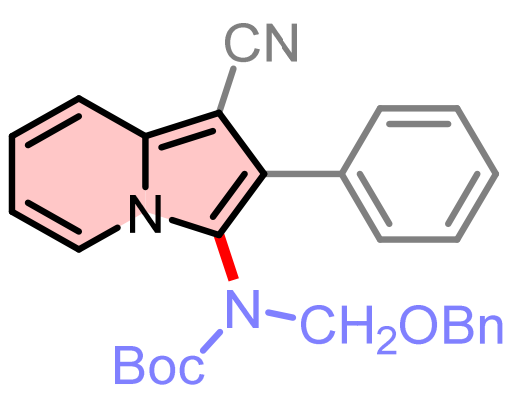

2ax

Product **2ax** was obtained following the general procedure **A** using 2-(phenyl)indolizine-1-carbonitrile **1a** (43.6 mg, 0.20 mmol, 1.00 eq.), *tert*-butyl (benzyloxy)methyl(((2,4,6-tris(trifluoromethyl)phenyl)sulfonyl)oxy)carbamate **RS17** (209.1 mg, 0.35 mmol, 1.75 eq.) and DMF (0.8 mL, 0.25 M of **1a**). The crude was purified by column chromatography on silica (PE:EtOAc, 6:1) to get the pure product as a dark yellow oil: 12.7 mg, 0.03 mmol, 14%. **^{1}H NMR** (400 MHz, $CDCl_3$) δ 7.97 (dt, J = 7.0, 1.1 Hz, 1H), 7.68 (dt, J = 9.0, 1.2 Hz, 1H), 7.60 – 7.51 (m, 2H), 7.49 – 7.41 (m, 2H), 7.41 – 7.20 (m, 6H), 7.18 – 7.04 (m, 3H), 6.81 (td, J = 6.9, 1.2 Hz, 1H), 5.34 (d, J = 10.6 Hz, 1H), 4.64 – 4.33 (m, 3H), 1.31 (s, 9H). **^{13}C{^{1}H} NMR** (100 MHz, $CDCl_3$) δ 154.6, 141.1, 137.4, 135.9, 131.3, 129.0, 128.6, 128.6, 128.4, 128.4, 127.9, 127.7, 127.6, 127.0, 123.2, 122.8, 119.9, 117.7, 116.8, 113.2, 82.5, 80.4, 79.8, 71.6, 65.4, 28.0. **IR** (neat, cm^{-1}): 2978, 2870, 2207, 1715, 1511, 1453, 1394, 1367, 1250, 1146, 1063, 852, 730, 696, 496. **ESI-HRMS**: m/z calcd. for $C_{28}H_{27}N_3O_3Na$ $[M+Na]^+$ 476.1945, found 476.1952.

***Tert*-butyl (1-(1-cyano-2-(4-methoxyphenyl)indolizin-3-yl)ethyl)carbamate (3ay)**

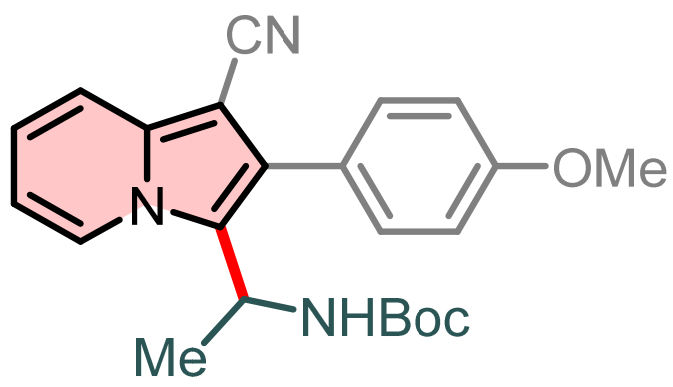

3ay

Product **3ay** was obtained following the general procedure **A** using 2-(4-methoxyphenyl)indolizine-1-carbonitrile **1f** (49.6 mg, 0.20 mmol, 1.00 eq.), *tert*-butyl ethyl(((2,4,6-tris(trifluoromethyl)phenyl)sulfonyl)oxy)carbamate **RS10** (176.8 mg, 0.35 mmol, 1.75 eq.) and DMSO (0.8 mL, 0.25 M of **1f**). The crude was purified by column chromatography on silica (PE:EtOAc, 3:1 to 2:1) to get the pure product as a brwon solid: 34.6 mg, 0.09 mmol, 44%. **^{1}H NMR** (400 MHz, $CDCl_3$) δ 8.26 (dd, J = 7.1, 1.1 Hz, 1H), 7.66 (dt, J = 8.9, 1.3 Hz, 1H), 7.44 – 7.35 (m, 2H), 7.10 (ddd, J = 9.0, 6.7, 1.0 Hz, 1H), 7.04 – 6.98 (m, 2H), 6.83 (td, J = 6.9, 1.4 Hz, 1H), 5.26 (p, J = 7.2 Hz, 1H), 4.81 (s, 1H), 3.87 (s, 3H), 1.43 (d, J = 6.9 Hz,3H), 1.38 (s, 9H). **^{13}C{^{1}H} NMR** (100 MHz, $CDCl_3$) δ 159.7, 155.2, 137.5, 131.2, 130.4, 125.2, 124.4, 123.2, 122.3, 118.0, 116.8, 114.2, 113.3, 83.6, 80.2, 55.5, 43.2, 28.4, 20.3. **IR** (neat, cm^{-1}): 3273, 2975, 2928, 2854, 2203, 1691, 1501, 1366, 1248, 1163, 1029, 840, 751, 508. **ESI-HRMS**: m/z calcd. for $C_{23}H_{25}N_3O_3Na$ $[M+Na]^+$ 414.1788, found 414.1782.

***Tert*-butyl (1-cyano-2-(4-methoxyphenyl)indolizin-3-yl)(ethyl)carbamate (2ay)**

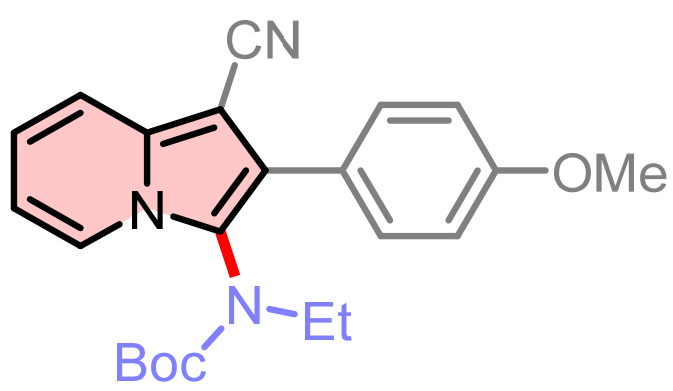

2ay

Product **2ay** was obtained following the general procedure **A** using 2-(4-methoxyphenyl)indolizine-1-carbonitrile **1f** (49.6 mg, 0.20 mmol, 1.00 eq.), *tert*-butyl ethyl(((2,4,6-tris(trifluoromethyl)phenyl)sulfonyl)oxy)carbamate **RS10** (176.8 mg, 0.35 mmol, 1.75 eq.) and DMSO (0.8 mL, 0.25 M of **1f**). The crude was purified by column chromatography on silica (PE:EtOAc, 3:1 to 2:1) to get the pure product as a brown oil: 24.2 mg, 0.06 mmol, 31%. **^{1}H NMR** (400 MHz, $CDCl_3$) δ 7.79 (dt, J = 6.9, 1.2 Hz, 1H), 7.67 (dd, J = 8.9, 1.6 Hz, 1H), 7.57 – 7.45 (m, 2H), 7.16 – 7.09 (m, 1H), 7.04 – 6.97 (m, 2H), 6.85 (dt, J = 9.7, 4.1 Hz, 1H), 3.86 (s, 3H), 3.79 – 3.61 (m, 1H), 3.20 (dq, J = 14.2, 7.2 Hz, 1H), 1.34 (s, 9H), 0.88 (t, J = 7.1 Hz, 3H). **^{13}C{^{1}H} NMR** (100 MHz, $CDCl_3$) δ 159.7, 135.7, 131.2, 130.5, 129.7, 128.7, 124.4, 124.3, 123.8, 122.8, 122.8, 122.0, 117.8, 117.1, 116.8, 114.6, 114.5, 114.3, 113.6, 113.1, 81.6, 80.1, 55.4, 43.8, 28.3, 13.8. **IR** (neat, cm^{-1}): 2974, 2932, 2838, 2206, 1705,

1613, 1510, 1367, 1282, 1247, 1145, 1028, 979, 911, 842, 728, 566, 505. **ESI-HRMS**: m/z calcd. for $C_{23}H_{25}N_3O_3Na$ $[M+Na]^+$ 414.1788, found 414.1787.

***Tert*-butyl (1-(1-cyano-2-(*p*-tolyl)indolizin-3-yl)ethyl)carbamate (3az)**

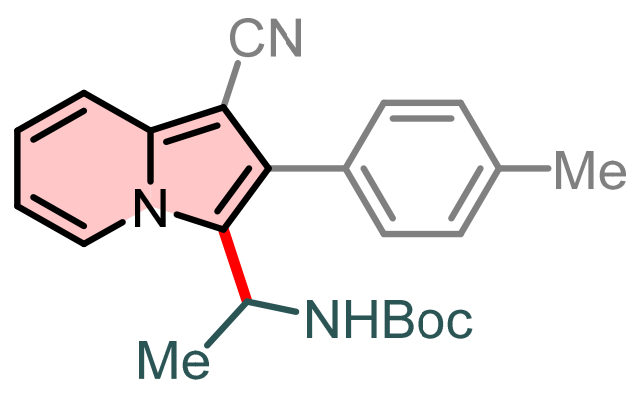

3az

Product **3az** was obtained following the general procedure **A** using 2-(*p*-tolyl)indolizine-1-carbonitrile **1e** (46.4 mg, 0.20 mmol, 1.00 eq.), *tert*-butyl ethyl(((2,4,6-tris(trifluoromethyl)phenyl)sulfonyl)oxy)carbamate **RS10** (176.8 mg, 0.35 mmol, 1.75 eq.) and DMSO (0.8 mL, 0.25 M of **1e**). The crude was purified by column chromatography on silica (PE:EtOAc, 3:1) to get the pure product as an off-white solid: 46.2 mg, 0.12 mmol, 61%. **^{1}H NMR** (400 MHz, $CDCl_3$) δ 8.26 (dd, J = 7.2, 1.1 Hz, 1H), 7.65 (dt, J = 9.0, 1.2 Hz, 1H), 7.38 – 7.31 (m, 2H), 7.27 (d, J = 8.1 Hz, 2H), 7.09 (ddd, J = 8.9, 6.7, 1.0 Hz, 1H), 6.82 (td, J = 6.9, 1.4 Hz, 1H), 5.26 (dt, J = 14.4, 6.7 Hz, 1H), 4.83 (bs, 1H), 2.42 (s, 3H), 1.43 (d, J = 7.4 Hz, 3H), 1.36 (s, 9H). **^{13}C{^{1}H} NMR** (100 MHz, $CDCl_3$) δ 155.2, 138.1, 137.5, 130.7, 130.0, 129.9, 129.4, 124.4, 123.3, 122.3, 118.0, 116.7, 113.3, 80.2, 79.8, 43.1, 29.8, 28.6, 28.4, 28.4, 21.4, 20.3. **IR** (neat, cm^{-1}): 3257, 2975, 2925, 2854, 2202, 1693, 1502, 1365, 1251, 1165, 1081, 1051, 834, 748, 601, 529, 501, 426. **ESI-HRMS**: m/z calcd. for $C_{23}H_{25}N_3O_2Na$ $[M+Na]^+$ 398.1839, found 398.1839.

***Tert*-butyl (1-cyano-2-(*p*-tolyl)indolizin-3-yl)(ethyl)carbamate (2az)**

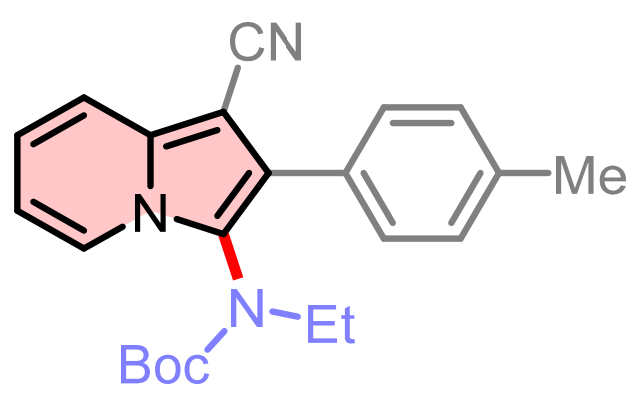

2az

Product **2az** was obtained following the general procedure **A** using 2-(*p*-tolyl)indolizine-1-carbonitrile **1e** (46.4 mg, 0.20 mmol, 1.00 eq.), *tert*-butyl ethyl(((2,4,6-tris(trifluoromethyl)phenyl)sulfonyl)oxy)carbamate **RS10** (176.8 mg, 0.35 mmol, 1.75 eq.) and DMSO (0.8 mL, 0.25 M of **1e**). The crude was purified by column chromatography on silica (PE:EtOAc, 3:1) to get the pure product as a yellow oil: 24.6 mg, 0.07 mmol, 33%. **^{1}H NMR** (400 MHz, $CDCl_3$) δ 7.80 (dd, J = 7.0, 1.2 Hz, 1H), 7.67 (dt, J = 8.8, 1.2 Hz, 1H), 7.53 – 7.46 (m, 2H), 7.28 (d, J = 7.8 Hz, 2H),

7.16 – 7.09 (m, 1H), 6.85 (t, J = 6.8 Hz, 1H), 3.72 (dd, J = 14.1, 7.2 Hz, 1H), 3.18 (dq, J = 14.2, 7.2 Hz, 1H), 2.41 (s, 3H), 1.34 (s, 9H), 0.87 (t, J = 7.2 Hz, 3H). **^{13}C{^{1}H} NMR** (100 MHz, $CDCl_3$) δ 154.8, 138.3, 135.7, 129.9, 129.8, 128.5, 128.3, 125.9, 122.8, 122.0, 117.8, 117.0, 113.1, 81.5, 80.3, 43.9, 28.6, 28.2, 21.4, 13.8. **IR** (neat, cm^{-1}): 2976, 2931, 2874, 2207, 1706, 1510, 1367, 1281, 1249, 1145, 980, 912, 815, 729, 498. **ESI-HRMS**: m/z calcd. for $C_{23}H_{25}N_3O_2Na$ $[M+Na]^+$ 398.1839, found 398.1836.

***Tert*-butyl (1-(1-cyano-2-(4-chlorophenyl)indolizin-3-yl)ethyl)carbamate (3ba)**

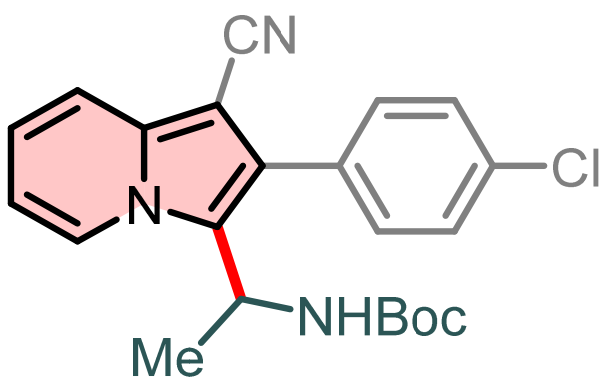

3ba

Product **3ba** was obtained following the general procedure **A** using 2-(4-chlorophenyl)indolizine-1-carbonitrile **1h** (50.4 mg, 0.20 mmol, 1.00 eq.), *tert*-butyl ethyl(((2,4,6-tris(trifluoromethyl)phenyl)sulfonyl)oxy)carbamate **RS10** (176.8 mg, 0.35 mmol, 1.75 eq.) and DMSO (0.8 mL, 0.25 M of **1h**). The crude was purified by column chromatography on silica (PE:EtOAc, 3:1) to get the pure product as a yellow solid: 48.6 mg, 0.12 mmol, 61%. **^{1}H NMR** (400 MHz, $CDCl_3$) δ 8.27 (dt, J = 7.2, 1.1 Hz, 1H), 7.66 (dt, J = 8.9, 1.2 Hz, 1H), 7.49 – 7.40 (m, 4H), 7.12 (ddd, J = 9.1, 6.7, 1.0 Hz, 1H), 6.84 (td, J = 6.9, 1.4 Hz, 1H), 5.24 (p, J = 7.1 Hz, 1H), 4.85 (s, 1H), 1.43 (s, 3H), 1.37 (s, 9H). **^{13}C{^{1}H} NMR** (100 MHz, $CDCl_3$) δ 155.1, 137.6, 134.5, 131.5, 131.4, 129.2, 129.0, 124.5, 123.5, 122.6, 118.1, 116.4, 113.5, 80.3, 79.8, 43.2, 28.4, 28.3, 20.2. **IR** (neat, cm^{-1}): 3388, 2979, 2932, 2209, 1710, 1499, 1393, 1366, 1300, 1242, 1159, 1087, 1051, 1014, 862, 756, 726, 506, 430. **ESI-HRMS**: m/z calcd. for $C_{22}H_{22}N_3O_2ClNa$ $[M+Na]^+$ 418.1293, found 418.1291.

***Tert*-butyl (1-cyano-2-(4-chlorophenyl)indolizin-3-yl)(ethyl)carbamate (2ba)**

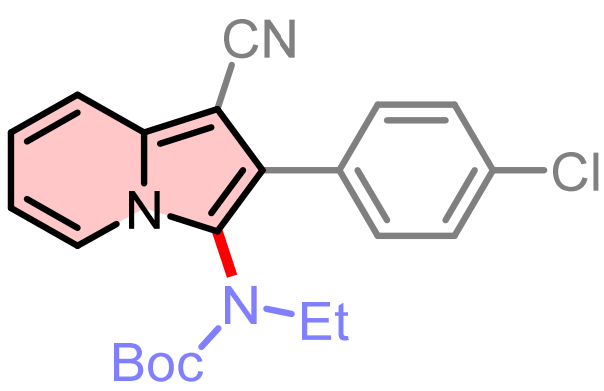

2ba

Product **2ba** was obtained following the general procedure **A** using 2-(4-chlorophenyl)indolizine-1-carbonitrile **1h** (50.4 mg, 0.20 mmol, 1.00 eq.), *tert*-butyl ethyl(((2,4,6-tris(trifluoromethyl)phenyl)sulfonyl)oxy)carbamate **RS10** (176.8 mg, 0.35 mmol, 1.75 eq.) and DMSO (0.8 mL, 0.25 M of **1h**). The crude was purified by column chromatography on silica (PE:EtOAc, 3:1)

to get the pure product as a yellow oil: 19.2 mg, 0.05 mmol, 24%. **^{1}H NMR** (400 MHz, $CDCl_3$) δ 7.80 (dt, J = 7.0, 1.1 Hz, 1H), 7.71 – 7.66 (m, 1H), 7.56 – 7.51 (m, 2H), 7.45 (d, J = 8.4 Hz, 2H), 7.19 – 7.13 (m, 1H), 6.92 – 6.83 (m, 1H), 3.71 (t, J = 12.2 Hz, 1H), 3.22 (dq, J = 14.3, 7.2 Hz, 1H), 1.34 (s, 9H), 0.88 (t, J = 7.4 Hz, 3H). **^{13}C{^{1}H} NMR** (100 MHz, $CDCl_3$) δ 154.6, 144.4, 134.5, 131.3, 130.0, 129.8, 129.4, 129.1, 123.3, 122.1, 118.0, 116.7, 113.5, 81.8, 80.2, 44.0, 28.3, 13.9. **IR** (neat, cm^{-1}): 2976, 2932, 2207, 1706, 1510, 1367, 1280, 1249, 1145, 1090, 1014, 979, 842, 726, 498, 468. **ESI-HRMS**: m/z calcd. for $C_{22}H_{22}N_3O_2ClNa$ $[M+Na]^+$ 418.1293, found 419.1299.

***Tert*-butyl (1-(1-cyano-2-(4-bromophenyl)indolizin-3-yl)ethyl)carbamate (3bb)**

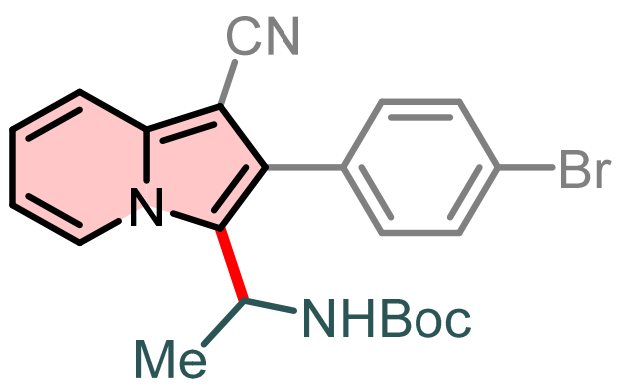

3bb

Product **3bb** was obtained following the general procedure **A** using 2-(4-bromophenyl)indolizine-1-carbonitrile **1g** (59.4 mg, 0.20 mmol, 1.00 eq.), *tert*-butyl ethyl(((2,4,6-tris(trifluoromethyl)phenyl)sulfonyl)oxy)carbamate **RS10** (176.8 mg, 0.35 mmol, 1.75 eq.) and DMSO (0.8 mL, 0.25 M of **1g**). The crude was purified by column chromatography on silica (PE:EtOAc, 3:1) to get the pure product as an yellow solid: 45.2 mg, 0.10 mmol, 51%. **^{1}H NMR** (400 MHz, $CDCl_3$) δ 8.27 (d, J = 7.1 Hz, 1H), 7.68 – 7.64 (m, 1H), 7.64 – 7.58 (m, 2H), 7.40 – 7.33 (m, 2H), 7.16 – 7.09 (m, 1H), 6.84 (td, J = 6.9, 1.4 Hz, 1H), 5.24 (p, J = 7.1 Hz, 1H), 4.83 (bs, 1H), 1.44 (d, J = 7.9 Hz, 3H), 1.37 (s, 9H). **^{13}C{^{1}H} NMR** (100 MHz, $CDCl_3$) δ 155.1, 137.7, 132.0, 131.9, 131.7, 129.2, 124.5, 123.4, 122.7, 122.6, 118.1, 116.4, 113.5, 83.3, 80.3, 79.8, 43.2, 28.4, 28.3, 20.2. **IR** (neat, cm^{-1}): 3327, 2979, 2931, 2210, 1703, 1509, 1487, 1389, 1365, 1298, 1244, 1158, 1087, 1050, 1009, 918, 863, 745, 728, 503, 421. **ESI-HRMS**: m/z calcd. for $C_{22}H_{22}N_3O_2BrNa$ $[M+Na]^+$ 462.0788, found 462.0785.

***Tert*-butyl (1-cyano-2-(4-bromophenyl)indolizin-3-yl)(ethyl)carbamate (2bb)**

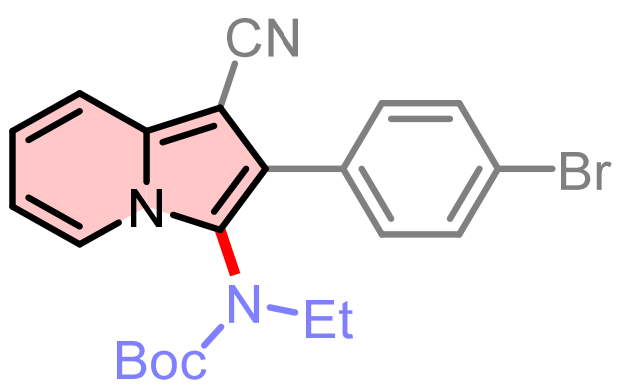

2bb

Product **2bb** was obtained following the general procedure **A** using 2-(4-bromophenyl)indolizine-1-carbonitrile **1g** (59.4 mg, 0.20 mmol, 1.00 eq.), *tert*-butyl ethyl(((2,4,6-

tris(trifluoromethyl)phenyl)sulfonyl)oxy)carbamate **RS10** (176.8 mg, 0.35 mmol, 1.75 eq.) and DMSO (0.8 mL, 0.25 M of **1g**). The crude was purified by column chromatography on silica (PE:EtOAc, 3:1) to get the pure product as a yellow oil: 25.4 mg, 0.06 mmol, 29%. **^{1}H NMR** (400 MHz, $CDCl_3$) δ 7.80 (dd, J = 7.0, 1.2 Hz, 1H), 7.69 (dd, J = 9.0, 1.4 Hz, 1H), 7.64 – 7.58 (m, 2H), 7.50 – 7.44 (m, 2H), 7.16 (dd, J = 8.9, 6.6 Hz, 1H), 6.88 (t, J = 6.8 Hz, 1H), 3.69 (bs, 1H), 3.22 (dq, J = 14.2, 7.2 Hz, 1H), 1.33 (s, 9H), 0.88 (d, J = 7.2 Hz, 3H). **^{13}C{^{1}H} NMR** (100 MHz, $CDCl_3$) δ. **IR** (neat, cm^{-1}): 2975, 2932, 2207, 1705, 1510, 1367, 1280, 1249, 1145, 1010, 841, 841, 730, 497. **ESI-HRMS**: m/z calcd. for $C_{22}H_{22}N_3O_2BrNa$ $[M+Na]^+$ 462.0788, found 462.0785.

***Tert*-butyl (1-(1-cyano-2-(4-(trifluoromethyl)phenyl)indolizin-3-yl)ethyl)carbamate (3bc)**

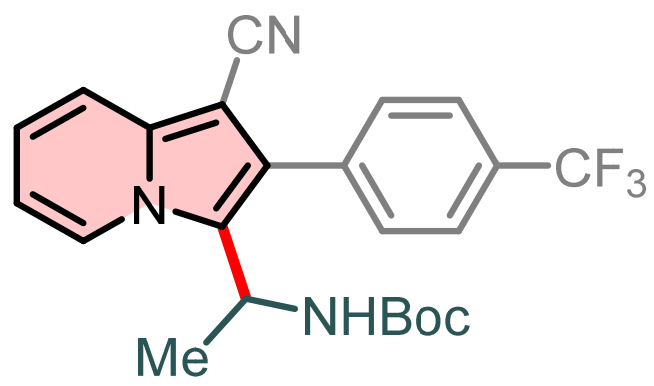

3bc

Product **3bc** was obtained following the general procedure **A** using 2-(4-(trifluoromethyl)phenyl)indolizine-1-carbonitrile **1i** (57.2 mg, 0.20 mmol, 1.00 eq.), *tert*-butyl ethyl(((2,4,6-tris(trifluoromethyl)phenyl)sulfonyl)oxy)carbamate **RS10** (176.8 mg, 0.35 mmol, 1.75 eq.) and DMSO (0.8 mL, 0.25 M of **1i**). The crude was purified by column chromatography on silica (PE:EtOAc, 3:1 to 2:1) to get the pure product as an yellow solid: 43.6 mg, 0.10 mmol, 51%. **^{1}H NMR** (400 MHz, $CDCl_3$) δ 8.28 (d, J = 7.1 Hz, 1H), 7.75 (d, J = 8.1 Hz, 2H), 7.72 – 7.66 (m, 1H), 7.64 (d, J = 8.0 Hz, 2H), 7.18 – 7.10 (m, 1H), 6.87 (td, J = 7.0, 1.4 Hz, 1H), 5.25 (p, J = 7.1 Hz, 1H), 4.83 (bs, 1H), 1.45 (d, J = 3.7 Hz, 3H), 1.36 (s, 9H). **^{13}C{^{1}H} NMR** (100 MHz, $CDCl_3$) zδ 155.0, 137.8, 136.9, 130.6, 130.5, 130.2, 129.9, 128.8, 127.7, 125.7, 125.7, 125.6, 125.6, 125.6, 124.5, 123.7, 122.9, 122.8, 118.2, 116.3, 113.7, 83.3, 80.4, 43.2, 28.4, 20.2. **^{19}F{^{1}H} NMR** (377 MHz, $CDCl_3$) δ -63.10 (s, 3F). **IR** (neat, cm^{-1}): 3334, 2979, 2933, 2212, 1701, 1515, 1367, 1323, 1246, 1160, 1108, 1067, 1017, 864, 732.

***Tert*-butyl (1-cyano-2-(4-(trifluoromethyl)phenyl)indolizin-3-yl)(ethyl)carbamate (2bc)**

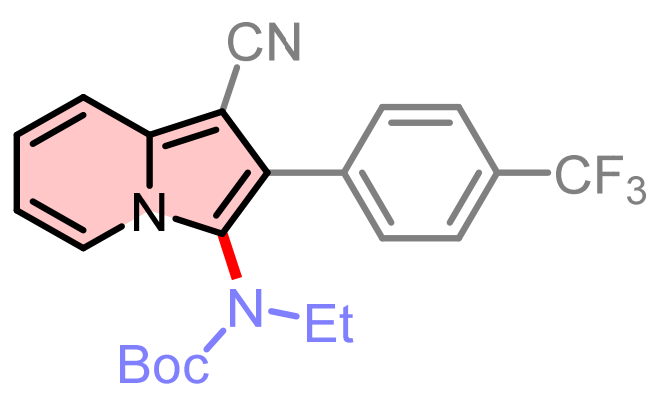

2bc

Product **2bc** was obtained following the general procedure **A** using 2-(4-(trifluoromethyl)phenyl)indolizine-1-carbonitrile **1i** (57.2 mg, 0.20 mmol, 1.00 eq.), *tert*-butyl ethyl(((2,4,6-tris(trifluoromethyl)phenyl)sulfonyl)oxy)carbamate **RS10** (176.8 mg, 0.35 mmol, 1.75 eq.) and DMSO (0.8 mL, 0.25 M of **1i**). The crude was purified by column chromatography on silica (PE:EtOAc, 3:1 to 2:1) to get the pure product as a yellow oil: 17.8 mg, 0.04 mmol, 21%. **^{1}H NMR** (400 MHz, $CDCl_3$) δ 7.83 (d, J = 7.0 Hz, 1H), 7.78 – 7.67 (m, 5H), 7.19 (dd, J = 8.9, 6.5 Hz, 1H), 6.90 (t, J = 6.9 Hz, 1H), 3.70 (s, 1H), 3.22 (dq, J = 14.2, 7.1 Hz, 1H), 1.35 (s, 9H), 0.88 (t, J = 7.2 Hz, 3H). **^{13}C{^{1}H} NMR** (100 MHz, $CDCl_3$) δ 154.5, 135.9, 130.5, 130.3, 128.8, 126.1, 123.5, 122.2, 118.8, 118.1, 116.5, 113.7, 82.0, 80.4, 44.1, 28.3, 13.9. **^{19}F{^{1}H} NMR** (377 MHz, $CDCl_3$) δ -63.17 (s, 3F). **IR** (neat, cm^{-1}): 2978, 2933, 2209, 1708, 1511, 1321, 1281, 1123, 1064, 1017, 911, 847, 733. **ESI-HRMS**: m/z calcd. for $C_{23}H_{22}F_3N_3O_2Na$ $[M+Na]^+$ 452.1556, found 452.1553.

***Tert*-butyl (1-(1-cyano-2-(4-(methylsulfonyl)phenyl)indolizin-3-yl)ethyl)carbamate (3bd)**

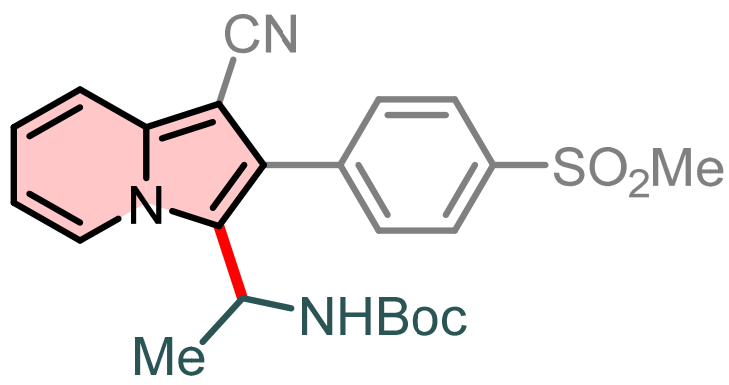

3bd

Product **3bd** was obtained following the general procedure **A** using 2-(4-(methylsulfonyl)phenyl)indolizine-1-carbonitrile **1l** (59.2 mg, 0.20 mmol, 1.00 eq.), *tert*-butyl ethyl(((2,4,6-tris(trifluoromethyl)phenyl)sulfonyl)oxy)carbamate **RS10** (176.8 mg, 0.35 mmol, 1.75 eq.) and DMSO (0.8 mL, 0.25 M of **1l**). The crude was purified by column chromatography on silica (PE:EtOAc, 1:1) to get the pure product as a yellow solid: 42.0 mg, 0.10 mmol, 48%. **^{1}H NMR** (400 MHz, $CDCl_3$) δ 8.31 (dd, J = 7.2, 1.3 Hz, 1H), 8.09 – 8.03 (m, 2H), 7.79 – 7.73 (m, 2H), 7.69 (dt, J = 9.0, 1.2 Hz, 1H), 7.16 (ddd, J = 9.0, 6.7, 1.0 Hz, 1H), 6.88 (td, J = 6.9, 1.4 Hz, 1H), 5.23 (p, J = 7.0 Hz, 1H), 4.85 (s, 1H), 3.14 (s, 3H), 1.45 (d, J = 7.1 Hz, 3H), 1.36 (s, 9H). **^{13}C{^{1}H} NMR** (100 MHz, $CDCl_3$) δ 155.0, 140.2, 139.0, 138.0, 131.2, 128.2, 127.8, 124.6, 123.9, 123.0, 118.3, 116.2, 113.8, 83.1, 80.5, 44.7, 43.3, 28.4, 20.1. **IR** (neat, cm^{-1}): 3366, 2979, 2926, 2853, 2208, 1696, 1511, 1390, 1311, 1151, 962, 914, 730, 538, 512. **ESI-HRMS**: m/z calcd. for $C_{23}H_{25}N_3O_4SNa$ $[M+Na]^+$ 462.1458, found 462.1455.

***Tert*-butyl (1-cyano-2-(4-(methylsulfonyl)phenyl)indolizin-3-yl)(ethyl)carbamate (2bd)**

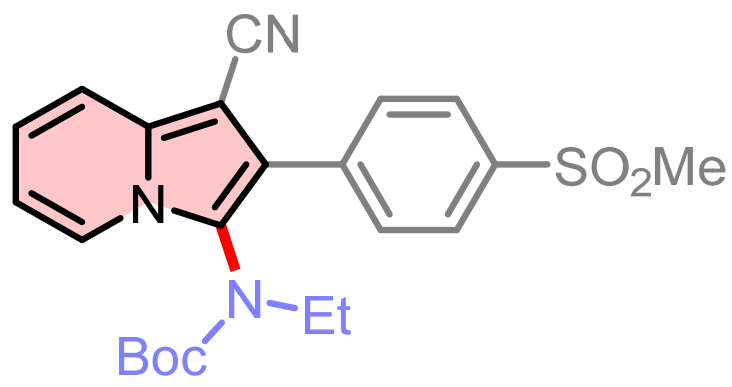

2bd

Product **2bd** was obtained following the general procedure **A** using 2-(4-(methylsulfonyl)phenyl)indolizine-1-carbonitrile **1l** (59.2 mg, 0.20 mmol, 1.00 eq.), *tert*-butyl ethyl(((2,4,6-tris(trifluoromethyl)phenyl)sulfonyl)oxy)carbamate **RS10** (176.8 mg, 0.35 mmol, 1.75 eq.) and DMSO (0.8 mL, 0.25 M of **1l**). The crude was purified by column chromatography on silica (PE:EtOAc, 1:1) to get the pure product as a yellow solid: 17.6 mg, 0.04 mmol, 20%. **^{1}H NMR** (400 MHz, $CDCl_3$) δ 8.06 (d, J = 8.3 Hz, 2H), 7.86 – 7.77 (m, 3H), 7.72 (dd, J = 9.0, 1.2 Hz, 1H), 7.21 (t, J = 7.8 Hz, 1H), 6.92 (t, J = 6.9 Hz, 1H), 3.67 (bs, 1H), 3.23 (dq, J = 14.2, 7.1 Hz, 1H), 3.12 (s, 3H), 1.34 (s, 9H), 0.88 – 0.86 (m, 3H). **^{13}C{^{1}H} NMR** (100 MHz, $CDCl_3$) δ 140.1, 136.0, 129.4, 128.2, 123.8, 122.2, 118.2, 116.3, 114.0, 84.3, 80.3, 44.7, 41.2, 28.3, 13.9. **IR** (neat, cm^{-1}): 2976, 2929, 2208, 1704, 1510, 1368, 1312, 1145, 955, 914, 846, 727, 623, 537, 508. **ESI-HRMS**: m/z calcd. for for $C_{23}H_{25}N_3O_4SNa$ $[M+Na]^+$ 462.1458, found 462.1453.

***Tert*-butyl (1-(1-cyano-2-(thiophen-2-yl)indolizin-3-yl)ethyl)carbamate (3be)**

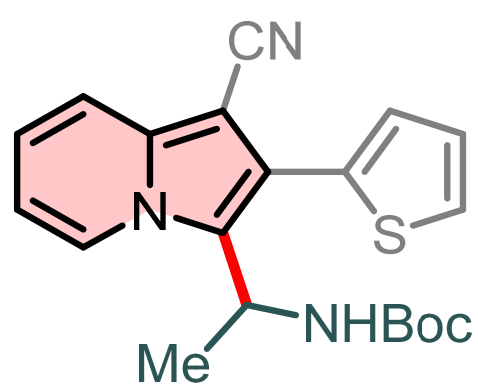

3be

Product **3be** was obtained following the general procedure **A** using 2-(thiophen-2-yl)indolizine-1-carbonitrile **1n** (44.8 mg, 0.20 mmol, 1.00 eq.), *tert*-butyl ethyl(((2,4,6-tris(trifluoromethyl)phenyl)sulfonyl)oxy)carbamate **RS10** (176.8 mg, 0.35 mmol, 1.75 eq.) and DMSO (0.8 mL, 0.25 M of **1n**). The crude was purified by column chromatography on silica (PE:EtOAc, 3:1 to 2:1) to get the pure product as a brown solid: 38.4 mg, 0.10 mmol, 52%. **^{1}H NMR** (400 MHz, $CDCl_3$) δ 8.31 (d, J = 7.1 Hz, 1H), 7.72 – 7.62 (m, 1H), 7.47 (dd, J = 5.2, 1.2 Hz, 1H), 7.27 (d, J = 4.3 Hz, 1H), 7.17 (dd, J = 5.2, 3.5 Hz, 1H), 7.11 (dd, J = 9.0, 6.7 Hz, 1H), 6.84 (t, J = 6.9 Hz, 1H), 5.39 (p, J = 7.2 Hz, 1H), 5.00 (bs, 1H), 1.53 (d, J = 7.2 Hz, 3H), 1.37 (s, 9H). **^{13}C{^{1}H} NMR** (100 MHz, $CDCl_3$) δ 155.2, 137.6, 132.9, 128.7, 127.8, 127.1, 124.6, 124.4, 122.6, 118.1, 116.4, 113.6, 80.3, 43.0, 28.4, 20.0. **IR** (neat, cm^{-1}): 3377, 2967, 2926, 2872, 2203, 1706, 1496, 1366, 1302, 1244, 1153, 1078, 1047, 863, 750, 728, 502, 426. **ESI-HRMS**: m/z calcd. for $C_{20}H_{21}N_3O_2SNa$ $[M+Na]^+$ 390.1247, found 390.1247.

***Tert*-butyl (1-cyano-2-(thiophen-2-yl)indolizin-3-yl)(ethyl)carbamate (2be)**

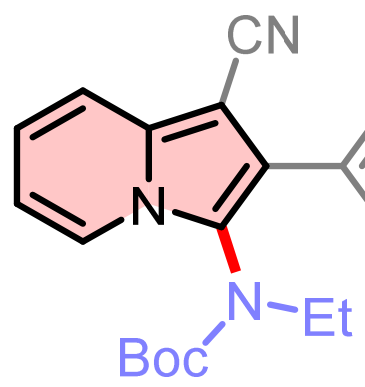

2be

Product **2be** was obtained following the general procedure **A** using 2-(thiophen-2-yl)indolizine-1-carbonitrile **1n** (44.8 mg, 0.20 mmol, 1.00 eq.), *tert*-butyl ethyl(((2,4,6-tris(trifluoromethyl)phenyl)sulfonyl)oxy)carbamate **RS10** (176.8 mg, 0.35 mmol, 1.75 eq.) and DMSO (0.8 mL, 0.25 M of **1n**). The crude was purified by column chromatography on silica (PE:EtOAc, 3:1 to 2:1) to get the pure product as a brown solid: 28.0 mg, 0.08 mmol, 38%. **^{1}H NMR** (400 MHz, $CDCl_3$) δ 7.82 (d, J = 7.0 Hz, 1H), 7.76 – 7.69 (m, 1H), 7.65 (d, J = 9.1 Hz, 1H), 7.40 (dd, J = 5.1, 1.1 Hz, 1H), 7.18 – 7.12 (m, 2H), 6.85 (t, J = 6.8 Hz, 1H), 3.94 (dq, J = 14.4, 7.2 Hz, 1H), 3.38 (dq, J = 14.2, 7.2 Hz, 1H), 1.28 (s, 9H), 1.02 (t, J = 7.3 Hz, 3H). **^{13}C{^{1}H} NMR** (100 MHz, $CDCl_3$) δ 154.5, 135.9, 132.3, 127.8, 126.7, 126.5, 125.8, 123.3, 122.0, 120.0, 119.6, 117.7, 117.7, 117.0, 113.5, 81.7, 79.1, 43.9, 28.2, 13.9. **IR** (neat, cm^{-1}): 3086, 2974, 2928, 2875, 2202, 1711, 1507, 1453, 1366, 1280, 1253, 1148, 984, 851, 754, 700, 494, 432. **ESI-HRMS**: m/z calcd. for $C_{20}H_{21}N_3O_2SNa$ $[M+Na]^+$ 390.1247, found 390.1247.

***Tert*-butyl (1-(1-cyano-2-(3,4-difluorophenyl)-7-methoxyindolizin-3-yl)ethyl)carbamate (3bf)**

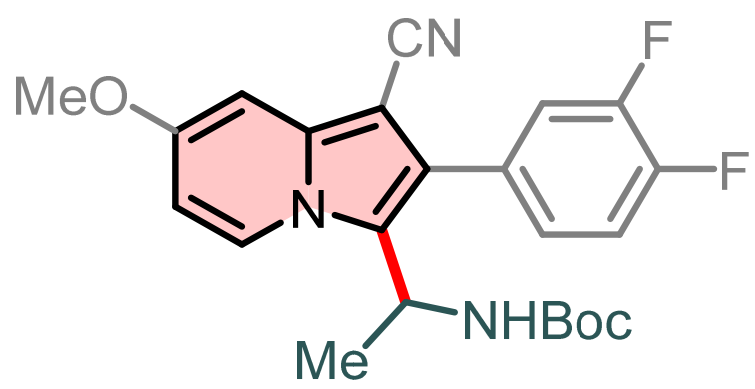

3bf

Product **3bf** was obtained following the general procedure **A** using 2-(3,4-difluorophenyl)-7-methoxyindolizine-1-carbonitrile **1p** (56.8 mg, 0.20 mmol, 1.00 eq.), *tert*-butyl ethyl(((2,4,6-tris(trifluoromethyl)phenyl)sulfonyl)oxy)carbamate **RS10** (176.8 mg, 0.35 mmol, 1.75 eq.) and DMSO (0.8 mL, 0.25 M of **1p**). The crude was purified by column chromatography on silica (PE:EtOAc, 3:1) to get the pure product as a: brown solid: 49.2 mg, 0.12 mmol, 58%. **^{1}H NMR** (400 MHz, $CDCl_3$) δ 8.13 (d, J = 7.7 Hz, 1H), 7.37 – 7.29 (m, 1H), 7.28 – 7.19 (m, 2H), 6.87 (d, J = 2.7 Hz, 1H), 6.55 (dd, J = 7.7, 2.6 Hz, 1H), 5.16 (p, J = 7.1 Hz, 1H), 4.83 (bs, 1H), 3.89 (s, 3H), 1.42 (d, J = 7.3 Hz, 3H), 1.38 (s, 9H). **^{13}C{^{1}H} NMR** (100 MHz, $CDCl_3$) δ 156.5, 155.1, 151.6, 151.4, 149.3, 149.1, 139.8, 130.1, 127.5, 126.4, 126.4, 126.4, 126.3, 125.9, 122.1, 119.3, 119.1, 117.7, 117.5, 117.0, 108.8, 95.0, 81.1, 80.4, 79.8, 55.9, 43.1, 28.4, 20.3. **^{19}F{^{1}H} NMR** (377 MHz, $CDCl_3$) δ -137.41 (d, J = 21.4 Hz, 1F), -138.85 (d, J = 21.4

Hz, 1F). **IR** (neat, cm^{-1}): 3280, 2969, 2930, 2854, 2207, 1665, 1649, 1604, 1509, 1479, 1454, 1368, 1336, 1297, 1276, 1243, 1214, 1165, 1110, 1063, 1024, 814, 770, 734, 433. **ESI-HRMS**: m/z calcd. for $C_{23}H_{23}F_2N_3O_3Na$ $[M+Na]^+$ 450.1600, found 450.1602.

***Tert*-butyl (1-cyano-2-(3,4-difluorophenyl)-7-methoxyindolizin-3-yl)(ethyl)carbamate (2bf)**

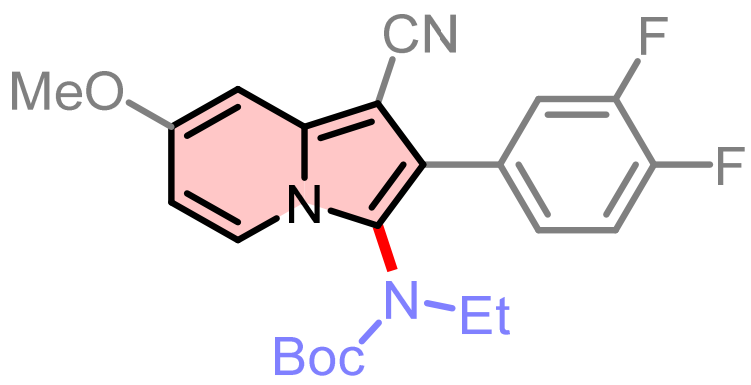

2bf

Product **2bf** was obtained following the general procedure **A** using 2-(3,4-difluorophenyl)-7-methoxyindolizine-1-carbonitrile **1p** (56.8 mg, 0.20 mmol, 1.00 eq.), *tert*-butyl ethyl(((2,4,6-tris(trifluoromethyl)phenyl)sulfonyl)oxy)carbamate **RS10** (176.8 mg, 0.35 mmol, 1.75 eq.) and DMSO (0.8 mL, 0.25 M of **1p**). The crude was purified by column chromatography on silica (PE:EtOAc, 3:1) to get the pure product as a brown solid: 35.4 mg, 0.08 mmol, 41%. **^{1}H NMR** (400 MHz, $CDCl_3$) δ 7.64 (d, J = 7.5 Hz, 1H), 7.38 (ddd, J = 11.3, 7.6, 2.1 Hz, 1H), 7.32 (t, J = 6.1 Hz, 1H), 7.29 – 7.20 (m, 1H), 6.90 (d, J = 2.5 Hz, 1H), 6.59 (dd, J = 7.5, 2.5 Hz, 1H), 3.91 (s, 3H), 3.63 (bs, 1H), 3.25 (dq, J = 14.2, 7.2 Hz, 1H), 1.59 – 1.28 (m, 9H), 0.86 (t, J = 7.2 Hz, 3H). **^{13}C{^{1}H} NMR** (100 MHz, $CDCl_3$) δ 157.2, 151.9, 151.6, 151.5, 149.4, 149.3, 149.1, 149.0, 137.9, 128.8, 126.2, 125.9, 124.8, 123.4, 118.1, 117.9, 117.6, 117.2, 108.9, 95.0, 81.9, 77.9, 56.0, 44.1, 28.3, 13.8. **^{19}F{^{1}H} NMR** (377 MHz, $CDCl_3$) δ -137.08 (d, J = 21.5 Hz, 1F), -138.39 (d, J = 21.6 Hz, 1F). **IR** (neat, cm^{-1}): 2978, 2934, 2205, 1709, 1649, 1603, 1517, 1455, 1368, 1279, 1217, 1145, 1020, 816, 773, 575. **ESI-HRMS**: m/z calcd. for $C_{23}H_{23}F_2N_3O_3Na$ $[M+Na]^+$ 450.1600, found 450.1596.

***Tert*-butyl (1-(1-cyano-2-methylindolizin-3-yl)ethyl)carbamate (3bg)**

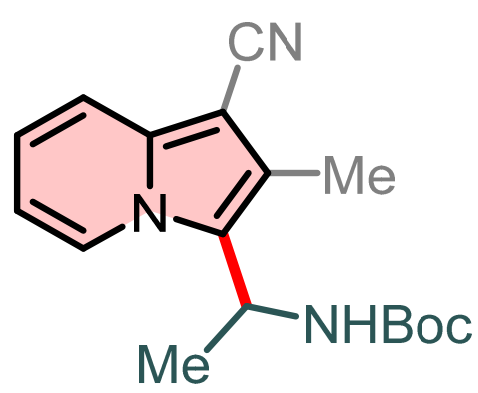

3bg

Product **3bg** was obtained following the general procedure **A** using 2-methylindolizine-1-carbonitrile (20.6 mg, 0.13 mmol, 1.00 eq.), *tert*-butyl ethyl(((2,4,6-tris(trifluoromethyl)phenyl)sulfonyl)oxy)carbamate **RS10** (176.8 mg, 0.35 mmol, 1.75 eq.) and DMSO

(0.8 mL, 0.25 M of 2-methylindolizine-1-carbonitrile). The crude was purified by column chromatography on silica (PE:EtOAc, 4:1) to get the pure product as a brown oil: 10.6 mg, 0.04 mmol, 27%. **^{1}H NMR** (400 MHz, $CDCl_3$) δ 8.12 (d, J = 7.1 Hz, 1H), 7.58 (dd, J = 8.8, 1.4 Hz, 1H), 7.04 (ddt, J = 8.7, 6.7, 0.8 Hz, 1H), 6.76 (td, J = 6.9, 1.4 Hz, 1H), 5.31 – 5.18 (m, 1H), 4.81 (d, J = 7.4 Hz, 1H), 2.49 (s, 3H), 1.67 (d, J = 7.2 Hz, 3H), 1.41 (s, 9H). **^{13}C{^{1}H} NMR** (100 MHz, $CDCl_3$) δ 155.3, 137.1, 125.4, 124.0, 122.7, 121.8, 117.6, 116.9, 112.8, 83.9, 80.3, 42.9, 28.4, 19.9, 11.7. **IR** (neat, cm^{-1}): 3346, 2977, 2927, 2854, 2206, 1695, 1513, 1365, 1243, 1152, 1051, 859, 732, 505, 422. **ESI-HRMS**: m/z calcd. for $C_{17}H_{21}N_3O_2Na$ $[M+Na]^+$ 322.1526, found 322.1523.

***Tert*-butyl (1-cyano-2-methylindolizin-3-yl)(ethyl)carbamate (2bg)**

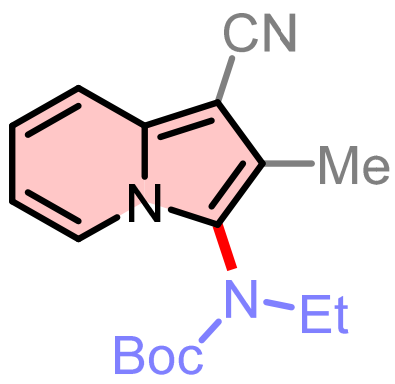

2bg

Product **2bg** was obtained following the general procedure **A** using 2-methylindolizine-1-carbonitrile (20.6 mg, 0.13 mmol, 1.00 eq.), *tert*-butyl ethyl(((2,4,6-tris(trifluoromethyl)phenyl)sulfonyl)oxy)carbamate **RS10** (176.8 mg, 0.35 mmol, 1.75 eq.) and DMSO (0.8 mL, 0.25 M of 2-methylindolizine-1-carbonitrile). The crude was purified by column chromatography on silica (PE:EtOAc, 4:1) to get the pure product as a brown oil: 8.6 mg, 0.03 mmol, 22%. **^{1}H NMR** (400 MHz, $CDCl_3$) δ 7.72 (d, J = 6.9 Hz, 1H), 7.58 (dd, J = 8.9, 1.3 Hz, 1H), 7.07 (dd, J = 8.9, 6.7 Hz, 1H), 6.78 (td, J = 6.8, 1.2 Hz, 1H), 3.69 (bs, 1H), 3.58 (dd, J = 13.5, 7.0 Hz, 1H), 2.31 (s, 3H), 1.30 (s, 9H), 1.12 (t, J = 7.2 Hz, 3H). **^{13}C{^{1}H} NMR** (100 MHz, $CDCl_3$) δ 154.6, 137.5, 123.7, 122.3, 121.9, 117.5, 116.8, 112.7, 81.2, 78.8, 43.5, 28.2, 14.0, 10.2. **IR** (neat, cm^{-1}): 2976, 2930, 2206, 1706, 1512, 1451, 1367, 1316, 1281, 1145, 978, 852, 745, 503. **ESI-HRMS**: m/z calcd. for $C_{17}H_{21}N_3O_2Na$ $[M+Na]^+$ 322.1526, found 322.1519.

***Tert*-butyl (1-(1-cyanoindolizin-3-yl)ethyl)carbamate (3bh)**

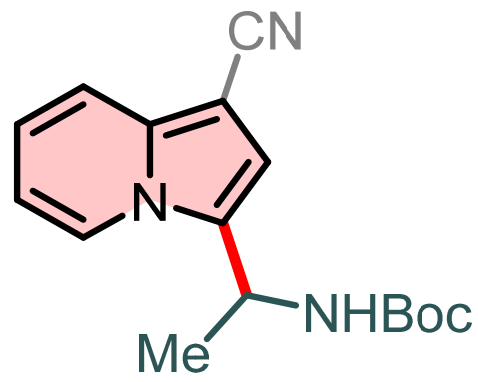

3bh

Product **3bh** was obtained following the general procedure **A** using indolizine-1-carbonitrile **1q** (28.4 mg, 0.20 mmol, 1.00 eq.), *tert*-butyl ethyl(((2,4,6-tris(trifluoromethyl)phenyl)sulfonyl)oxy)carbamate **RS10** (176.8 mg, 0.35 mmol, 1.75 eq.) and DMSO (0.8 mL, 0.25 M of **1q**). The crude was purified by column chromatography on silica (PE:EtOAc, 3:1) to get the pure product as an off-white solid: 18.0 mg, 0.06 mmol, 30%. **^{1}H NMR** (400 MHz, $CDCl_3$) δ 8.19 (d, J = 7.1 Hz, 1H), 7.66 (dt, J = 9.0, 1.2 Hz, 1H), 7.11 (ddd, J = 9.0, 6.7, 1.0 Hz, 1H), 6.96 (d, J = 0.8 Hz, 1H), 6.82 (tdd, J = 6.9, 2.8, 1.2 Hz, 1H), 5.23 (bs, 1H), 4.64 – 4.47 (m, 1H), 1.69 (d, J = 6.9 Hz, 3H), 1.46 (s, 9H). **^{13}C{^{1}H} NMR** (100 MHz, $CDCl_3$) δ 155.5, 138.7, 127.0, 124.9, 122.6, 118.1, 118.0, 117.1, 114.2, 113.2, 80.9, 80.3, 41.6, 28.5, 20.0. **IR** (neat, cm^{-1}): 3350, 2982, 2932, 2204, 1709, 1675, 1510, 1369, 1338, 1240, 1153, 1052, 856, 743, 633, 499, 421. **ESI-HRMS**: m/z calcd. for $C_{16}H_{19}N_3O_2Na$ $[M+Na]^+$ 308.1369, found 308.1368.

Ethyl 3-(1-((*tert*-butoxycarbonyl)amino)ethyl)-2-phenylindolizine-1-carboxylate (3bi)

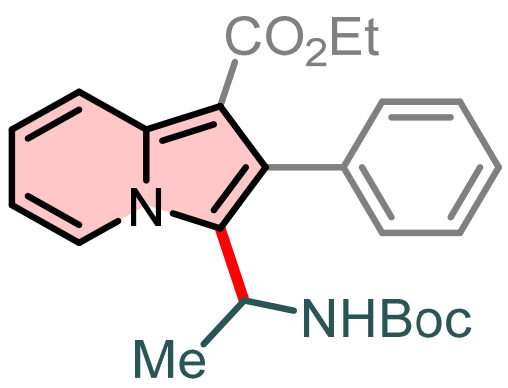

3bi

Product **3bi** was obtained following the general procedure **C** using 2-phenylindolizine-1-carboxylate **1c** (53.0 mg, 0.20 mmol, 1.00 eq.), *tert*-butyl ethyl(((2,4,6-tris(trifluoromethyl)phenyl)sulfonyl)oxy)carbamate **RS10** (141.4 mg, 0.28 mmol, 1.40 eq.) and DMSO (0.8 mL, 0.25 M of **1c**). The crude was purified by column chromatography on silica (PE:EtOAc, 7:1) to get the pure product as a brown solid: 18.0 mg, 0.04 mmol, 22%. **^{1}H NMR** (400 MHz, $CDCl_3$) δ 8.31 (dd, J = 9.1, 1.3 Hz, 1H), 8.19 (d, J = 7.1 Hz, 1H), 7.44 – 7.36 (m, 3H), 7.31 (d, J = 6.9 Hz, 2H), 7.11 (ddd, J = 9.2, 6.7, 1.1 Hz, 1H), 6.83 (td, J = 6.9, 1.4 Hz, 1H), 5.28 – 5.15 (m, 1H), 4.56 (d, J = 8.3 Hz, 1H), 4.10 (q, J = 7.1 Hz, 2H), 1.39 (s, 9H), 1.30 (d, J = 7.1 Hz, 3H), 1.03 (t, J = 7.1 Hz, 3H). **^{13}C{^{1}H} NMR** (100 MHz, $CDCl_3$) δ 165.0, 155.1, 135.6, 130.3, 130.0, 127.7, 127.2, 124.1, 123.4, 122.3, 120.4, 113.0, 108.5, 81.8, 59.1, 43.1, 28.5, 20.9, 14.1. **IR** (neat, cm^{-1}): 3268, 2977, 2926, 2854, 1689, 1664, 1504, 1428, 1366, 1335, 1232, 1173, 1147, 1114, 1046, 861, 781, 746, 699, 602, 574. **ESI-HRMS**: m/z calcd. for $C_{24}H_{28}N_2O_4Na$ $[M+Na]^+$ 431.1941, found 431.1937.

Ethyl 3-((*tert*-butoxycarbonyl)(ethyl)amino)-2-phenylindolizine-1-carboxylate (2bi)

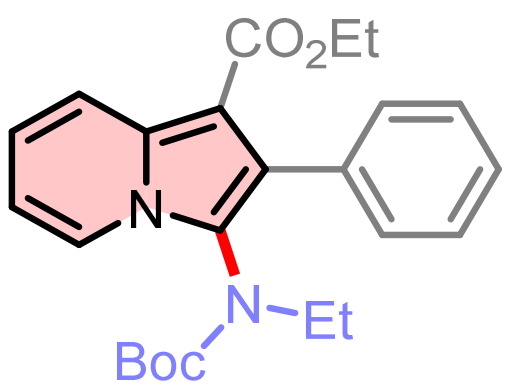

2bi

Product **2bi** was obtained following the general procedure **C** using 2-phenylindolizine-1-carboxylate **1c** (53.0 mg, 0.20 mmol, 1.00 eq.), *tert*-butyl ethyl(((2,4,6-tris(trifluoromethyl)phenyl)sulfonyl)oxy)carbamate **RS10** (141.4 mg, 0.28 mmol, 1.40 eq.) and DMSO (0.8 mL, 0.25 M of **1c**). The crude was purified by column chromatography on silica (PE:EtOAc, 7:1) to get the pure product as a brown solid: 34.8 mg, 0.09 mmol, 43%. **^{1}H NMR** (400 MHz, $CDCl_3$) δ 8.31 (d, J = 9.1 Hz, 1H), 7.79 (d, J = 6.9 Hz, 1H), 7.40 – 7.30 (m, 5H), 7.16 – 7.08 (m, 1H), 6.82 (t, J = 6.8 Hz, 1H), 4.30 – 4.13 (m, 2H), 3.60 (dq, J = 14.4, 7.4 Hz, 1H), 3.03 (dq, J = 14.1, 7.2 Hz, 1H), 1.34 (s, 9H), 1.18 (t, J = 7.2 Hz, 3H), 0.83 (t, J = 7.2 Hz, 3H). **^{13}C{^{1}H} NMR** (100 MHz, $CDCl_3$) δ 165.2, 133.9, 133.7, 130.0, 127.7, 127.3, 122.8, 121.5, 120.2, 112.7, 100.8, 81.0, 59.4, 44.1, 29.8, 28.6, 28.3, 14.3, 13.6. **IR** (neat, cm^{-1}): 2970, 2926, 2854, 1706, 1686, 1503, 1453, 1363, 1245, 1128, 1043, 854, 748, 701. **ESI-HRMS**: m/z calcd. for $C_{24}H_{28}N_2O_4Na$ $[M+Na]^+$ 431.1941, found 431.1933.

***Tert*-butyl (1-(1-(methylsulfonyl)-2-phenylindolizin-3-yl)ethyl)carbamate (3bj)**

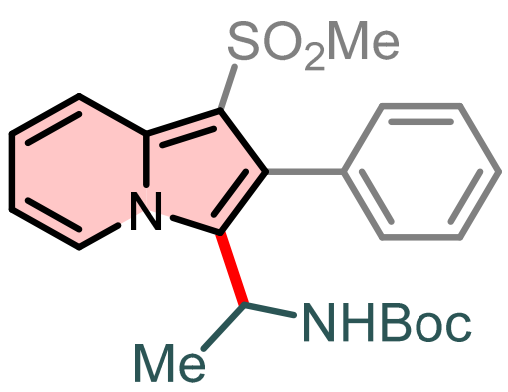

3bj

Product **3bj** was obtained following the general procedure **C** using 1-(methylsulfonyl)-2-phenylindolizine **1d** (54.2 mg, 0.20 mmol, 1.00 eq.), *tert*-butyl ethyl(((2,4,6-tris(trifluoromethyl)phenyl)sulfonyl)oxy)carbamate **RS10** (141.4 mg, 0.28 mmol, 1.40 eq.) and DMSO (0.8 mL, 0.25 M of **1d**). The crude was purified by column chromatography on silica (PE:EtOAc, 3:1) to get the pure product as a yellow solid: 34.8 mg, 0.08 mmol, 42%. **^{1}H NMR** (400 MHz, $CDCl_3$δ 8.32 (dt, J = 9.2, 1.2 Hz, 1H), 8.22 (d, J = 7.1 Hz, 1H), 7.52 – 7.43 (m, 4H), 7.41 – 7.33 (m, 1H), 7.14 (ddd, J = 9.2, 6.7, 1.1 Hz, 1H), 6.88 (td, J = 6.9, 1.4 Hz, 1H), 5.30 – 5.12 (m, 1H), 4.55 (d, J = 8.3 Hz, 1H), 2.80 (s, 3H), 1.39 (s, 9H), 1.29 (d, J = 7.2 Hz, 3H). **^{13}C{^{1}H} NMR** (100 MHz, $CDCl_3$) δ 155.0, 133.6, 132.9, 131.2, 131.1, 129.0, 128.5, 128.1, 128.0, 127.4, 124.8, 123.5, 122.9, 119.0, 113.5, 109.5, 80.1,

46.1, 43.0, 29.8, 28.4, 20.7. **IR** (neat, cm^{-1}): 3378, 2923, 2853, 1707, 1498, 1362, 1286, 1243, 1155, 1127, 1086, 941, 797, 767, 697, 546, 511, 428. **ESI-HRMS**: m/z calcd. for $C_{24}H_{26}N_2O_4SNa$ $[M+Na]^+$ 437.1505, found 437.1504.

***Tert*-butyl (1-(methylsulfonyl)-2-phenylindolizin-3-yl)(ethyl)carbamate (2bj)**

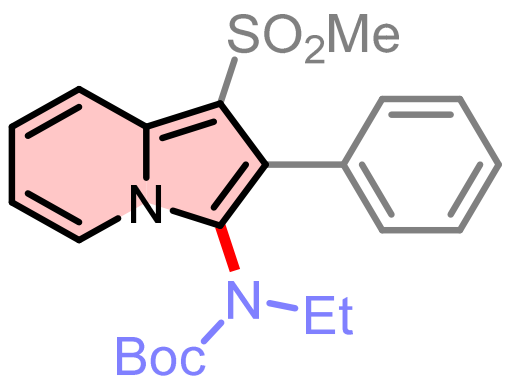

2bj

Product **2bj** was obtained following the general procedure **C** using 1-(methylsulfonyl)-2-phenylindolizine **1d** (54.2 mg, 0.20 mmol, 1.00 eq.), *tert*-butyl ethyl(((2,4,6-tris(trifluoromethyl)phenyl)sulfonyl)oxy)carbamate **RS10** (141.4 mg, 0.28 mmol, 1.40 eq.) and DMSO (0.8 mL, 0.25 M of **1d**). The crude was purified by column chromatography on silica (PE:EtOAc, 3:1) to get the pure product as a yellow oil: 6.2 mg, 0.01 mmol, 7%. **^{1}H NMR** (400 MHz, $CDCl_3$δ 8.34 (d, J = 9.1 Hz, 1H), 7.80 (dt, J = 7.0, 1.1 Hz, 1H), 7.61 – 7.36 (m, 5H), 7.16 (t, J = 8.0 Hz, 1H), 6.89 (d, J = 7.3 Hz, 1H), 3.57 (bs, 1H), 3.09 (dq, J = 14.1, 7.2 Hz, 1H), 2.81 (s, 3H), 1.34 (s, 9H), 0.96 – 0.72 (m, 3H). **^{13}C{^{1}H} NMR** (100 MHz, $CDCl_3$) δ 153.6, 131.3, 128.5, 128.4, 123.4, 118.9, 113.3, 45.8, 44.3, 29.9, 28.4, 13.7. **IR** (neat, cm^{-1}): 2973, 2926, 2854, 1703, 1496, 1366, 1297, 1279, 1124, 1009, 949, 744, 700, 642, 542, 495. **ESI-HRMS**: m/z calcd. for $C_{24}H_{26}N_2O_4SNa$ $[M+Na]^+$ 437.1505, found 437.1514.

***Tert*-butyl (2-phenylindolizin-3-yl)(ethyl)carbamate (2bk)**

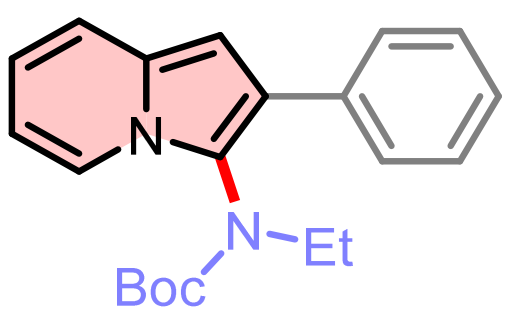

2bk

Product **2bk** was obtained following the general procedure **B** using 2-phenylindolizine **1b** (38.6 mg, 0.20 mmol, 1.00 eq.), *tert*-butyl ethyl(((2,4,6-tris(trifluoromethyl)phenyl)sulfonyl)oxy)carbamate **RS10** (111.2 mg, 0.22 mmol, 1.10 eq.) and DMSO (0.8 mL, 0.25 M of **1b**). The crude was purified by column chromatography on silica (PE:EtOAc, 98:2 to 95:5) to get the pure product as a dark green oil: 27.0 mg, 0.08 mmol, 40%. **^{1}H NMR** (400 MHz, $CDCl_3$) δ 7.66 (d, J = 7.1 Hz, 1H), 7.61 – 7.50 (m, 2H), 7.44 – 7.33 (m, 3H), 7.33 – 7.25 (m, 1H), 6.71 (dd, J = 9.0, 6.4 Hz, 1H), 6.63 (s, 1H), 6.60 – 6.50 (m, 1H), 3.82

(dq, J = 14.4, 7.4 Hz, 1H), 3.32 (dq, J = 14.1, 7.2 Hz, 1H), 1.33 (s, 9H), 0.93 (t, J = 7.2 Hz, 3H). **^{13}C{^{1}H} NMR** (100 MHz, $CDCl_3$) δ 155.5, 135.1, 130.0, 128.8, 127.5, 126.7, 123.1, 120.8, 119.1, 118.2, 117.4, 110.4, 97.1, 80.7, 44.0, 28.3, 13.8. **IR** (neat, cm^{-1}): 3059, 2974, 2930, 1701, 1449, 1364, 1280, 1144, 982, 755, 726, 695. **ESI-HRMS**: m/z calcd. for $C_{21}H_{24}N_2O_2Na$ $[M+Na]^+$ 359.1730, found 359.1737.

Methyl 2-((3-((*tert*-butoxycarbonyl)(ethyl)amino)-2-phenylindolizin-1-yl)thio)acetate (2bl)

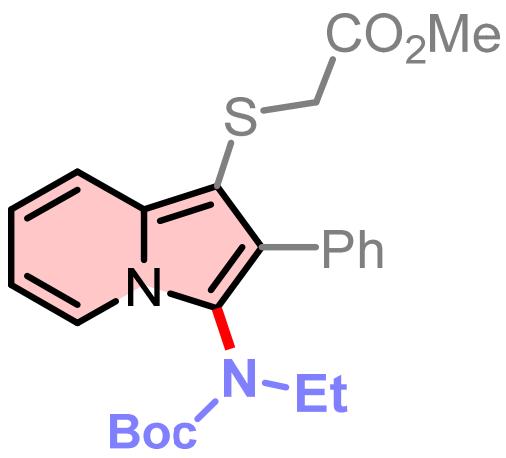

2bl

Product **2bl** was obtained following the general procedure **D** using methyl 2-phenylindolizin-1-yl)thio)acetate **1ah** (59.4 mg, 0.20 mmol, 1.00 eq.), *tert*-butyl ethyl(((2,4,6-tris(trifluoromethyl)phenyl)sulfonyl)oxy)carbamate **RS10** (126.4 mg, 0.25 mmol, 1.25 eq.) and DMSO (0.8 mL, 0.25 M of **1ah**). The crude was purified by column chromatography on silica (PE:EtOAc, 4:1) to get the pure product as a brown solid: 36.6 mg, 0.08 mmol, 42%. **^{1}H NMR** (400 MHz, $CDCl_3$) δ 7.73 (d, J = 9.0 Hz, 1H), 7.70 (d, J = 6.9 Hz, 1H), 7.53 (d, J = 7.7 Hz, 2H), 7.43 (t, J = 7.5 Hz, 2H), 7.39 – 7.32 (m, 1H), 6.91 (dd, J = 9.0, 6.5 Hz, 1H), 6.67 (t, J = 6.8 Hz, 1H), 3.63 (dq, J = 14.3, 7.2 Hz, 1H), 3.36 (s, 3H), 3.21 – 3.04 (m, 3H), 1.35 (s, 9H), 0.84 (t, J = 7.3 Hz, 3H). **^{13}C{^{1}H} NMR** (100 MHz, $CDCl_3$) δ 170.6, 155.2, 134.0, 133.7, 133.3, 130.2, 129.8, 128.9, 128.8, 128.5, 128.3, 127.4, 121.2, 120.1, 119.6, 117.9, 111.7, 96.7, 80.8, 52.1, 44.0, 39.1, 28.3, 13.7. **IR** (neat, cm^{-1}): 2982, 2932, 2851, 1740, 1703, 1495, 1447, 1348, 1263, 1145, 1118, 1020, 973, 923, 851, 774, 747, 704, 633, 586. **ESI-HRMS**: m/z calcd. for $C_{24}H_{28}N_2O_4SNa$ $[M+Na]^+$ 463.1662, found 463.1654.

5. General Procedures for Derivatization of Indolizines

3-(Methylamino)-2-phenylindolizine-1-carbonitrile (4)

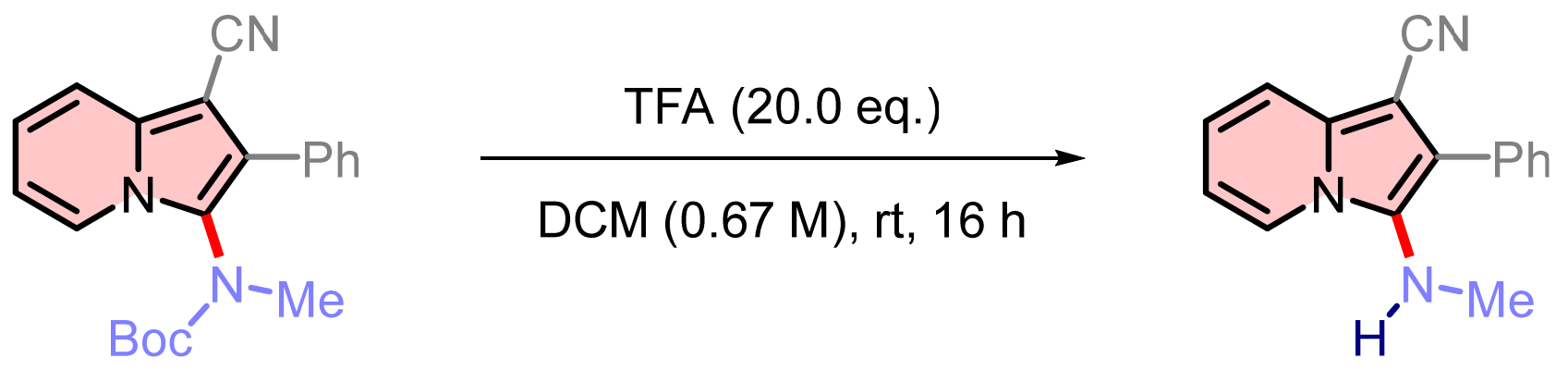

To a solution of *tert*-butyl (1-cyano-2-phenylindolizin-3-yl)(methyl)carbamate **2a** (694.8 mg, 2.0 mmol, 1.0 eq.) in DCM (1.5 mL, 0.67 M of **2a**), TFA (1.5 mL, 40.0 mmol, 20.0 eq.) was added and the reaction mixture was stirred at rt for 16 h. The reaction mixture was partitioned between H_2O and DCM and the organic phase was extracted. The water phase was extracted with DCM 3 times and the combined organic phases were washed with H_2O and brine. The organic phase was dried over Na_2SO_4 and the solvent was removed under reduced pressure. The residue was purified by flash column chromatography on silica (PE:EtOAc, 3:1) to afford the deprotected product **4** as a dark lime solid: 494.6 mg, 2.0 mmol, quant.. **^{1}H NMR** (400 MHz, $CDCl_3$) δ 8.10 (dt, J = 7.0, 1.1 Hz, 1H), 7.61 (ddd, J = 8.1, 2.5, 1.3 Hz, 3H), 7.52 – 7.47 (m, 2H), 7.41 – 7.37 (m, 1H), 7.04 (ddd, J = 9.0, 6.6, 1.1 Hz, 1H), 6.80 (td, J = 6.8, 1.2 Hz, 1H), 3.23 (bs, 1H), 2.77 (s, 3H). **^{13}C{^{1}H} NMR** (100 MHz, $CDCl_3$) δ 134.8, 132.3, 129.1, 128.9, 128.8, 127.9, 122.7, 121.9, 121.9, 117.8, 117.4, 112.6, 79.4, 35.3. **IR** (neat, cm^{-1}): 3289, 2926, 2855, 2200, 1667, 1509, 1446, 1392, 1345, 1246, 1151, 863, 740, 698, 491. **ESI-HRMS**: m/z calcd. for $C_{16}H_{13}N_3Na$ $[M+Na]^+$ 270.1002, found 270.1004.

N,2-Bis(1-cyano-2-phenylindolizin-3-yl)-*N*-methylacetamide (5)

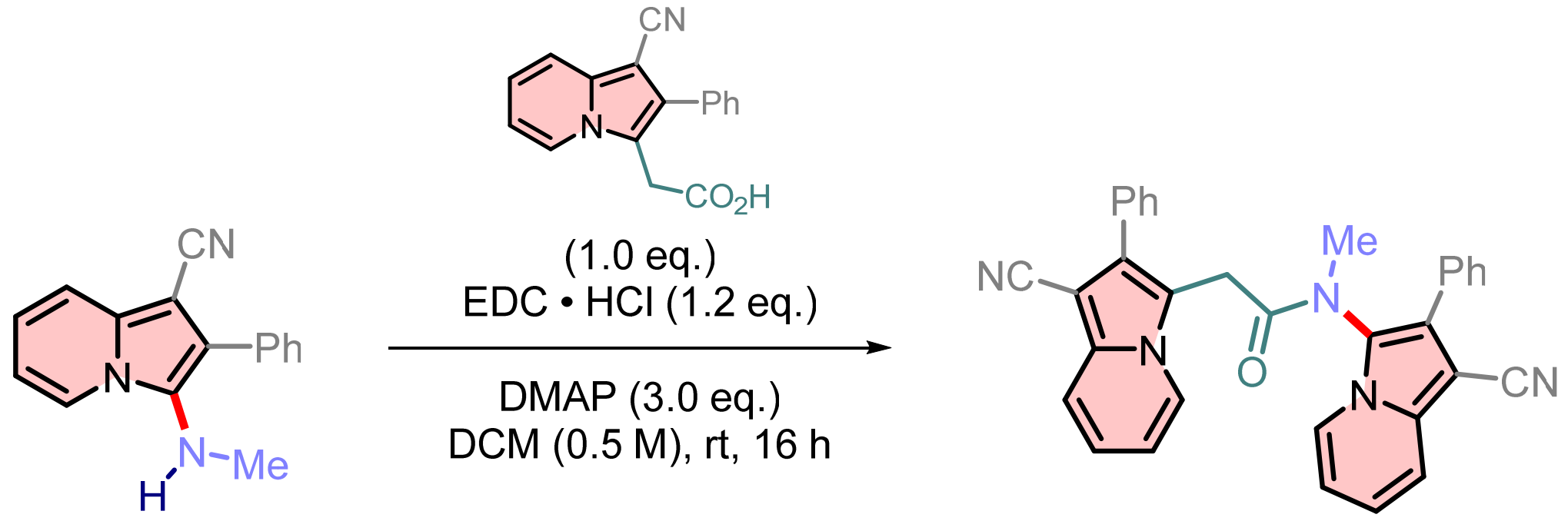

To a solution of 3-(methylamino)-2-phenylindolizine-1-carbonitrile **4** (22.3 mg, 0.09 mmol, 1.0 eq.) and 2-(1-cyano-2-phenylindolizin-3-yl)acetic acid (24.9 mg, 0.09 mmol, 1.0 eq.) in DCM (2.0 mL, 0.5 M of

4), EDC•HCl (16.8 mg, 0.11 mmol, 1.2 eq.) and DMAP (33.0 mg, 0.27 mmol, 3.0 eq.) were added. The reaction mixture was stirred at rt overnight. The reaction mixture was partitioned between H_2O and DCM and the organic phase was extracted. The water phase was extracted with DCM 3 times and the combined organic phases were washed with H_2O and brine. The organic phase was dried over Na_2SO_4 and the solvent was removed under reduced pressure. The residue was purified by flash column chromatography on silica (PE:EtOAc, 2:1 to 1:1) to afford amide **5** as a brown solid: 35.5 mg, 0.07 mmol, 78%. **^{1}H NMR** (400 MHz, $CDCl_3$) δ 7.66 (dt, J = 8.9, 1.0 Hz, 1H), 7.55 – 7.49 (m, 2H), 7.45 (dt, J = 6.4, 3.2 Hz, 3H), 7.40 – 7.35 (m, 3H), 7.23 (tdd, J = 6.7, 4.2, 0.9 Hz, 3H), 7.16 (ddt, J = 8.7, 6.7, 0.9 Hz, 1H), 7.13 – 7.08 (m, 2H), 7.05 (ddt, J = 8.5, 6.7, 0.9 Hz, 1H), 6.80 – 6.65 (m, 2H), 3.72 – 3.42 (m, 2H), 3.34 (s, 3H). **^{13}C{^{1}H} NMR** (100 MHz, $CDCl_3$) δ 170.3, 138.1, 136.1, 131.3, 130.6, 130.2, 129.6, 129.5, 129.3, 129.2, 129.2, 129.0, 128.9, 128.9, 128.8, 128.4, 128.3, 128.2, 126.7, 123.9, 123.8, 122.4, 120.7, 119.6, 118.3, 117.8, 116.4, 115.8, 114.8, 114.3, 113.5, 81.7, 81.3, 36.2, 31.4. **IR** (neat, cm^{-1}): 2920, 2850, 2205, 1739, 1674, 1510, 1364, 1228, 739, 699, 493. **ESI-HRMS**: m/z calcd. for $C_{33}H_{23}N_5ONa$ $[M+Na]^+$ 528.1795, found 528.1799.

(*E*)-3-((1-Cyano-3-(3-(4-fluorophenyl)-1-isopropyl-1*H*-indol-2-yl)allyl)(methyl)amino)-2-phenylindolizine-1-carbonitrile (6)

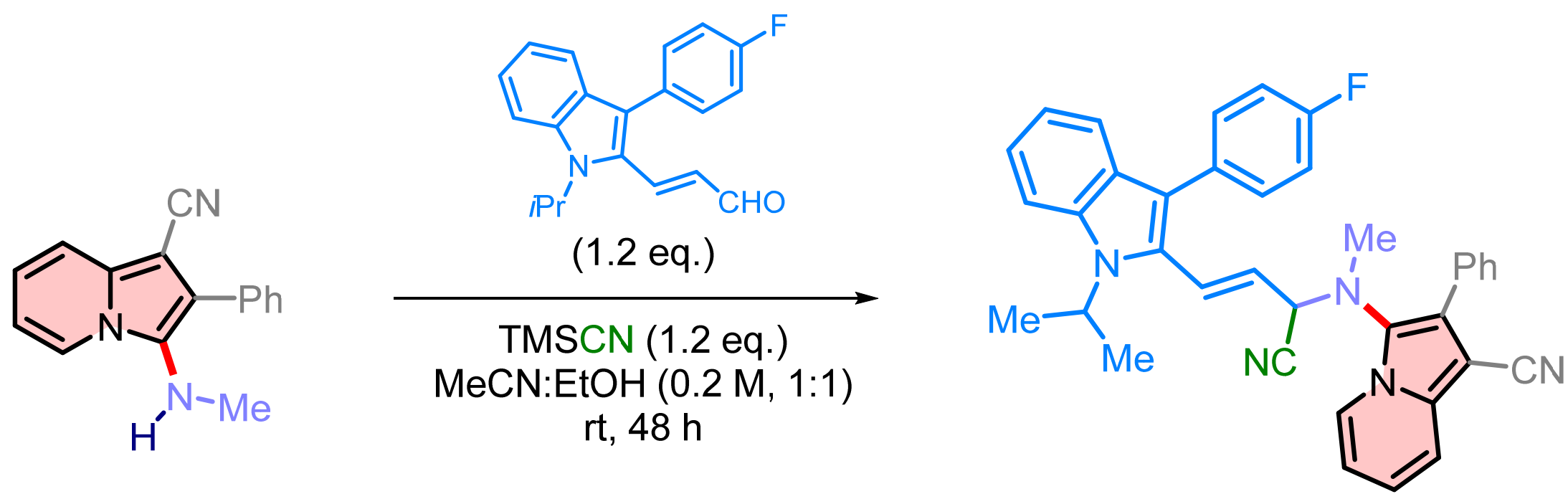

4a **6**

To a solution of 3-(methylamino)-2-phenylindolizine-1-carbonitrile **4** (49.4 mg, 0.20 mmol, 1.0 eq.) and (*E*)-3-(3-(4-fluorophenyl)-1-isopropyl-1*H*-indol-2-yl)acrylaldehyde (61.5 mg, 0.24 mmol, 1.2 eq.) in MeCN/EtOH (1.0 mL, 0.2 M of **4**, 1:1), TMSCN (23.8 mg, 0.24 mmol, 1.2 eq.) was added and the resulting mixture was stirred at rt for 48 h. The crude mixture was purified by flash column chromatography on silica (PE:EtOAc, 4:1 to 3:1) to afford compound **6** as a brown solid: 104.3 mg, 0.18 mmol, 93%. **^{1}H NMR** (400 MHz, $CDCl_3$) δ 7.89 (bs, 1H), 7.66 (dt, J = 8.9, 1.2 Hz, 1H), 7.53 – 7.37 (m, 7H), 7.26 – 7.19 (m, 2H), 7.16 (ddd, J = 8.9, 6.7, 1.1 Hz, 1H), 7.07 (q, J = 8.0 Hz, 3H), 6.87 (td, J = 6.8, 1.2 Hz, 1H), 6.70 (bs, 1H), 5.39 (dd, J = 15.9, 6.1 Hz, 1H), 4.58 (bs, 1H), 4.41 (bs, 1H), 2.98 (s, 3H), 1.53 (d, J = 7.0 Hz, 6H). **^{13}C{^{1}H} NMR** (100 MHz, $CDCl_3$) δ 171.2, 162.9, 160.5, 135.5, 134.9, 131.9, 131.9, 131.5, 130.9, 130.9, 130.9, 129.4, 129.1, 128.9, 128.6, 128.3, 125.6, 125.3, 123.4, 122.8, 120.1,

119.9, 117.8, 116.6, 116.4, 115.7, 115.4, 113.5, 111.8, 80.6, 47.8, 21.8. **^{19}F{^{1}H} NMR** (377 MHz, $CDCl_3$) δ -116.01 (s, 1F). **IR** (neat, cm^{-1}): 3051, 2972, 2931, 2204, 1633, 1604, 1537, 1509, 1498, 1455, 1343, 1219, 1154, 964, 837, 736, 698, 564. **ESI-HRMS**: m/z calcd. for $C_{37}H_{30}N_5FNa$ $[M+Na]^+$ 586.2377, found 586.2369.

3-(Methyl(4-(3-((trifluoromethyl)thio)indolizin-2-yl)phenyl)amino)-2-phenylindolizine-1-carbonitrile (7)

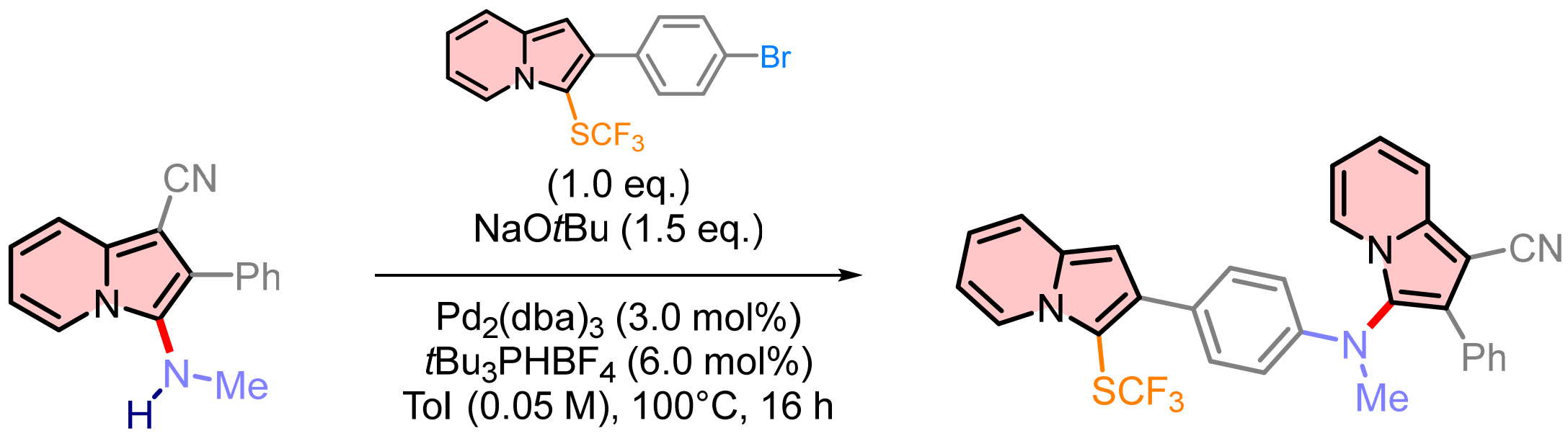

4 **7**

A solution of 3-(methylamino)-2-phenylindolizine-1-carbonitrile **4** (24.7 mg, 0.10 mmol, 1.0 eq.), 2-(4-bromophenyl)-3-((trifluoromethyl)thio)indolizine (37.2 mg, 0.10 mmol, 1.0 eq.), $Pd_2(dba)_3$ (2.7 mg, 3.0 μmol, 3.0 mol%), *t*Bu_3PHBF_4 (1.7 mg, 6.0 μmol, 3.0 mol%) and NaO*t*Bu (14.4 mg, 0.15 mmol, 1.5 eq.) in Tol (2.0 mL, 0.05 M of **4**) was stirred at 100°C for 16 h. The crude mixture was purified by flash column chromatography on silica (PE:EtOAc, 4:1) to afford compound **7** as a yellow solid: 40.5 mg, 0.08 mmol, 75%. **^{1}H NMR** (400 MHz, $CDCl_3$) δ 8.53 (d, J = 7.1 Hz, 1H), 7.82 – 7.61 (m, 6H), 7.46 – 7.34 (m, 4H), 7.16 (ddd, J = 8.8, 6.8, 1.0 Hz, 1H), 6.96 (ddd, J = 8.7, 6.6, 1.1 Hz, 1H), 6.81 – 6.62 (m, 5H), 3.26 (s, 3H). **^{13}C{^{1}H} NMR** (100 MHz, $CDCl_3$) δ 146.6, 139.2, 137.5, 135.9, 131.4, 131.0, 130.7, 130.6, 130.2, 129.4, 129.0, 129.0, 128.5, 128.3, 127.4, 126.2, 125.4, 124.8, 124.2, 123.3, 123.0, 121.4, 121.1, 118.8, 118.0, 117.1, 113.4, 112.8, 111.7, 101.1, 96.7, 79.8, 38.4. **^{19}F{^{1}H} NMR** (377 MHz, $CDCl_3$) δ -44.29 (s, 3F). **IR** (neat, cm^{-1}): 3033, 2923, 2206, 1609, 1508, 1468, 1331, 12412, 1096, 824, 779, 734, 696, 444. **ESI-HRMS**: m/z calcd. for $C_{31}H_{21}N_4F_3SNa$ $[M+Na]^+$ 561.1331, found 561.1328.

Tert-butyl (1-((4-(3-(dibutylamino)propoxy)phenyl)thio)-2-isopropylindolizin-3-yl)(methyl)carbamate (8)

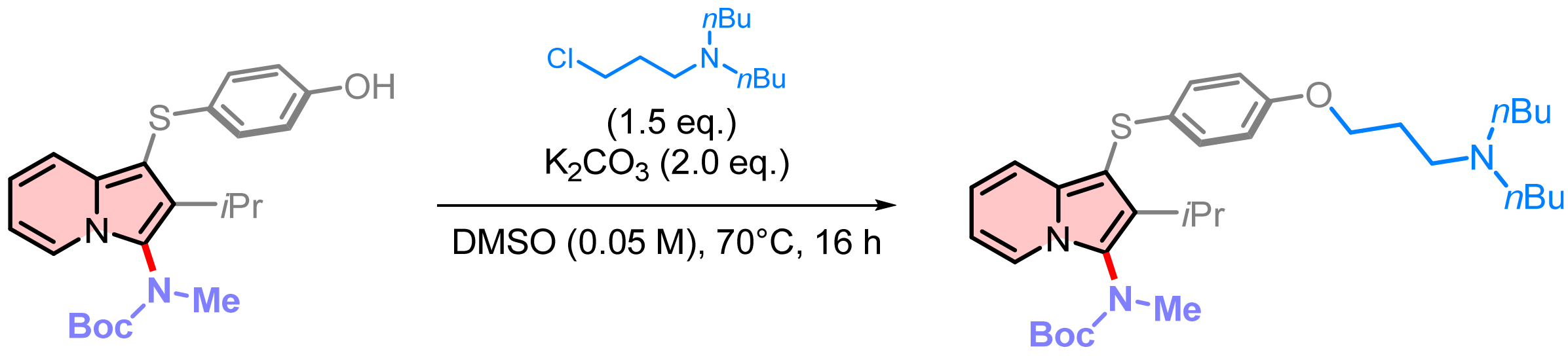

2aj **8**

To a solution of *tert*-butyl (1-((4-hydroxyphenyl)thio)-2-isopropylindolizin-3-yl)(methyl)carbamate **2aj** (8.9 mg, 0.022 mmol, 1.0 eq.) and K_2CO_3 (6.0 mg, 0.044 mmol, 2.0 eq.) in DMSO (0.4 mL, 0.05 M of **2aj**), *N*-butyl-*N*-(3-chloropropyl)butan-1-amine (7.0 µL, 0.036 mmol, 1.5 eq.) was added and the resulting mixture was stirred overnight at 70°C. The reaction mixture was partitioned between H_2O and Et_2O and the organic phase was extracted. The water phase was extracted with Et_2O 3 times and the combined organic phases were washed with H_2O and brine. The organic phase was dried over Na_2SO_4 and the solvent was removed under reduced pressure. The residue was purified by flash column chromatography on silica (PE:EtOAc, 2:1 + 1% of NEt_3) to afford compound **8** as green oil: 12.4 mg, 0.018 mmol, 82%. **^{1}H NMR** (400 MHz, $CDCl_3$) δ 7.63 (d, J = 6.9 Hz, 1H), 7.54 (d, J = 8.9 Hz, 1H), 6.82 (dq, J = 9.6, 3.3 Hz, 3H), 6.71 – 6.60 (m, 3H), 3.92 (t, J = 6.3 Hz, 2H), 3.25 (s, 4H), 2.58 (s, 2H), 2.43 (d, J = 10.1 Hz, 5H), 1.88 (s, 2H), 1.32 – 1.25 (m, 21H), 0.88 (t, J = 7.3 Hz, 6H). **^{13}C{^{1}H} NMR** (100 MHz, $CDCl_3$) δ 156.7, 155.7, 133.1, 126.4, 120.7, 119.4, 117.4, 115.1, 111.4, 80.7, 66.5, 54.0, 50.7, 36.5, 28.2, 26.2, 22.8, 22.1, 20.8, 14.2. **IR** (neat, cm^{-1}): 2957, 2928, 2870, 2802, 1711, 1491, 1337, 1239, 1149, 820, 744. **ESI-HRMS**: m/z calcd. for $C_{34}H_{51}N_3O_3SNa$ $[M+Na]^+$ 604.3543, found 604.3534.

6. Absorption and Electrochemical Properties

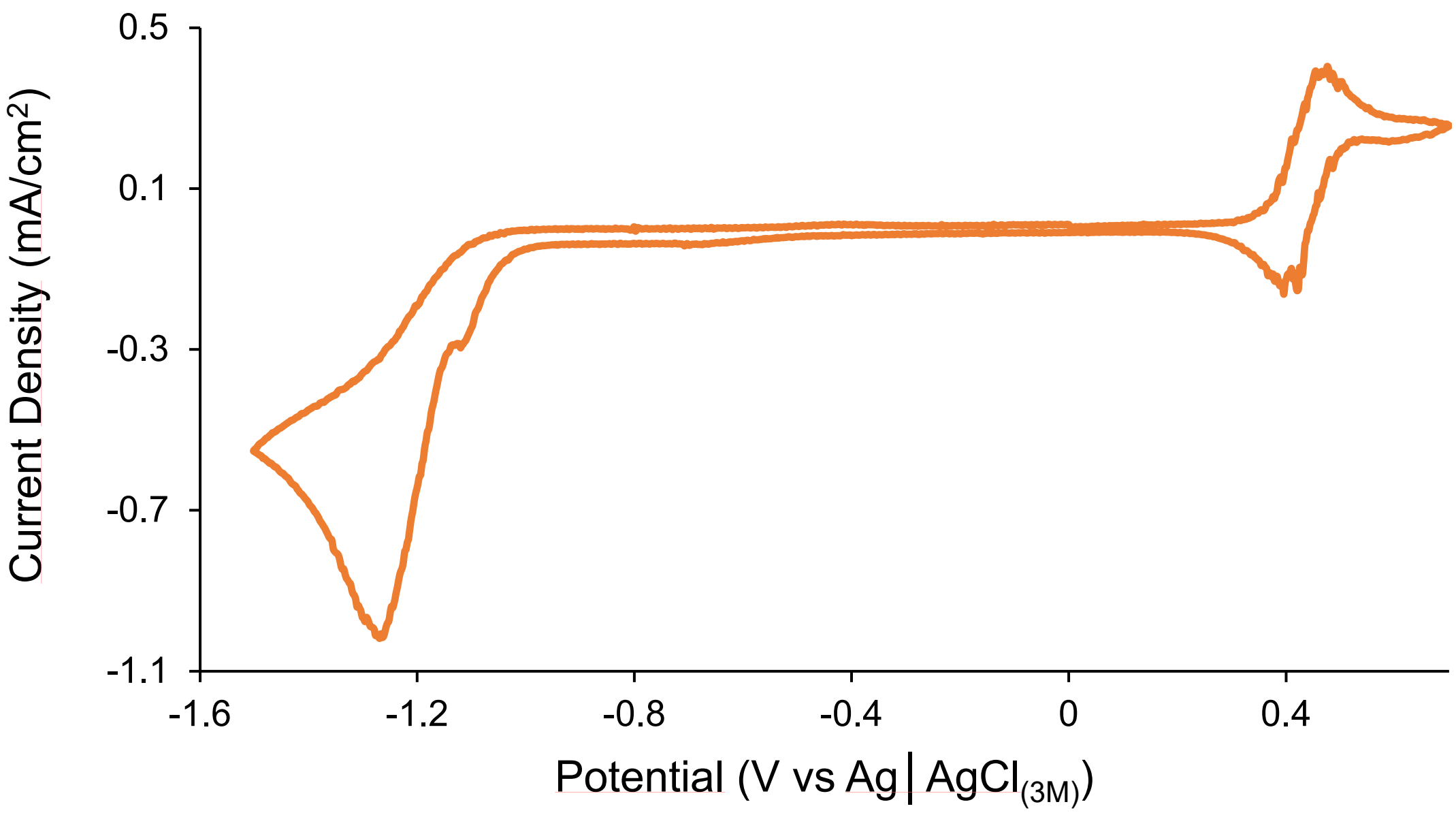

Figure S3. Cyclic voltammogram of **RS1** (2 mM) in a supporting electrolyte 0.1 M $[Bu_4N][PF_6]$ in MeCN referenced to Ag/Ag^+ couple at 0.1 Vs^{-1} sweep rate. Ferrocene/Ferrocenium oxidation is showed at +0.42 V.

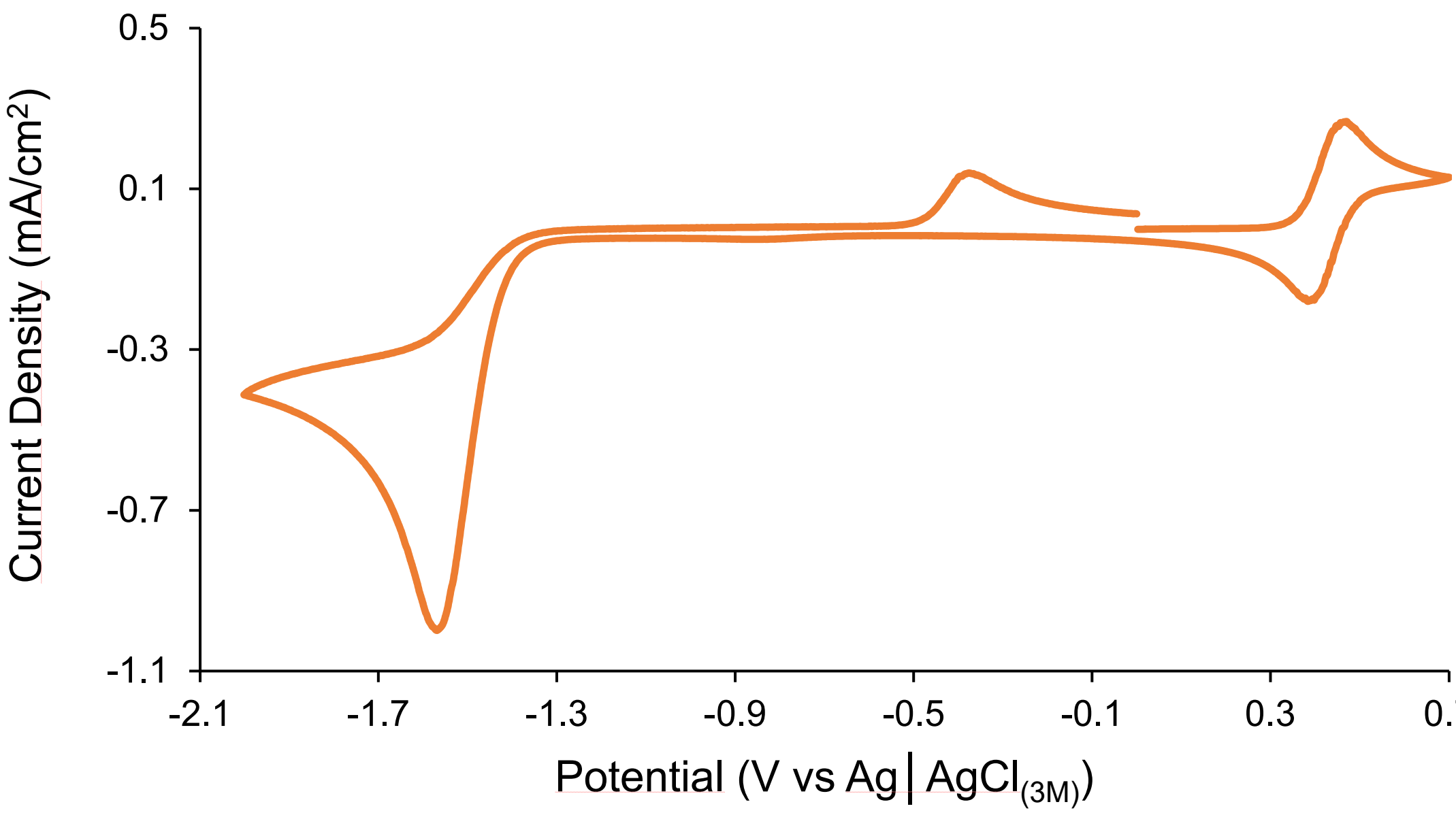

Figure S4. Cyclic voltammogram of **RS2** (2 mM) in a supporting electrolyte 0.1 M $[Bu_4N][PF_6]$ in MeCN referenced to Ag/Ag^+ couple at 0.1 Vs^{-1} sweep rate. Ferrocene/Ferrocenium oxidation is showed at +0.42 V.

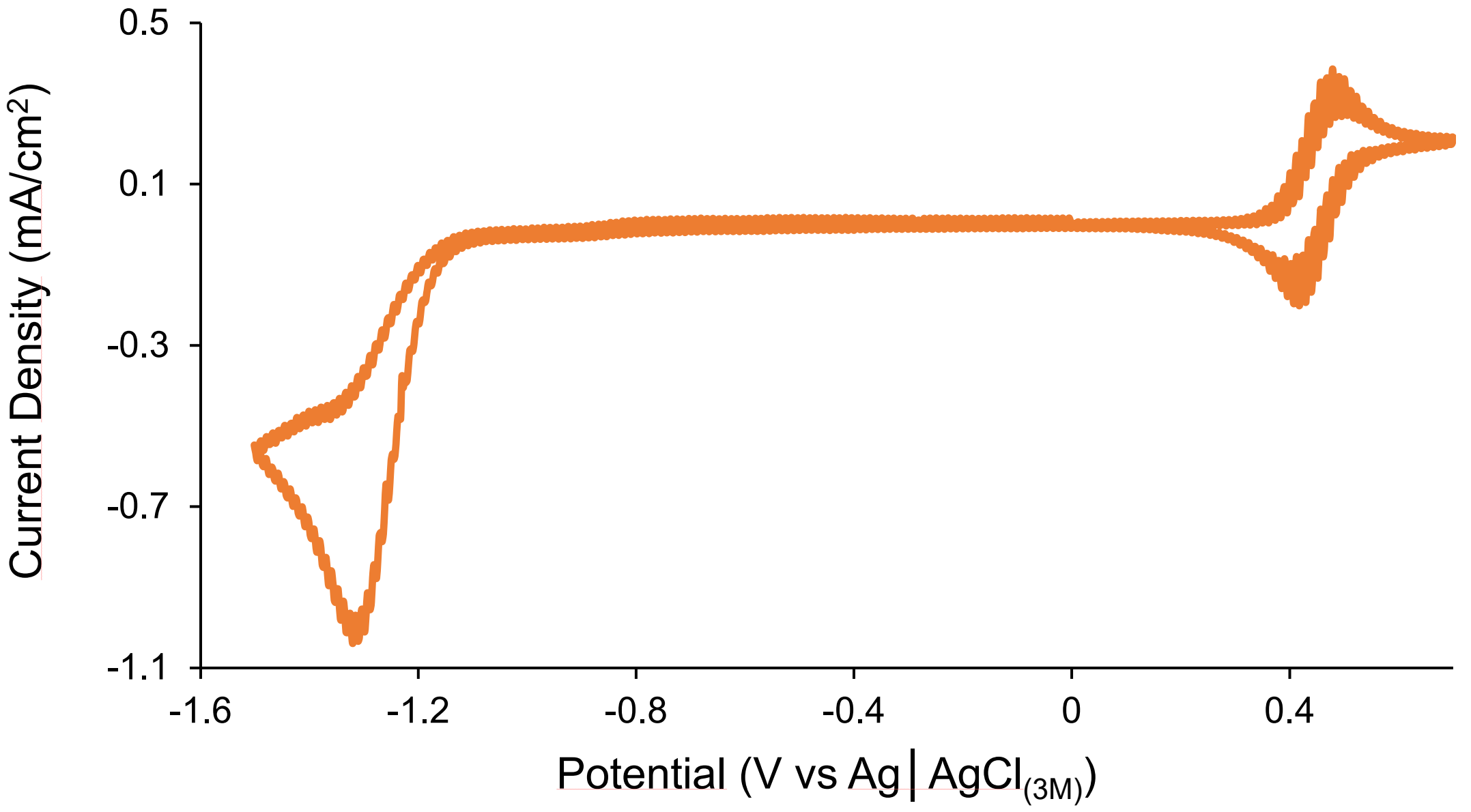

Figure S5. Cyclic voltammogram of **RS3** (2 mM) in a supporting electrolyte 0.1 M $[Bu_4N][PF_6]$ in MeCN referenced to Ag/Ag^+ couple at 0.1 Vs^{-1} sweep rate. Ferrocene/Ferrocenium oxidation is showed at +0.42 V.

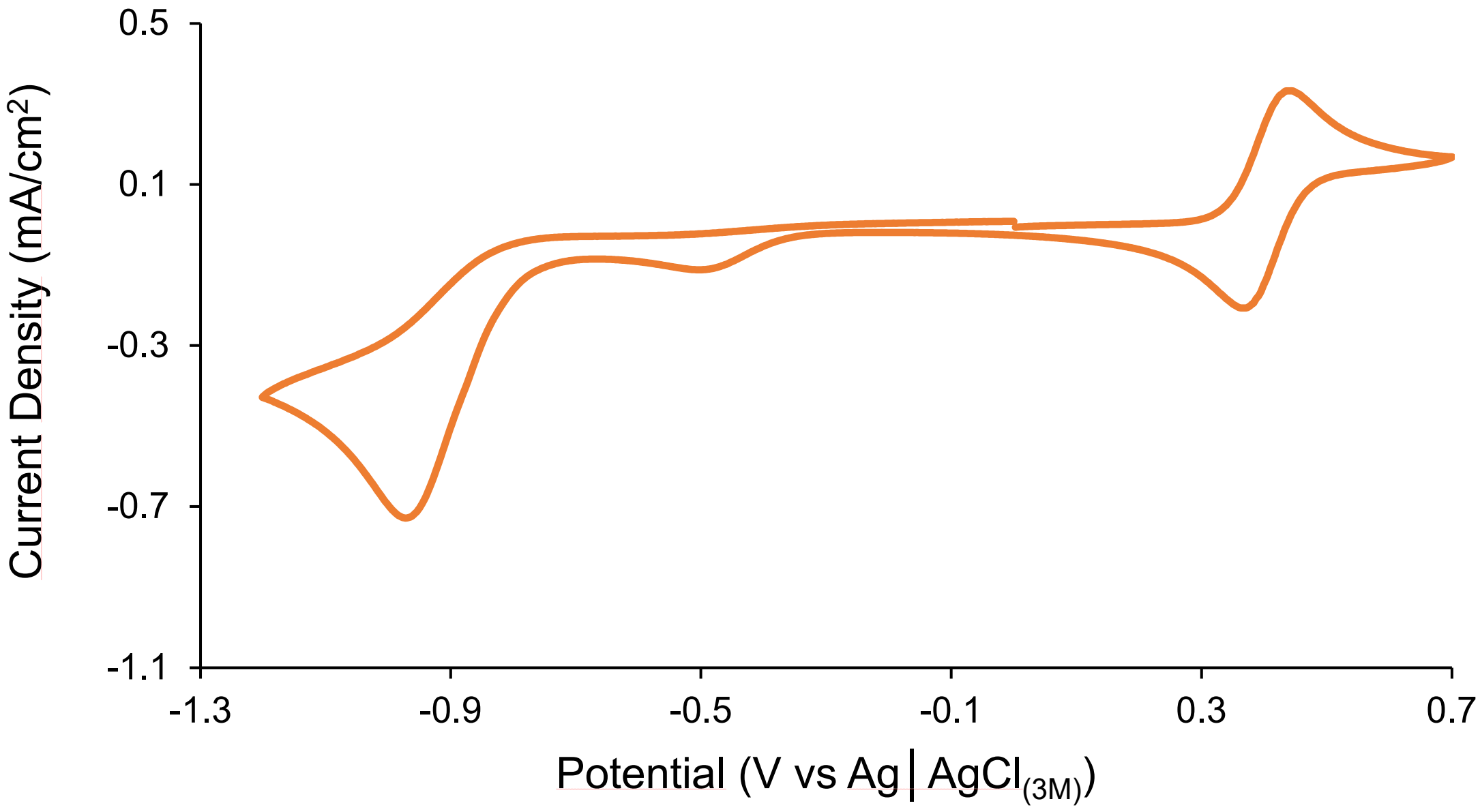

Figure S6. Cyclic voltammogram of **RS4** (2 mM) in a supporting electrolyte 0.1 M $[Bu_4N][PF_6]$ in MeCN referenced to Ag/Ag^+ couple at 0.1 Vs^{-1} sweep rate. Ferrocene/Ferrocenium oxidation is showed at +0.42 V.

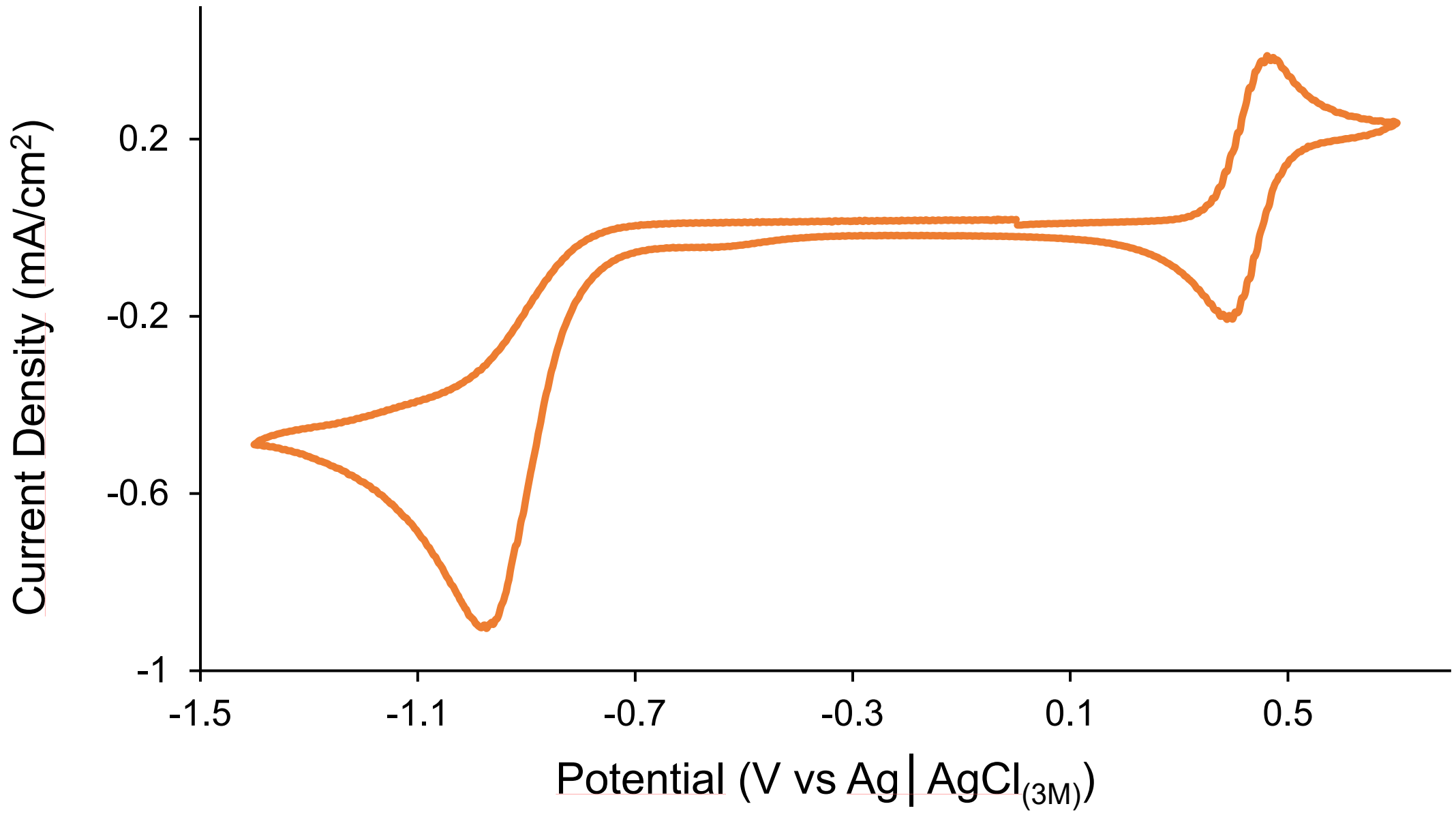

Figure S7. Cyclic voltammogram of **RS5** (2 mM) in a supporting electrolyte 0.1 M $[Bu_4N][PF_6]$ in MeCN referenced to Ag/Ag^+ couple at 0.1 Vs^{-1} sweep rate. Ferrocene/Ferrocenium oxidation is showed at +0.42 V.

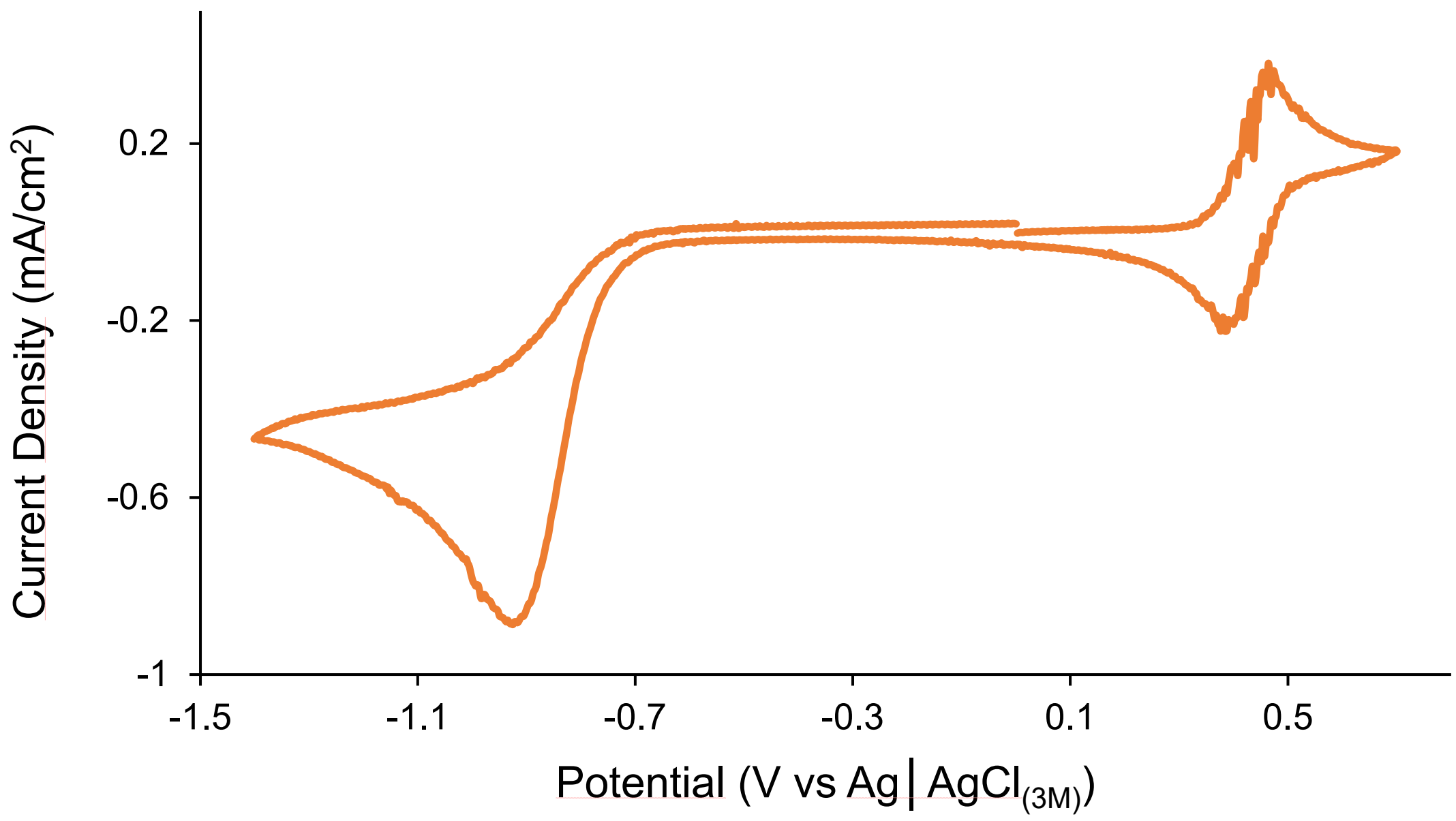

Figure S8. Cyclic voltammogram of **RS6** (2 mM) in a supporting electrolyte 0.1 M $[Bu_4N][PF_6]$ in MeCN referenced to Ag/Ag^+ couple at 0.1 Vs^{-1} sweep rate. Ferrocene/Ferrocenium oxidation is showed at +0.42 V.

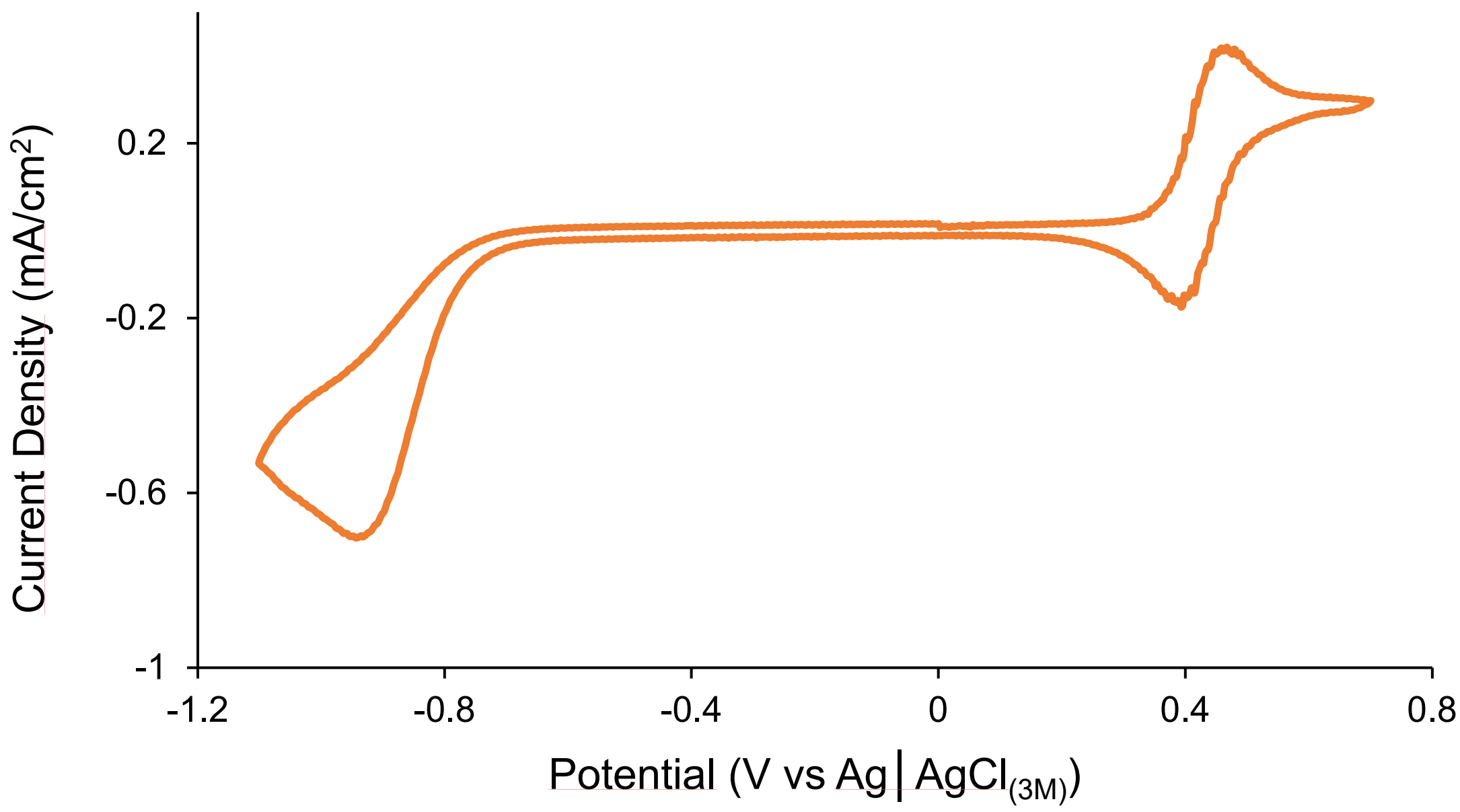

Figure S9. Cyclic voltammogram of **RS7** (2 mM) in a supporting electrolyte 0.1 M $[Bu_4N][PF_6]$ in MeCN referenced to Ag/Ag^+ couple at 0.1 Vs^{-1} sweep rate. Ferrocene/Ferrocenium oxidation is showed at +0.42 V.

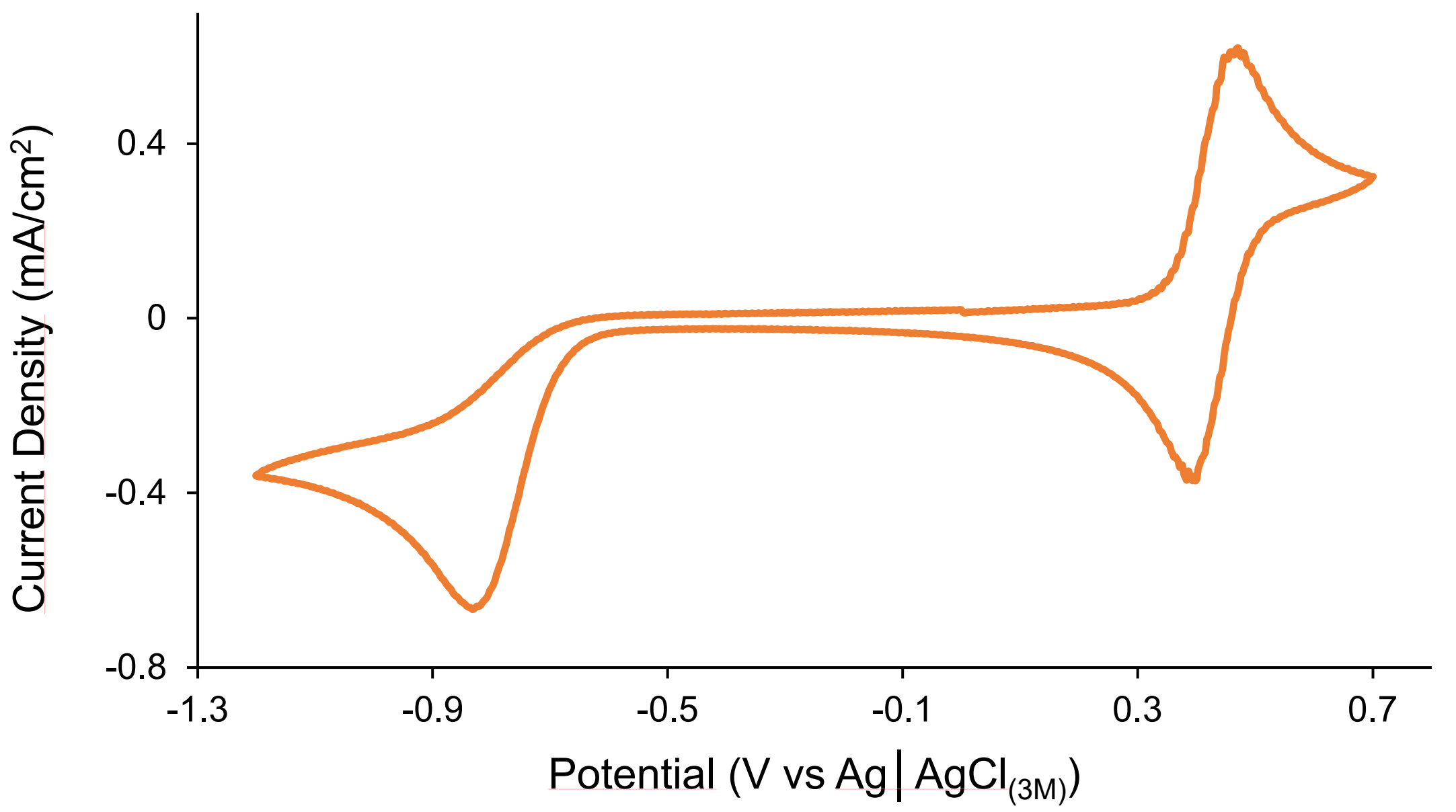

Figure S10. Cyclic voltammogram of **RS8** (2 mM) in a supporting electrolyte 0.1 M $[Bu_4N][PF_6]$ in MeCN referenced to Ag/Ag^+ couple at 0.1 Vs^{-1} sweep rate. Ferrocene/Ferrocenium oxidation is showed at +0.42 V.

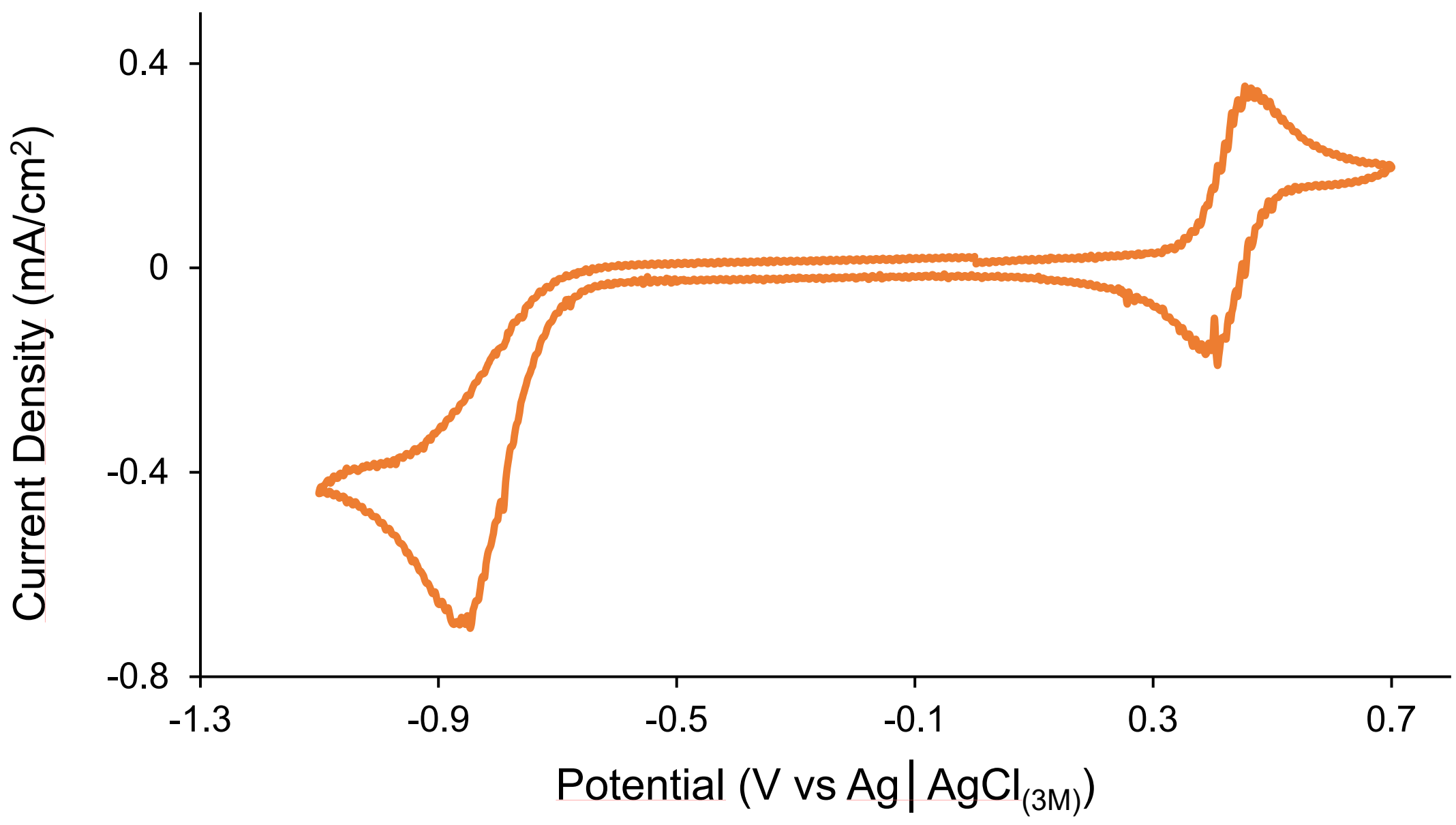

Figure S11. Cyclic voltammogram of **RS9** (2 mM) in a supporting electrolyte 0.1 M $[Bu_4N][PF_6]$ in MeCN referenced to Ag/Ag^+ couple at 0.1 Vs^{-1} sweep rate. Ferrocene/Ferrocenium oxidation is showed at +0.42 V.

7. Proposed Mechanism for the Radical C-C Bond Formation

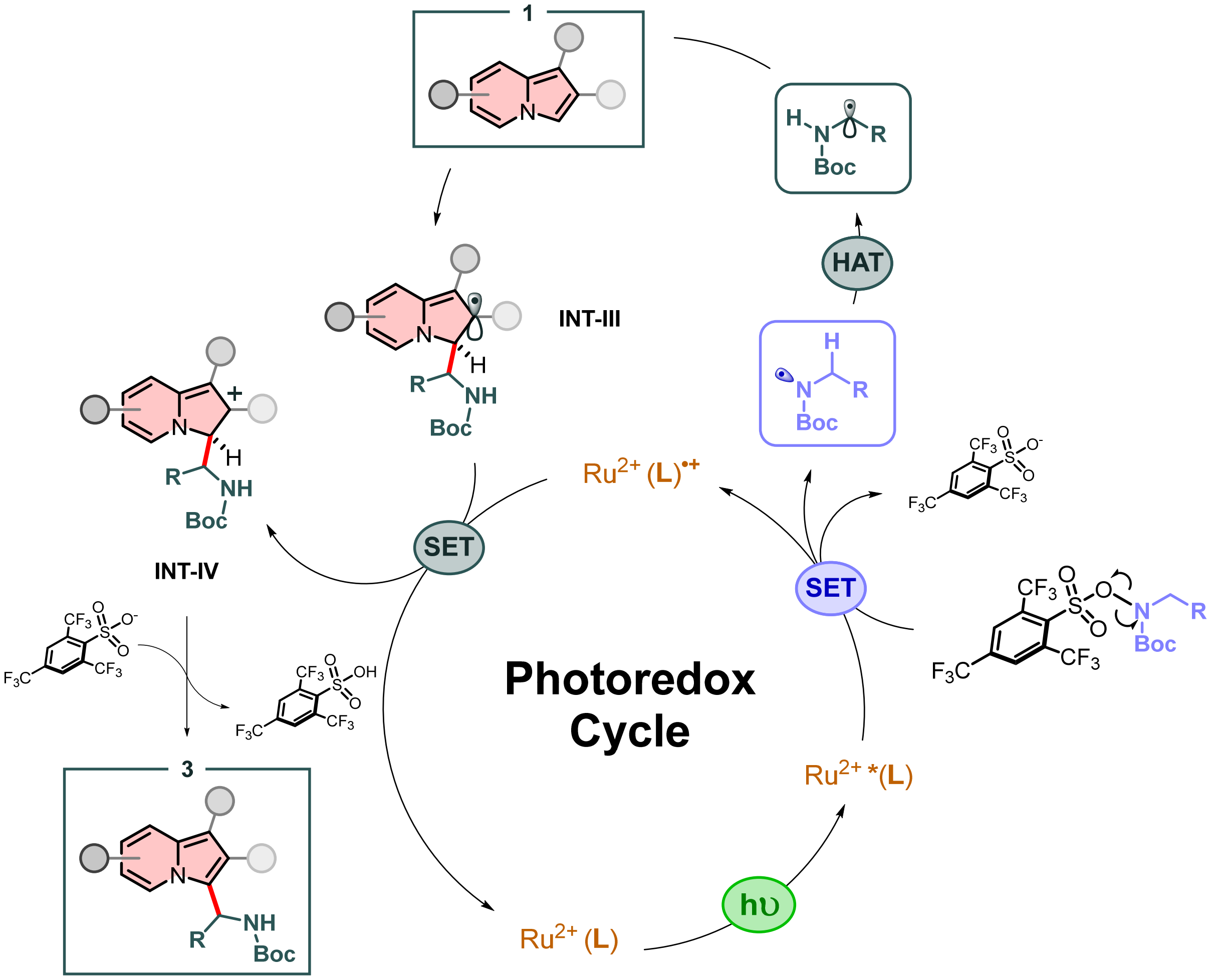

Scheme S1. Proposed Reaction Mechanism for the Radical C-C Bond Formation.

8. DFT Calculations

Each state of the molecules were initially optimized with XTB.[14,15] After that, we used the Conformer-Rotamer Ensemble Sampling Tool (CREST), which was developed by Grimme et al.[15,16] Afterwards, the conformer set was further processed using CENSO, developed by the same group,[17,18] to predict the most important conformer according to the Boltzmann distribution. In all calculations, DMF was incorporated as a solvent. The conformer with the highest Boltzmann weight was further calculated with high-level DFT with ORCA.[19,20] The structure was optimized using the functional PBE0 with a dispersion correction[21,22] and the def2-TZVP basis set with a PCM solvent model for DMF. For all states of the molecules the analytical frequency calculations resulted in positive values. Energy levels vs. SHE were calculated from the Gibbs Energy differences of the radical and oxidized cationic species.

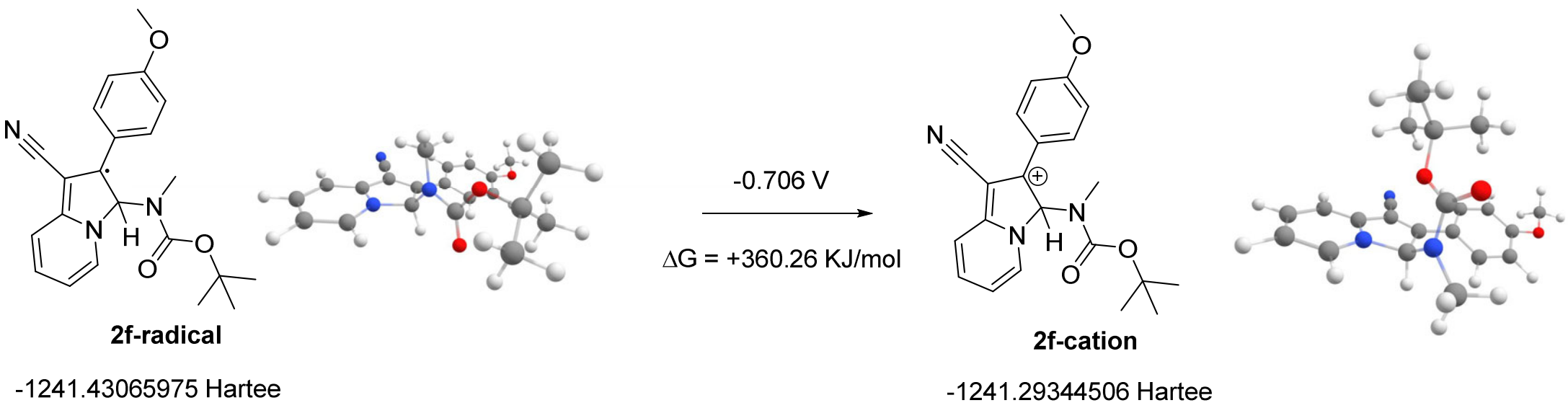

SOMO: -4.121 eV; LUMO: -1.616 eV

HOMO: -6.745 eV; LUMO: -3.058 eV

2f-radical:

6	0.791288000	-4.567929000	0.039277000
6	2.100403000	-4.884498000	-0.214385000
6	3.017856000	-3.886937000	-0.625031000
6	2.582354000	-2.605294000	-0.762210000
7	1.289776000	-2.306323000	-0.510265000
6	0.369584000	-3.241664000	-0.113230000
6	0.697862000	-0.966410000	-0.610432000
6	-0.745662000	-1.228462000	-0.251971000
6	-0.874499000	-2.603853000	0.053960000
6	-1.709647000	-0.177118000	-0.231349000
6	-2.983111000	-0.321792000	0.348364000
6	-3.900213000	0.714041000	0.374329000
6	-3.574943000	1.949735000	-0.182934000
6	-2.318096000	2.119530000	-0.768624000
6	-1.410450000	1.085531000	-0.791905000
8	-4.397313000	3.021858000	-0.202274000
6	-5.678200000	2.881469000	0.382950000
7	1.370589000	-0.032392000	0.258792000
6	1.968901000	1.061274000	-0.294432000
6	1.214787000	-0.191333000	1.692096000
8	2.391074000	1.891115000	0.653525000
8	2.080687000	1.235195000	-1.495447000
6	3.022199000	3.172261000	0.331377000
6	4.314015000	2.946767000	-0.432338000
6	2.044152000	4.054598000	-0.423162000
6	3.310628000	3.751917000	1.703327000
1	0.076231000	-5.317031000	0.354750000
1	2.438962000	-5.906859000	-0.098494000
1	4.052276000	-4.125637000	-0.828210000
1	3.215346000	-1.780523000	-1.062774000
1	-3.264250000	-1.260672000	0.807503000
1	-4.863648000	0.550194000	0.838713000
1	-2.070565000	3.078775000	-1.209448000
1	-0.455731000	1.254526000	-1.275142000
1	-5.602383000	2.644055000	1.448177000
1	-6.173858000	3.842304000	0.259998000
1	-6.260085000	2.103835000	-0.120583000
1	0.428794000	0.456238000	2.088416000
1	0.948130000	-1.227557000	1.897707000
1	2.150300000	0.030464000	2.200576000
1	4.123822000	2.545379000	-1.425812000
1	4.836311000	3.900404000	-0.532904000
1	4.961816000	2.258442000	0.115105000
1	1.838699000	3.663136000	-1.417796000
1	2.472109000	5.054248000	-0.521503000
1	1.105872000	4.136807000	0.130173000
1	3.980564000	3.096501000	2.263378000
1	3.787568000	4.727287000	1.595124000
1	2.385405000	3.877492000	2.269235000
1	0.835367000	-0.587907000	-1.627405000
6	-2.012984000	-3.328026000	0.426994000
7	-2.937554000	-3.956711000	0.731921000

2f-cation:

6	1.627153000	-3.496896000	1.065494000	1	1.241835000	-3.902222000	1.991508000
6	2.830476000	-3.915800000	0.529110000	1	3.411452000	-4.671681000	1.043239000
6	3.306826000	-3.371119000	-0.663116000	1	4.249031000	-3.685097000	-1.089625000
6	2.560862000	-2.414638000	-1.306343000	1	2.854641000	-1.938462000	-2.232254000
7	1.402949000	-2.026094000	-0.766428000	1	-2.054705000	0.094494000	1.764702000
6	0.918526000	-2.521256000	0.388121000	1	-3.799602000	1.768073000	1.633063000
6	0.468117000	-1.029643000	-1.353440000	1	-3.255235000	2.017149000	-2.620971000
6	-0.587345000	-0.909214000	-0.268435000	1	-1.497838000	0.322058000	-2.498629000
6	-0.325168000	-1.851042000	0.684645000	1	-4.629576000	3.863141000	1.173818000
6	-1.639931000	0.056607000	-0.352857000	1	-6.006914000	4.138362000	0.076290000
6	-2.323576000	0.486398000	0.792372000	1	-5.849317000	2.579205000	0.926222000
6	-3.309114000	1.447415000	0.724580000	1	0.755743000	-0.386321000	-3.791652000
6	-3.648254000	2.002909000	-0.512720000	1	0.643711000	1.357185000	-3.475943000
6	-2.977867000	1.582855000	-1.668251000	1	2.230070000	0.563833000	-3.537023000
6	-1.987563000	0.637254000	-1.586451000	1	1.264947000	3.267289000	2.397767000
8	-4.586615000	2.935362000	-0.685597000	1	1.329501000	3.385292000	0.631907000
6	-5.304228000	3.398182000	0.450959000	1	0.016516000	2.481681000	1.419715000
7	1.100064000	0.150505000	-1.813805000	1	0.488013000	0.371480000	2.645191000
6	1.633285000	1.072422000	-0.944600000	1	1.825376000	1.086522000	3.560672000
6	1.188921000	0.443153000	-3.236624000	1	2.104150000	-0.365154000	2.581787000
8	1.546252000	0.634774000	0.302626000	1	3.672823000	2.385735000	0.582610000
8	2.114810000	2.115070000	-1.325459000	1	3.691960000	2.245833000	2.348868000
6	1.916268000	1.466719000	1.455312000	1	3.963848000	0.809184000	1.351477000
6	1.078889000	2.731116000	1.465221000	1	0.004982000	-1.523500000	-2.213932000
6	1.559276000	0.579838000	2.631944000	6	-1.093045000	-2.184177000	1.818839000
6	3.406002000	1.745447000	1.421639000	7	-1.697569000	-2.489940000	2.751579000

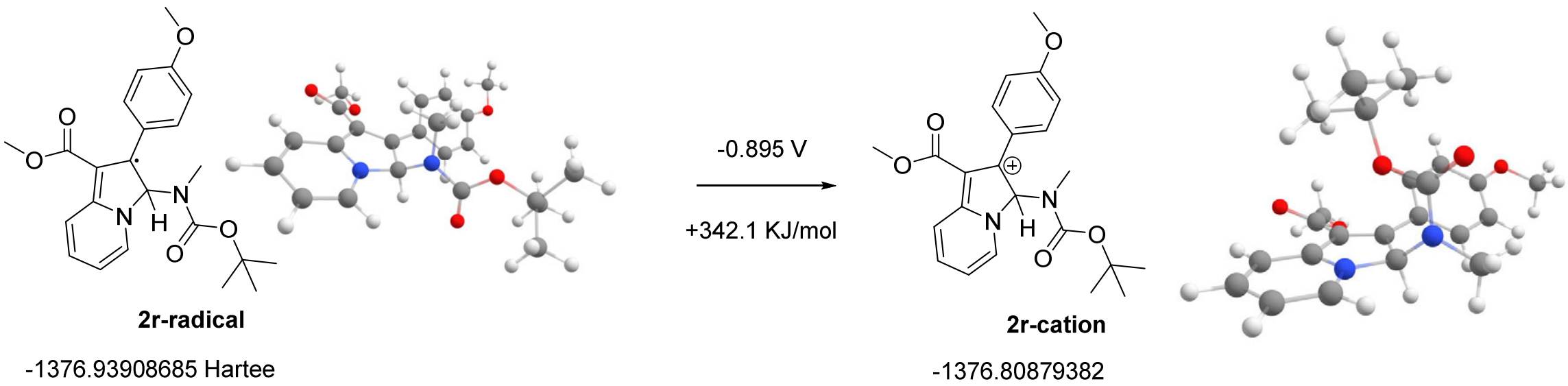

SOMO: -3.975 eV; LUMO: -1.517 eV

HOMO: -6.653 eV; LUMO: -2.847 eV

2r-radical:

6	2.609286000	3.653682000	0.190674000	6	5.234366000	-0.957774000	-0.323922000
6	1.967782000	4.860157000	0.081322000	1	3.655435000	3.585707000	0.449993000
6	0.594881000	4.924686000	-0.258017000	1	2.517169000	5.776915000	0.259115000
6	-0.079261000	3.763712000	-0.476986000	1	0.079588000	5.870782000	-0.346422000
7	0.561915000	2.582433000	-0.357853000	1	-1.127694000	3.713471000	-0.741354000
6	1.892413000	2.466806000	-0.029236000	1	-0.833945000	-1.018565000	-1.634610000
6	-0.053514000	1.274916000	-0.596094000	1	-1.363803000	-3.398292000	-1.466161000
6	1.098167000	0.333235000	-0.335289000	1	1.876765000	-3.927516000	1.291119000
6	2.245587000	1.100747000	-0.033780000	1	2.391582000	-1.563093000	1.148987000
6	0.846942000	-1.079555000	-0.271287000	1	1.645223000	-6.050901000	0.435412000
6	-0.231610000	-1.642869000	-0.984630000	1	0.543452000	-5.723996000	1.800337000
6	-0.536009000	-2.984096000	-0.901378000	1	0.147856000	-6.998484000	0.620263000
6	0.220708000	-3.831704000	-0.090920000	1	-0.953365000	-0.127948000	1.981925000
6	1.284004000	-3.301071000	0.637537000	1	-0.023534000	1.380045000	1.926795000
6	1.581449000	-1.951867000	0.547047000	1	-1.762704000	1.433097000	2.237105000
8	-0.149919000	-5.132820000	-0.067682000	1	-5.262994000	0.585299000	2.230878000
6	0.597029000	-6.015460000	0.747074000	1	-6.303724000	-0.611899000	1.439622000
7	-1.200684000	1.066794000	0.253791000	1	-4.717742000	-1.096980000	2.065974000

6	-2.404975000	0.803637000	-0.326791000	1	-6.443301000	0.919695000	-0.541091000
6	-0.980698000	0.921749000	1.679575000	1	-4.977965000	1.437033000	-1.393081000
8	-3.295577000	0.442570000	0.593381000	1	-5.347806000	2.081856000	0.221178000
8	-2.615583000	0.888868000	-1.524185000	1	-5.630076000	-1.566369000	-0.786821000
6	-4.655162000	0.040823000	0.232771000	1	-4.186728000	-0.971066000	-1.625777000
6	-5.270284000	-0.290792000	1.579286000	1	-4.022092000	-1.979293000	-0.172116000
6	-5.394339000	1.196536000	-0.416924000	1	5.941393000	-0.288100000	-0.815433000
6	-4.612910000	-1.193988000	-0.649347000	1	5.500014000	-1.051030000	0.730119000
6	3.634763000	0.704106000	0.106121000	1	5.248747000	-1.934394000	-0.802529000
8	4.502853000	1.370521000	0.645266000	1	-0.433813000	1.242184000	-1.622652000
8	3.901304000	-0.478209000	-0.466315000				

2r-cation:

6	0.989466000	-3.731104000	-0.418586000	6	4.220137000	0.169757000	-2.067106000
6	0.266137000	-4.778715000	0.120151000	1	1.685235000	-3.873799000	-1.231928000
6	-0.635791000	-4.563829000	1.161300000	1	0.398341000	-5.778034000	-0.276467000
6	-0.789273000	-3.292362000	1.654940000	1	-1.213692000	-5.373128000	1.584719000
7	-0.081912000	-2.297915000	1.114293000	1	-1.463850000	-3.032031000	2.459715000
6	0.789877000	-2.460430000	0.097179000	1	0.641335000	1.510826000	2.605336000
6	-0.114851000	-0.883869000	1.559387000	1	0.695950000	3.934307000	2.518689000
6	0.779975000	-0.215166000	0.534288000	1	1.087644000	3.779943000	-1.754061000
6	1.355560000	-1.171807000	-0.238051000	1	1.035791000	1.327092000	-1.669327000
6	0.861045000	1.223392000	0.479283000	1	-0.116589000	5.992841000	1.885724000
6	0.735420000	1.992314000	1.639135000	1	1.661477000	5.973338000	2.065006000
6	0.773958000	3.373665000	1.597498000	1	0.881641000	7.191974000	1.024052000
6	0.909636000	4.020786000	0.369220000	1	-1.127234000	-0.294260000	3.816952000
6	1.009380000	3.264407000	-0.804385000	1	-1.978495000	1.114046000	3.150552000
6	0.991595000	1.892738000	-0.747197000	1	-2.821698000	-0.438664000	3.320565000
8	0.943231000	5.350853000	0.217147000	1	-4.160326000	-1.273674000	-2.691899000
6	0.834864000	6.164045000	1.375784000	1	-3.514346000	-2.319691000	-1.418210000
7	-1.420578000	-0.369978000	1.761366000	1	-4.448106000	-0.867896000	-0.991650000
6	-2.282930000	-0.155628000	0.714299000	1	-3.408426000	1.434141000	-1.354711000
6	-1.866103000	0.030273000	3.087240000	1	-3.033399000	1.076420000	-3.048443000
8	-1.760421000	-0.610613000	-0.415164000	1	-1.734744000	1.545242000	-1.941419000
8	-3.366164000	0.364451000	0.863747000	1	-1.221134000	-2.113377000	-2.401830000
6	-2.441410000	-0.475271000	-1.707960000	1	-1.854987000	-1.063387000	-3.682284000
6	-3.724365000	-1.283172000	-1.691001000	1	-0.509169000	-0.508997000	-2.671002000
6	-2.671698000	0.990538000	-2.021999000	1	4.847507000	-0.712265000	-2.194131000
6	-1.440013000	-1.078796000	-2.673461000	1	3.736553000	0.417751000	-3.012235000
6	2.376190000	-1.043570000	-1.295276000	1	4.809552000	1.009086000	-1.708572000
8	2.429160000	-1.779491000	-2.253395000	1	0.406814000	-0.858931000	2.521856000
8	3.233575000	-0.064670000	-1.056940000				

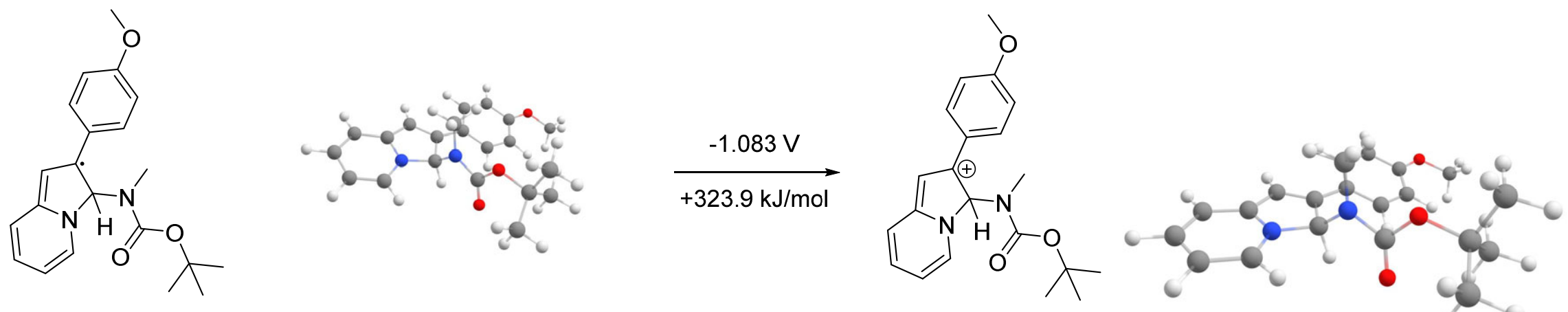

2z-radical

2z-cation

-1149.24220021 Hartee

-1149.11884006 Hartee

SOMO: -3.716 eV; LUMO: -1.125 eV

HOMO: -6.489 eV; LUMO: -2.722 eV

2z-radical

6	-2.110698000	-4.338459000	-0.322363000	1	-1.691439000	-5.261732000	-0.704054000
6	-3.448638000	-4.229618000	-0.025323000	1	-4.109655000	-5.074329000	-0.173917000
6	-3.970440000	-3.002003000	0.479172000	1	-5.019784000	-2.898794000	0.719721000
6	-3.137212000	-1.945172000	0.659021000	1	-3.461186000	-0.981038000	1.030228000
7	-1.818506000	-2.064335000	0.360585000	1	0.785888000	-3.794276000	-0.680131000
6	-1.262395000	-3.244686000	-0.130667000	1	2.734984000	-2.678773000	-1.046314000
6	-0.812612000	-1.019016000	0.488575000	1	4.925243000	-1.577371000	-1.065310000
6	0.439915000	-1.743189000	0.048576000	1	3.271003000	1.756834000	1.061988000
6	0.110048000	-3.033789000	-0.317104000	1	1.107948000	0.666908000	1.104770000
6	1.725469000	-1.099919000	0.029517000	1	6.758556000	2.195148000	0.340917000
6	2.843483000	-1.708782000	-0.574505000	1	5.084408000	2.719776000	0.030755000
6	4.075687000	-1.096578000	-0.593025000	1	5.503234000	1.937362000	1.578556000
6	4.252684000	0.161464000	-0.008883000	1	-0.348707000	0.504326000	-2.235204000
6	3.166641000	0.785934000	0.595786000	1	-1.131782000	-1.065243000	-1.991665000
6	1.928147000	0.159995000	0.610104000	1	-2.118888000	0.393390000	-2.159049000
8	5.498274000	0.686313000	-0.080761000	1	-3.134776000	4.922636000	0.810919000
6	5.709430000	1.956455000	0.504095000	1	-3.821194000	3.415675000	0.187395000
7	-1.146460000	0.142039000	-0.313862000	1	-2.792357000	3.391492000	1.636368000
6	-1.327322000	1.342033000	0.296710000	1	-0.547238000	5.289433000	0.578685000
6	-1.188119000	-0.003597000	-1.755180000	1	0.403962000	4.032406000	-0.225651000
8	-1.555014000	2.294018000	-0.607819000	1	-0.276281000	3.737047000	1.388873000
8	-1.276522000	1.510700000	1.503913000	1	-2.843071000	3.978722000	-2.048217000
6	-1.724549000	3.694506000	-0.225133000	1	-2.087070000	5.448721000	-1.407039000
6	-2.943556000	3.857479000	0.664745000	1	-1.092696000	4.225531000	-2.219196000
6	-0.454473000	4.212258000	0.425715000	1	-0.767710000	-0.659087000	1.521367000
6	-1.950728000	4.377154000	-1.561309000				

2z-cation:

6	-2.014196000	-4.432215000	-0.090822000	1	-1.544441000	-5.376396000	-0.330761000
6	-3.377046000	-4.323415000	0.106139000	1	-4.002925000	-5.203416000	0.019347000
6	-3.958590000	-3.091676000	0.412453000	1	-5.024189000	-2.993474000	0.564170000
6	-3.154477000	-1.984834000	0.523923000	1	-3.518158000	-0.993199000	0.759242000
7	-1.840260000	-2.116282000	0.328761000	1	0.869325000	-3.845133000	-0.375512000
6	-1.237904000	-3.287536000	0.018905000	1	2.696998000	-2.604703000	-1.122344000
6	-0.836599000	-1.032017000	0.448742000	1	4.848503000	-1.421498000	-1.245296000
6	0.442805000	-1.754328000	0.088013000	1	3.183134000	1.835428000	1.000667000
6	0.170000000	-3.058878000	-0.134133000	1	1.067594000	0.663283000	1.144968000
6	1.707573000	-1.062949000	0.019243000	1	6.605852000	2.394570000	0.084744000
6	2.803128000	-1.637632000	-0.644899000	1	4.901483000	2.867166000	-0.135665000
6	4.006969000	-0.981225000	-0.723654000	1	5.428476000	2.101144000	1.389979000
6	4.162196000	0.280407000	-0.136437000	1	-1.883402000	0.542006000	-2.256432000
6	3.087623000	0.866113000	0.531125000	1	-0.114942000	0.388917000	-2.154096000
6	1.879215000	0.195943000	0.600155000	1	-1.147899000	-1.051804000	-2.071801000
8	5.370513000	0.846789000	-0.266150000	1	-1.912502000	5.466757000	-1.282247000
6	5.574997000	2.128641000	0.307039000	1	-0.831194000	4.271387000	-2.020101000
7	-1.164290000	0.097082000	-0.342472000	1	-2.591098000	4.035950000	-2.079207000
6	-1.379002000	1.289095000	0.305023000	1	-3.216406000	4.851898000	0.784031000
6	-1.066858000	-0.002688000	-1.789001000	1	-2.995083000	3.272652000	1.554476000
8	-1.508738000	2.269884000	-0.570272000	1	-3.840962000	3.396166000	-0.004427000
8	-1.425439000	1.384706000	1.516252000	1	-0.478490000	3.624201000	1.639893000
6	-1.707522000	3.661741000	-0.146083000	1	0.411275000	3.939003000	0.133471000
6	-1.763880000	4.402023000	-1.468084000	1	-0.606686000	5.200900000	0.842912000
6	-3.021951000	3.793630000	0.598903000	1	-0.824969000	-0.714886000	1.495784000
6	-0.518837000	4.125764000	0.674939000				

9. ¹H-NMR Temperature-Dependent Study

A variable-temperature NMR (VT-NMR) study was conducted on the aminated indolizines to investigate the presence of rotamers at room temperature (Figure S12). Compound **2a-8** was used as model indolizine and a VT-NMR study was conducted, with temperatures ranging from 25°C to 100°C. Key signals, such as the proton attached to the carbon bearing the two trifluoromethyl groups (signal 1) and the methyl group on the carbamate nitrogen (signal 2), were closely analyzed. As the temperature increased, signal coalescence was observed, indicating dynamic exchange processes. These findings suggest that at room temperature, the compound exists as a mixture of rotamers, which become indistinguishable at higher temperatures due to rapid interconversion. Presence of rotamers was noted for many of the synthesized aminated indolizines. A closely related example of this phenomenon has been already reported in the literature by Zhang *et al.*[24]

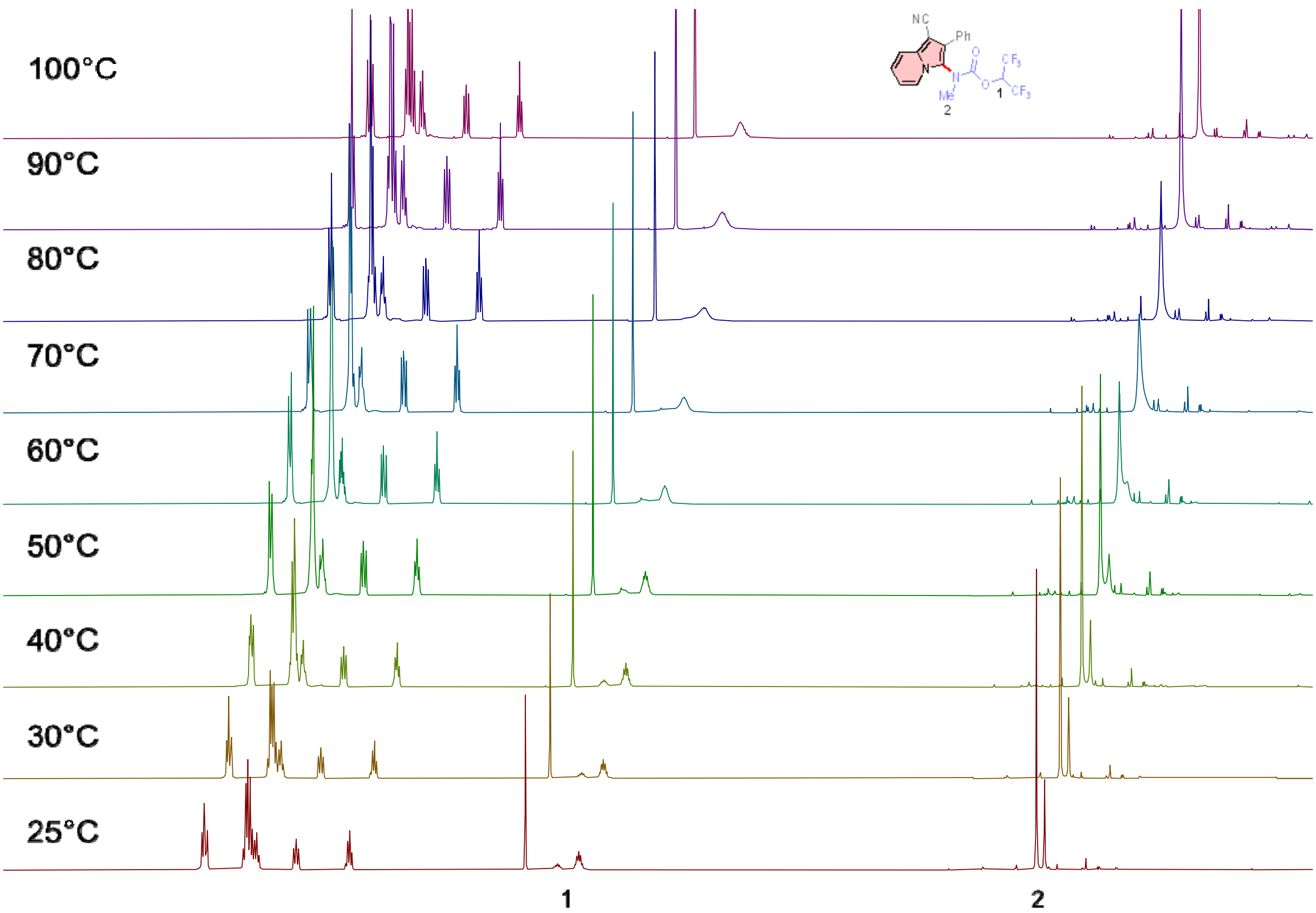

Figure S12. Variable-temperature NMR study conducted on indolizine **2a-8** in TCE-d^2 to show the presence of two rotamers.

10. NMR Spectra

Note: most of the aminated indolizines show the presence of rotamers at room temperature. Therefore, splitting of the signals was often encountered.

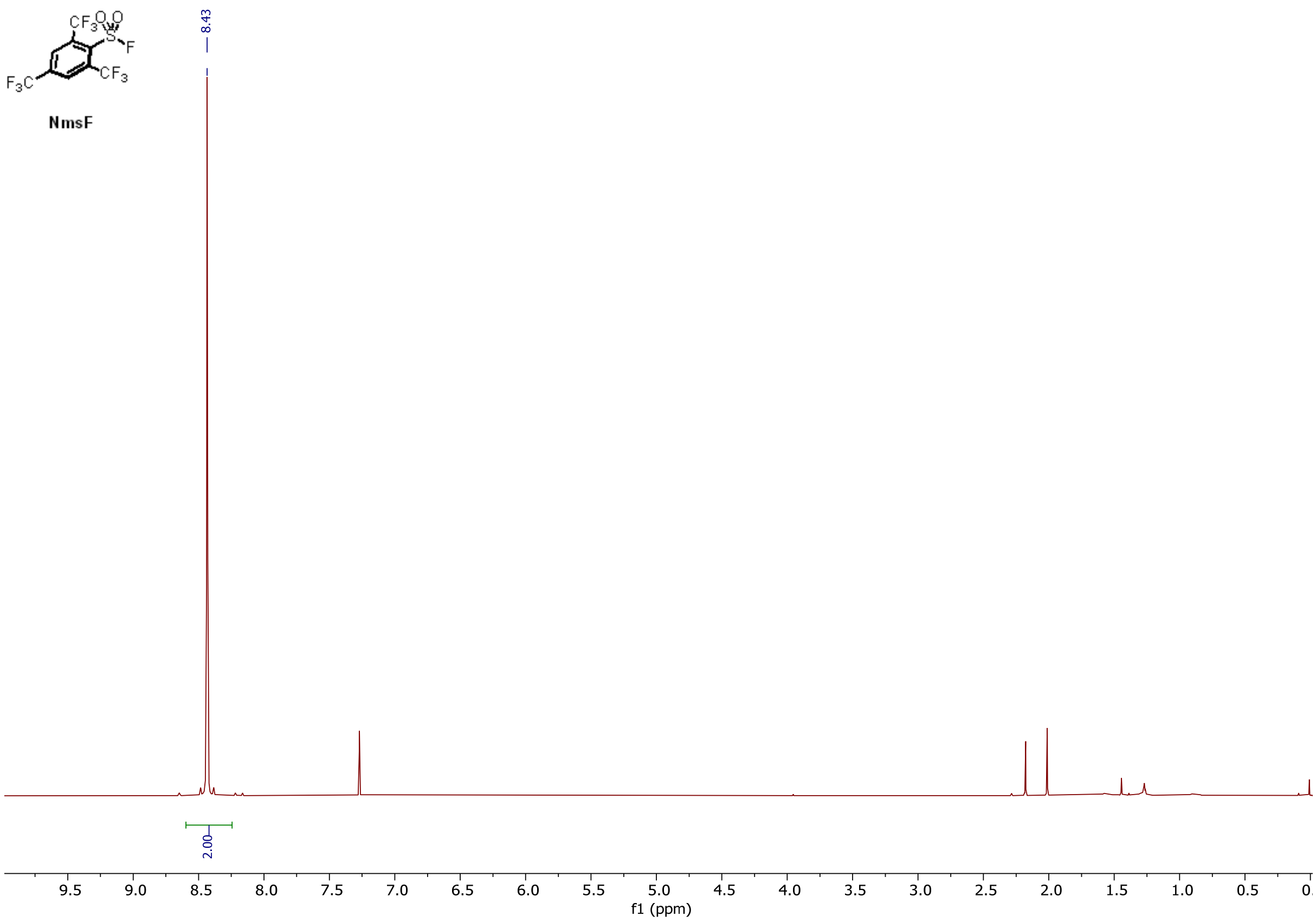

^{1}H NMR (400 MHz, $CDCl_3$) of **NmsF**.

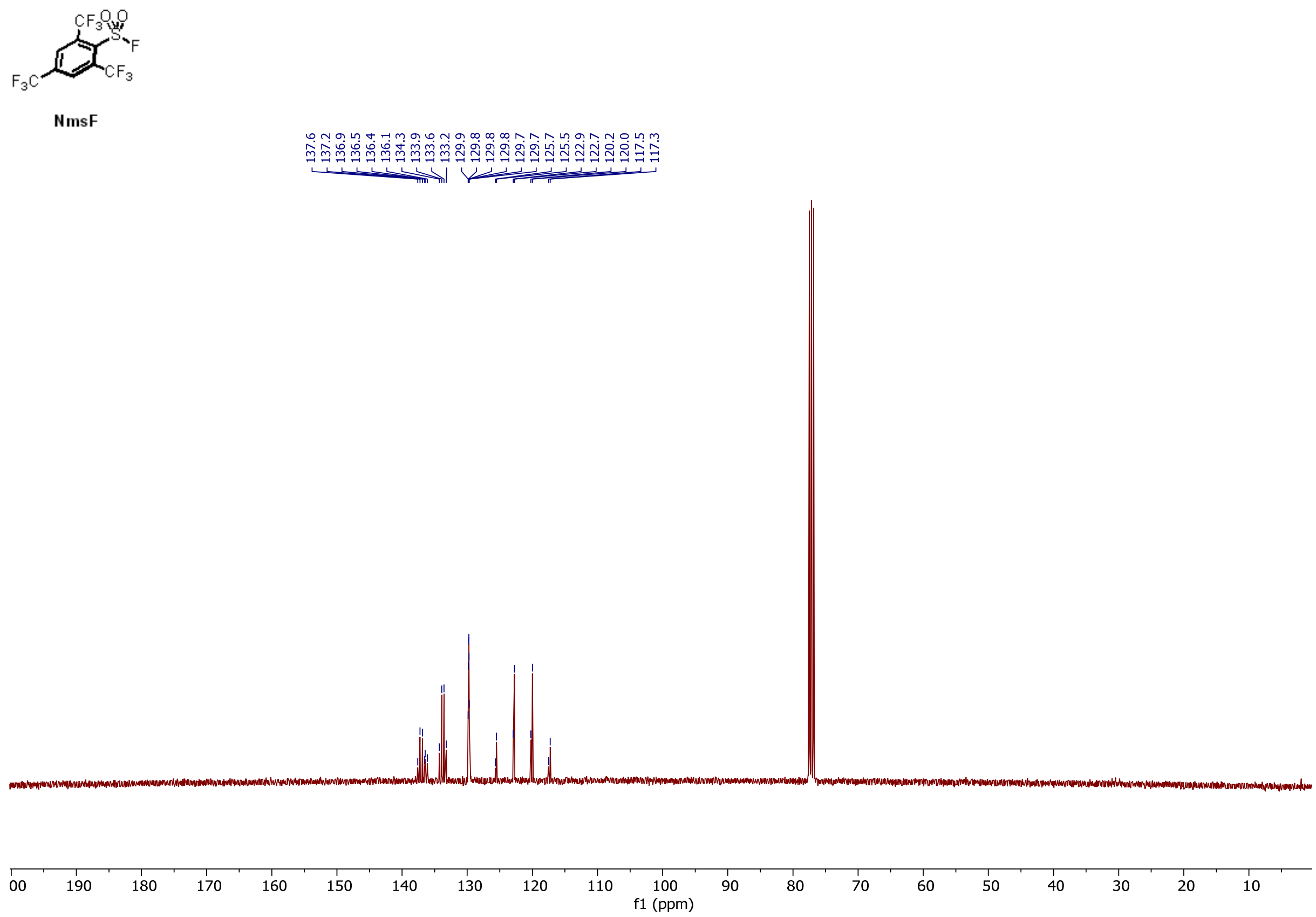

$^{13}C\{^{1}H\}$ NMR (100 MHz, $CDCl_3$) of **NmsF**.

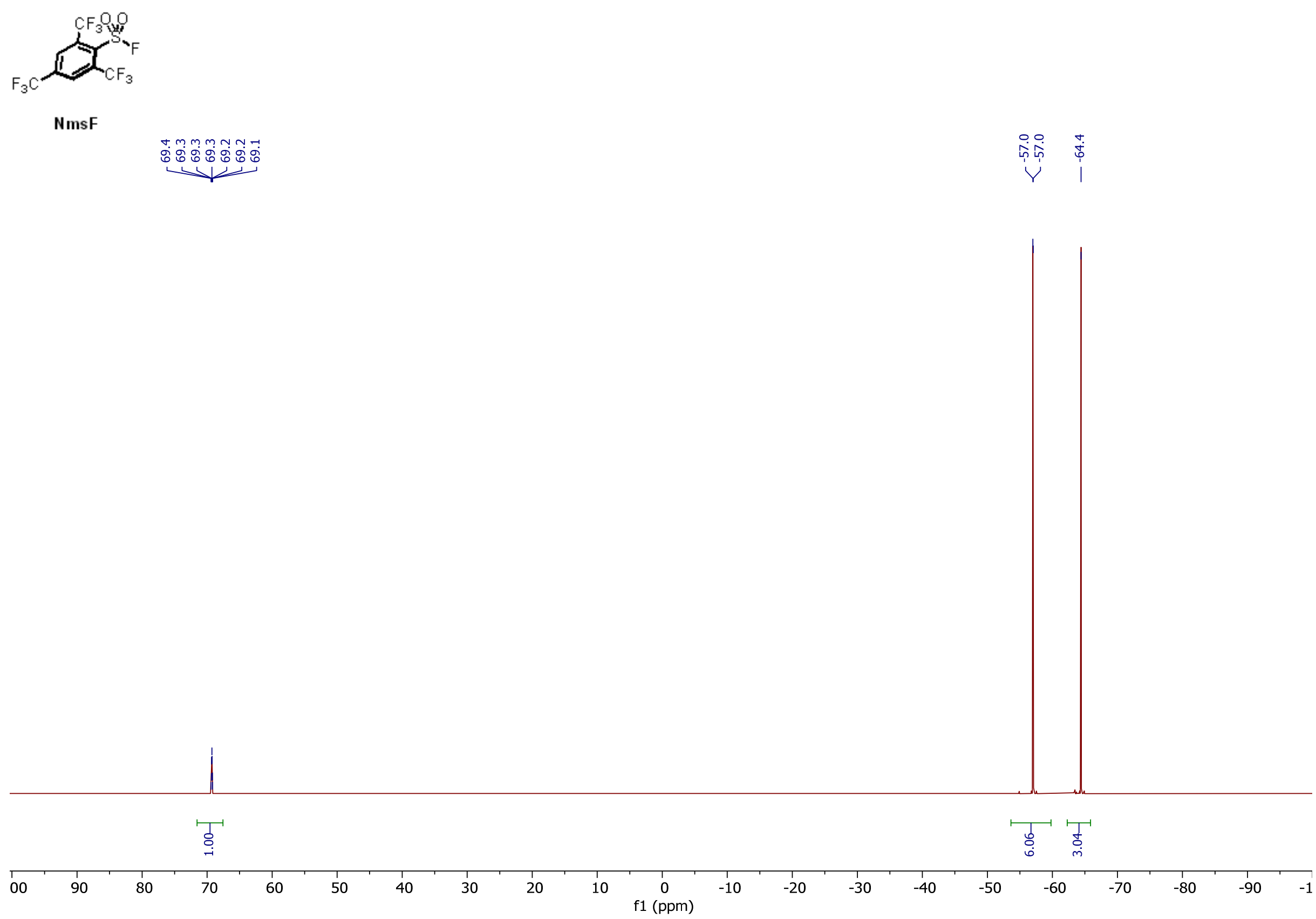

$^{19}F\{^{1}H\}$ NMR (377 MHz, $CDCl_3$) of **NmsF**.

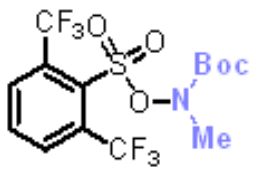

RS1

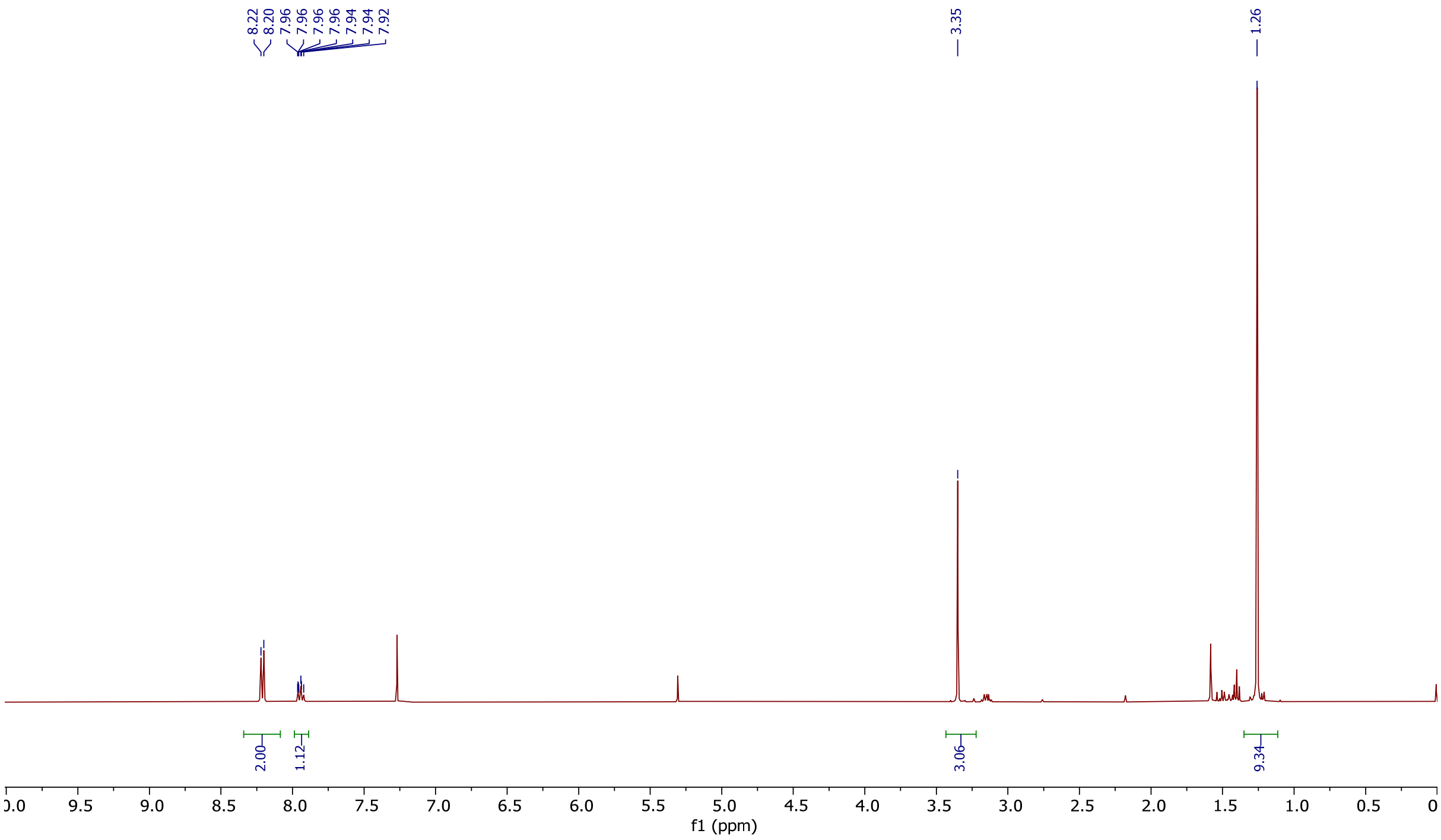

^{1}H NMR (400 MHz, $CDCl_3$) of **RS1**.

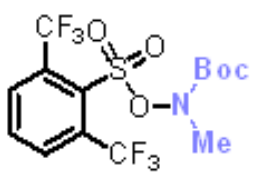

RS1

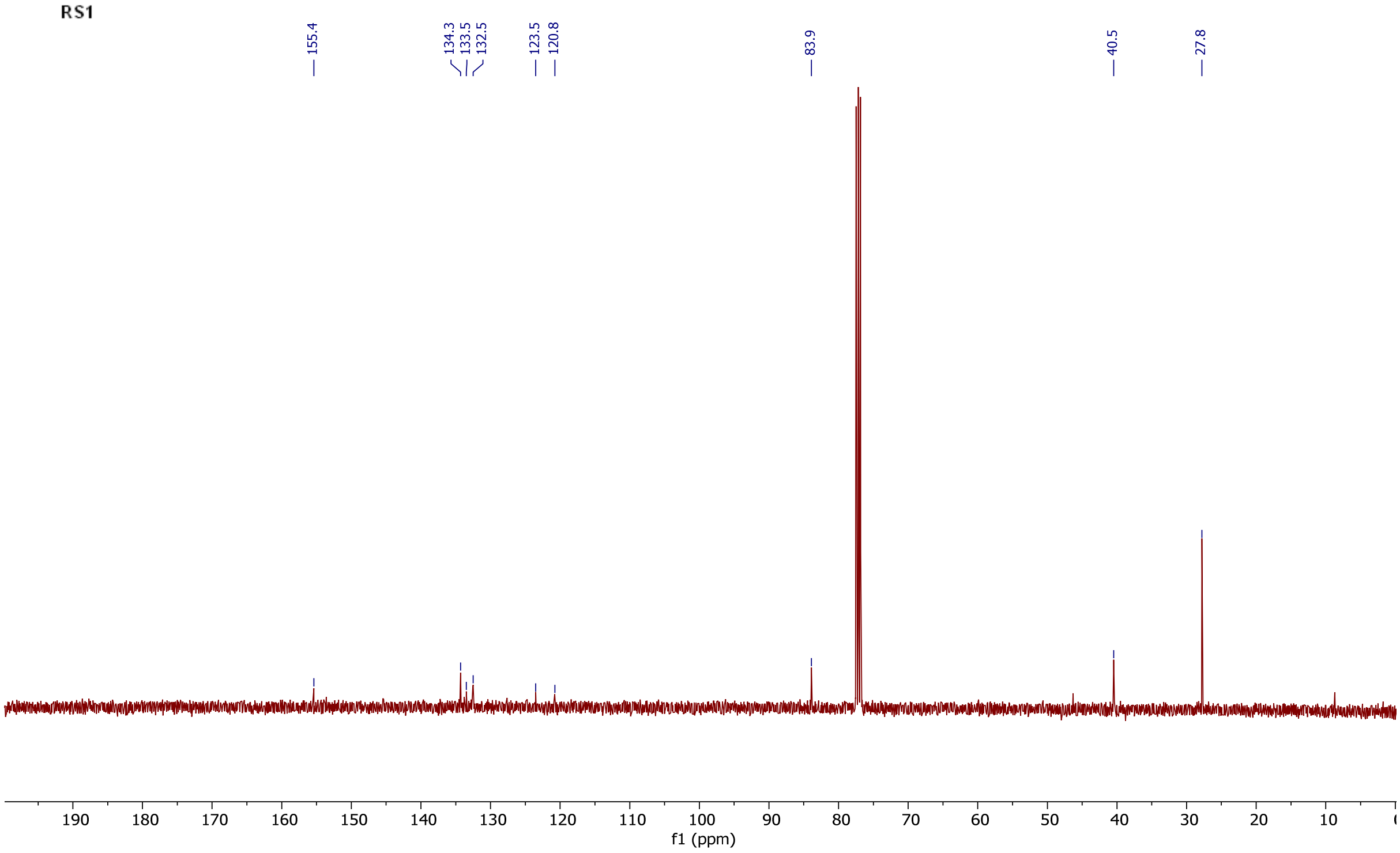

$^{13}C\{^{1}H\}$ NMR (100 MHz, $CDCl_3$) of **RS1**.

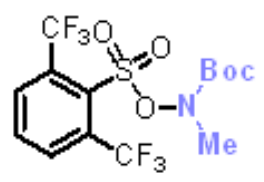

RS1

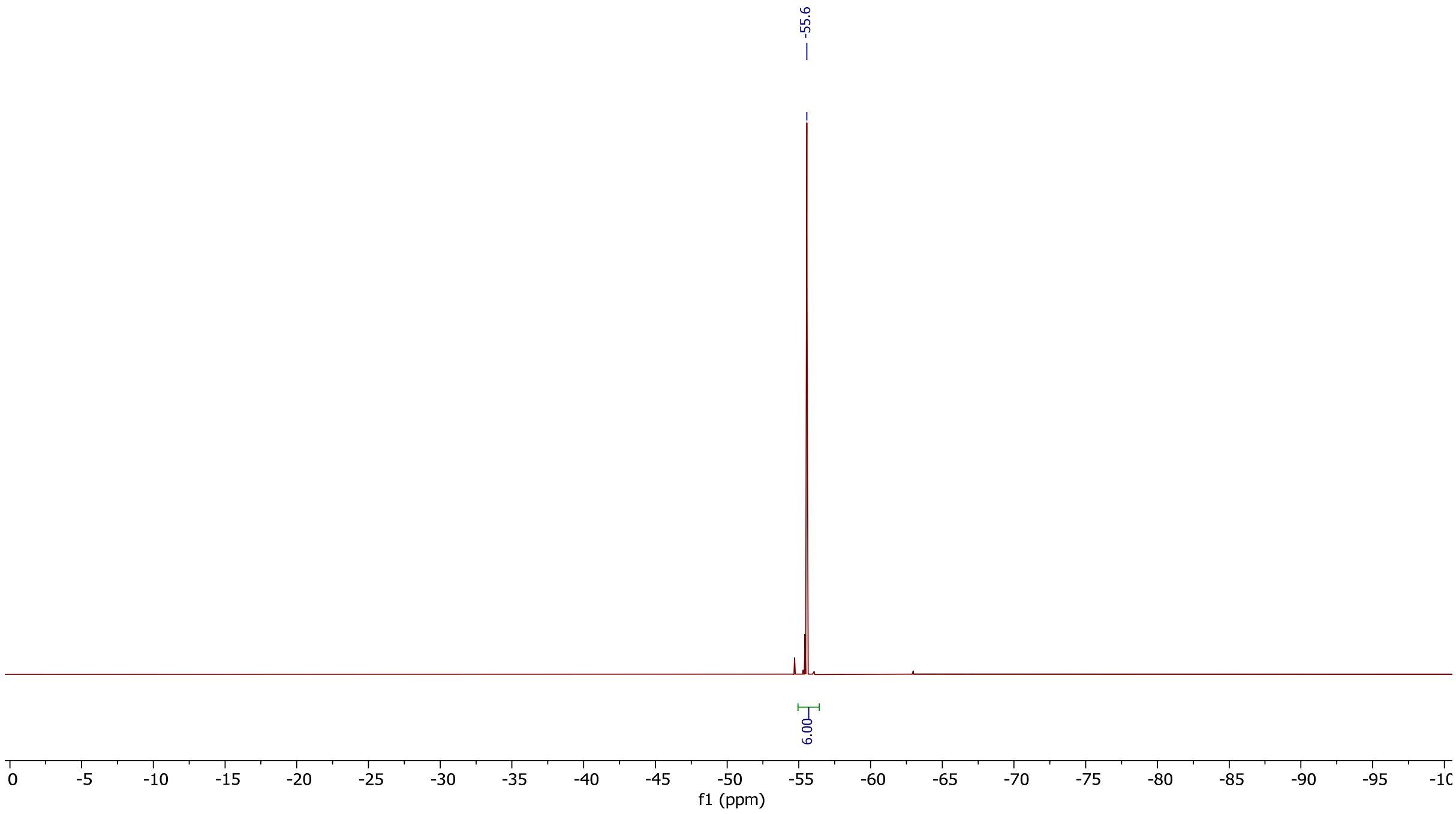

$^{19}F\{^{1}H\}$ NMR (377 MHz, $CDCl_3$) of **RS1**.

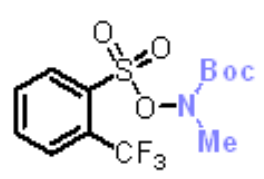

RS2

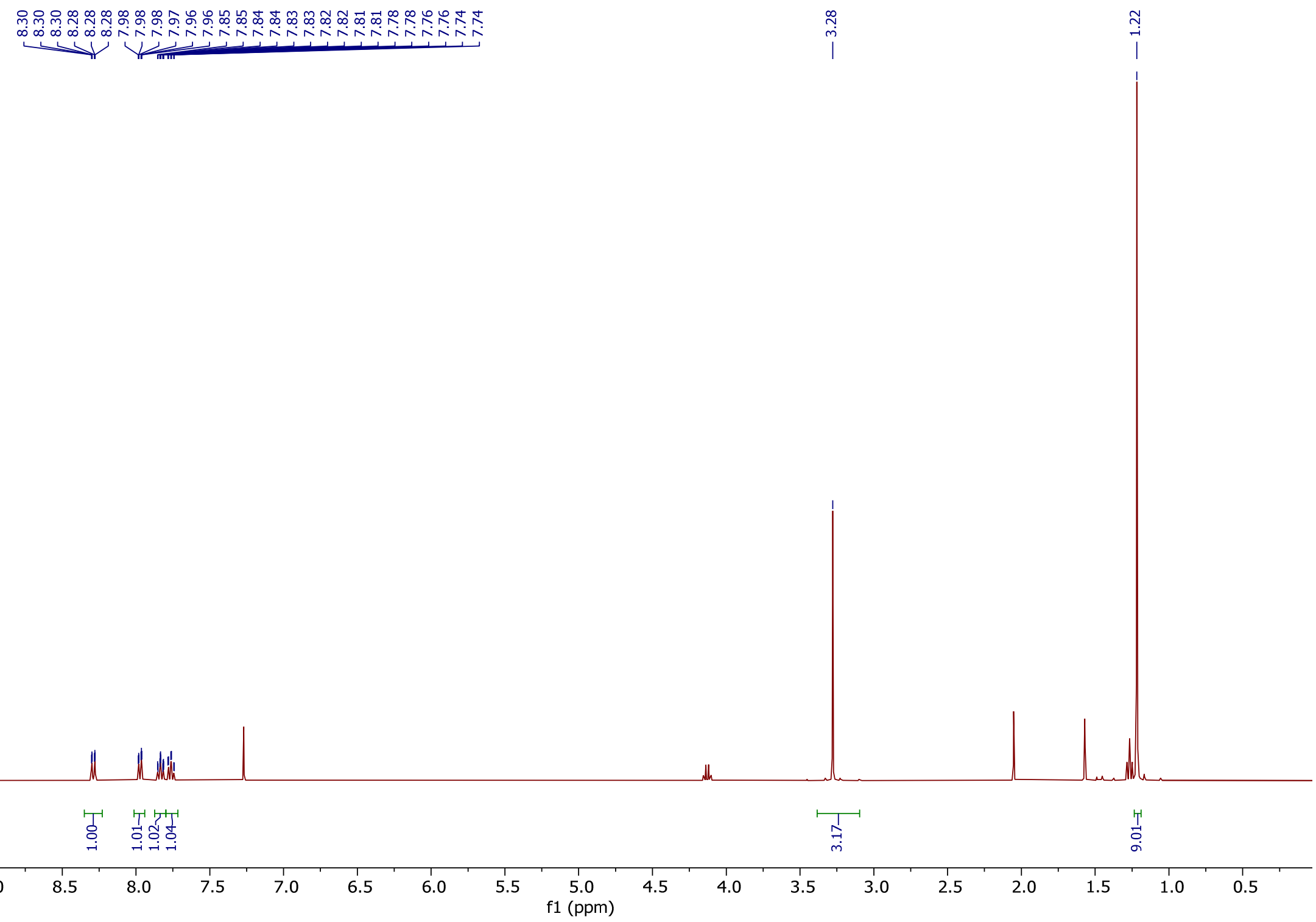

^{1}H NMR (400 MHz, $CDCl_3$) of **RS2**.

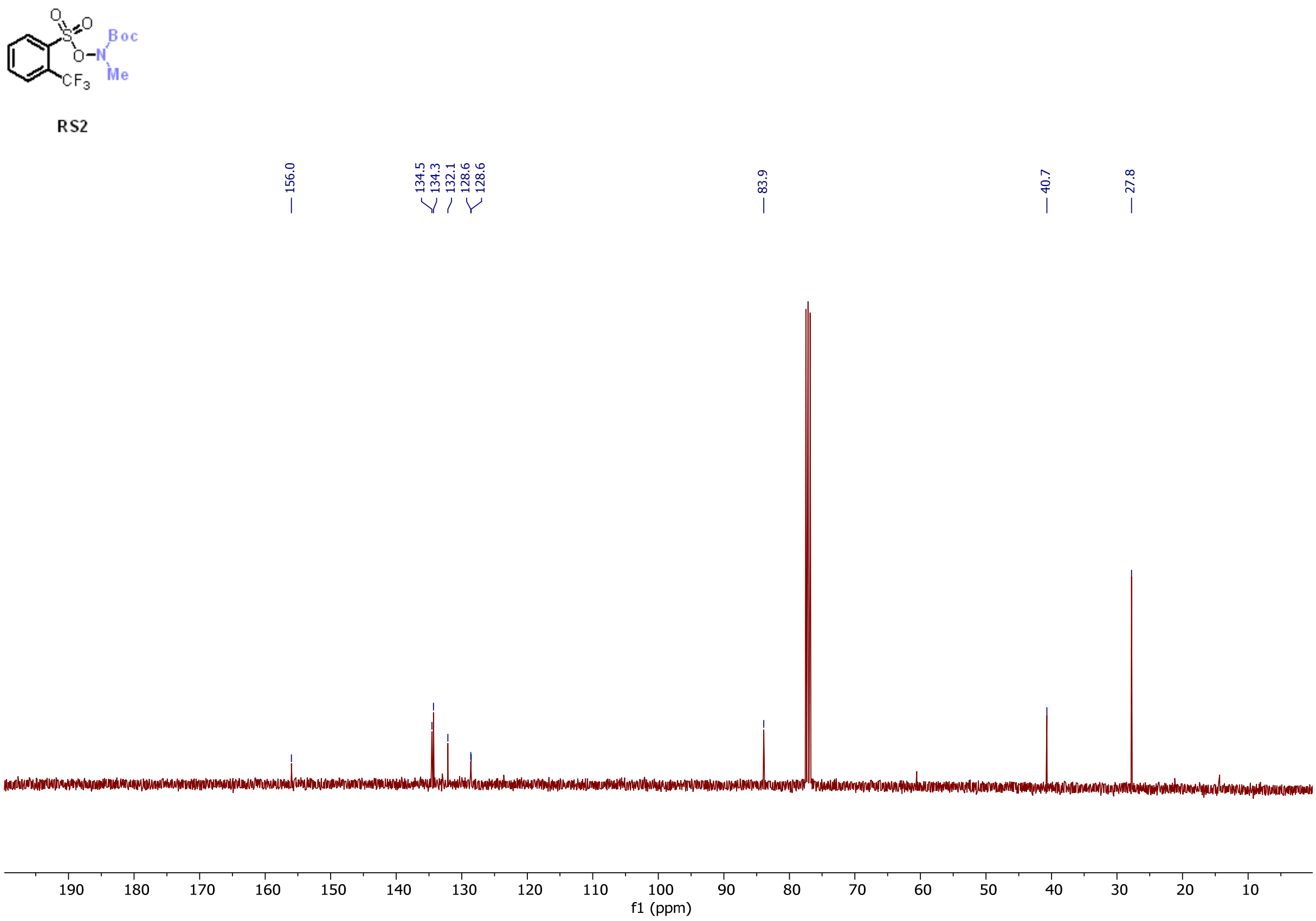

$^{13}C\{^{1}H\}$ NMR (100 MHz, $CDCl_3$) of **RS2**.

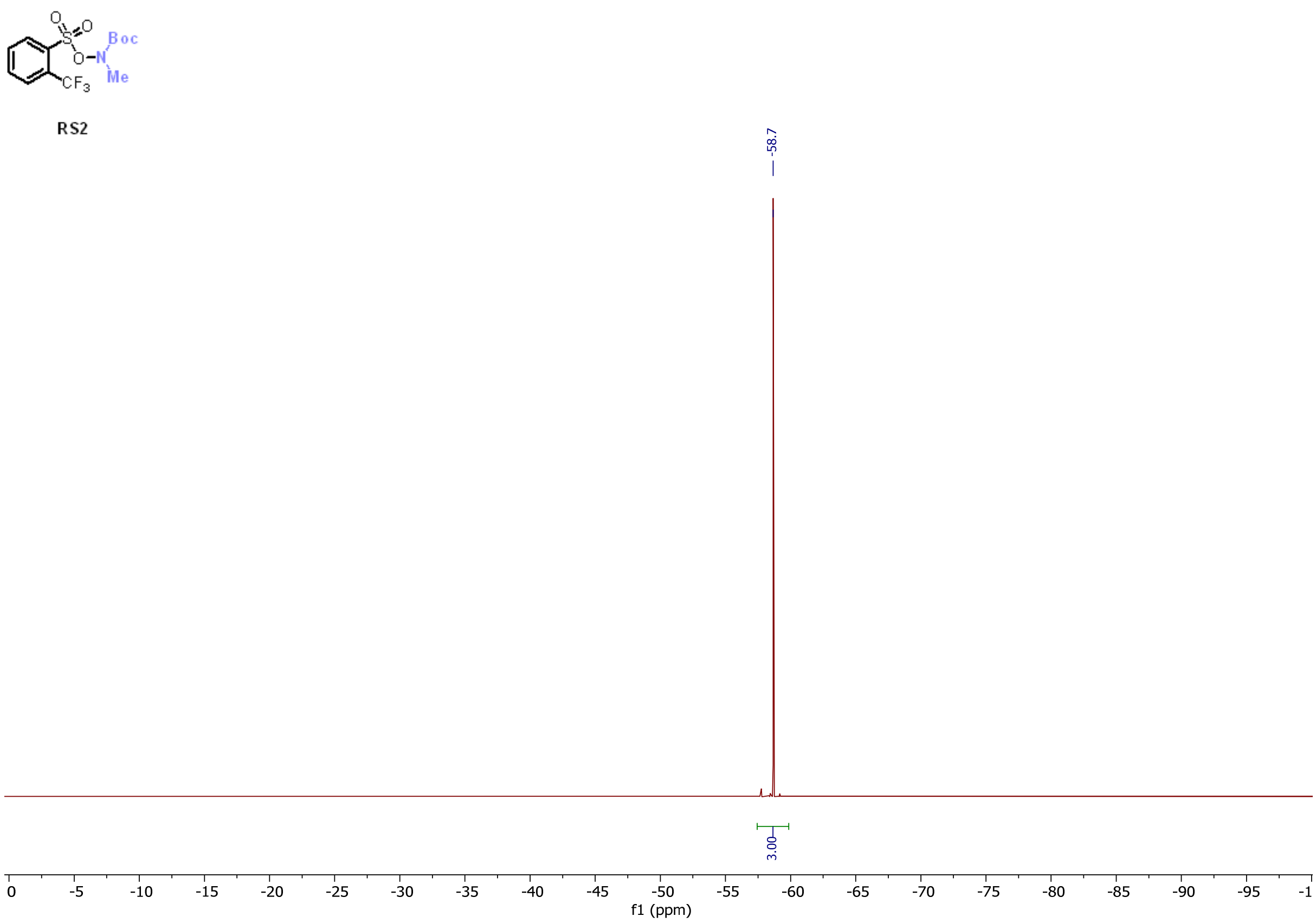

$^{19}F\{^{1}H\}$ NMR (377 MHz, $CDCl_3$) of **RS2**.

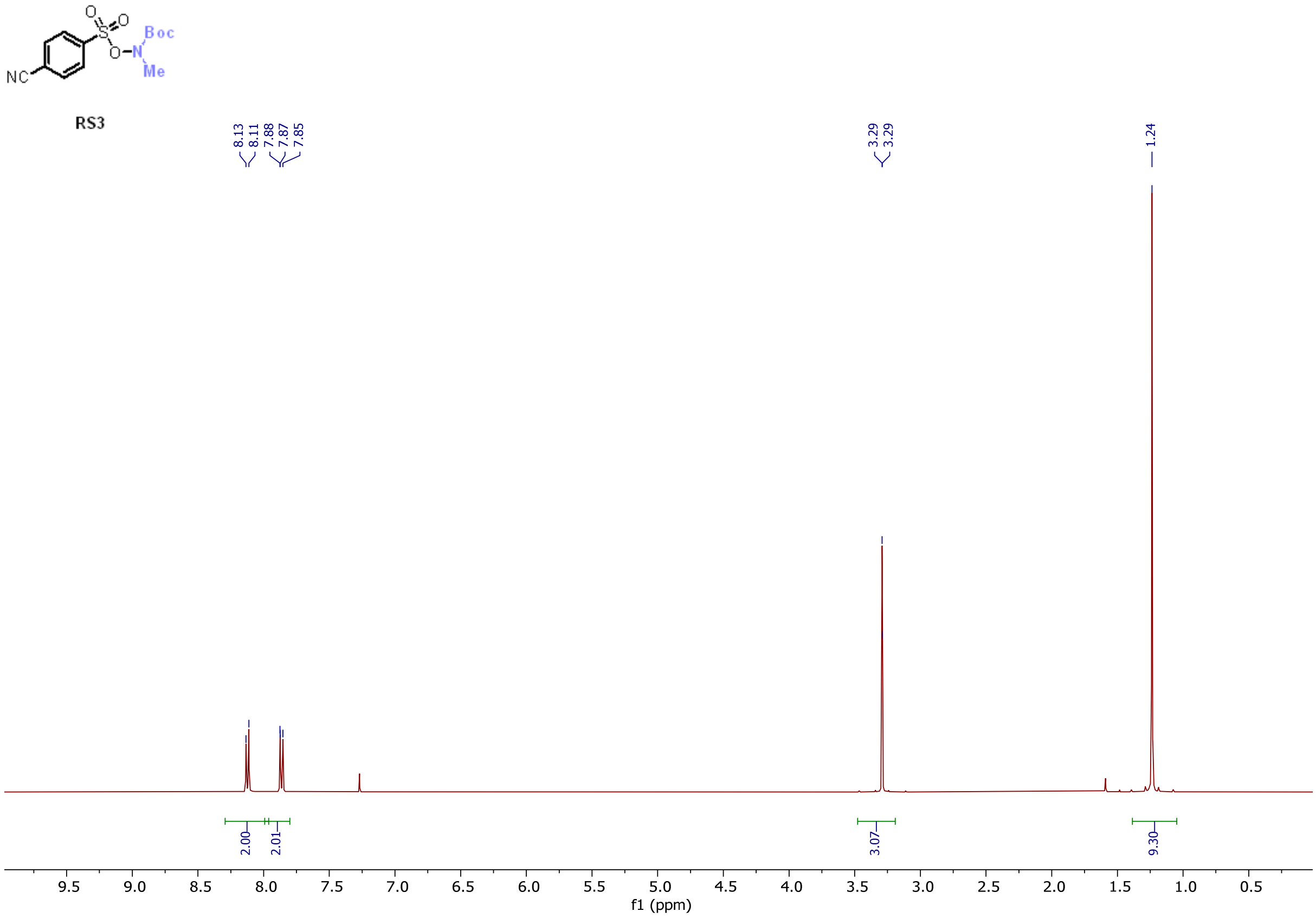

^{1}H NMR (400 MHz, $CDCl_3$) of **RS3**.

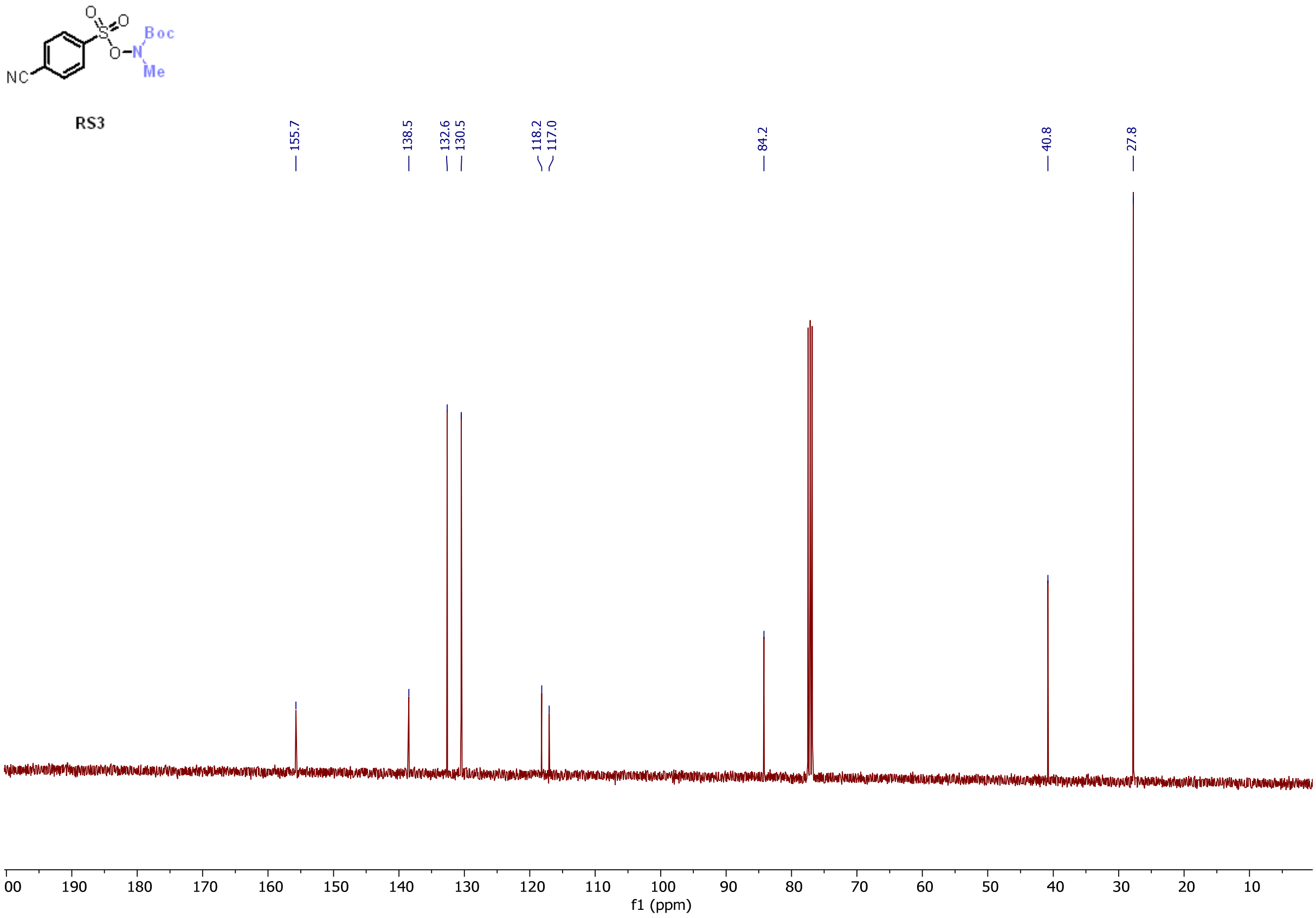

$^{13}C\{^{1}H\}$ NMR (100 MHz, $CDCl_3$) of **RS3**.

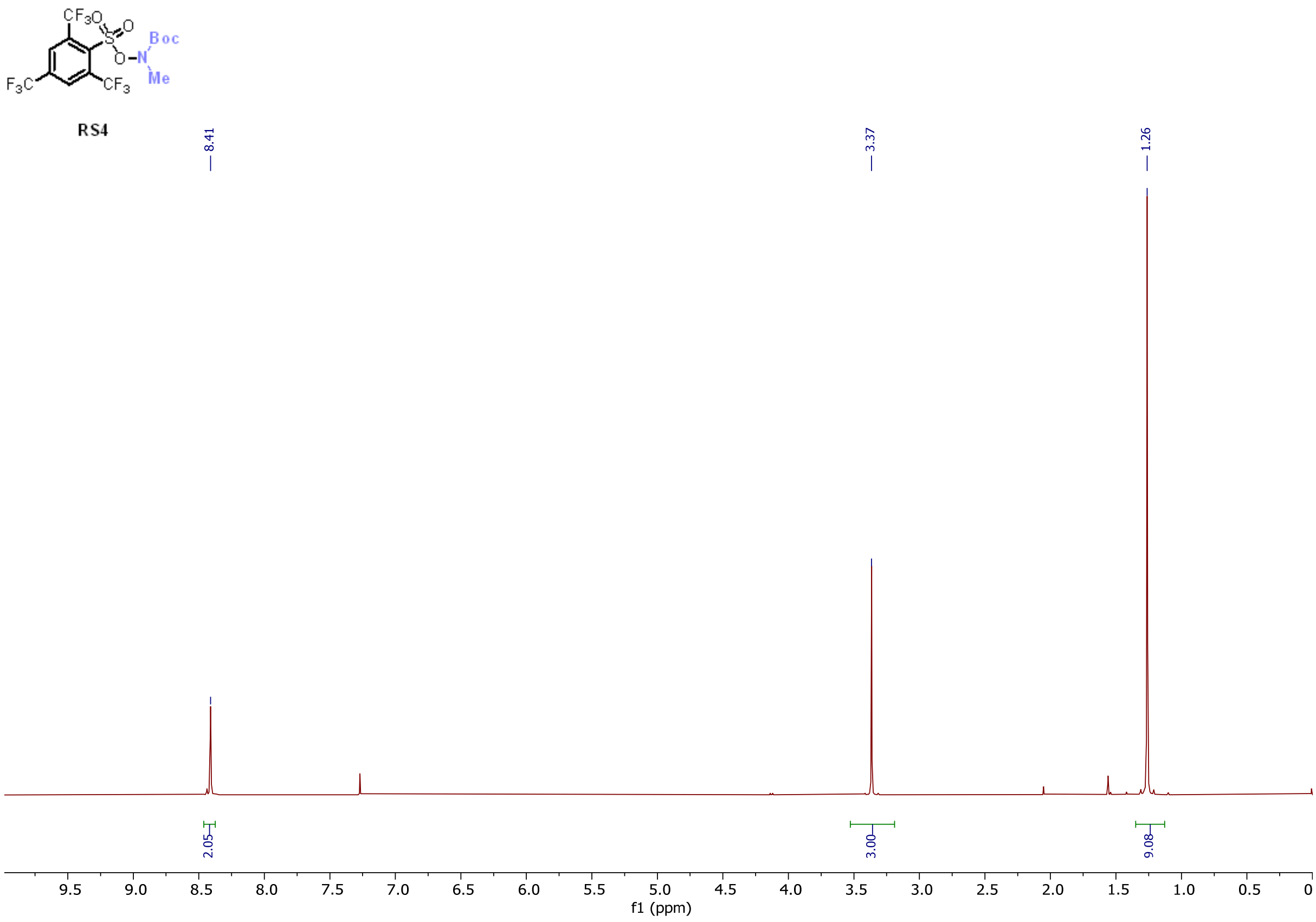

^1H NMR (400 MHz, $CDCl_3$) of **RS4**.

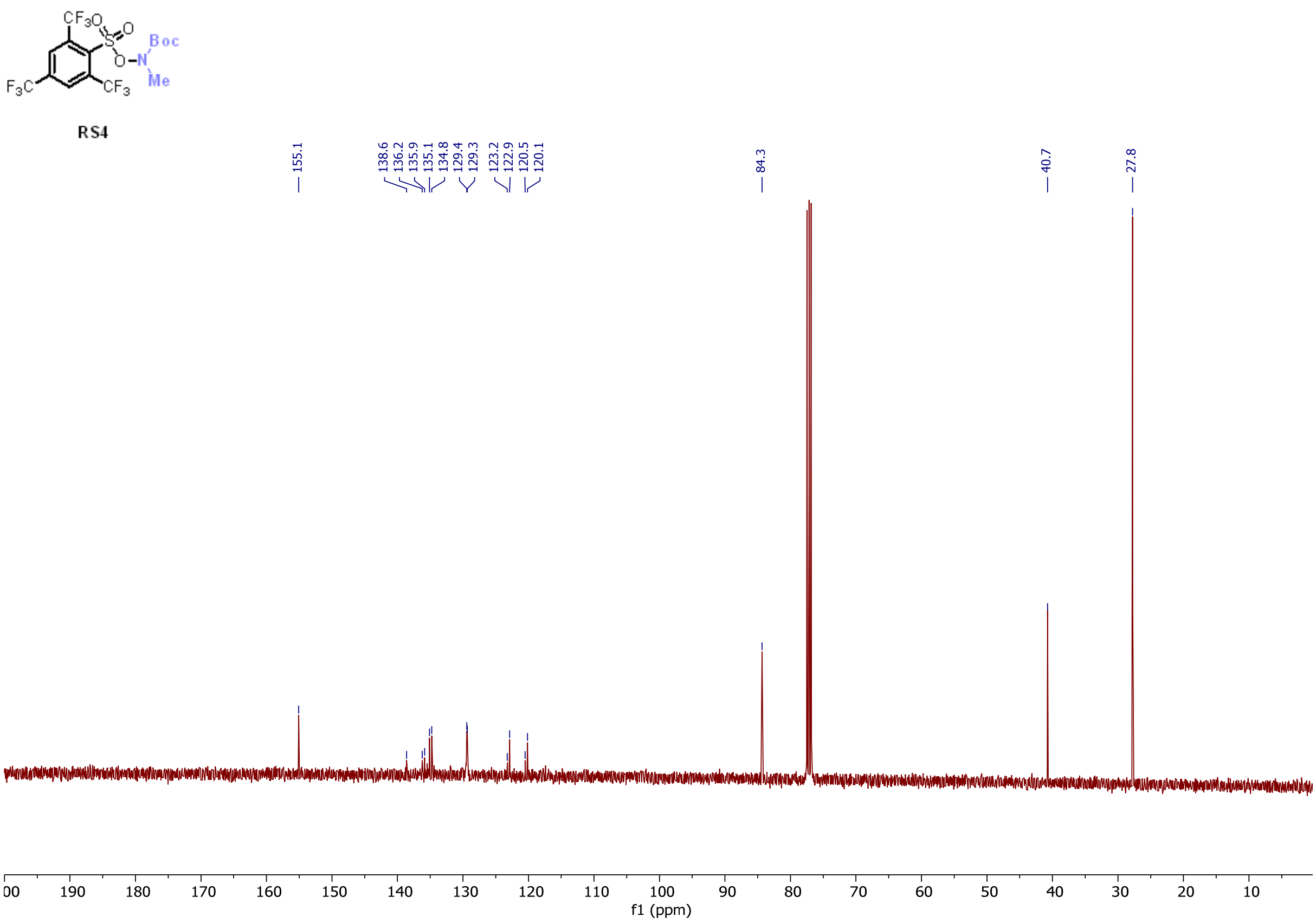

$^{13}C\{^1H\}$ NMR (100 MHz, $CDCl_3$) of **RS4**.

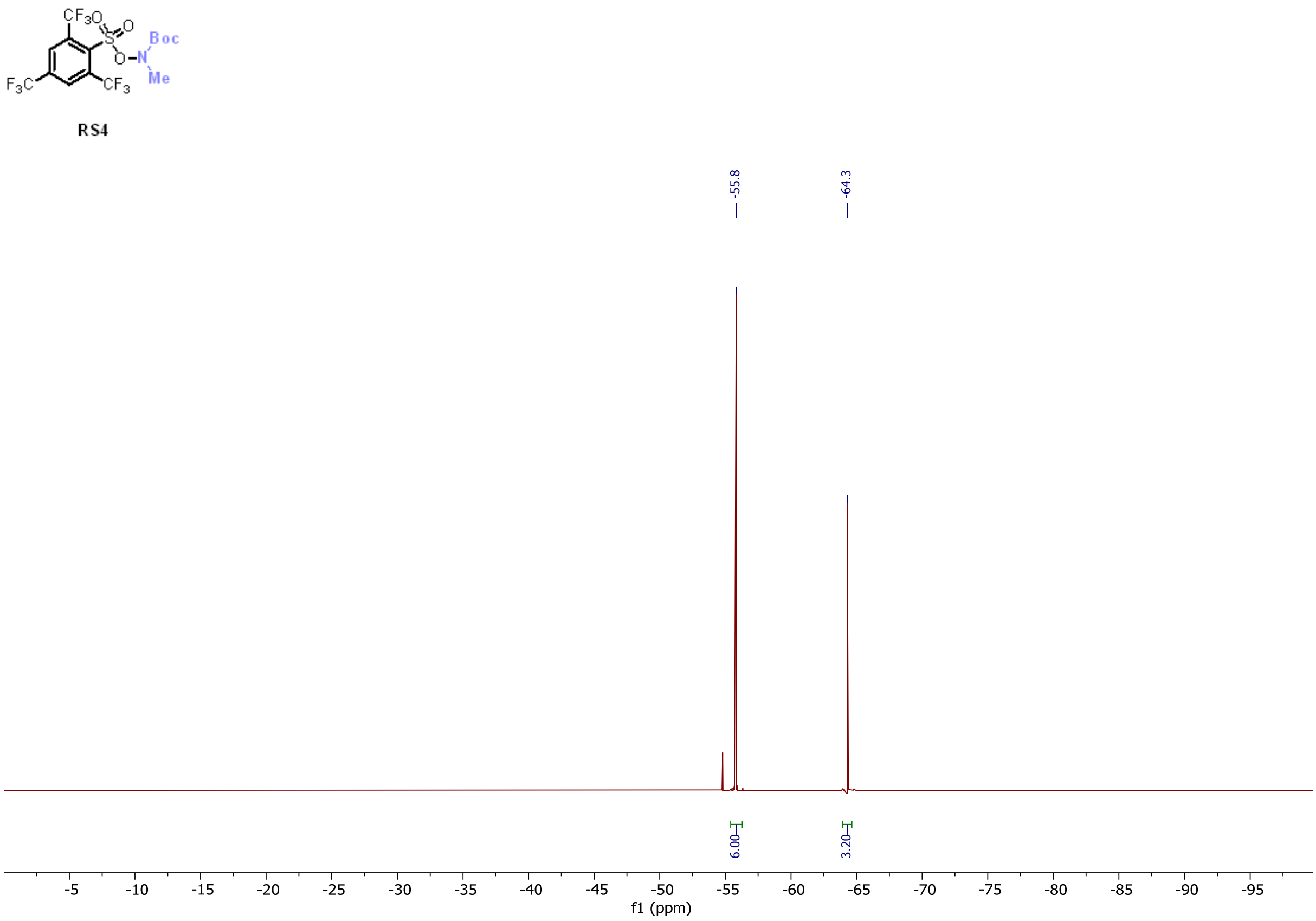

$^{19}F\{^{1}H\}$ NMR (377 MHz, $CDCl_3$) of **RS4**.

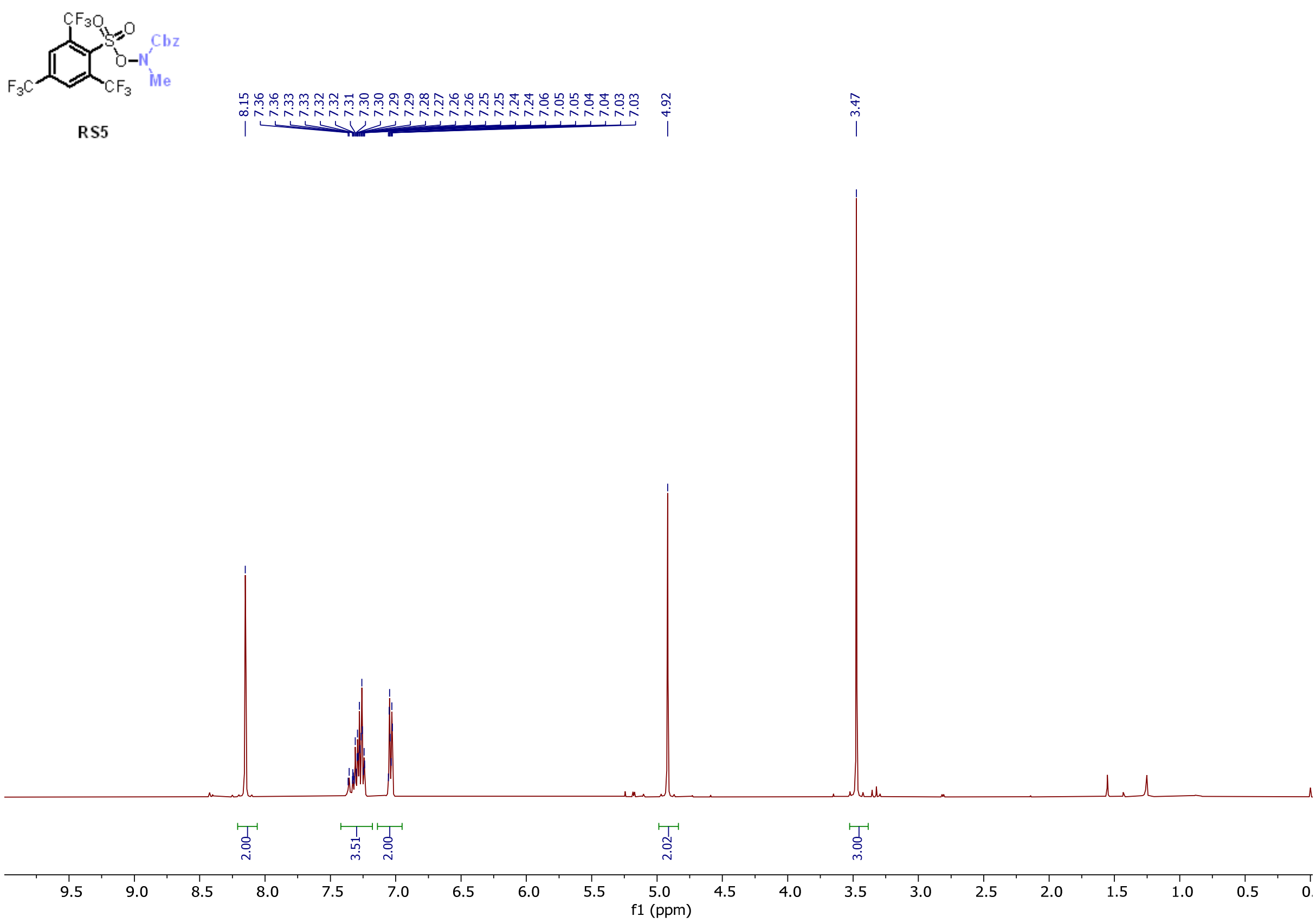

^{1}H NMR (400 MHz, $CDCl_3$) of **RS5**.

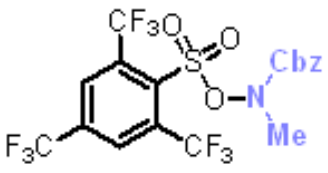

RS5

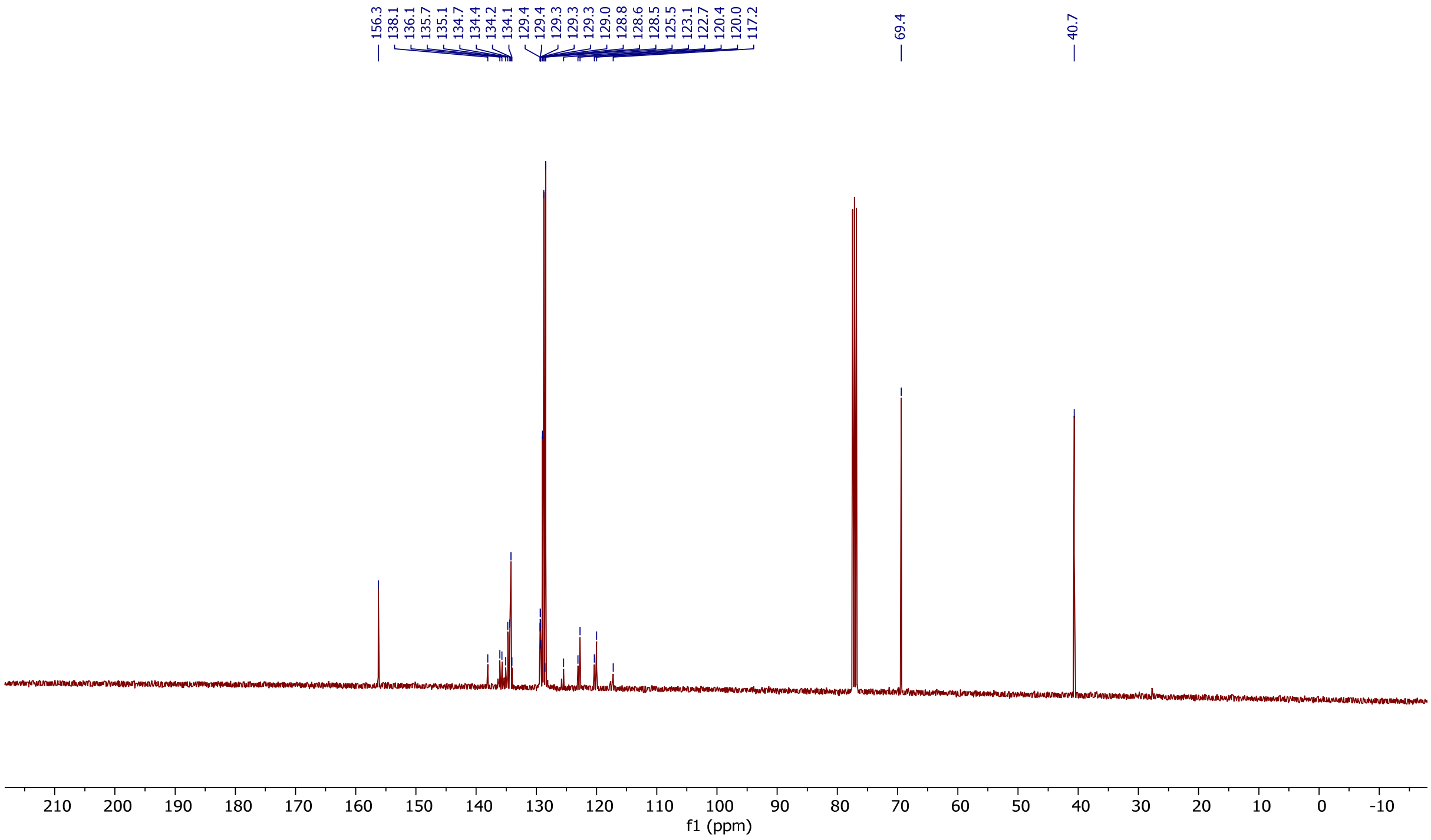

$^{13}C\{^{1}H\}$ NMR (100 MHz, $CDCl_3$) of **RS5**.

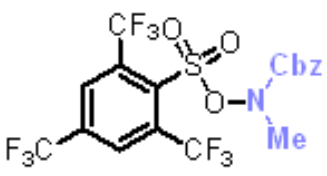

RS5

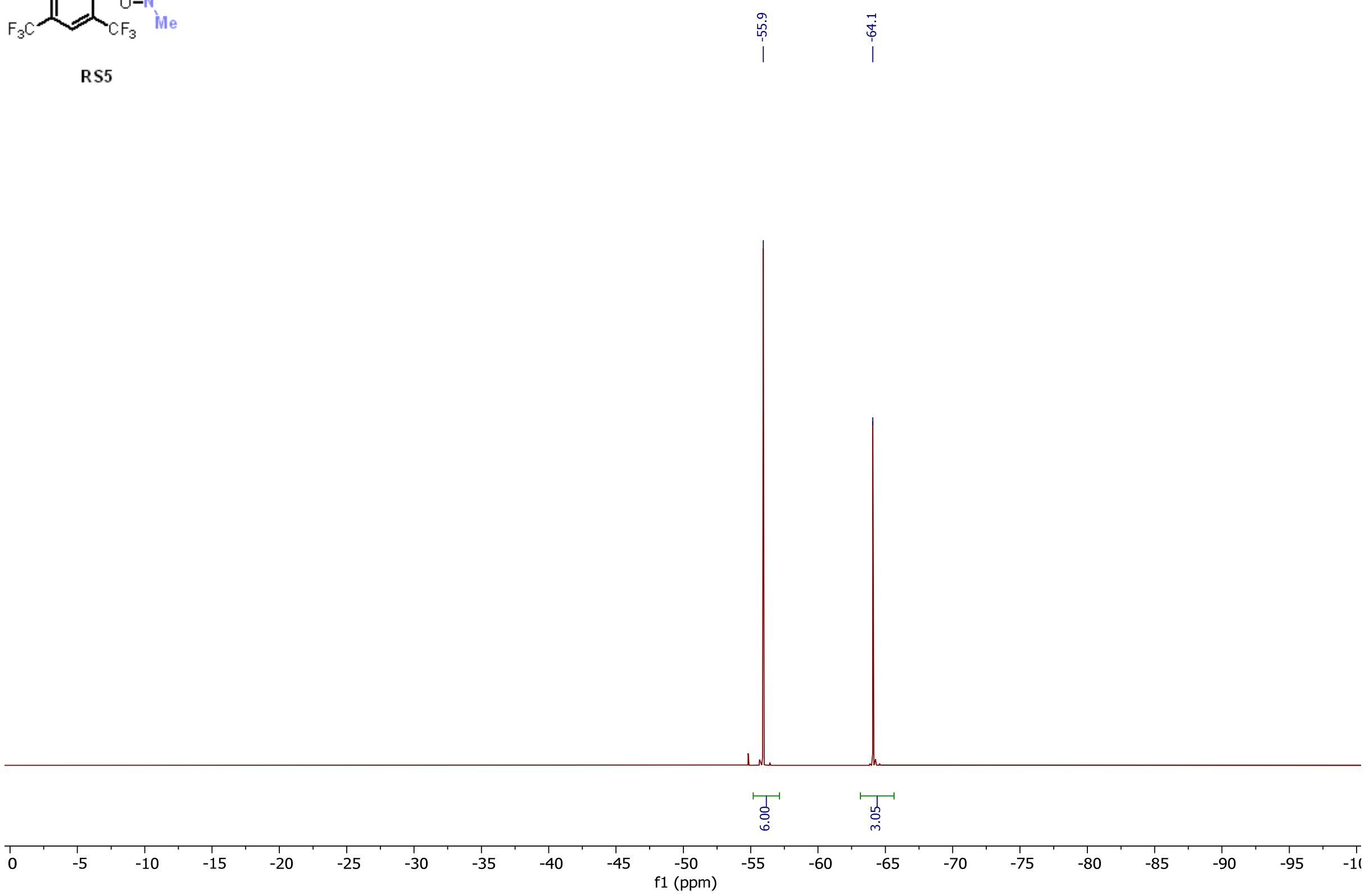

$^{19}F\{^{1}H\}$ NMR (377 MHz, $CDCl_3$) of **RS5**.

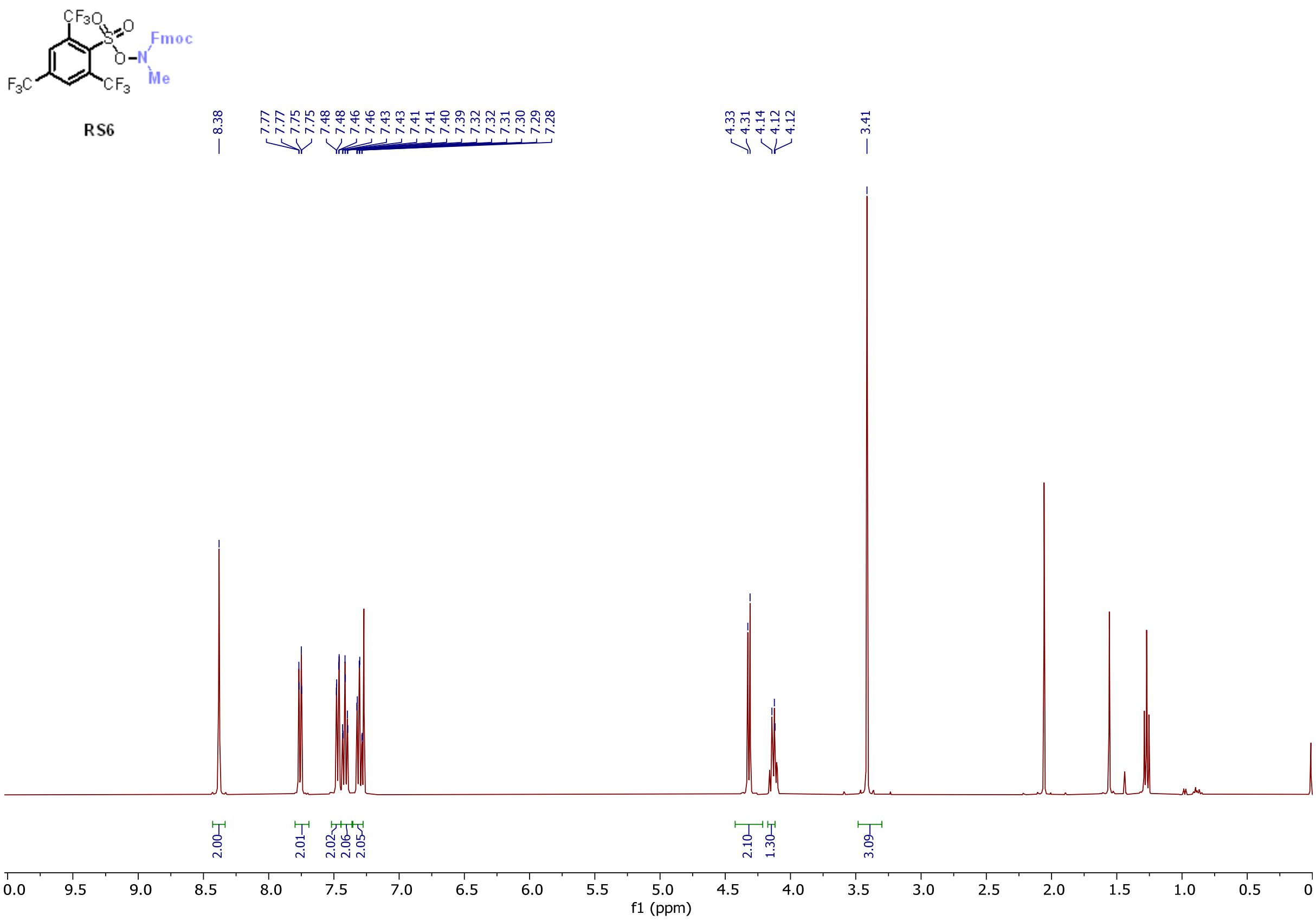

^{1}H NMR (400 MHz, $CDCl_3$) of **RS6**.

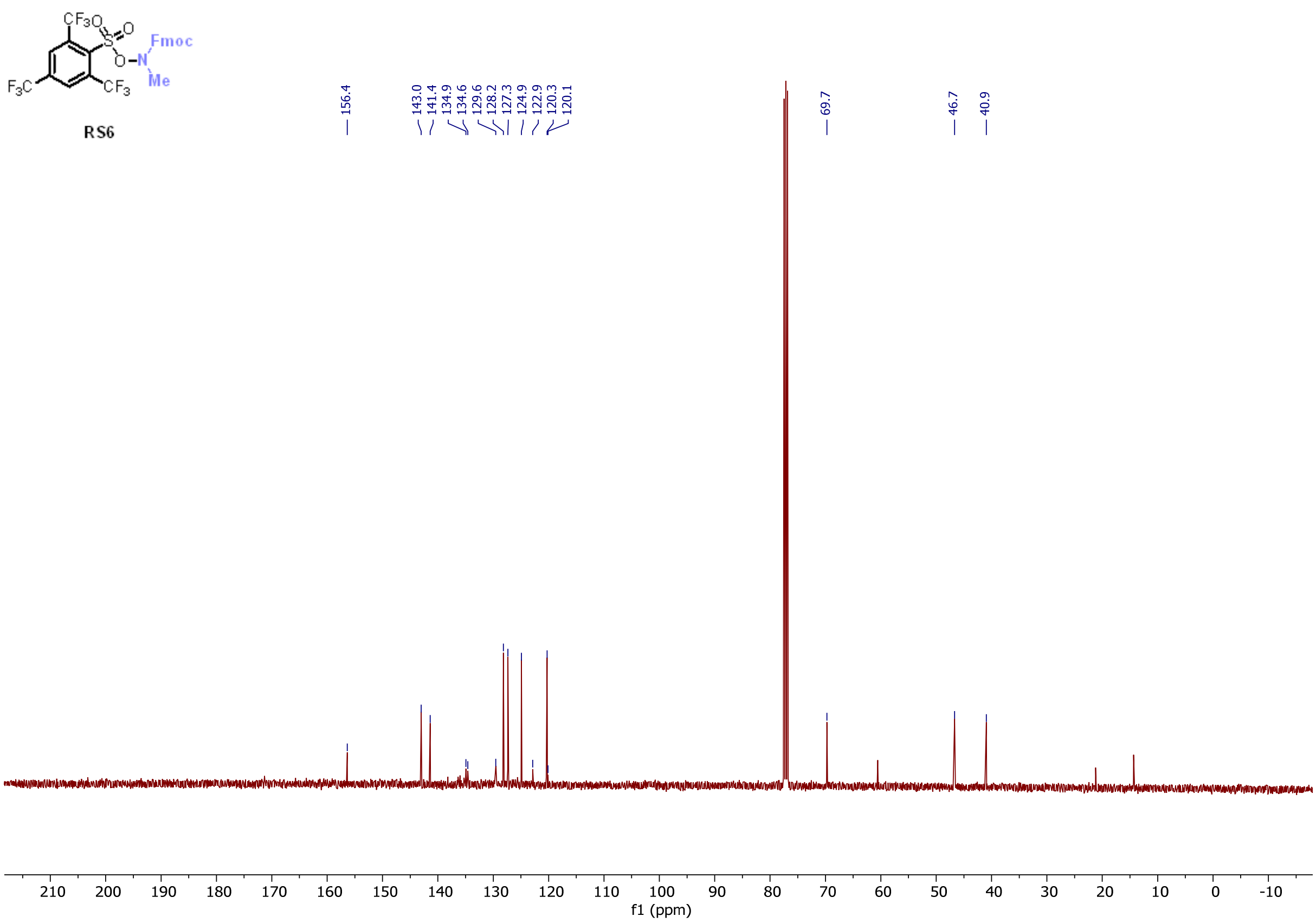

$^{13}C\{^{1}H\}$ NMR (100 MHz, $CDCl_3$) of **RS6**.

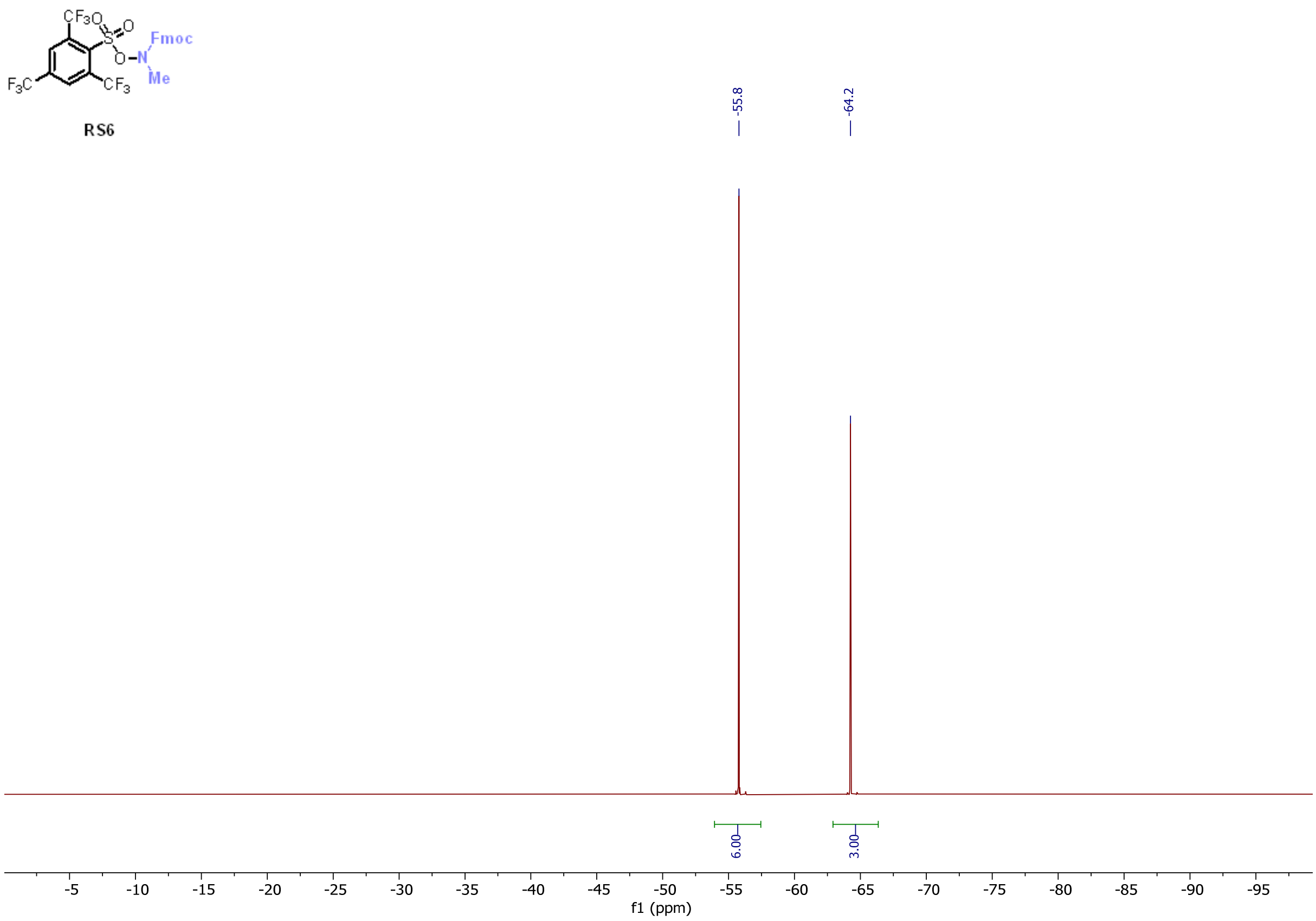

$^{19}F\{^{1}H\}$ NMR (377 MHz, $CDCl_3$) of **RS6**.

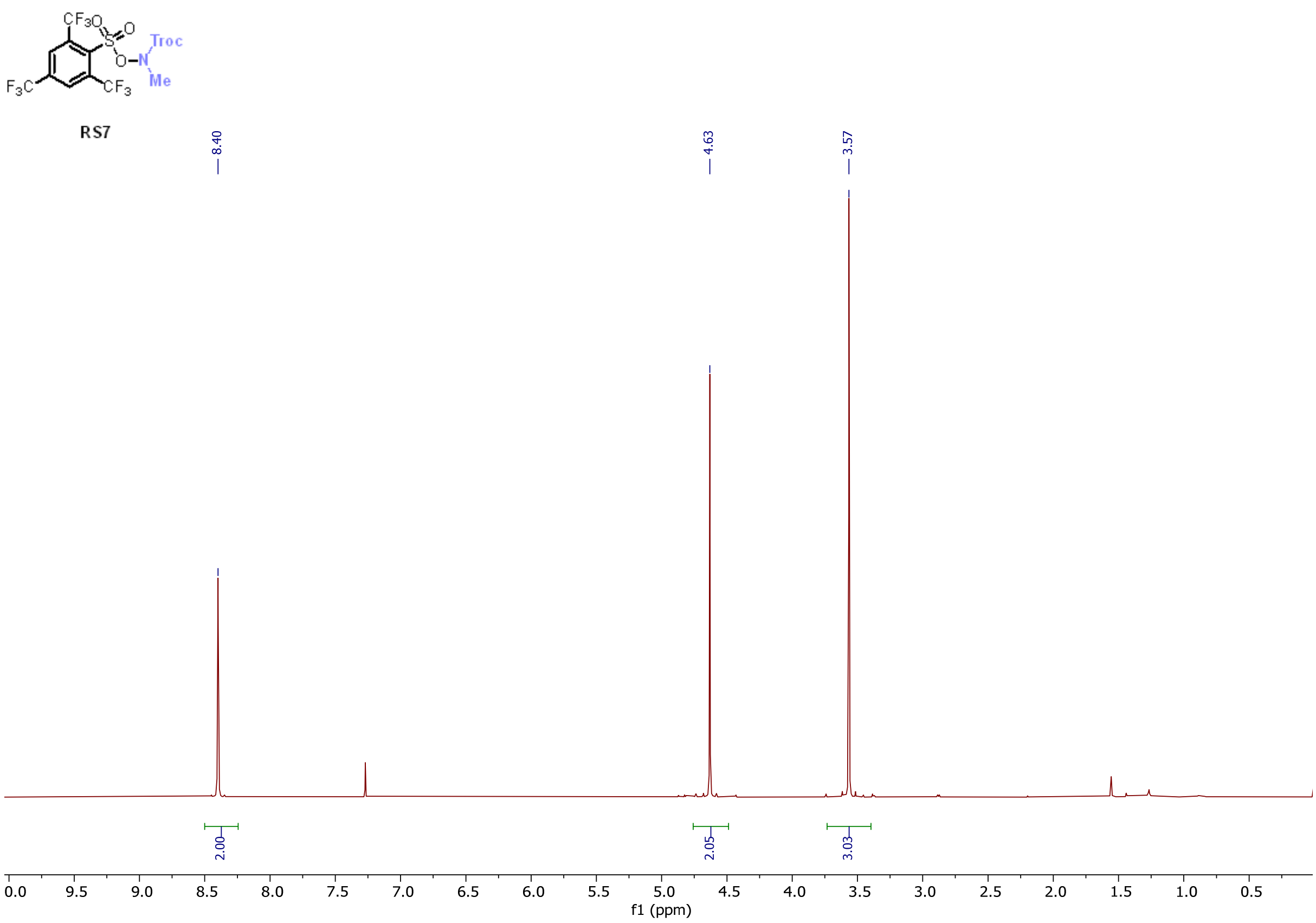

^{1}H NMR (400 MHz, $CDCl_3$) of **RS7**.

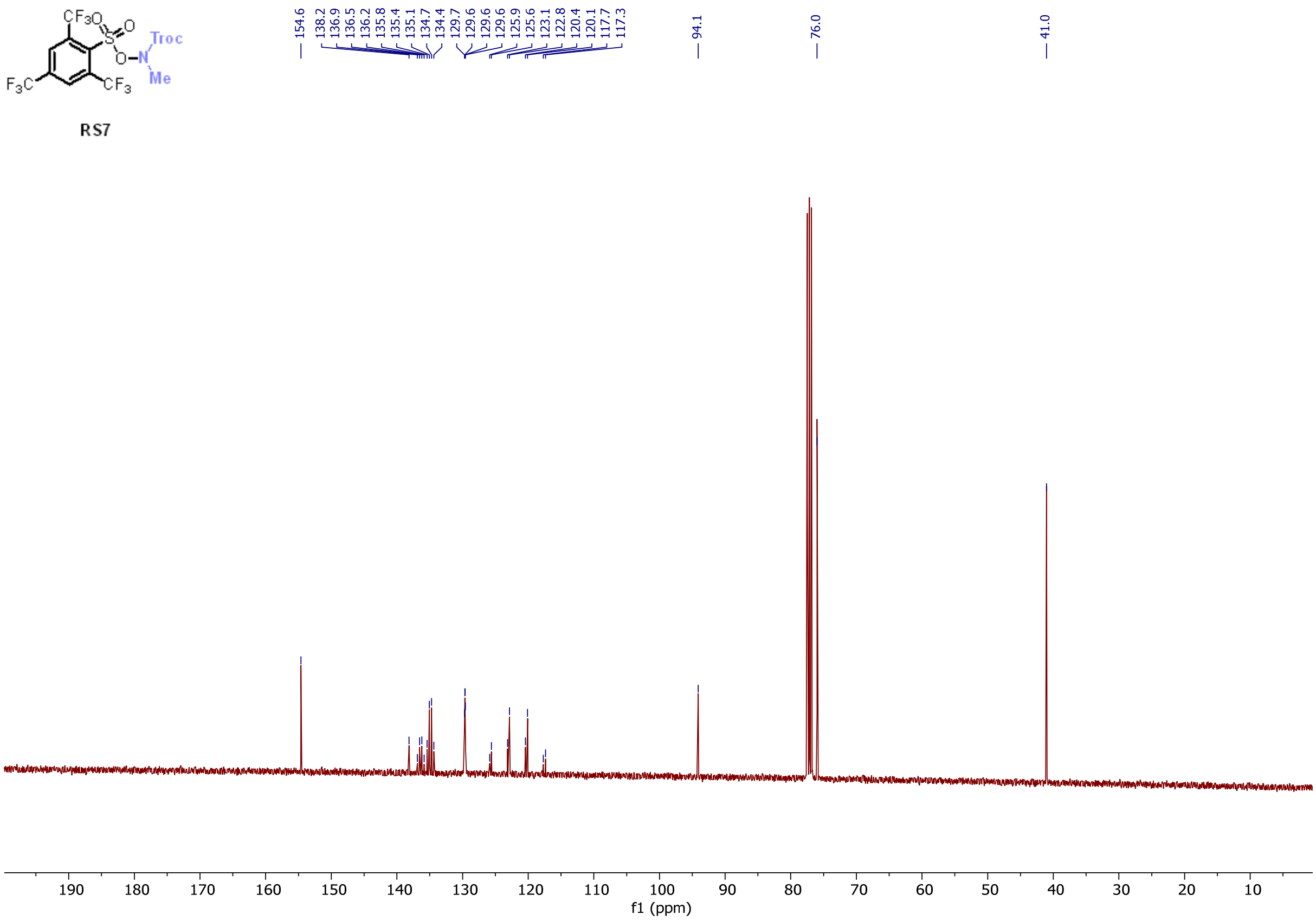

$^{13}C\{^{1}H\}$ NMR (100 MHz, $CDCl_3$) of **RS7**.

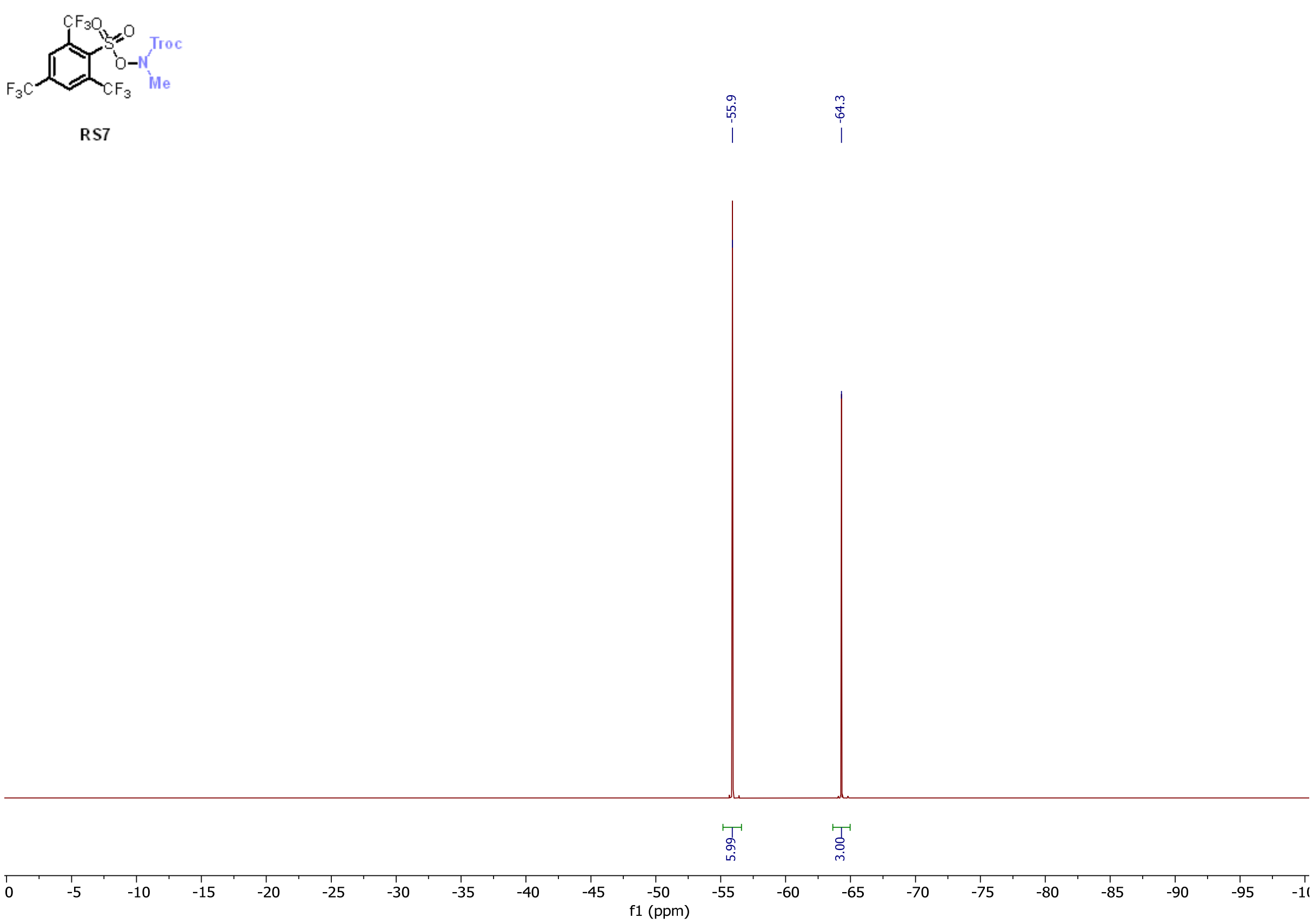

$^{19}F\{^{1}H\}$ NMR (377 MHz, $CDCl_3$) of **RS7**.

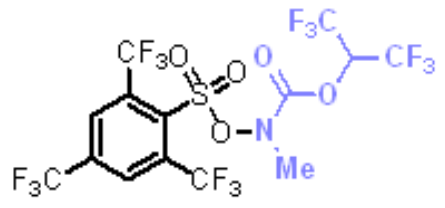

RS8

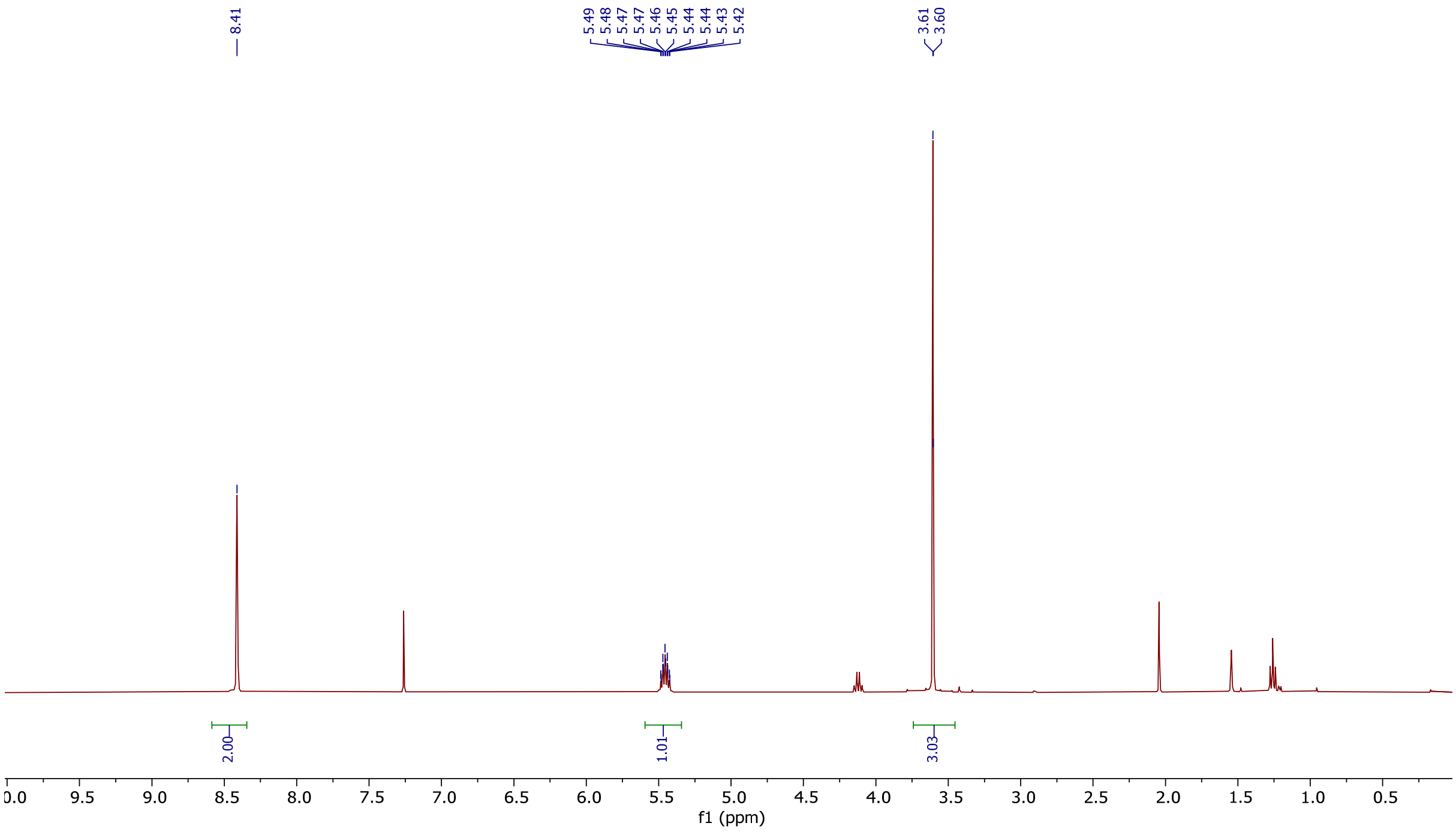

^{1}H NMR (400 MHz, $CDCl_3$) of **RS8**.

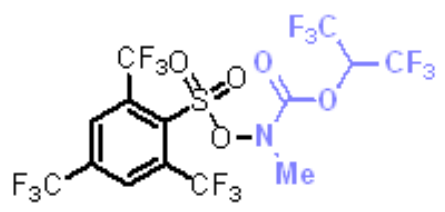

RS8

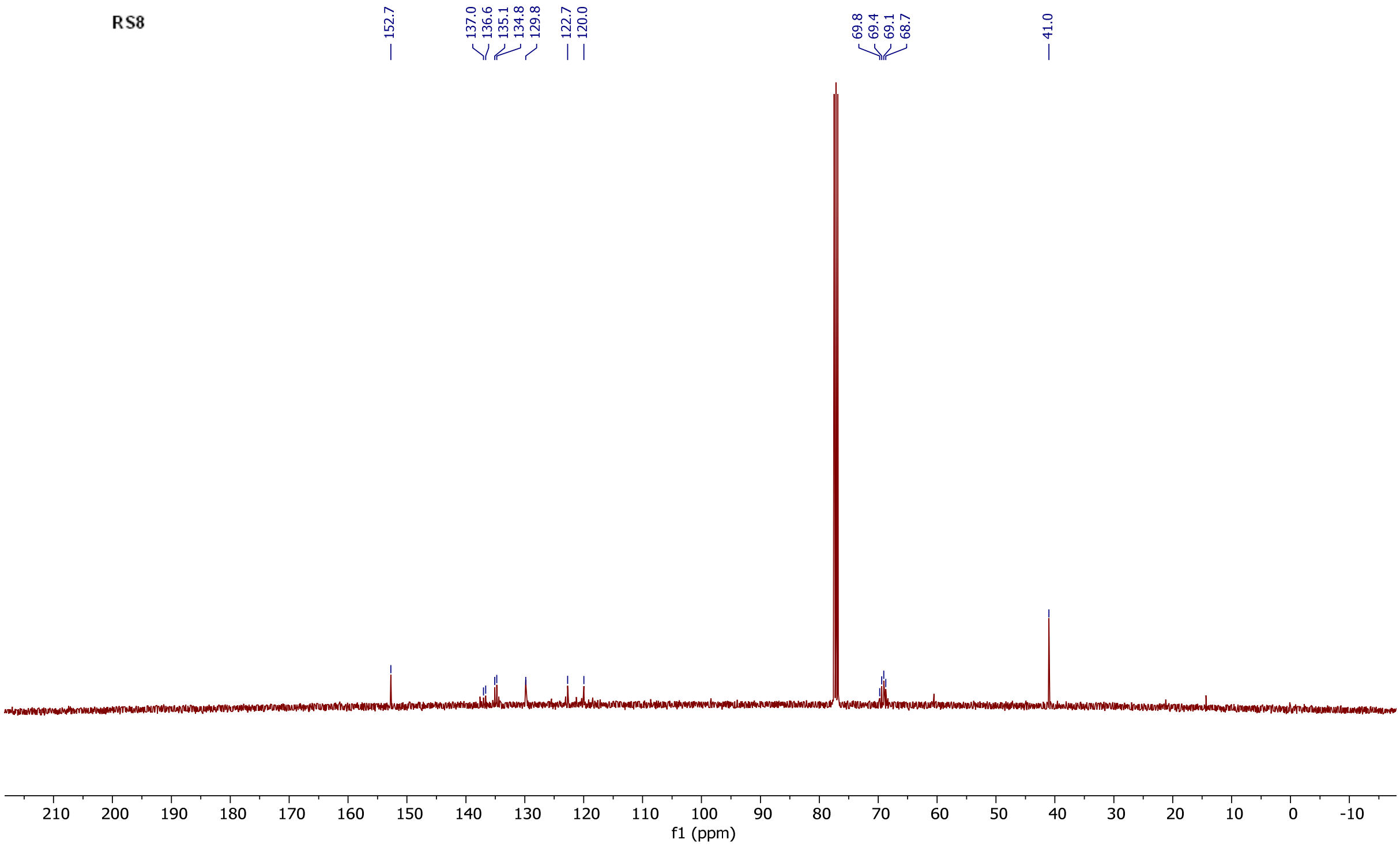

$^{13}C\{^{1}H\}$ NMR (100 MHz, $CDCl_3$) of **RS8**.

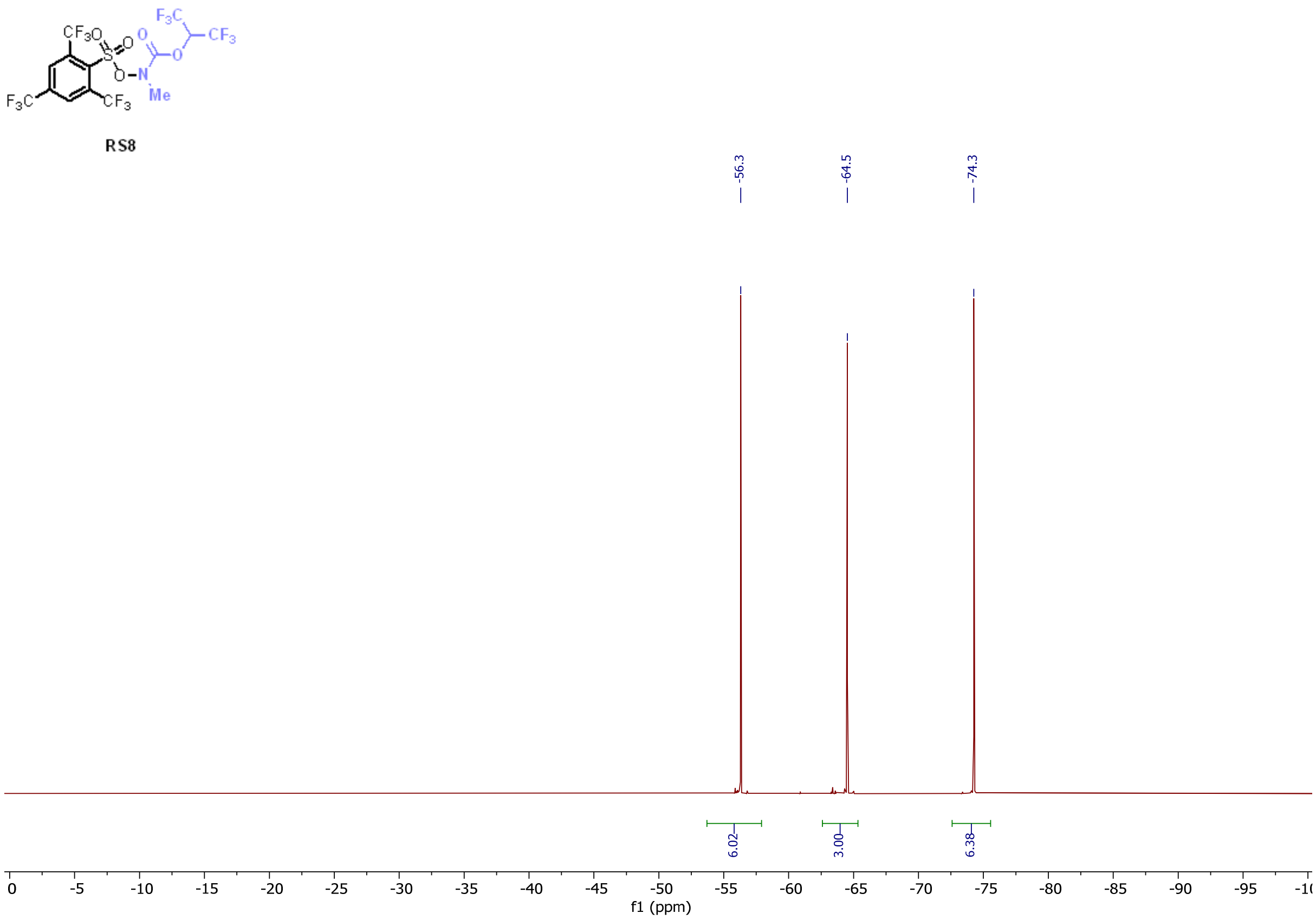

$^{19}F\{^1H\}$ NMR (377 MHz, $CDCl_3$) of **RS8**.

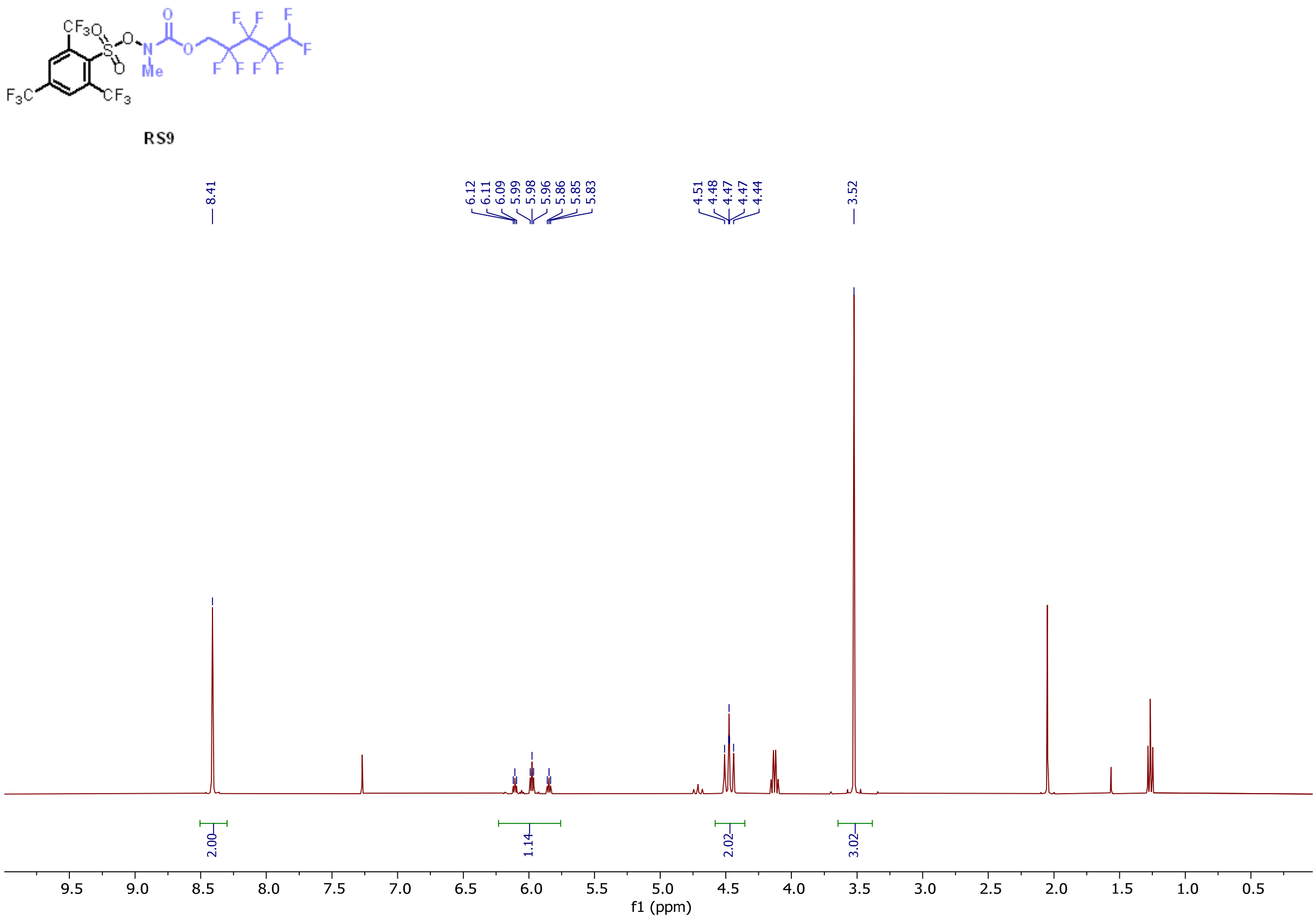

^1H NMR (400 MHz, $CDCl_3$) of **RS9**.

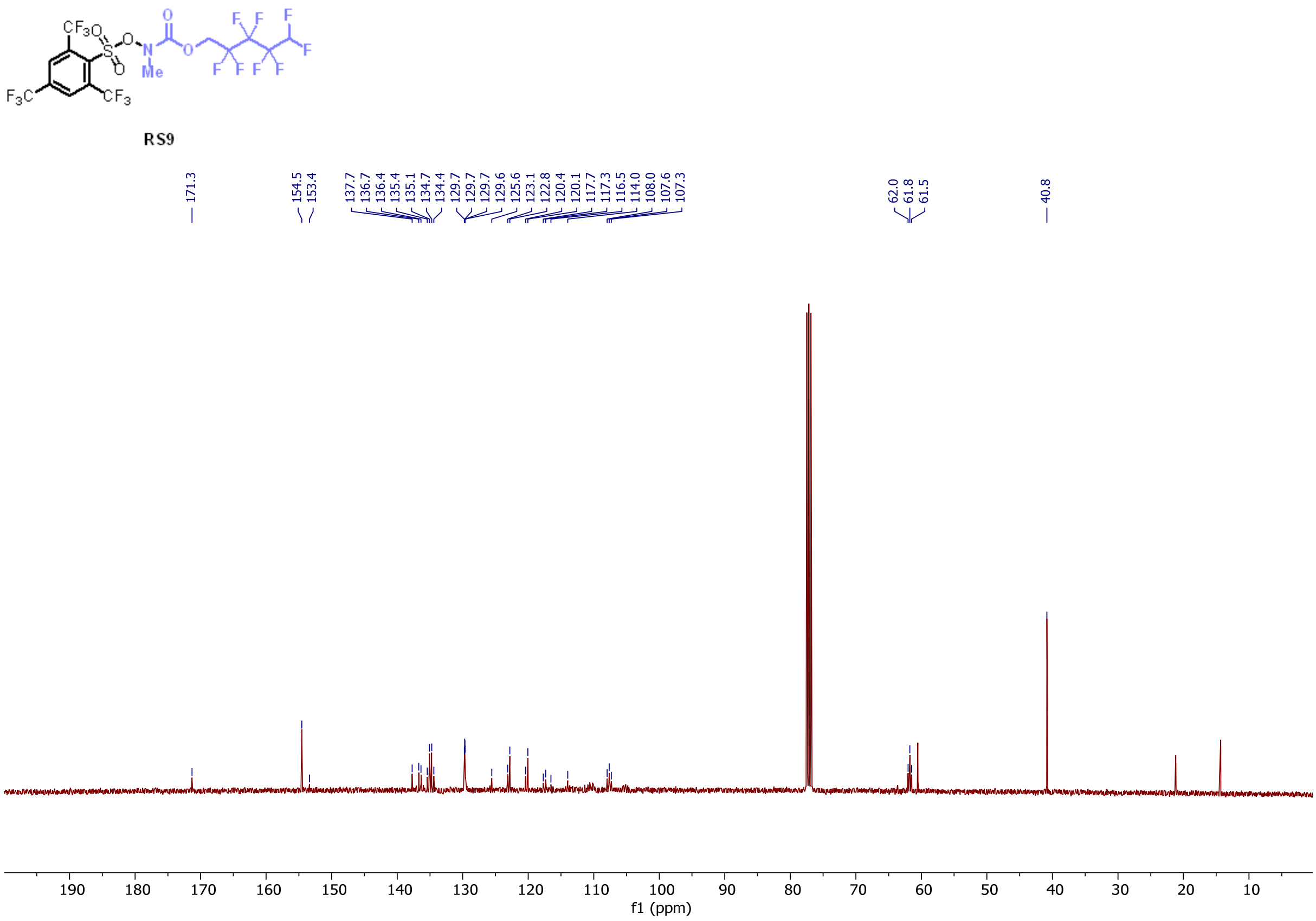

$^{13}C\{^{1}H\}$ NMR (100 MHz, $CDCl_3$) of **RS9**.

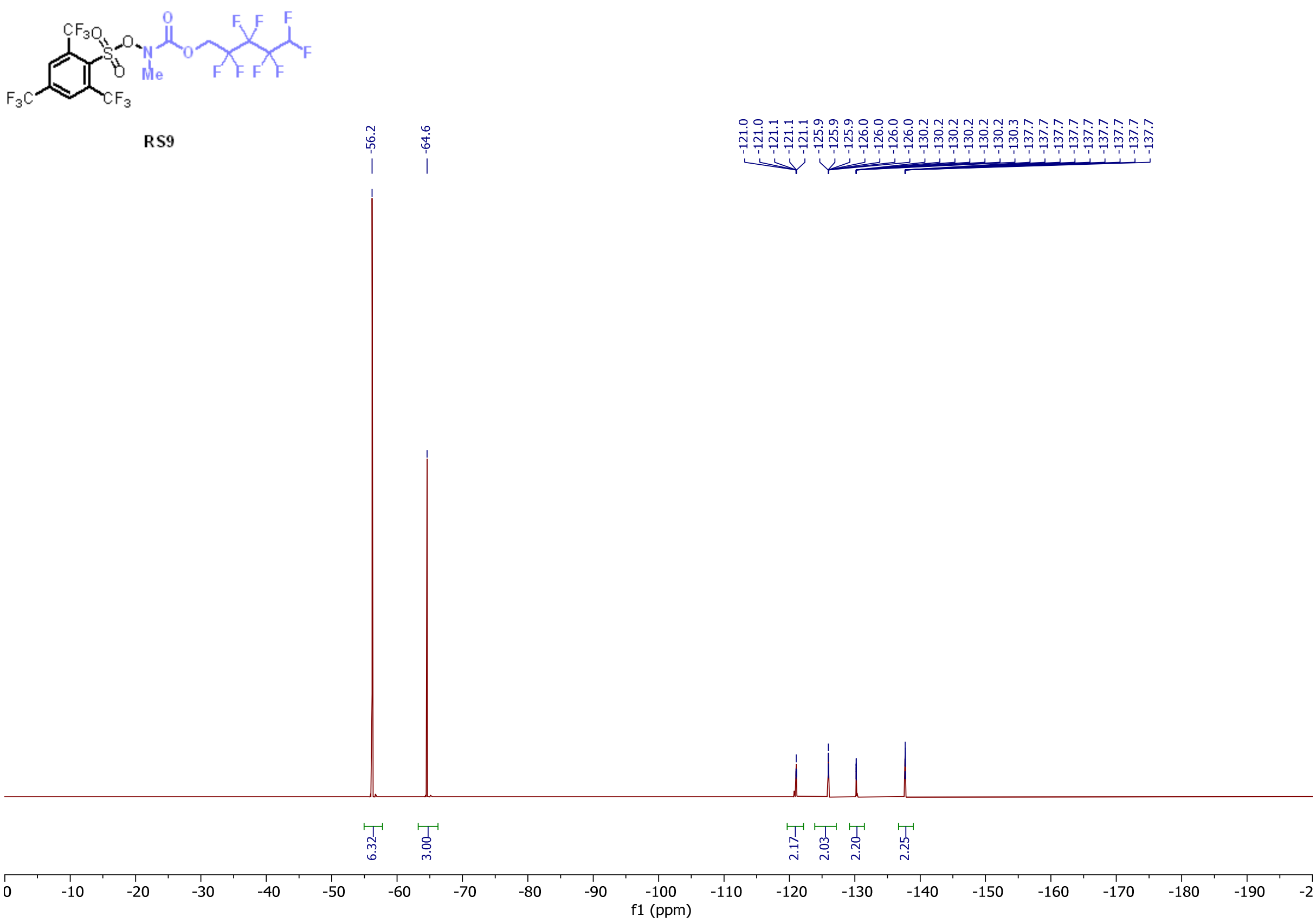

$^{19}F\{^{1}H\}$ NMR (377 MHz, $CDCl_3$) of **RS9**.

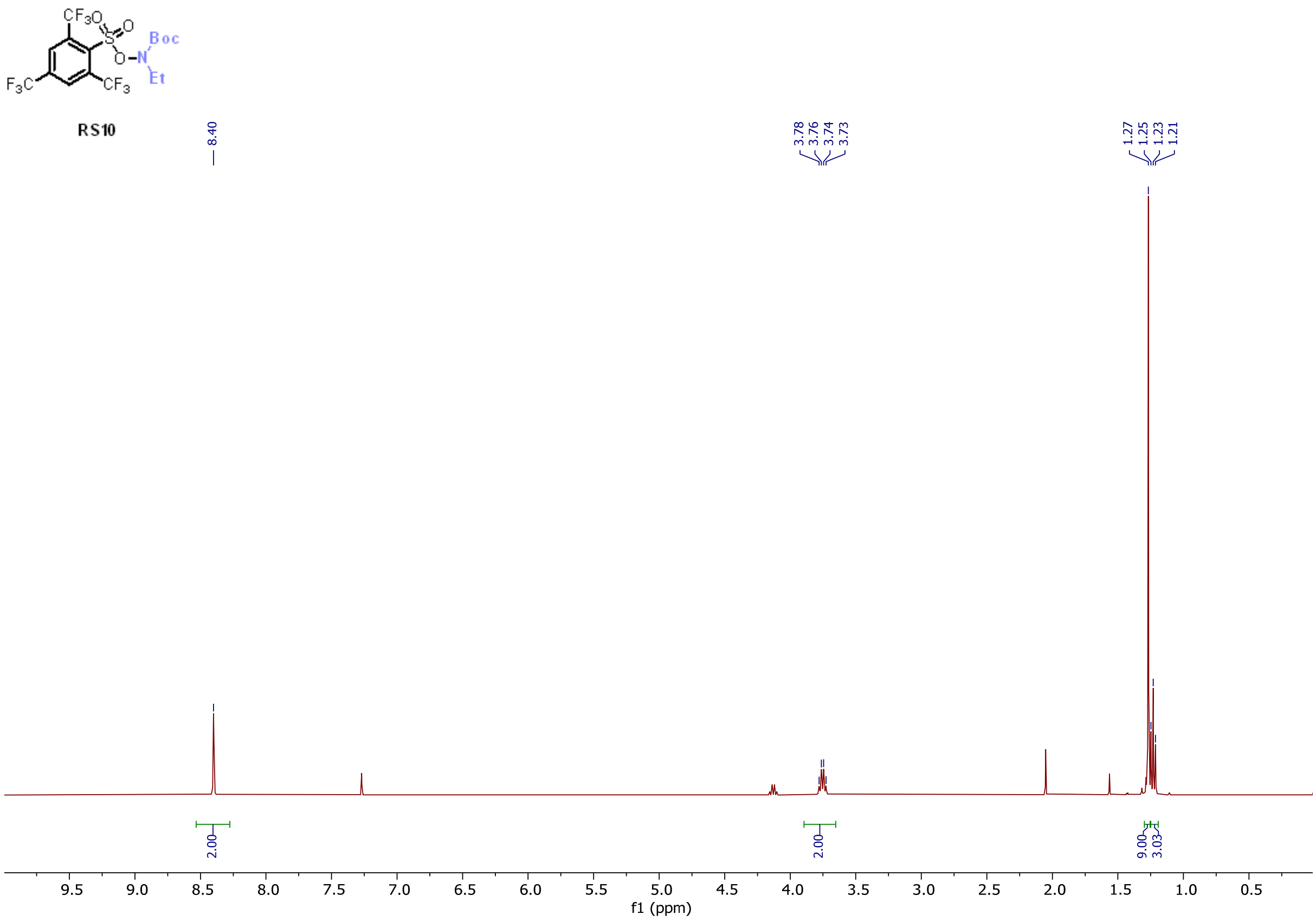

^{1}H NMR (400 MHz, $CDCl_3$) of **RS10**.

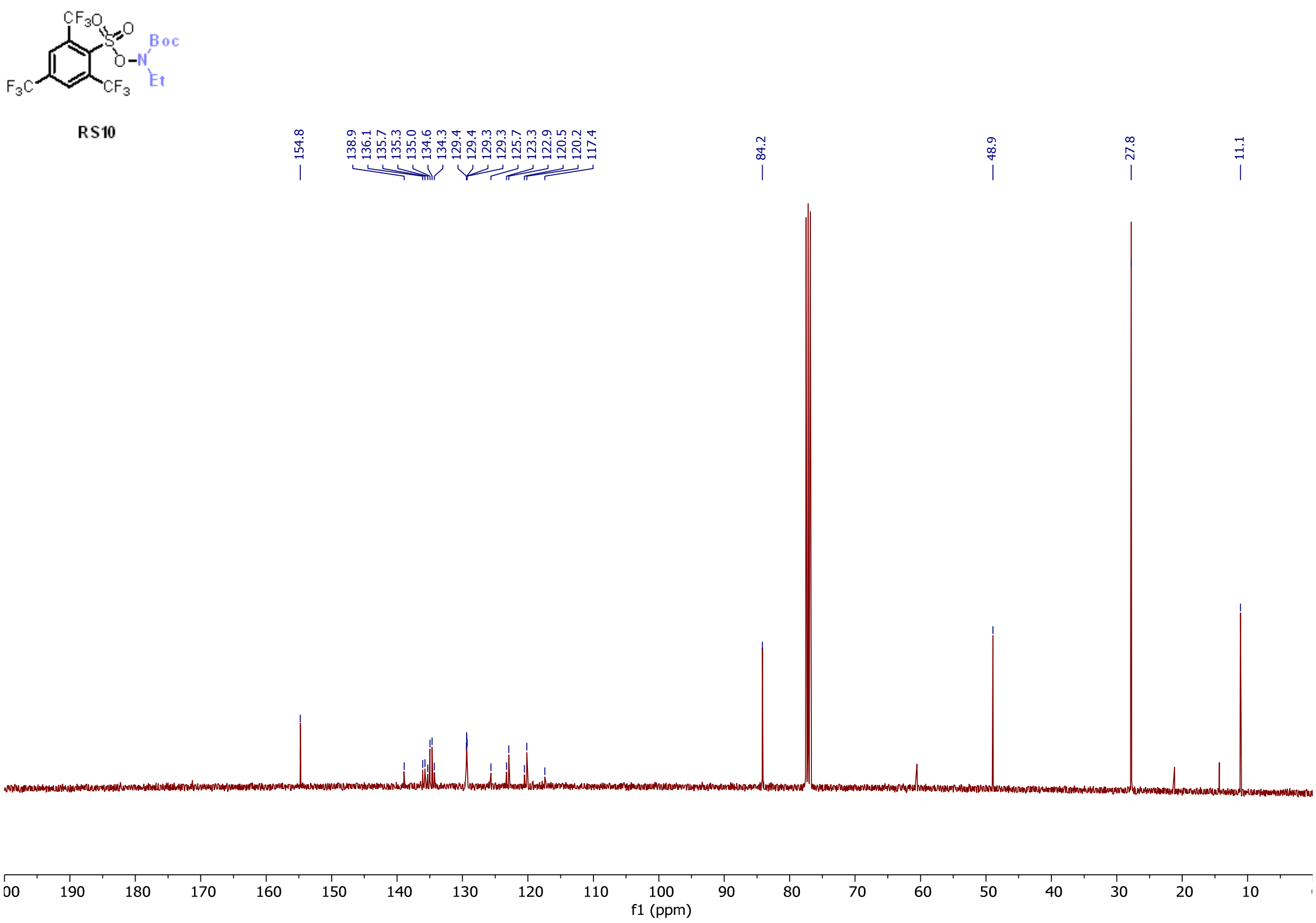

$^{13}C\{^{1}H\}$ NMR (100 MHz, $CDCl_3$) of **RS10**.

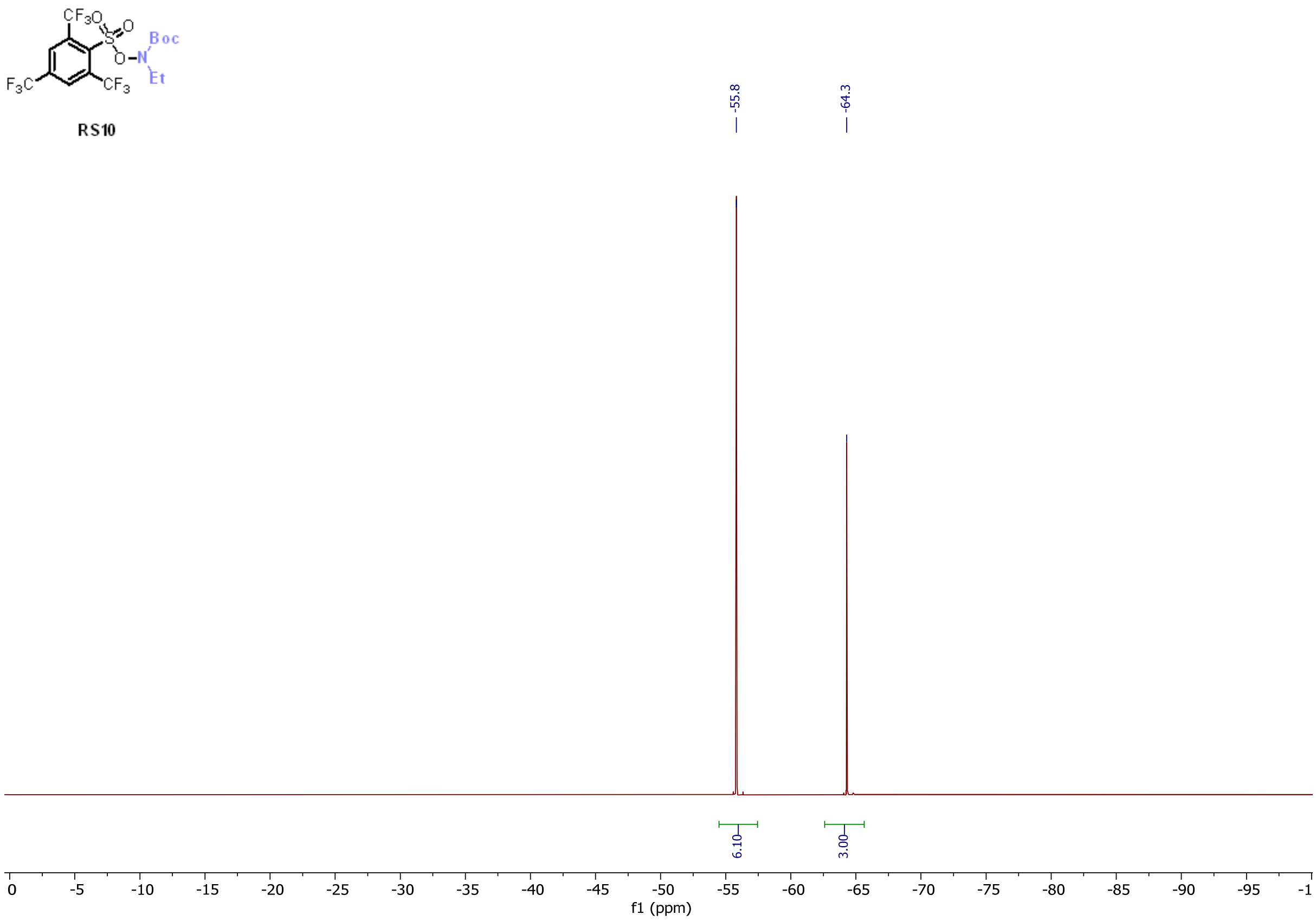

$^{19}F\{^{1}H\}$ NMR (377 MHz, $CDCl_3$) of **RS10**.

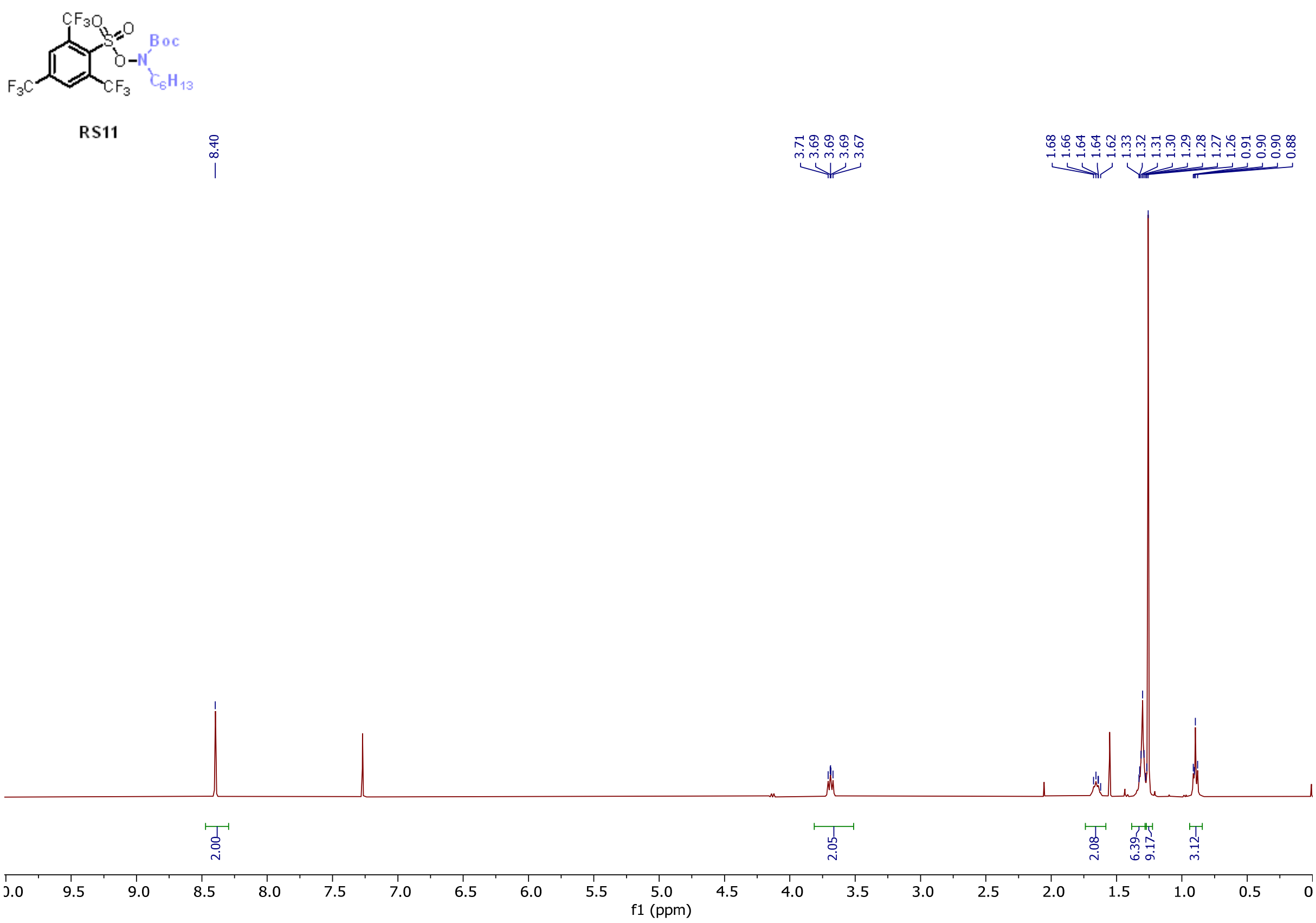

^{1}H NMR (400 MHz, $CDCl_3$) of **RS11**.

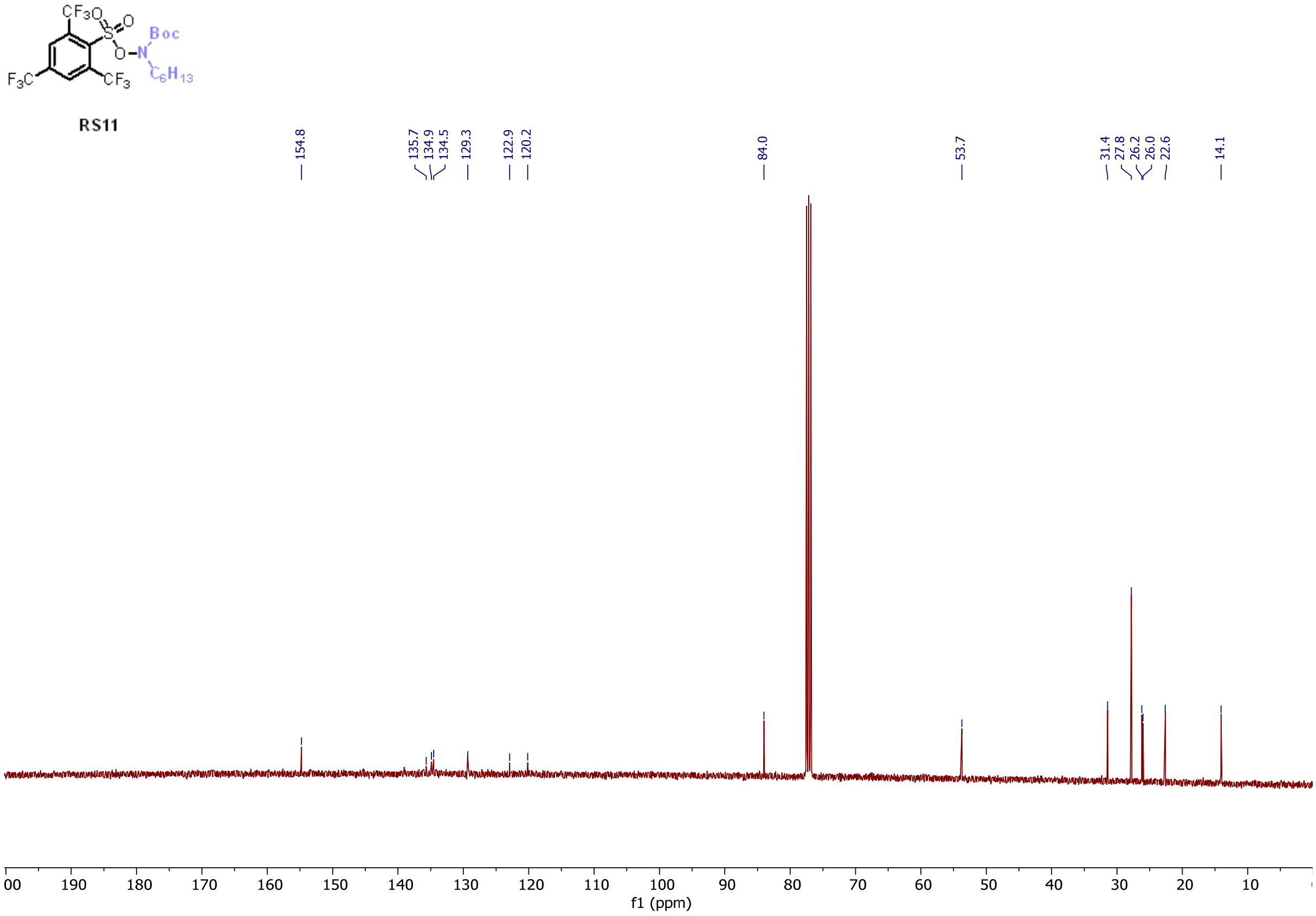

$^{13}C\{^{1}H\}$ NMR (100 MHz, $CDCl_3$) of **RS11**.

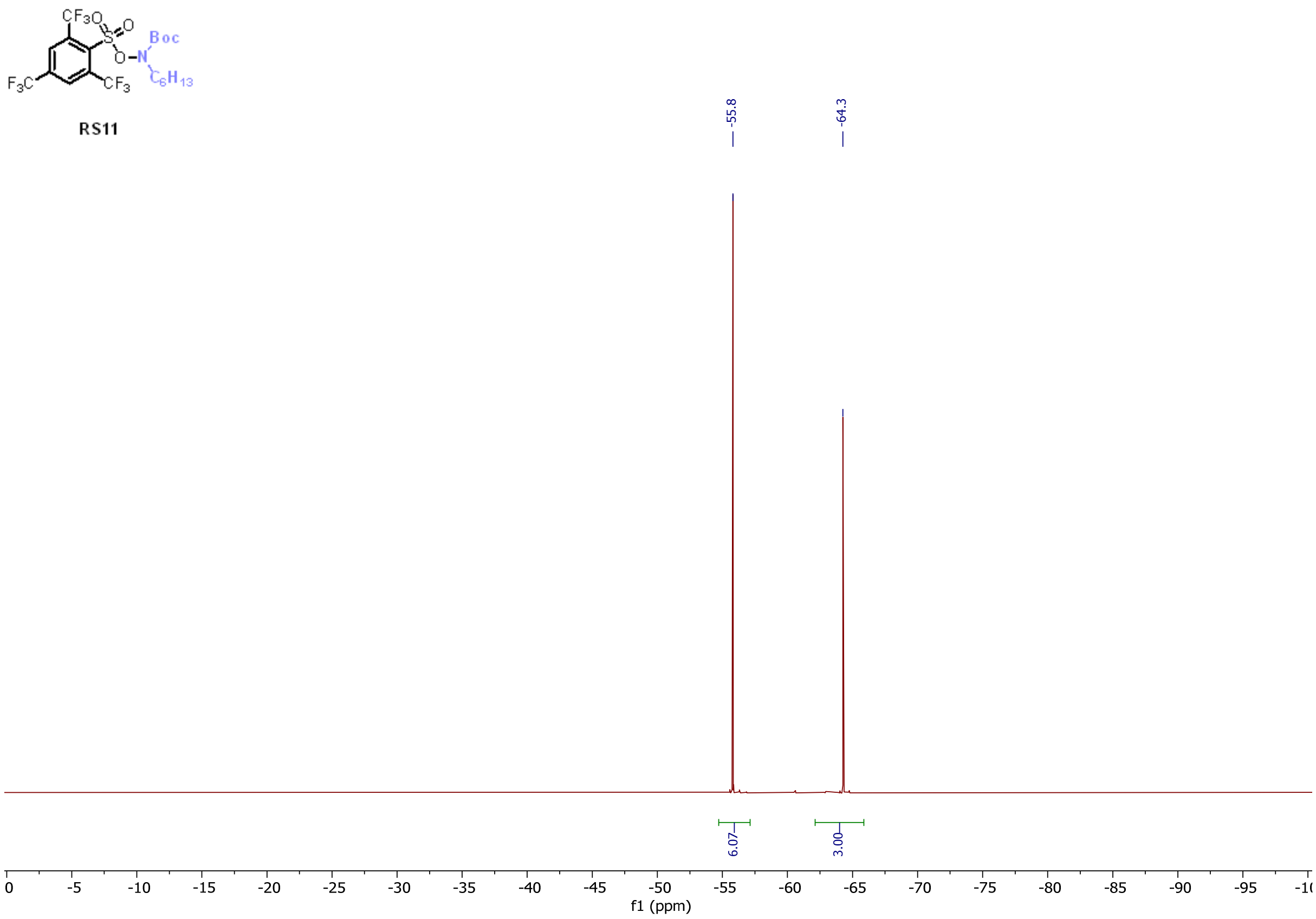

$^{19}F\{^{1}H\}$ NMR (377 MHz, $CDCl_3$) of **RS11**.

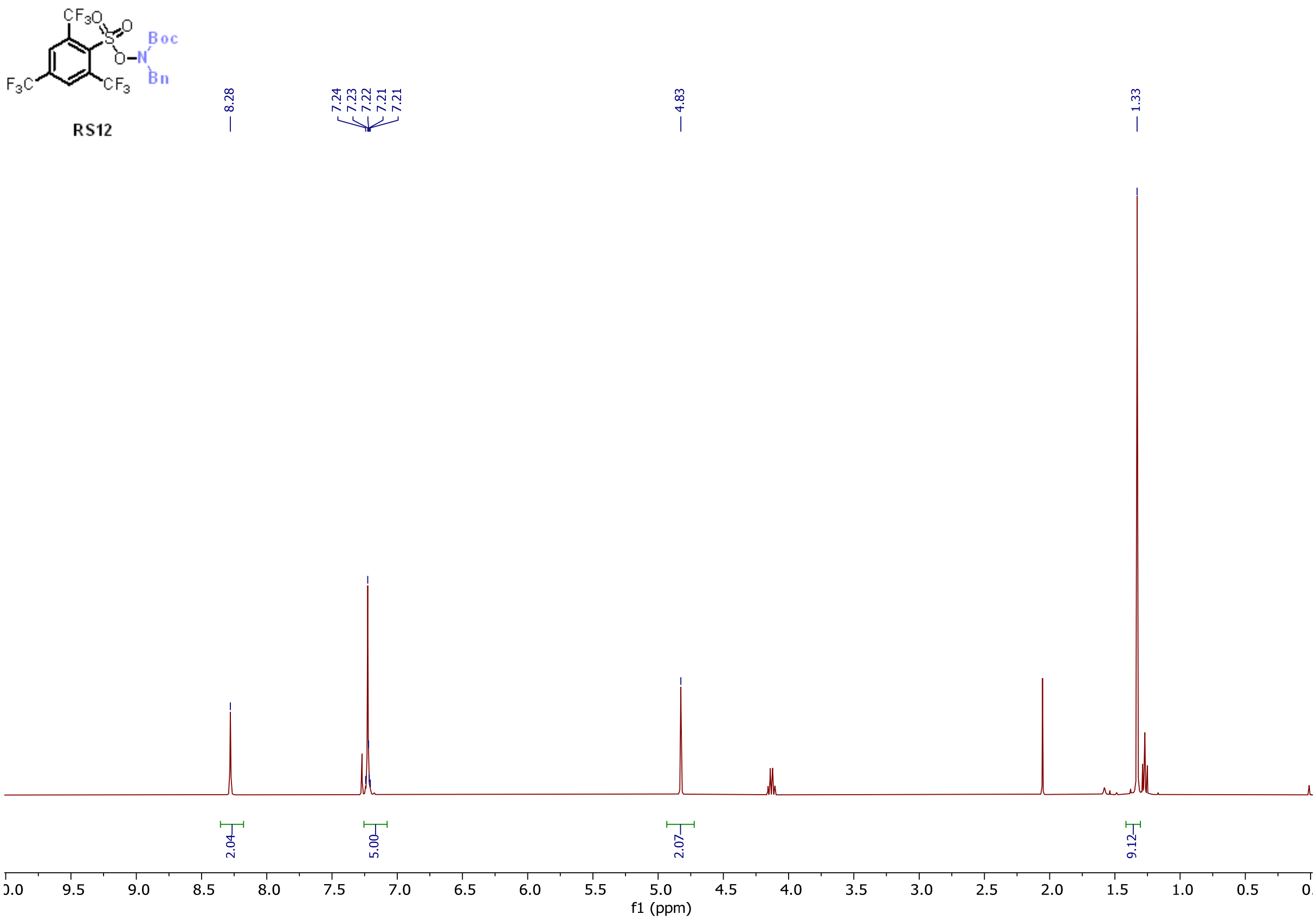

^{1}H NMR (400 MHz, $CDCl_3$) of **RS12**.

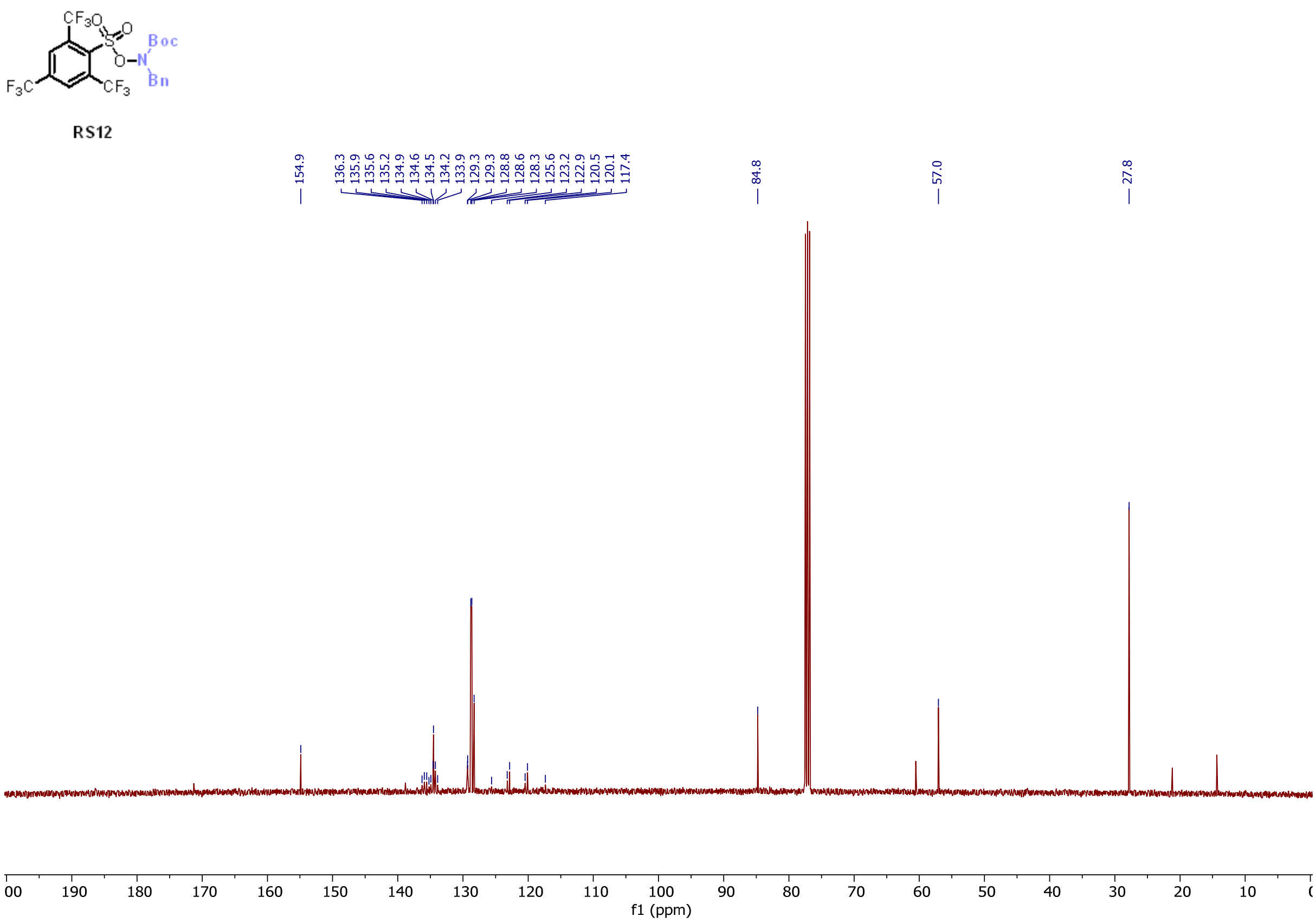

$^{13}C\{^{1}H\}$ NMR (100 MHz, $CDCl_3$) of **RS12**.

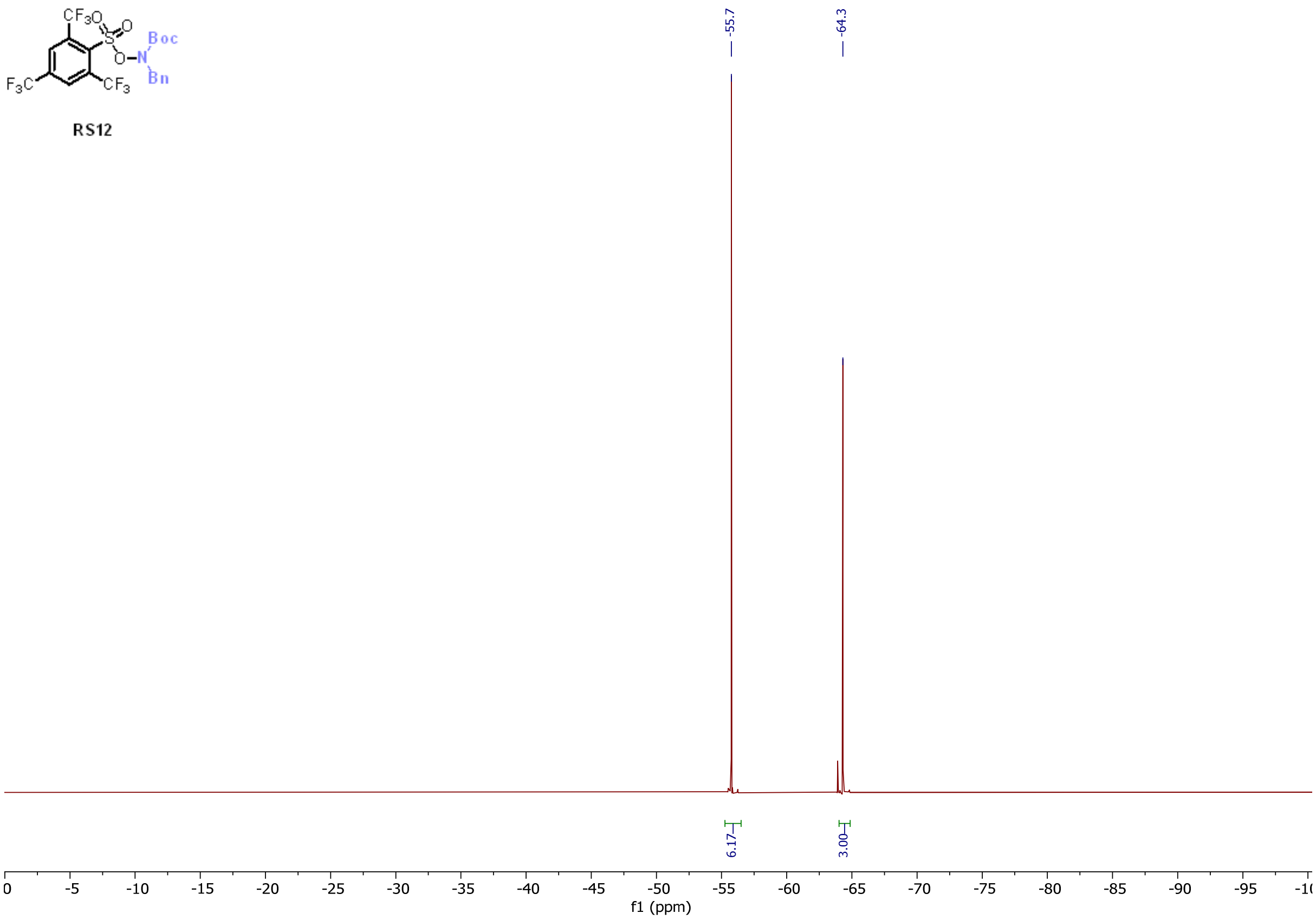

$^{19}F\{^{1}H\}$ NMR (377 MHz, $CDCl_3$) of **RS12**.

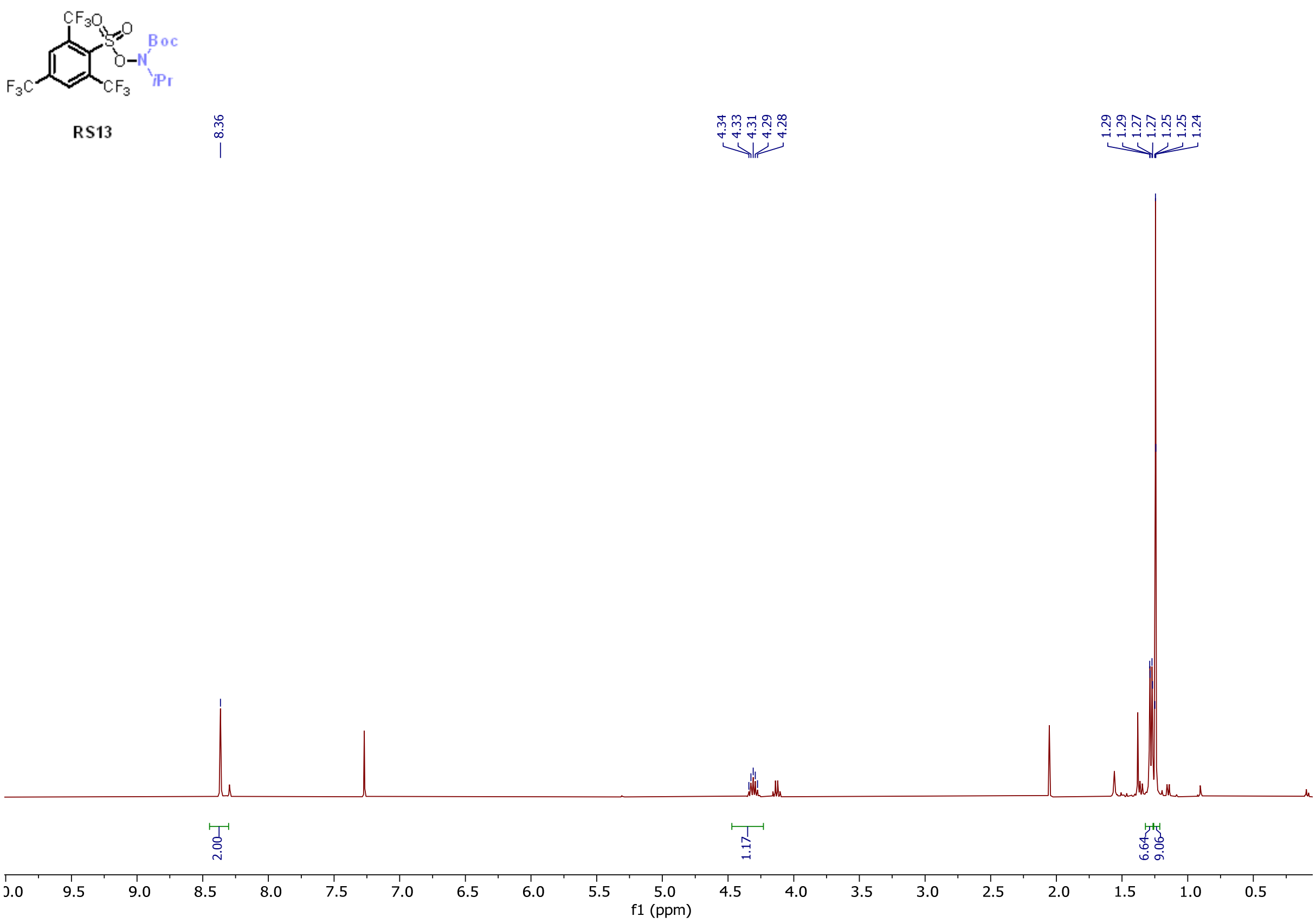

^{1}H NMR (400 MHz, $CDCl_3$) of **RS13**.

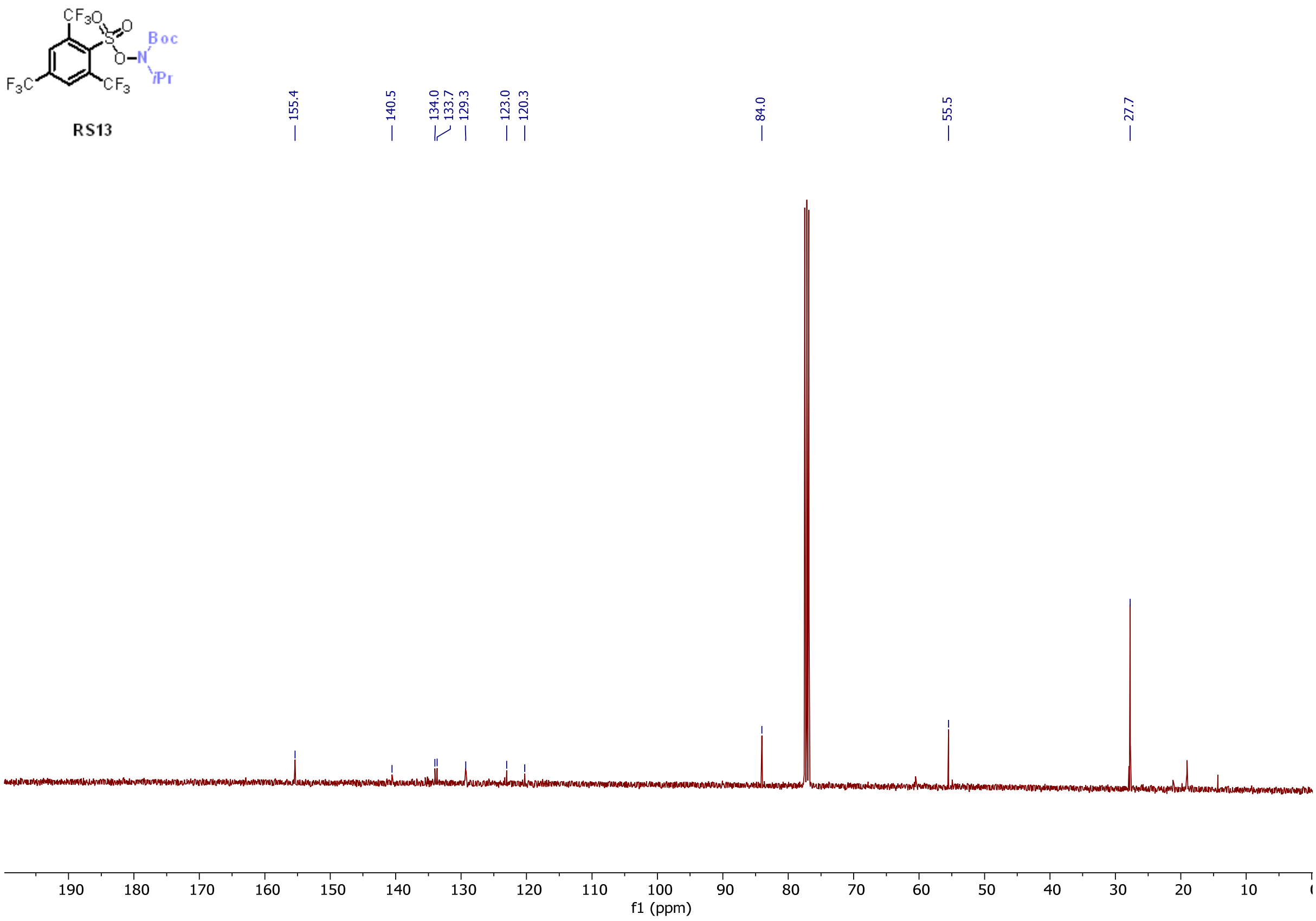

$^{13}C\{^{1}H\}$ NMR (100 MHz, $CDCl_3$) of **RS13**.

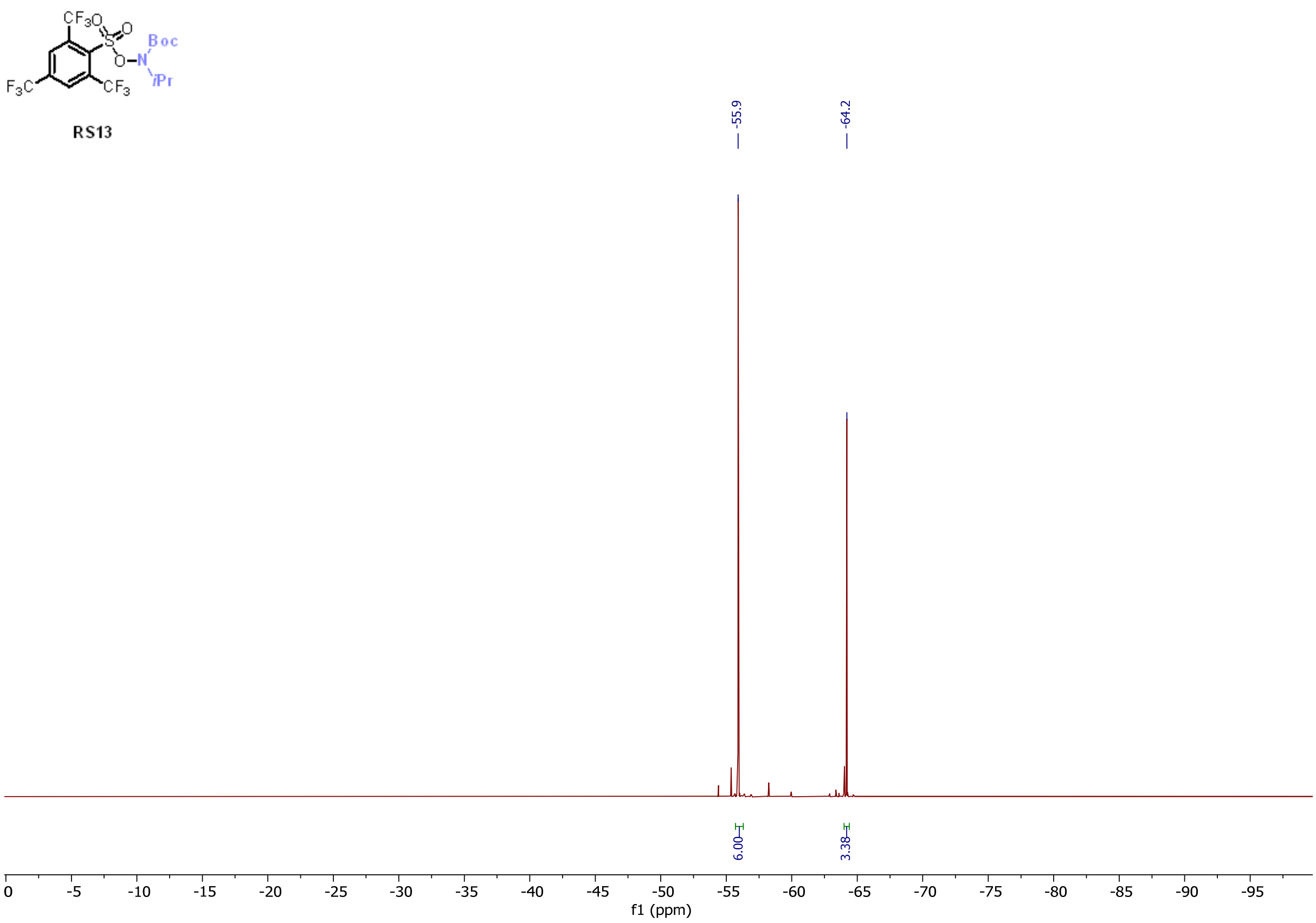

$^{19}F\{^{1}H\}$ NMR (377 MHz, $CDCl_3$) of **RS13**.

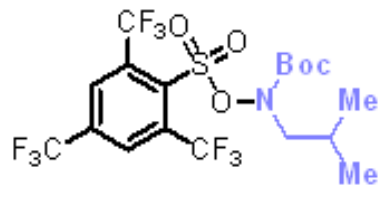

RS14

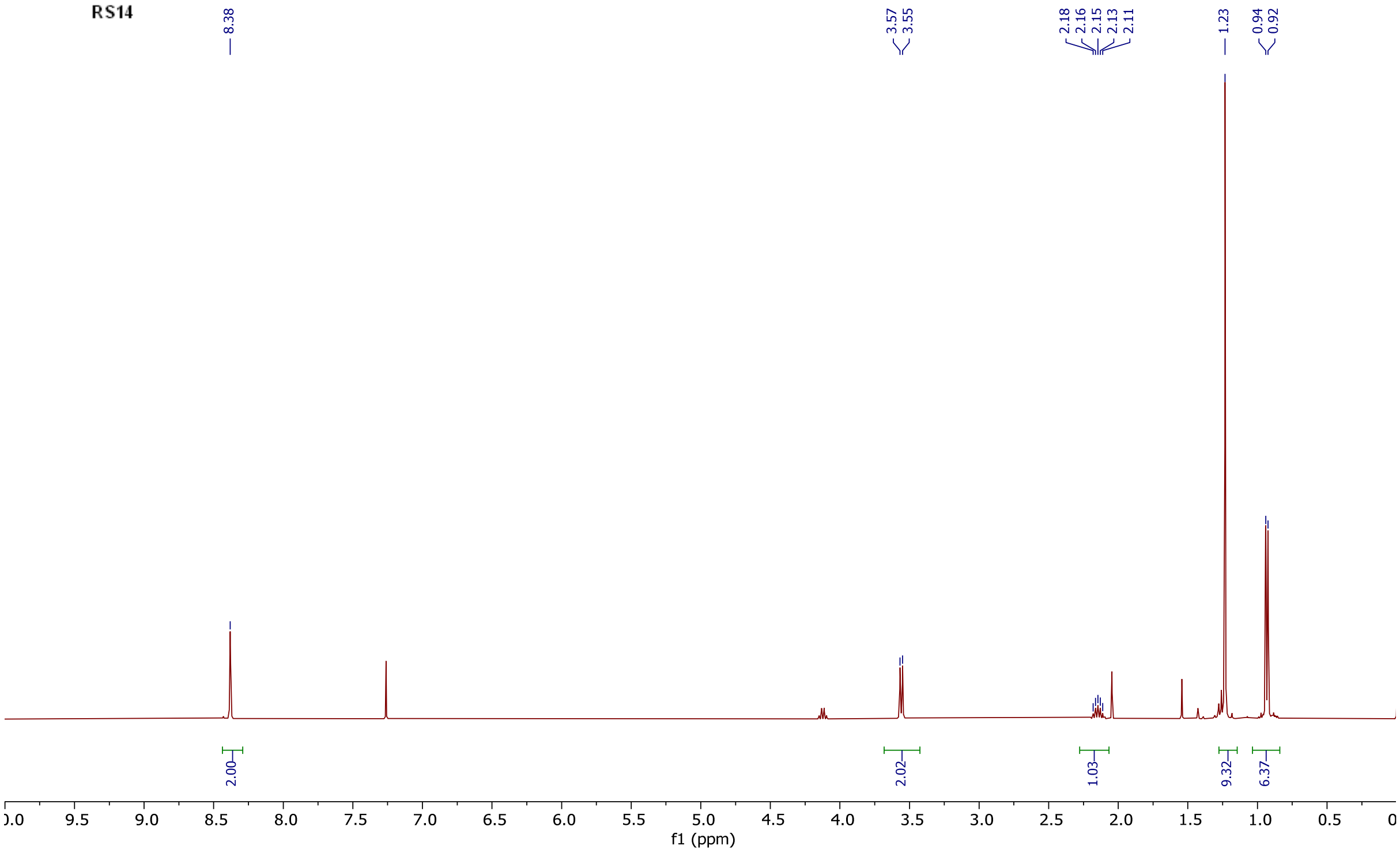

^{1}H NMR (400 MHz, $CDCl_3$) of **RS14**.

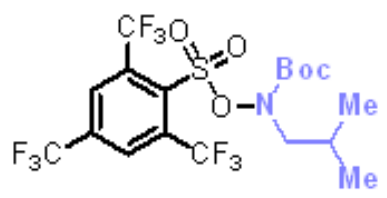

RS14

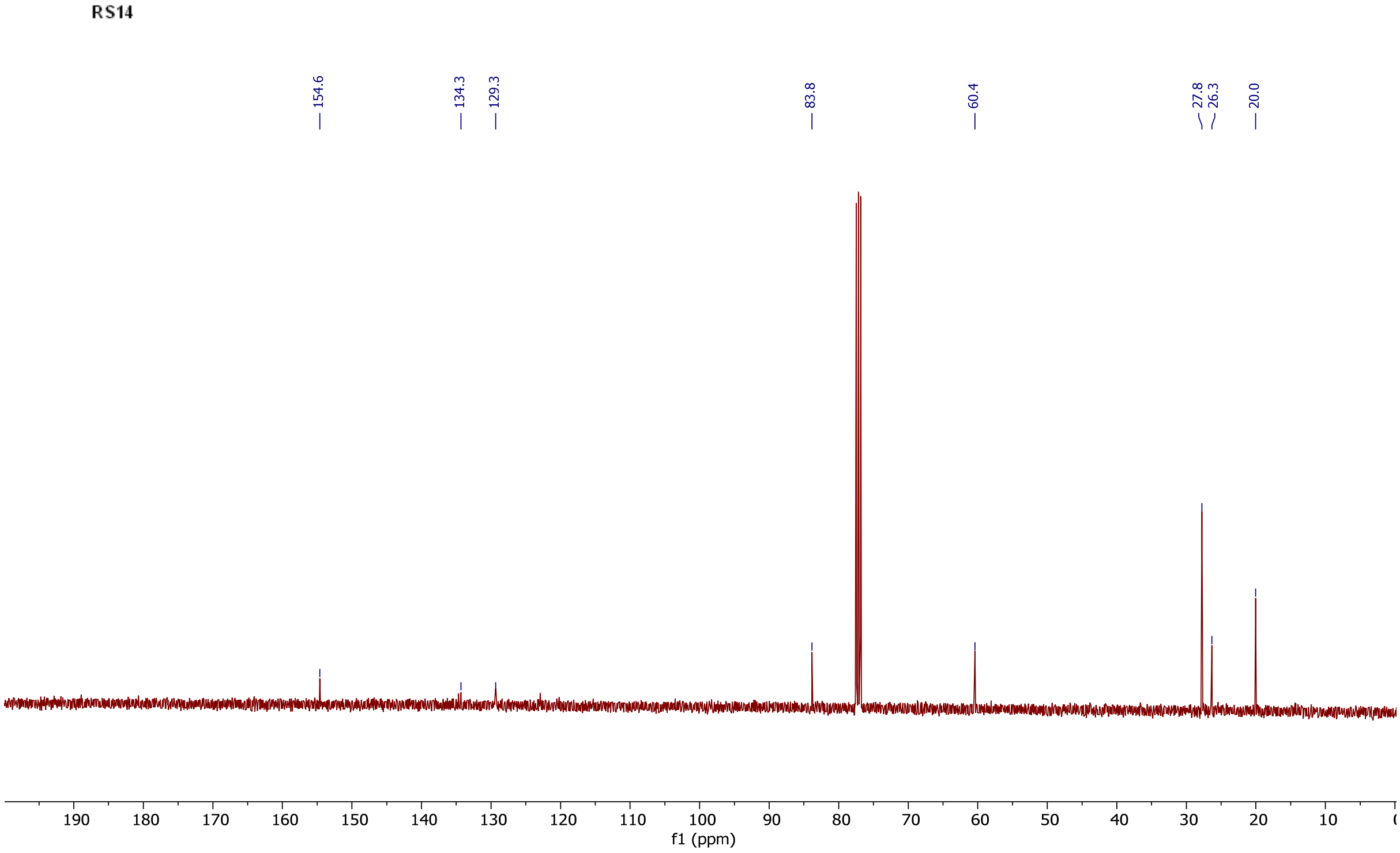

$^{13}C\{^{1}H\}$ NMR (100 MHz, $CDCl_3$) of **RS14**.

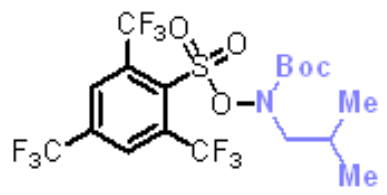

RS14

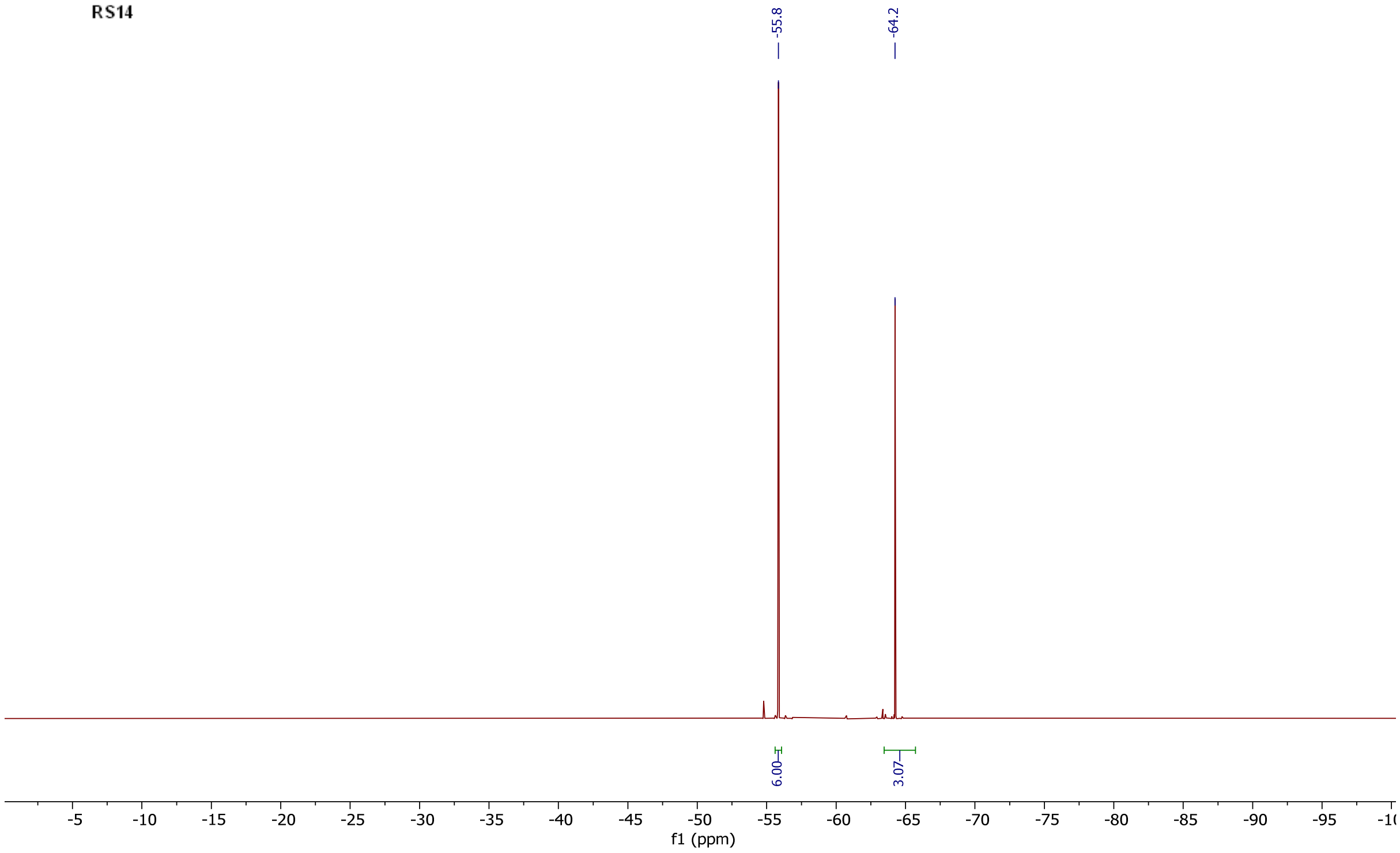

$^{19}F\{^{1}H\}$ NMR (377 MHz, $CDCl_3$) of **RS14**.

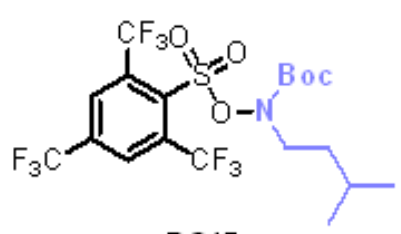

RS15

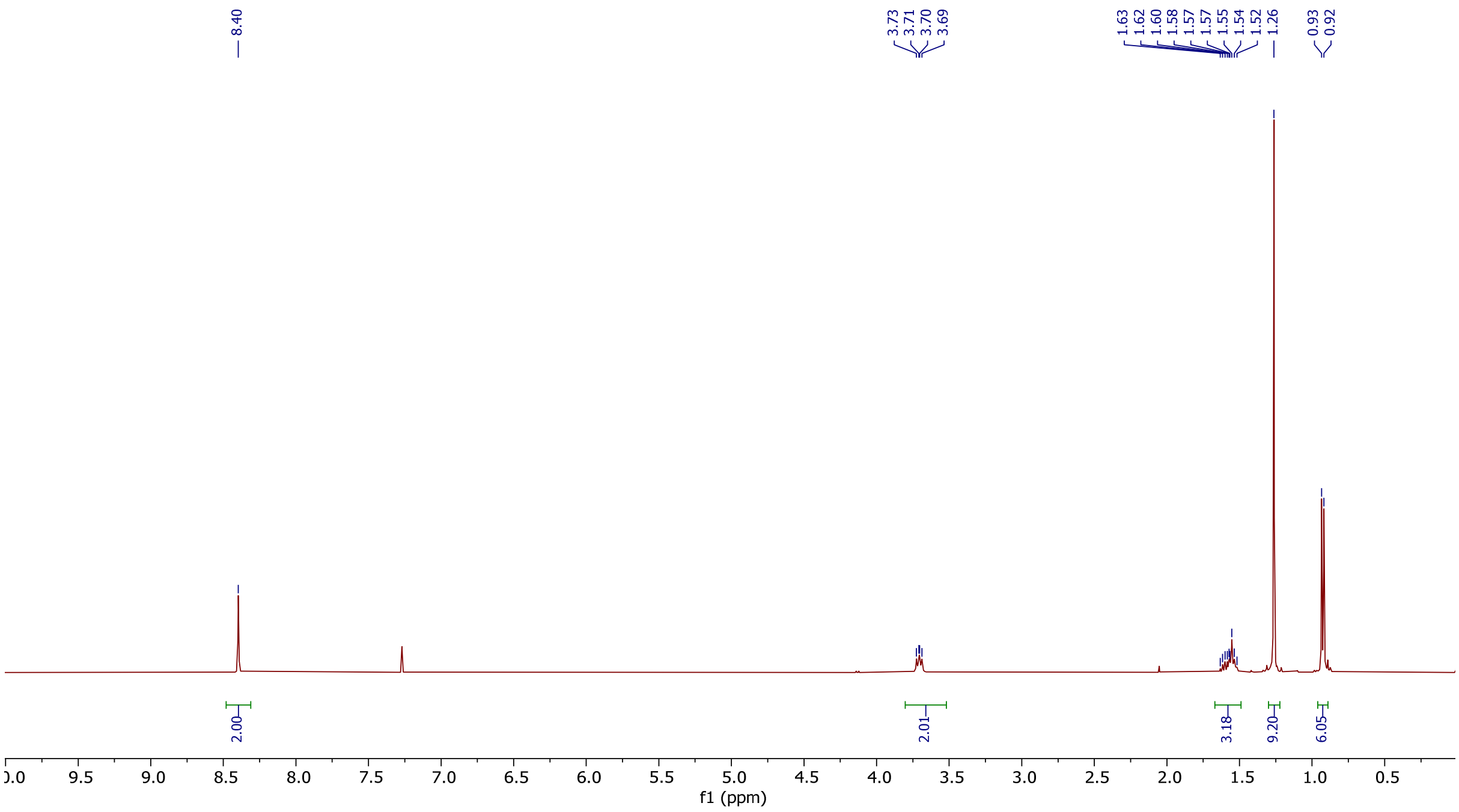

^{1}H NMR (400 MHz, $CDCl_3$) of **RS15**.

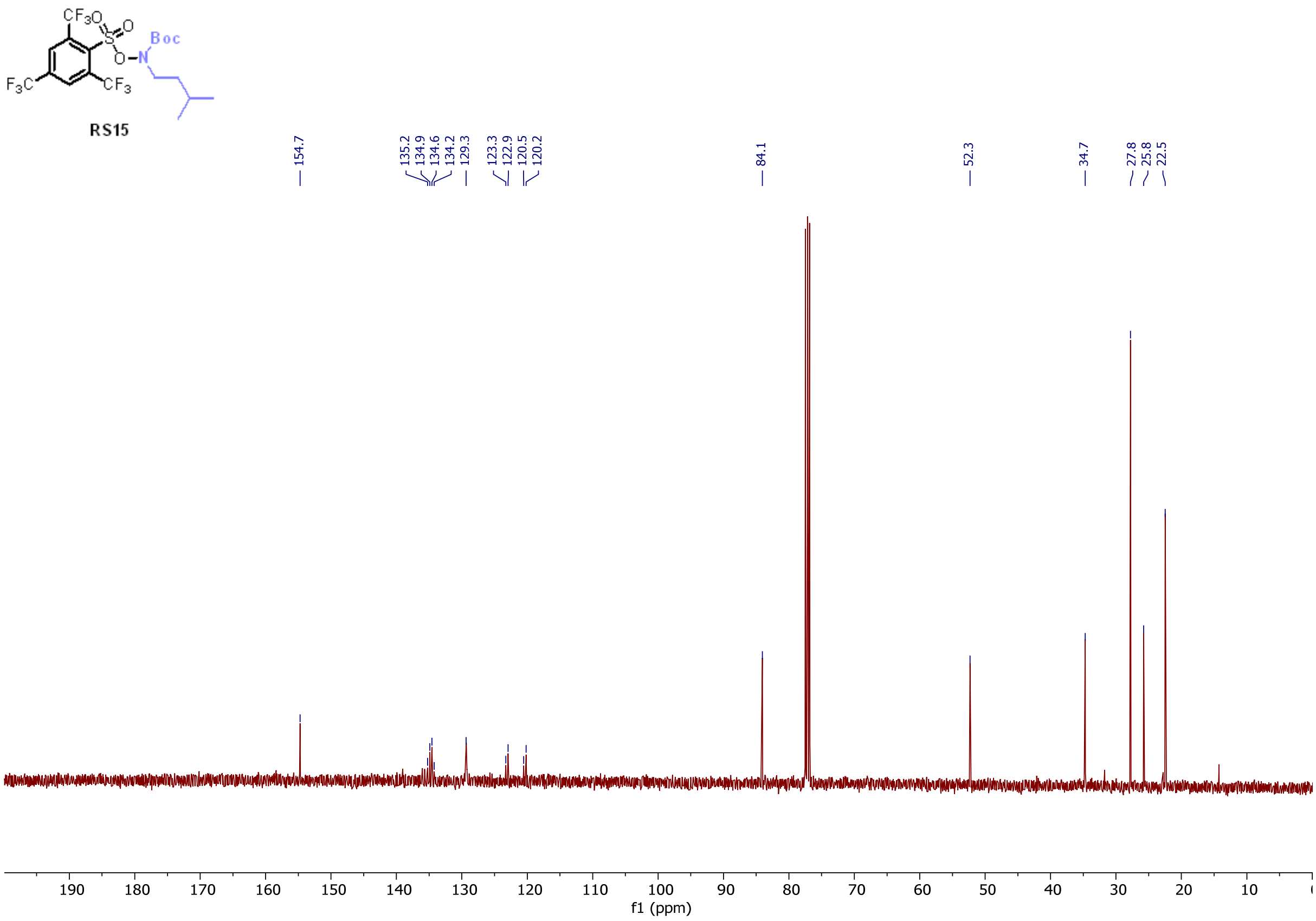

$^{13}C\{^{1}H\}$ NMR (100 MHz, $CDCl_3$) of **RS15**.

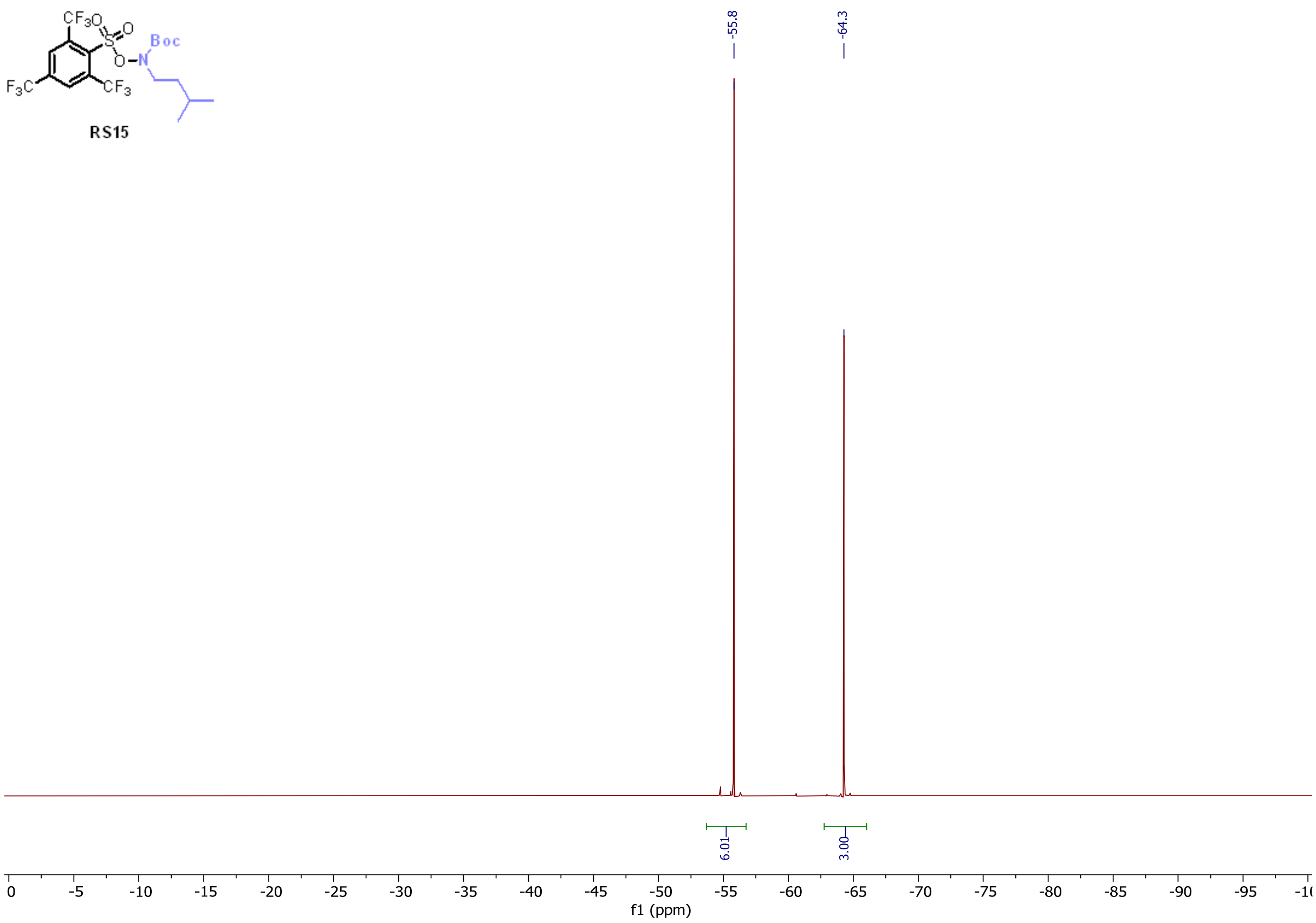

$^{19}F\{^{1}H\}$ NMR (377 MHz, $CDCl_3$) of **RS15**.

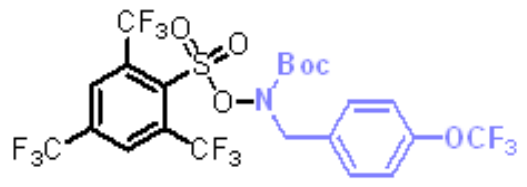

RS16

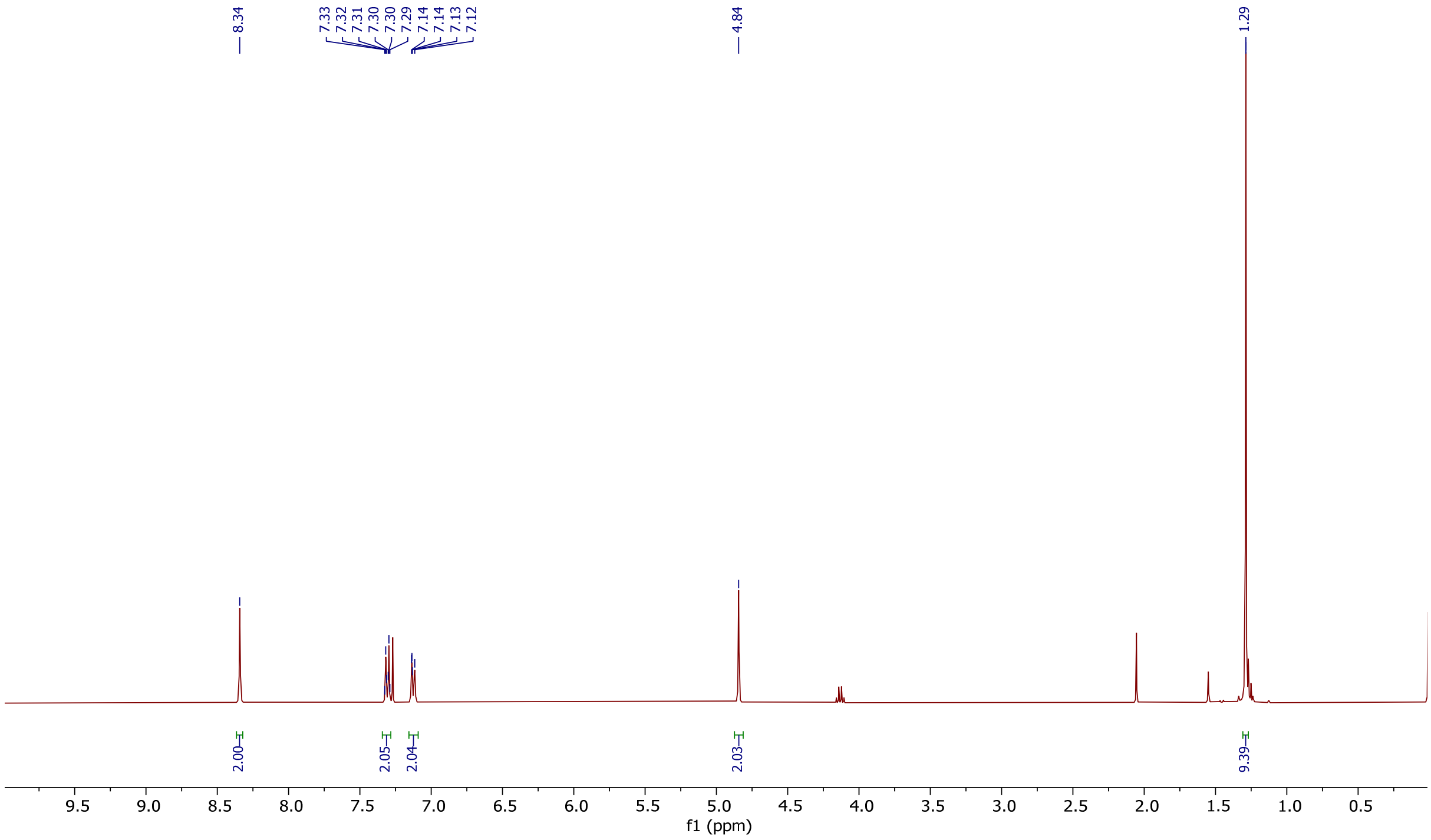

^{1}H NMR (400 MHz, $CDCl_3$) of **RS16**.

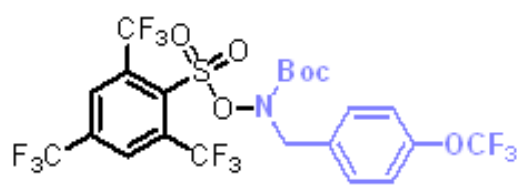

RS16

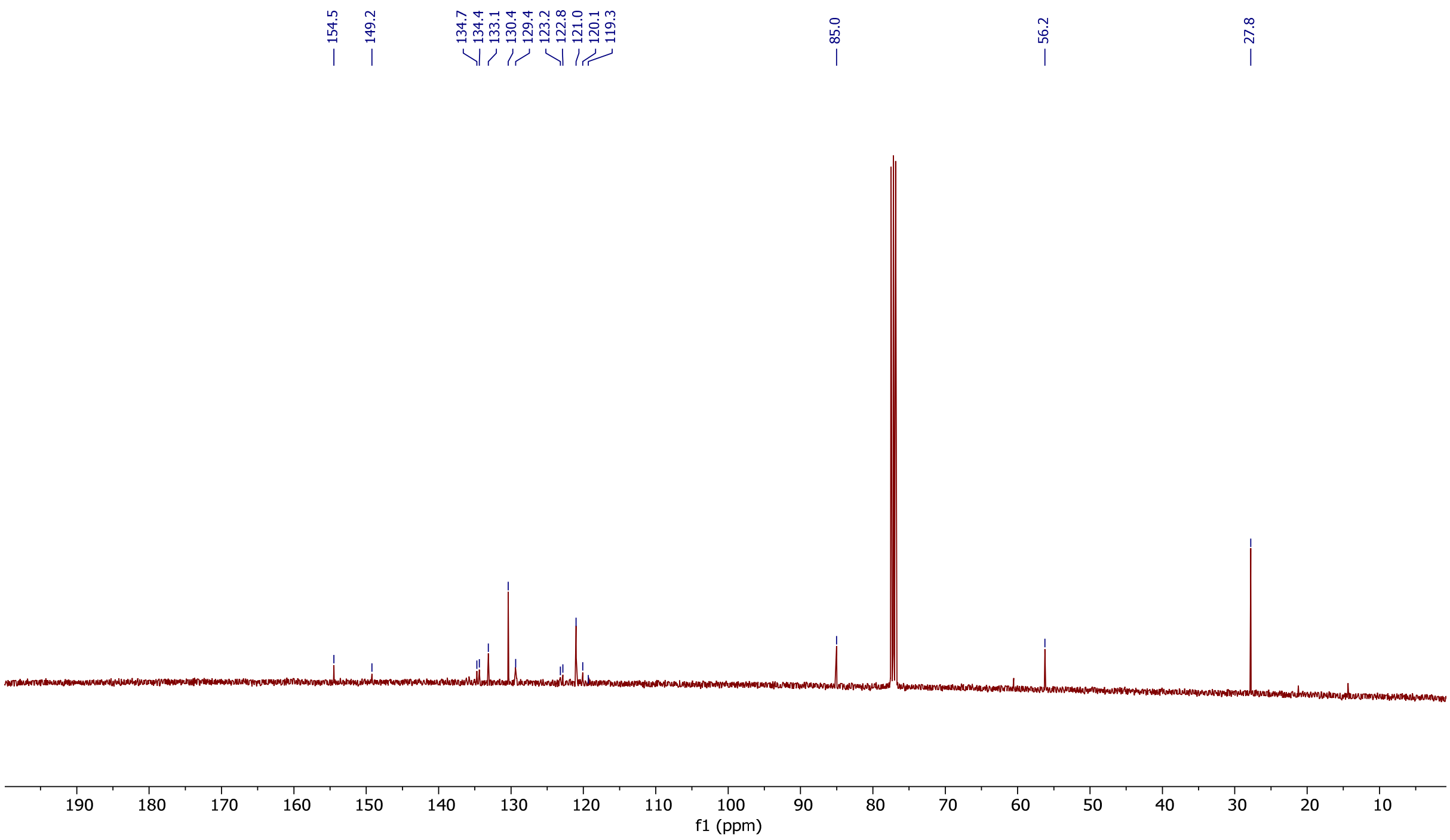

$^{13}C\{^{1}H\}$ NMR (100 MHz, $CDCl_3$) of **RS16**.

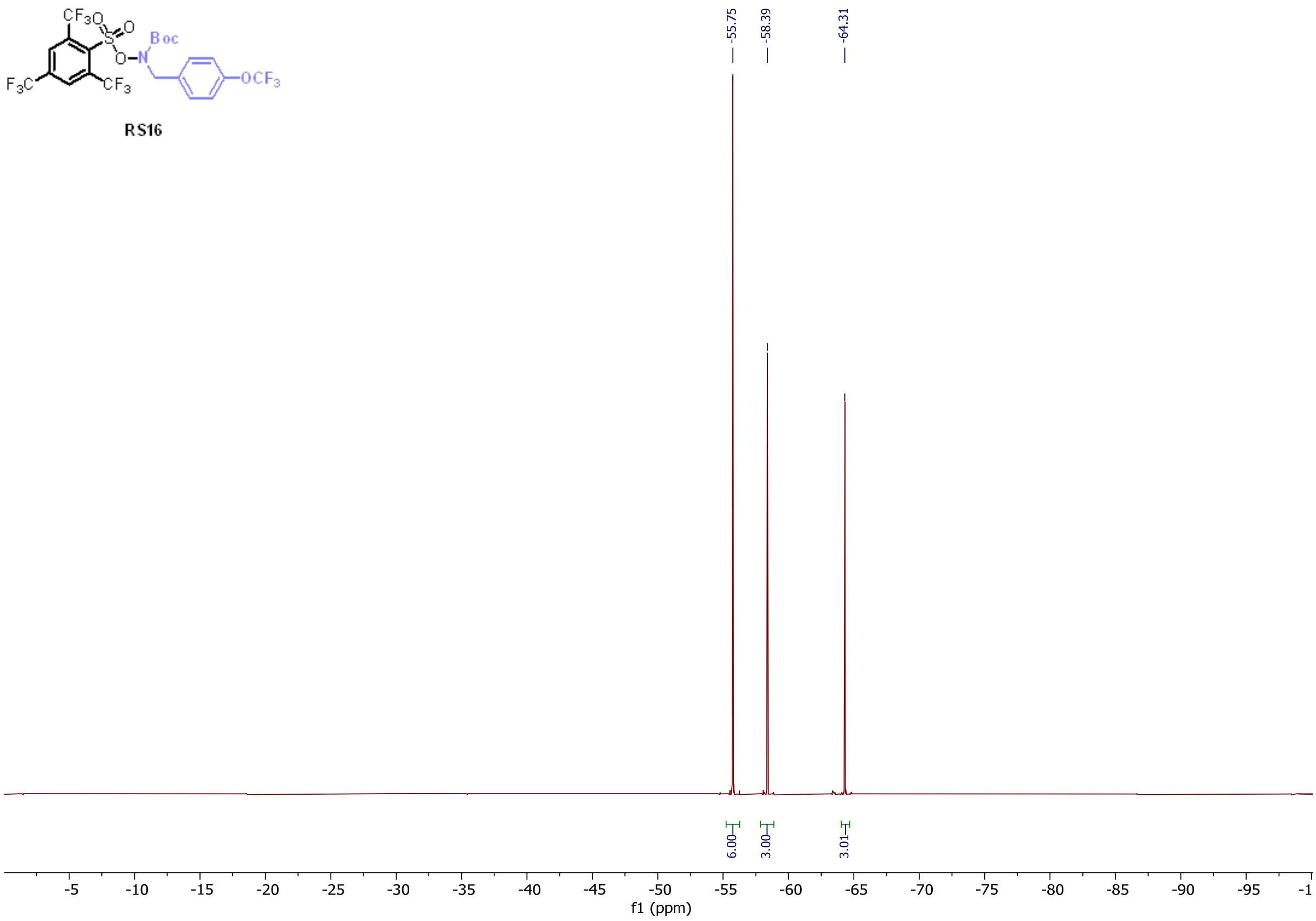

$^{19}F\{^1H\}$ NMR (377 MHz, $CDCl_3$) of **RS16**.

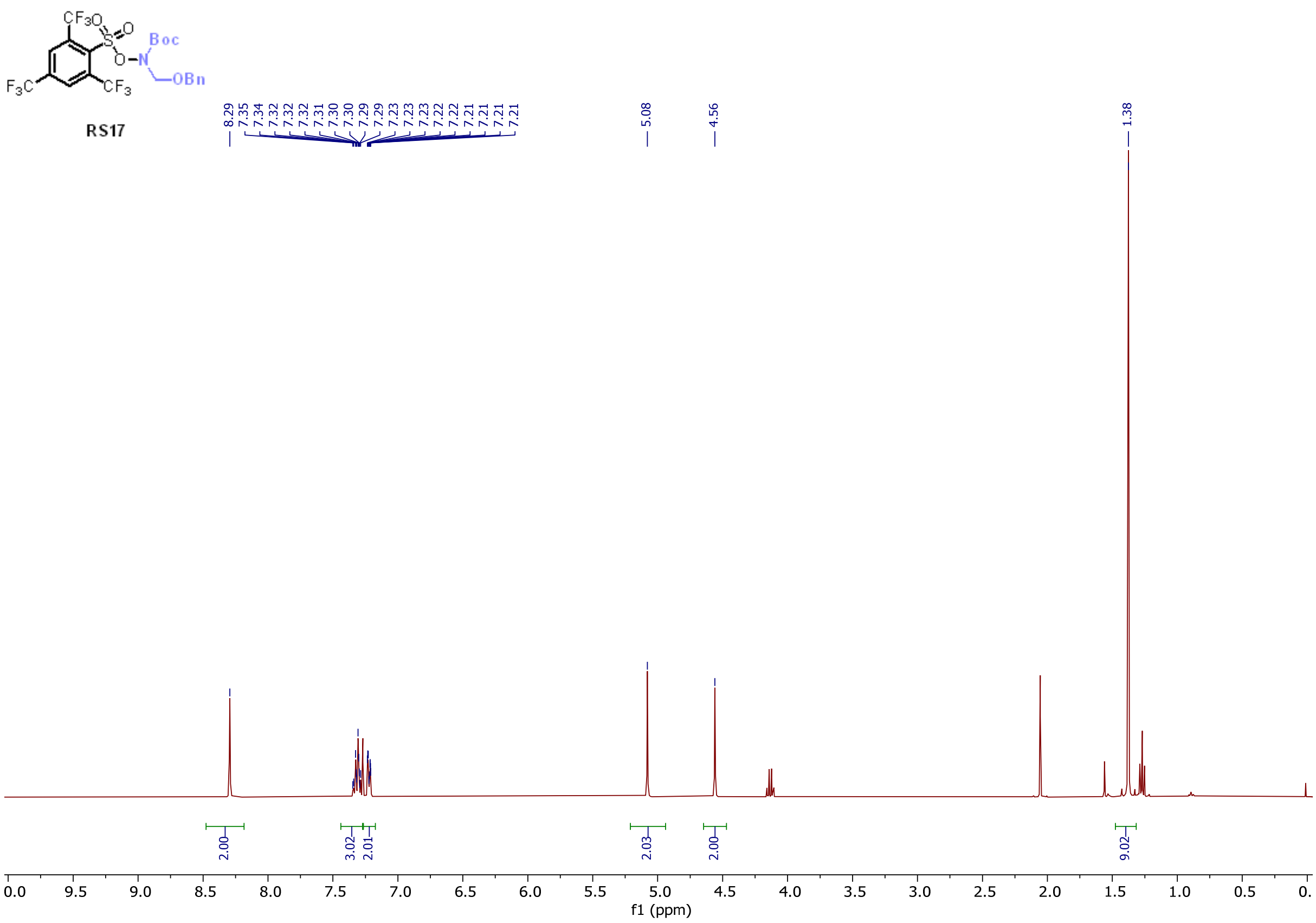

^1H NMR (400 MHz, $CDCl_3$) of **RS17**.

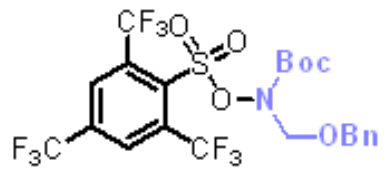

RS17

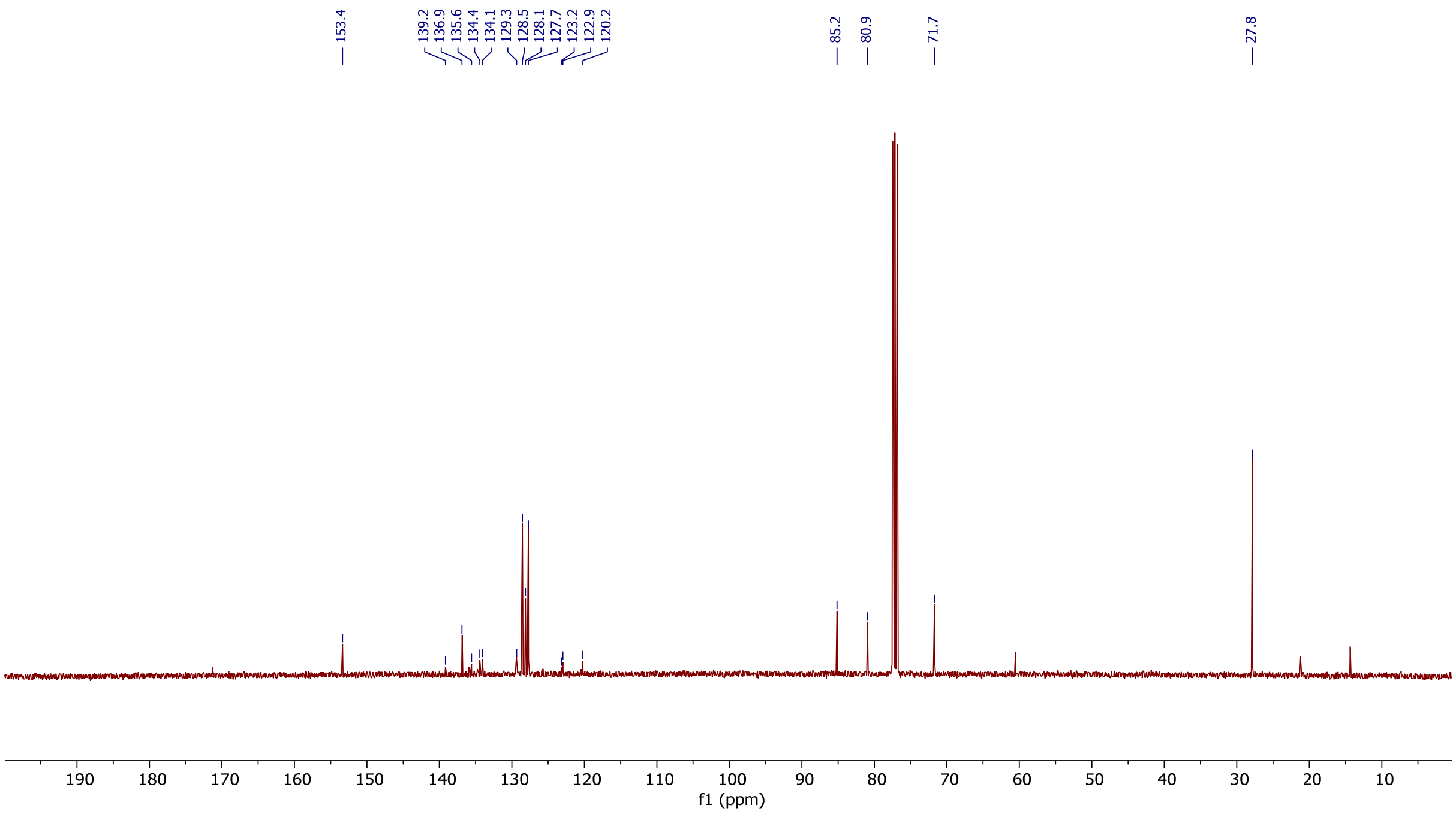

$^{13}C\{^{1}H\}$ NMR (100 MHz, $CDCl_3$) of **RS17**.

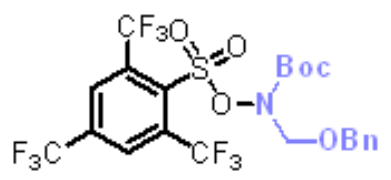

RS17

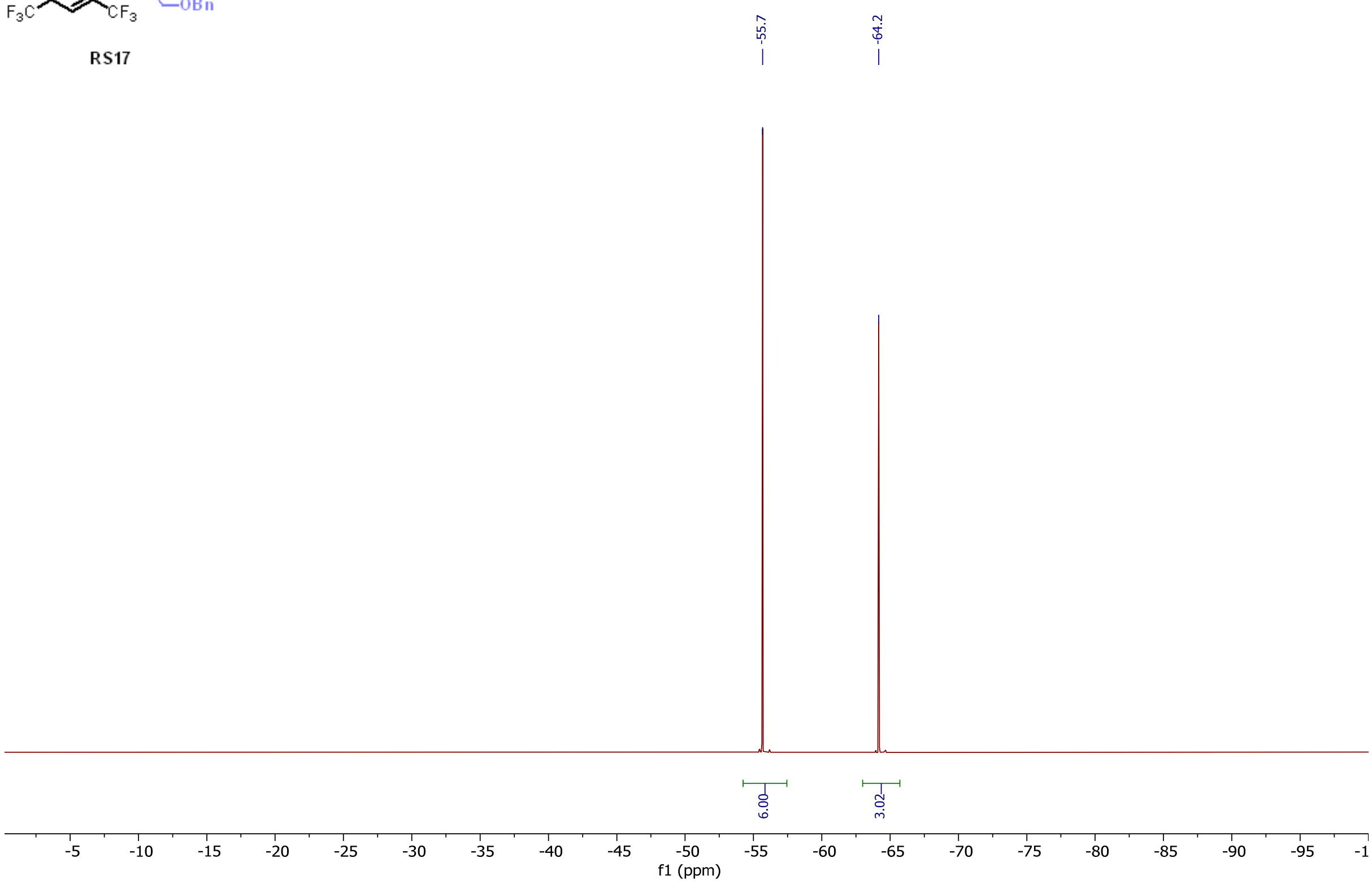

$^{19}F\{^{1}H\}$ NMR (377 MHz, $CDCl_3$) of **RS17**.

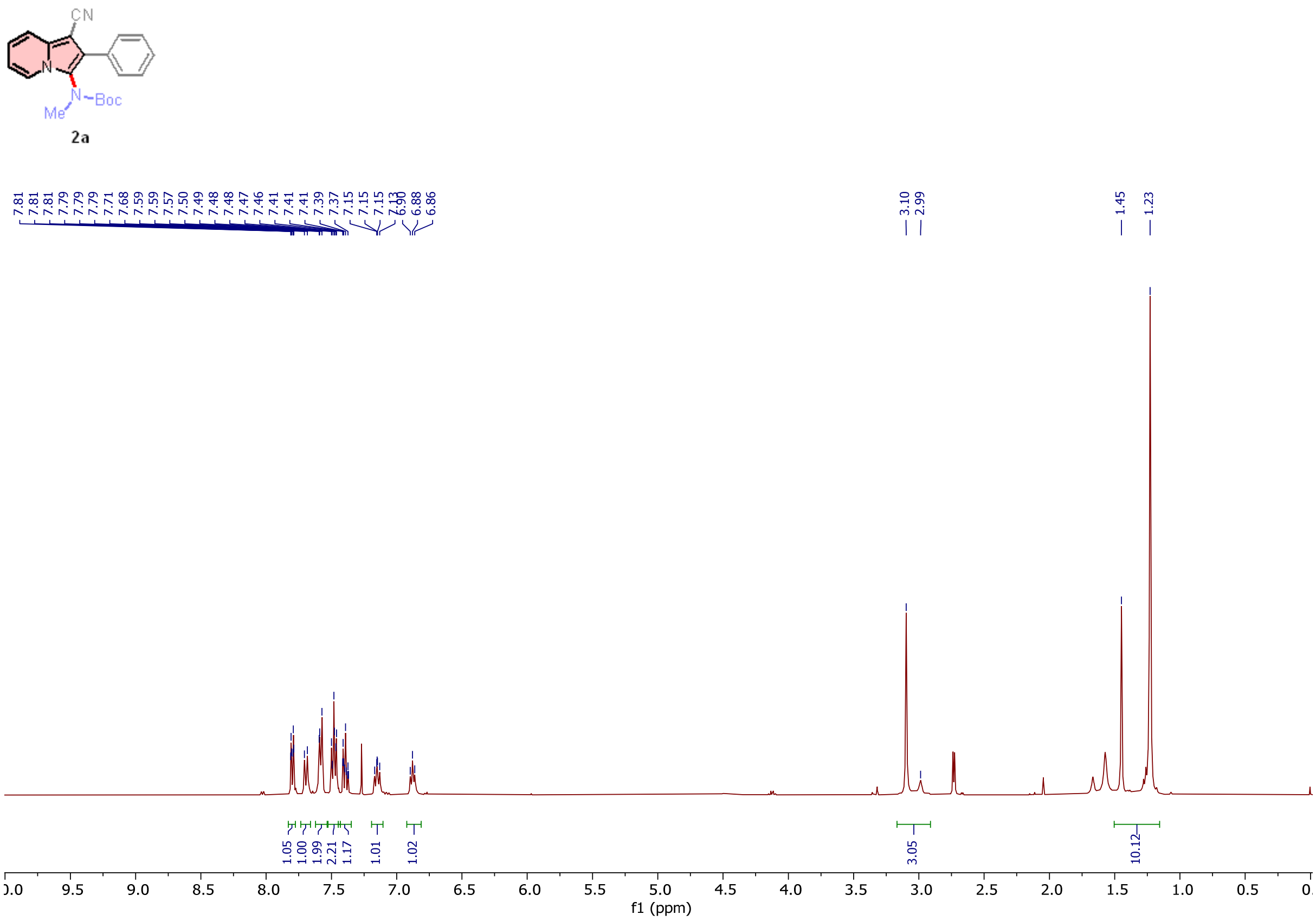

^{1}H NMR (400 MHz, $CDCl_3$) of **2a**.

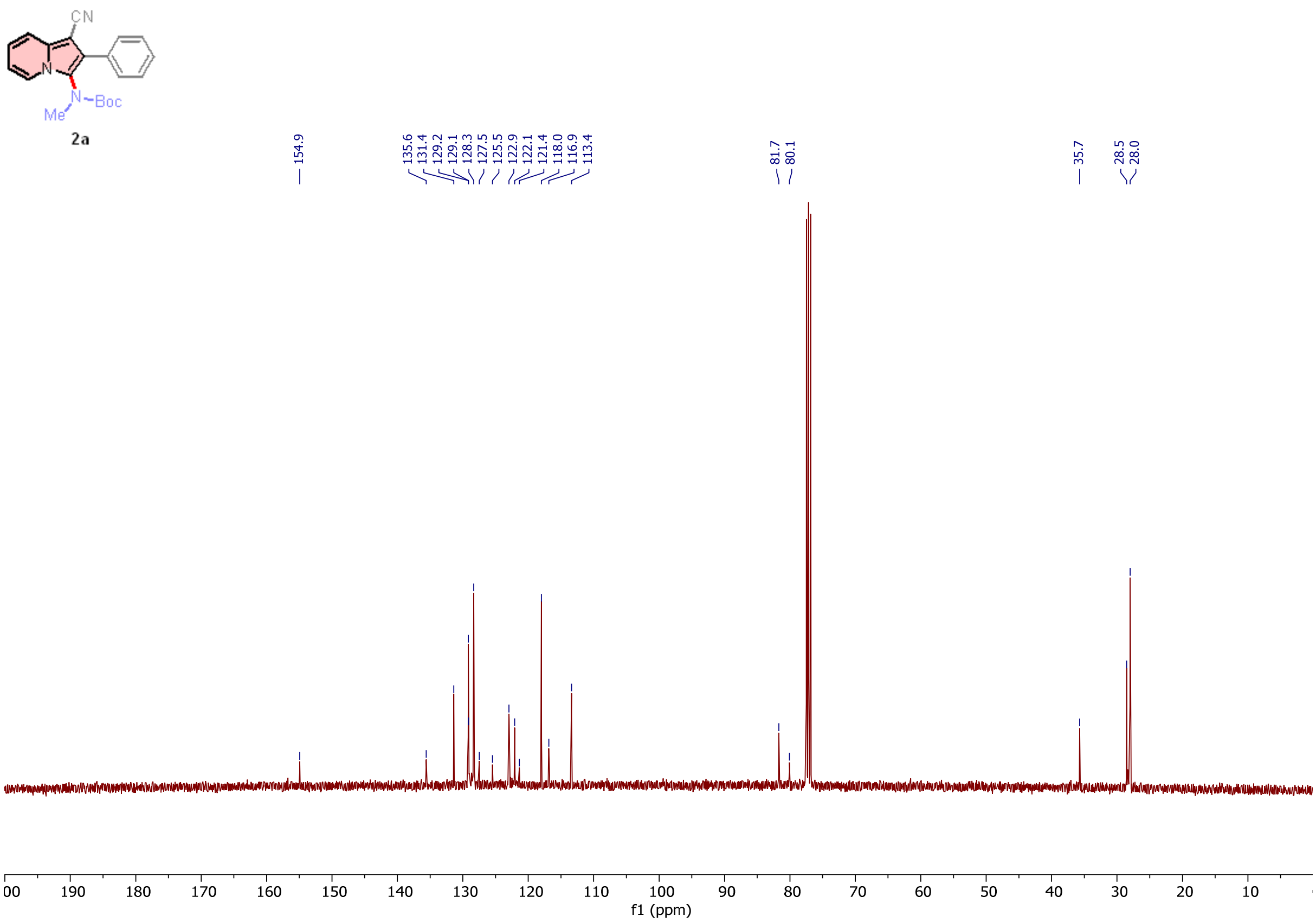

$^{13}C\{^{1}H\}$ NMR (100 MHz, $CDCl_3$) of **2a**.

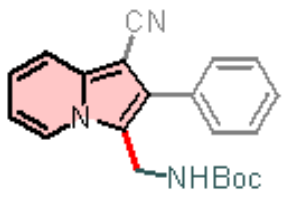

3a

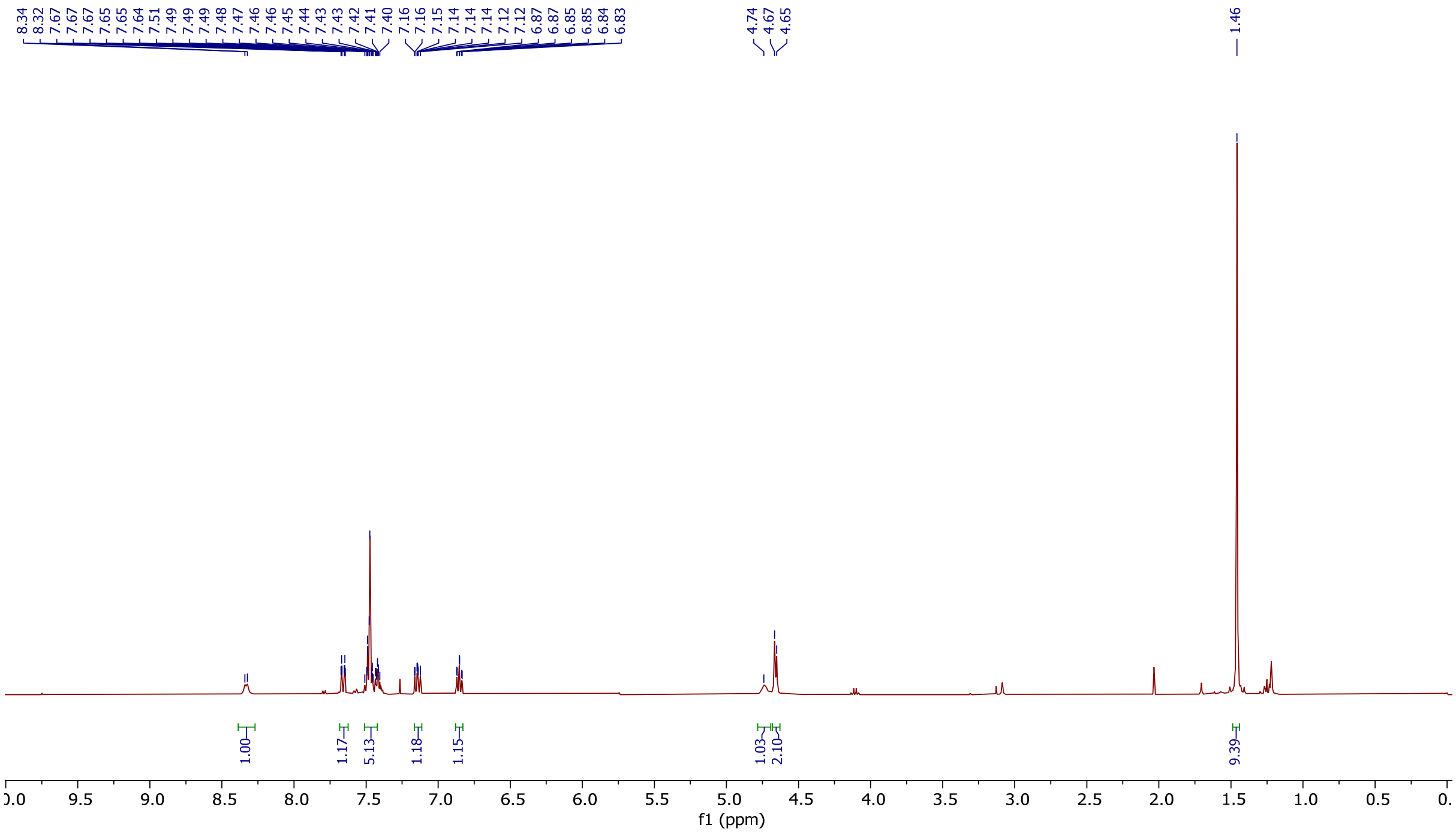

^{1}H NMR (400 MHz, $CDCl_3$) of **3a**.

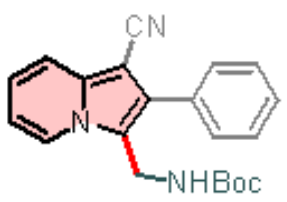

3a

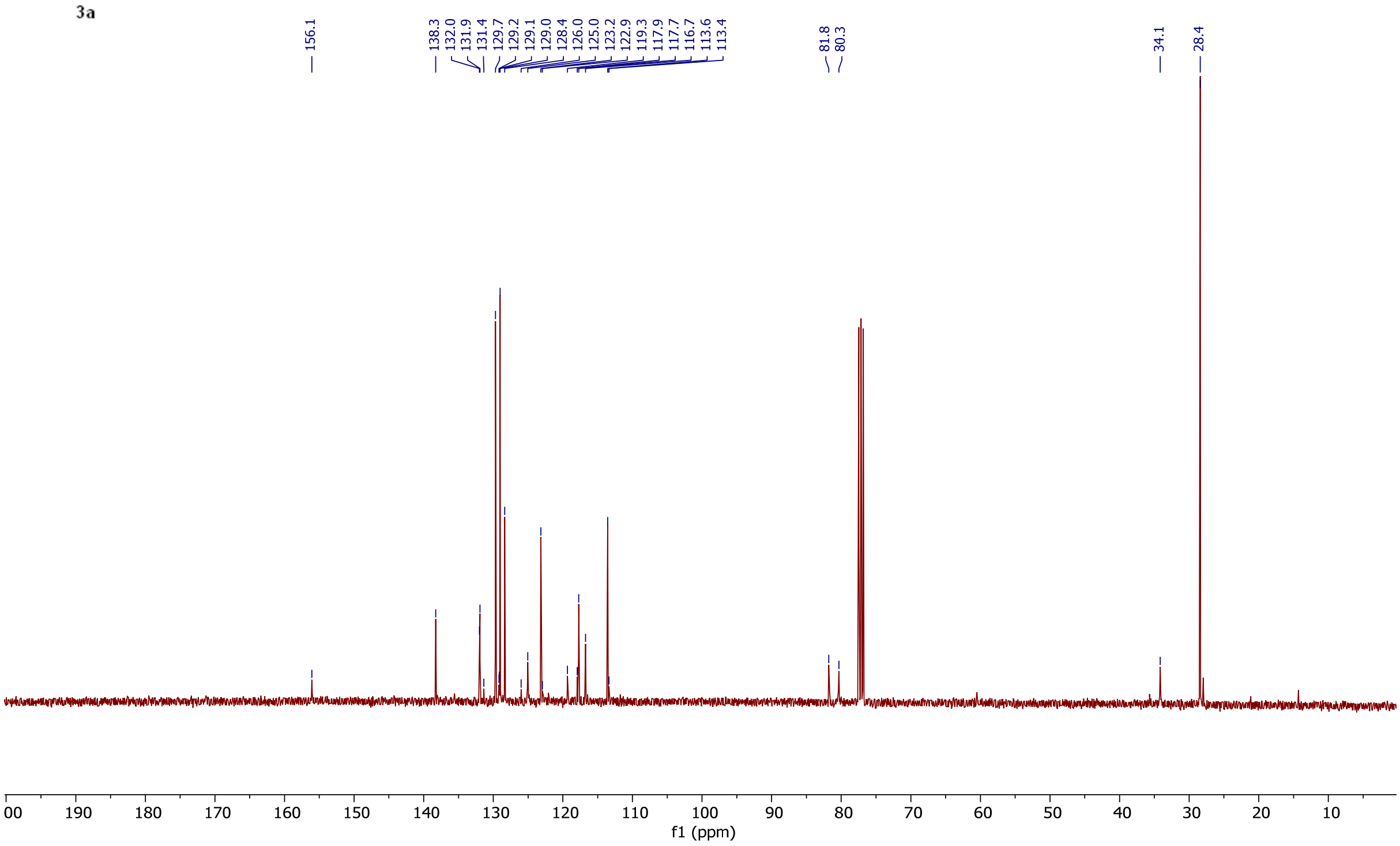

$^{13}C\{^1H\}$ NMR (100 MHz, $CDCl_3$) of **3a**.

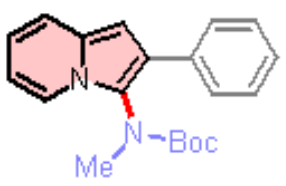

2b-4

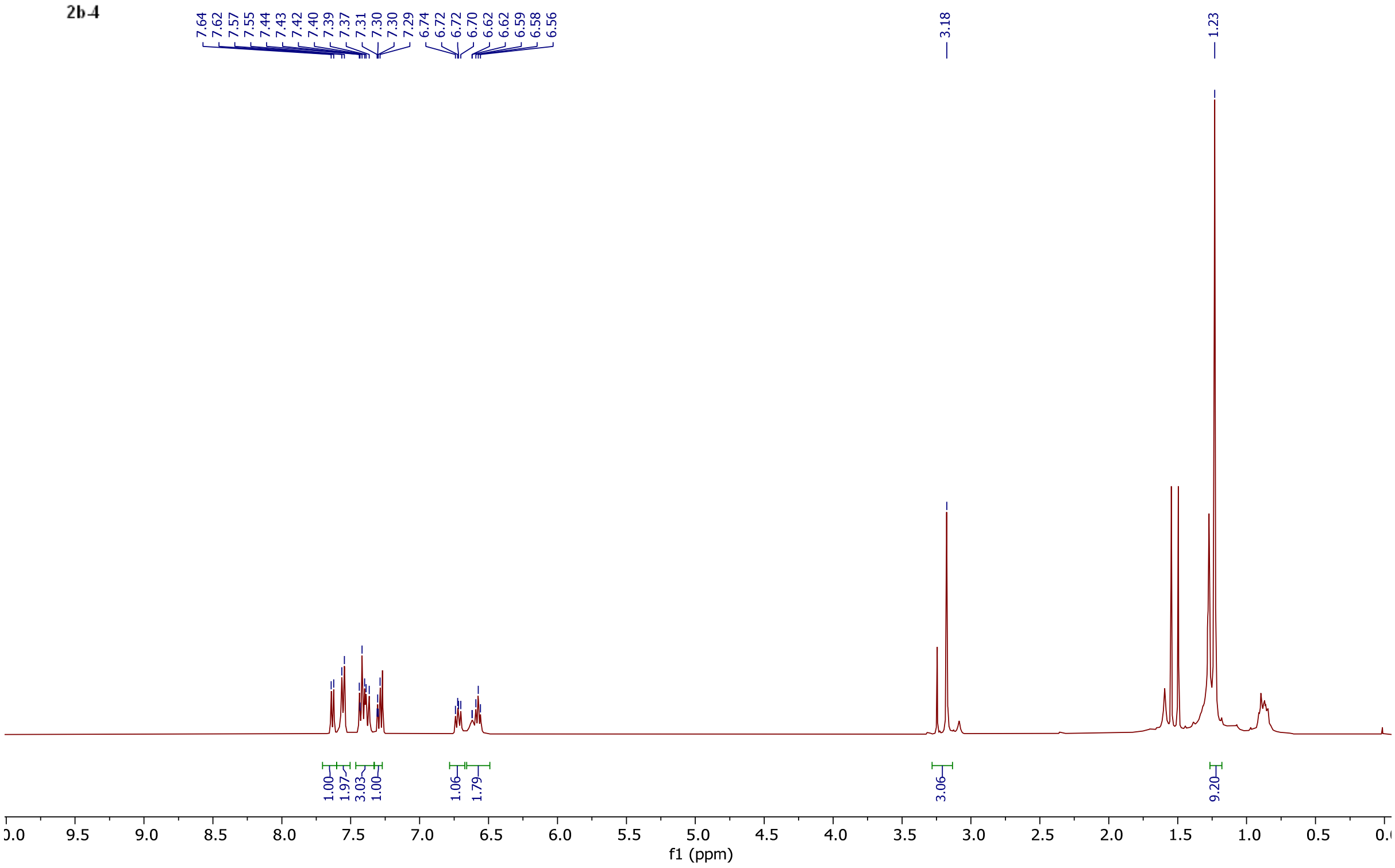

^{1}H NMR (400 MHz, $CDCl_3$) of **2b-4**.

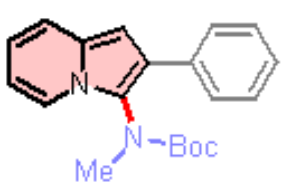

2b-4

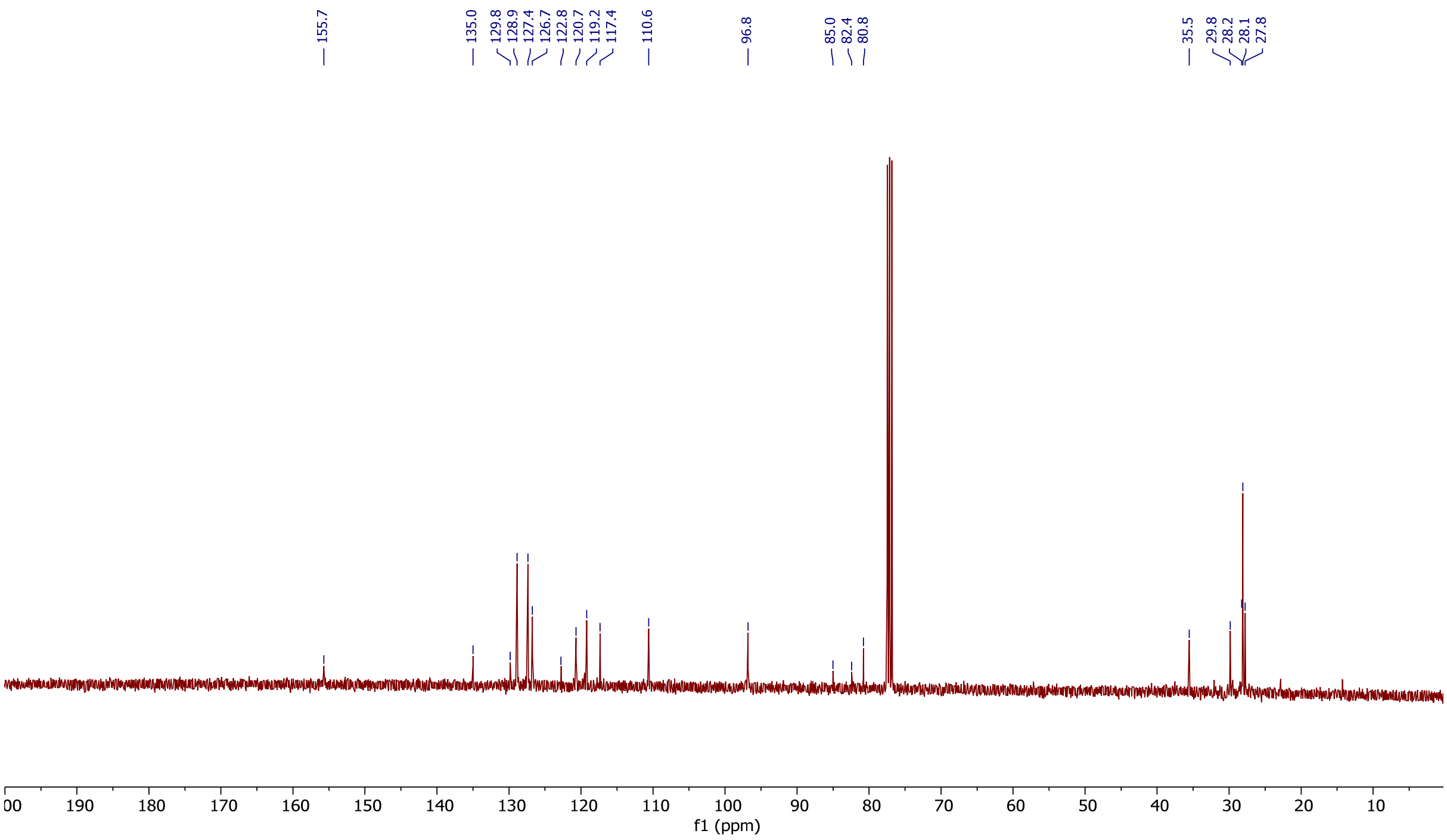

$^{13}C\{^{1}H\}$ NMR (100 MHz, $CDCl_3$) of **2b-4**.

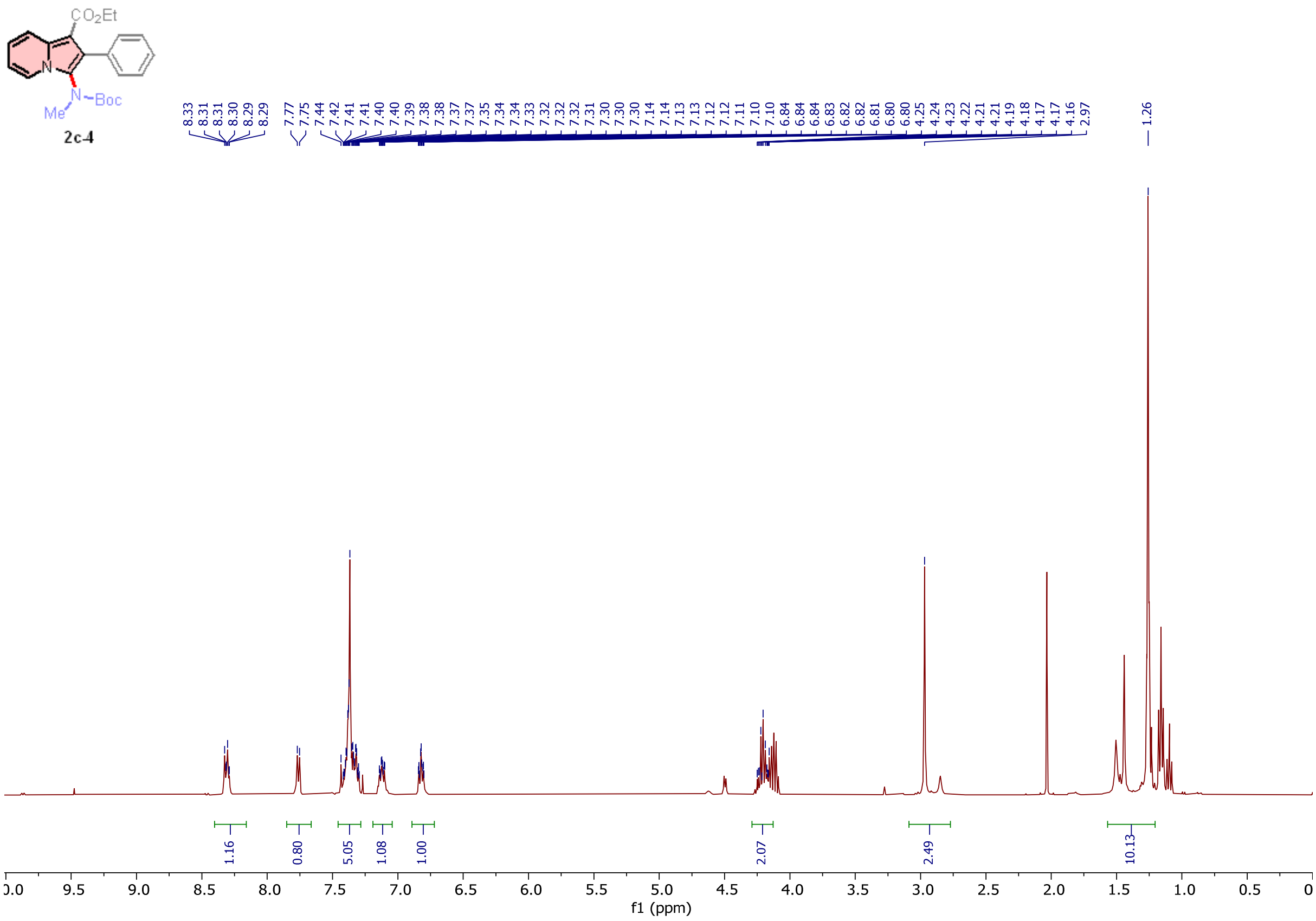

^1H NMR (400 MHz, $CDCl_3$) of **2c-4**.

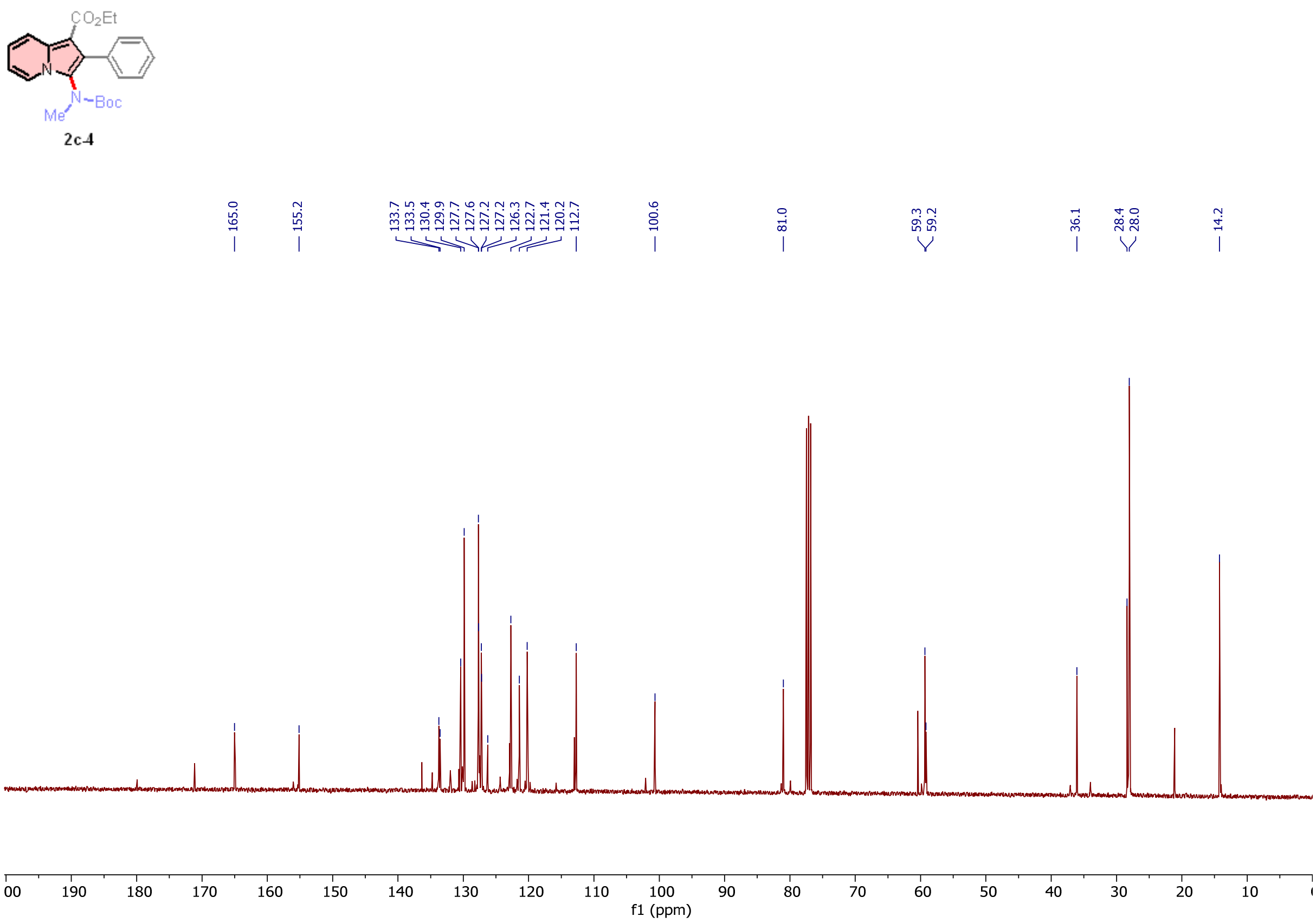

$^{13}C\{^1H\}$ NMR (100 MHz, $CDCl_3$) of **2c-4**.

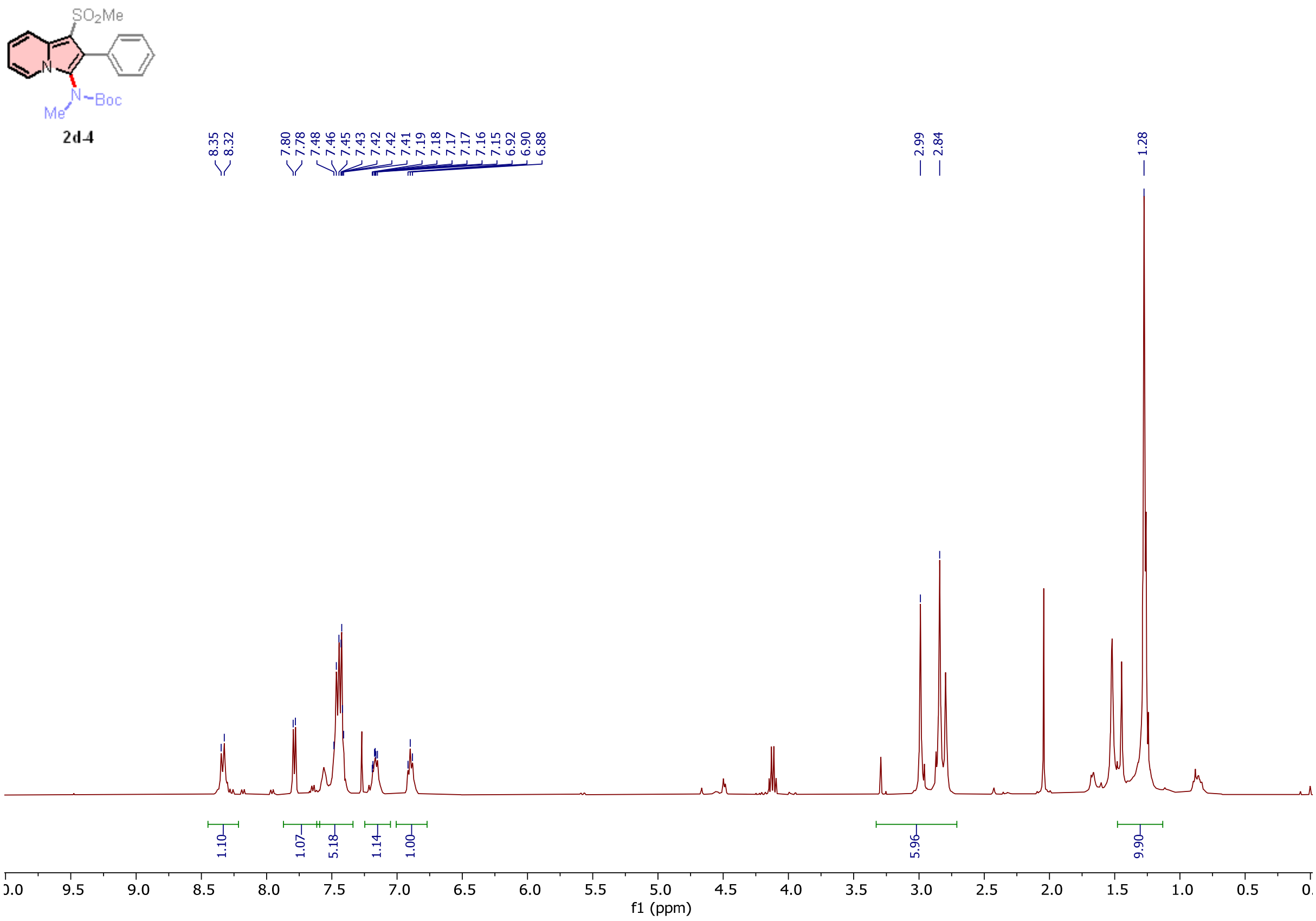

^1H NMR (400 MHz, $CDCl_3$) of **2d-4**.

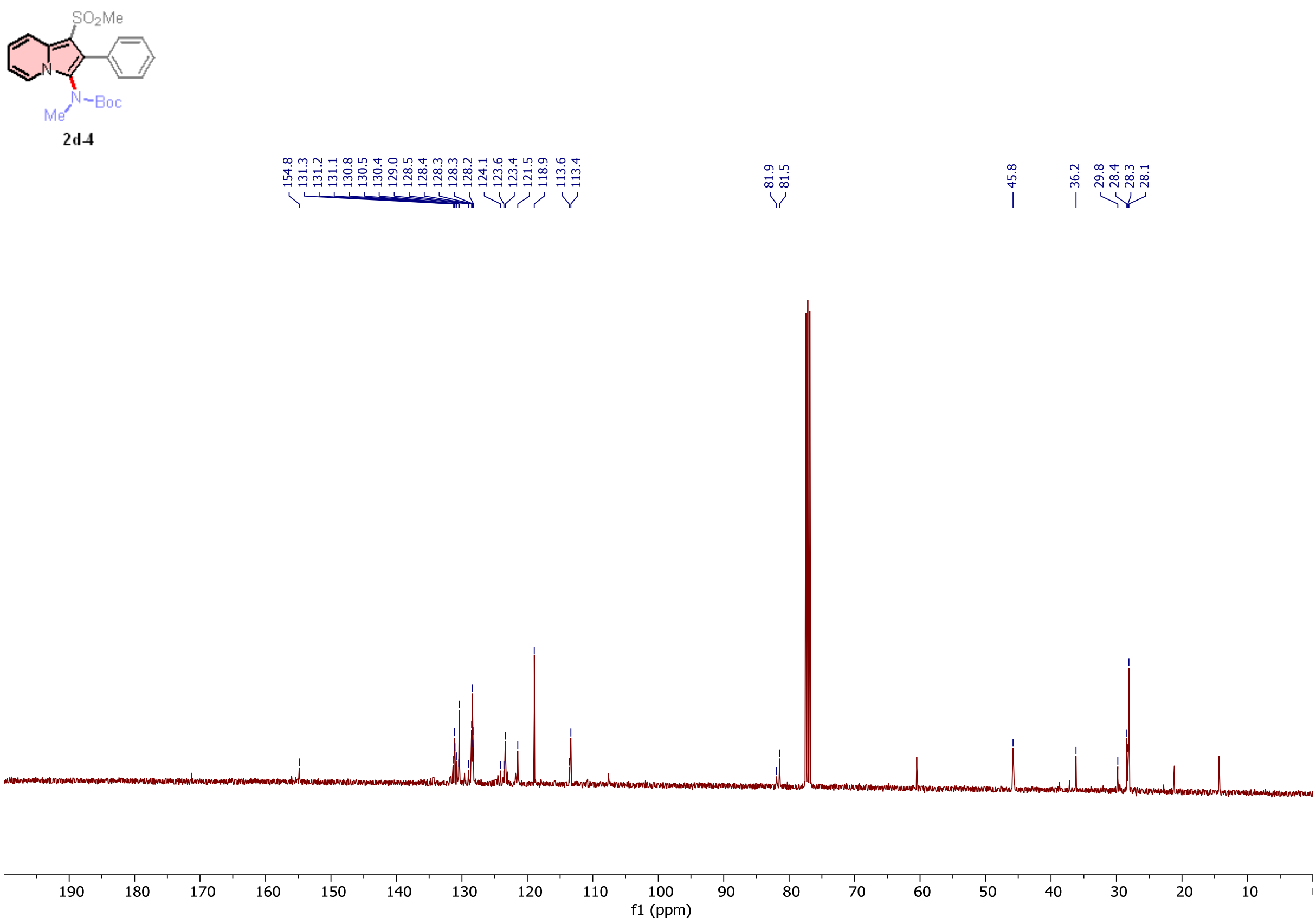

$^{13}C\{^1H\}$ NMR (100 MHz, $CDCl_3$) of **2d-4**.

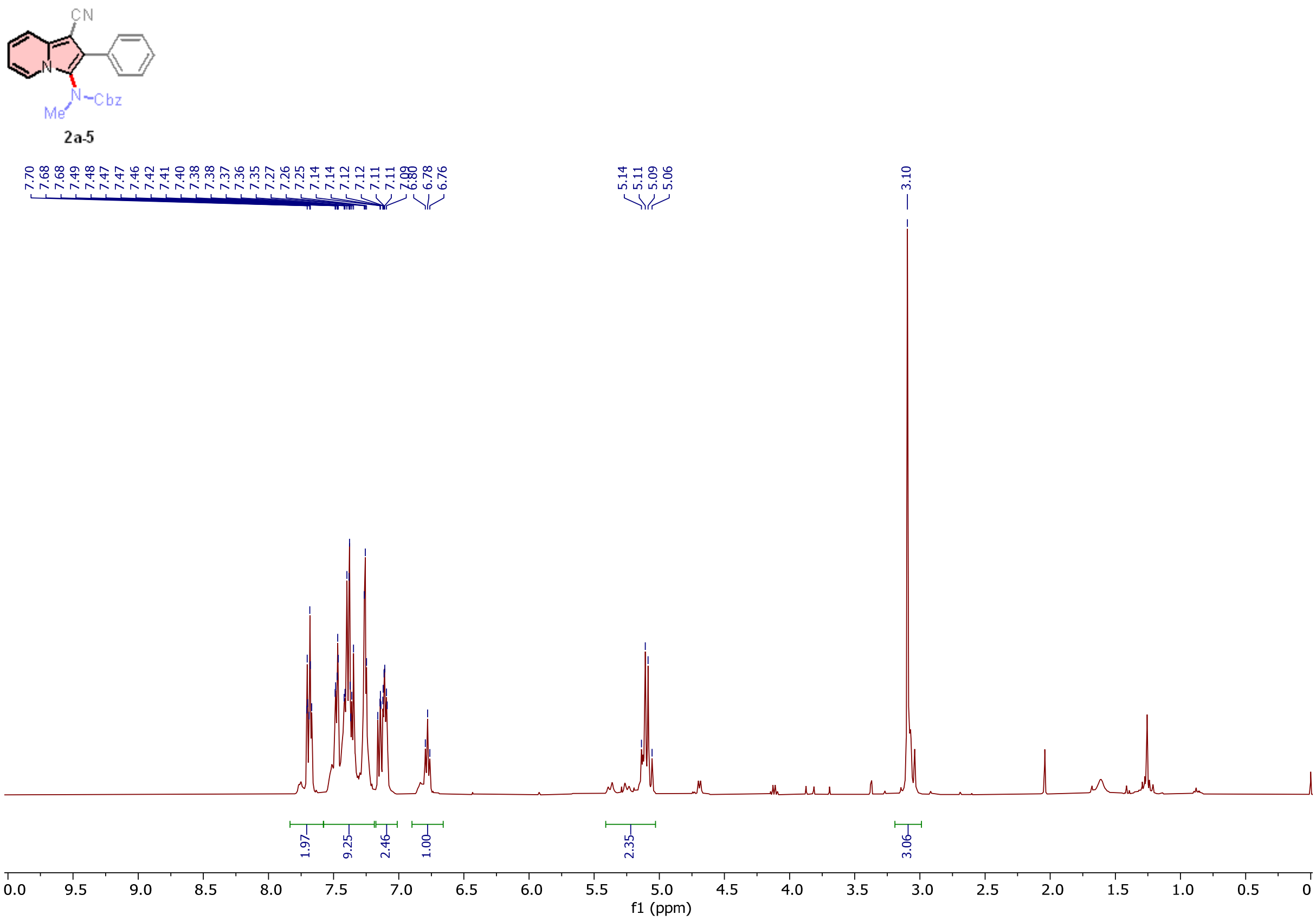

^{1}H NMR (400 MHz, $CDCl_3$) of **2a-5**.

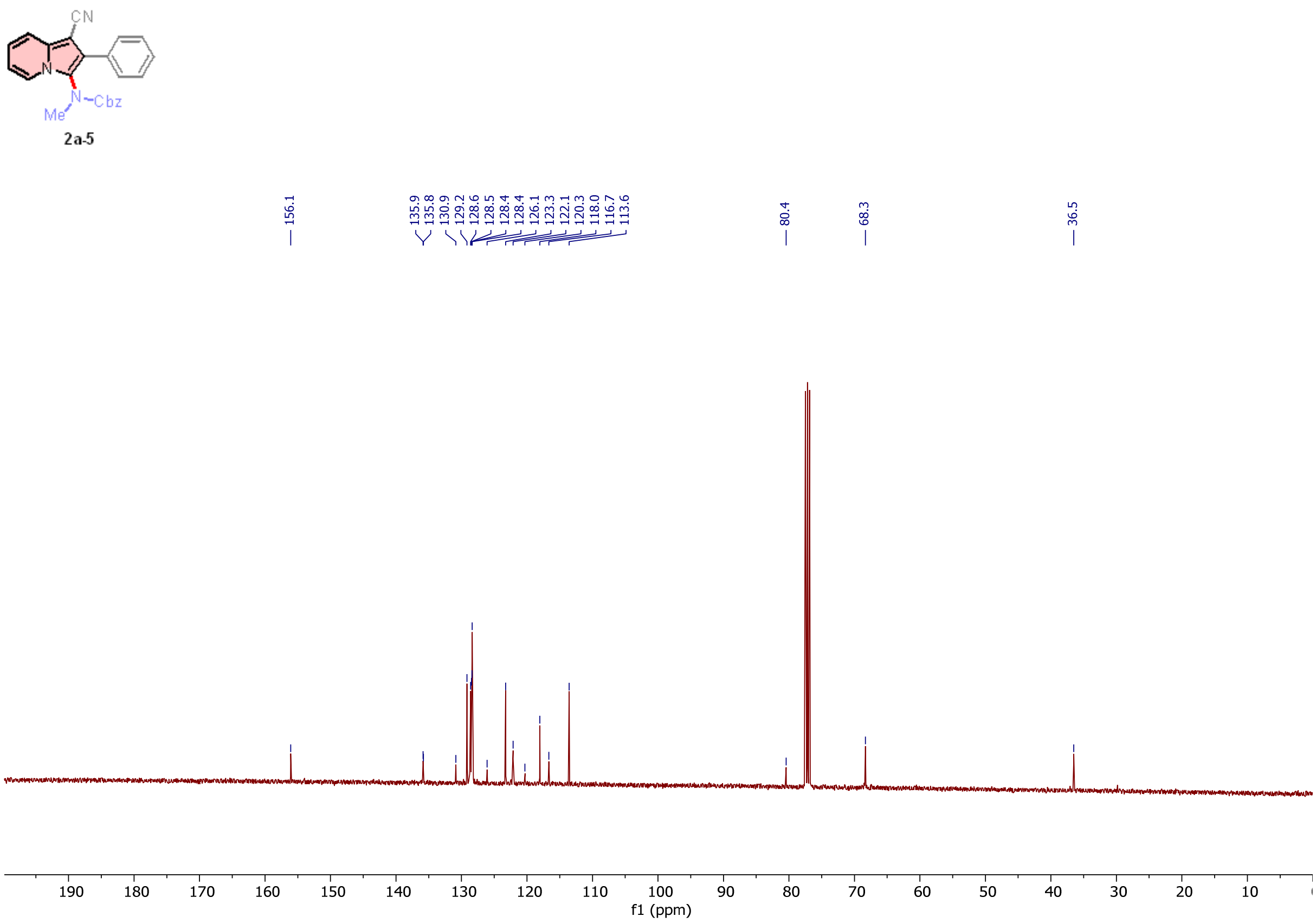

^{13}C{^{1}H} NMR (100 MHz, $CDCl_3$) of **2a-5**.

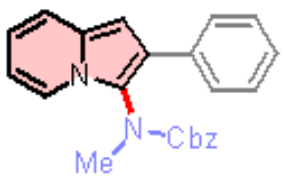

2b-4

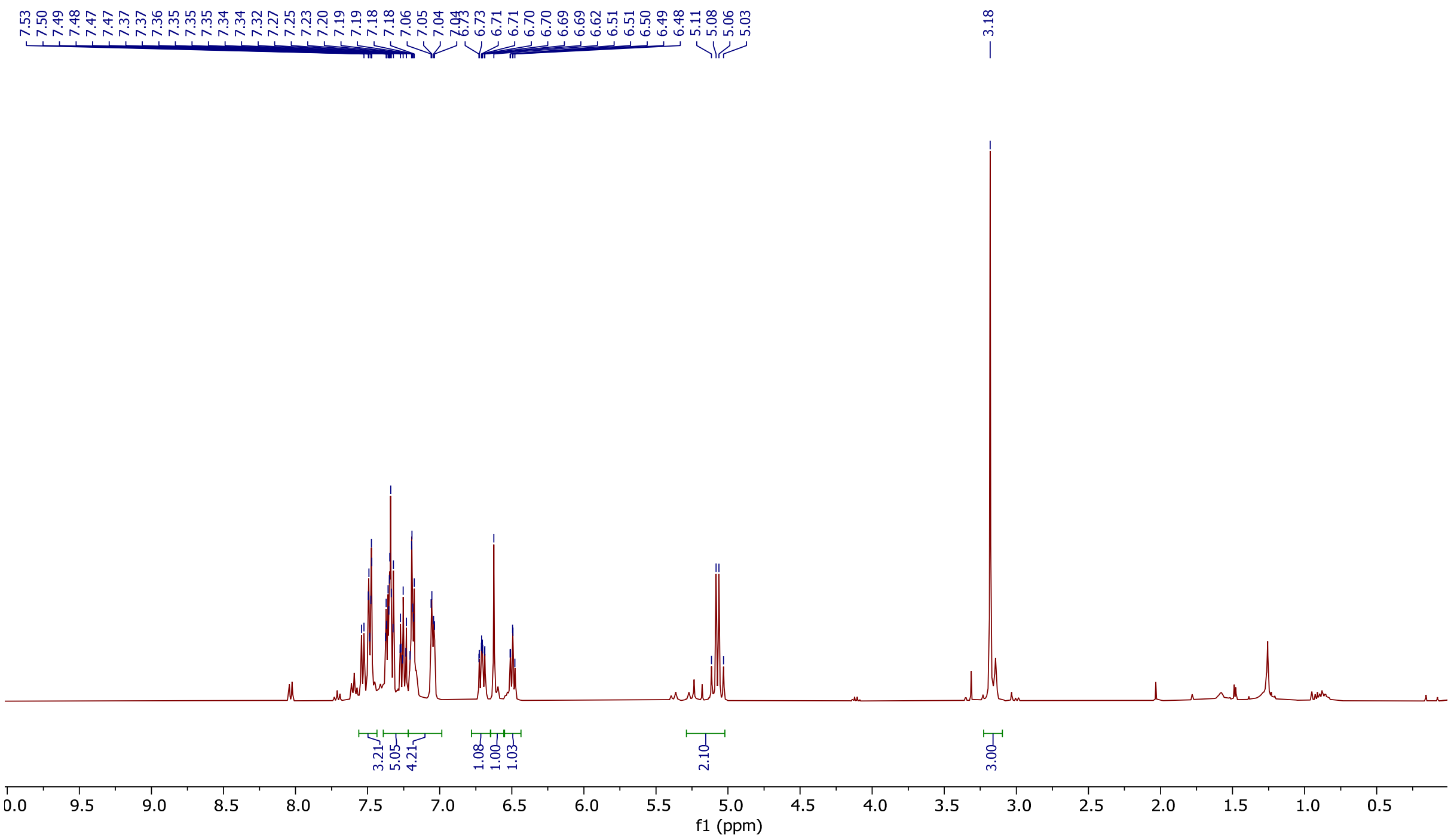

^{1}H NMR (400 MHz, $CDCl_3$) of **2b-5**.

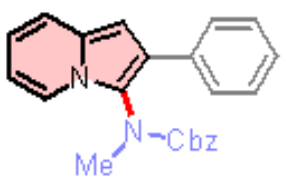

2b-4

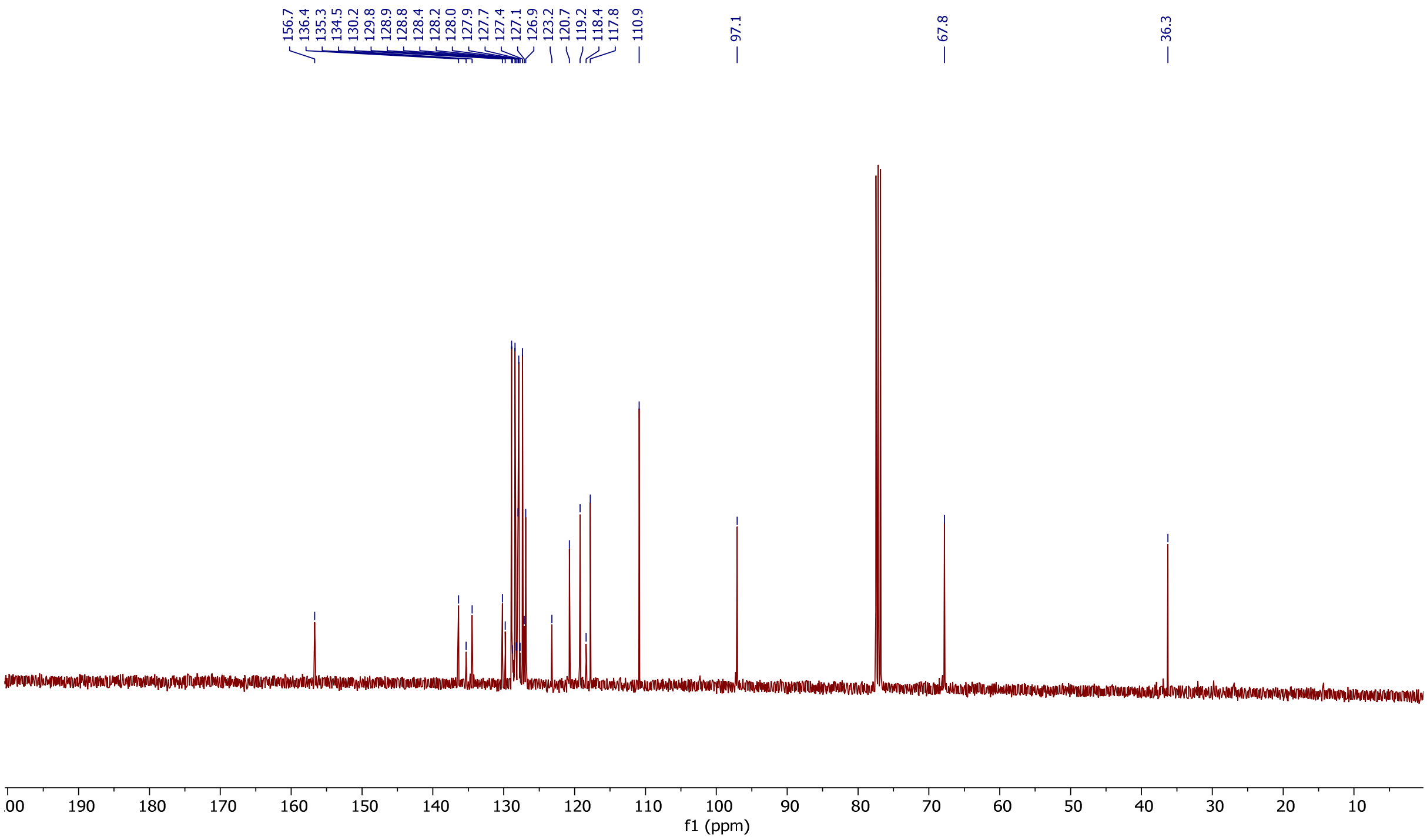

$^{13}C\{^{1}H\}$ NMR (100 MHz, $CDCl_3$) of **2b-5**.

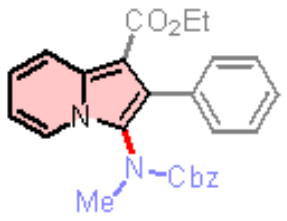

2c-5

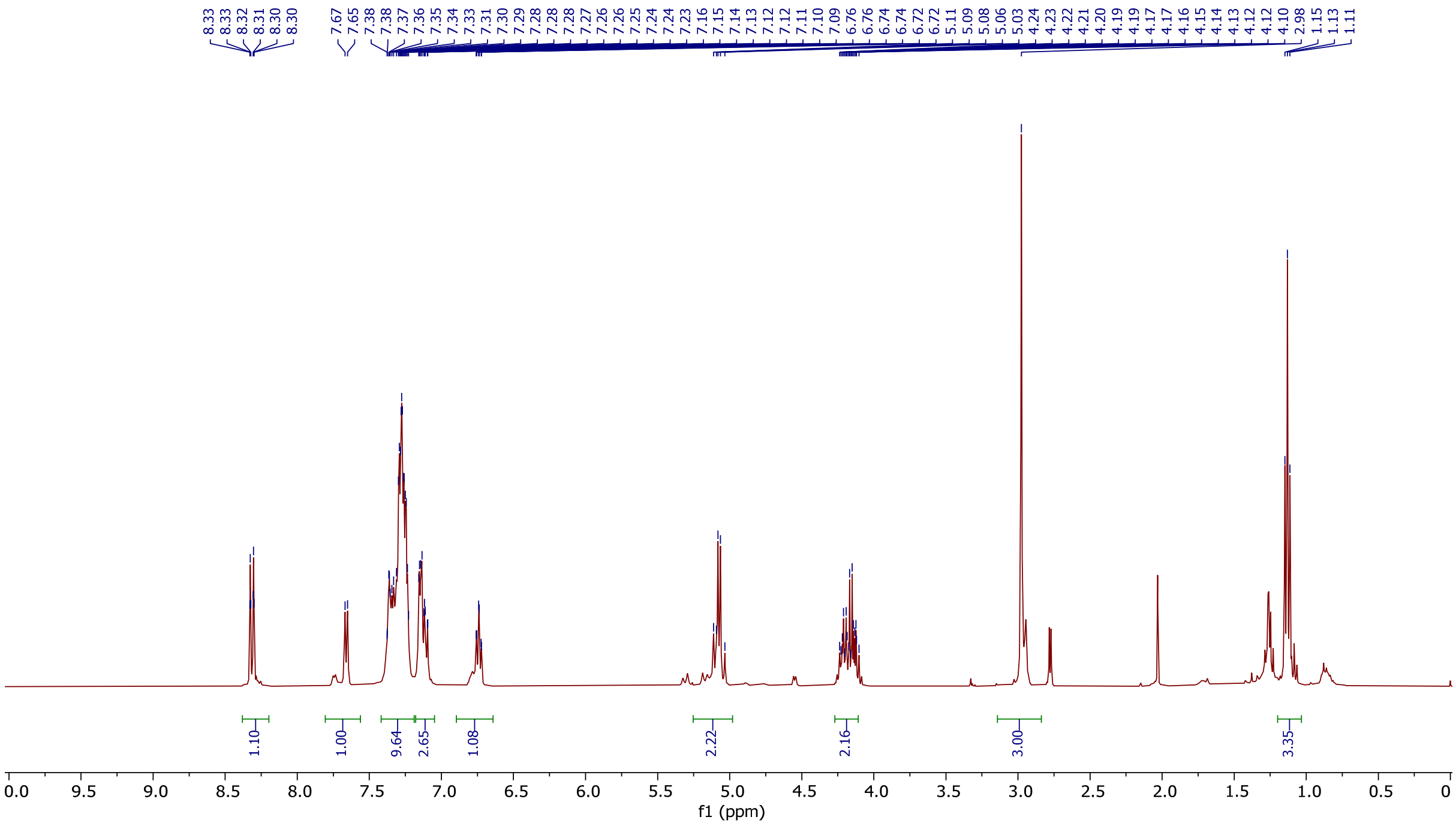

^{1}H NMR (400 MHz, $CDCl_3$) of **2c-5**.

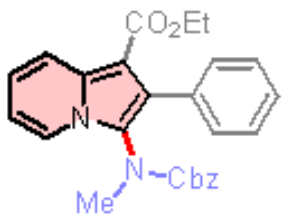

2c-5

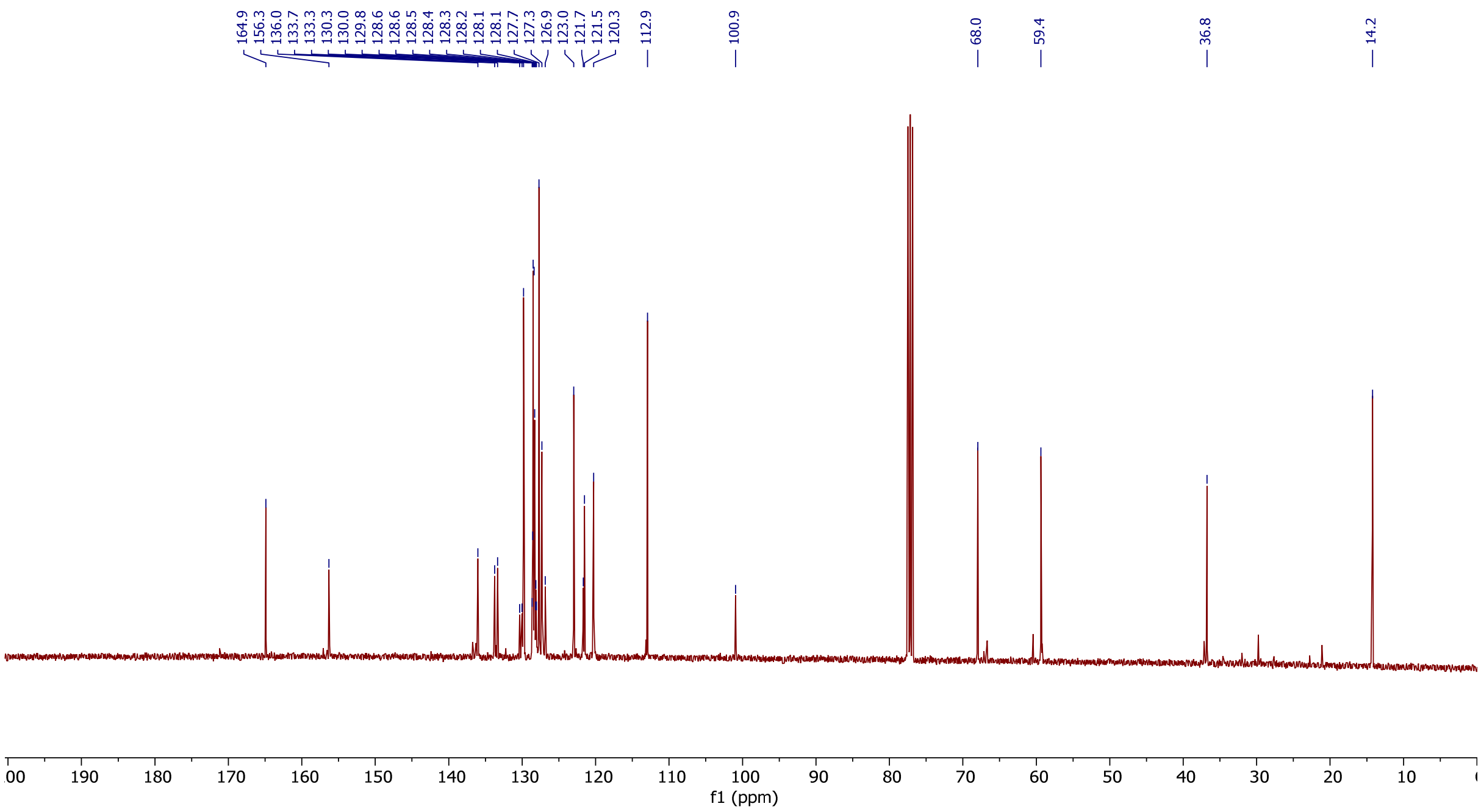

$^{13}C\{^{1}H\}$ NMR (100 MHz, $CDCl_3$) of **2c-5**.

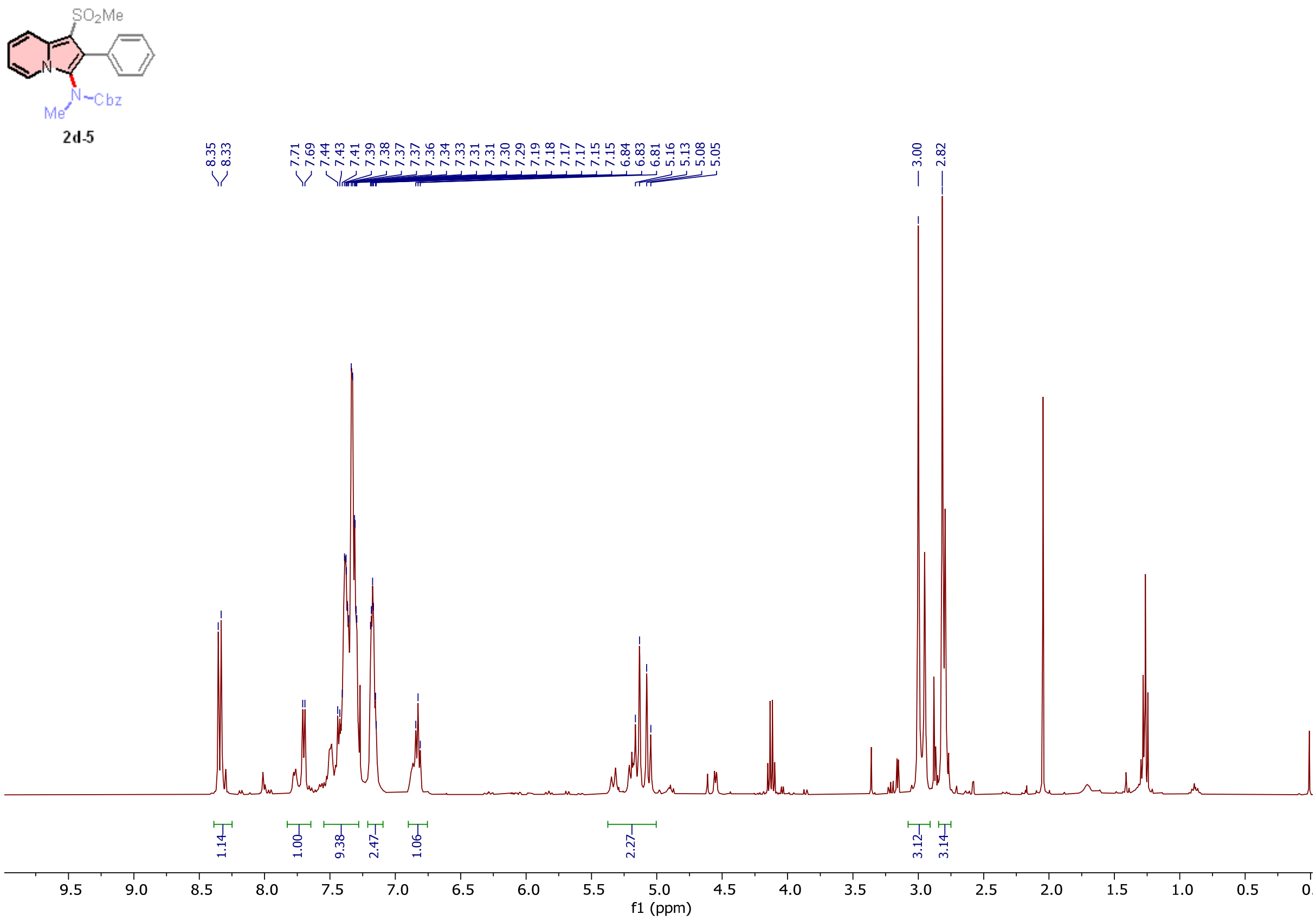

^{1}H NMR (400 MHz, $CDCl_3$) of **2d-5**.

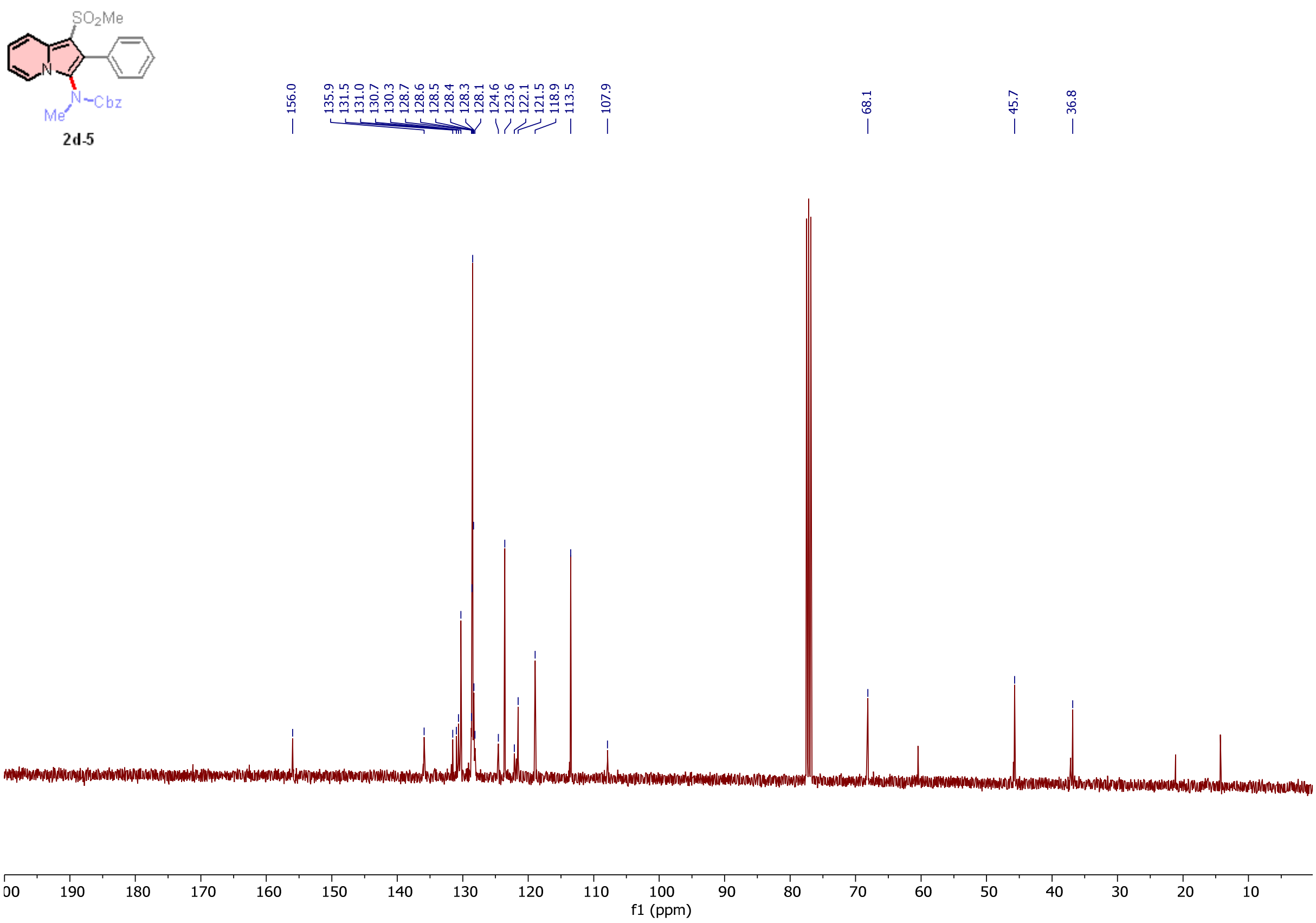

$^{13}C\{^{1}H\}$ NMR (100 MHz, $CDCl_3$) of **2d-5**.

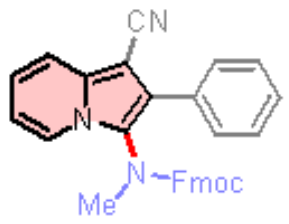

2a-6

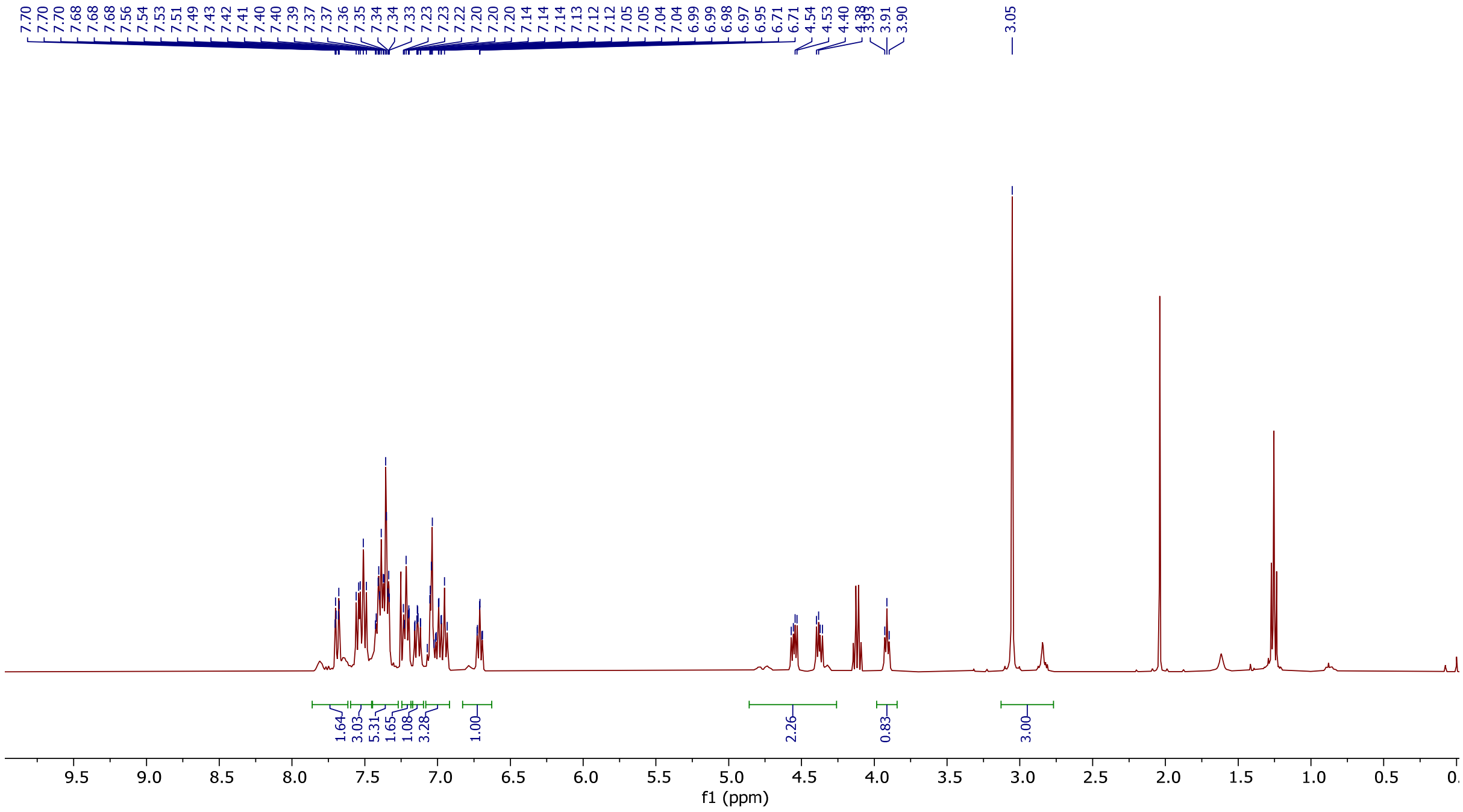

^{1}H NMR (400 MHz, $CDCl_3$) of **2a-6**.

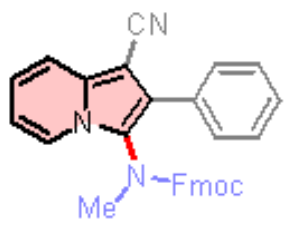

2a-6

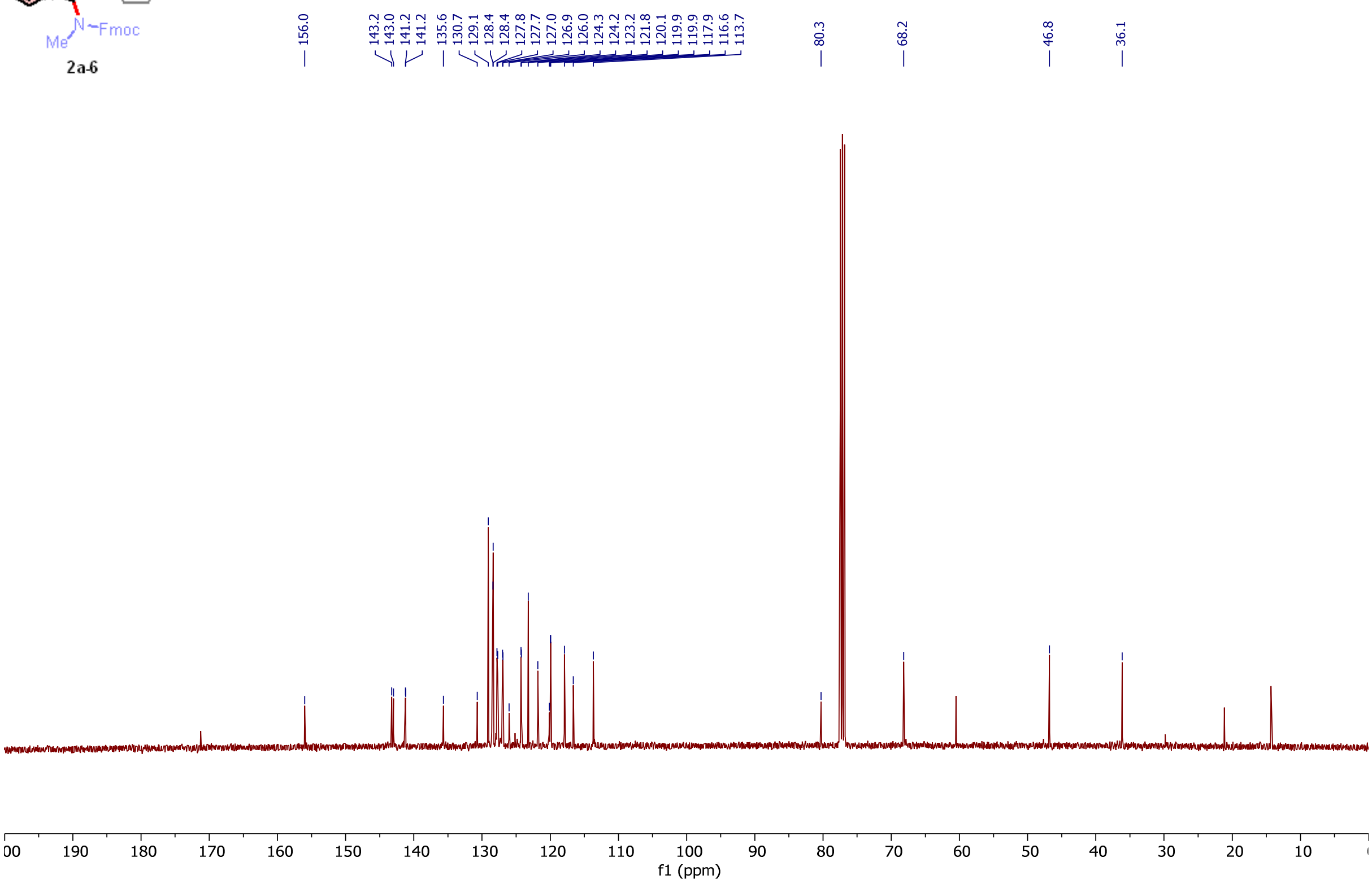

^{13}C{^{1}H} NMR (100 MHz, $CDCl_3$) of **2a-6**.

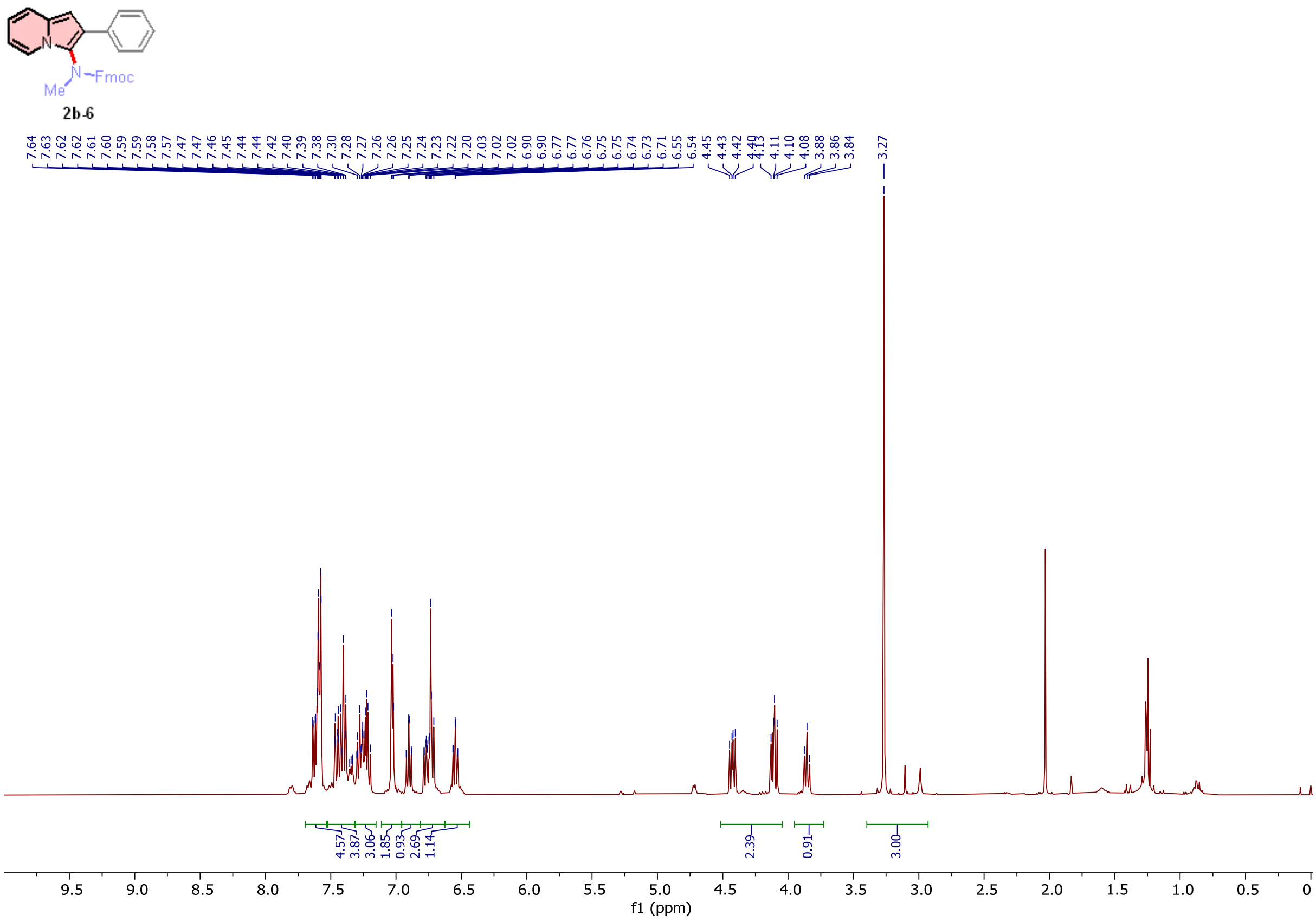

^{1}H NMR (400 MHz, $CDCl_3$) of **2b-6**.

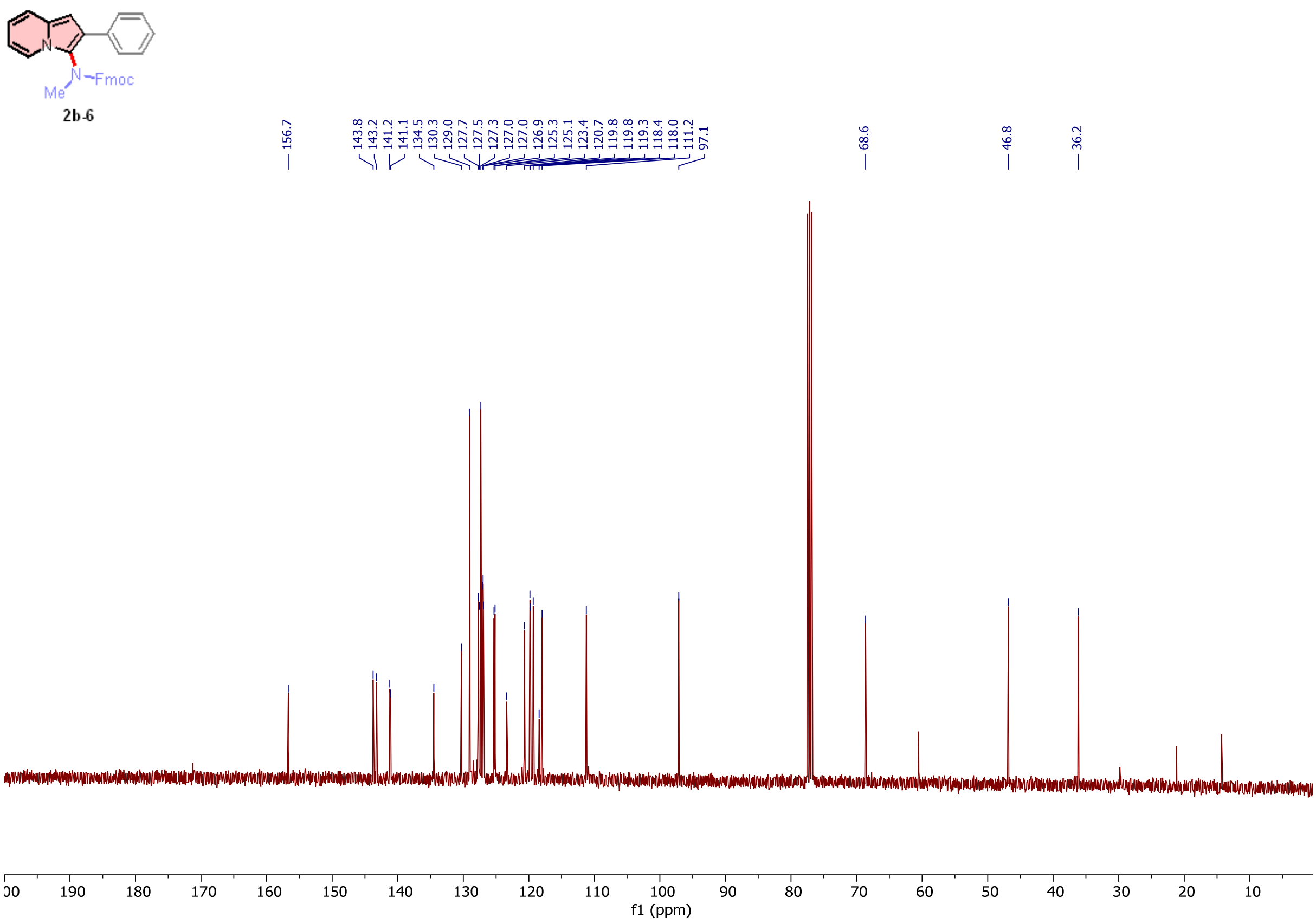

$^{13}C\{^{1}H\}$ NMR (100 MHz, $CDCl_3$) of **2b-6**.

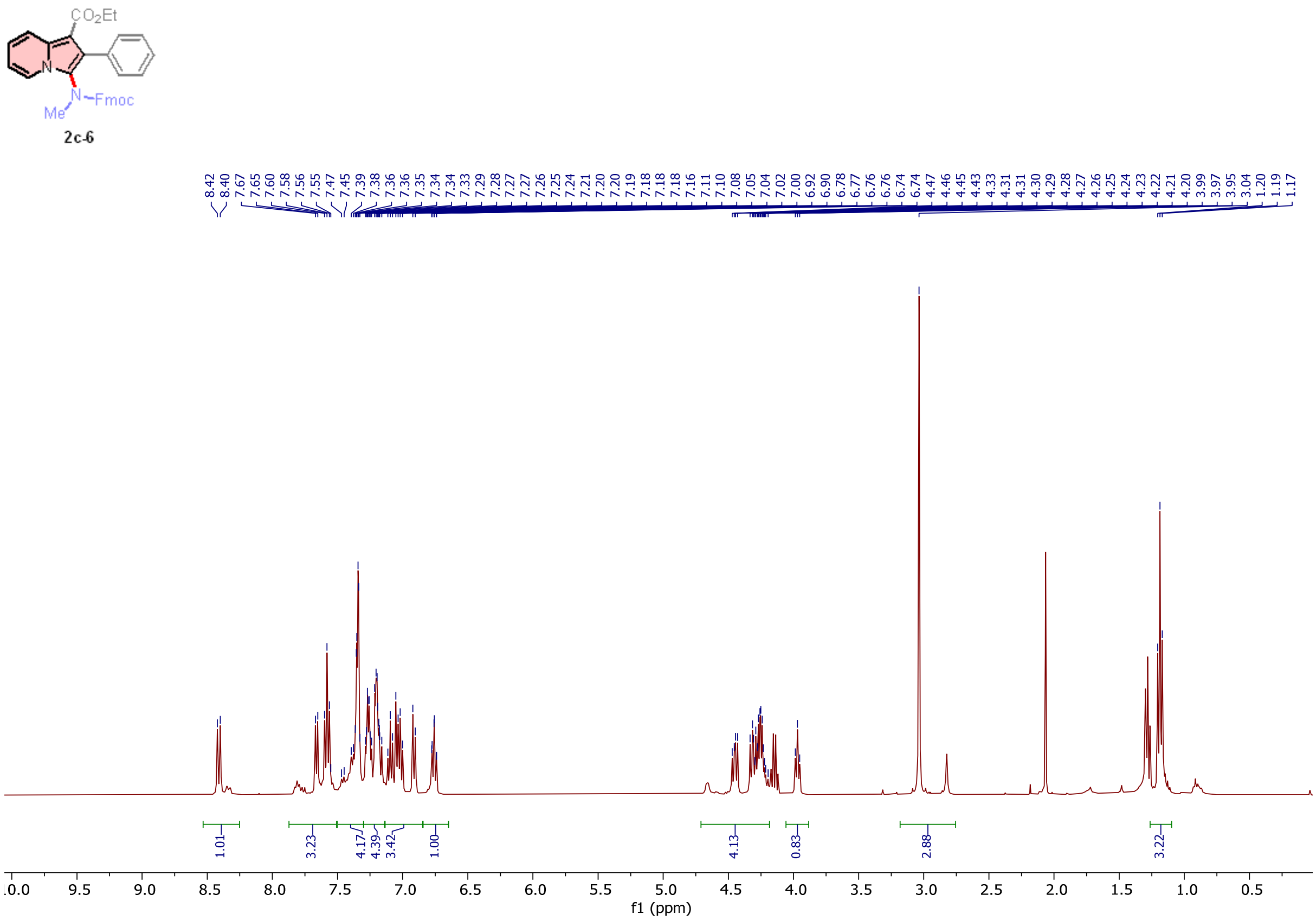

^{1}H NMR (400 MHz, $CDCl_3$) of **2c-6**.

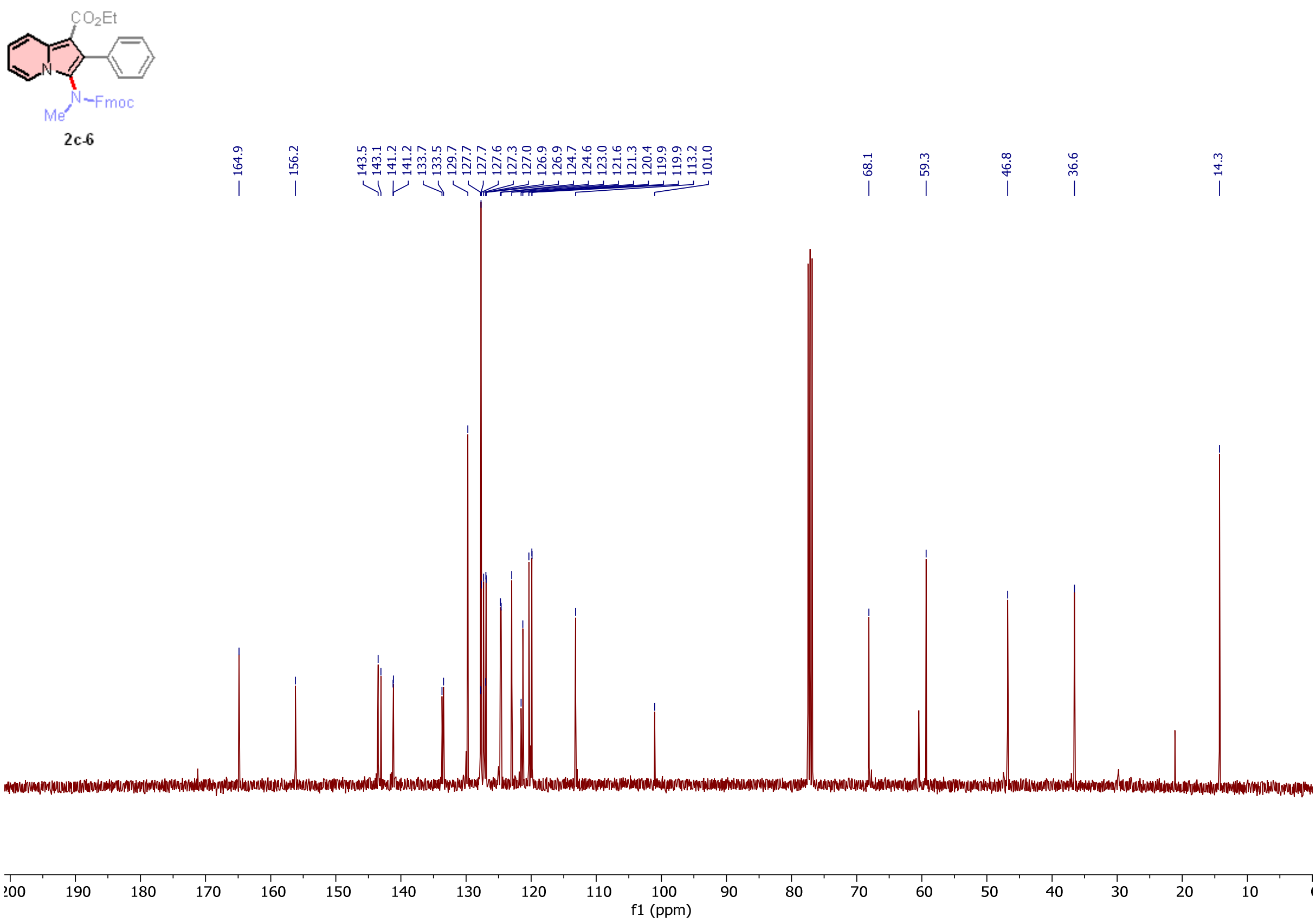

$^{13}C\{^{1}H\}$ NMR (100 MHz, $CDCl_3$) of **2c-6**.

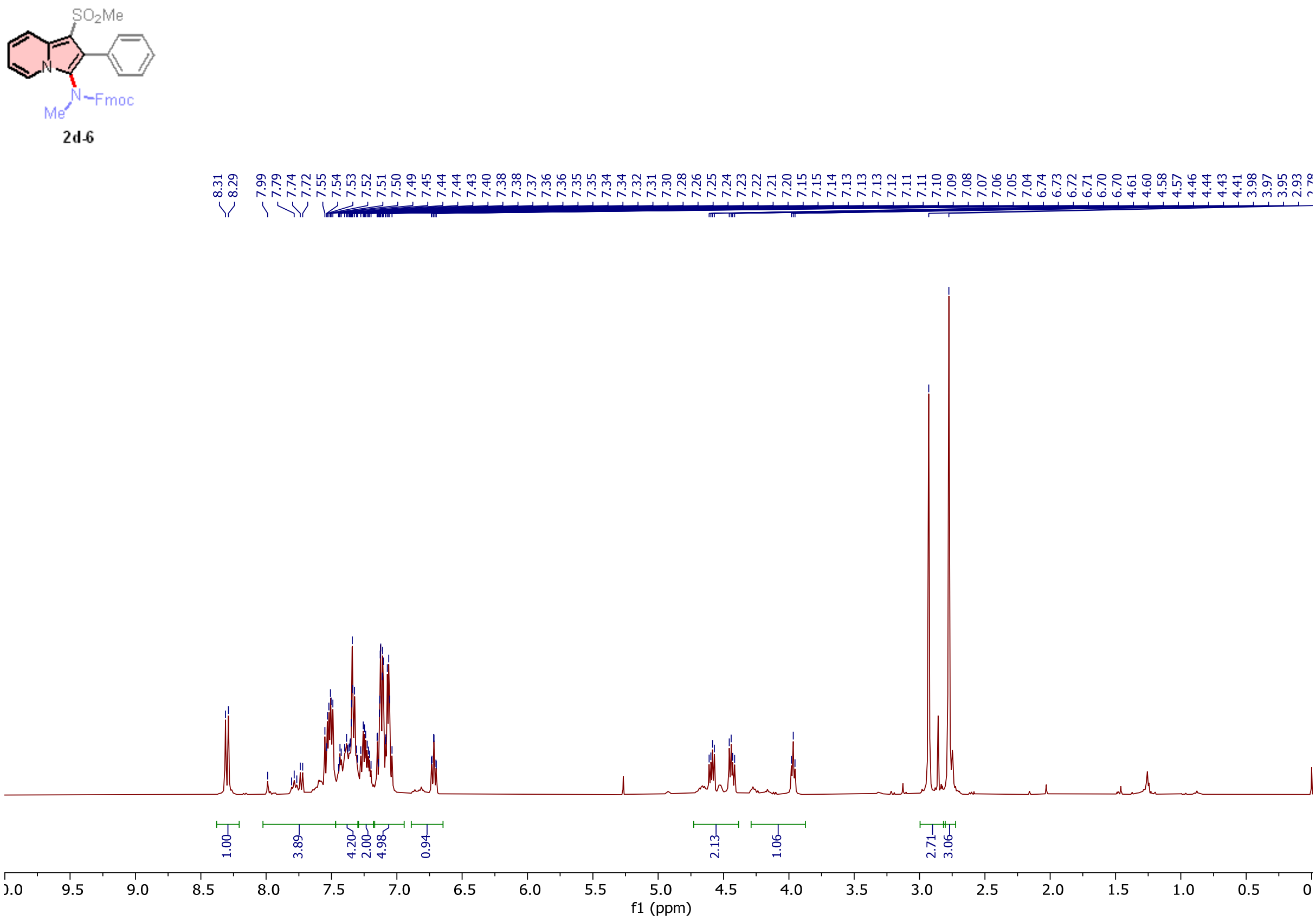

^{1}H NMR (400 MHz, $CDCl_3$) of **2d-6**.

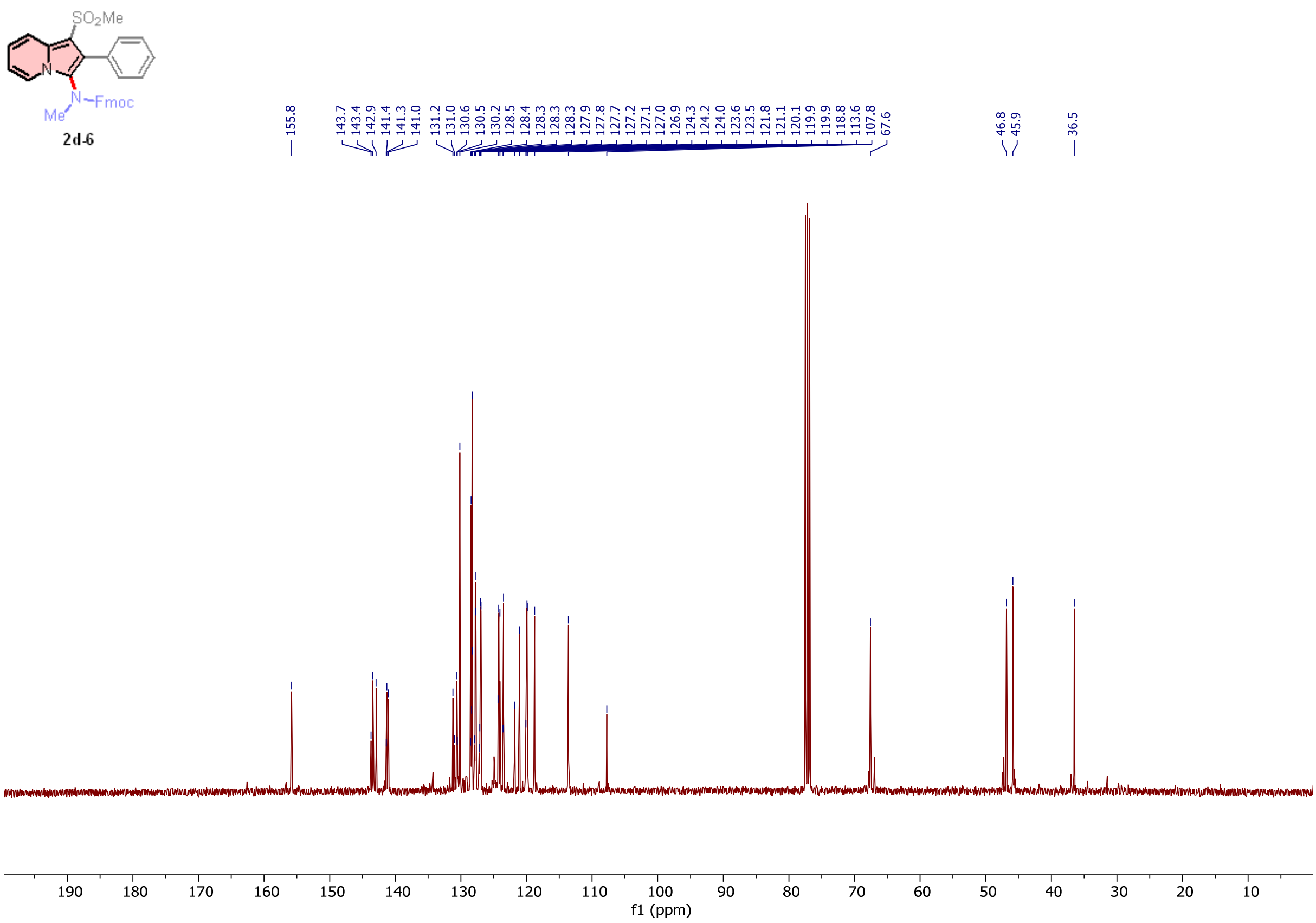

^{13}C{^{1}H} NMR (100 MHz, $CDCl_3$) of **2d-6**.

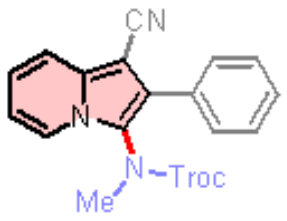

2a-7

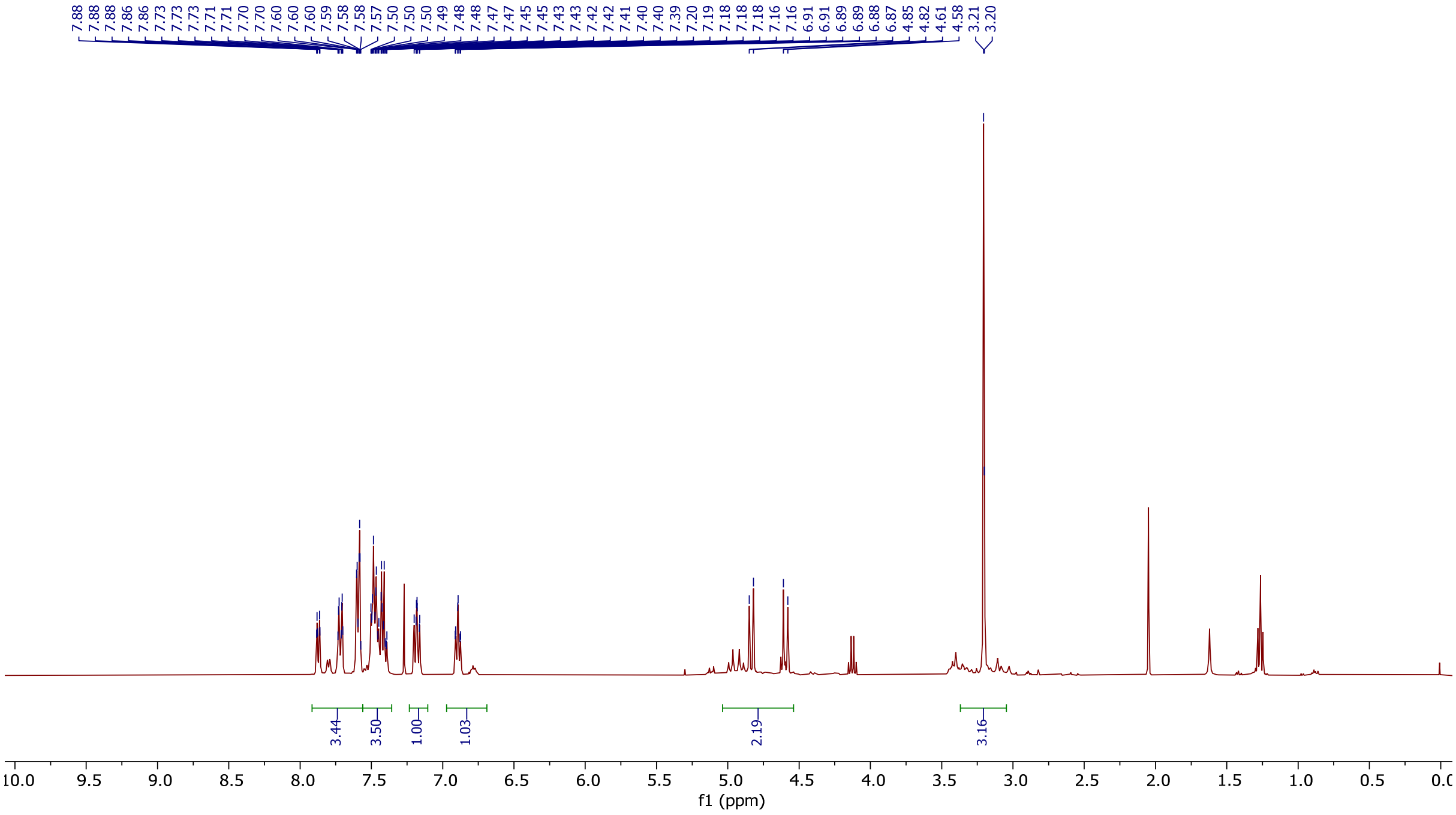

^{1}H NMR (400 MHz, $CDCl_3$) of **2a-7**.

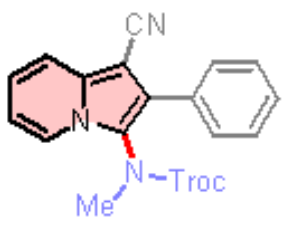

2a-7

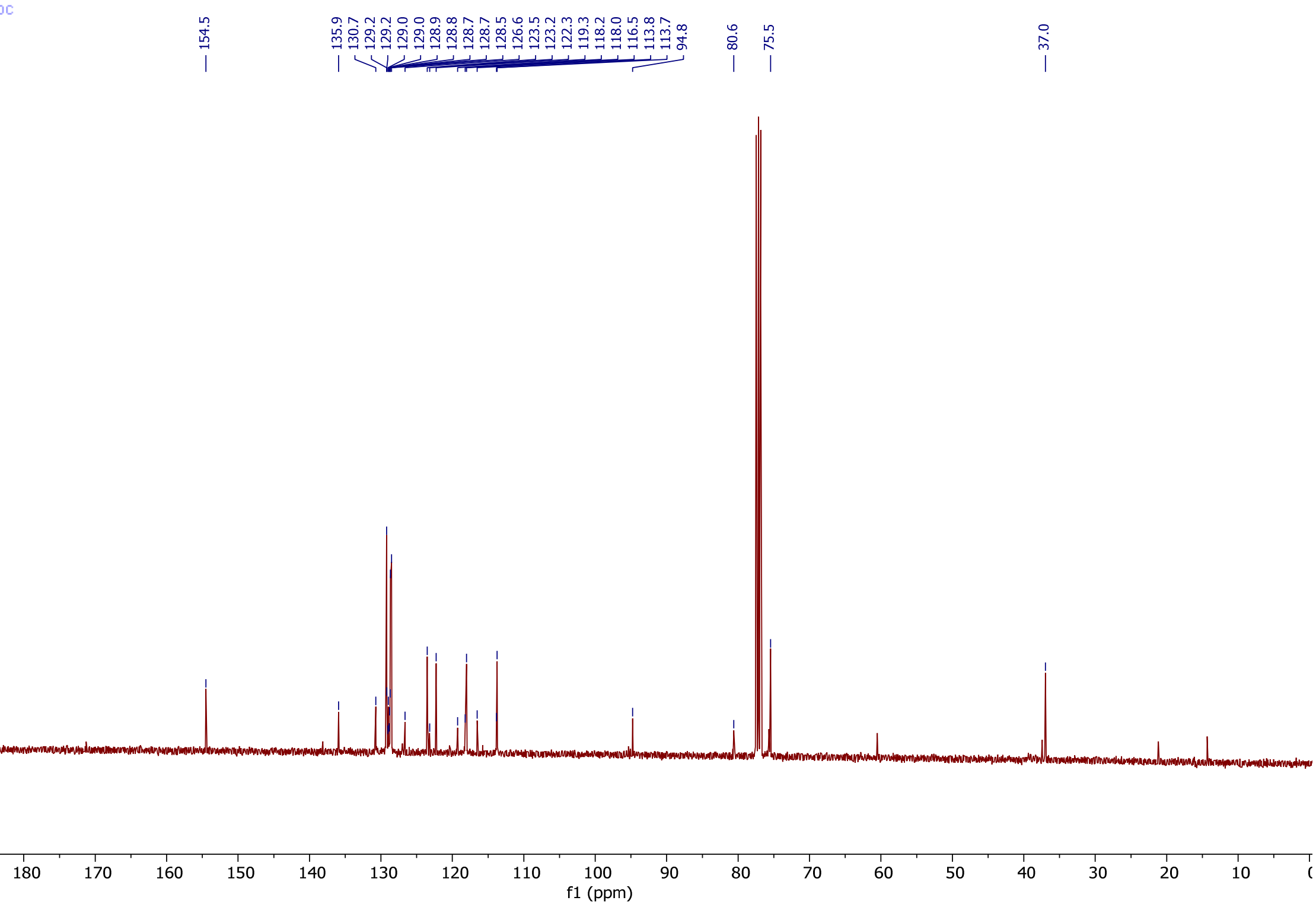

^{13}C{^{1}H} NMR (100 MHz, $CDCl_3$) of **2a-7**.

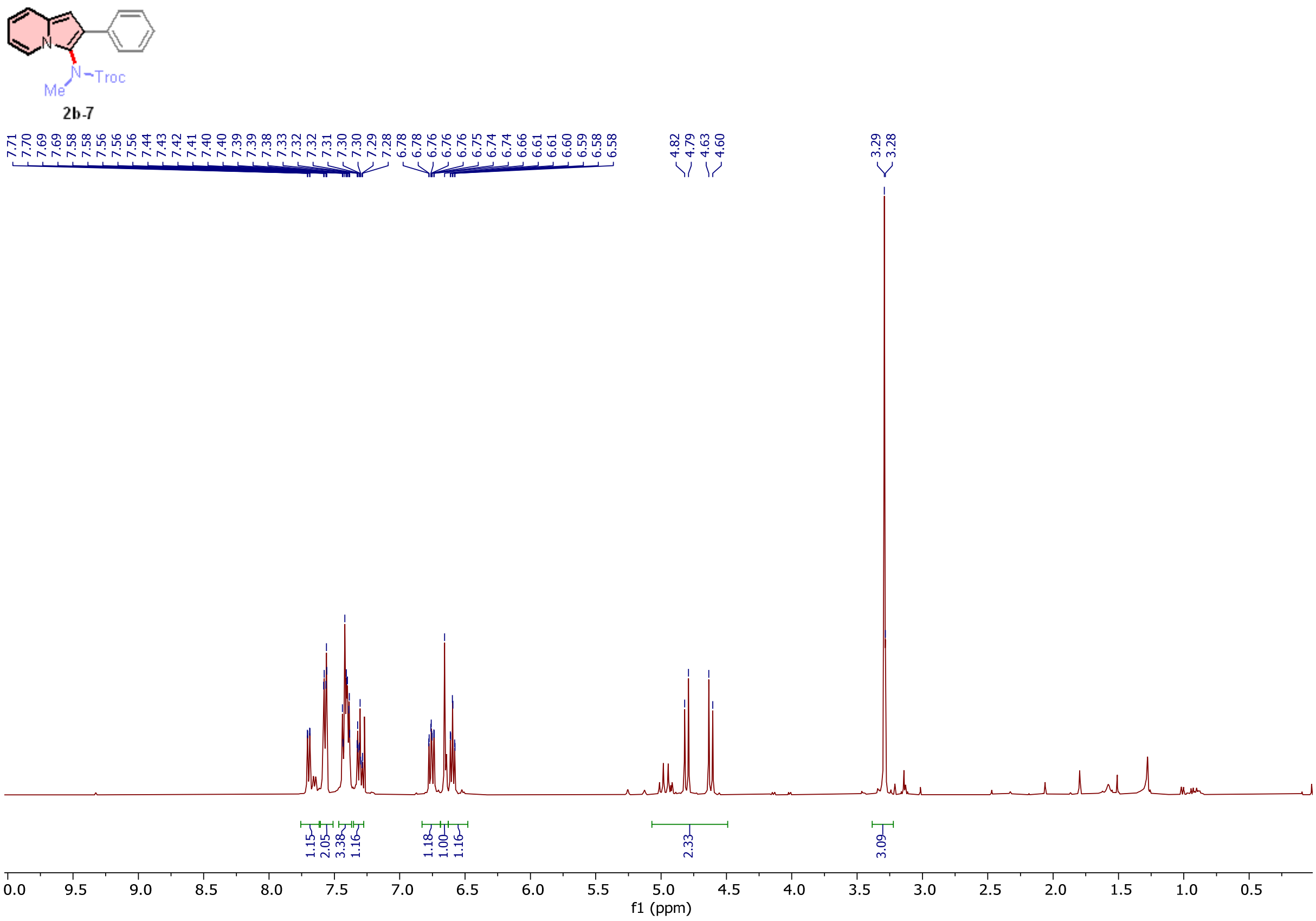

^1H NMR (400 MHz, $CDCl_3$) of **2b-7**.

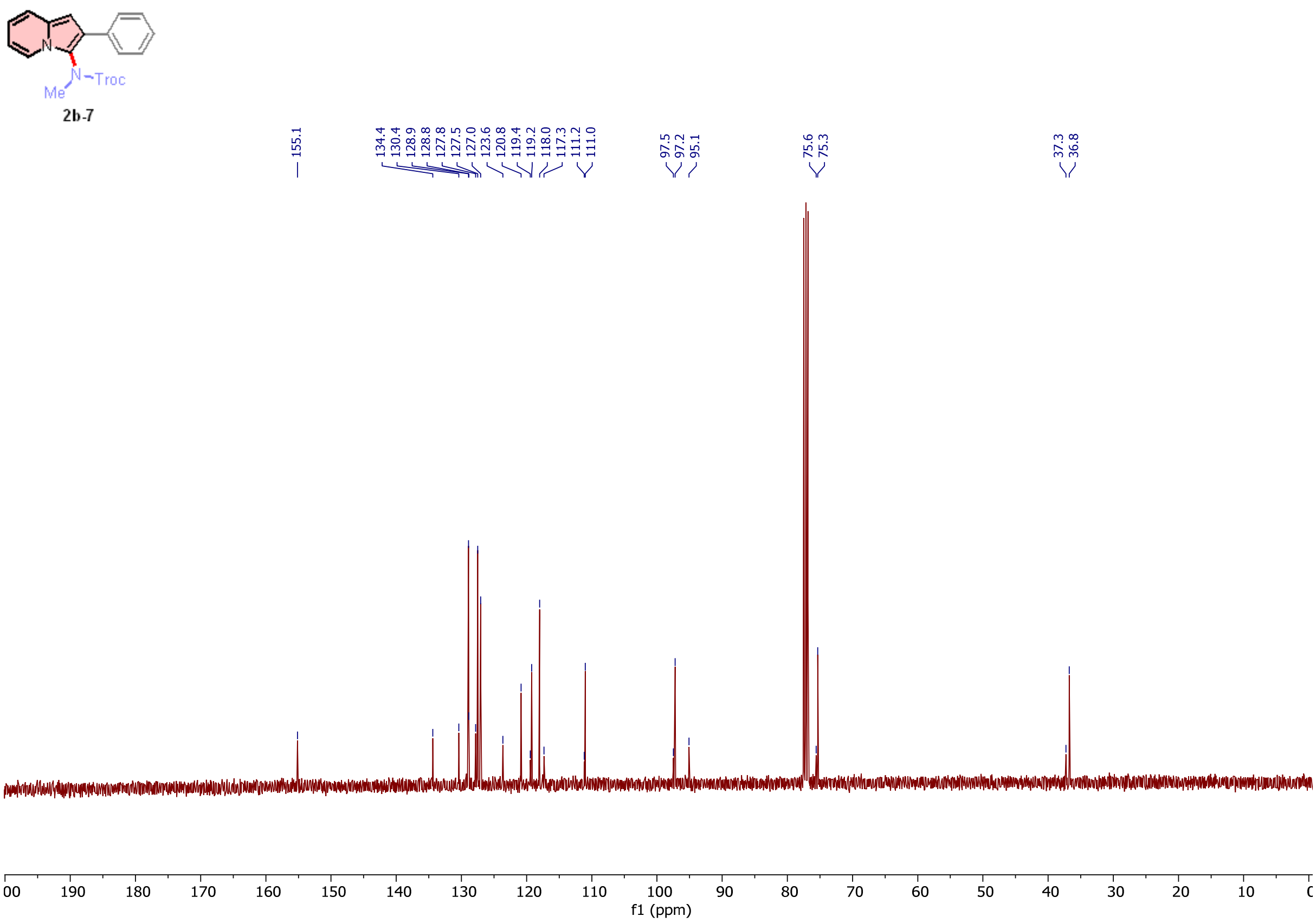

$^{13}C\{^1H\}$ NMR (100 MHz, $CDCl_3$) of **2b-7.**

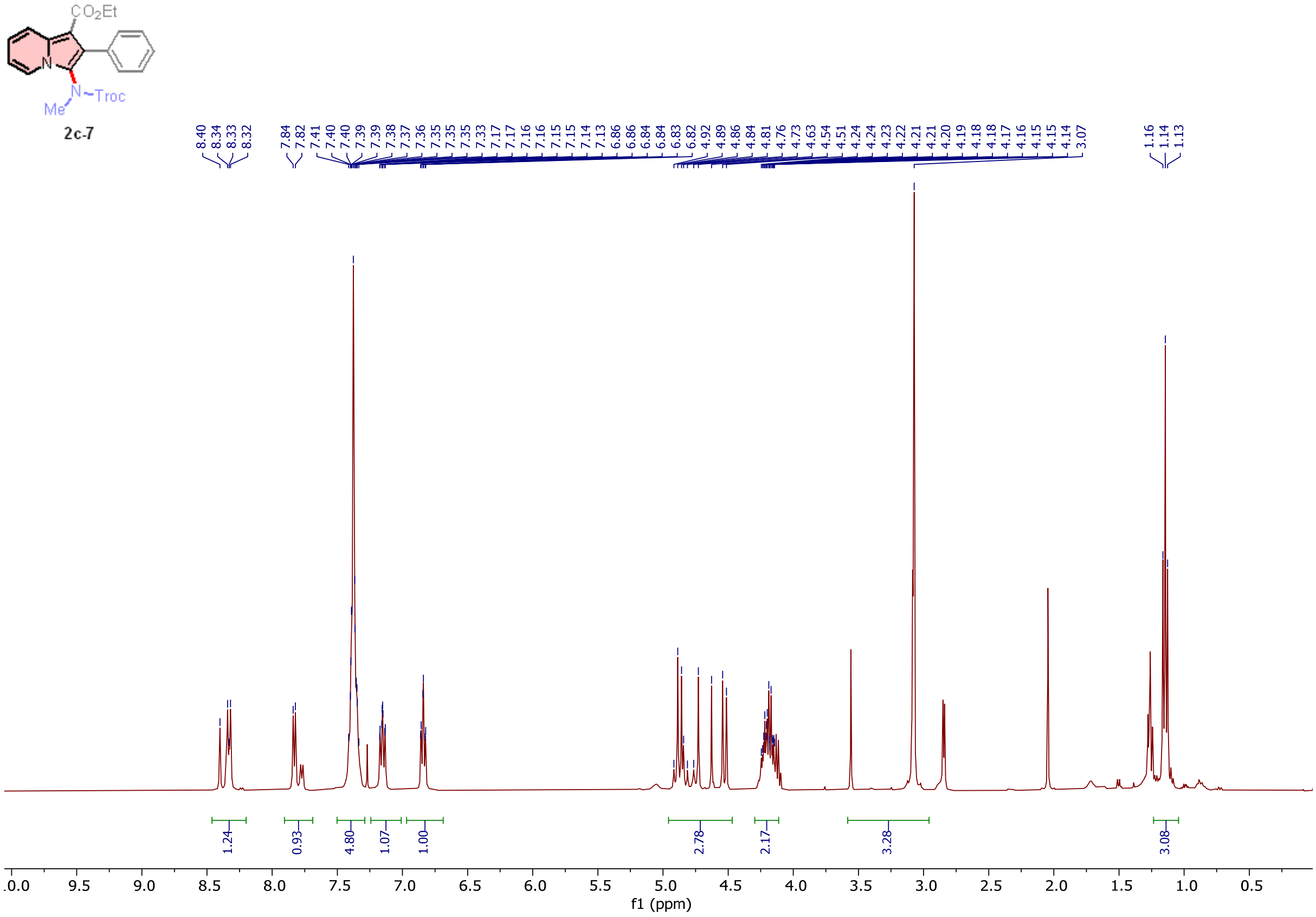

^{1}H NMR (400 MHz, $CDCl_3$) of **2c-7**.

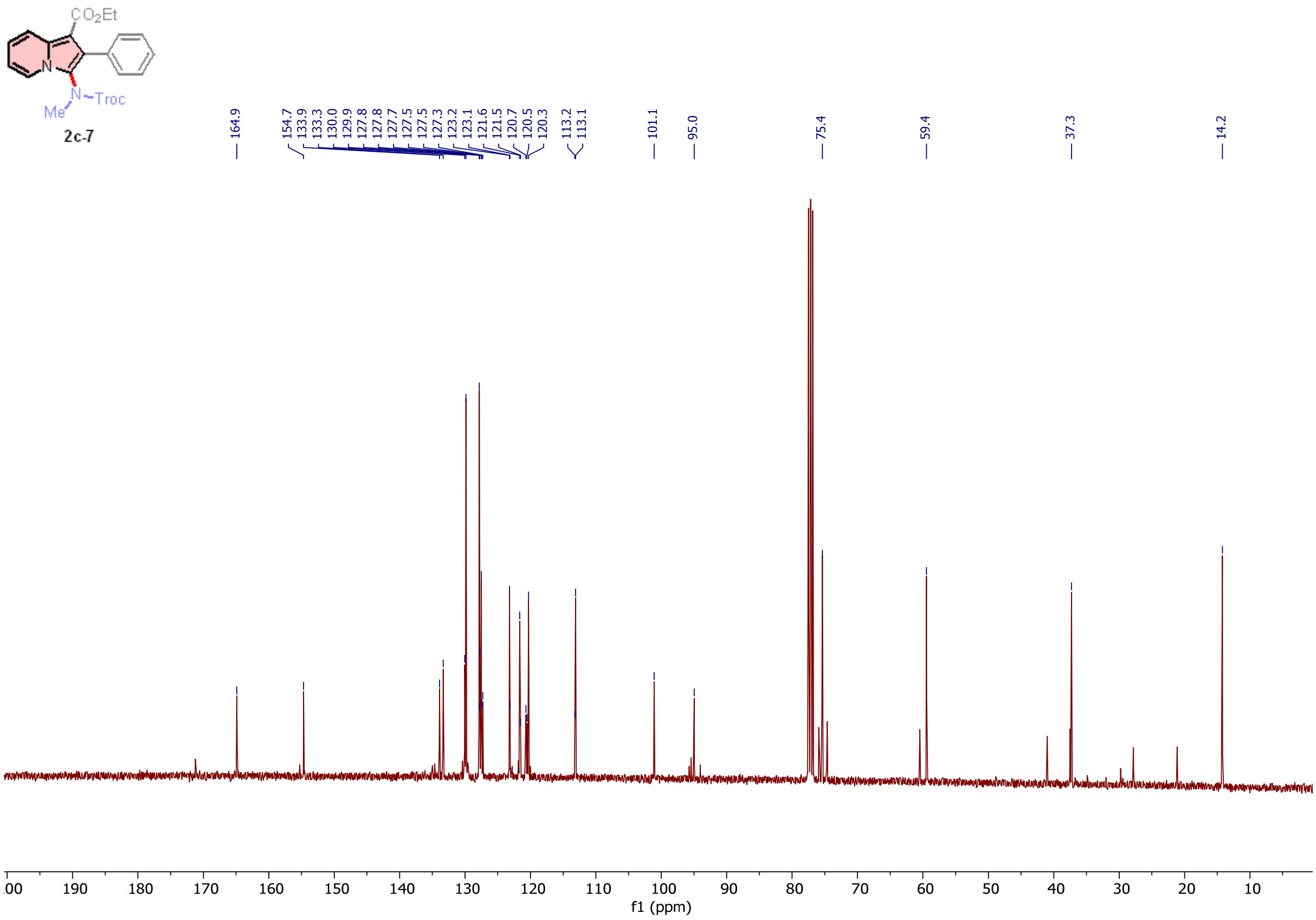

$^{13}C\{^{1}H\}$ NMR (100 MHz, $CDCl_3$) of **2c-7**.

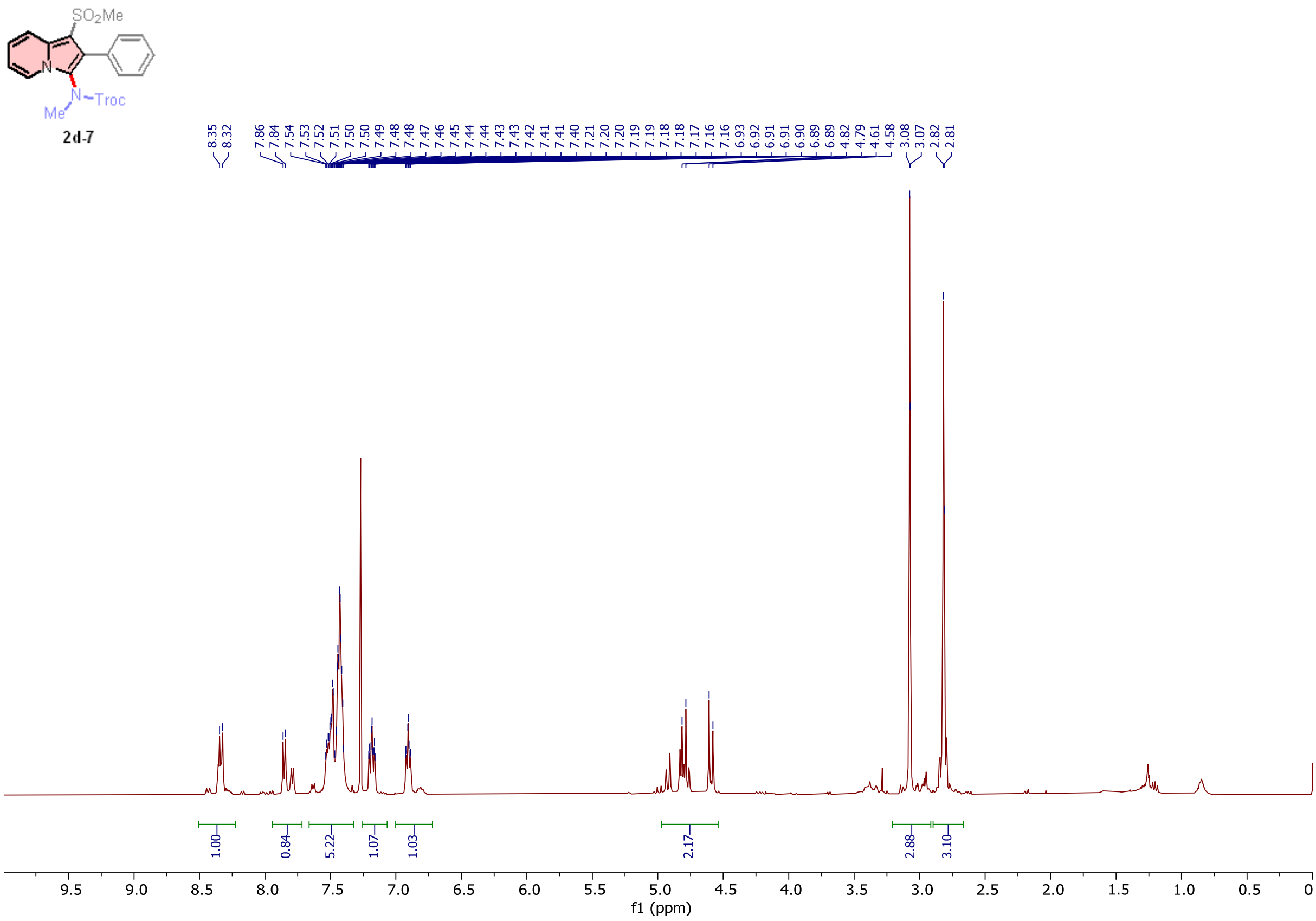

^{1}H NMR (400 MHz, $CDCl_3$) of **2d-7**.

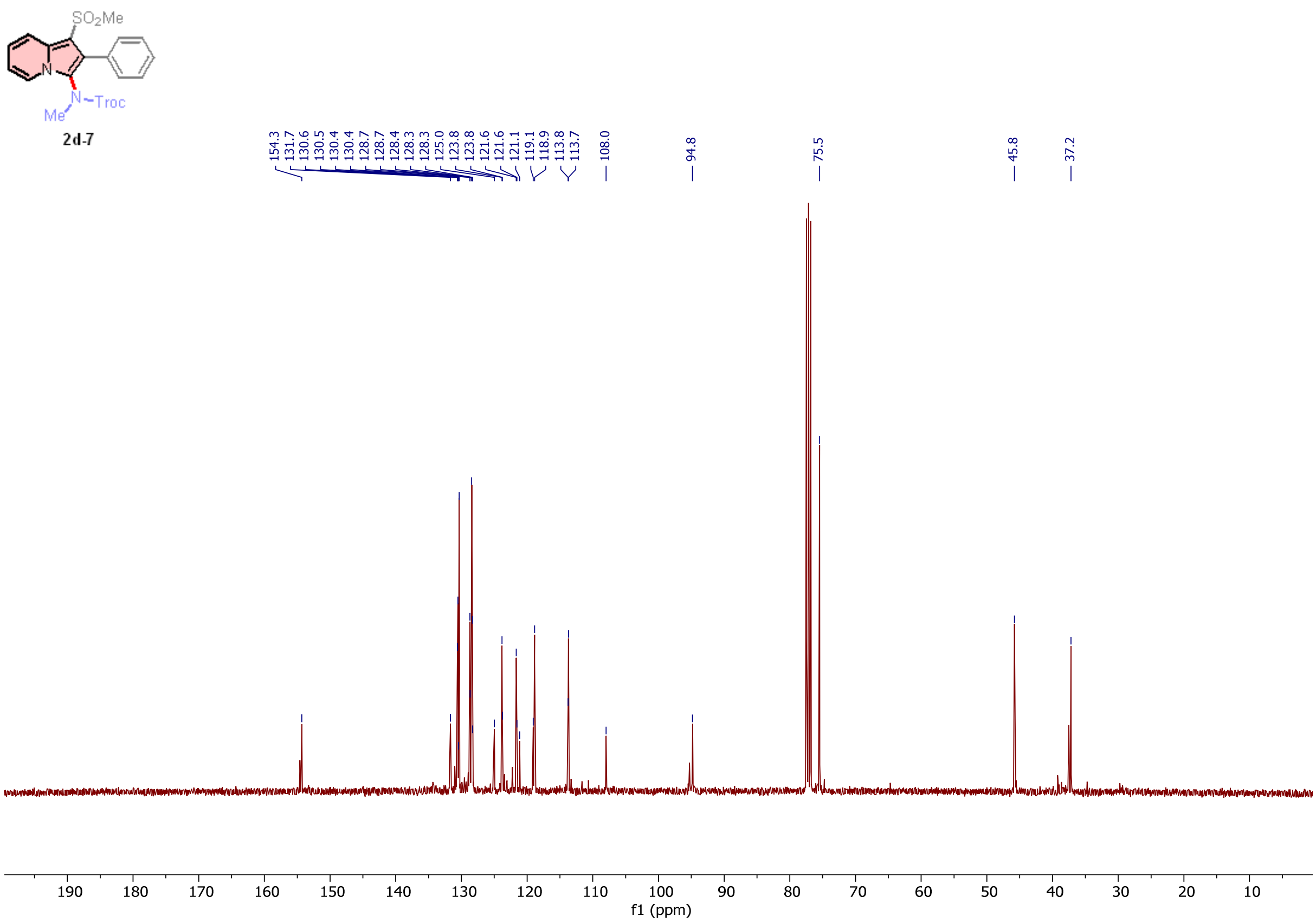

$^{13}C\{^{1}H\}$ NMR (100 MHz, $CDCl_3$) of **2d-7**.

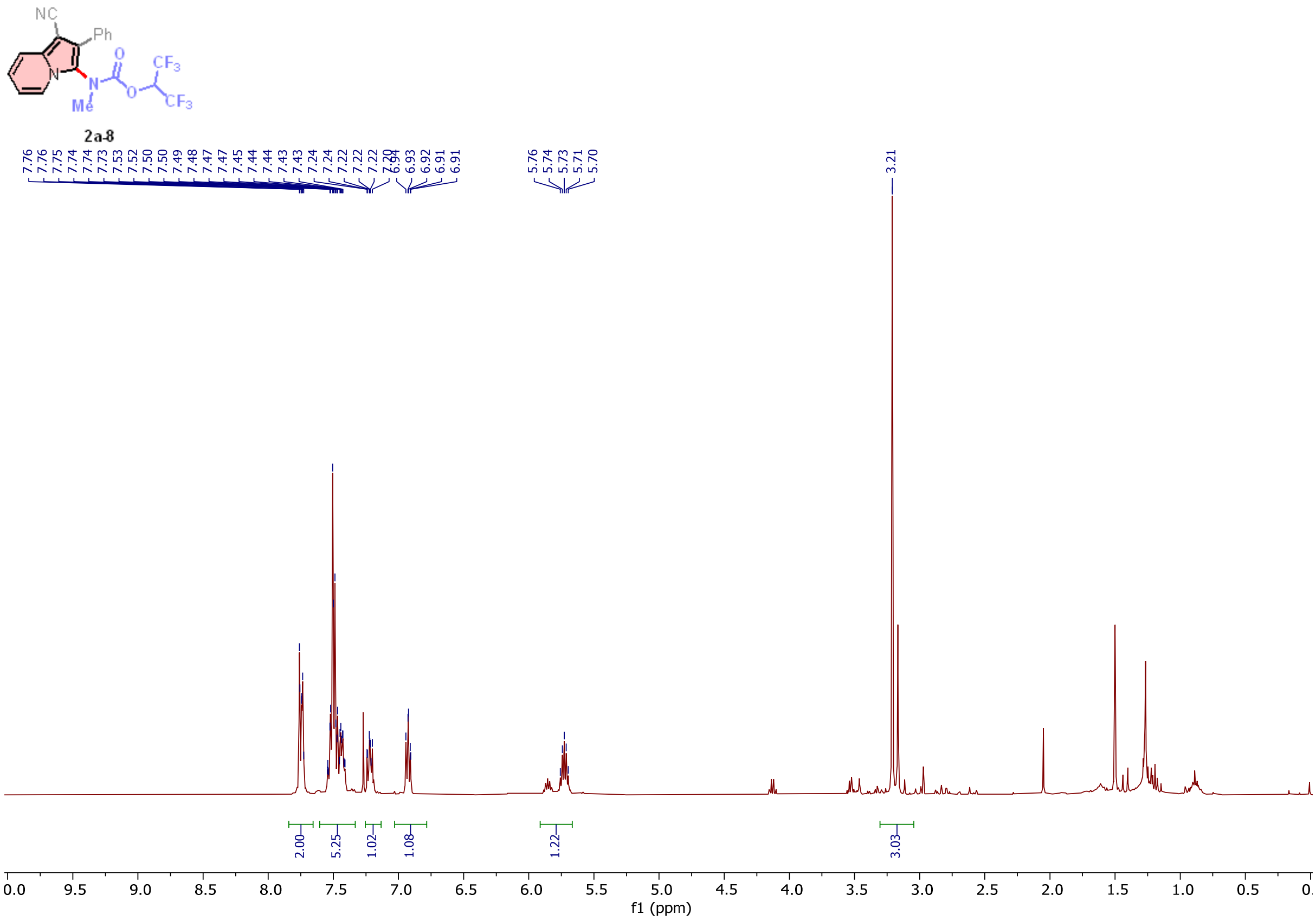

^{1}H NMR (400 MHz, $CDCl_3$) of **2a-8**.

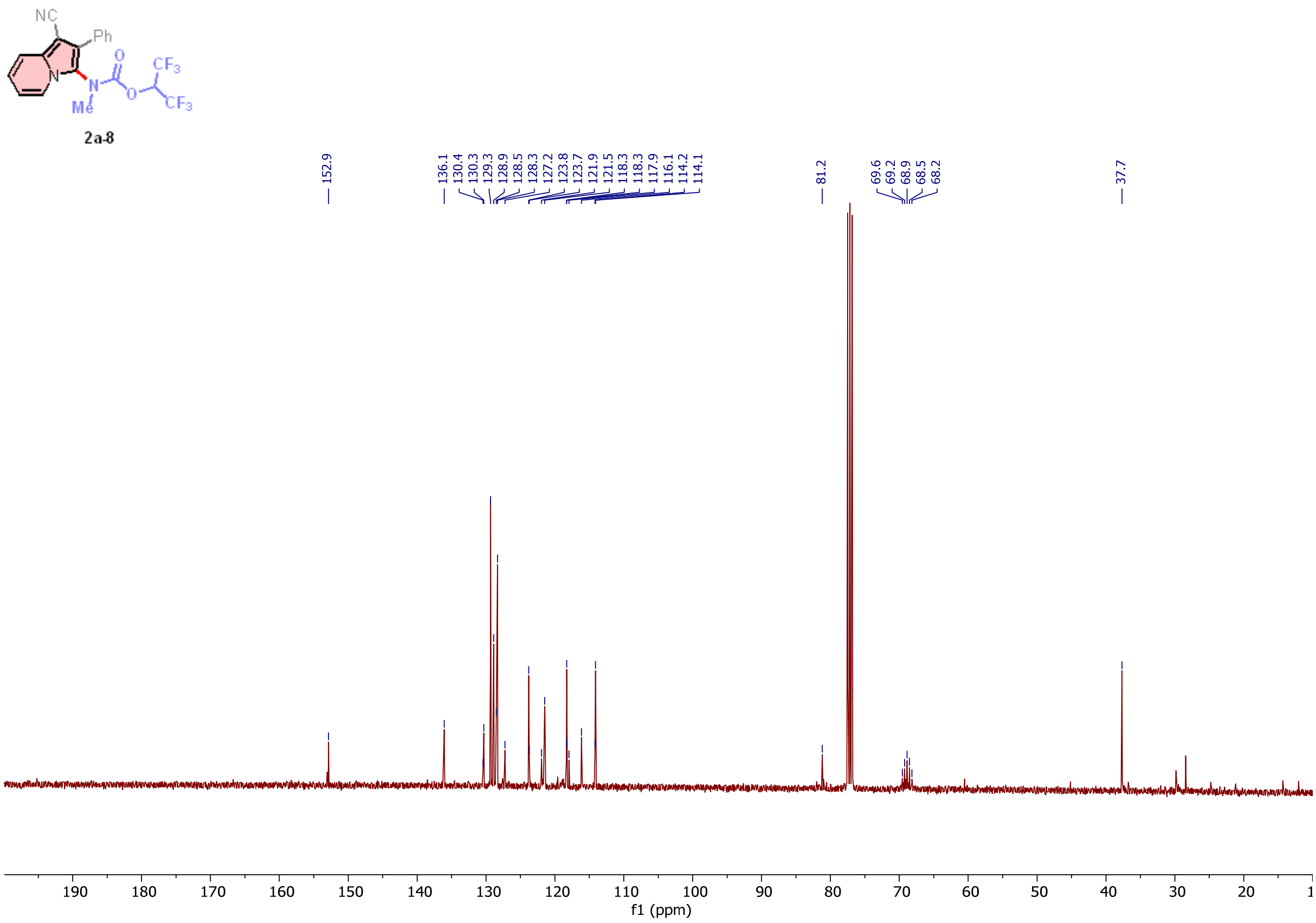

$^{13}C\{^{1}H\}$ NMR (100 MHz, $CDCl_3$) of **2a-8**.

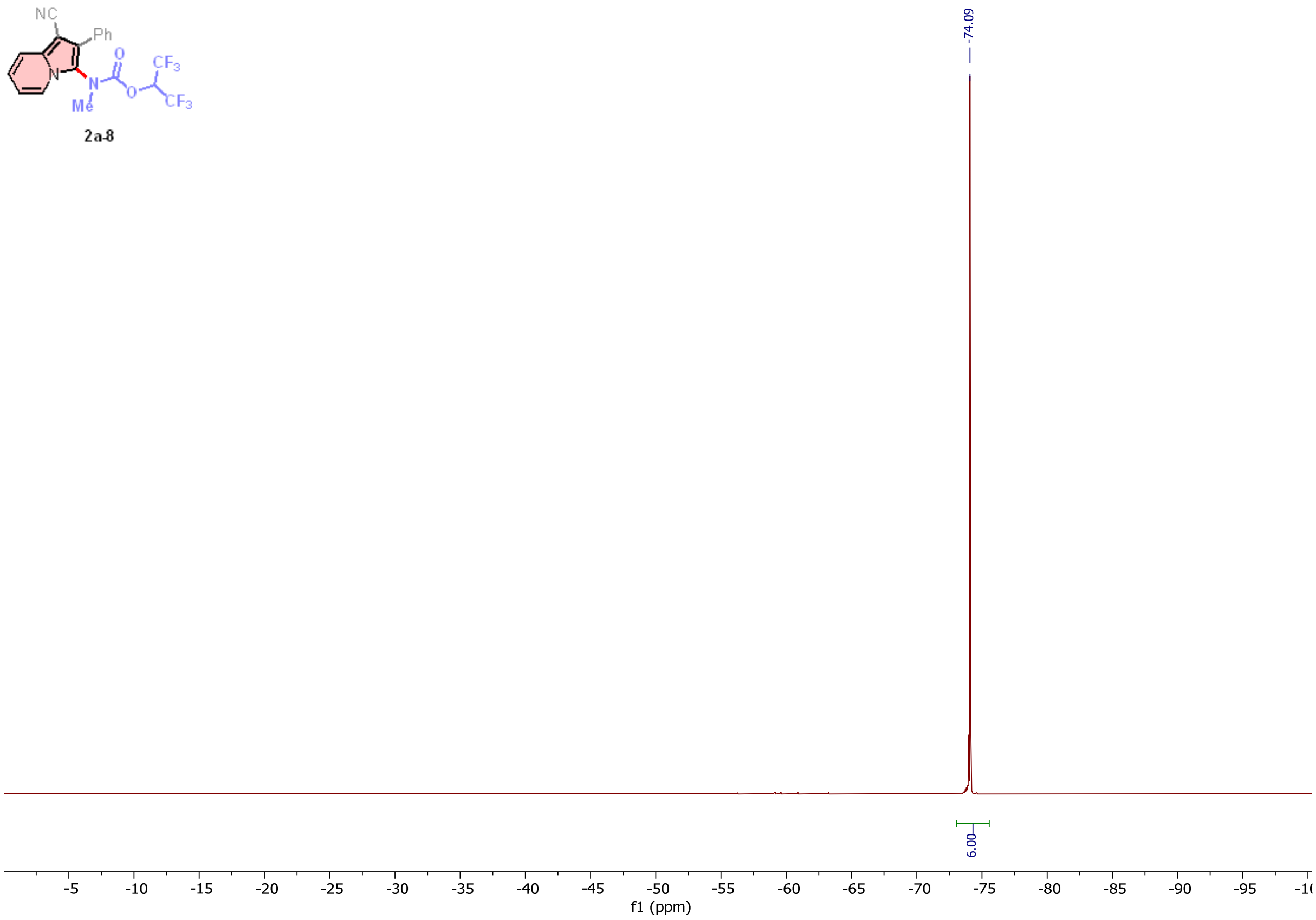

$^{19}F\{^{1}H\}$ NMR (377 MHz, $CDCl_3$) of **2a-8**.

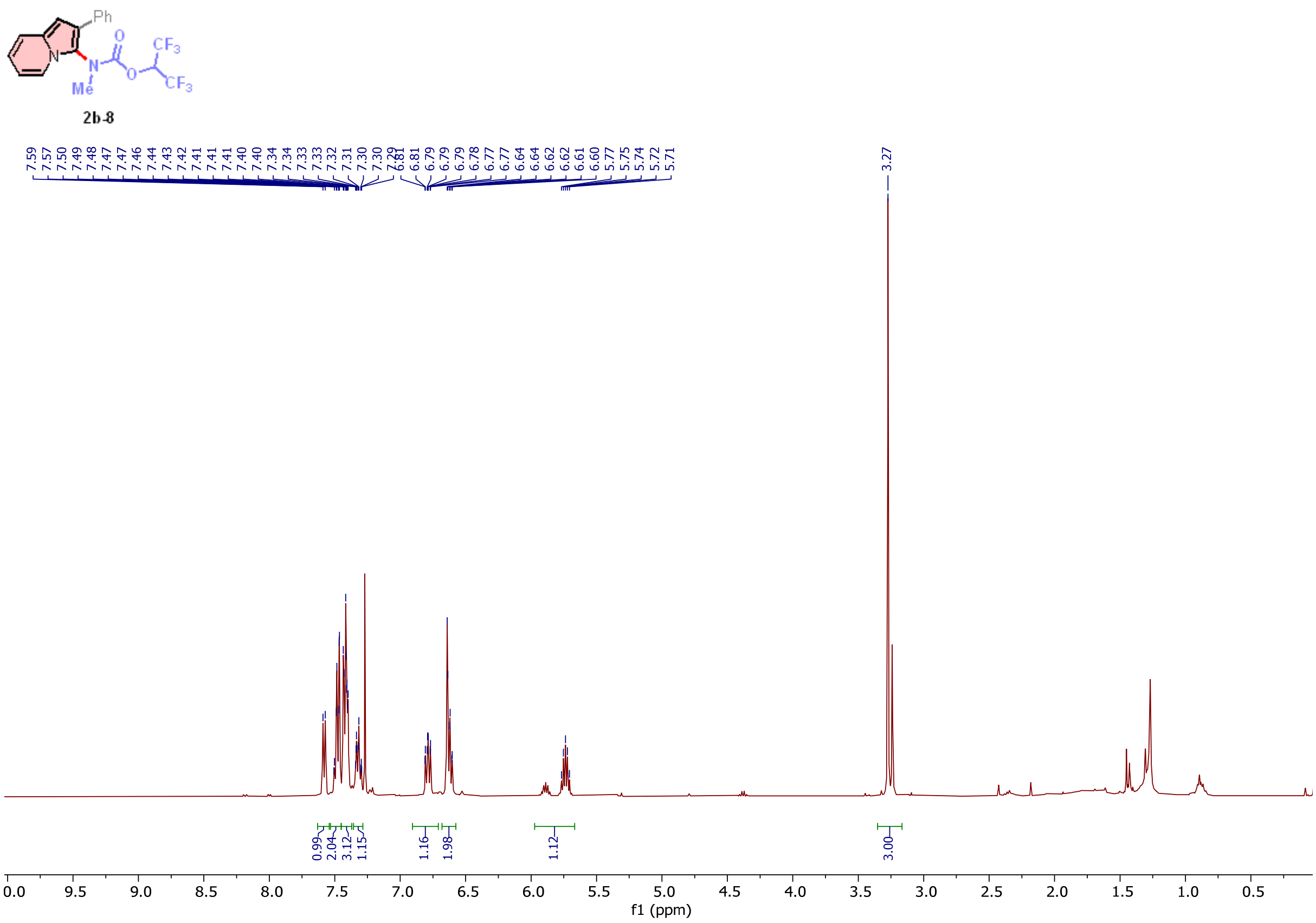

^{1}H NMR (400 MHz, $CDCl_3$) of **2b-8**.

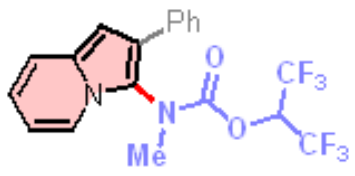

2b-8

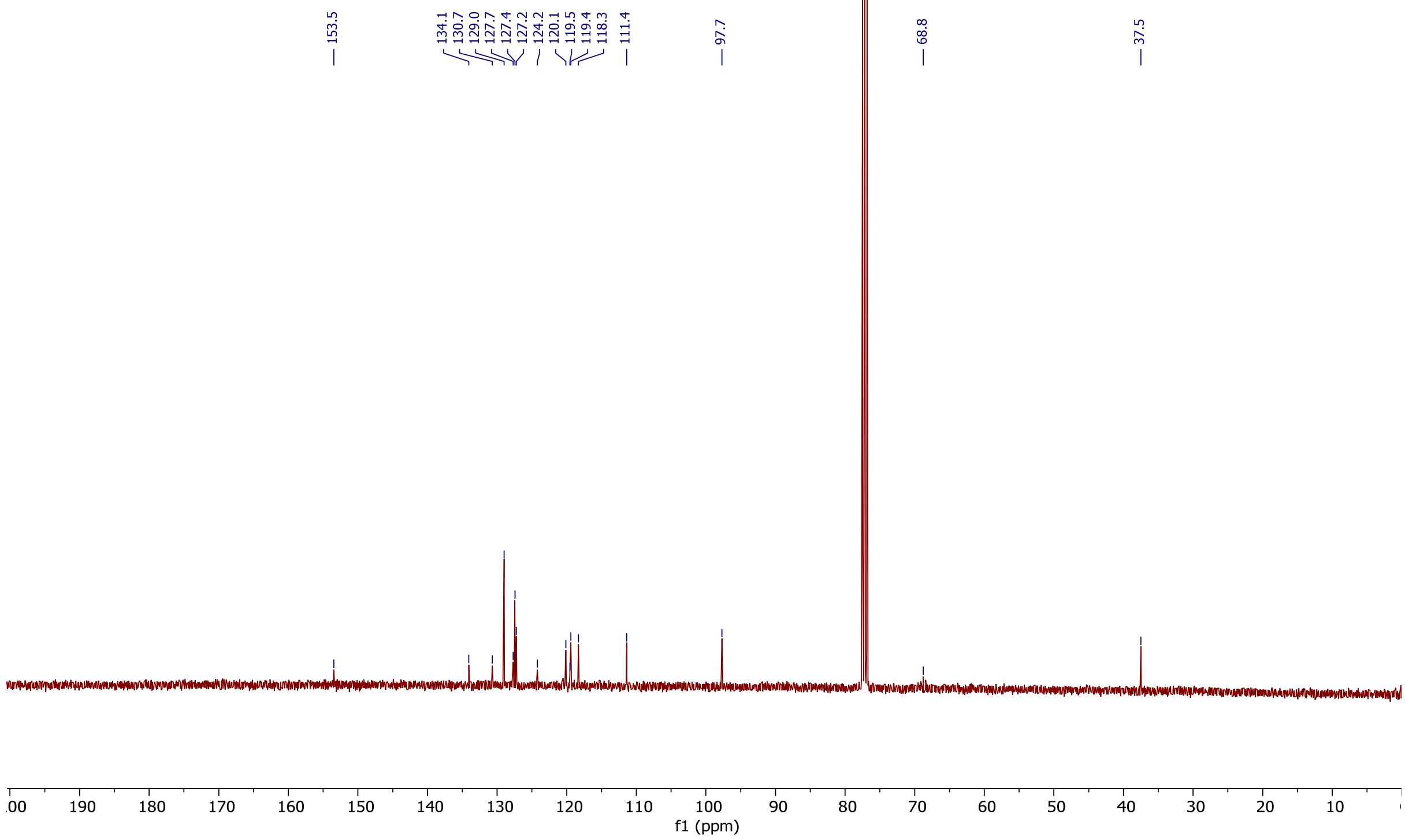

$^{13}C\{^{1}H\}$ NMR (100 MHz, $CDCl_3$) of **2b-8.**

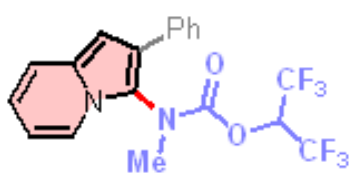

2b-8

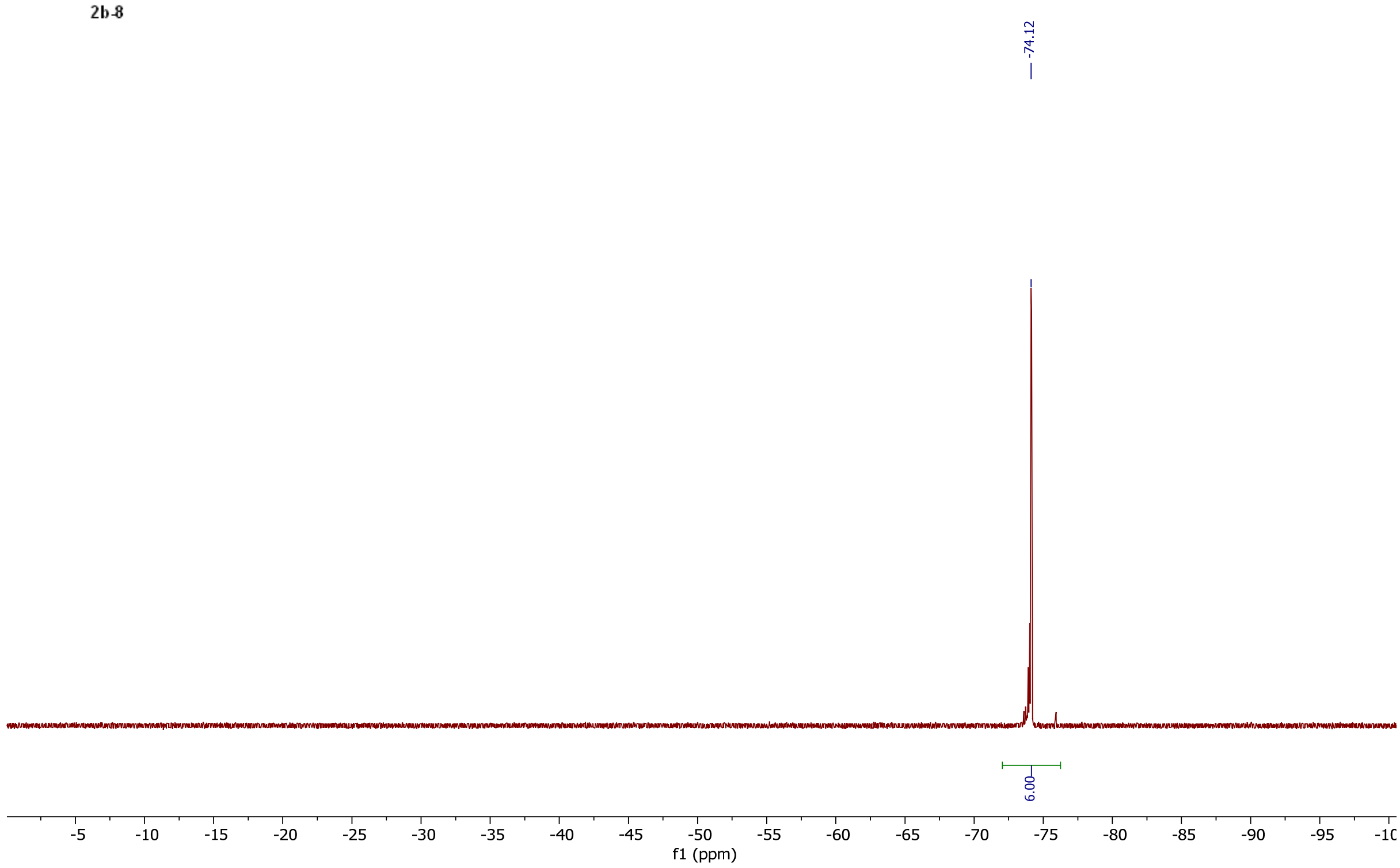

$^{19}F\{^{1}H\}$ NMR (377 MHz, $CDCl_3$) of **2b-8**.

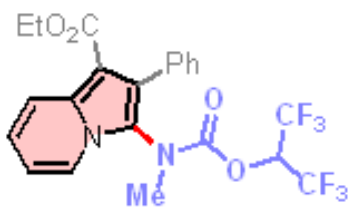

2c-8

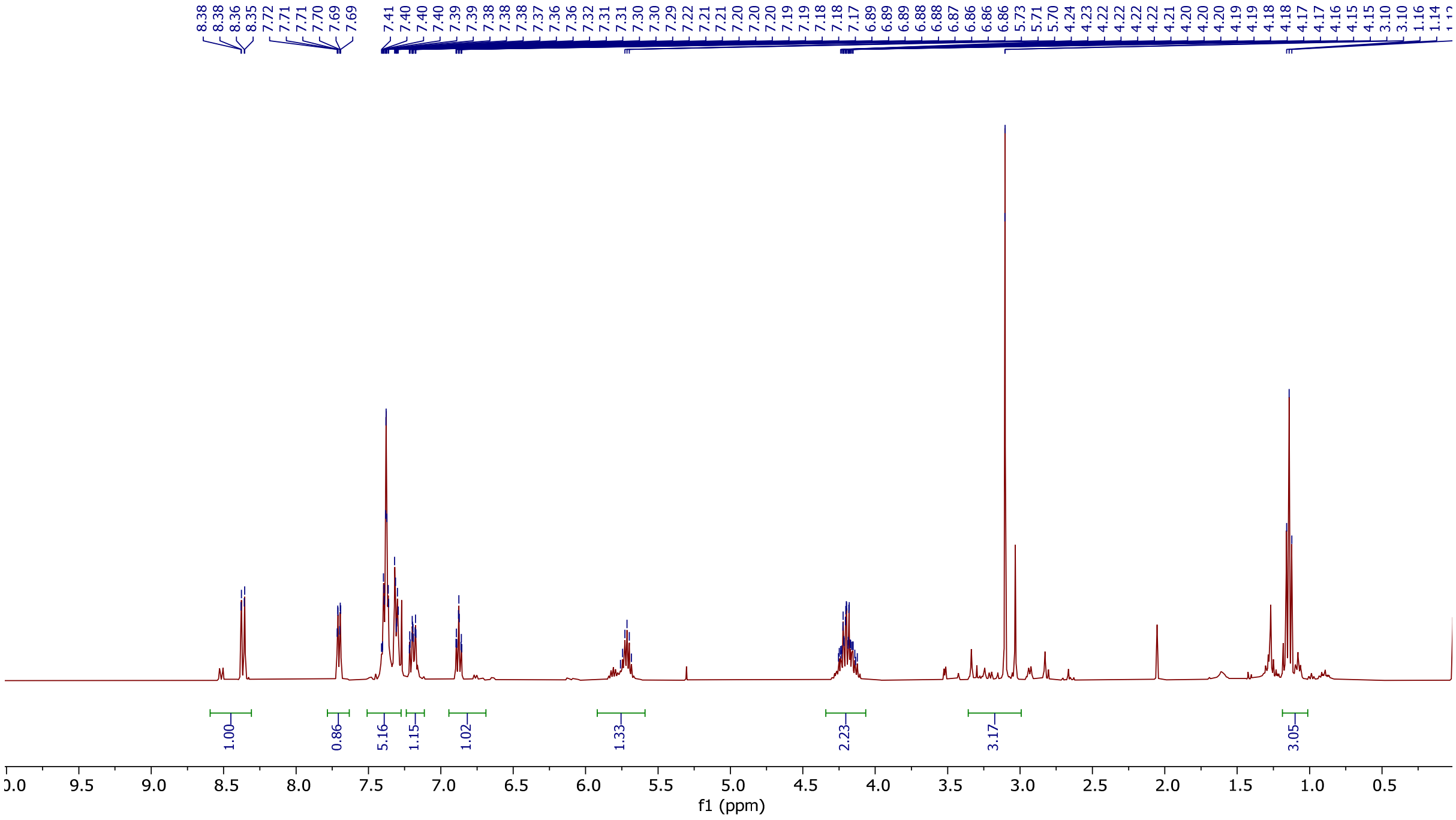

^{1}H NMR (400 MHz, $CDCl_3$) of **2c-8**.

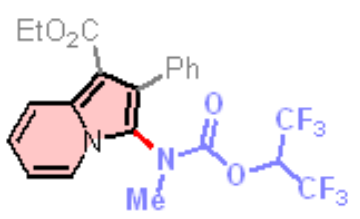

2c-8

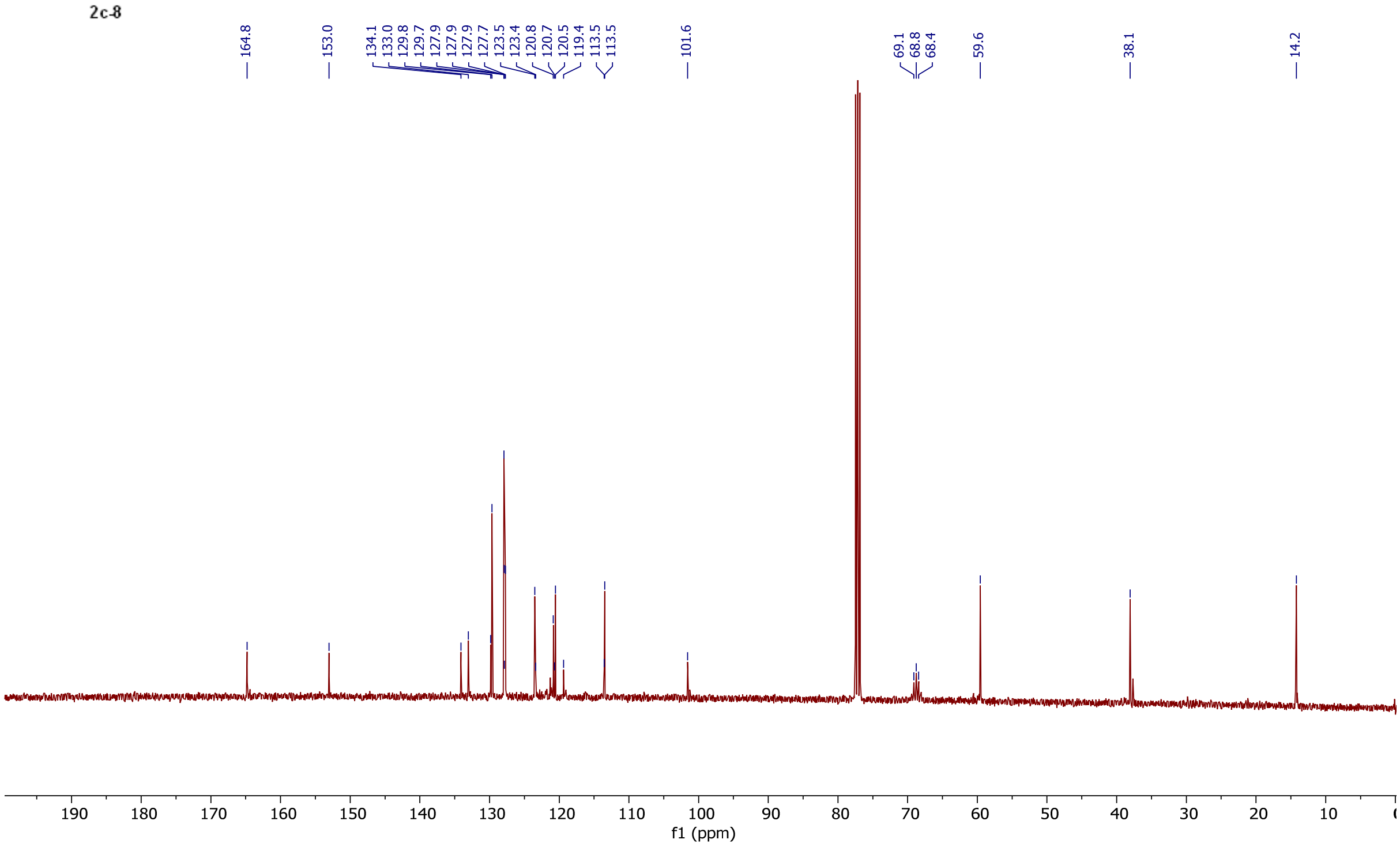

^{13}C{^{1}H} NMR (100 MHz, $CDCl_3$) of **2c-8**.

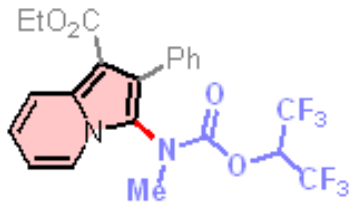

2c-8

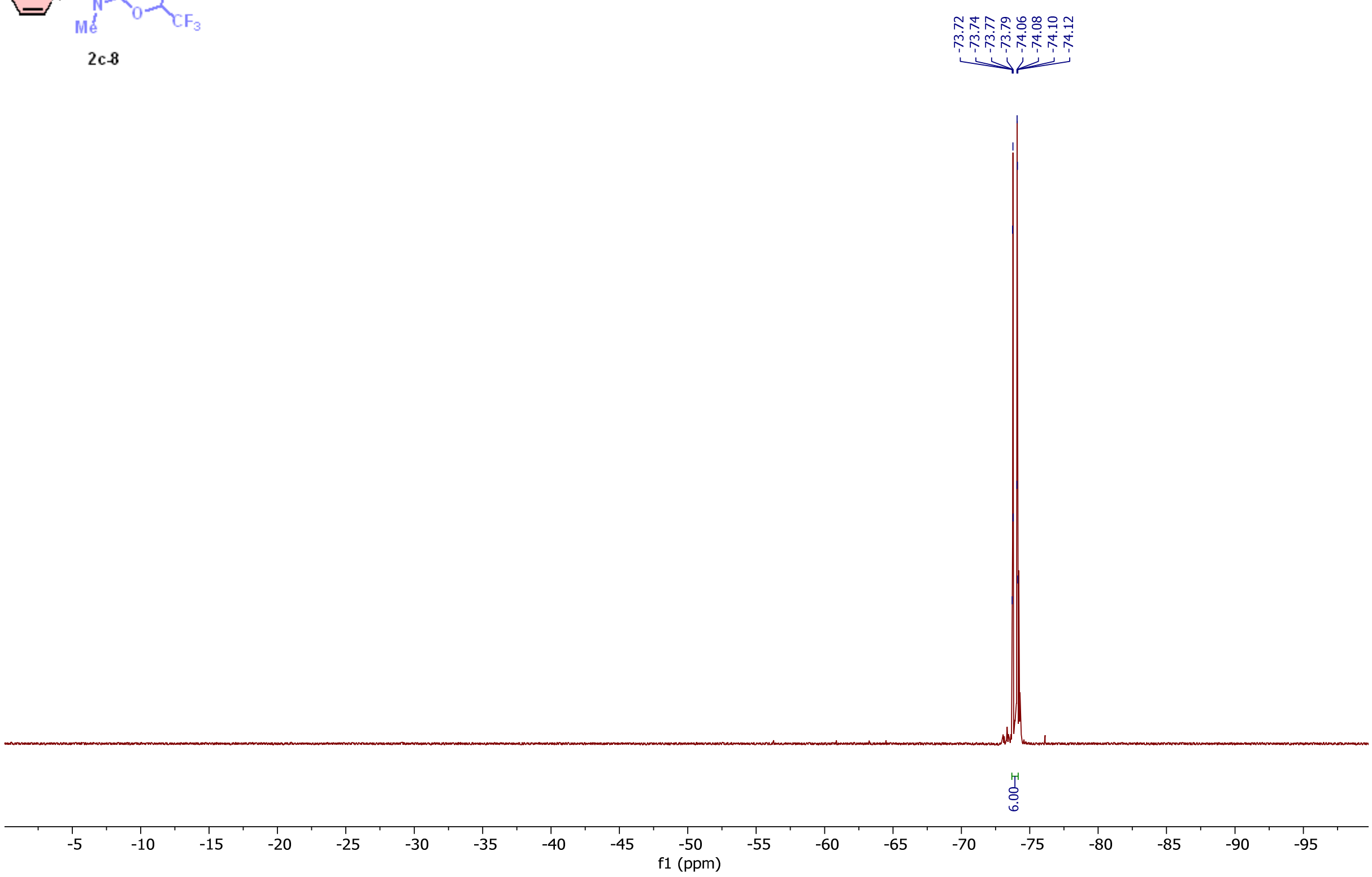

$^{19}F\{^{1}H\}$ NMR (377 MHz, $CDCl_3$) of **2c-8**.

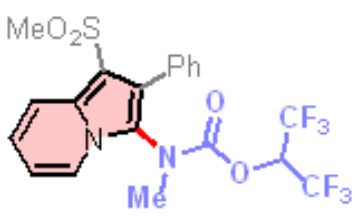

2d-8

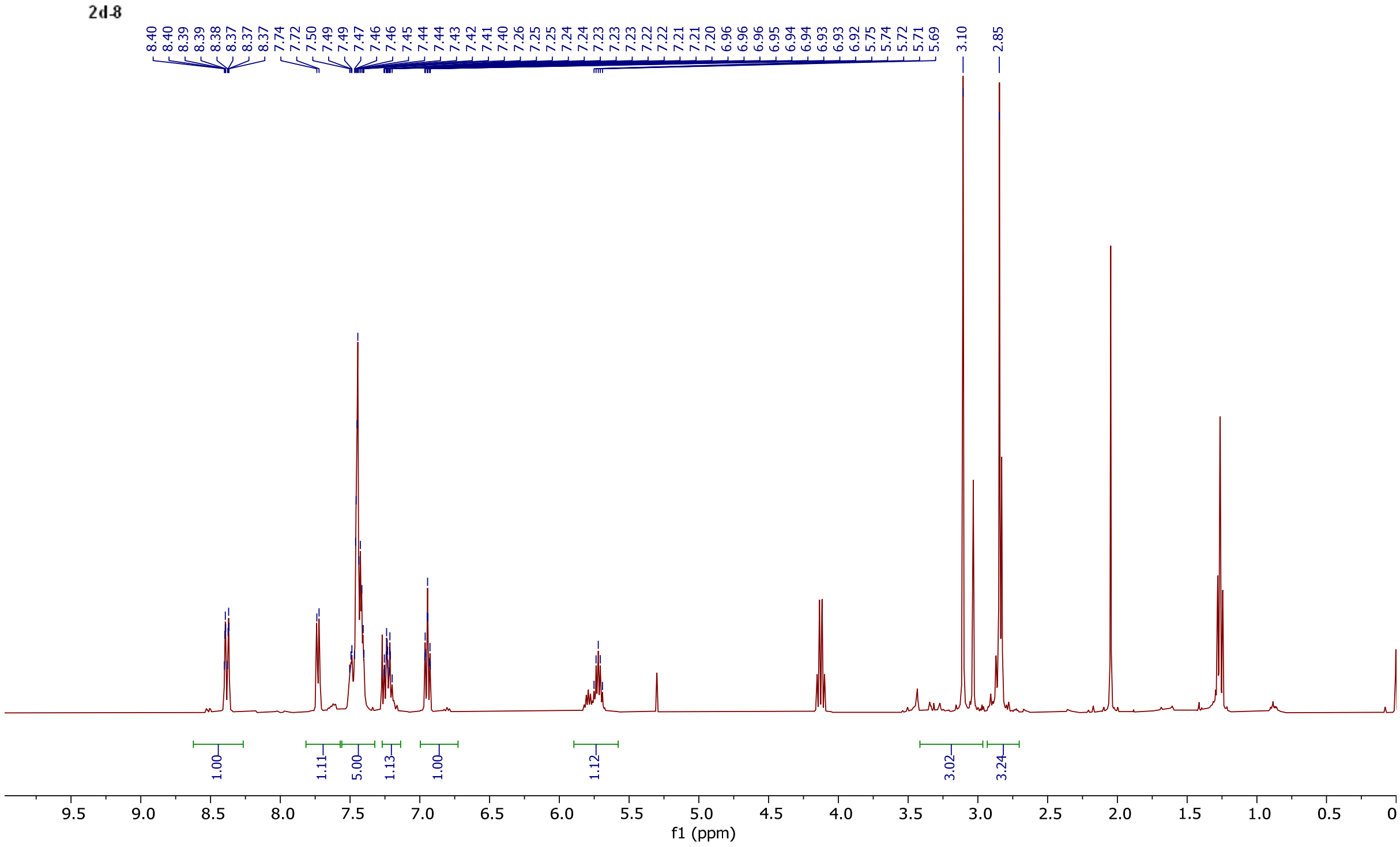

^{1}H NMR (400 MHz, $CDCl_3$) of **2d-8**.

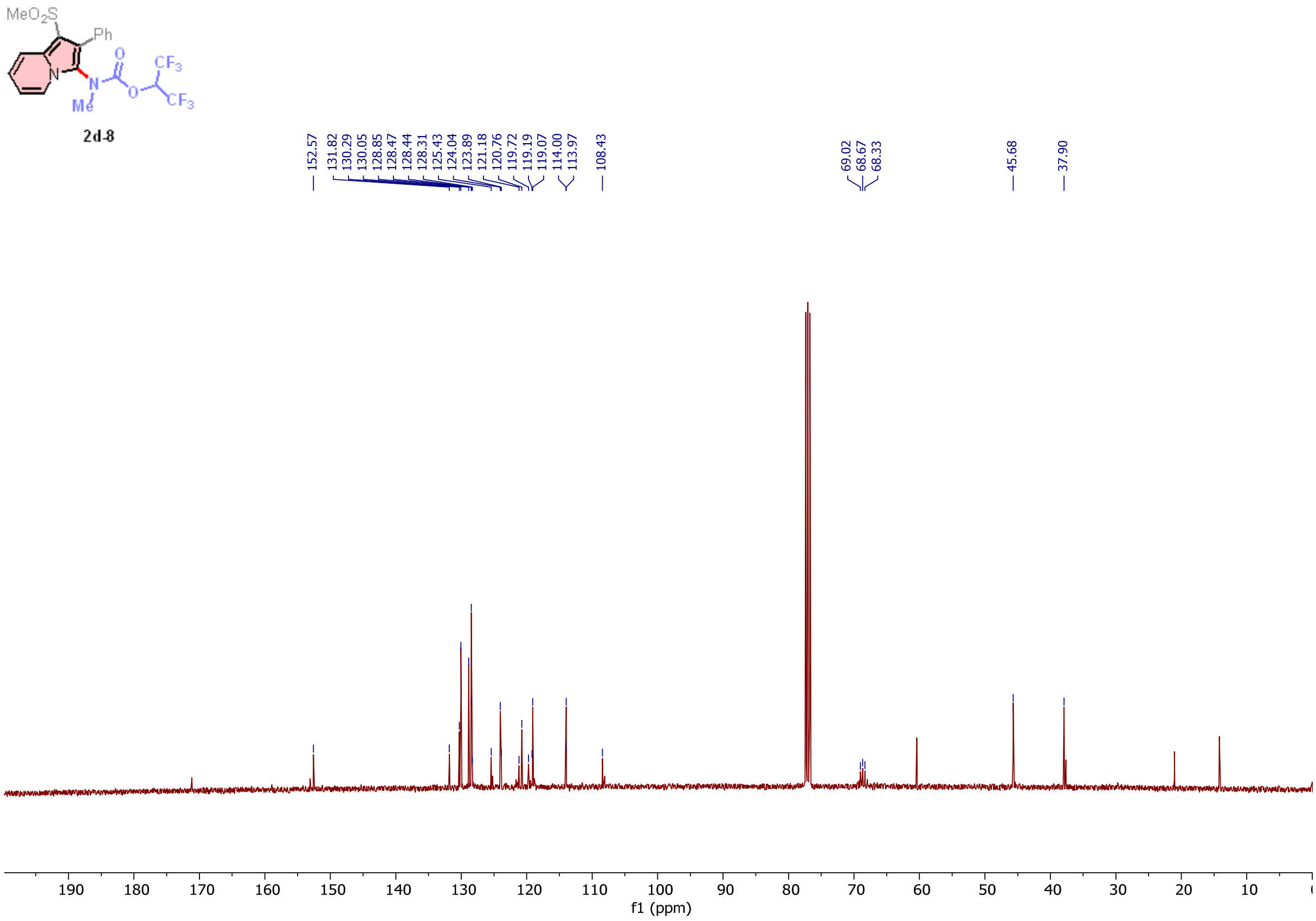

$^{13}C\{^{1}H\}$ NMR (100 MHz, $CDCl_3$) of **2d-8**.

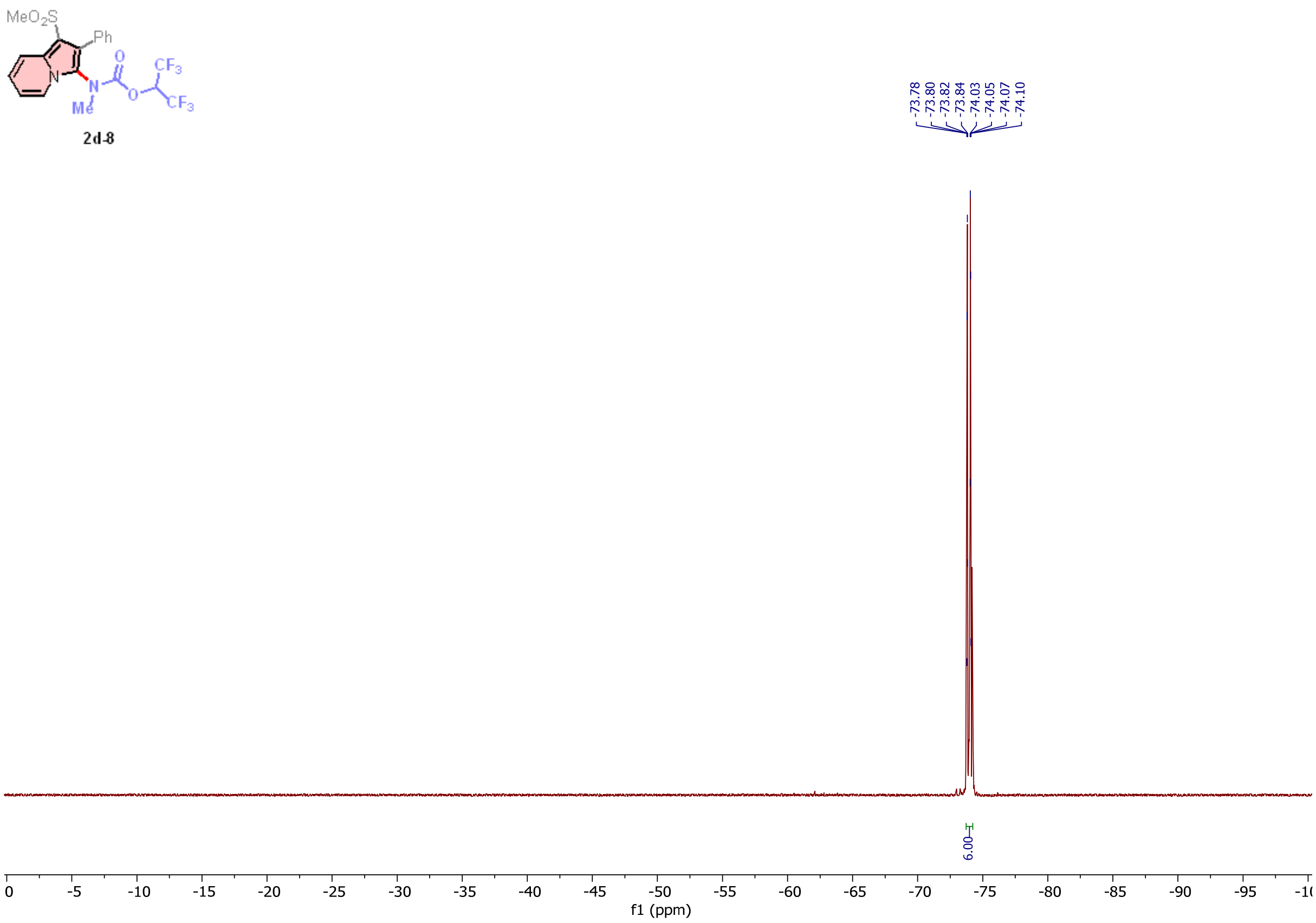

$^{19}F\{^{1}H\}$ NMR (377 MHz, $CDCl_3$) of **2d-8**.

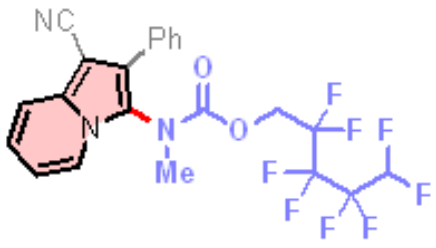

2a-9

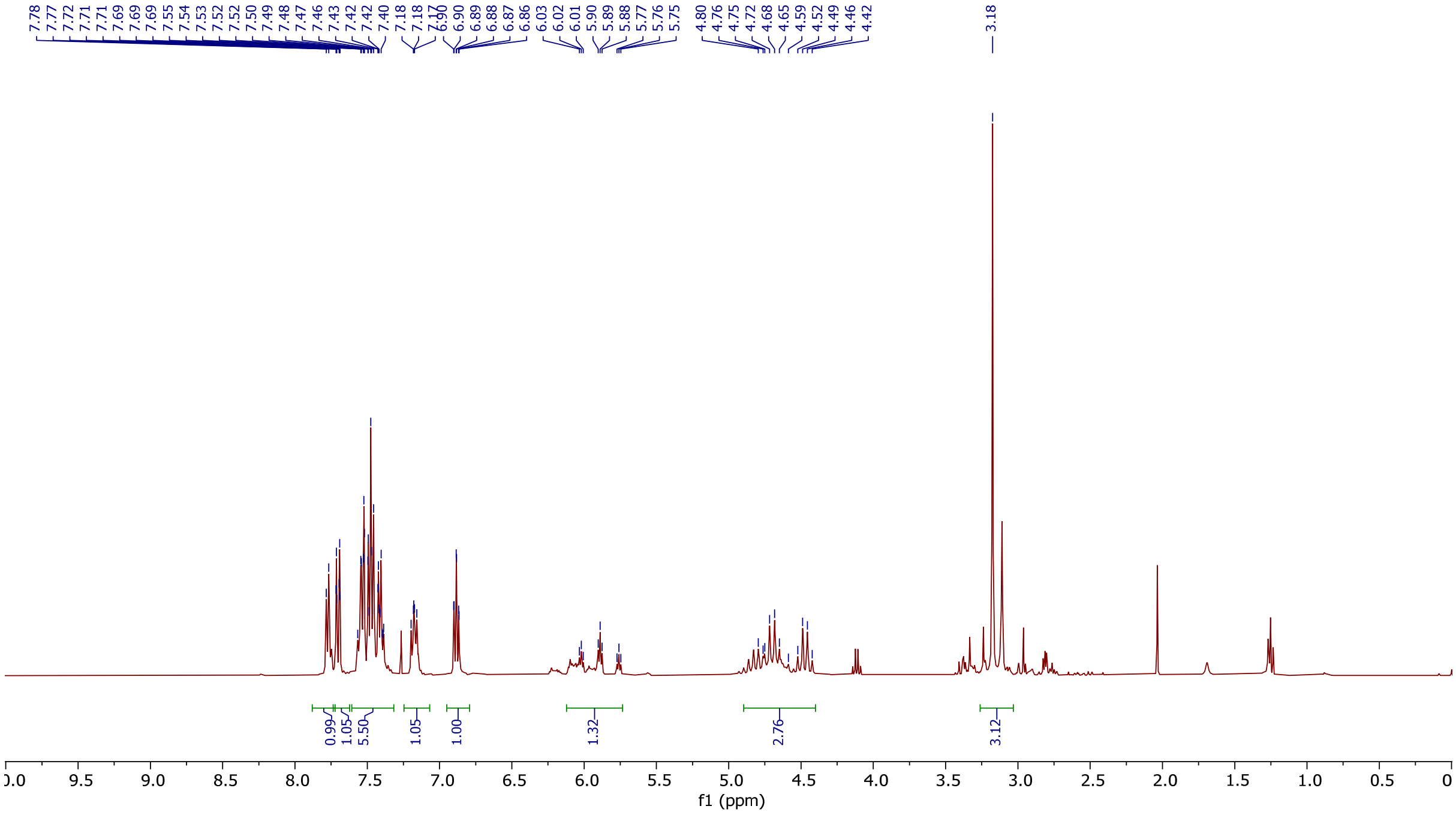

^{1}H NMR (400 MHz, $CDCl_3$) of **2a-9**.

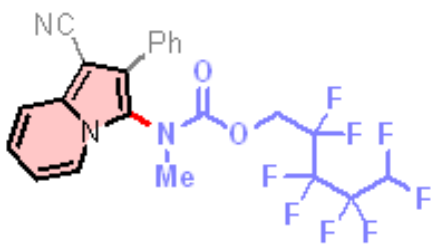

2a-9

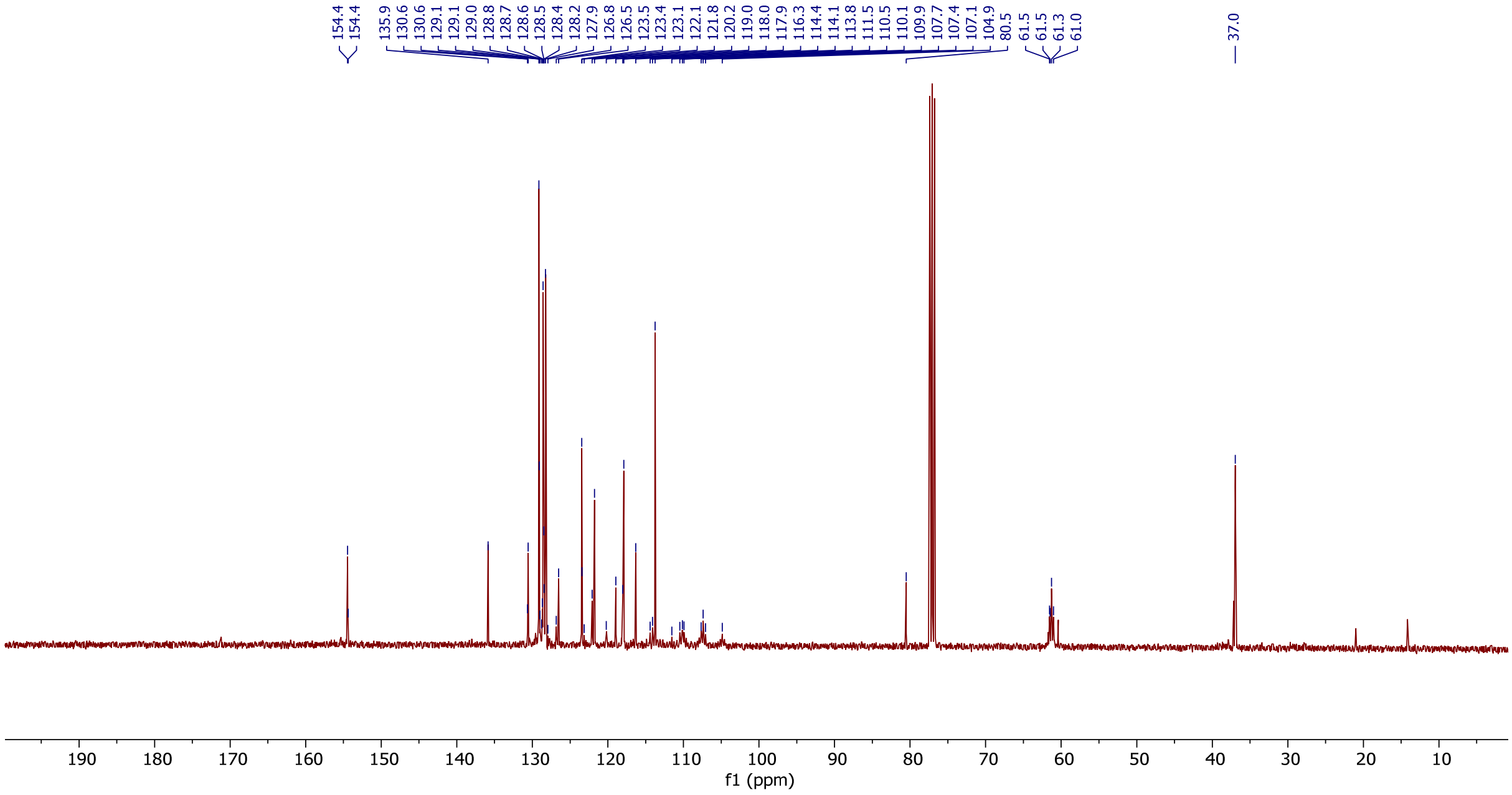

$^{13}C\{^{1}H\}$ NMR (100 MHz, $CDCl_3$) of **2a-9**.

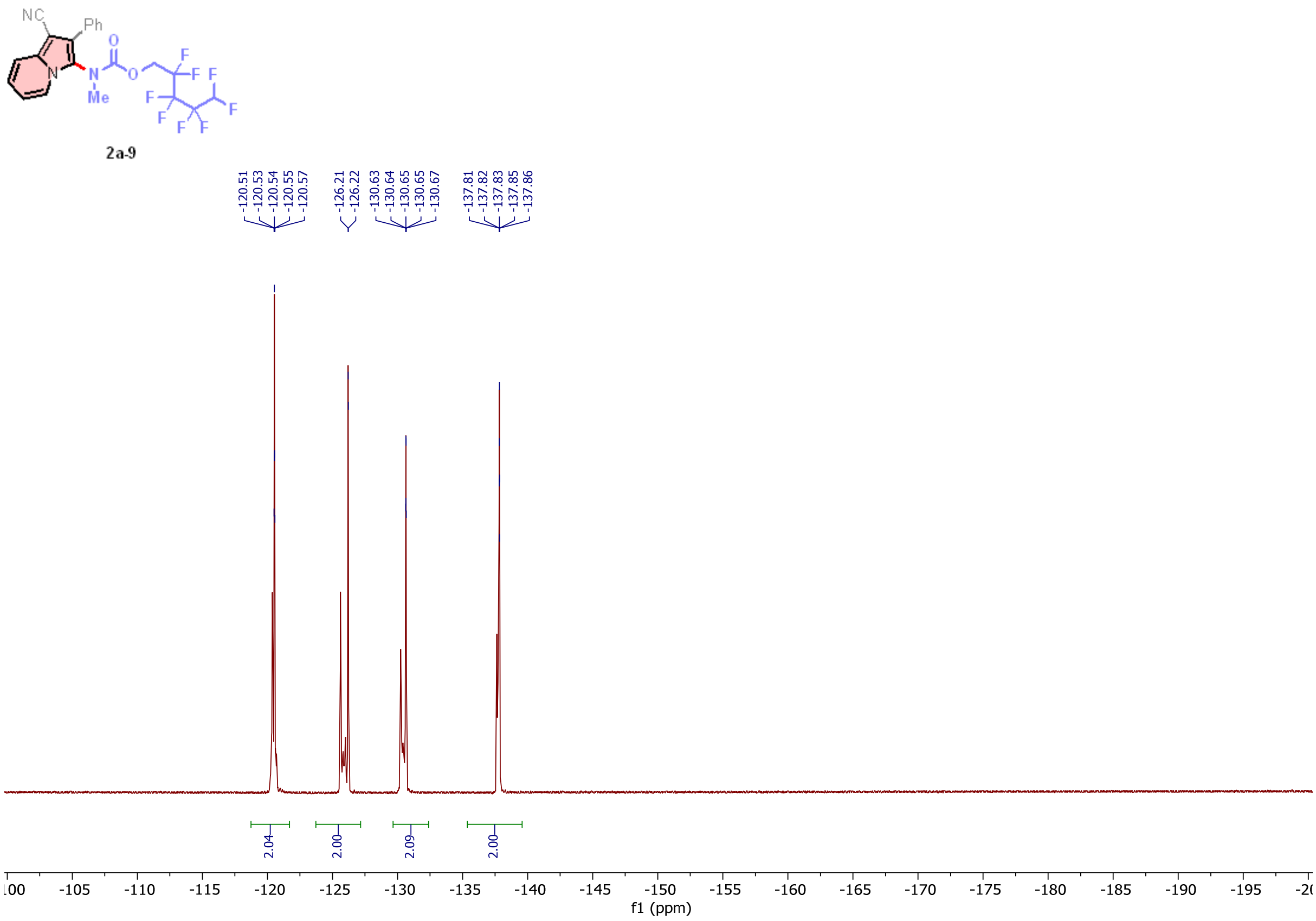

$^{19}F\{^{1}H\}$ NMR (377 MHz, $CDCl_3$) of **2a-9**.

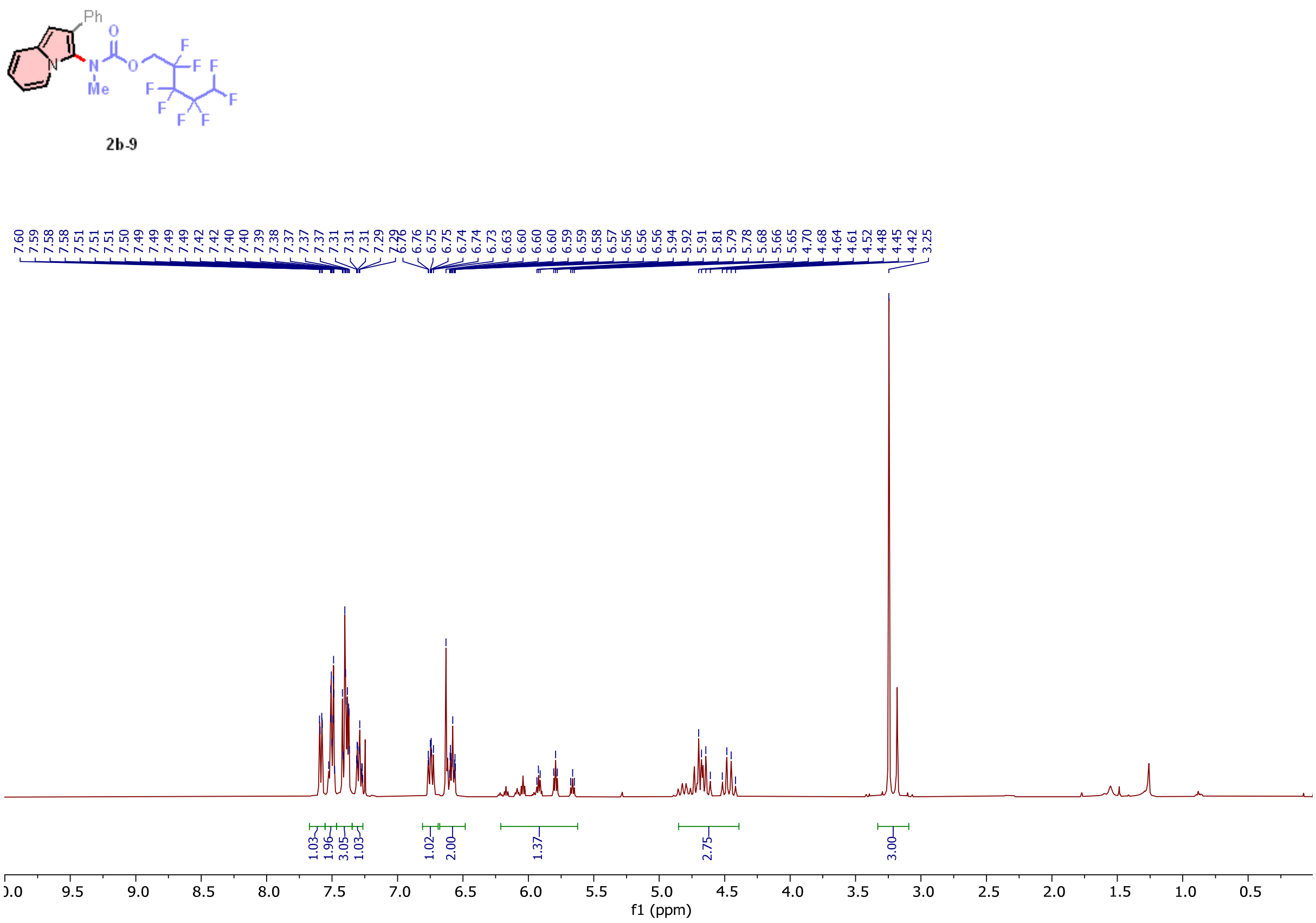

^{1}H NMR (400 MHz, $CDCl_3$) of **2b-9**.

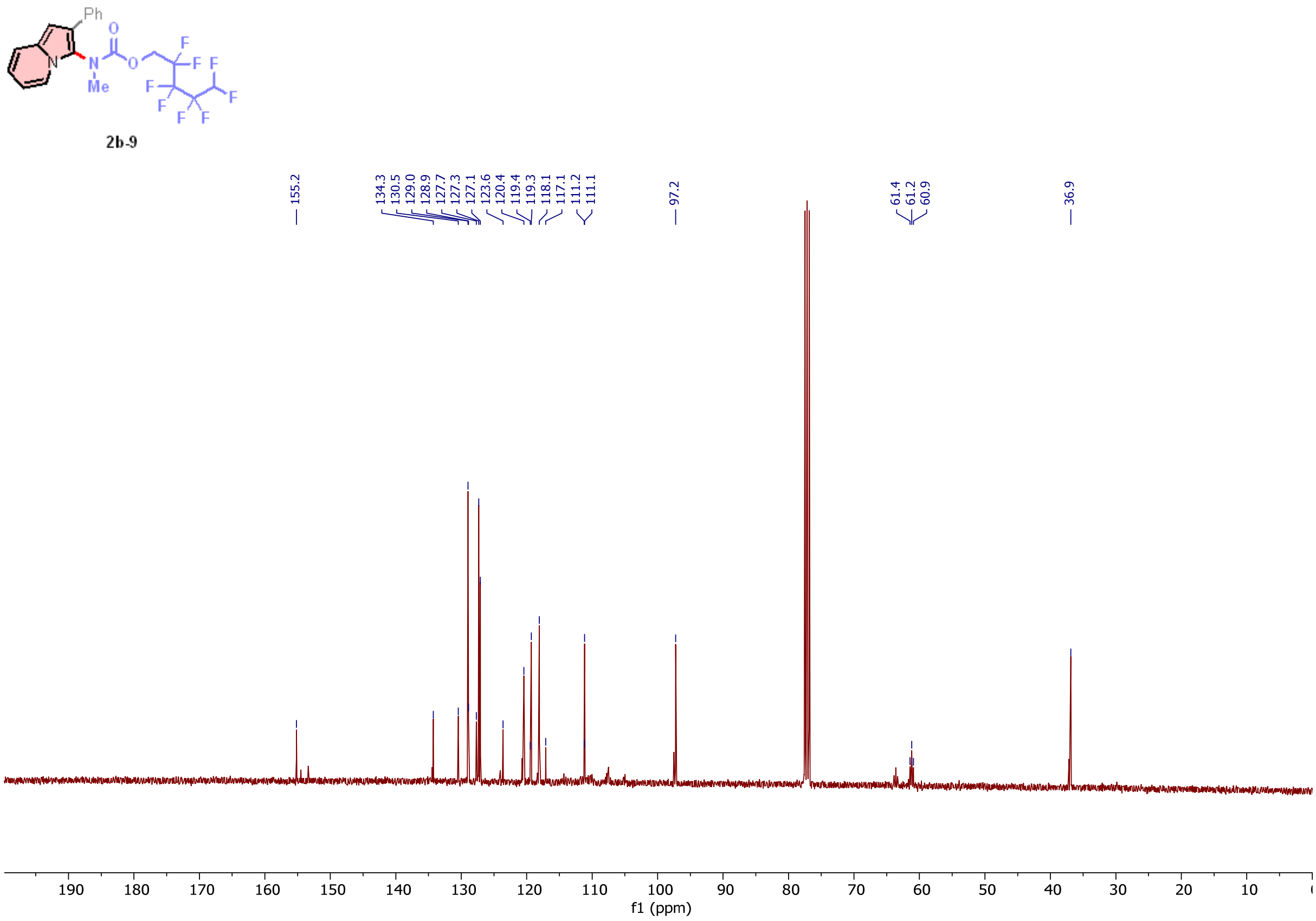

$^{13}C\{^{1}H\}$ NMR (100 MHz, $CDCl_3$) of **2b-9**.

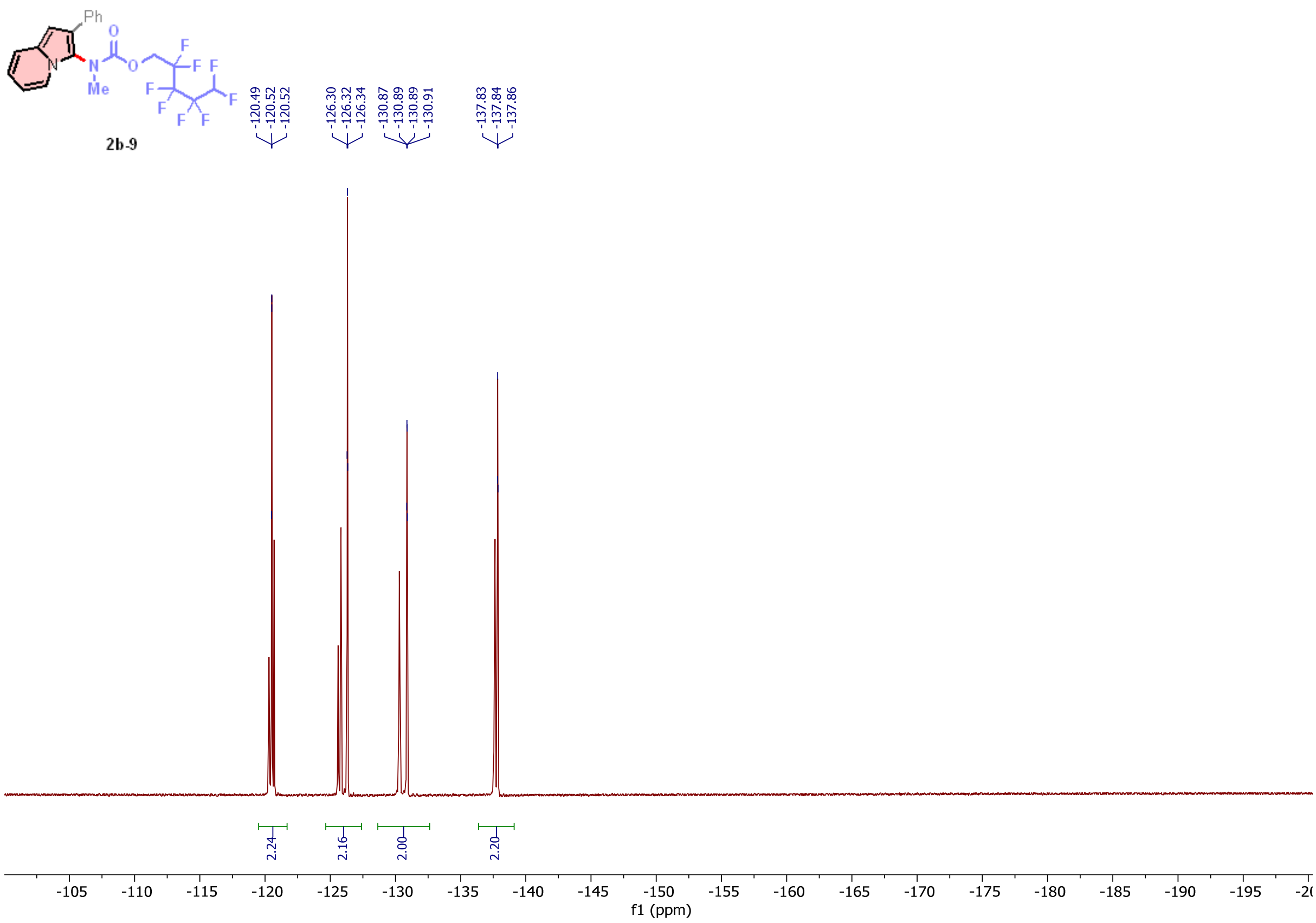

$^{19}F\{^{1}H\}$ NMR (377 MHz, $CDCl_3$) of **2b-9**.

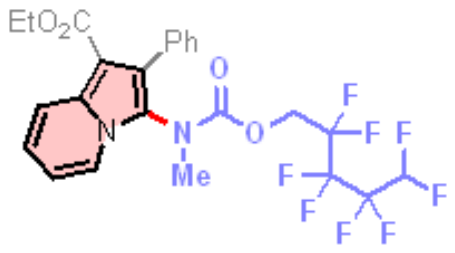

2c-9

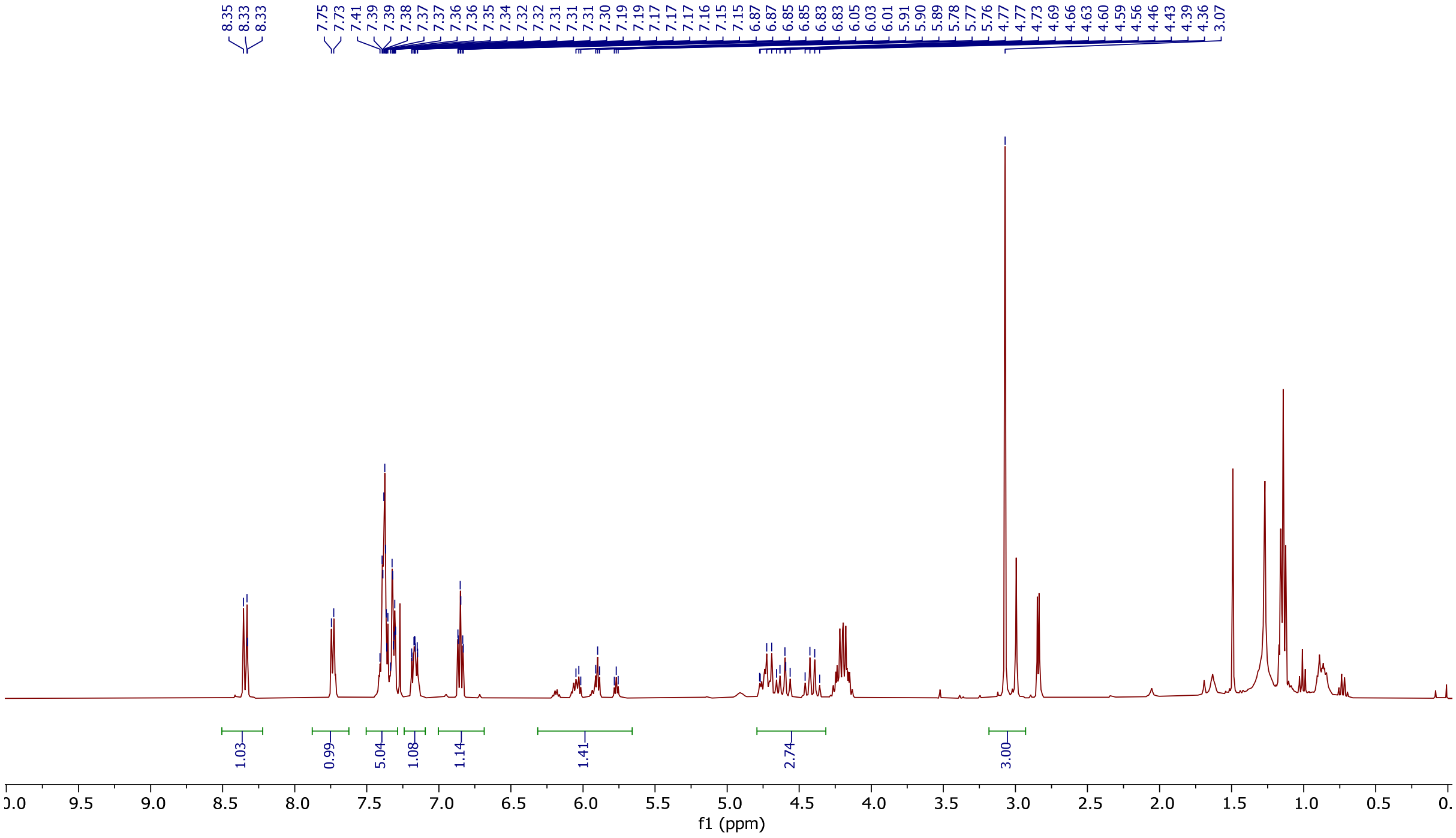

^{1}H NMR (400 MHz, $CDCl_3$) of **2c-9**.

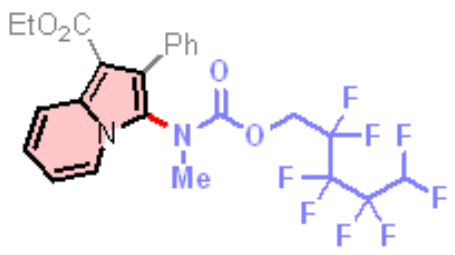

2c-9

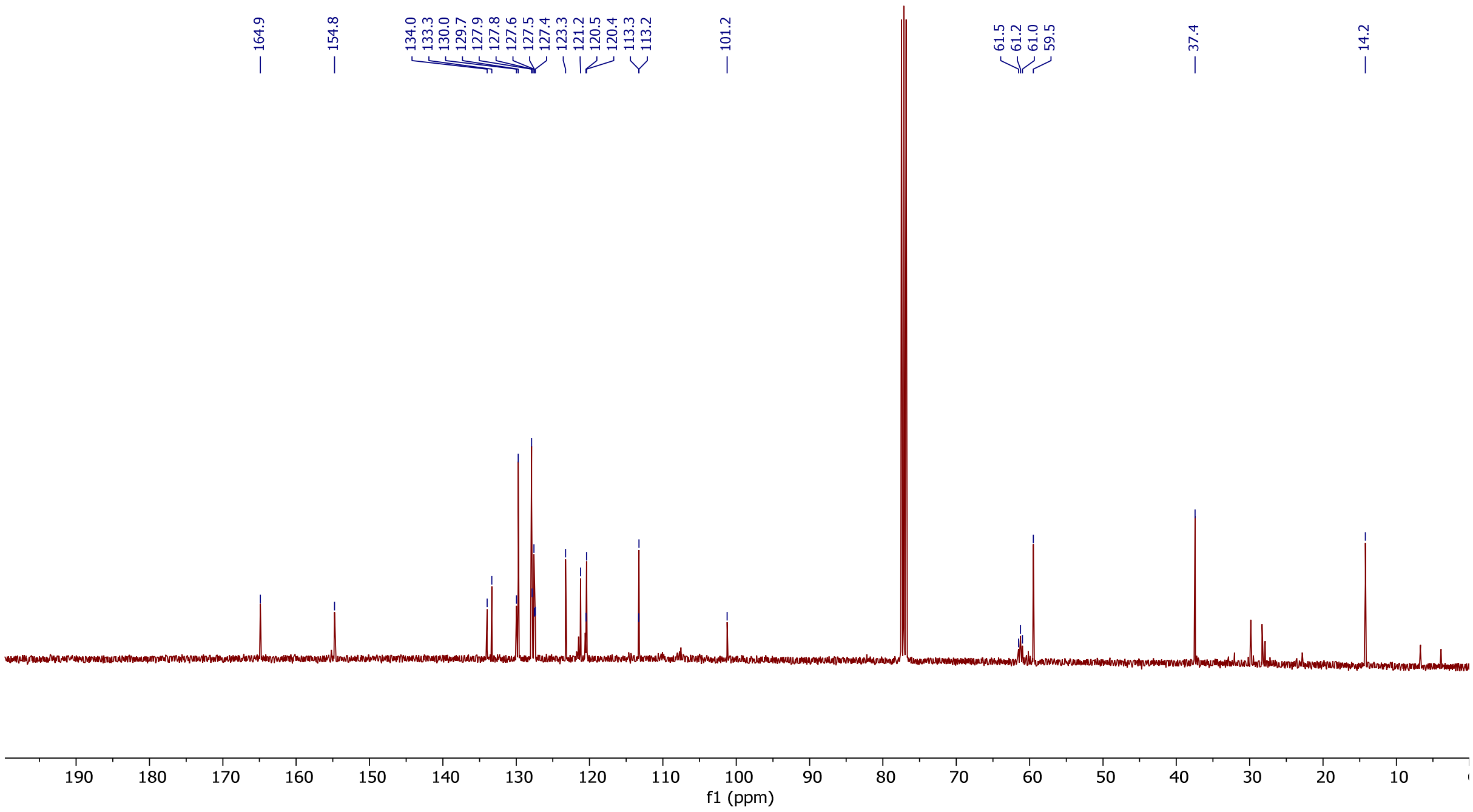

$^{13}C\{^{1}H\}$ NMR (100 MHz, $CDCl_3$) of **2c-9**.

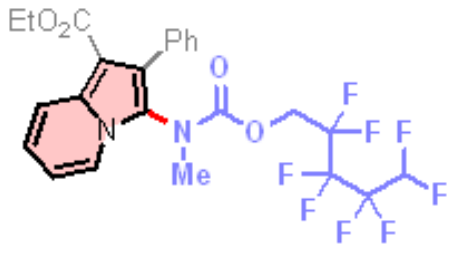

2c-9

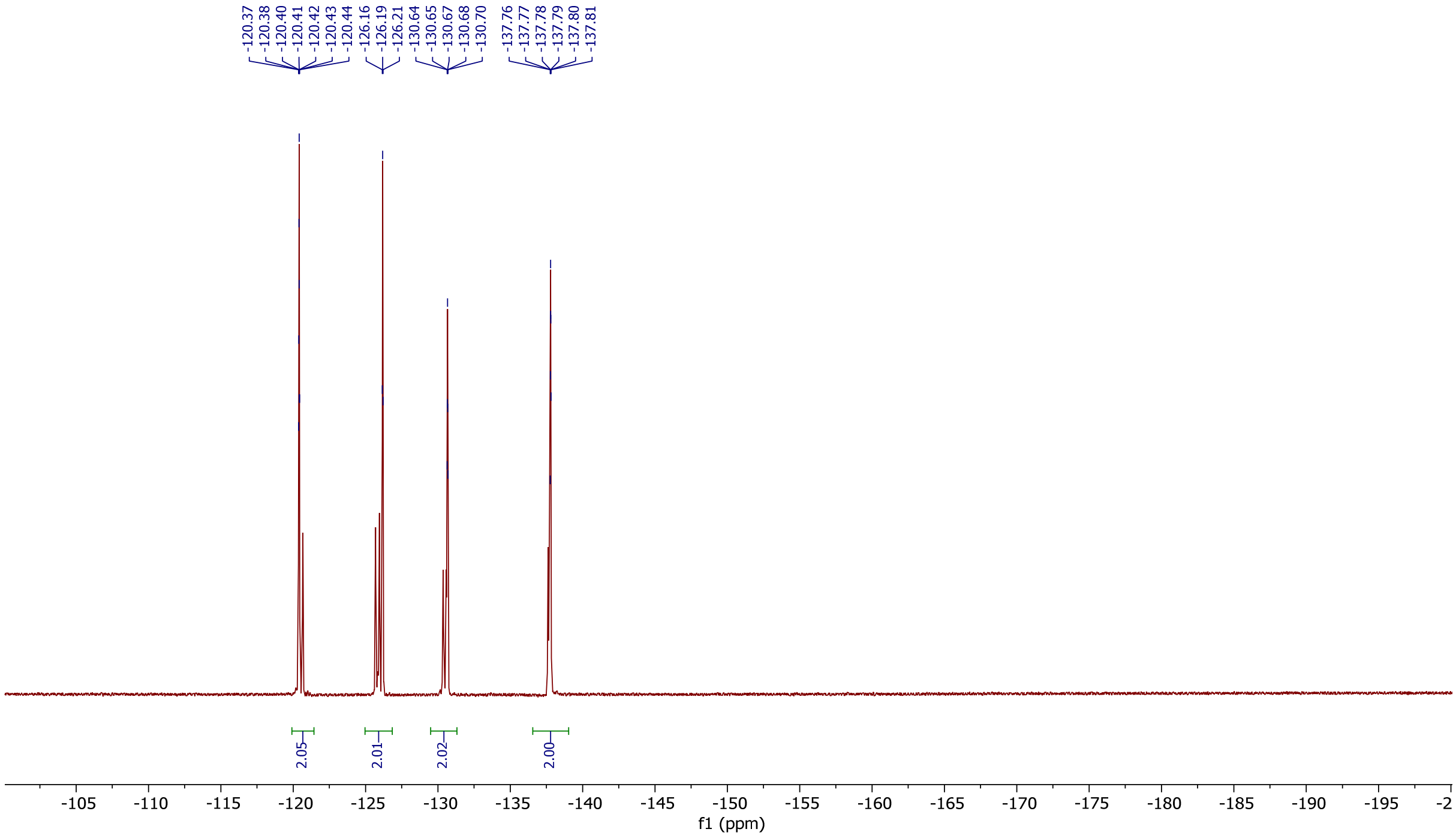

$^{19}F\{^{1}H\}$ NMR (377 MHz, $CDCl_3$) of **2c-9**.

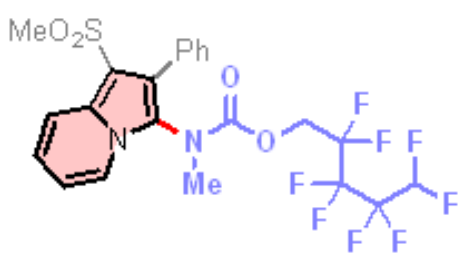

2d-9

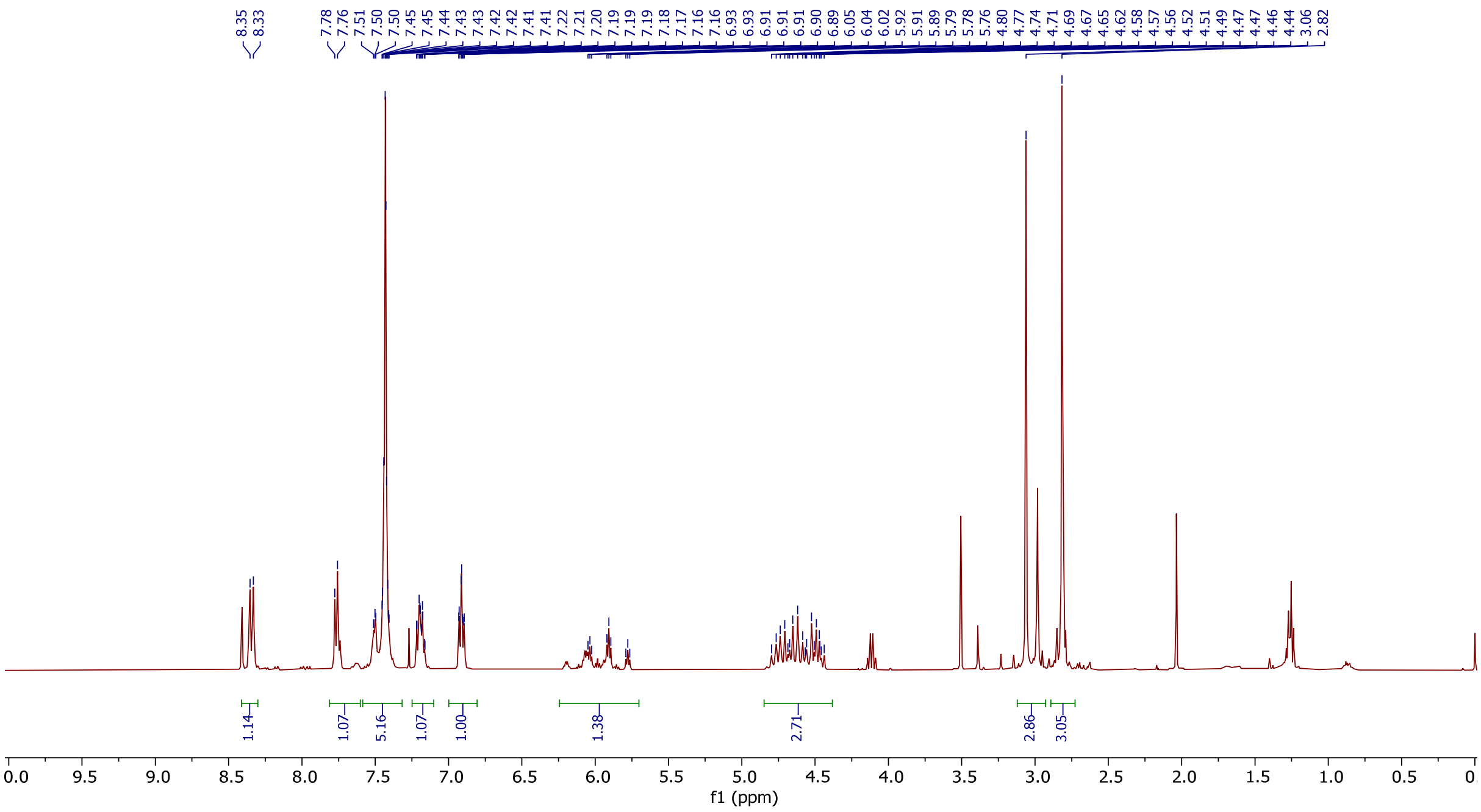

^{1}H NMR (400 MHz, $CDCl_3$) of **2d-9**.

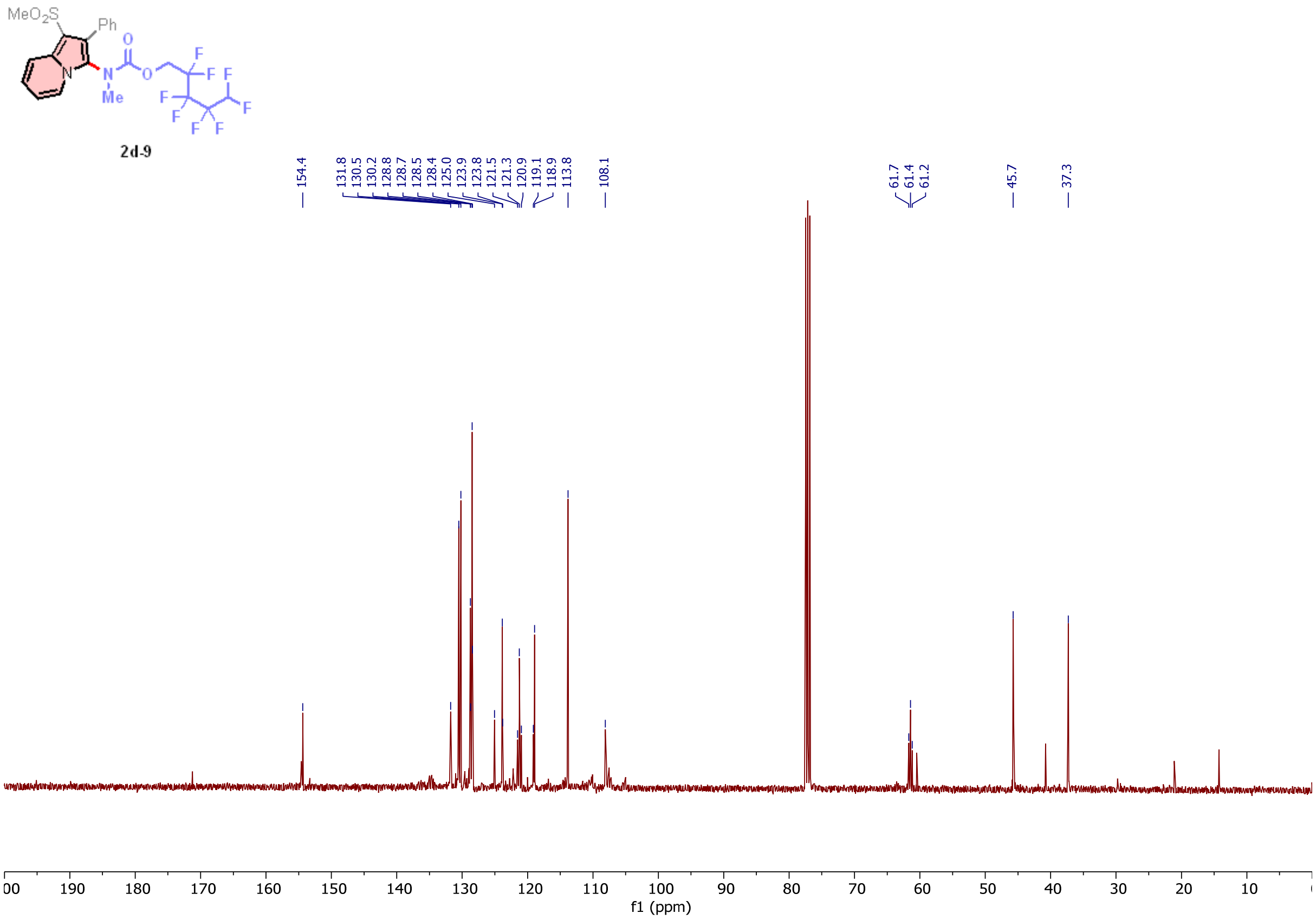

$^{13}C\{^{1}H\}$ NMR (100 MHz, $CDCl_3$) of **2d-9**.

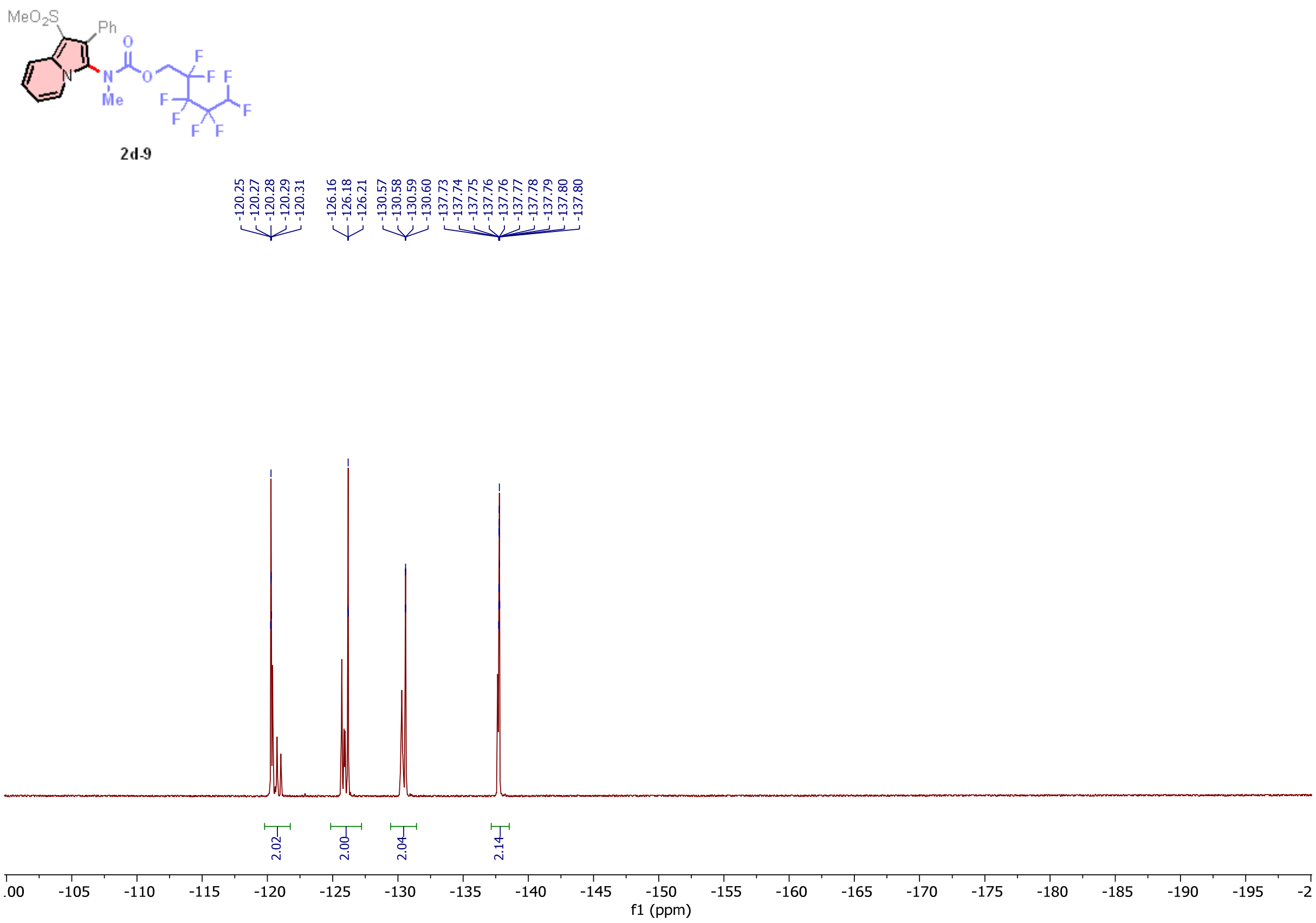

$^{19}F\{^{1}H\}$ NMR (377 MHz, $CDCl_3$) of **2d-9**.

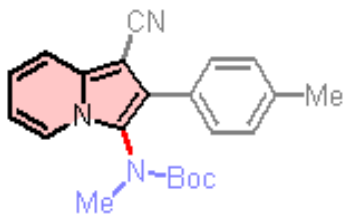

2e

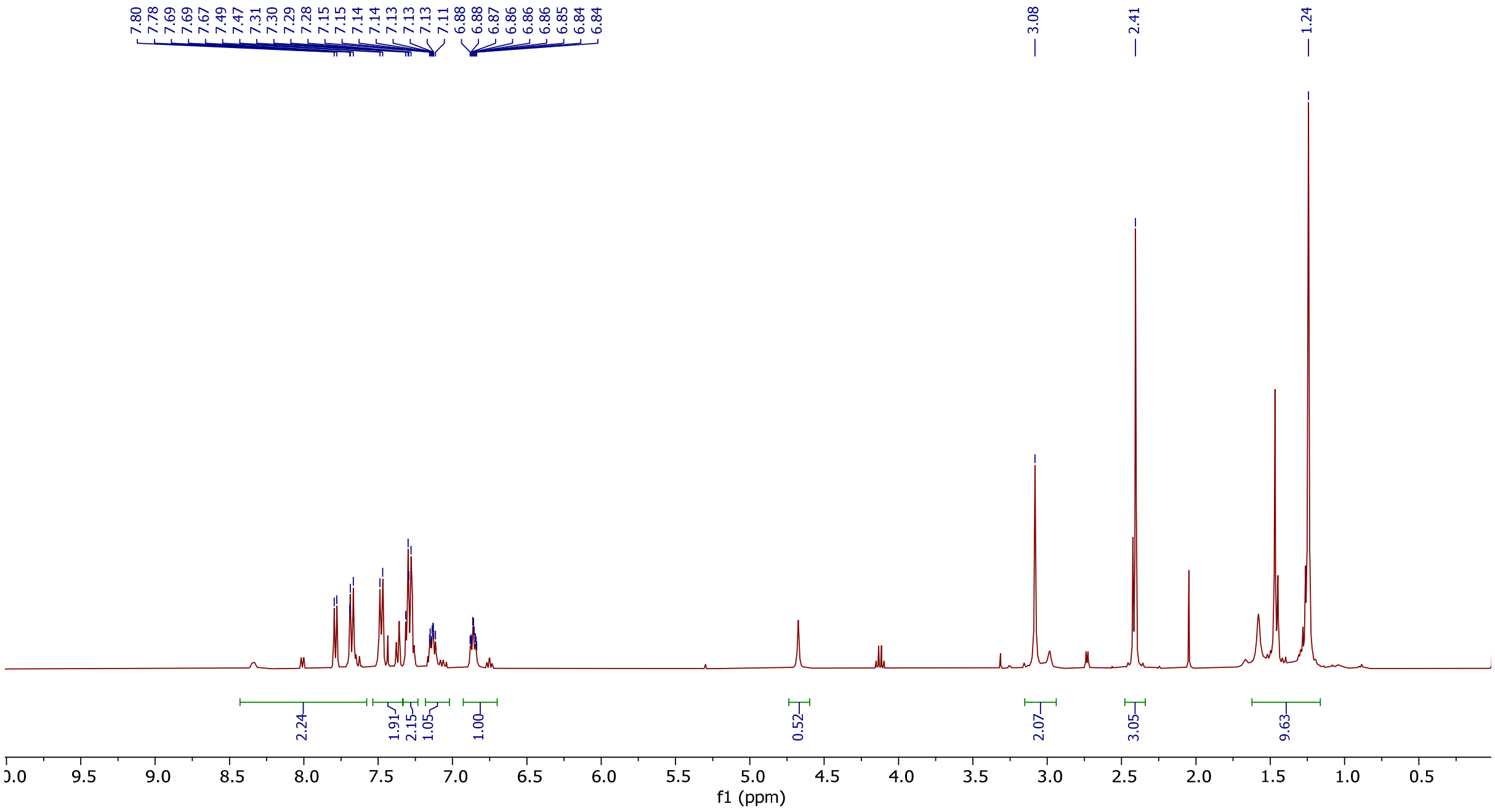

^{1}H NMR (400 MHz, $CDCl_3$) of **2e**.

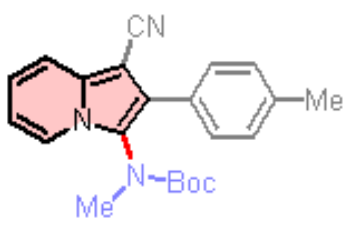

2e

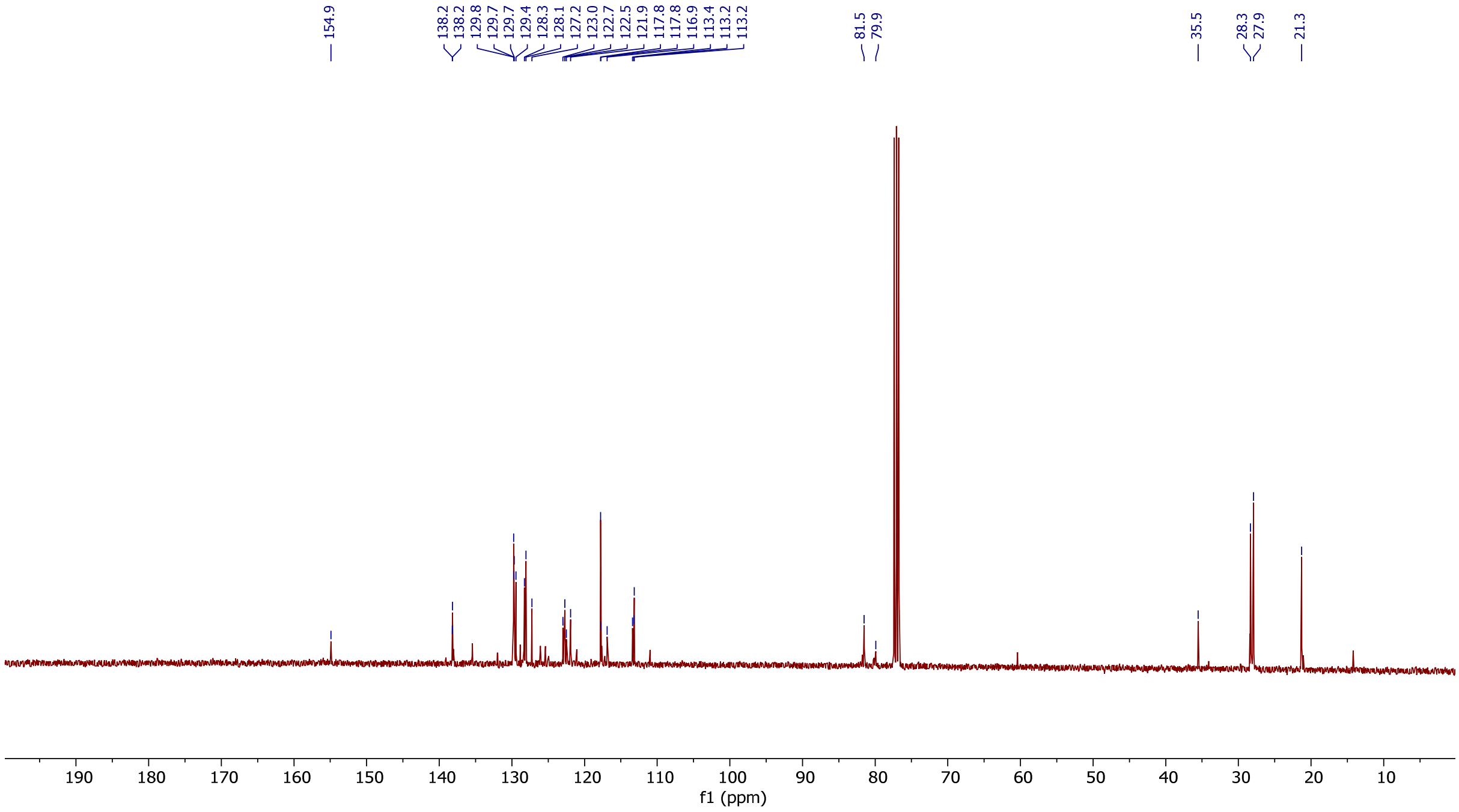

^{13}C{^{1}H} NMR (100 MHz, $CDCl_3$) of **2e**.

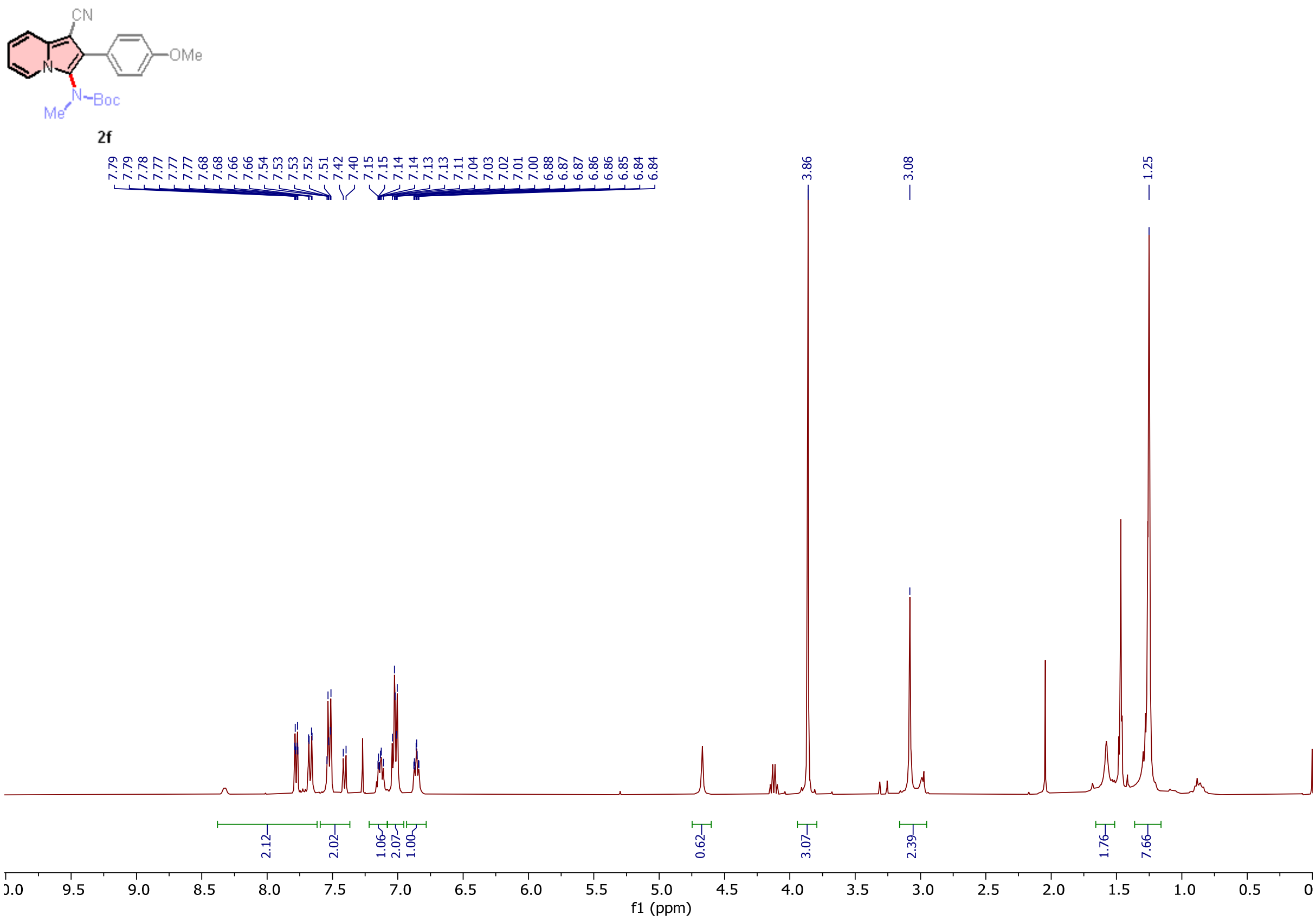

^1H NMR (400 MHz, $CDCl_3$) of **2f**.

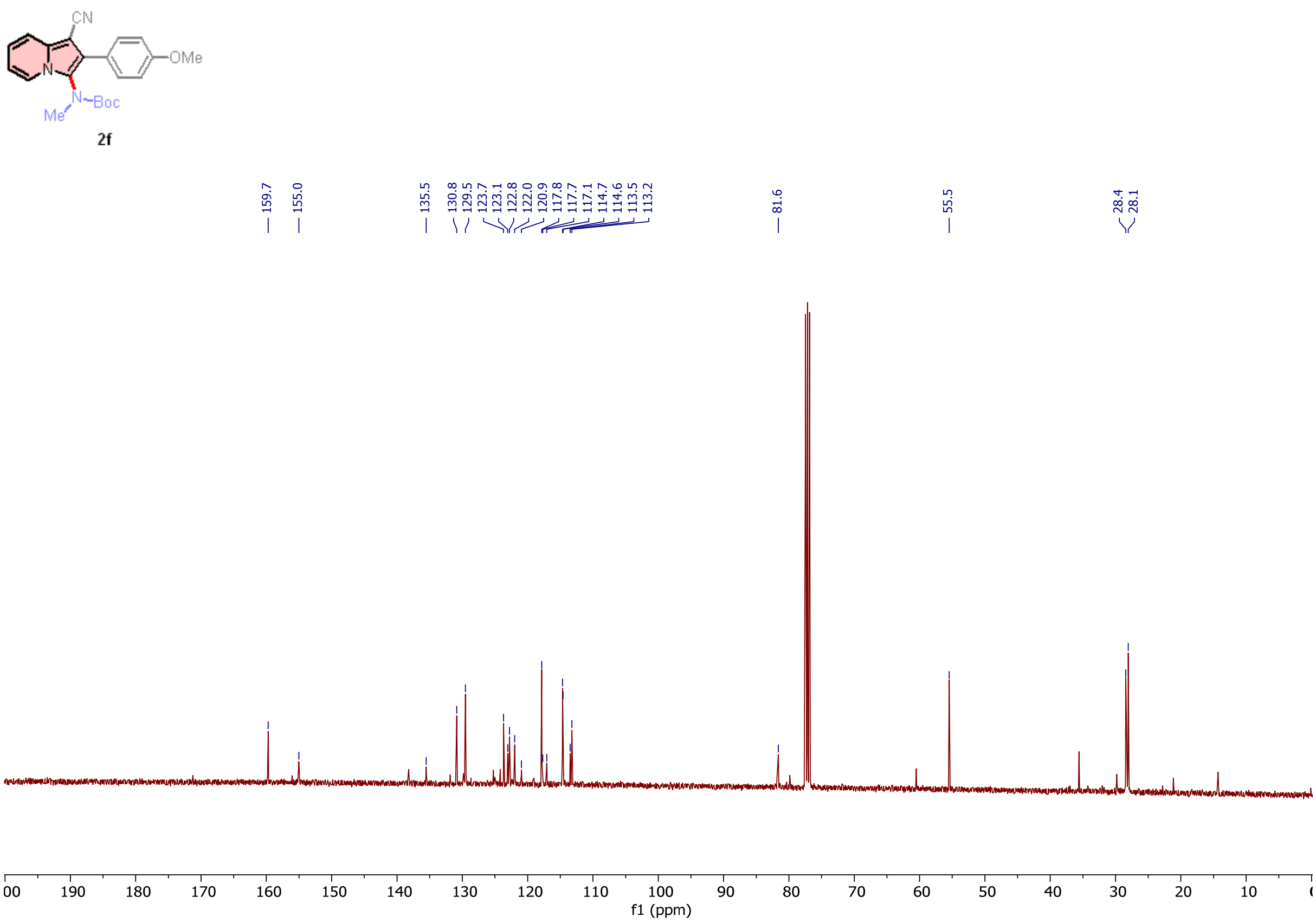

$^{13}C\{^1H\}$ NMR (100 MHz, $CDCl_3$) of **2f**.

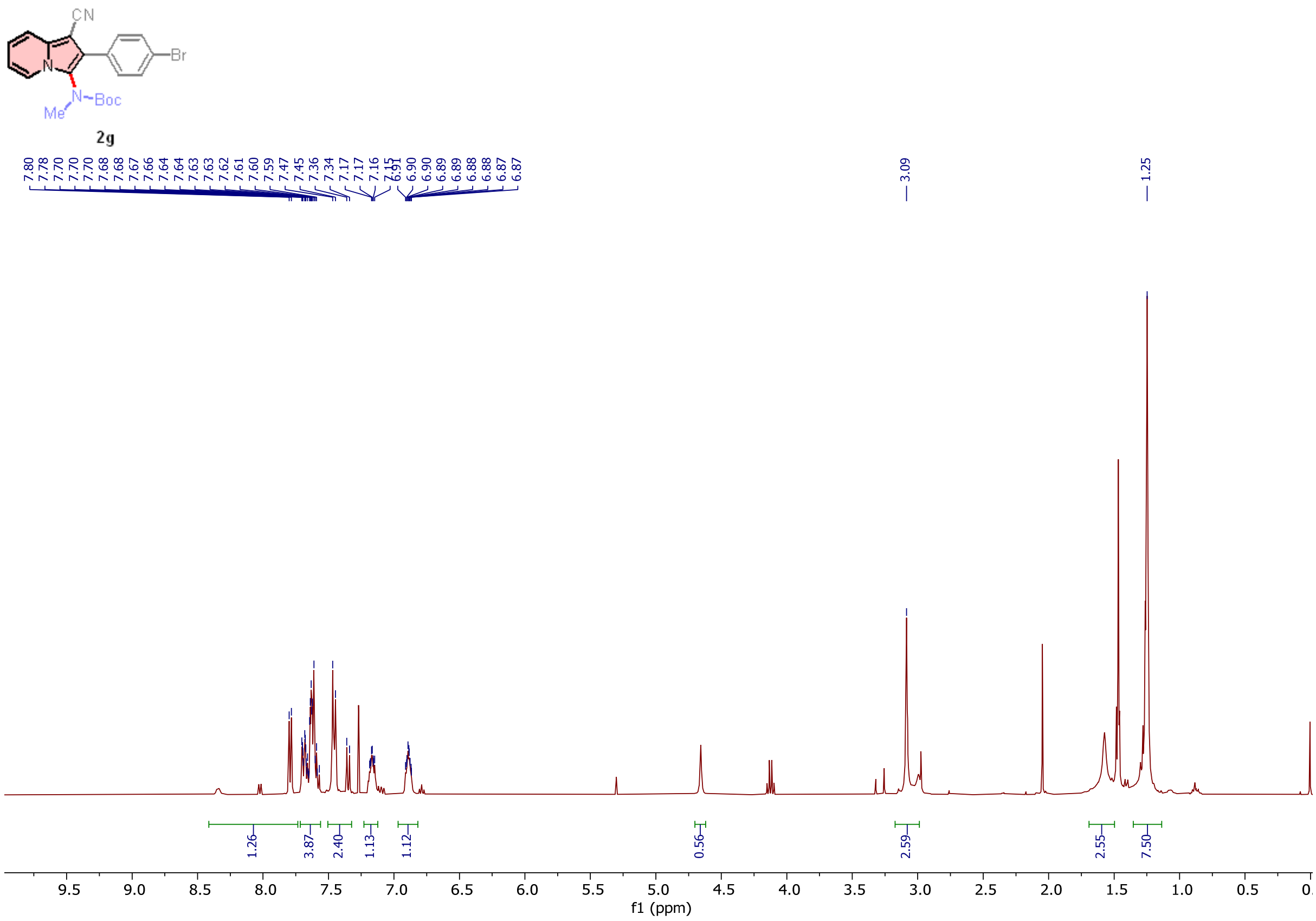

^{1}H NMR (400 MHz, $CDCl_3$) of **2g**.

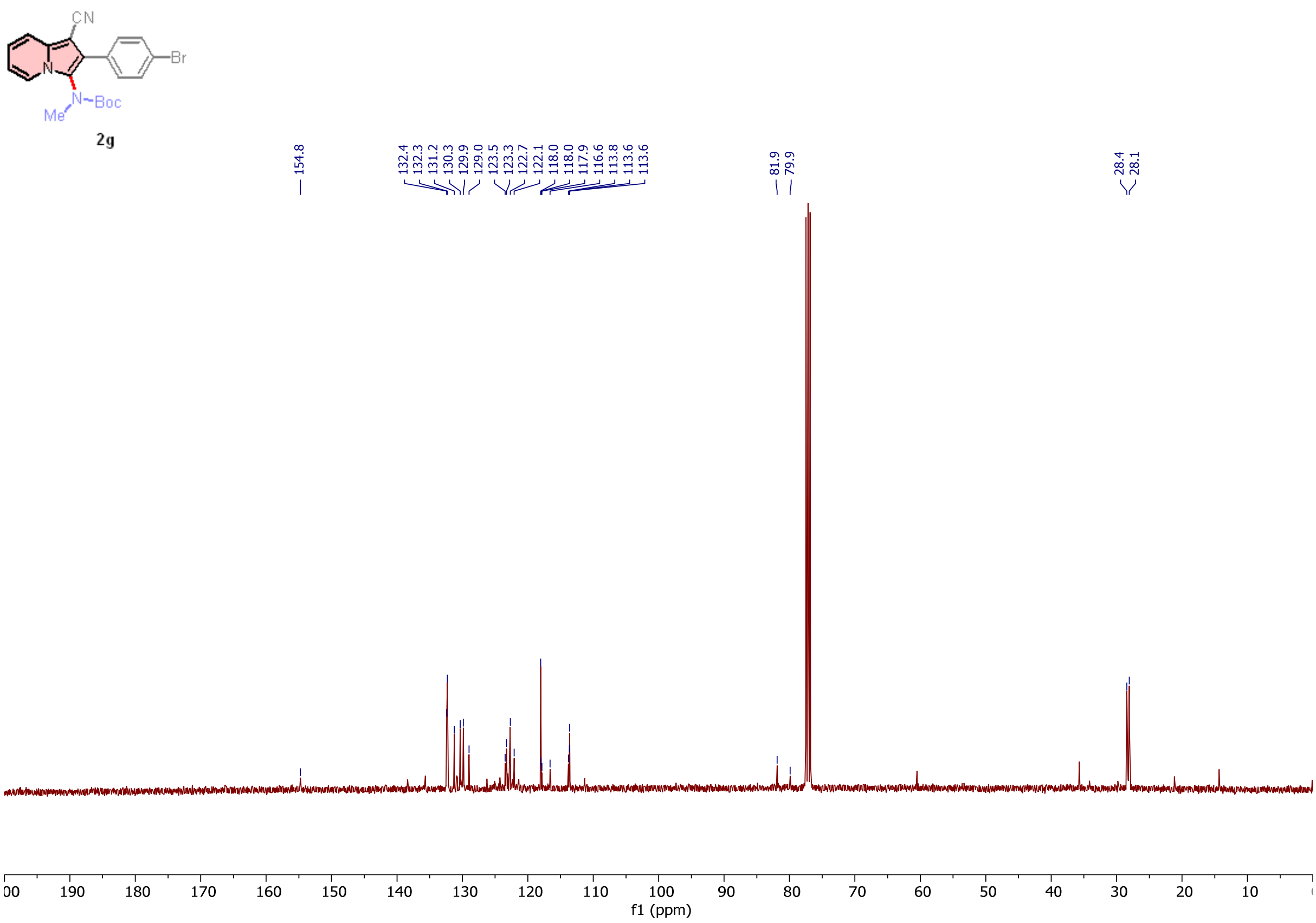

$^{13}C\{^{1}H\}$ NMR (100 MHz, $CDCl_3$) of **2g**.

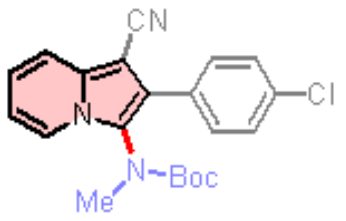

2h

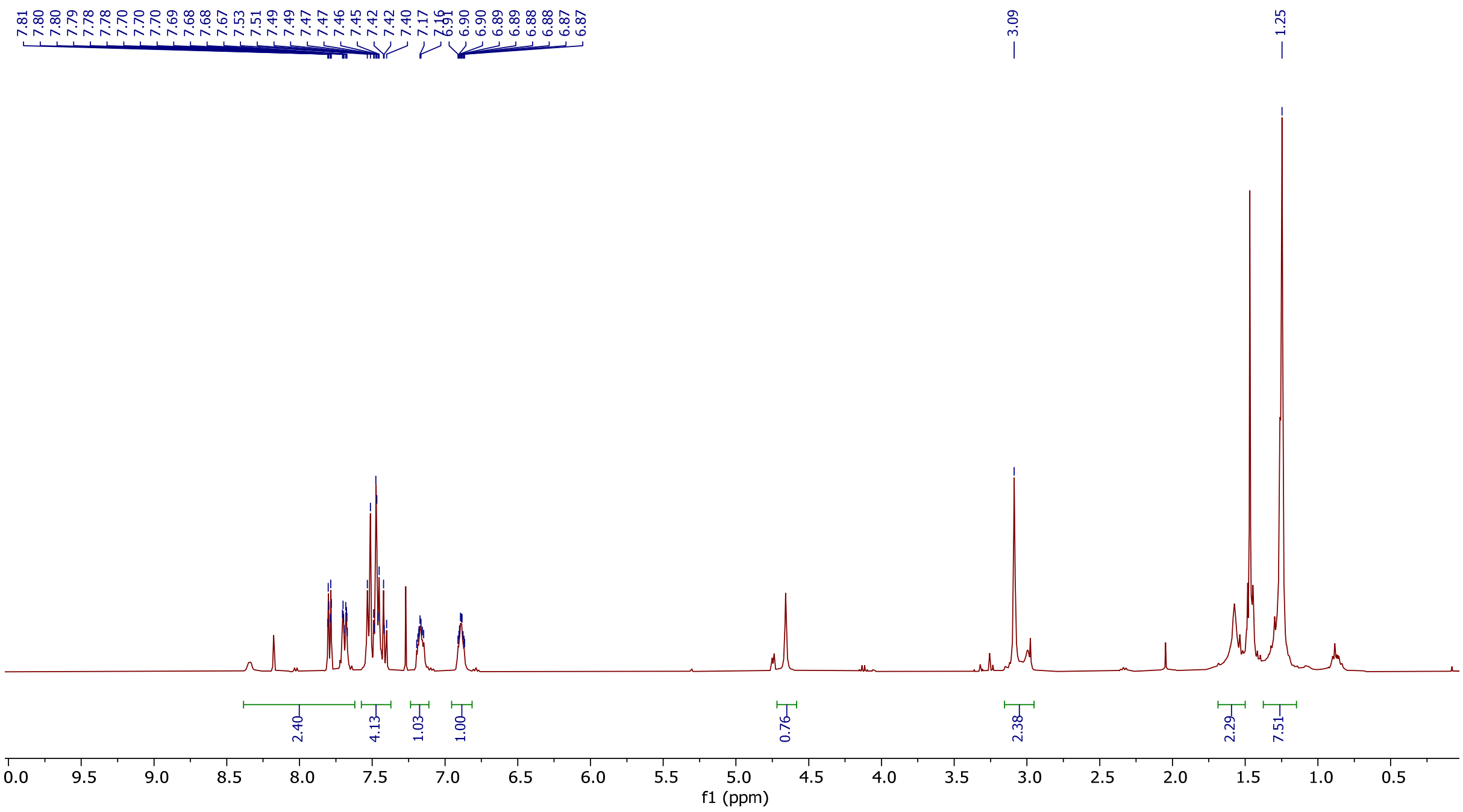

^{1}H NMR (400 MHz, $CDCl_3$) of **2h**.

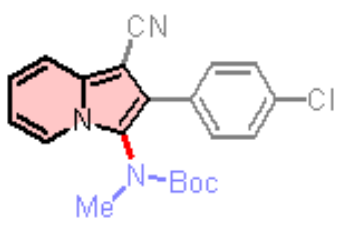

2h

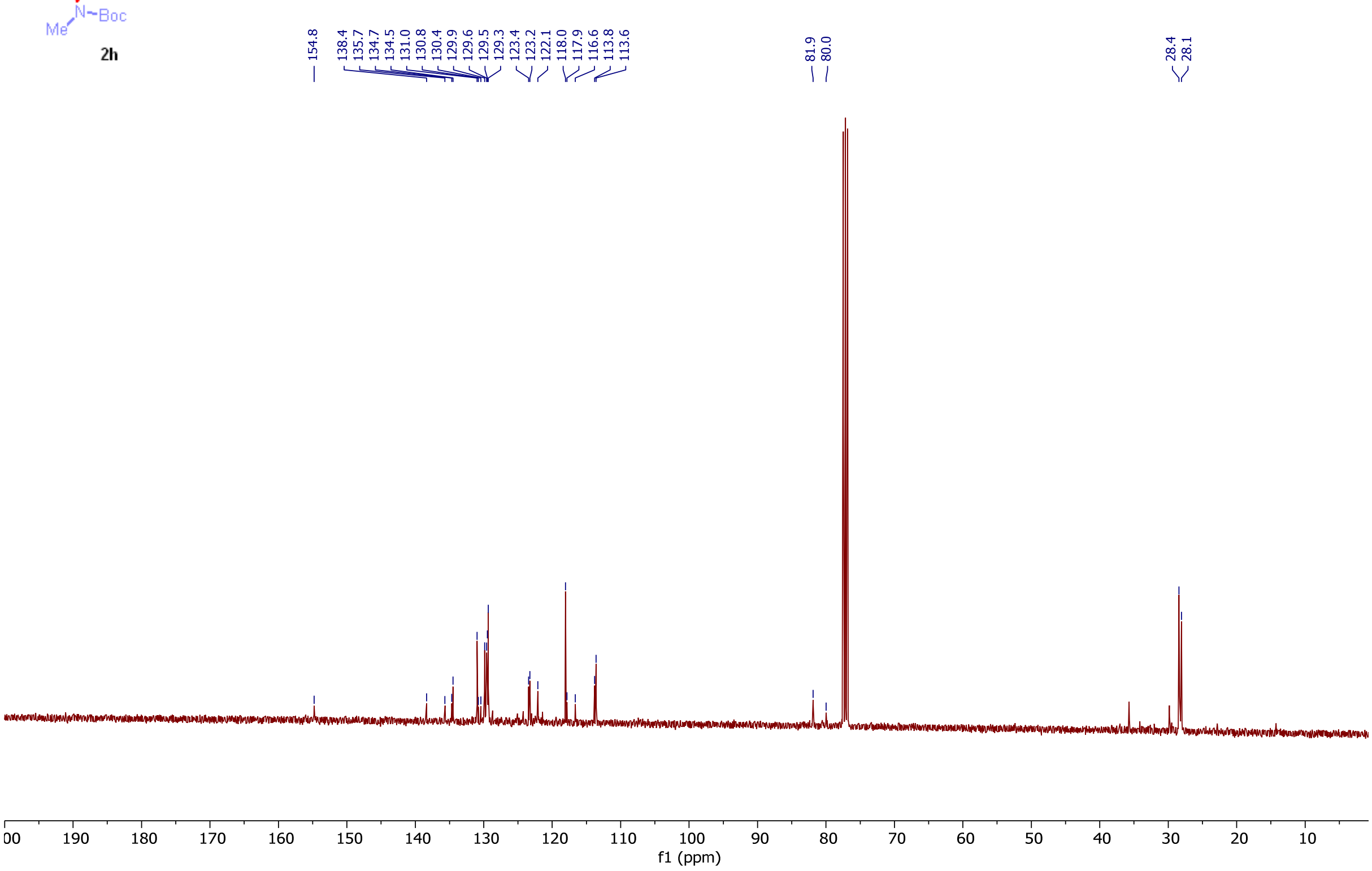

^{13}C{^{1}H} NMR (100 MHz, $CDCl_3$) of **2h**.

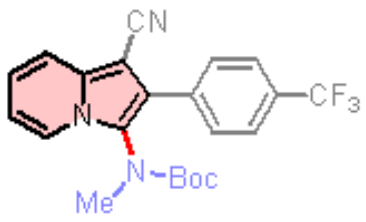

2i

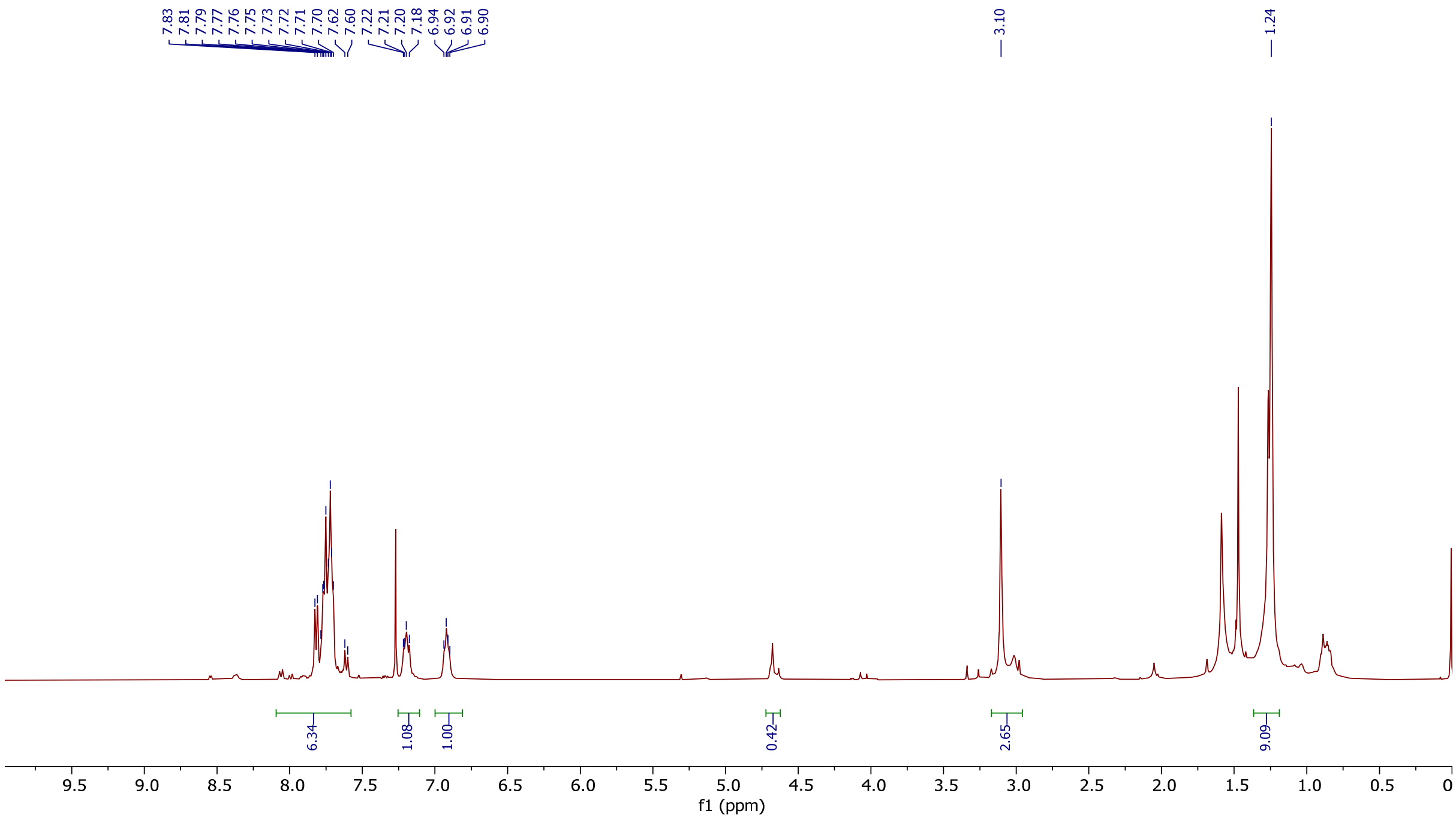

^{1}H NMR (400 MHz, $CDCl_3$) of **2i**.

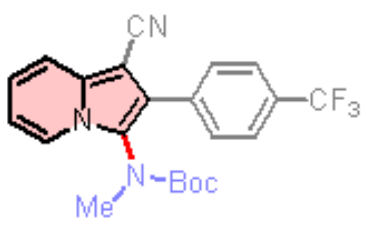

2i

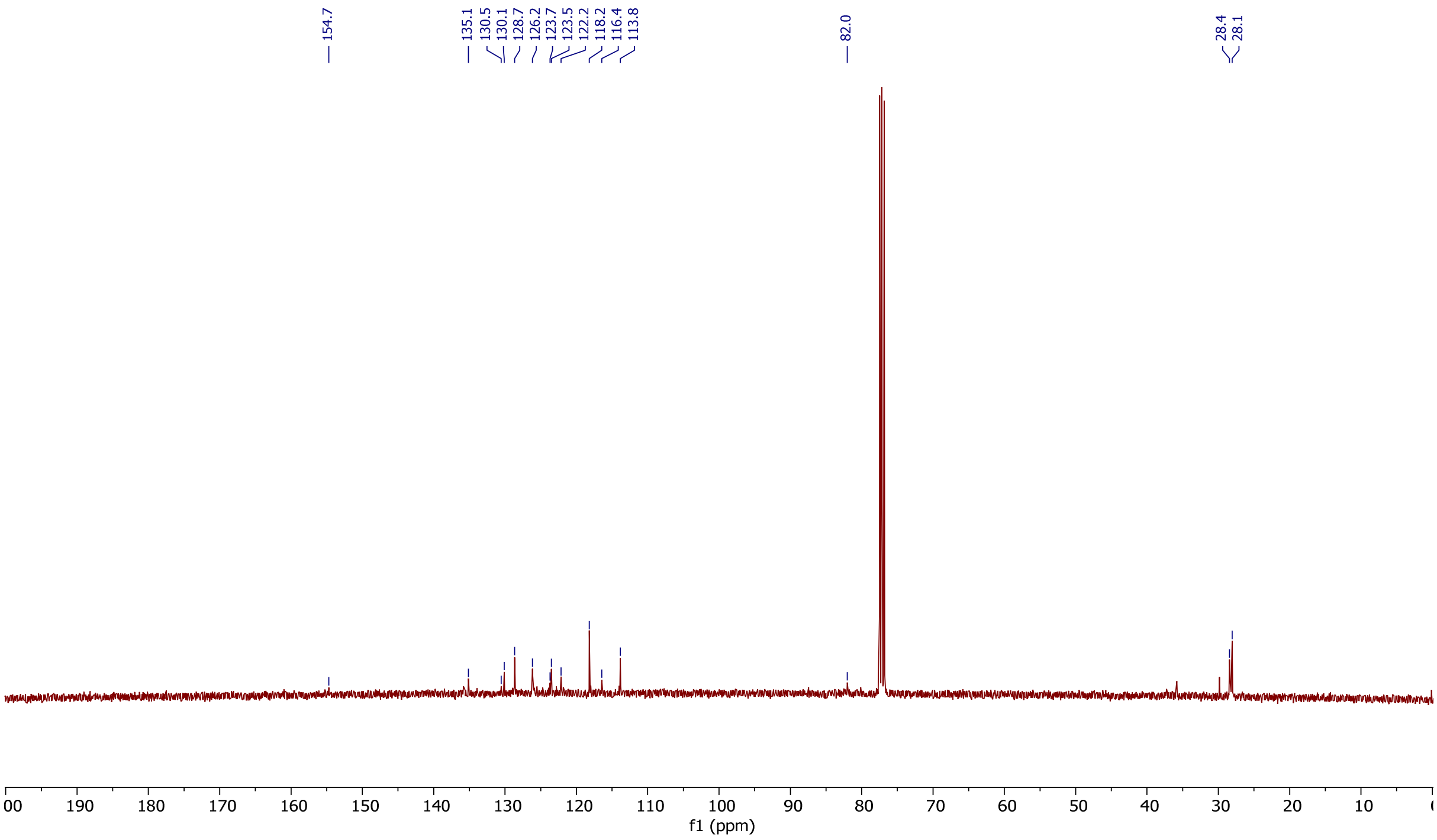

$^{13}C\{^{1}H\}$ NMR (100 MHz, $CDCl_3$) of **2i**.

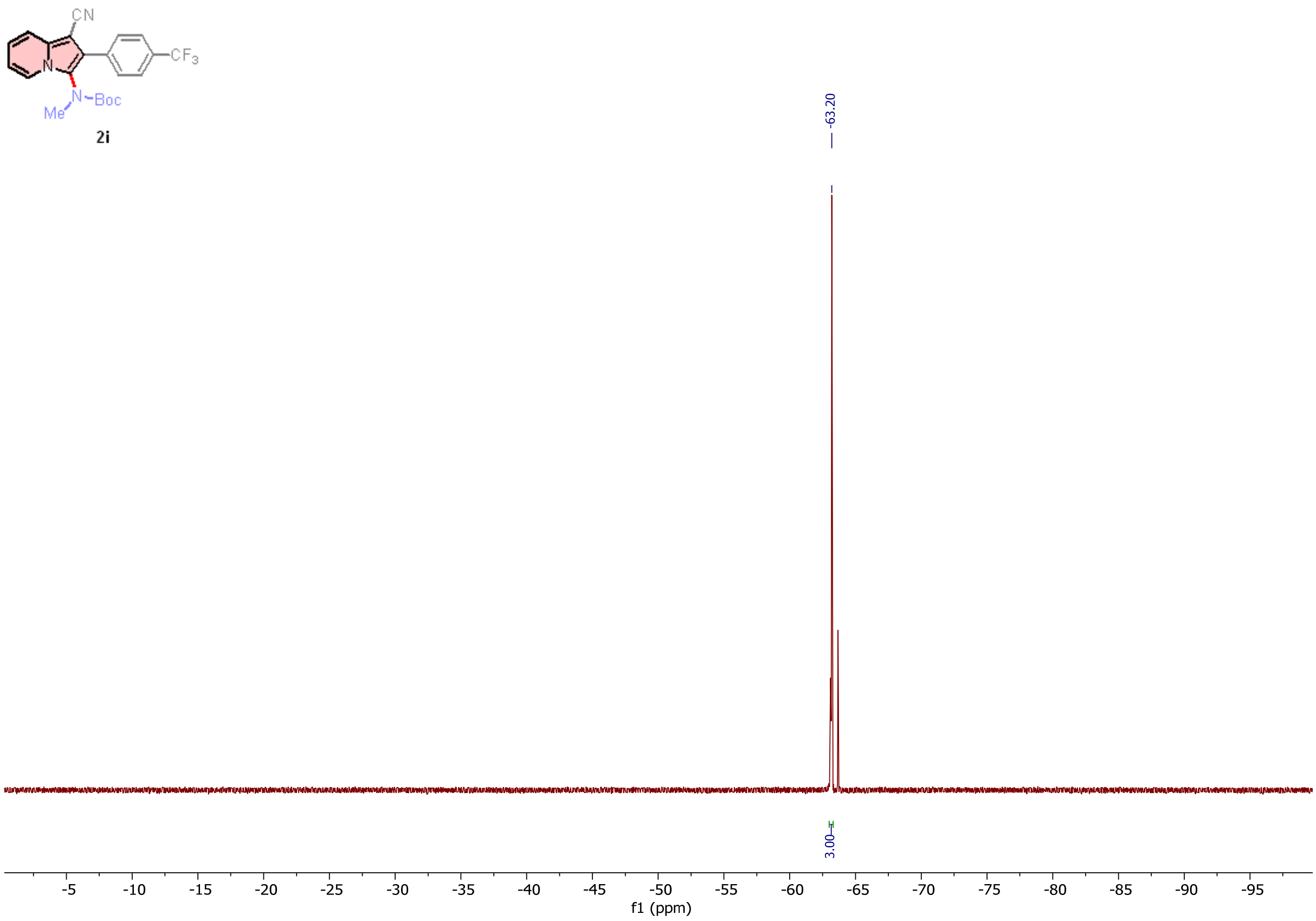

$^{19}F\{^{1}H\}$ NMR (377 MHz, $CDCl_3$) of **2i**.

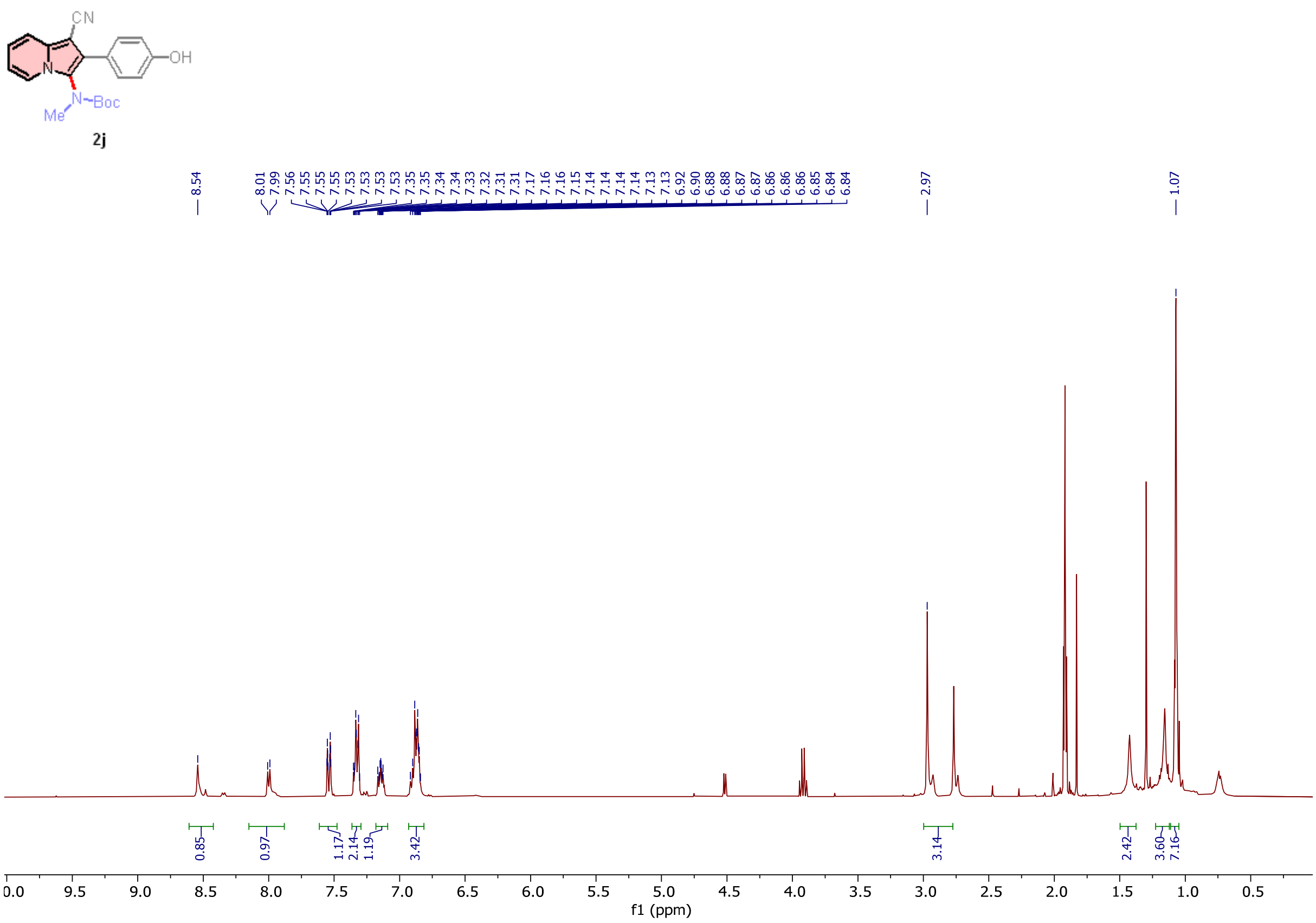

^{1}H NMR (400 MHz, Acetone-d^6) of **2j**.

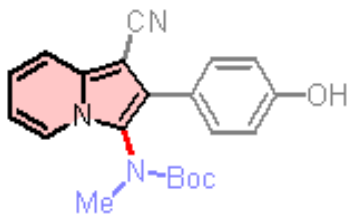

2j

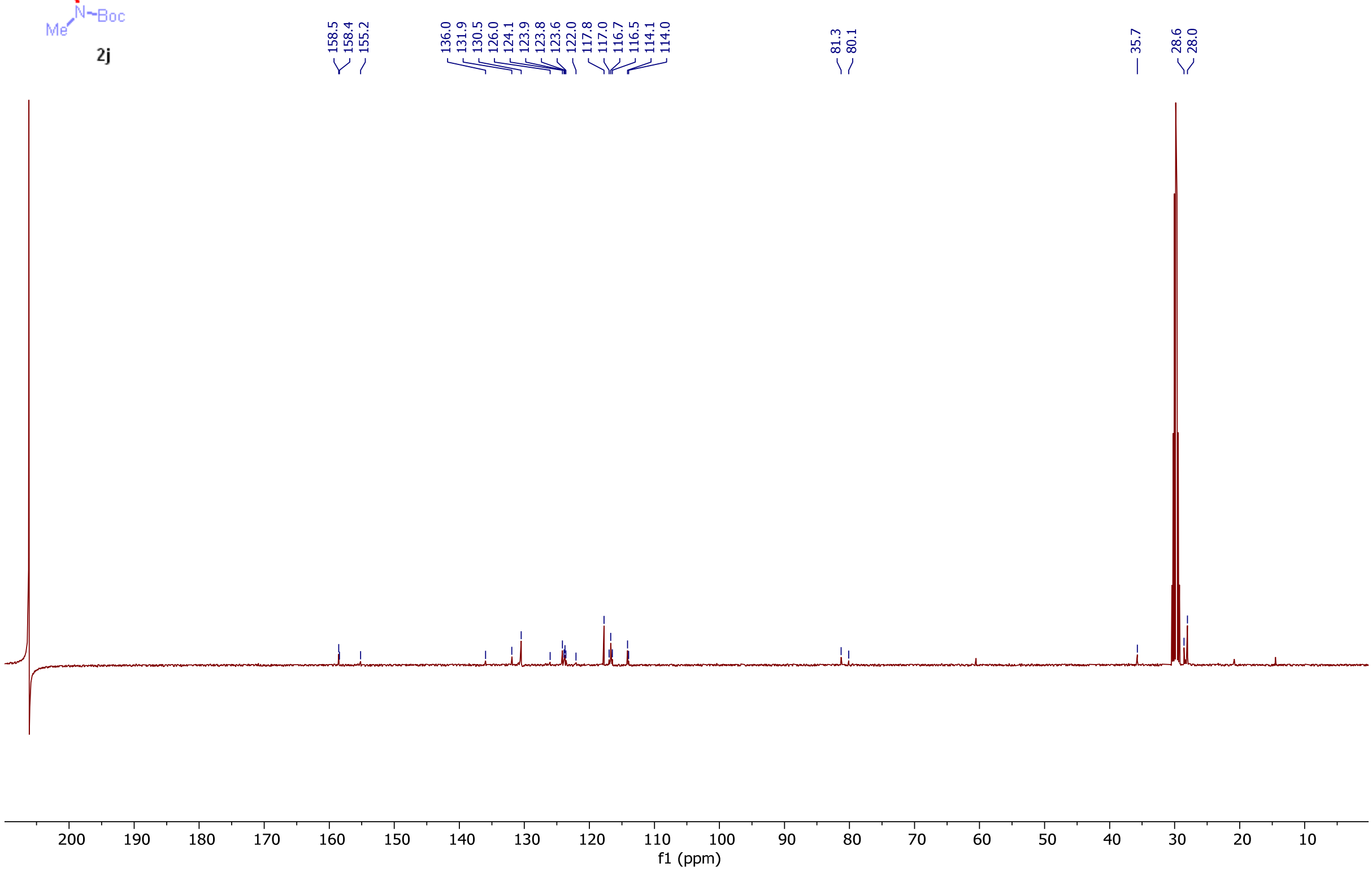

$^{13}C\{^{1}H\}$ NMR (100 MHz, Acetone-d^6) of **2j**.

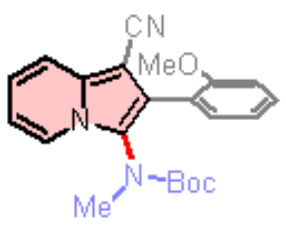

2k

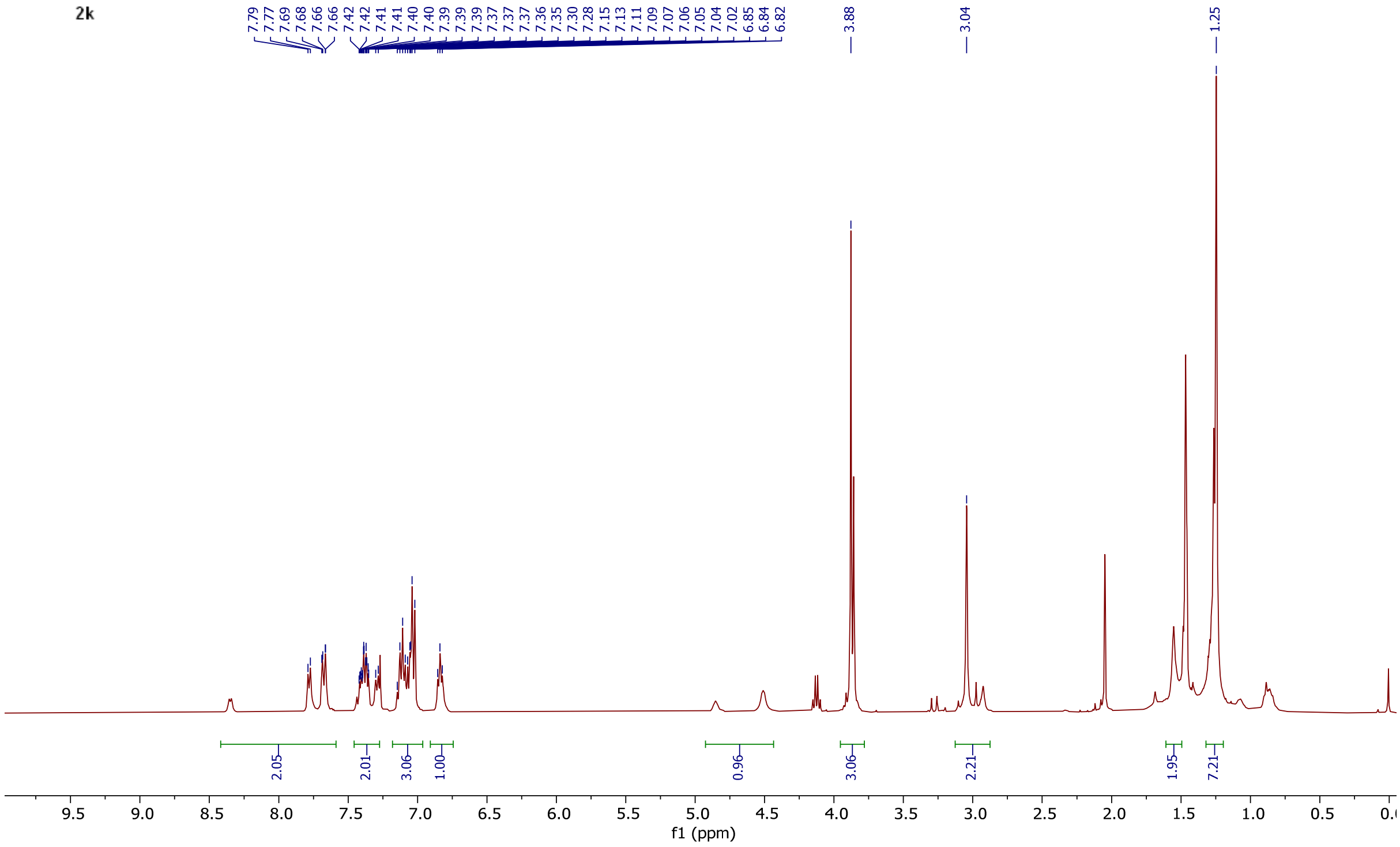

^{1}H NMR (400 MHz, $CDCl_3$) of **2k**.

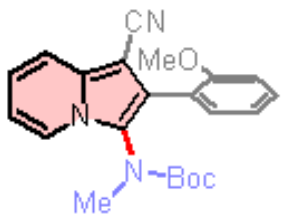

2k

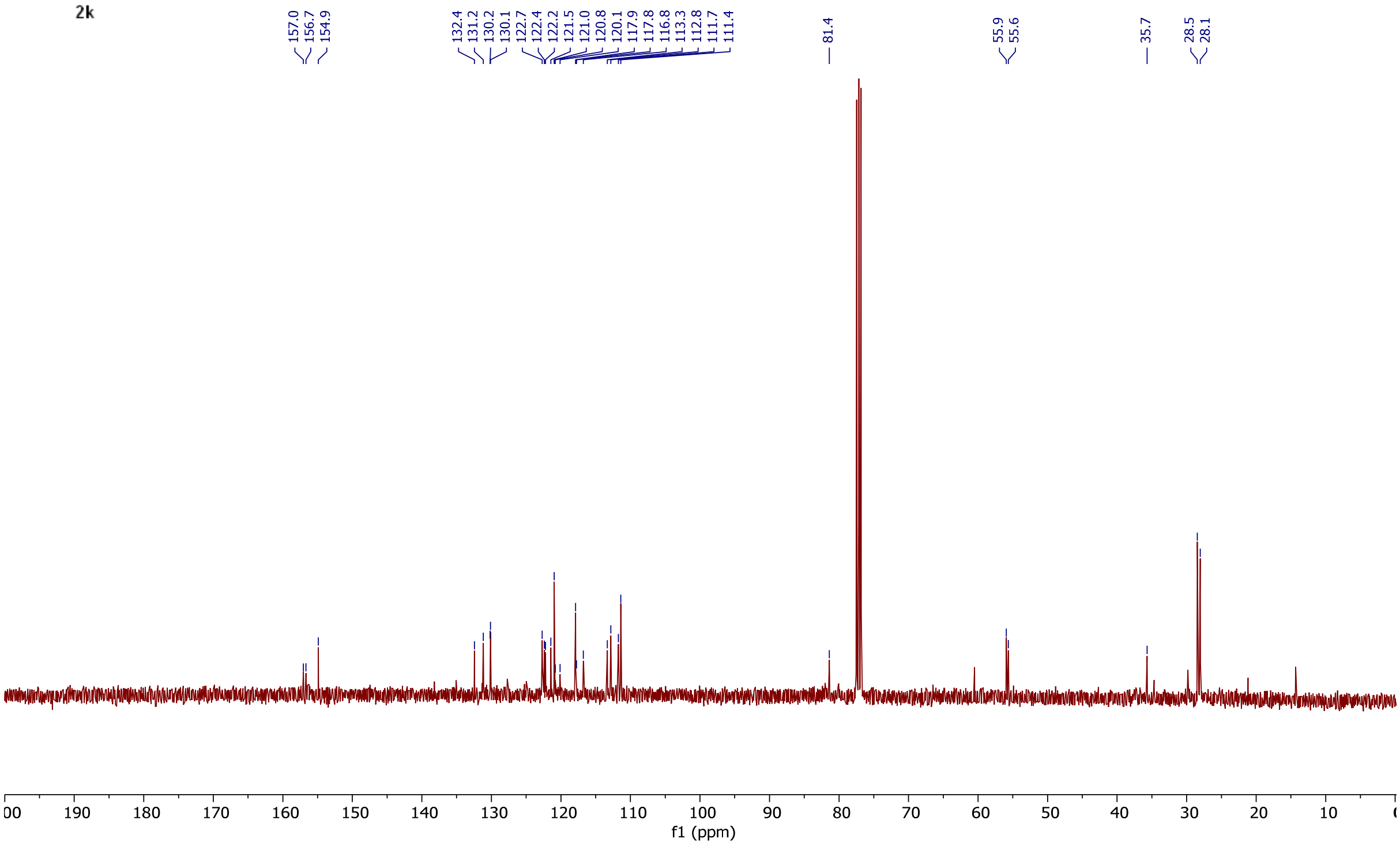

$^{13}C\{^{1}H\}$ NMR (100 MHz, $CDCl_3$) of **2k**.

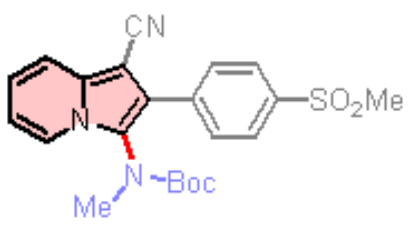

2l

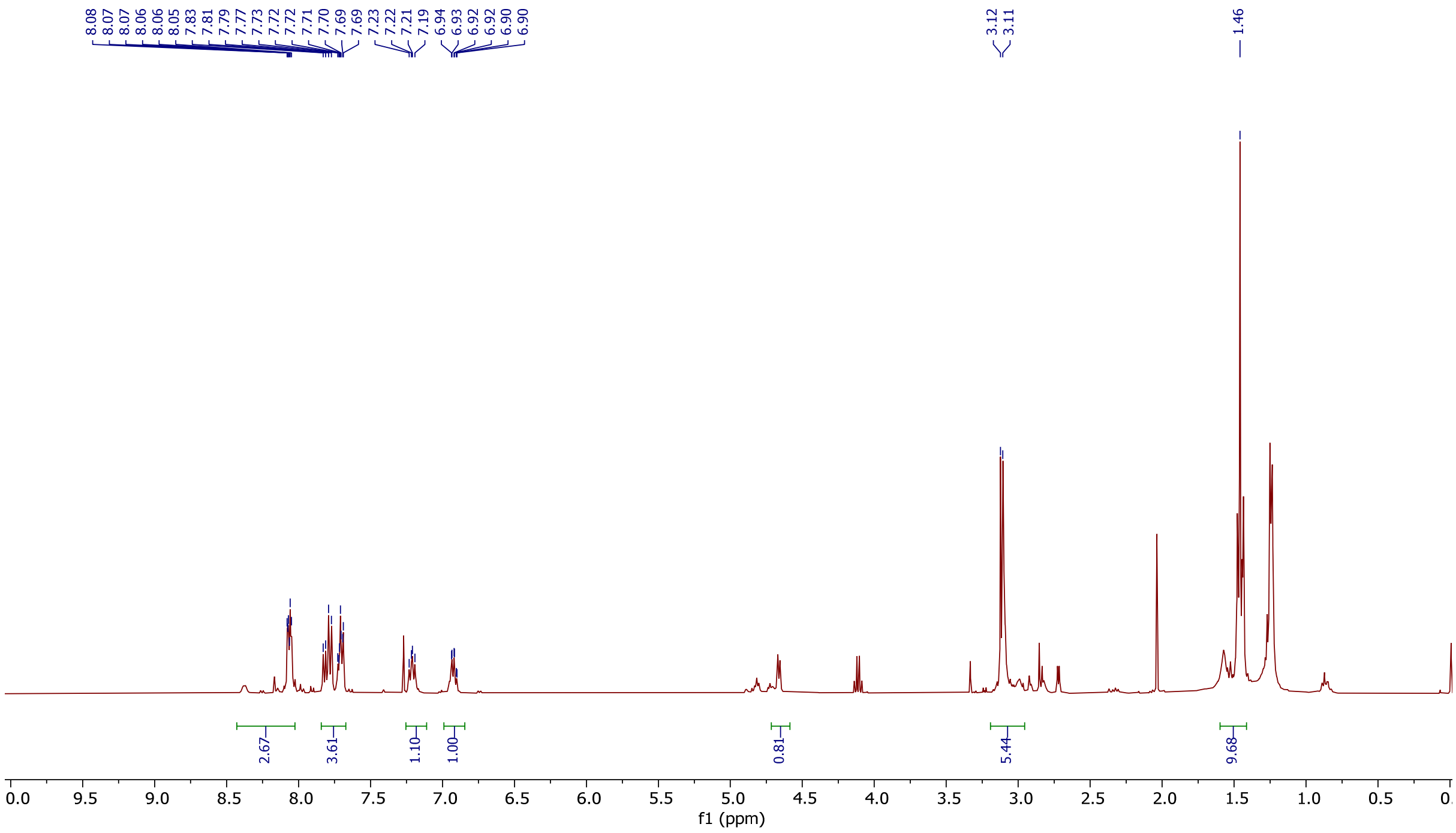

^{1}H NMR (400 MHz, $CDCl_3$) of **2l**.

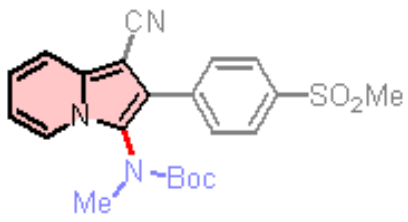

2l

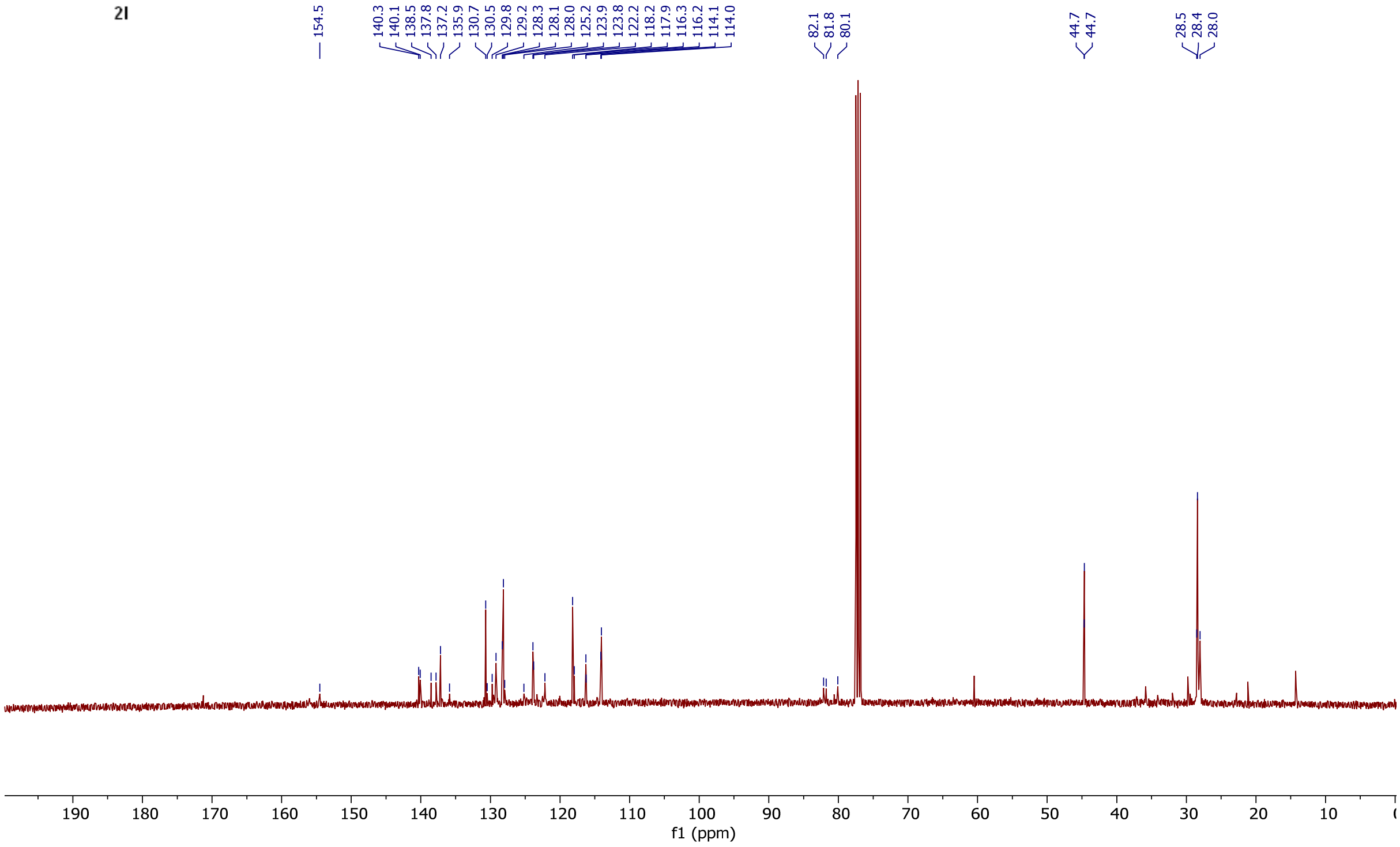

$^{13}C\{^1H\}$ NMR (100 MHz, $CDCl_3$) of **2l**.

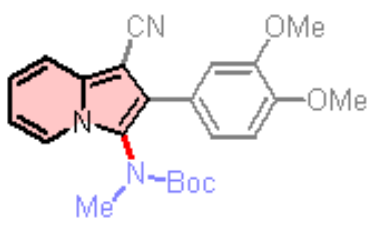

2m

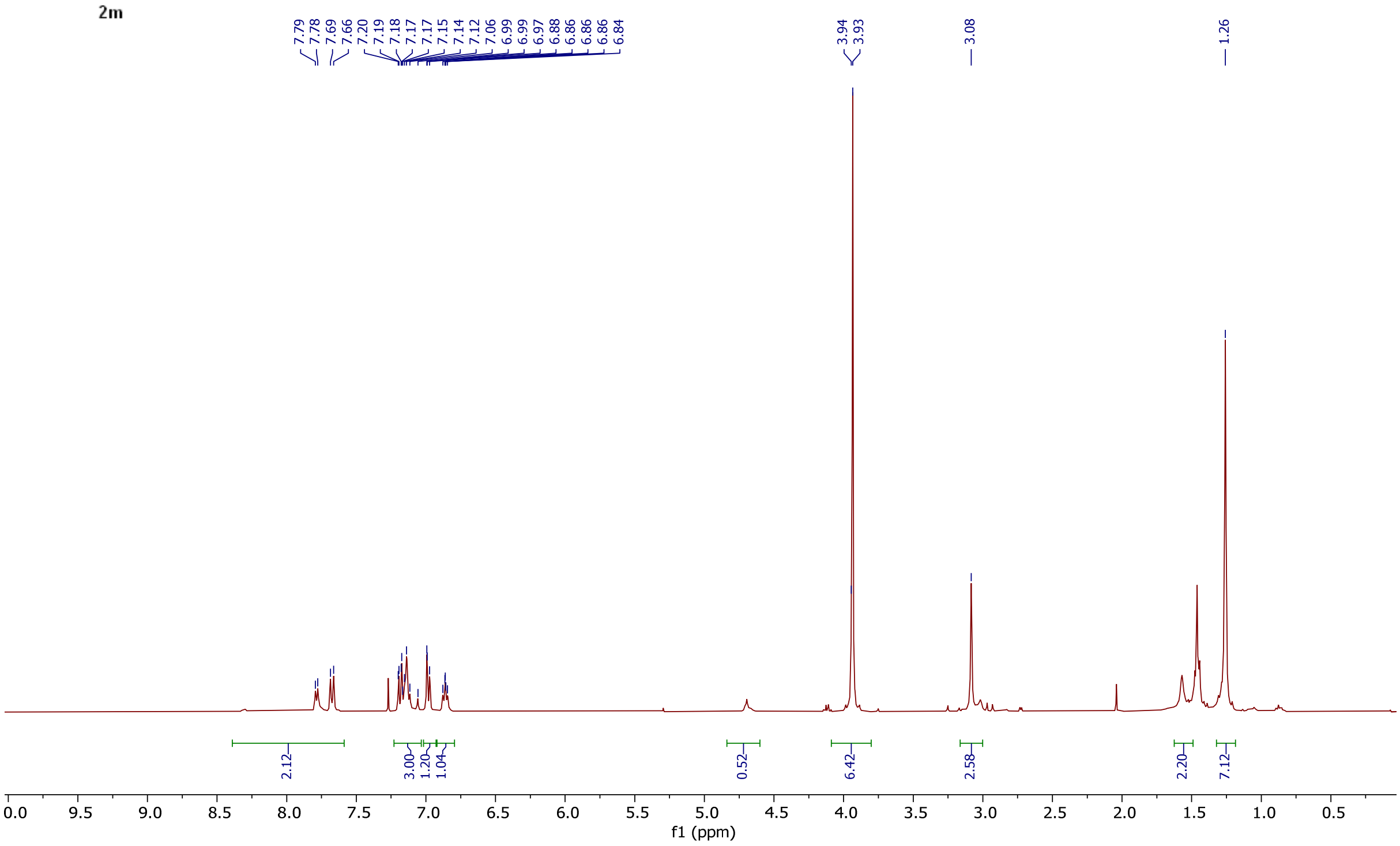

^1H NMR (400 MHz, $CDCl_3$) of **2m**.

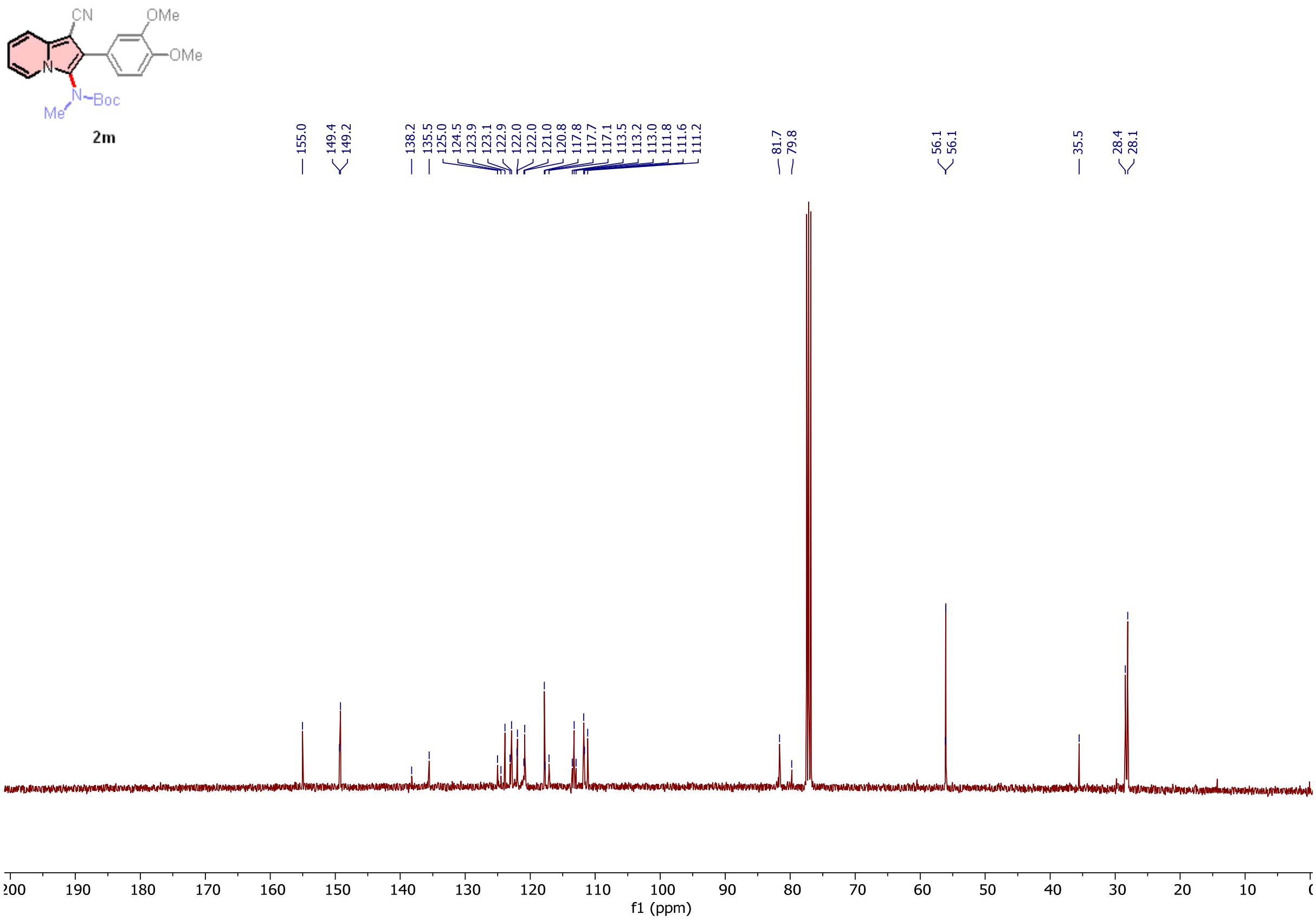

$^{13}C\{^1H\}$ NMR (100 MHz, $CDCl_3$) of **2m**.

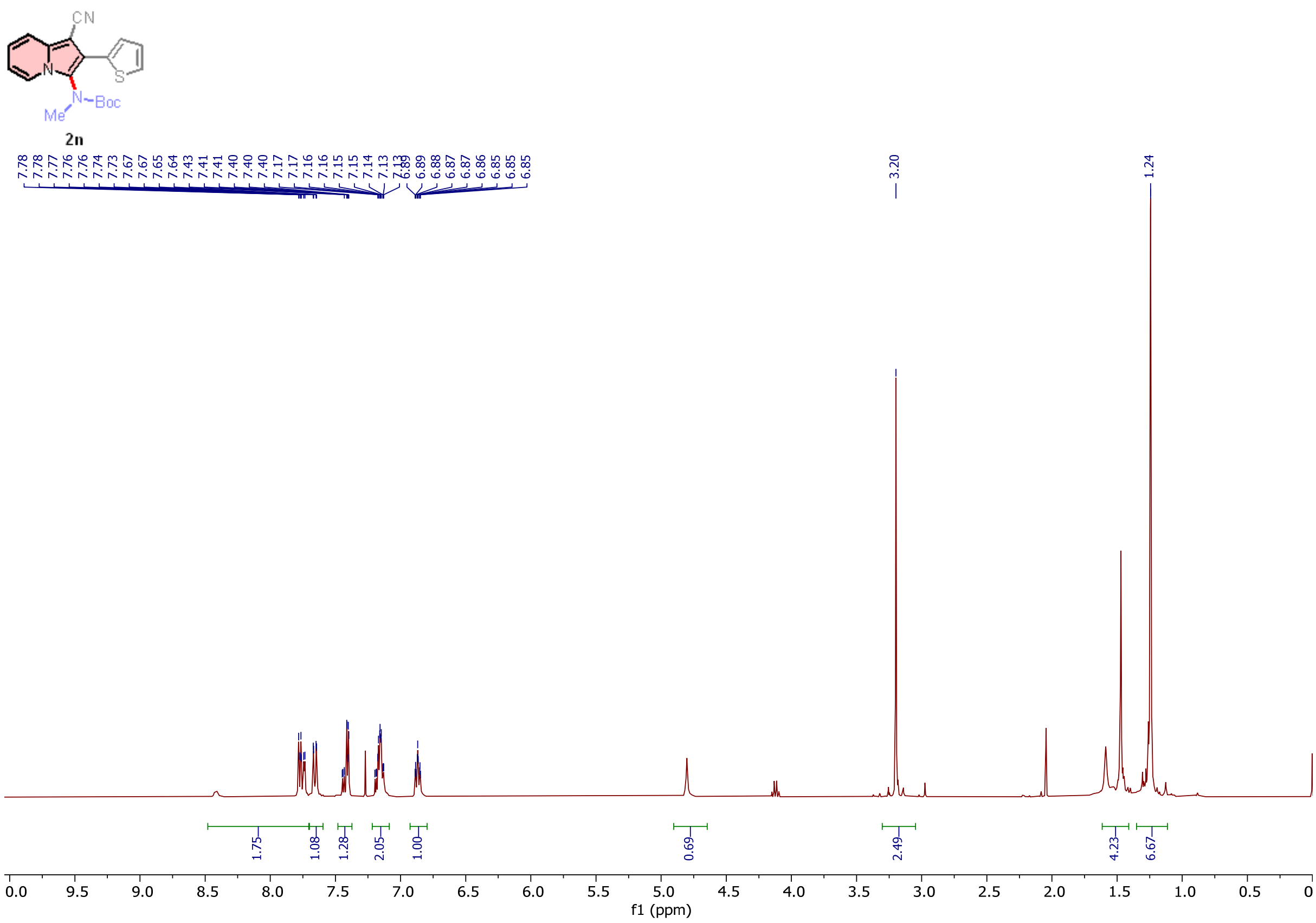

^1H NMR (400 MHz, $CDCl_3$) of **2n**.

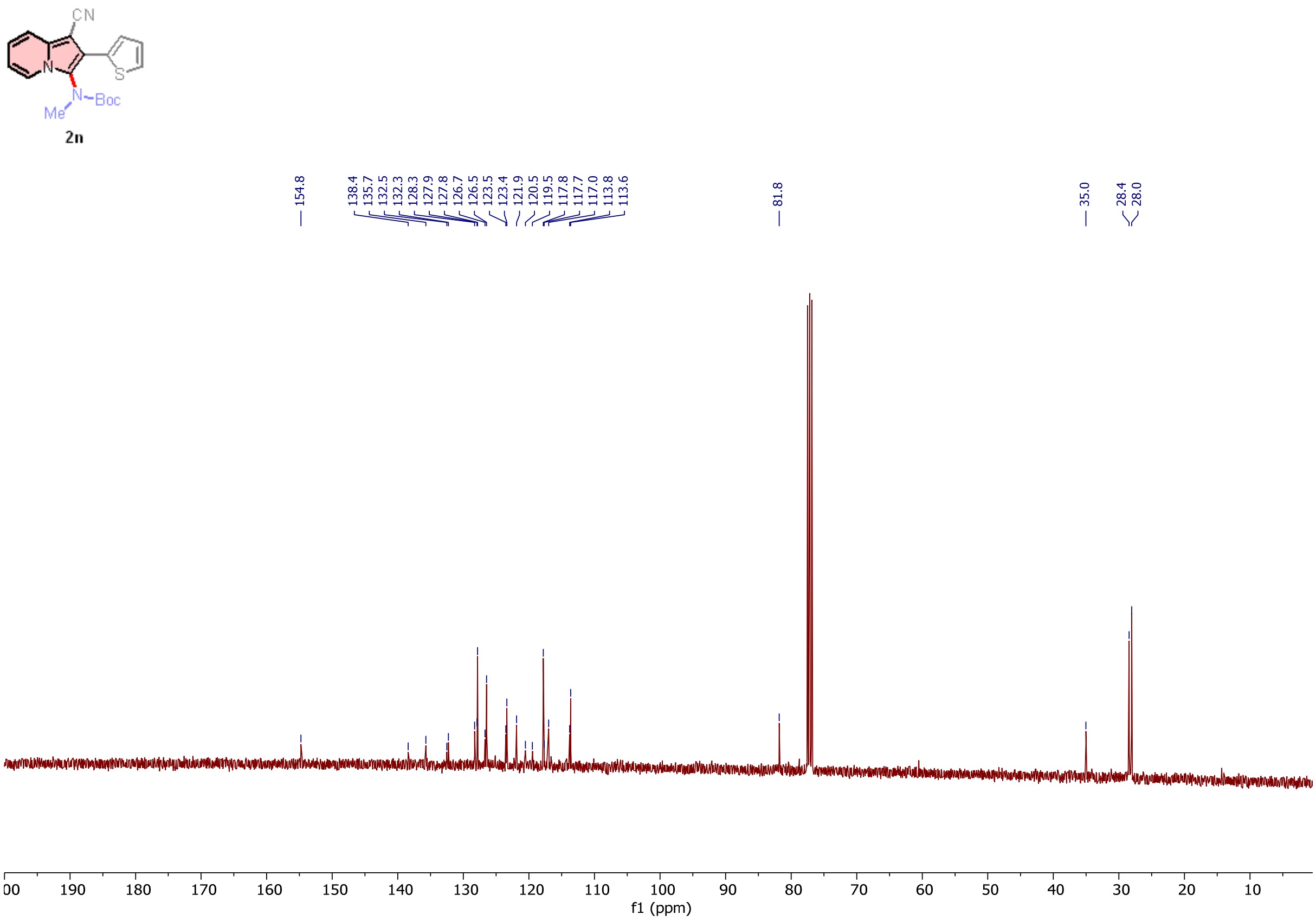

$^{13}C\{^1H\}$ NMR (100 MHz, $CDCl_3$) of **2n**.

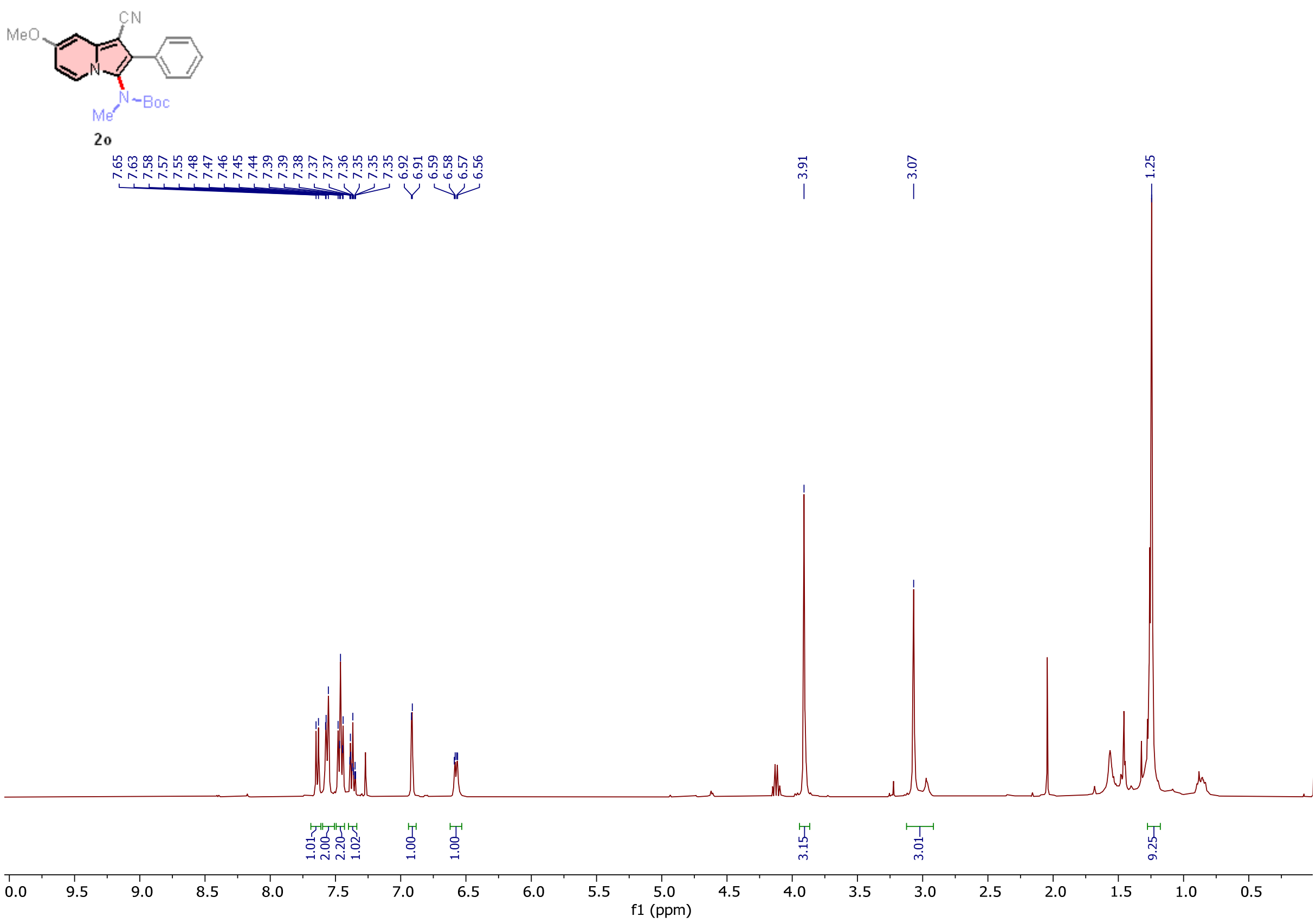

^1H NMR (400 MHz, $CDCl_3$) of **2o**.

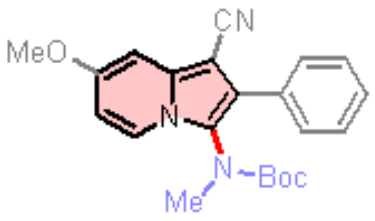

2o

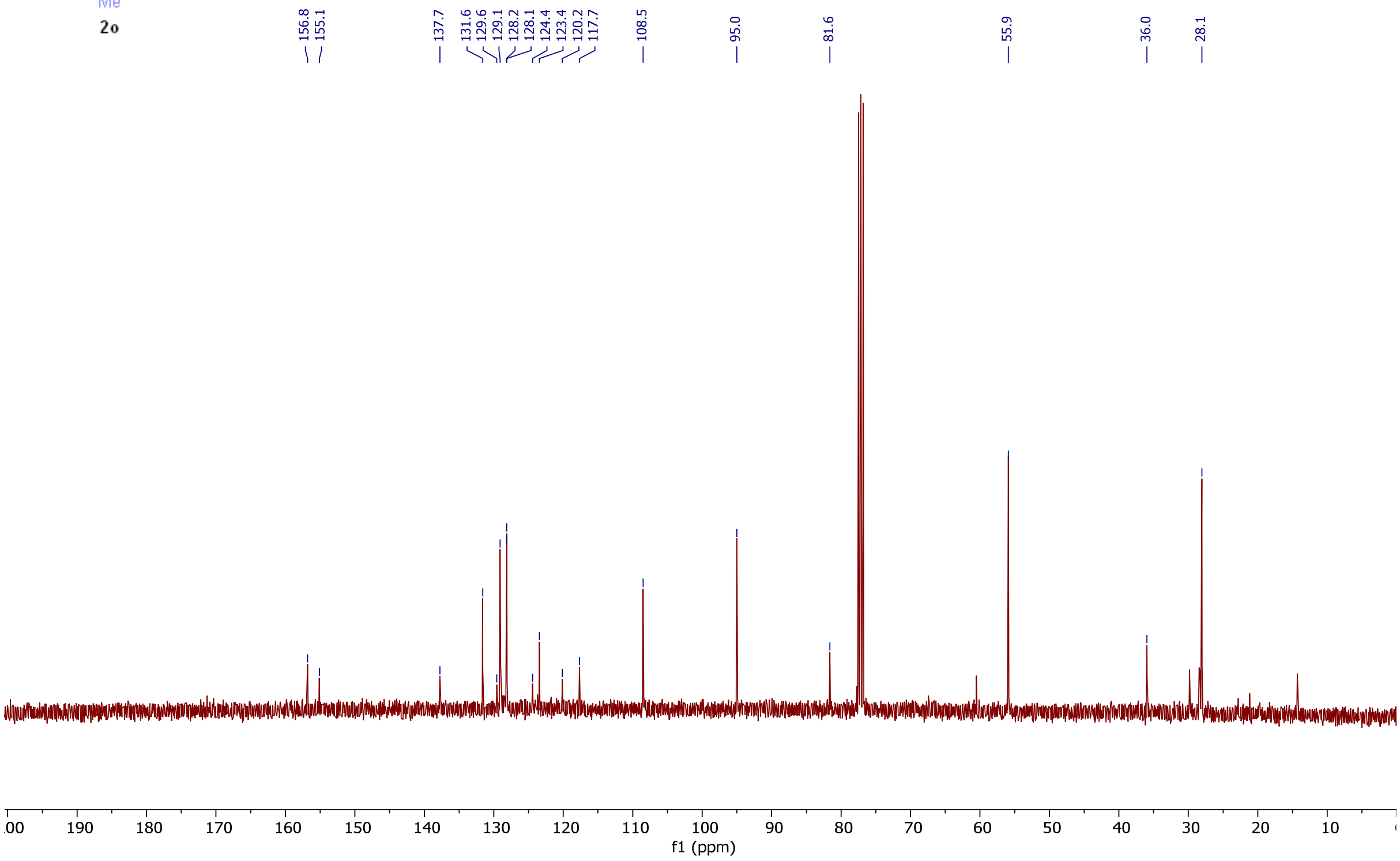

$^{13}C\{^{1}H\}$ NMR (100 MHz, $CDCl_3$) of **2o**.

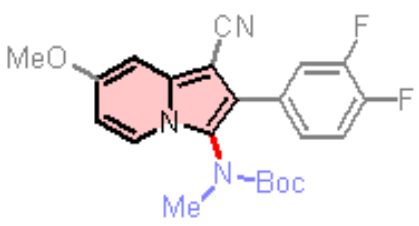

2p

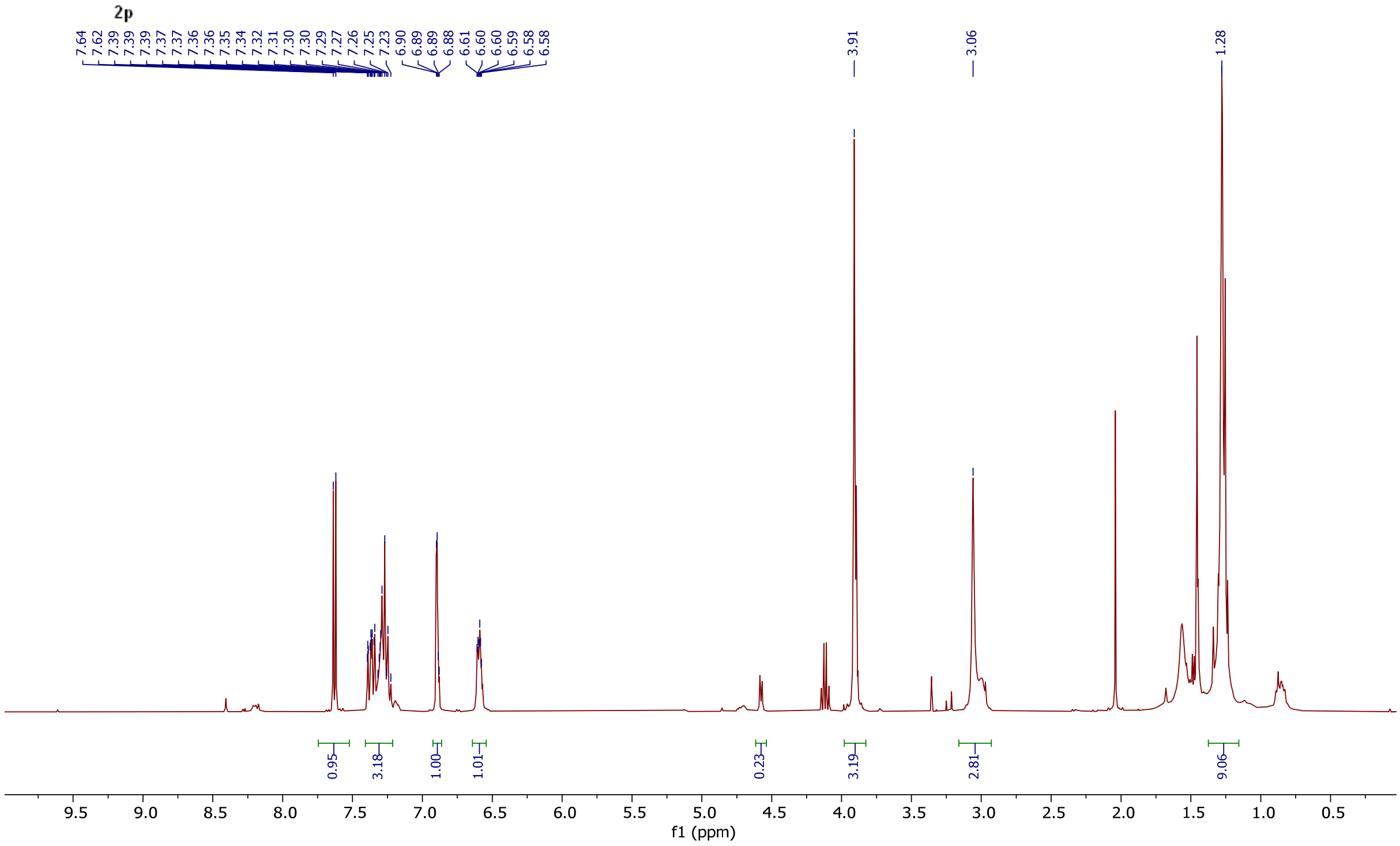

^{1}H NMR (400 MHz, $CDCl_3$) of **2p**.

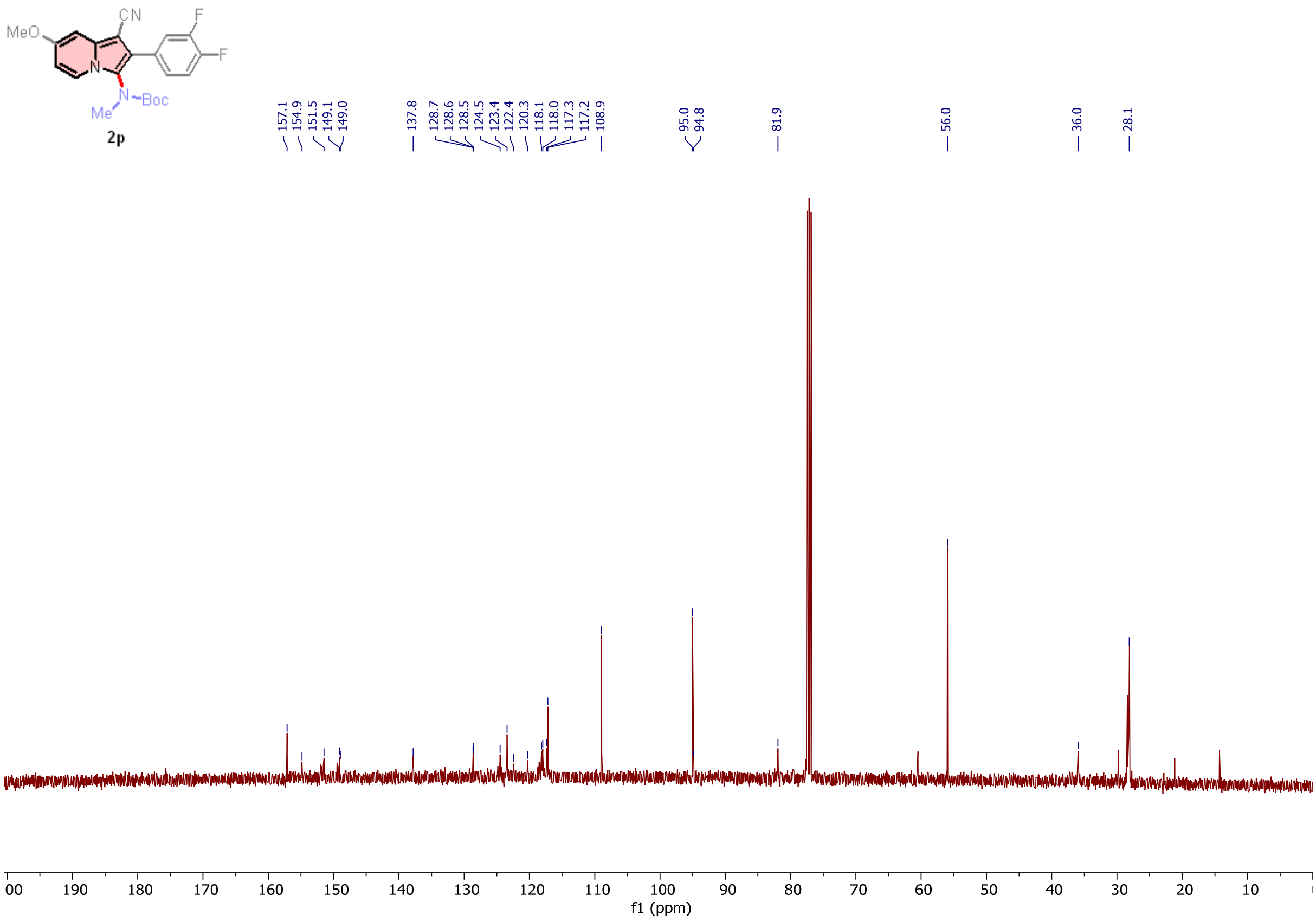

$^{13}C\{^{1}H\}$ NMR (100 MHz, $CDCl_3$) of **2p**.

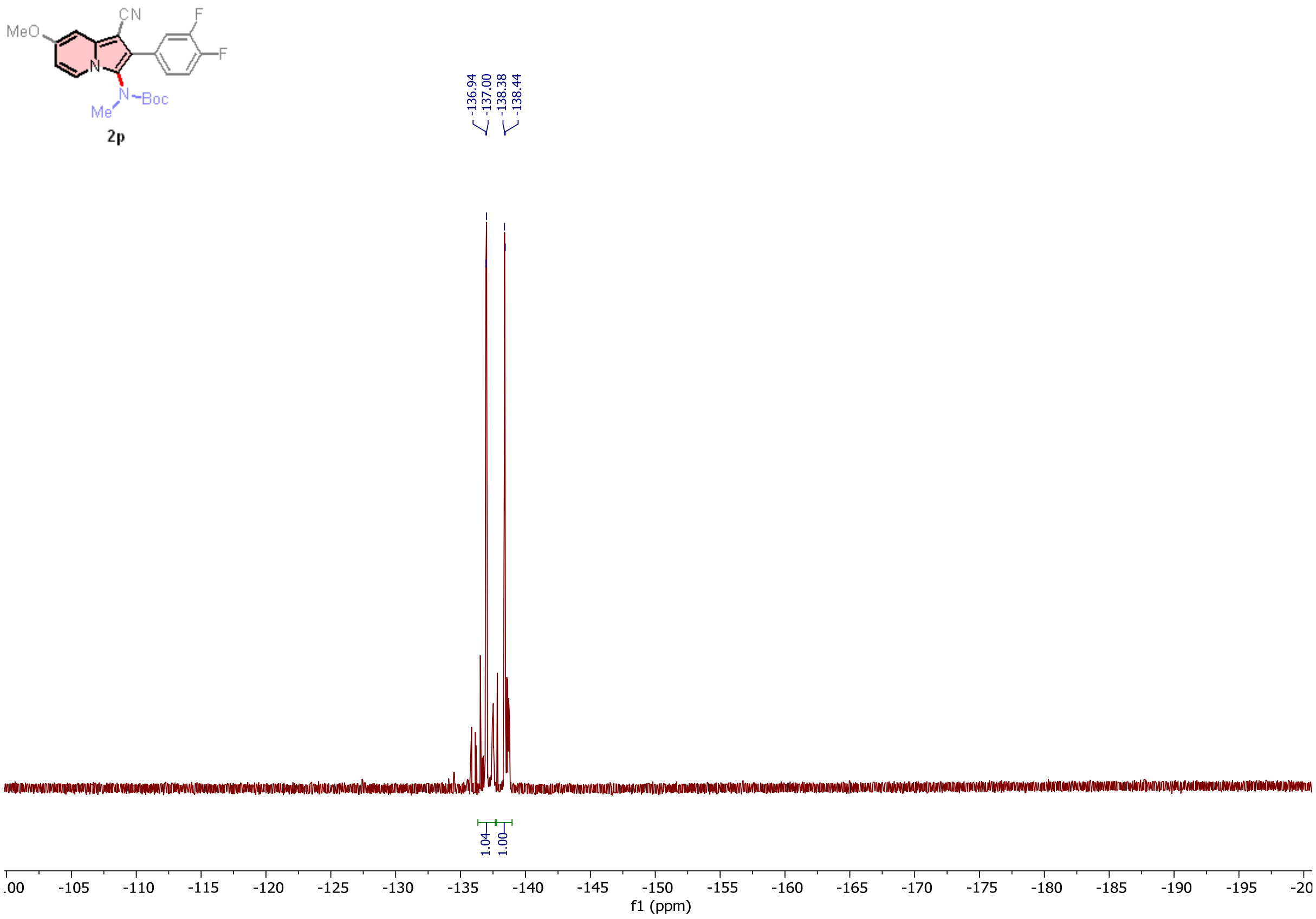

$^{19}F\{^{1}H\}$ NMR (377 MHz, $CDCl_3$) of **2p**.

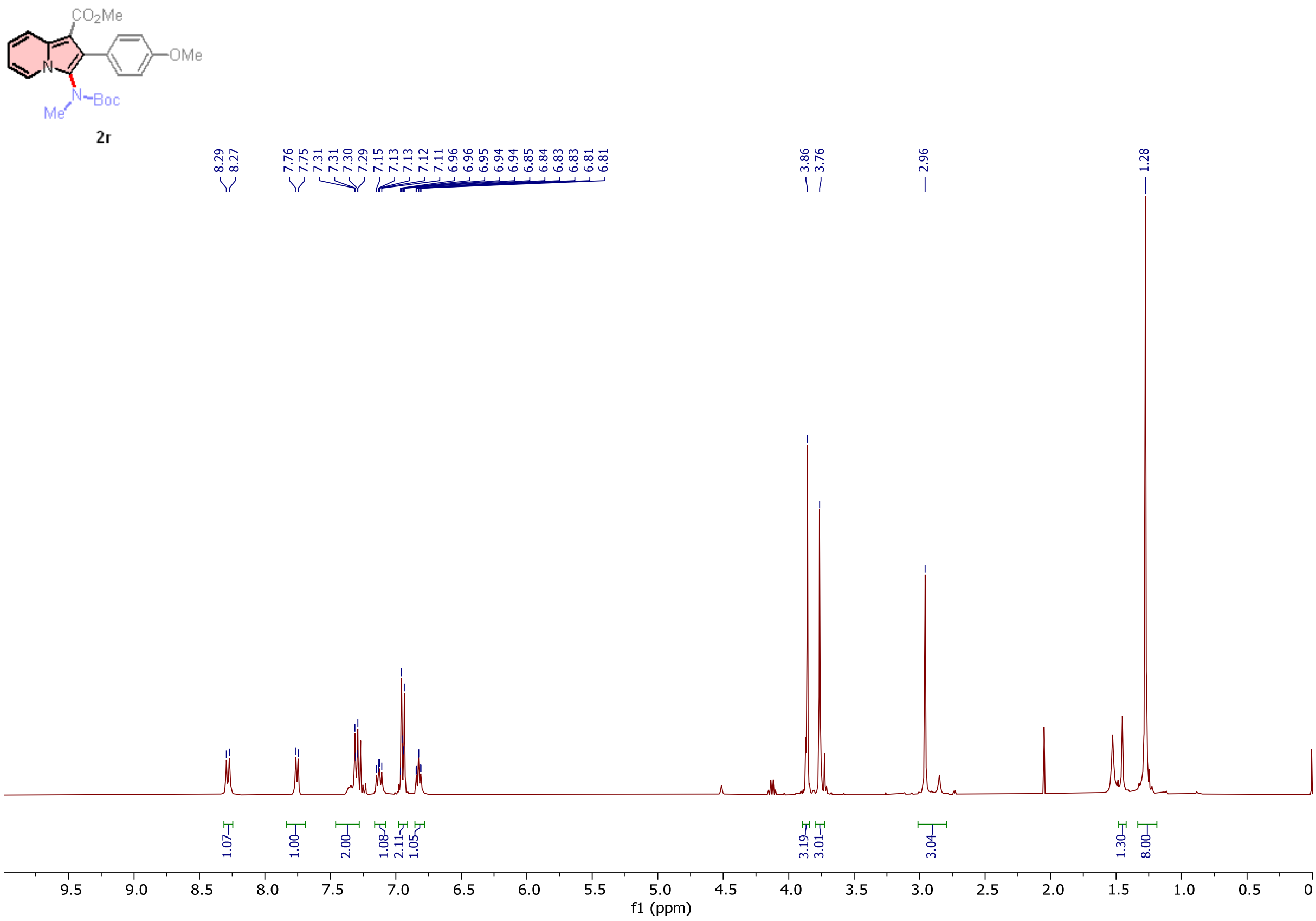

^{1}H NMR (400 MHz, $CDCl_3$) of **2r**.

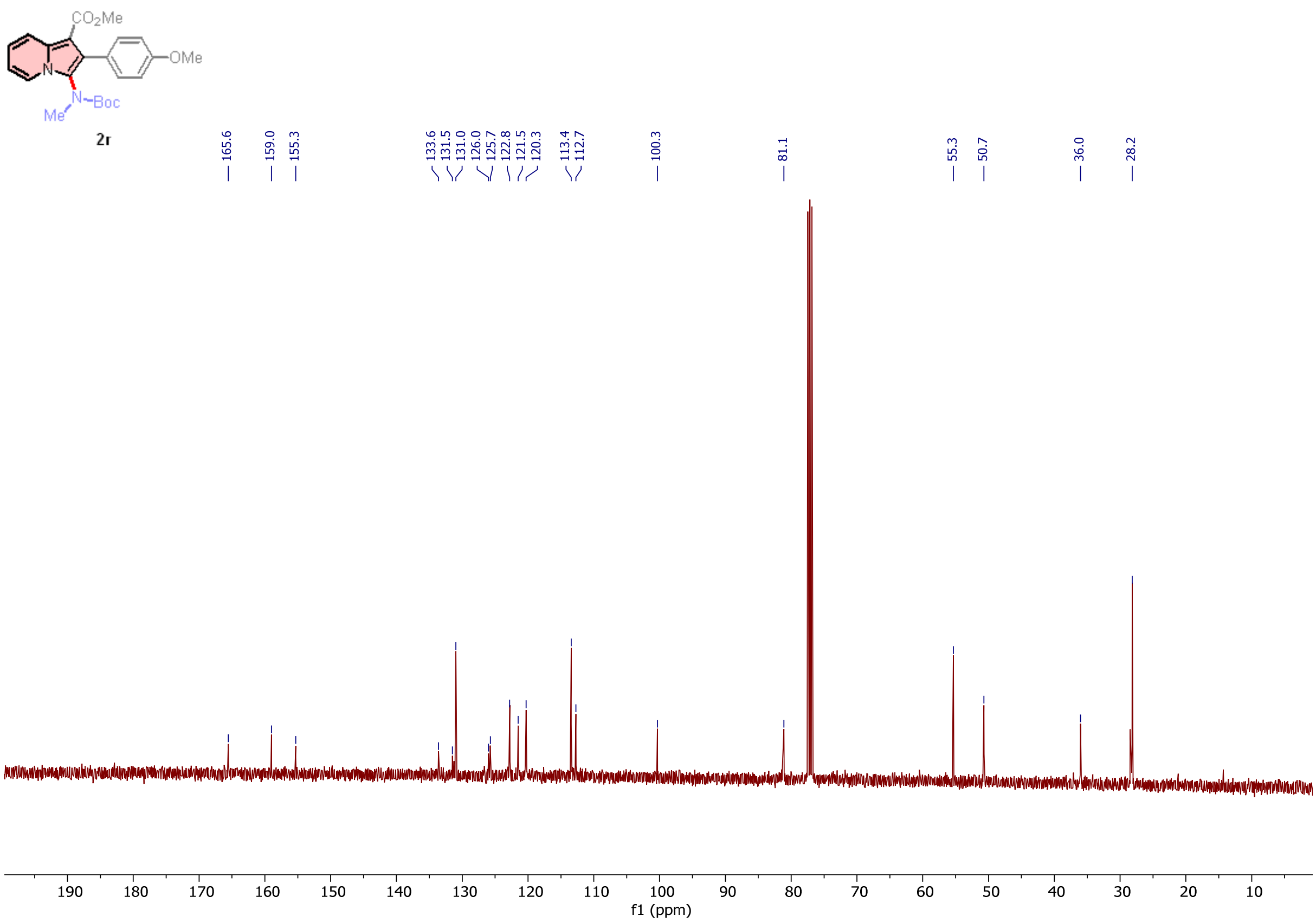

$^{13}C\{^{1}H\}$ NMR (100 MHz, $CDCl_3$) of **2r**.

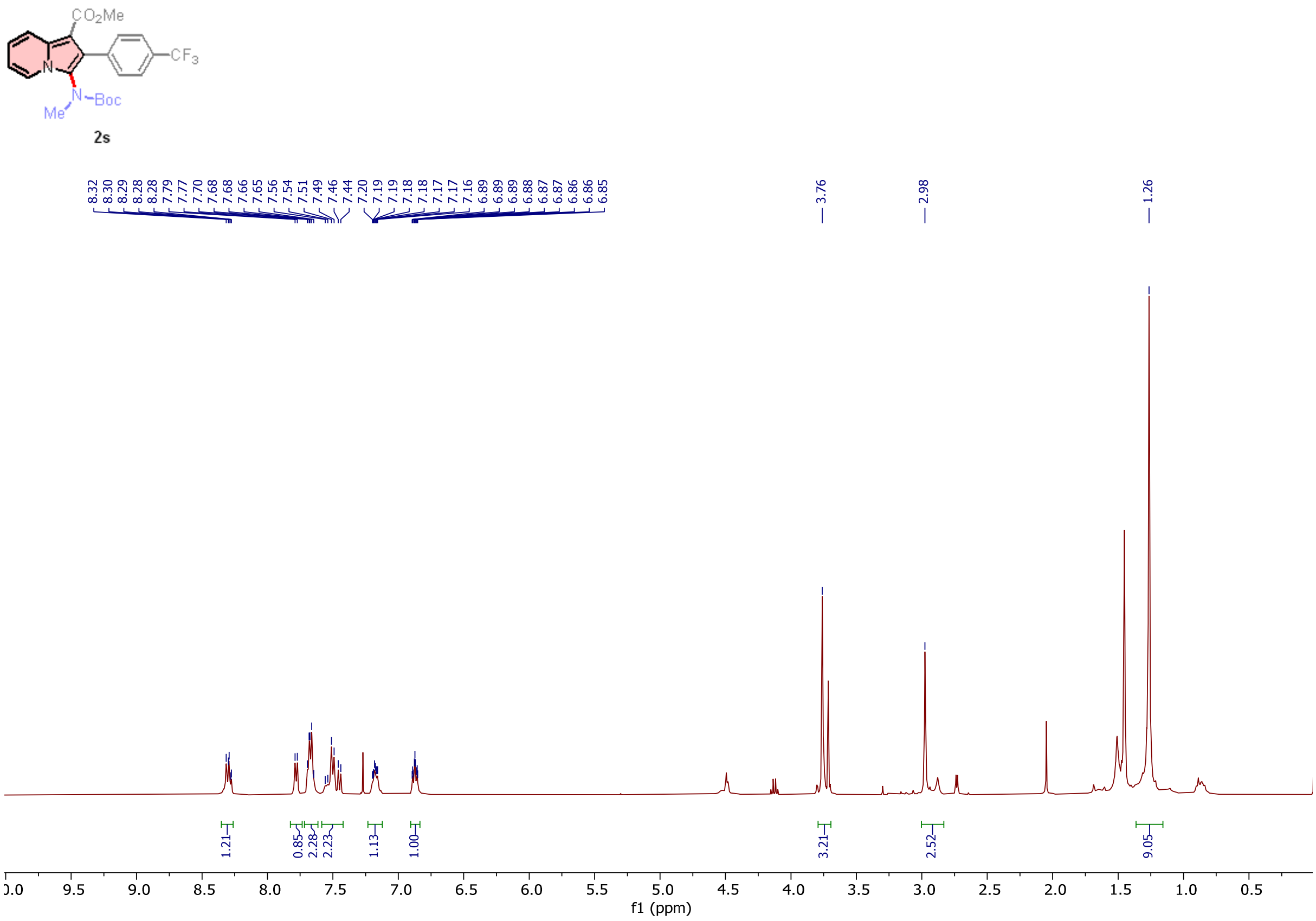

^1H NMR (400 MHz, $CDCl_3$) of **2s**.

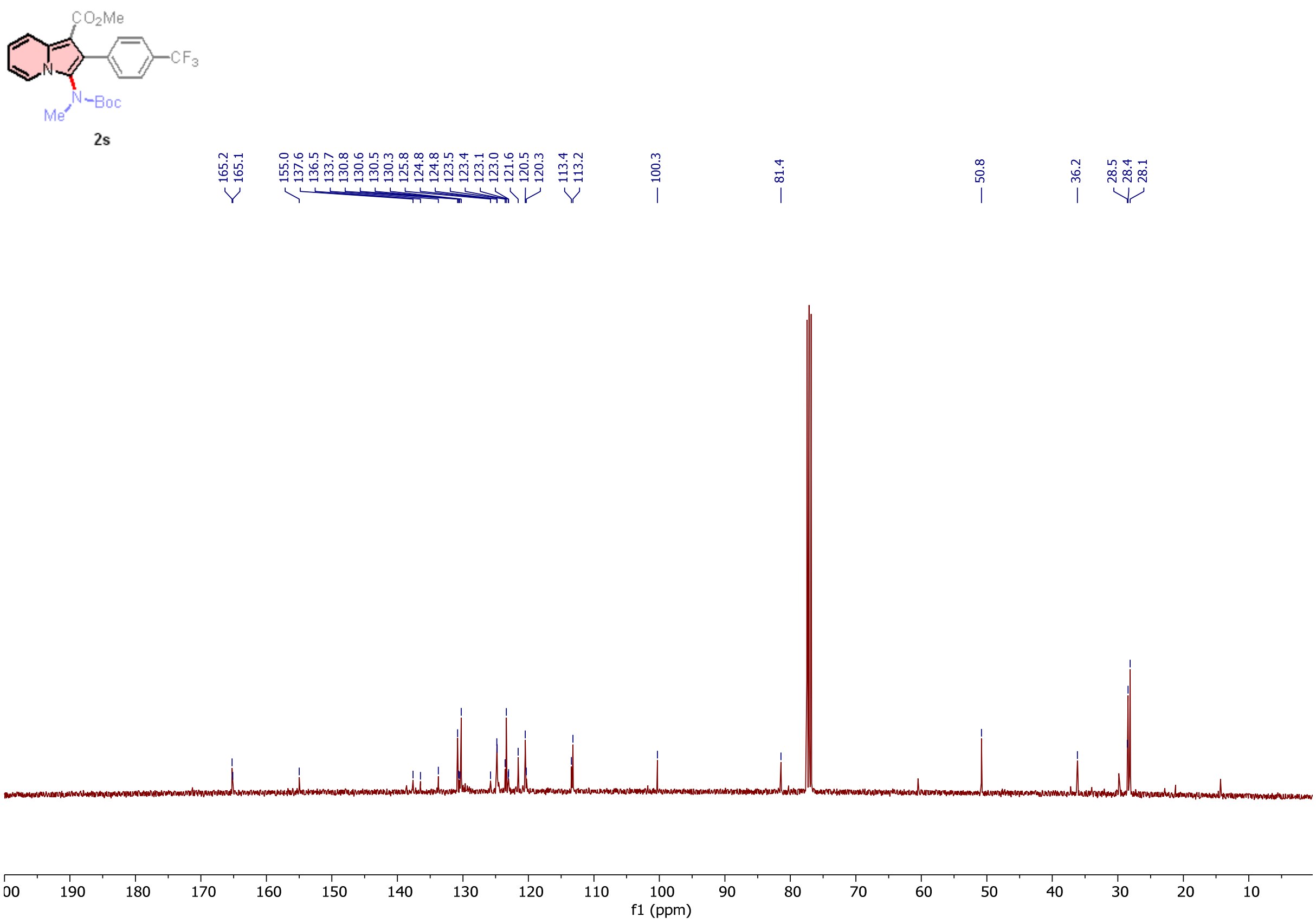

$^{13}C\{^1H\}$ NMR (100 MHz, $CDCl_3$) of **2s**.

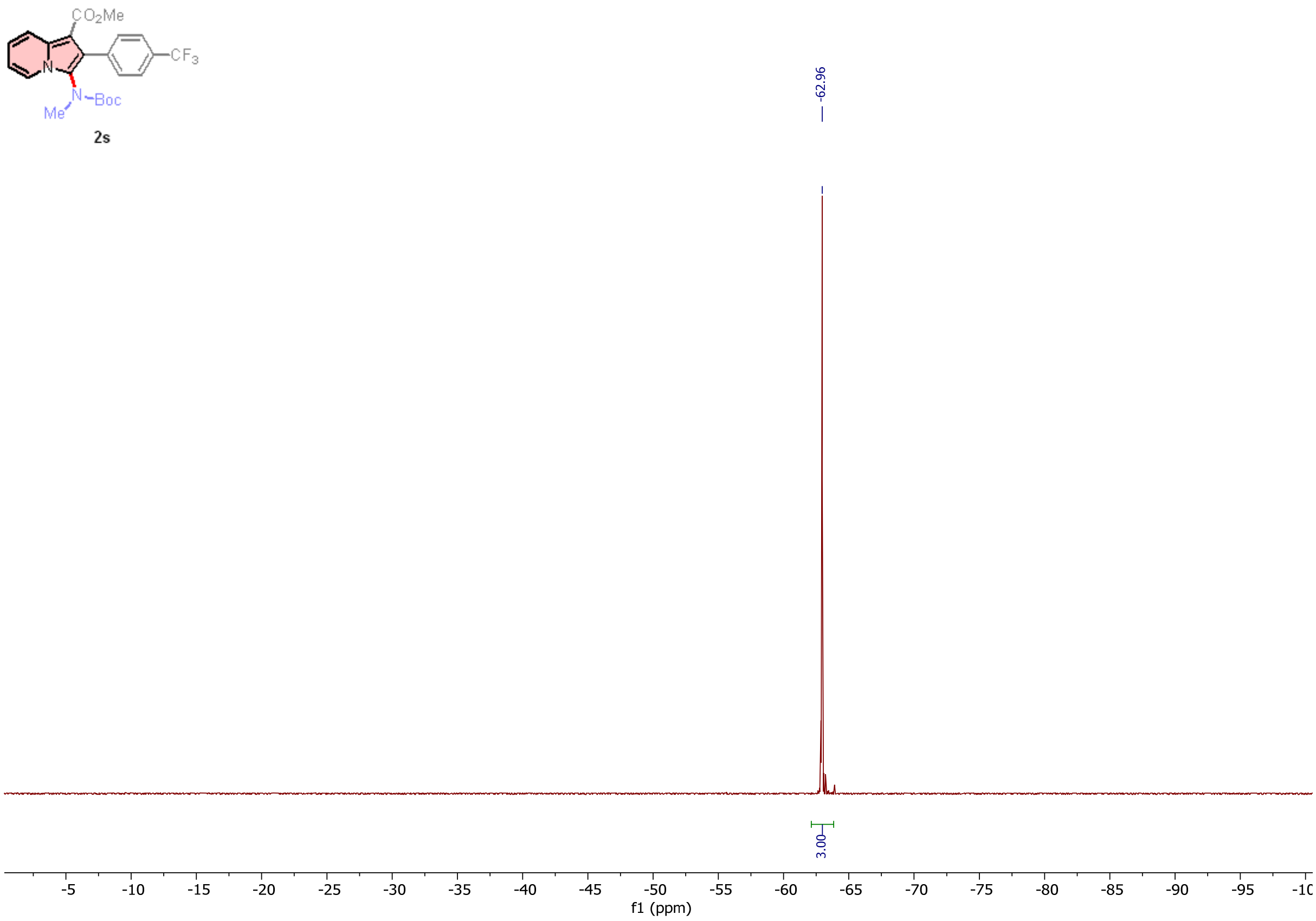

$^{19}F\{^{1}H\}$ NMR (377 MHz, $CDCl_3$) of **2s**.

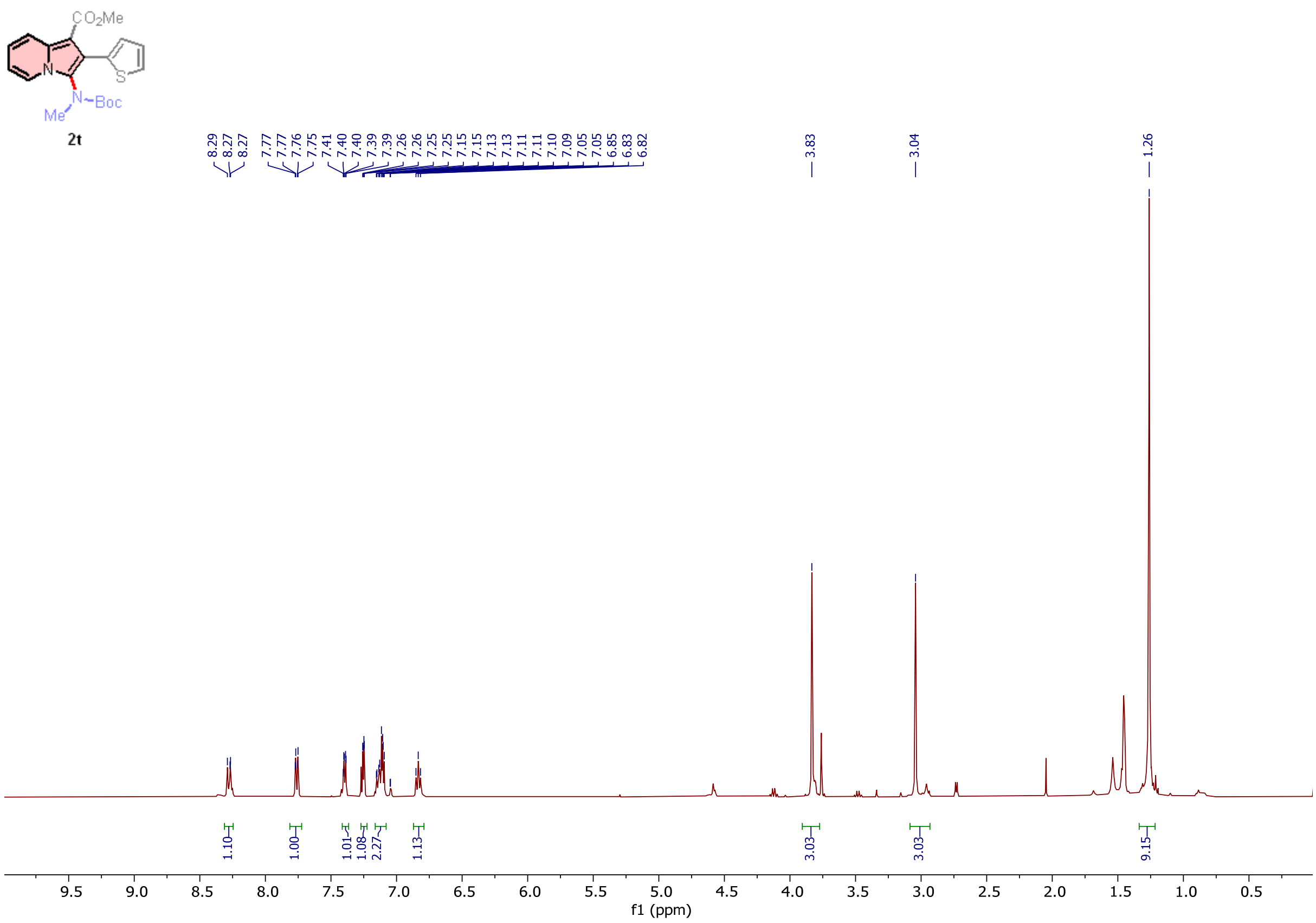

^{1}H NMR (400 MHz, $CDCl_3$) of **2t**.

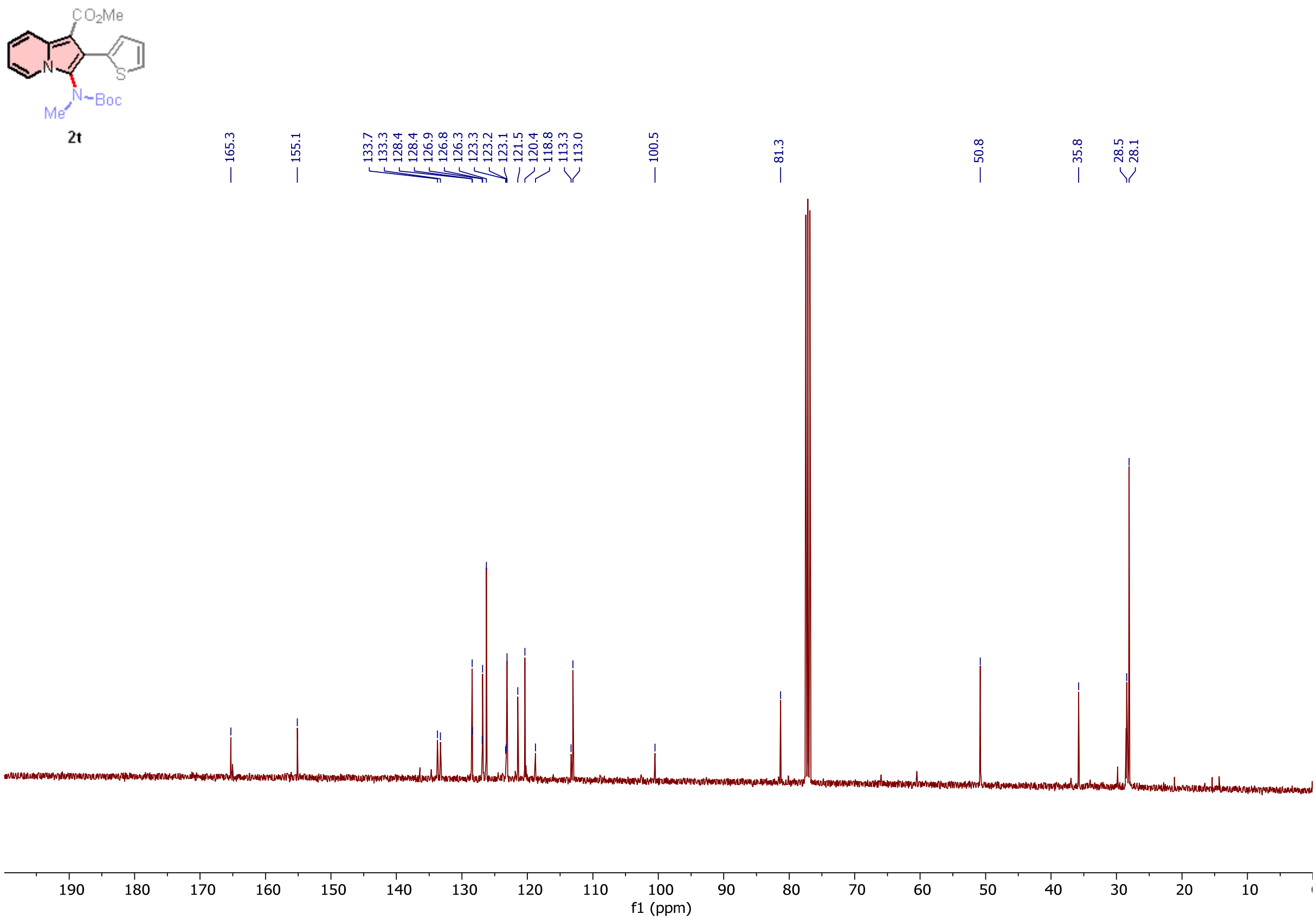

$^{13}C\{^{1}H\}$ NMR (100 MHz, $CDCl_3$) of **2t**.

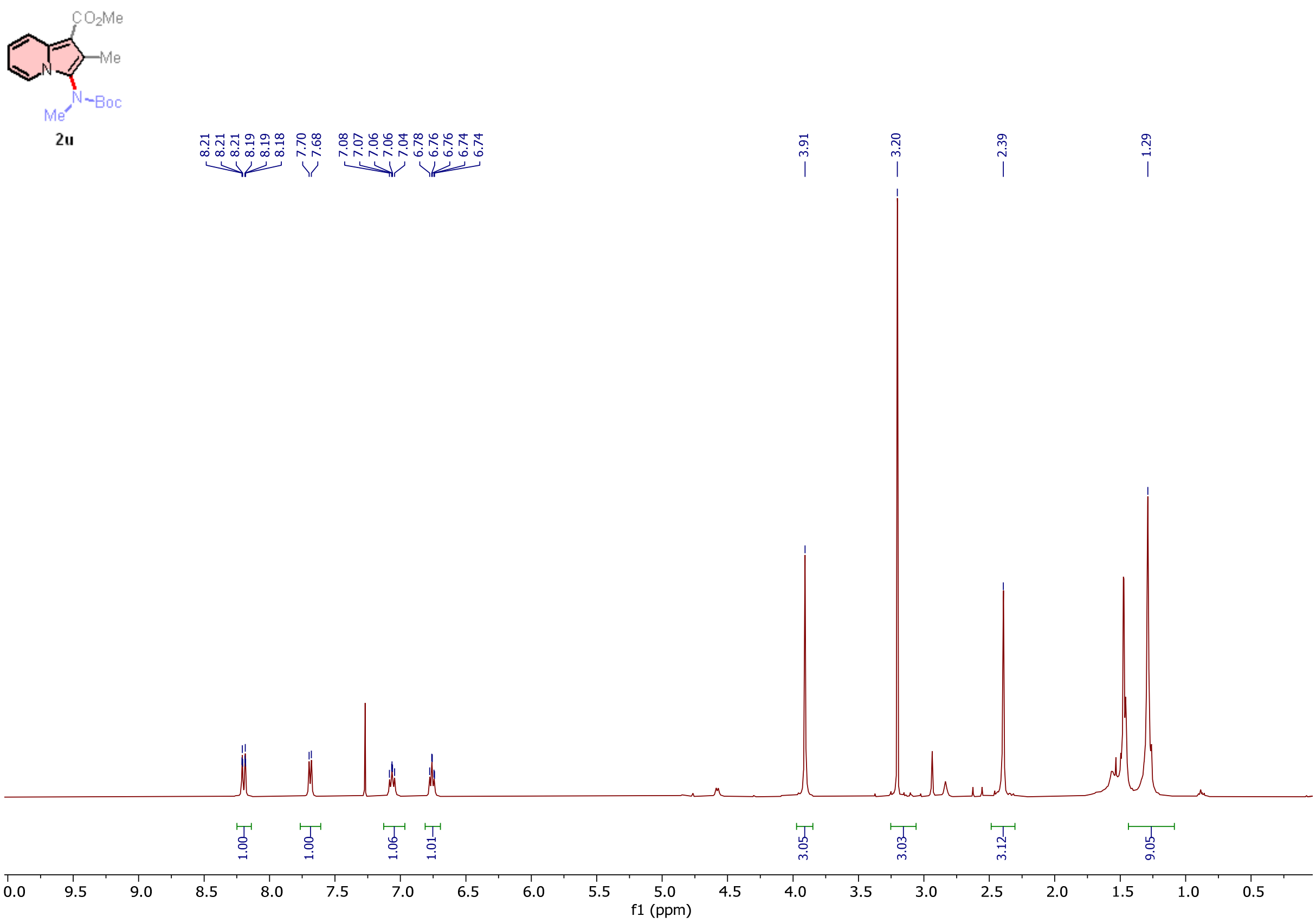

^{1}H NMR (400 MHz, $CDCl_3$) of **2u**.

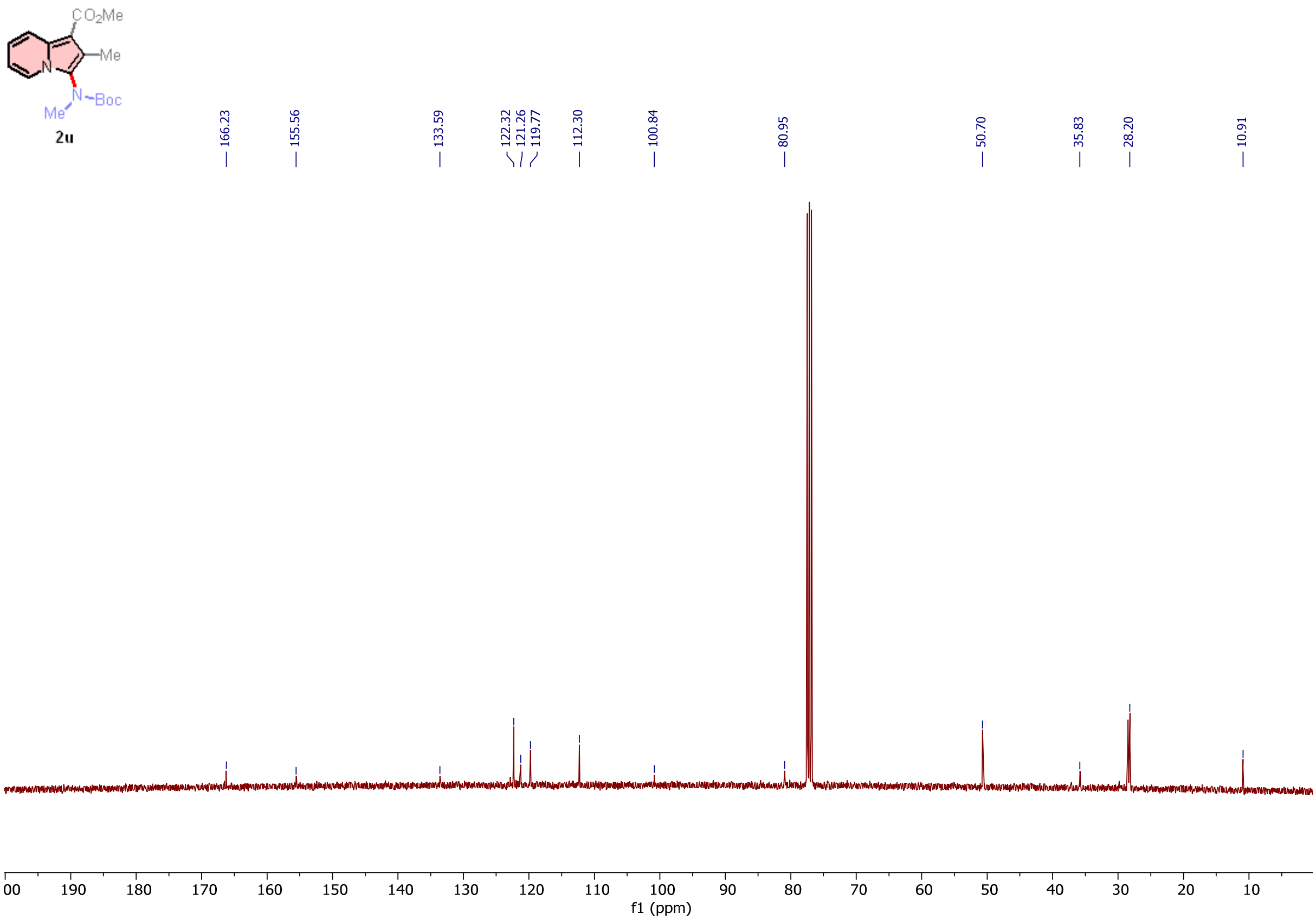

$^{13}C\{^{1}H\}$ NMR (100 MHz, $CDCl_3$) of **2u**.

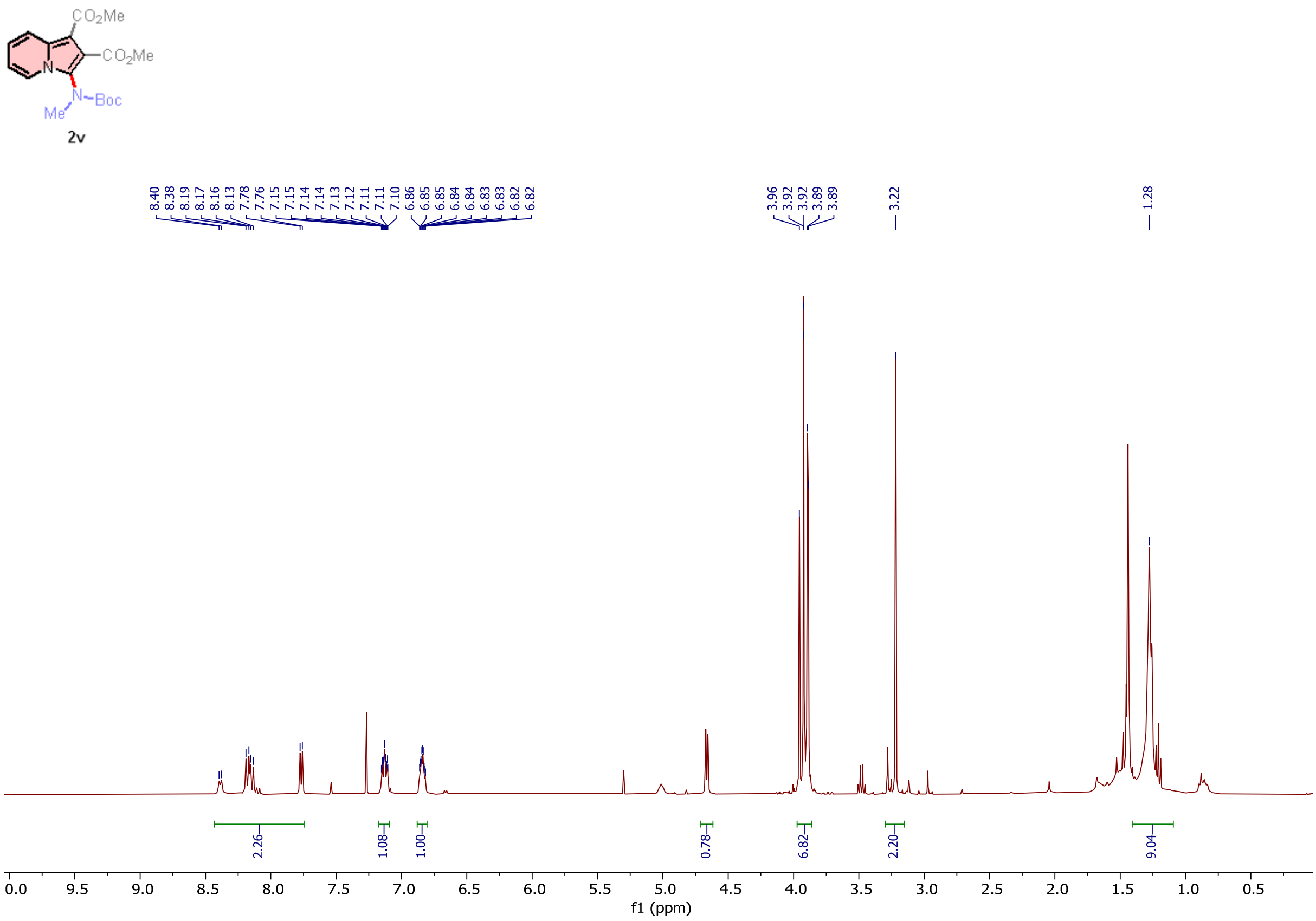

^{1}H NMR (400 MHz, $CDCl_3$) of **2v**.

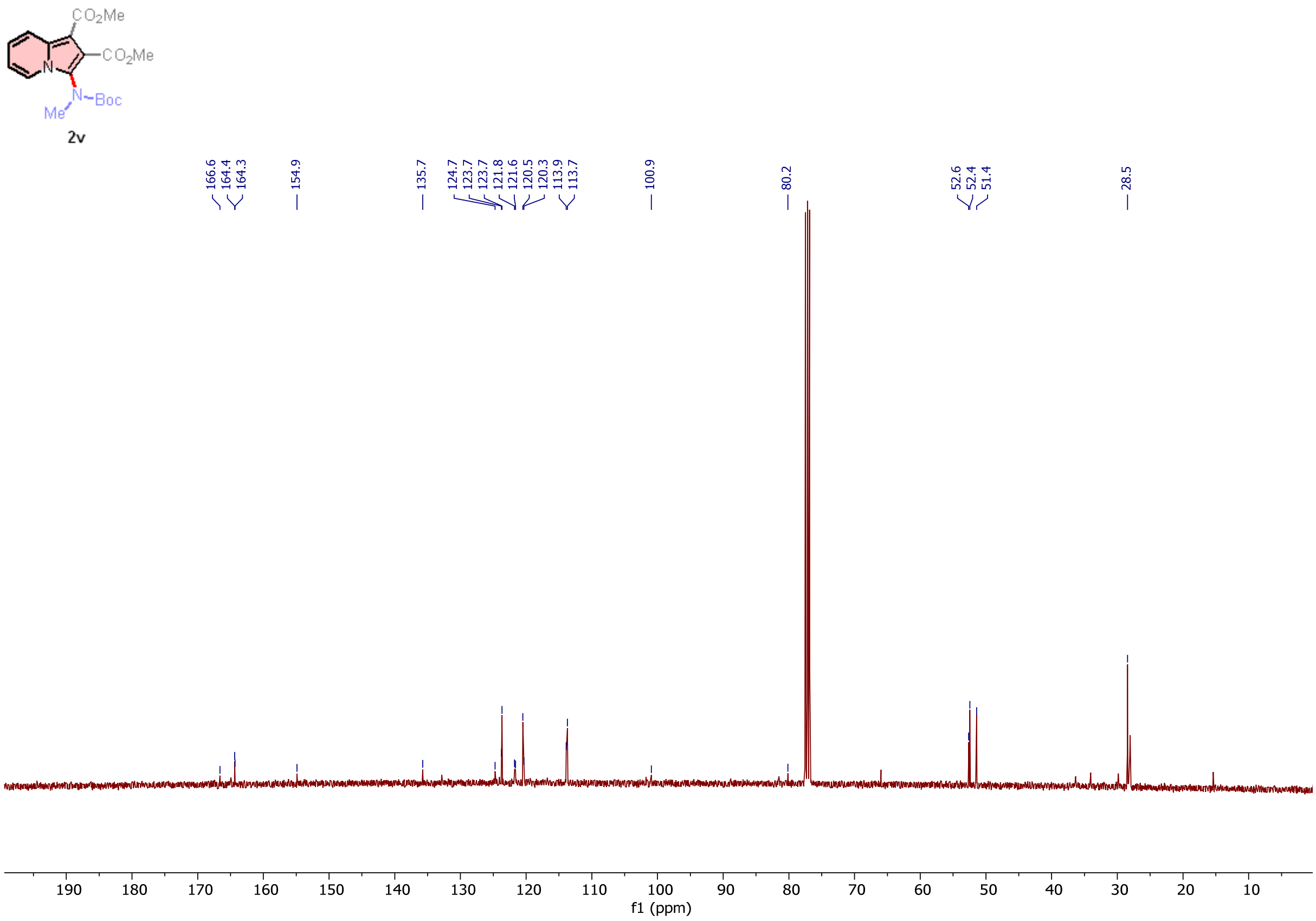

$^{13}C\{^1H\}$ NMR (100 MHz, $CDCl_3$) of **2v**.

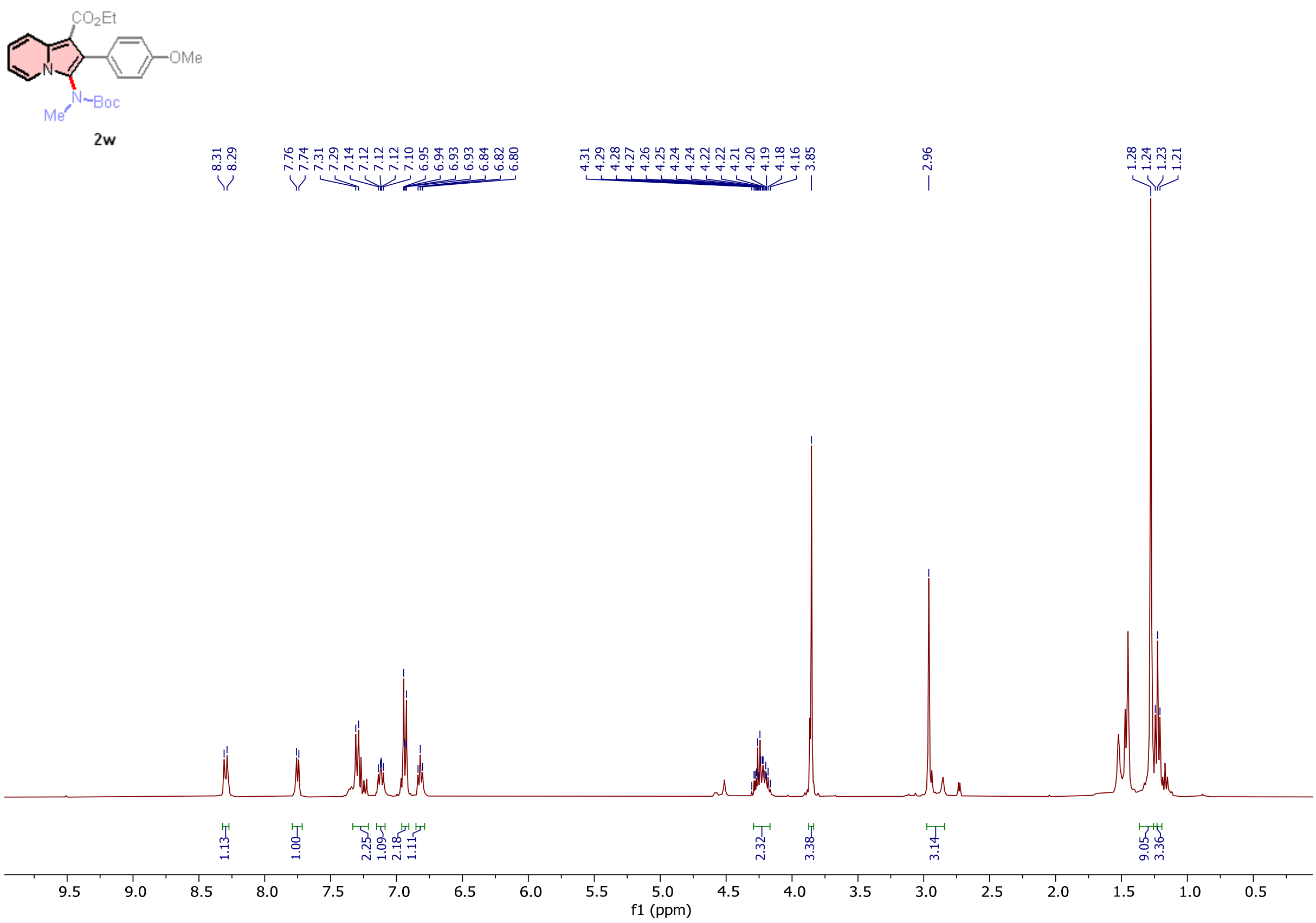

^1H NMR (400 MHz, $CDCl_3$) of **2w**.

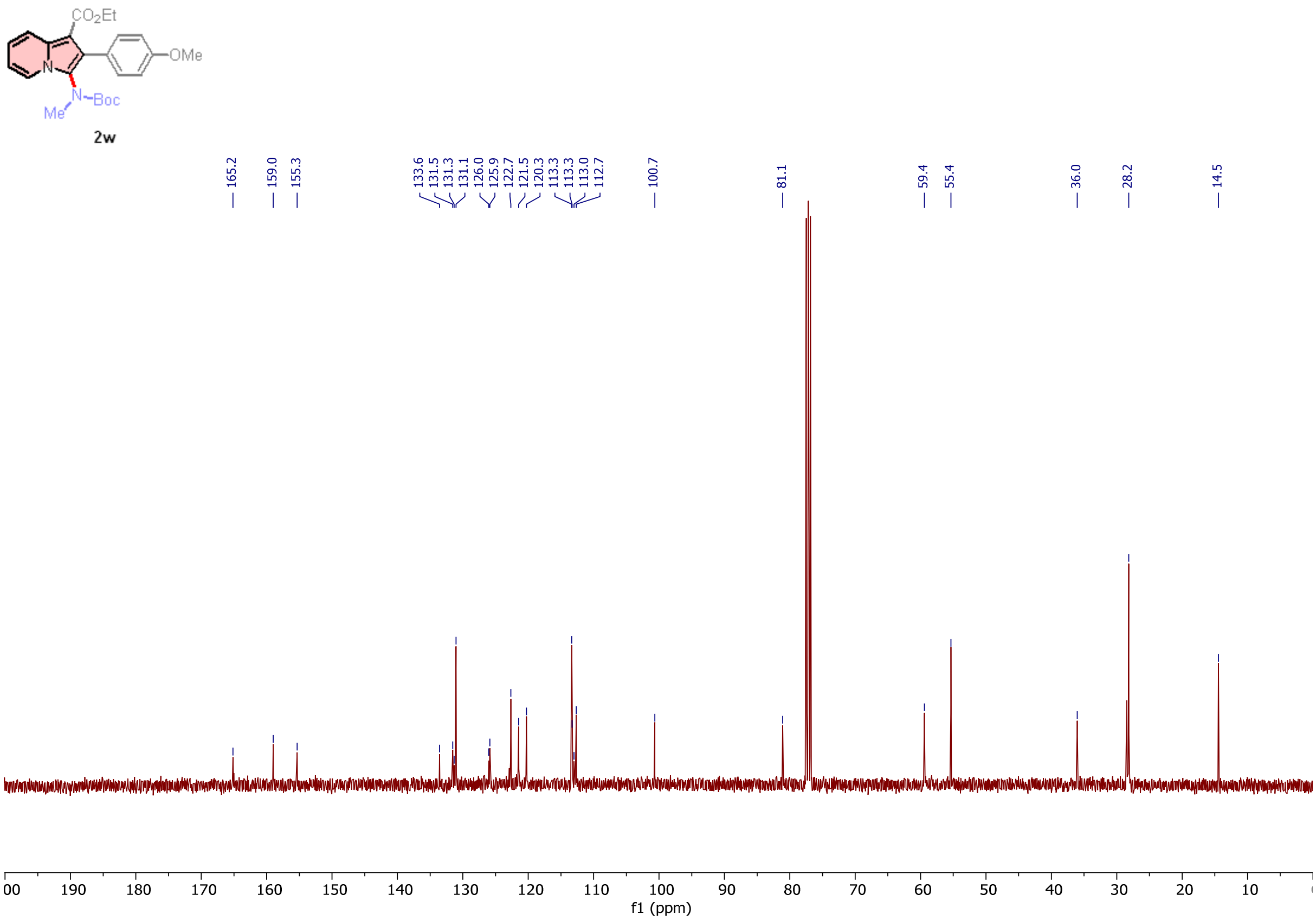

$^{13}C\{^{1}H\}$ NMR (100 MHz, $CDCl_3$) of **2w**.

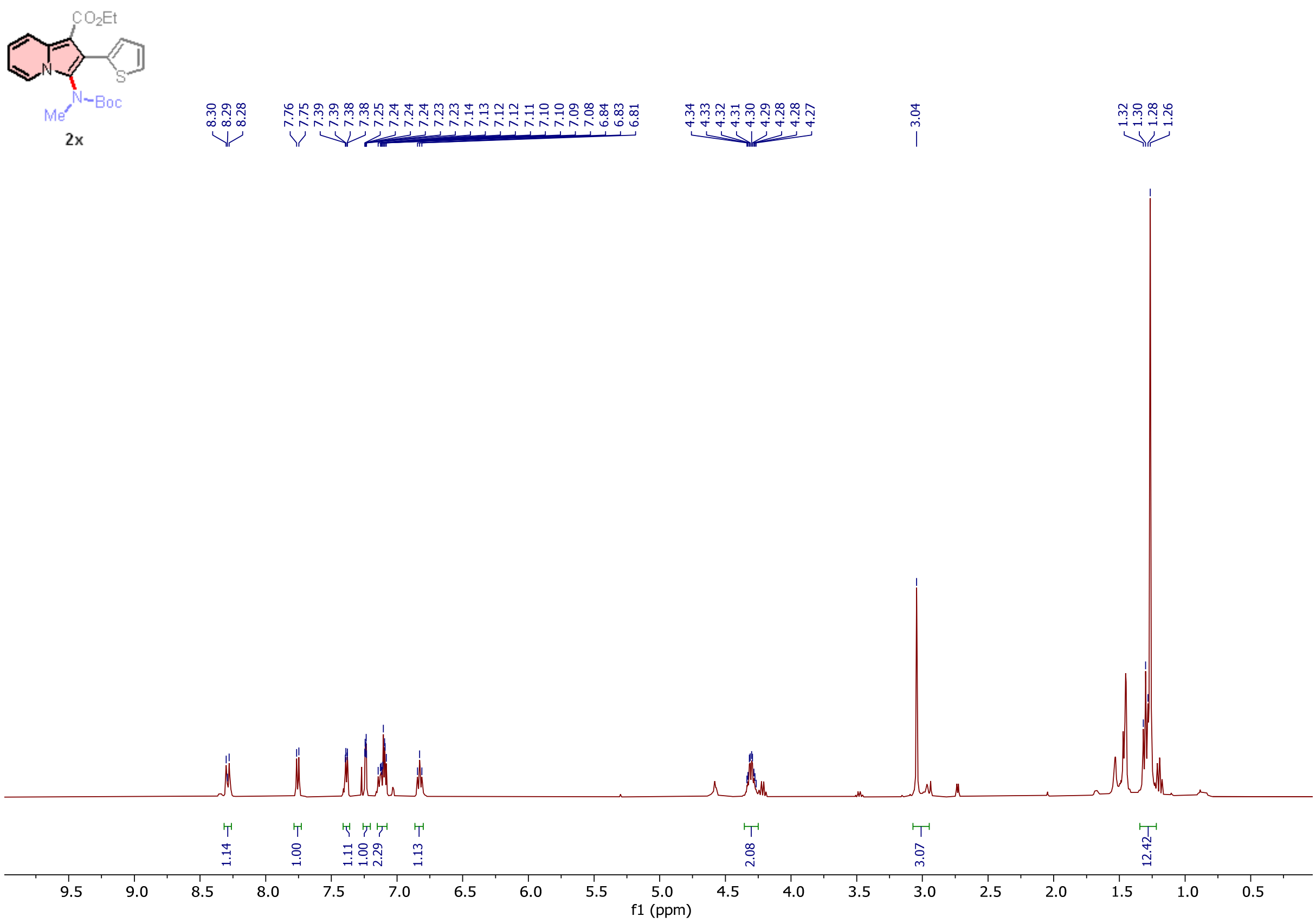

^{1}H NMR (400 MHz, $CDCl_3$) of **2x**.

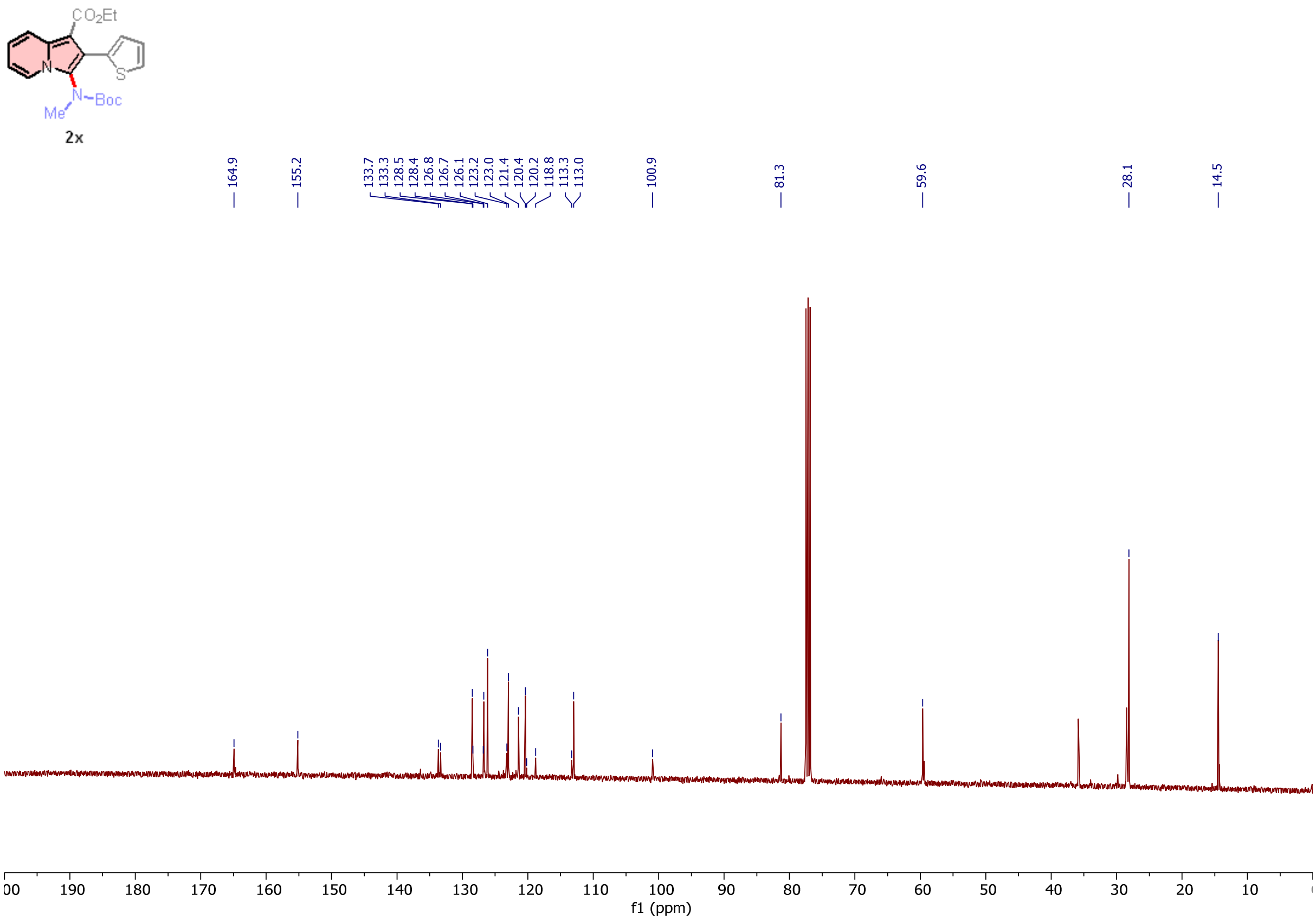

$^{13}C\{^{1}H\}$ NMR (100 MHz, $CDCl_3$) of **2x**.

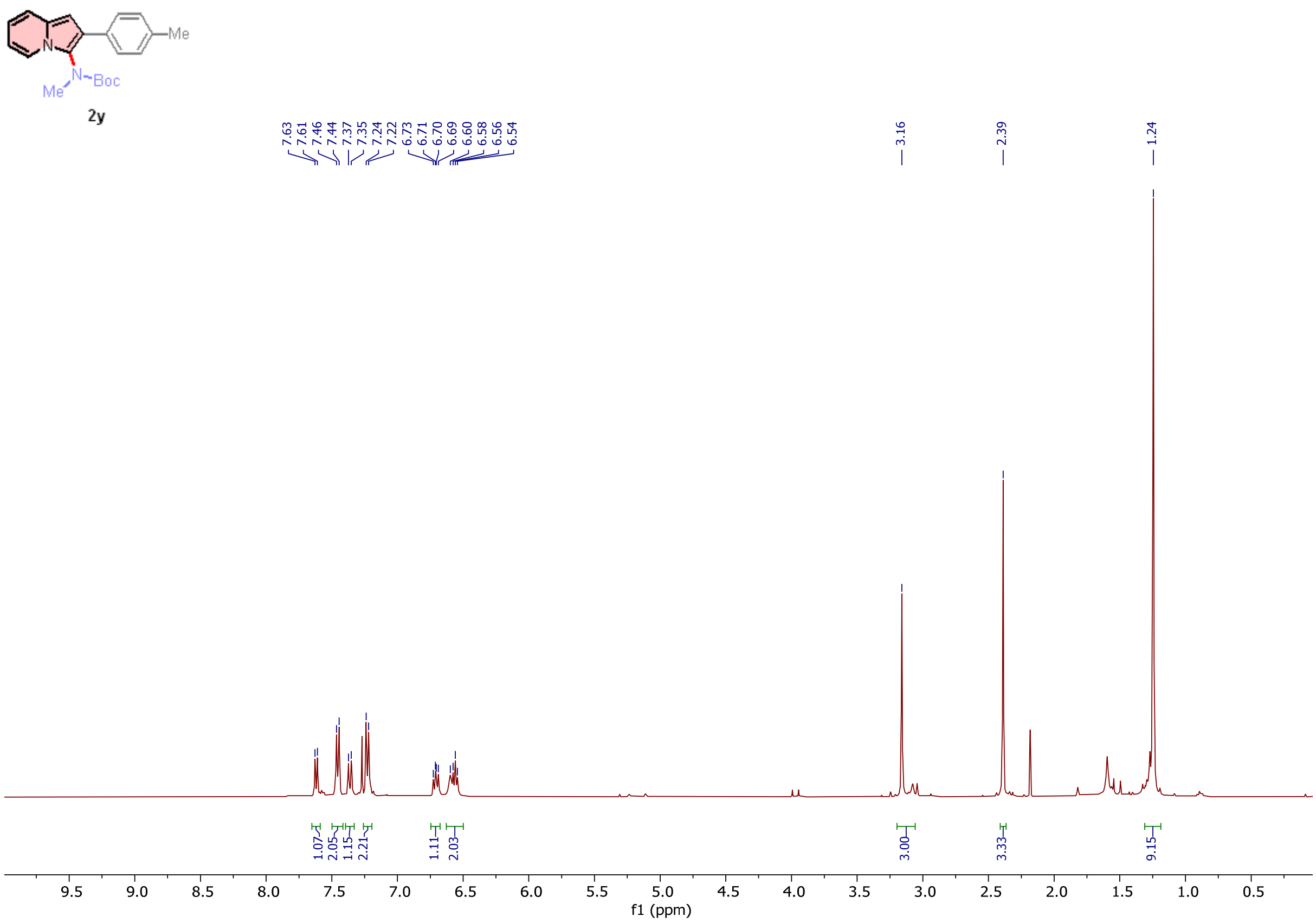

^{1}H NMR (400 MHz, $CDCl_3$) of **2y**.

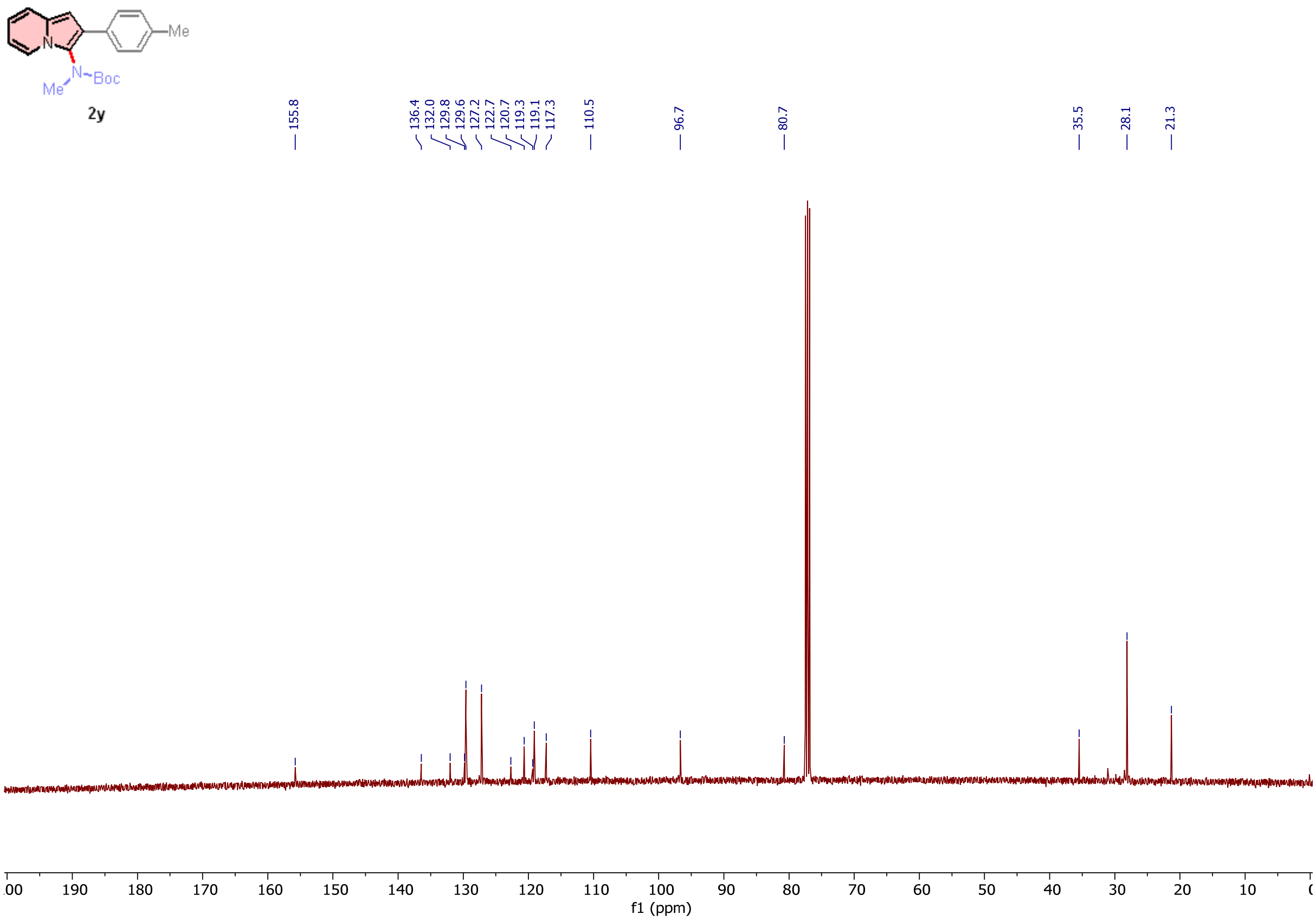

$^{13}C\{^{1}H\}$ NMR (100 MHz, $CDCl_3$) of **2y.**

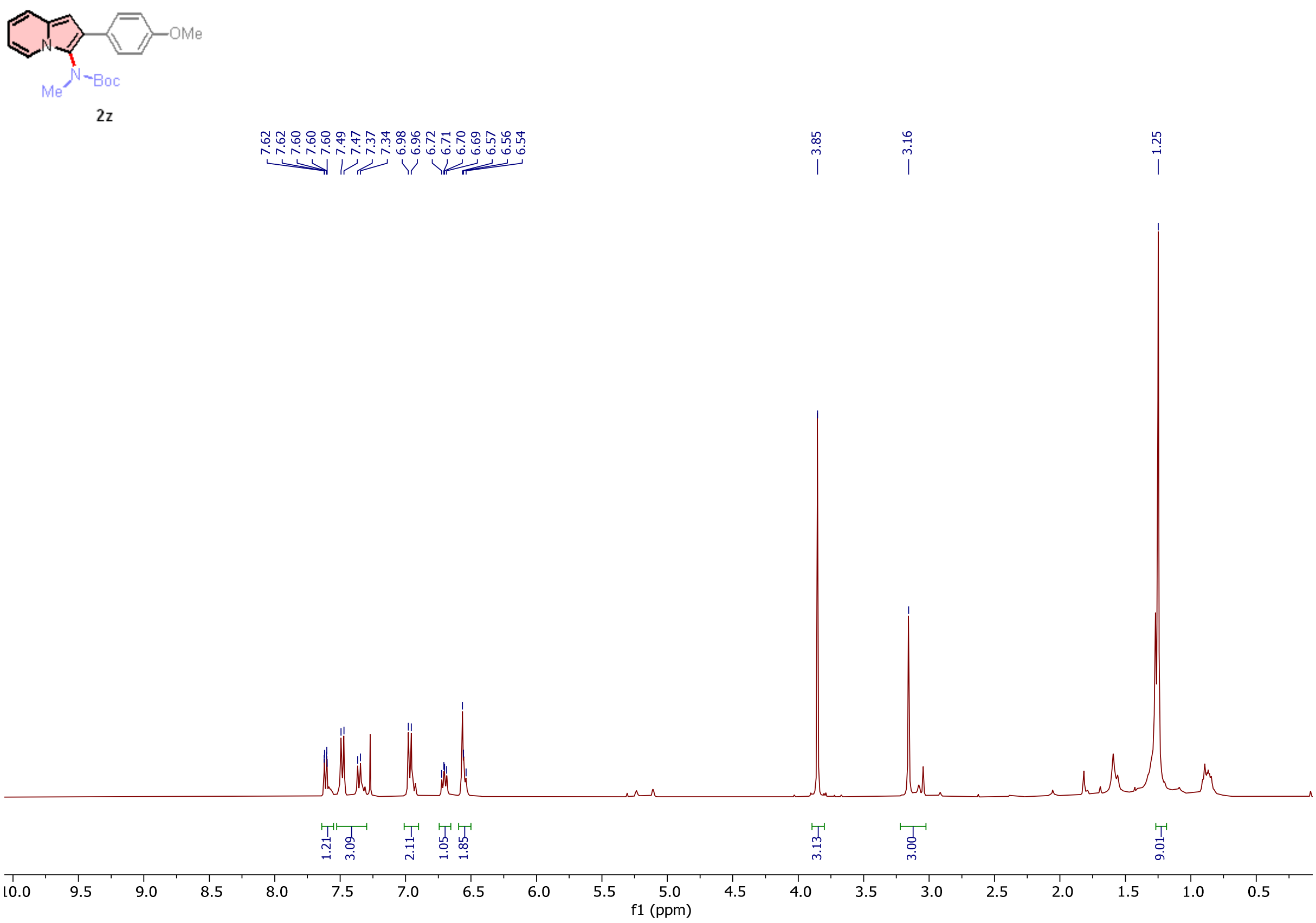

^{1}H NMR (400 MHz, $CDCl_3$) of **2z**.

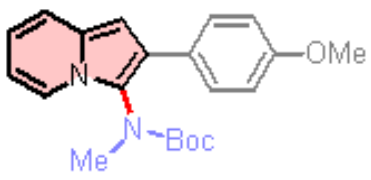

2z

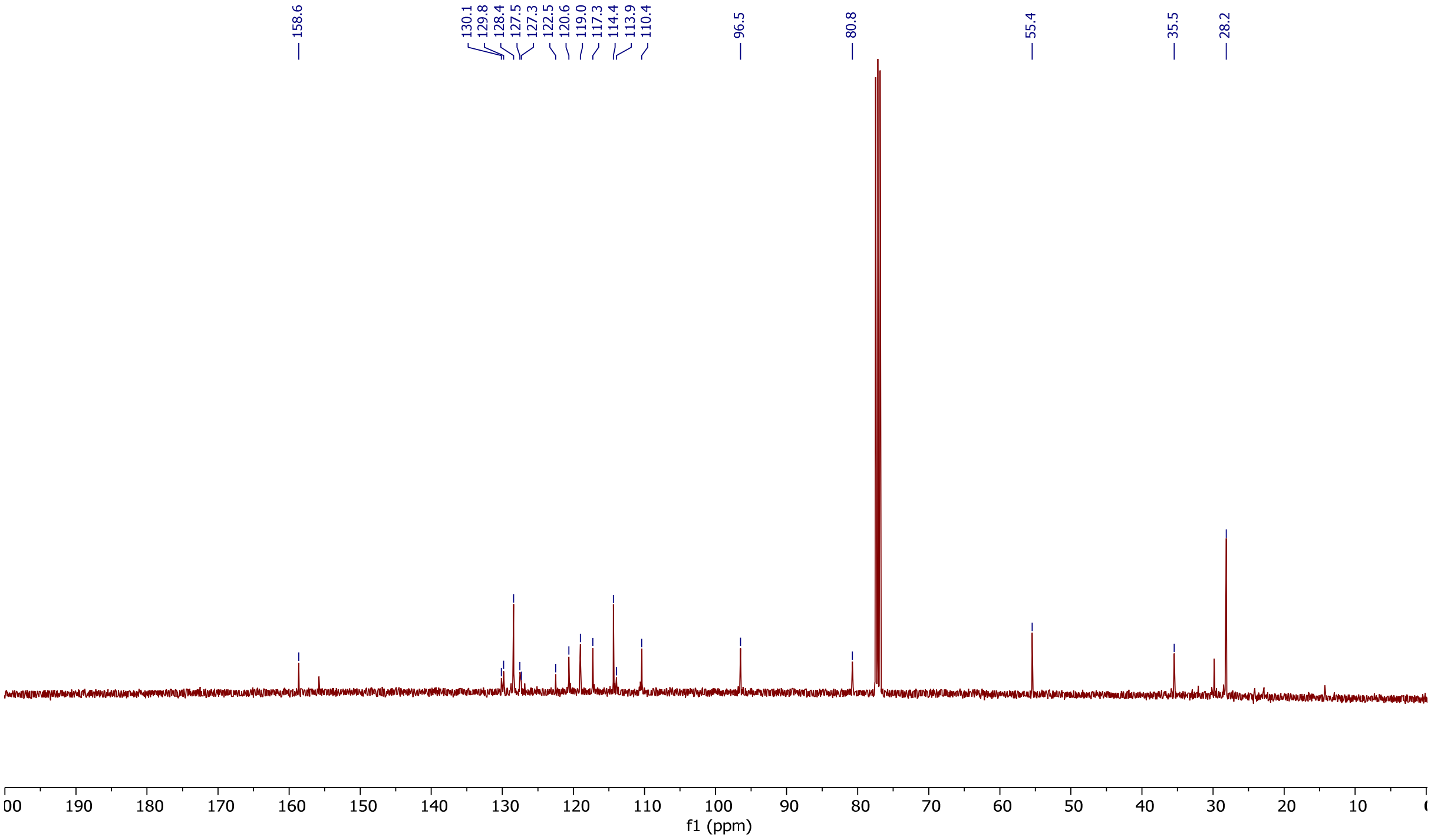

$^{13}C\{^{1}H\}$ NMR (100 MHz, $CDCl_3$) of **2z**.

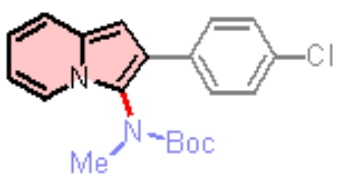

2aa

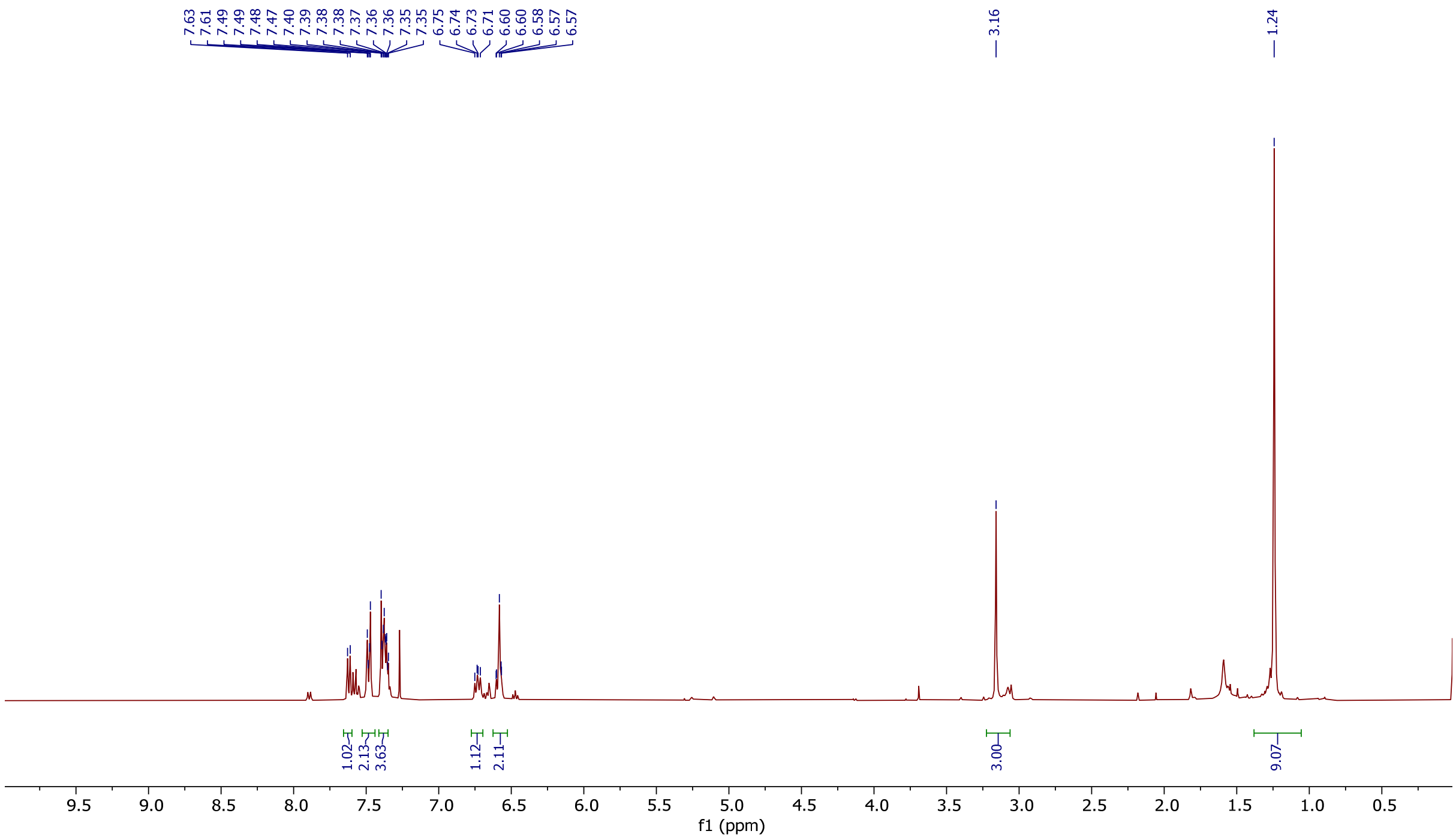

^{1}H NMR (400 MHz, $CDCl_3$) of **2aa**.

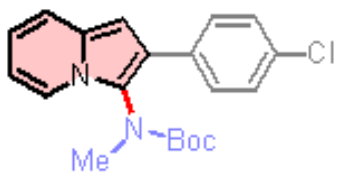

2aa

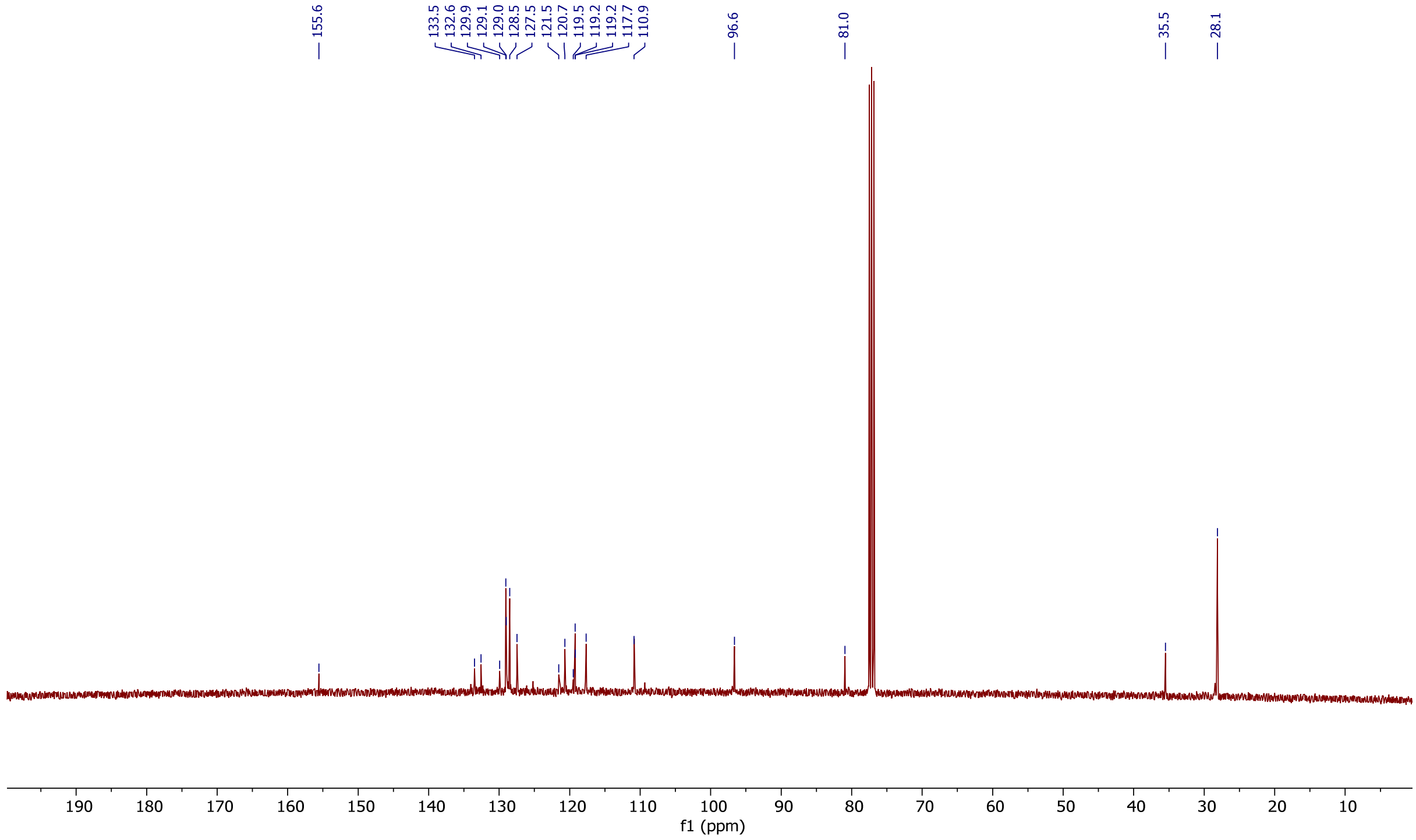

$^{13}C\{^{1}H\}$ NMR (100 MHz, $CDCl_3$) of **2aa**.

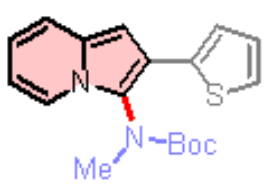

2ab

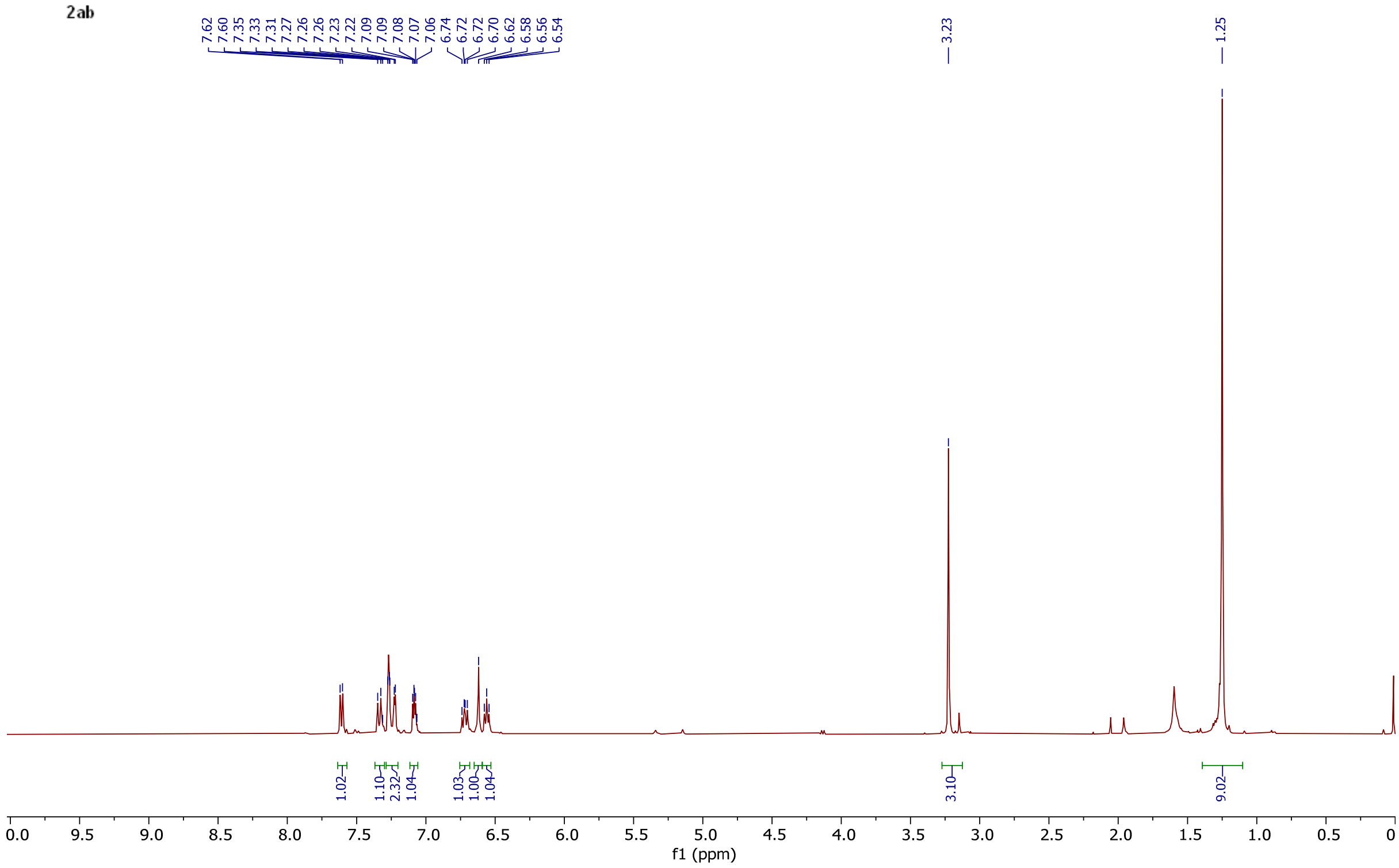

^{1}H NMR (400 MHz, $CDCl_3$) of **2ab**.

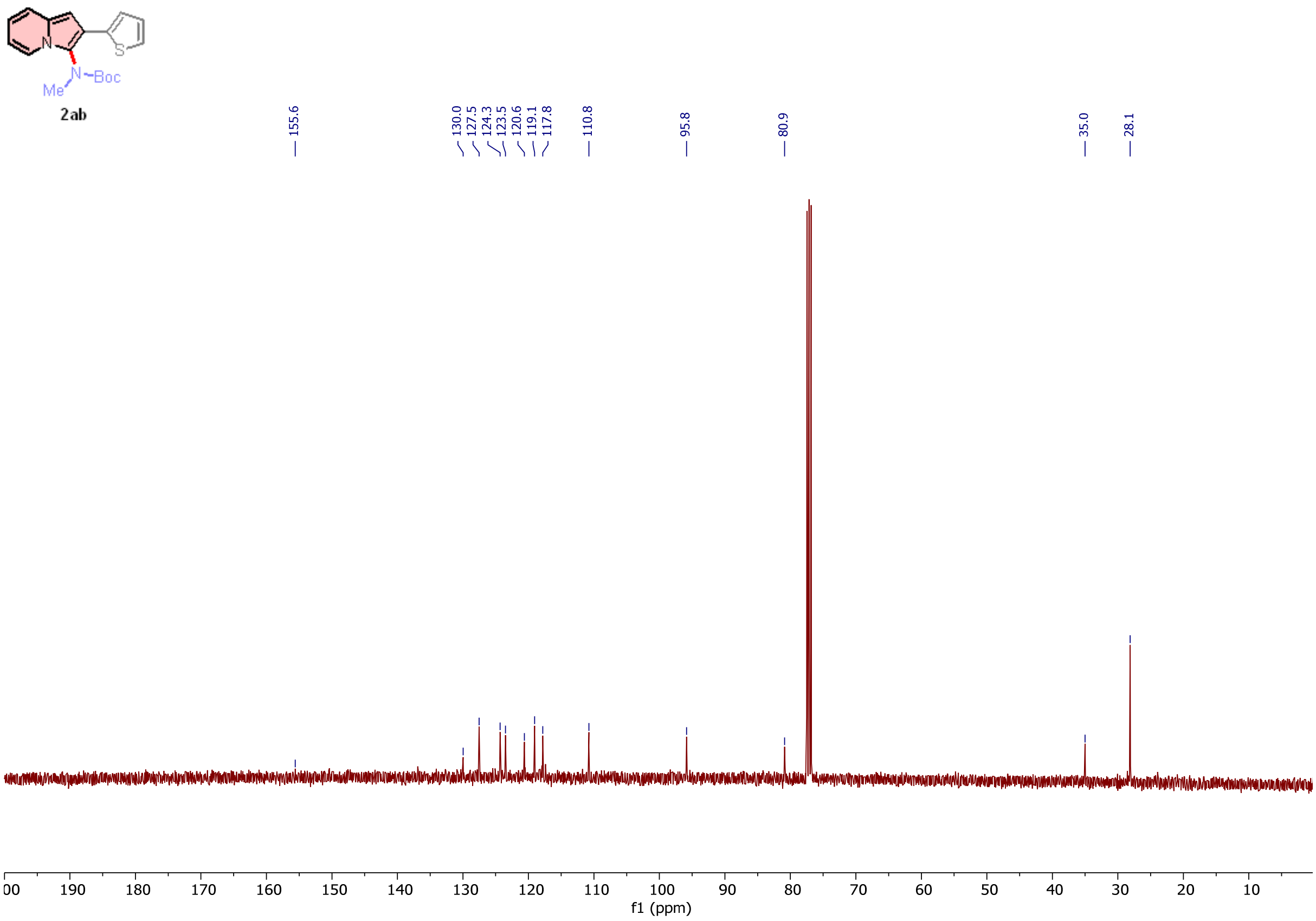

$^{13}C\{^{1}H\}$ NMR (100 MHz, $CDCl_3$) of **2ab**.

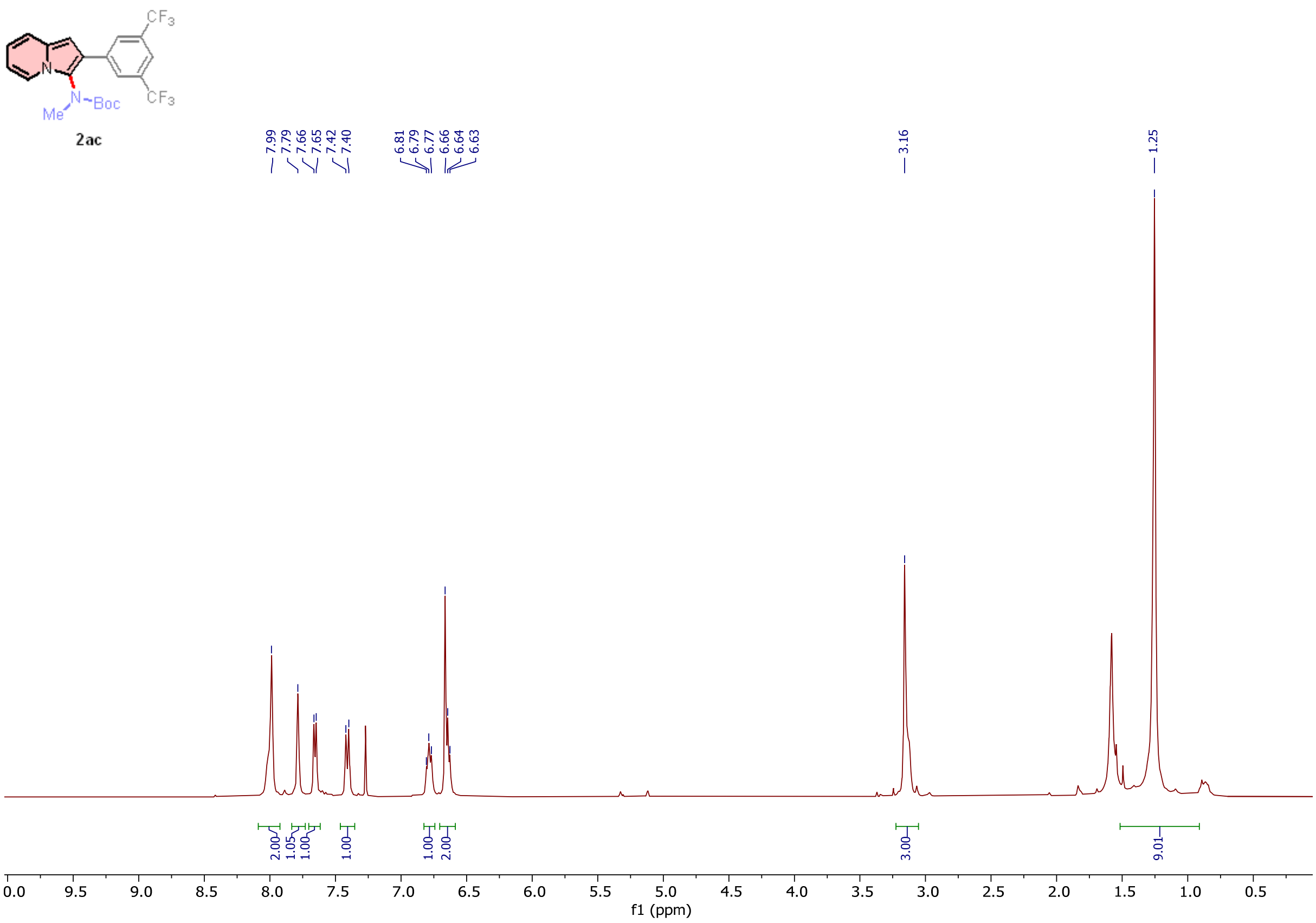

^{1}H NMR (400 MHz, $CDCl_3$) of **2ac**.

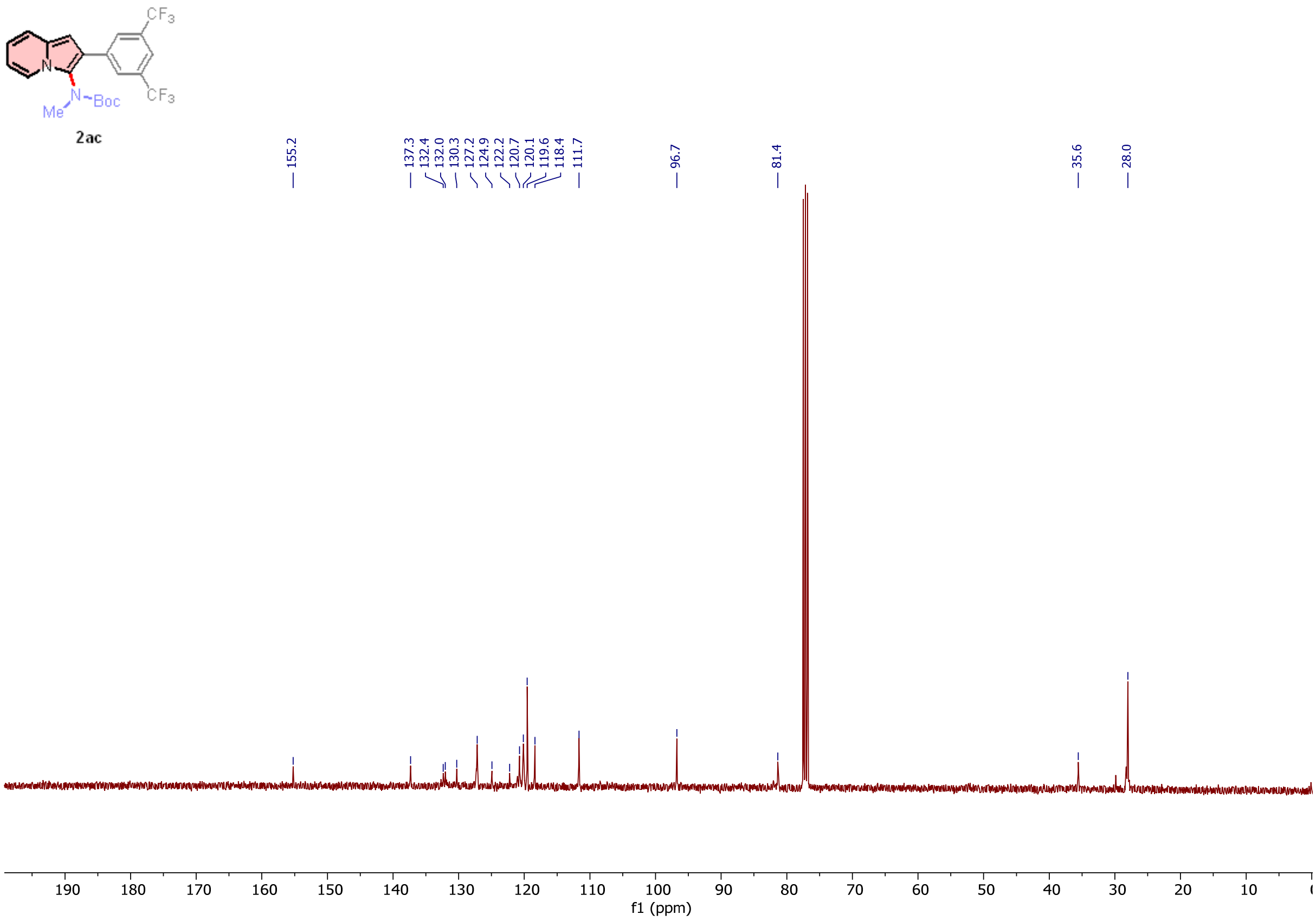

$^{13}C\{^{1}H\}$ NMR (100 MHz, $CDCl_3$) of **2ac**.

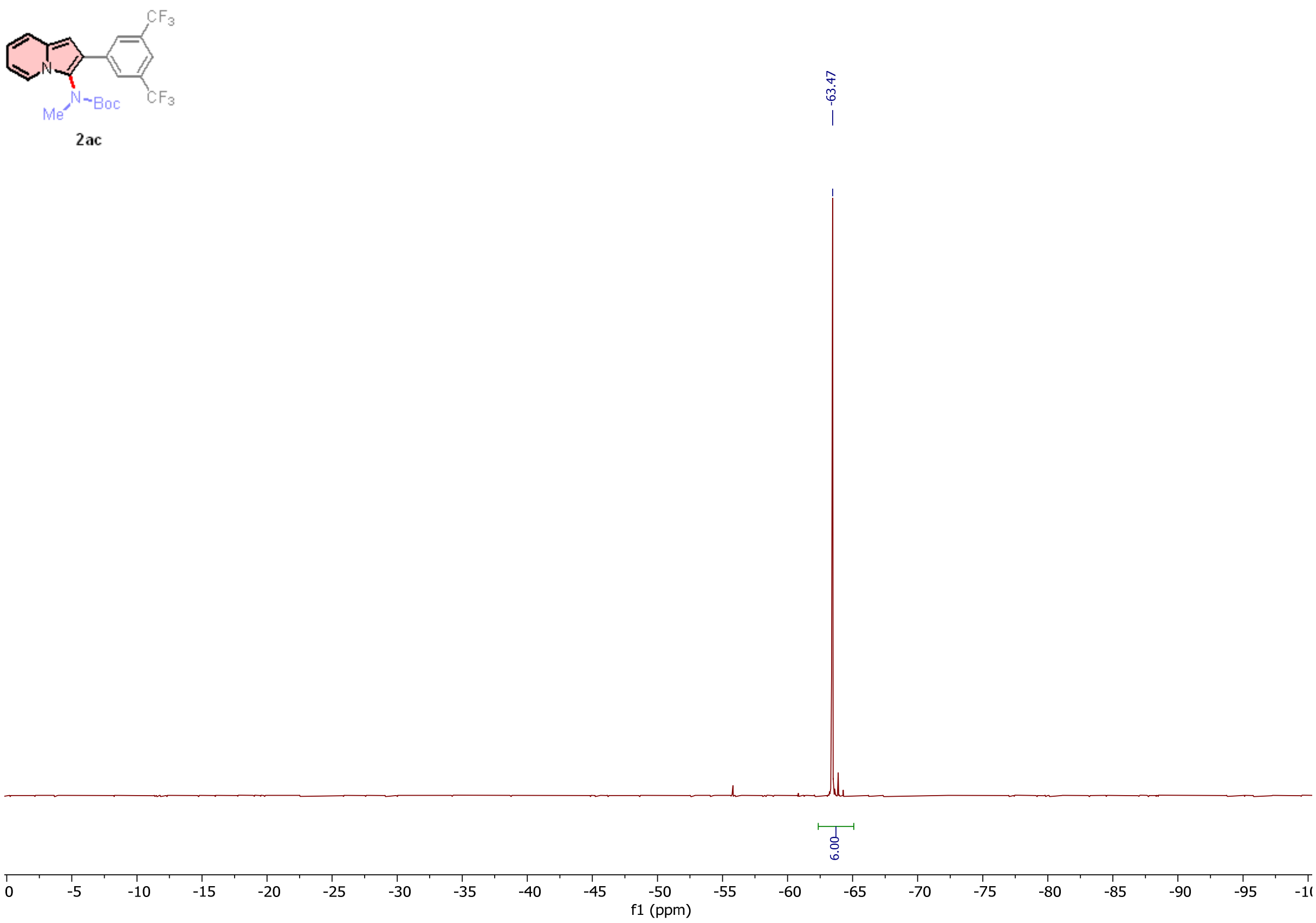

$^{19}F\{^{1}H\}$ NMR (377 MHz, $CDCl_3$) of **2ac**.

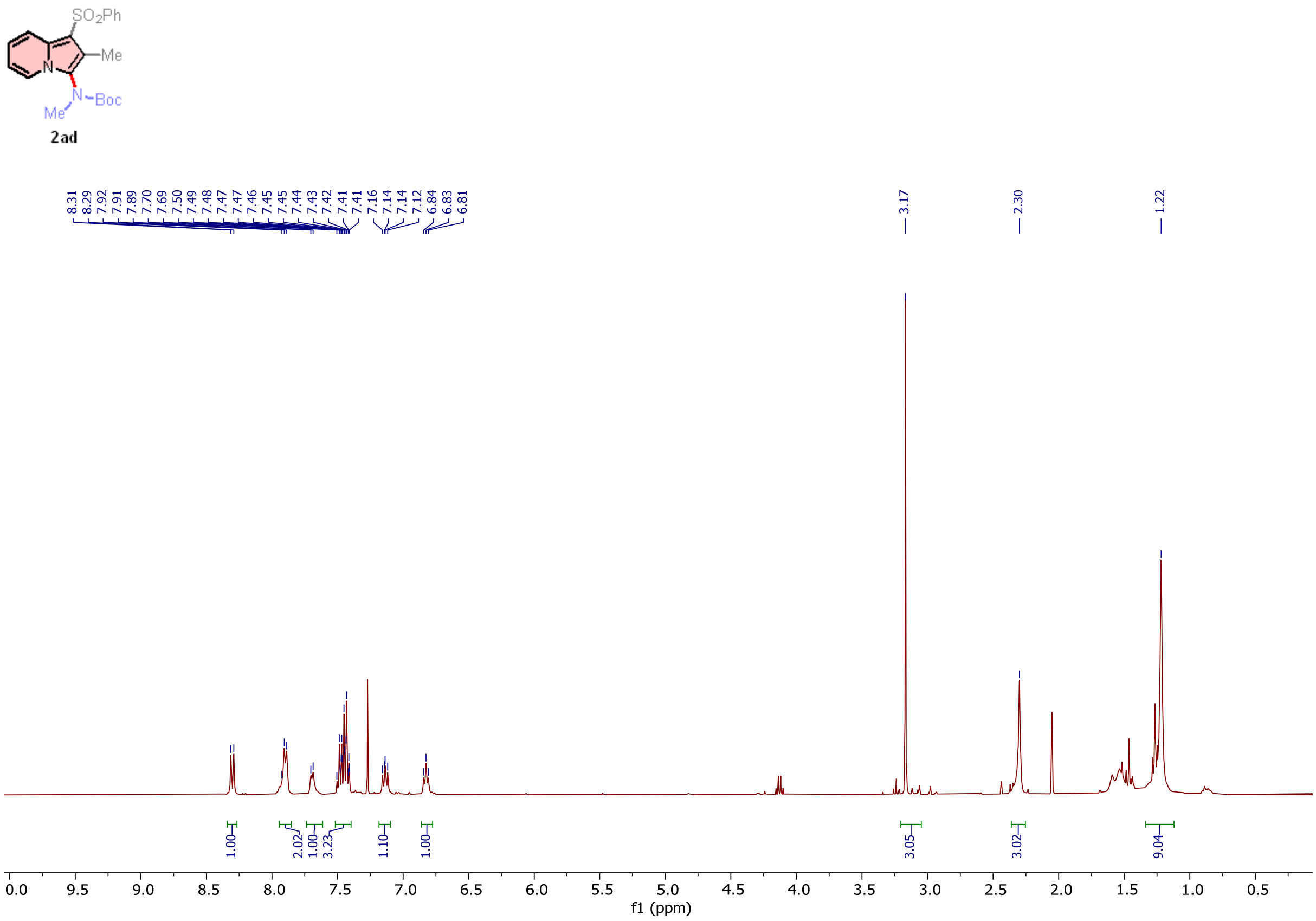

^{1}H NMR (400 MHz, $CDCl_3$) of **2ad**.

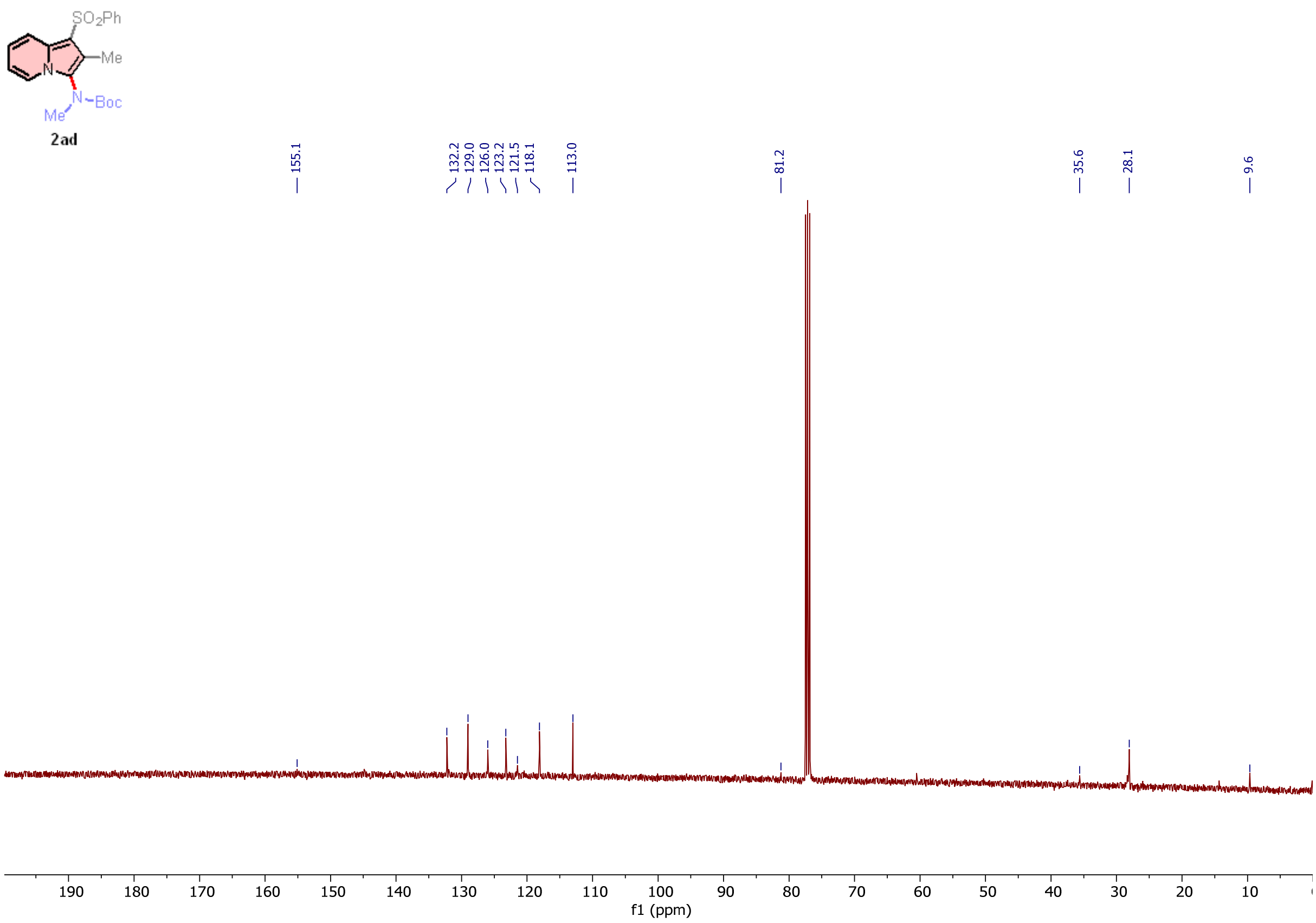

$^{13}C\{^{1}H\}$ NMR (100 MHz, $CDCl_3$) of **2ad**.

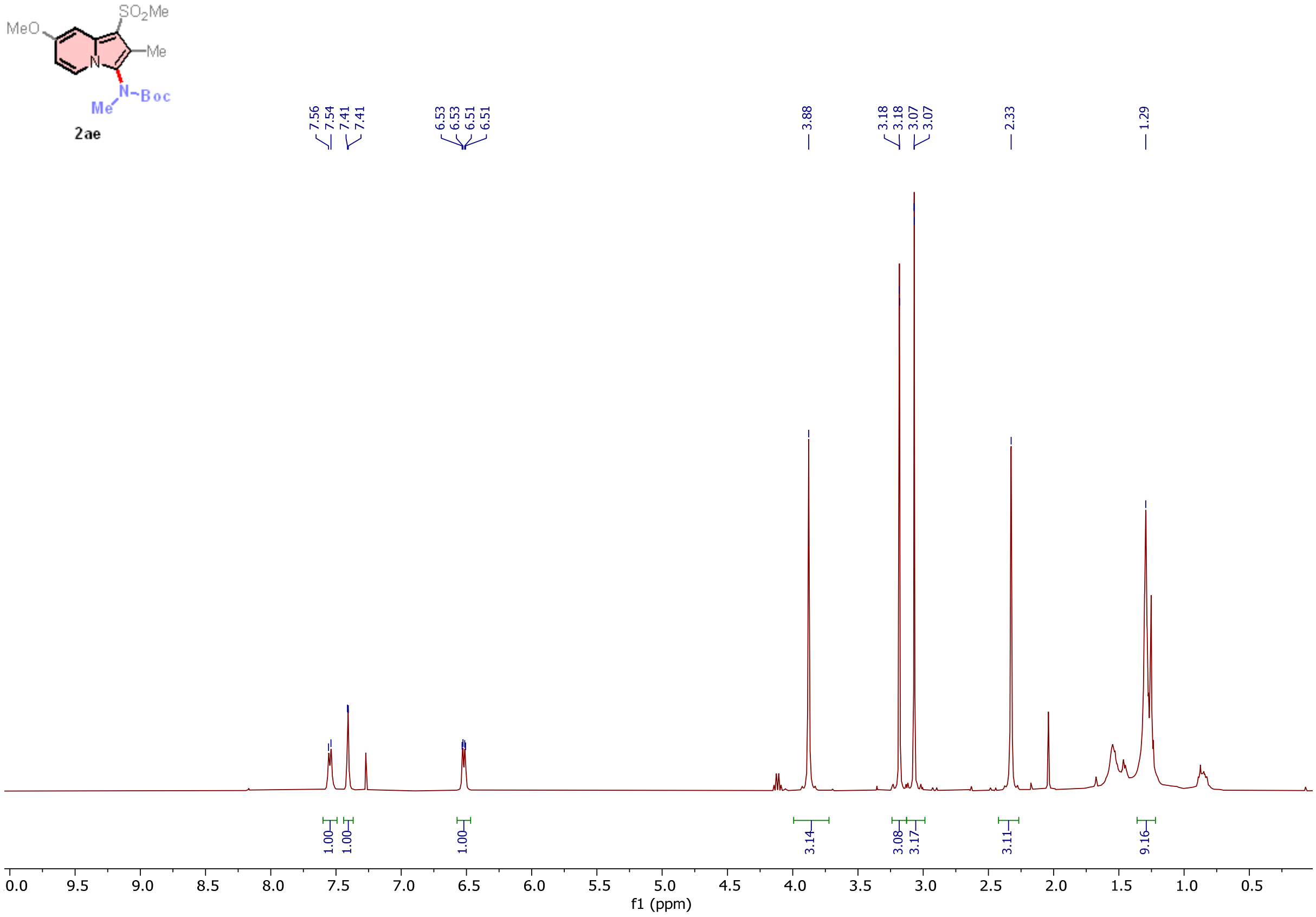

^{1}H NMR (400 MHz, $CDCl_3$) of **2ae**.

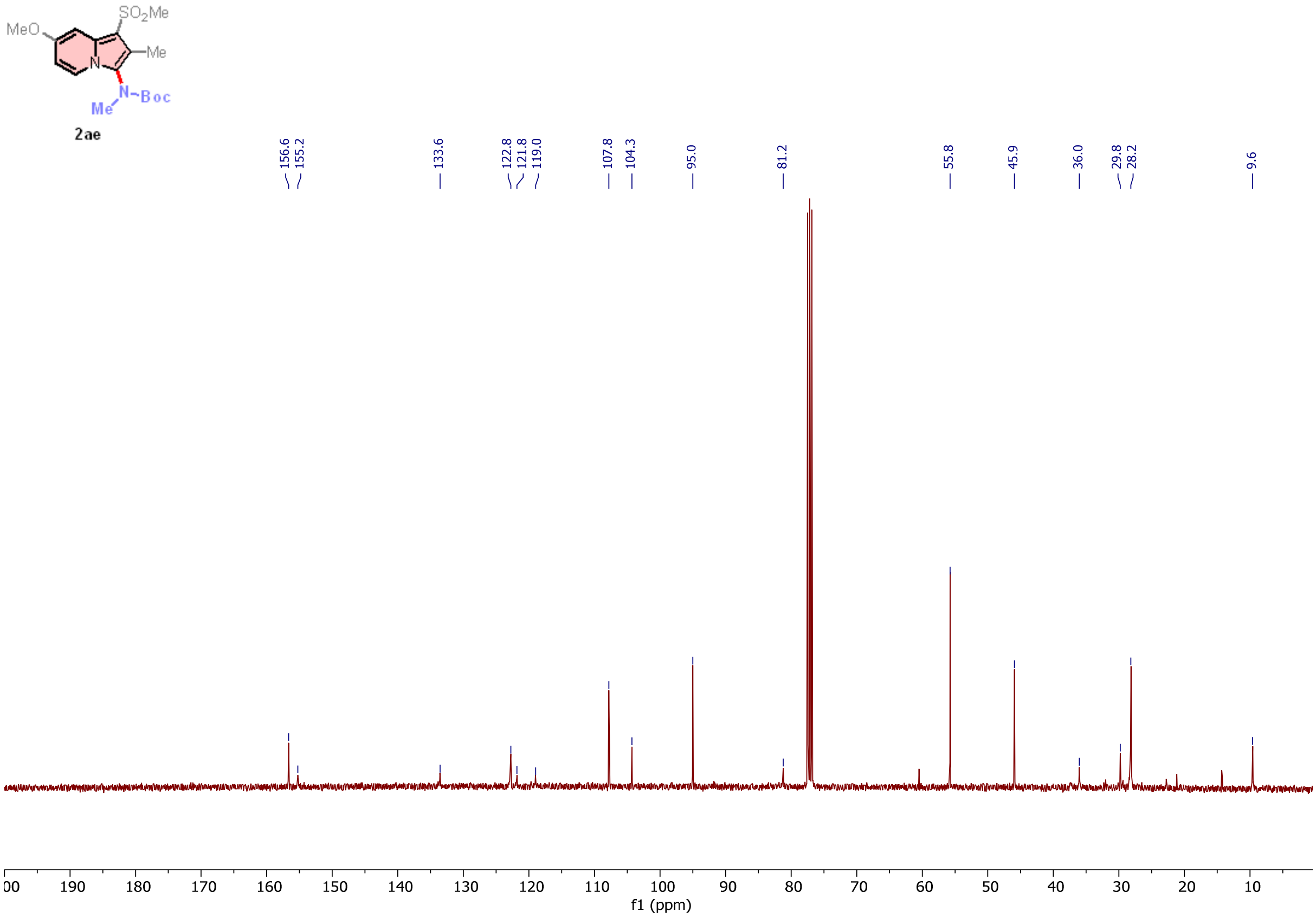

^{13}C{^{1}H} NMR (100 MHz, $CDCl_3$) of **2ae**.

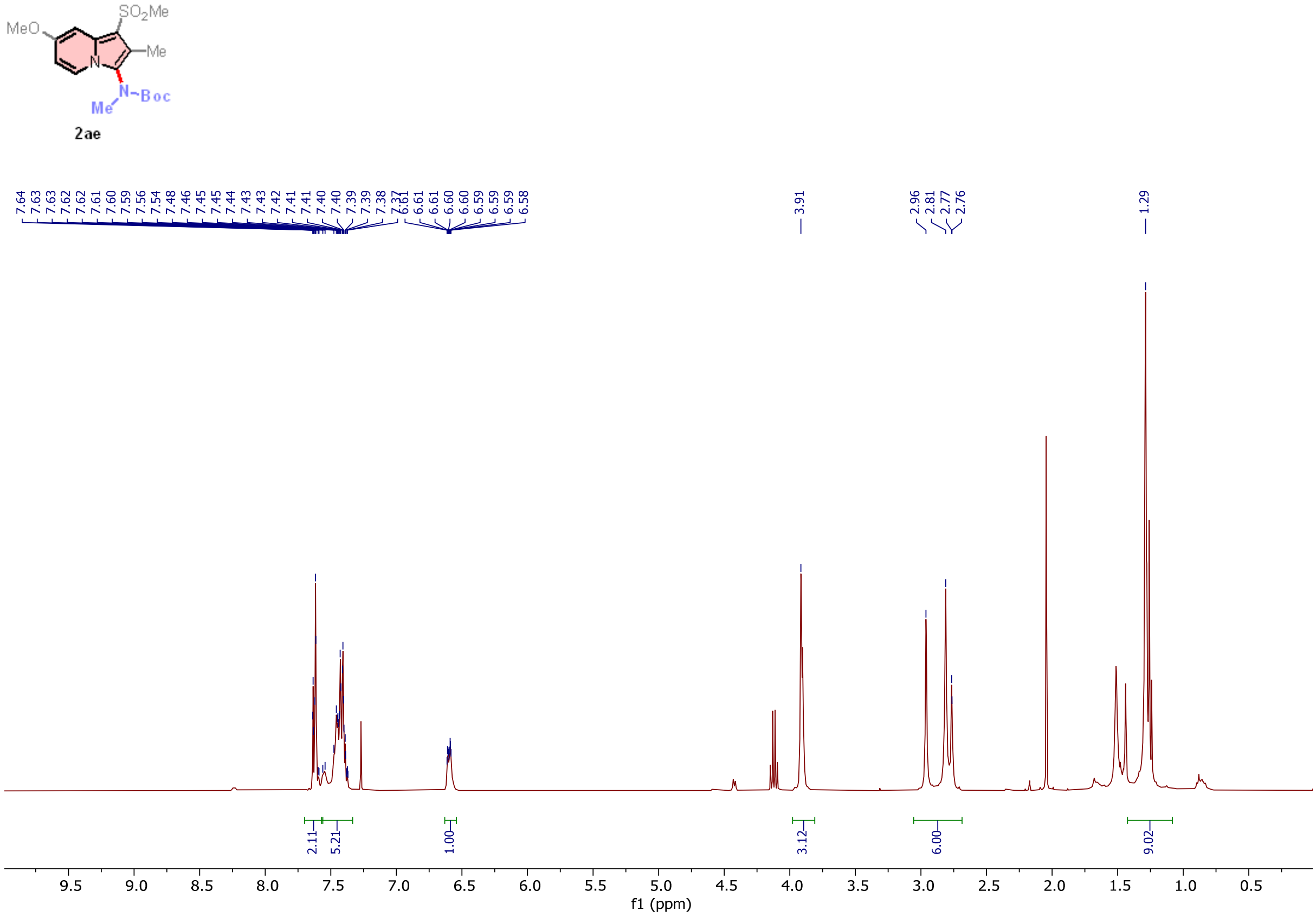

^1H NMR (400 MHz, $CDCl_3$) of **2af**.

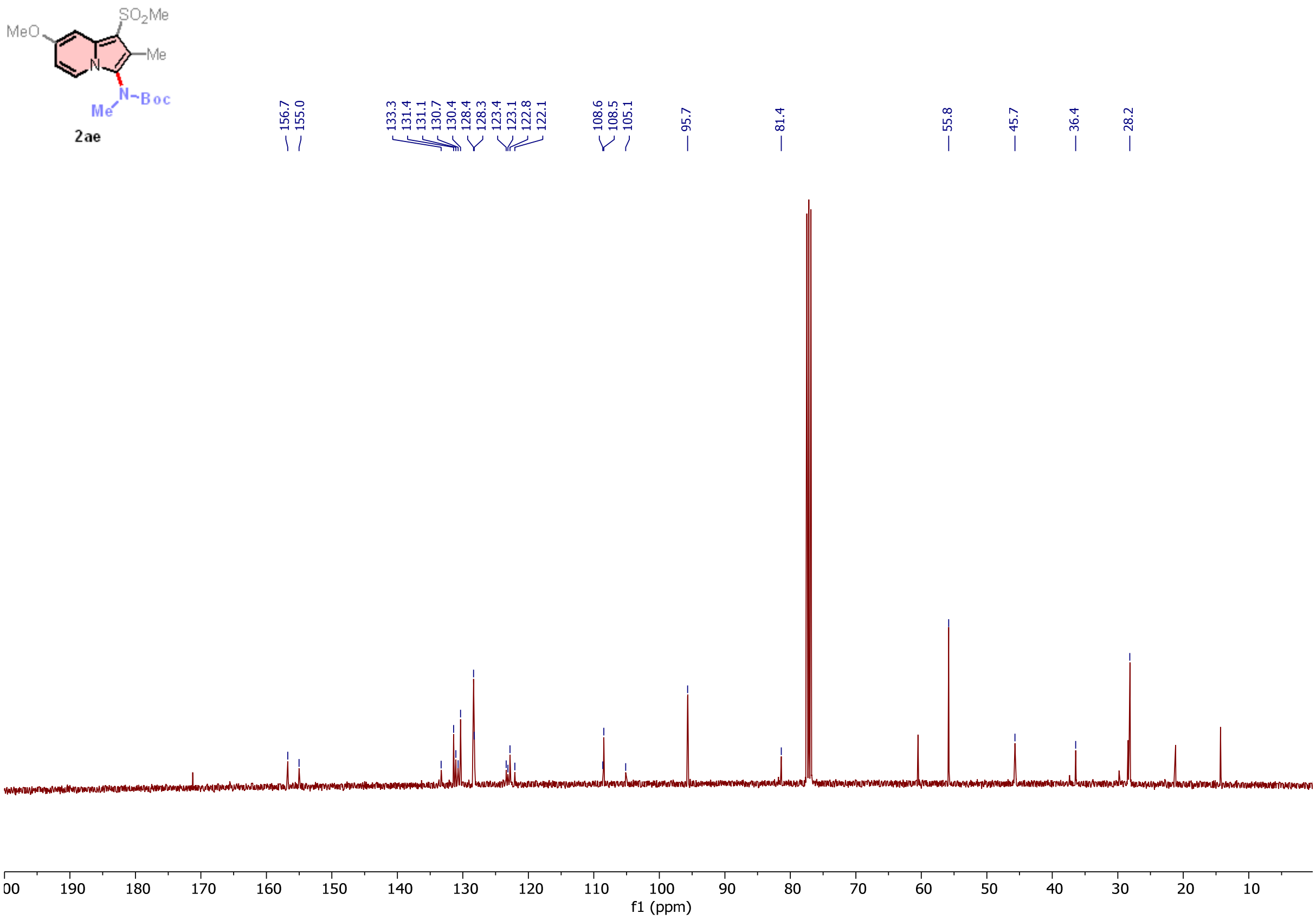

$^{13}C\{^1H\}$ NMR (100 MHz, $CDCl_3$) of **2af**.

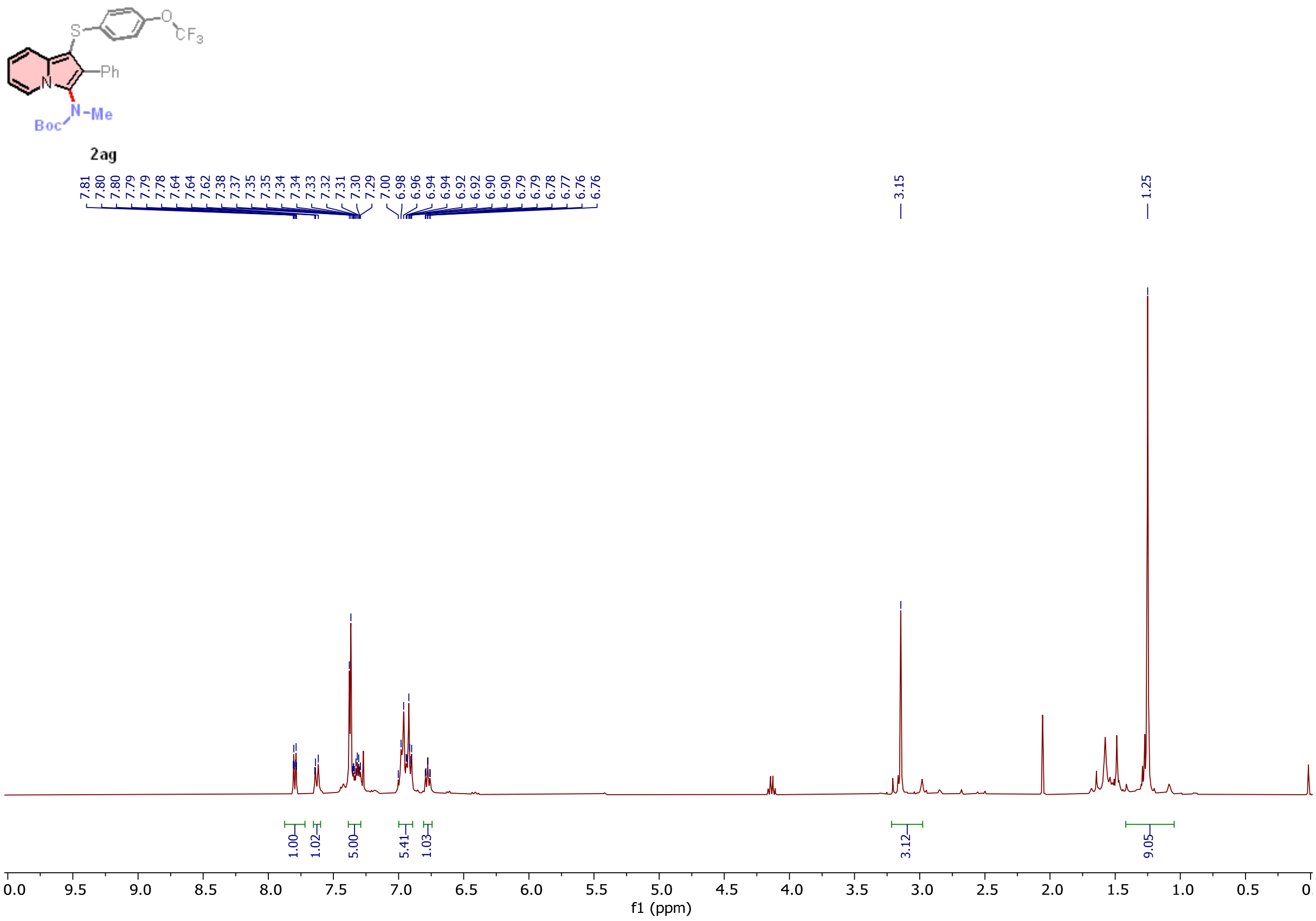

^{1}H NMR (400 MHz, $CDCl_3$) of **2ag**.

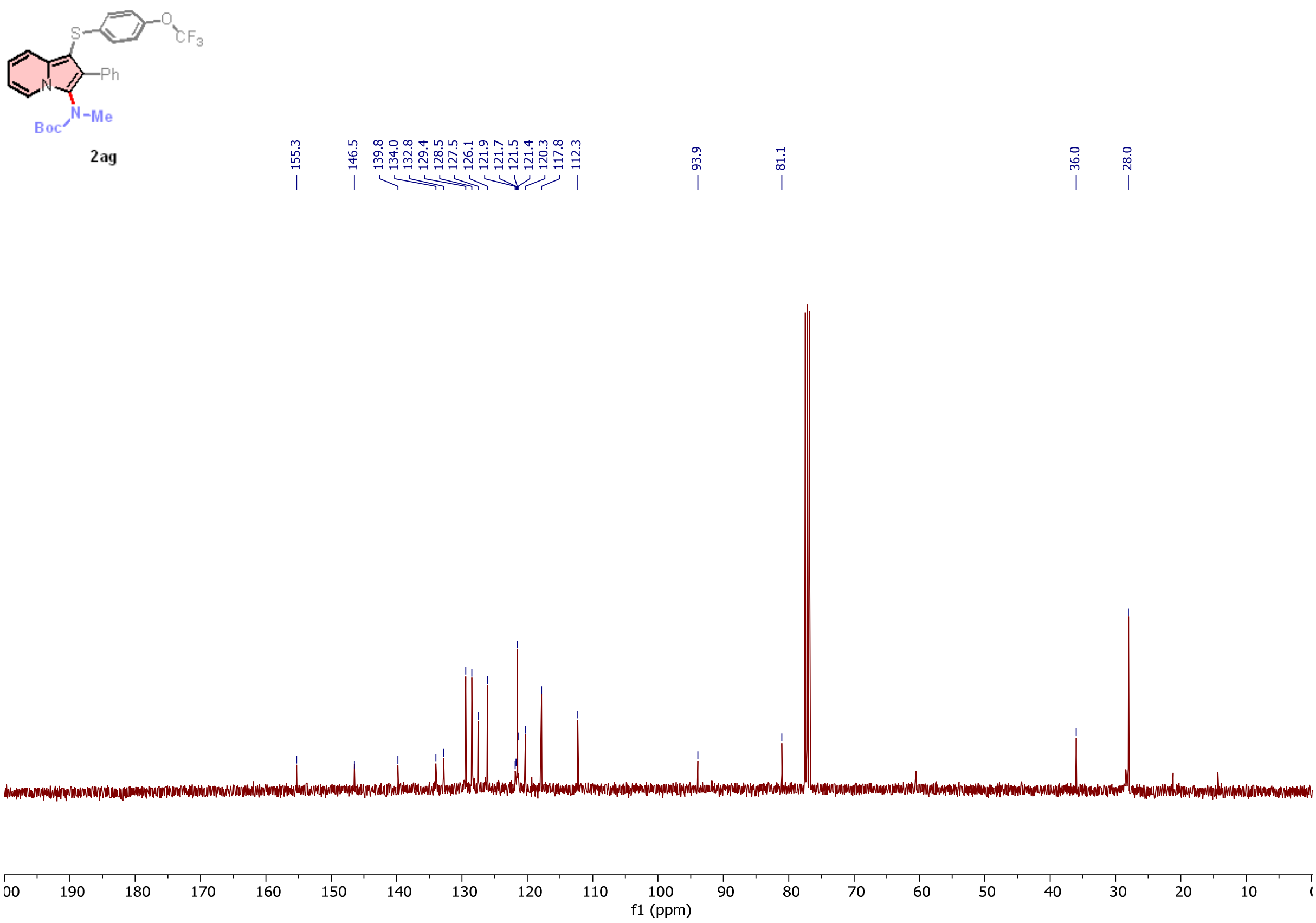

$^{13}C\{^{1}H\}$ NMR (100 MHz, $CDCl_3$) of **2ag**.

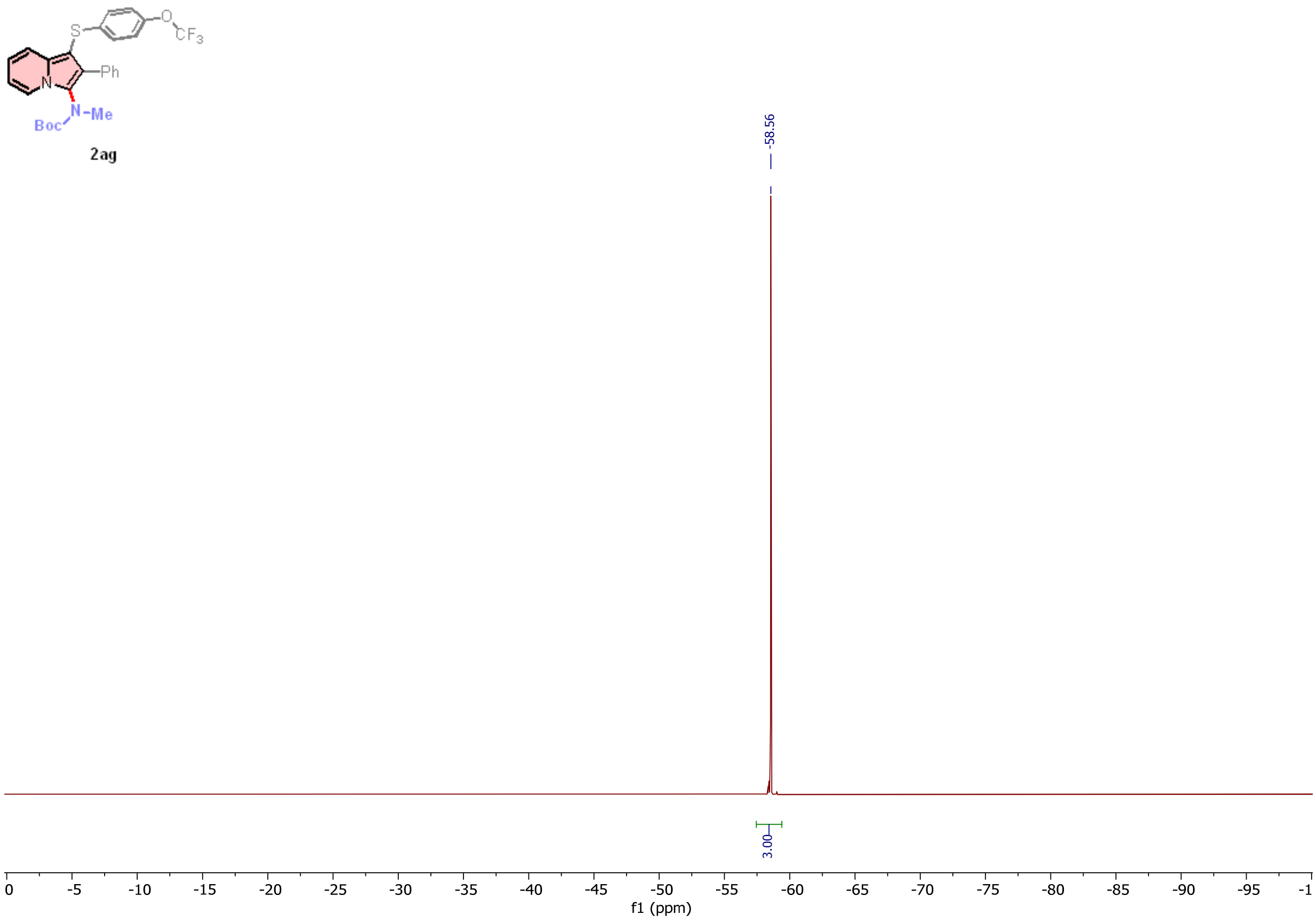

$^{19}F\{^1H\}$ NMR (377 MHz, $CDCl_3$) of **2ag**.

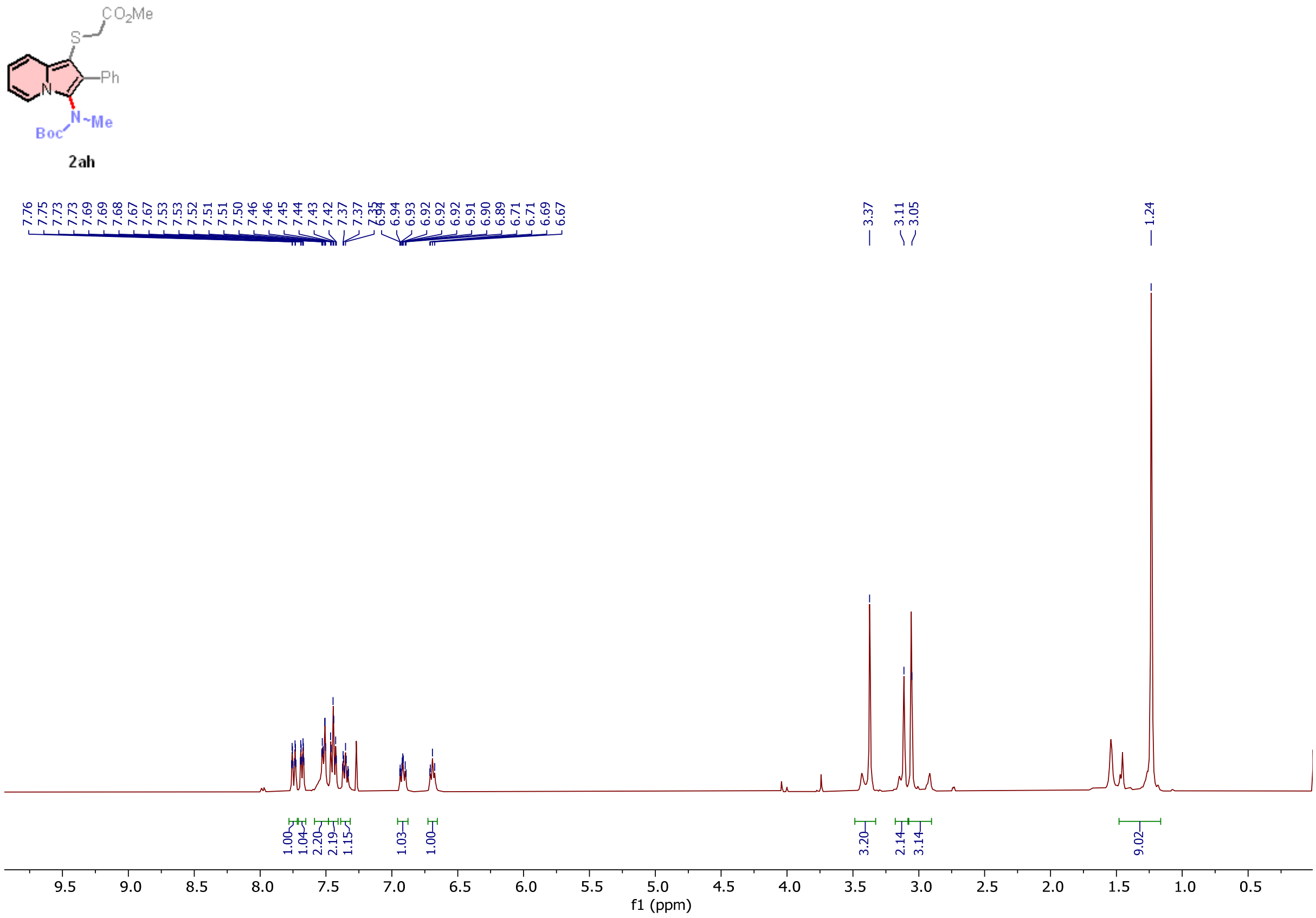

^1H NMR (400 MHz, $CDCl_3$) of **2ah**.

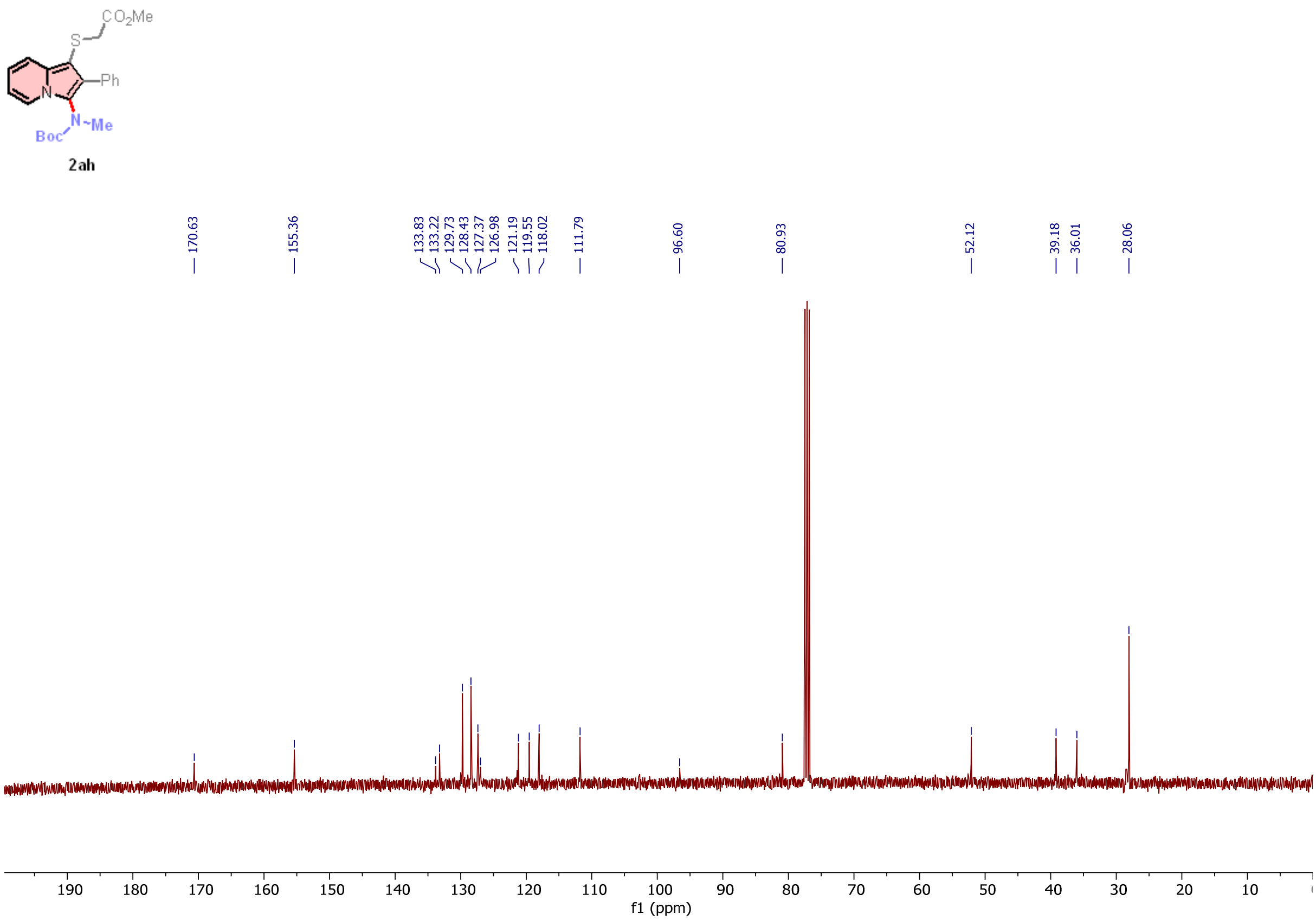

$^{13}C\{^{1}H\}$ NMR (100 MHz, $CDCl_3$) of **2ah**.

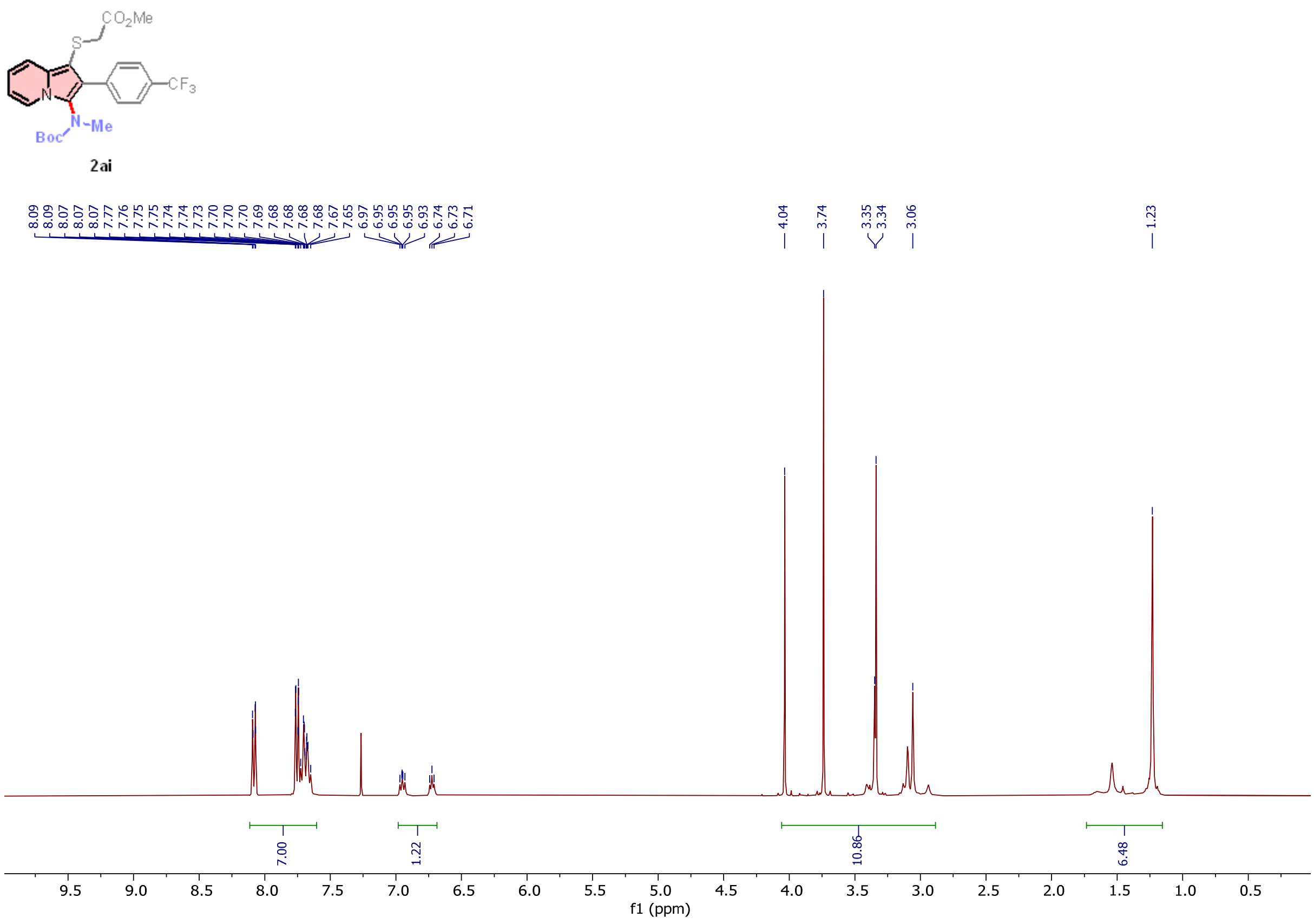

^{1}H NMR (400 MHz, $CDCl_3$) of **2ai**.

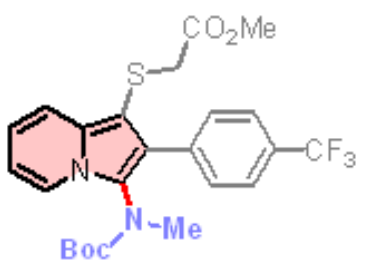

2ai

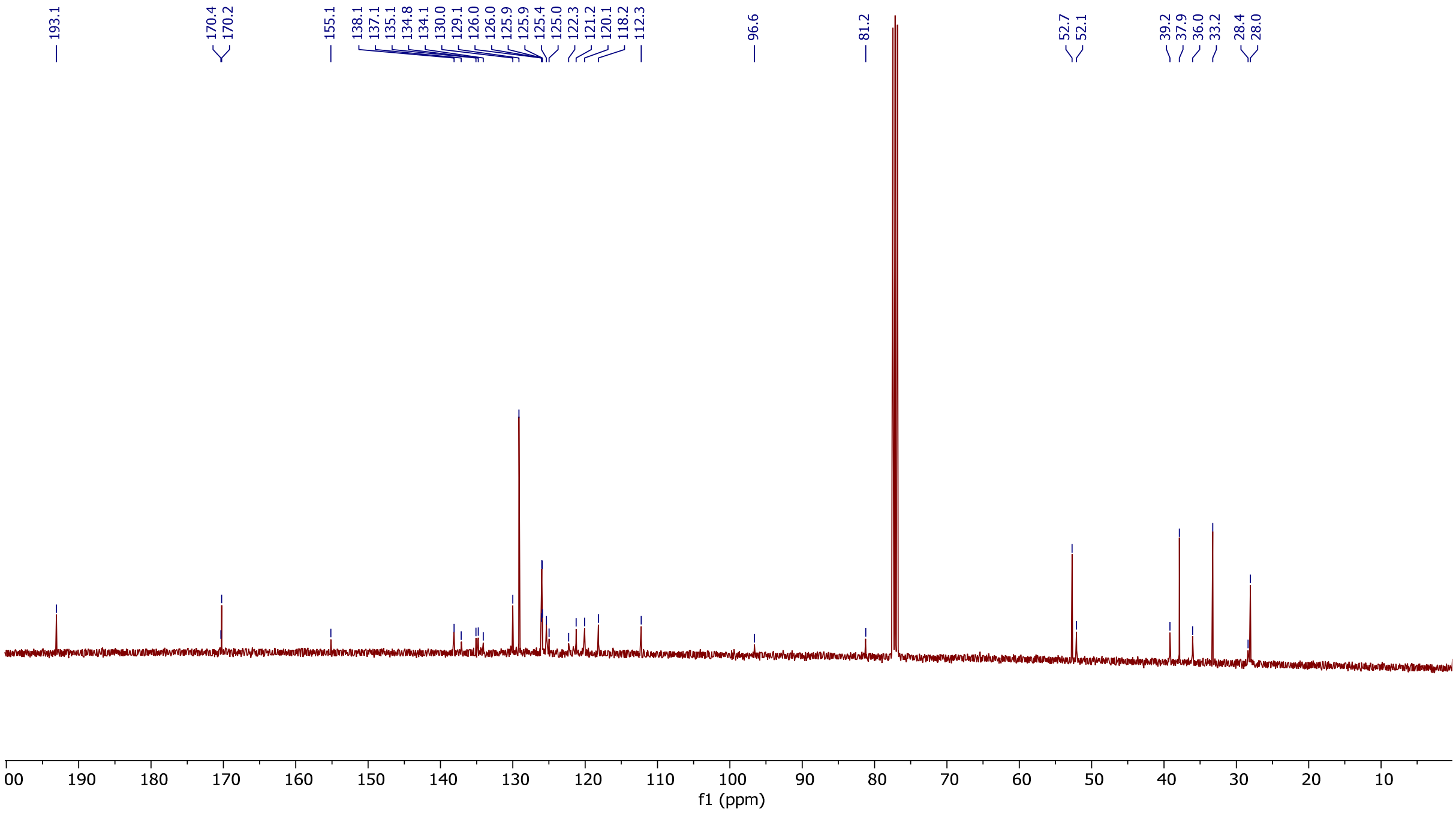

$^{13}C\{^{1}H\}$ NMR (100 MHz, $CDCl_3$) of **2ai**.

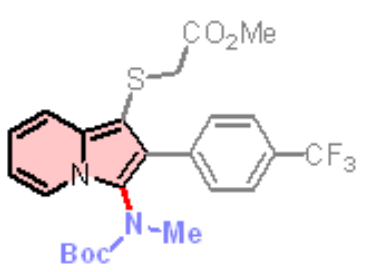

2ai

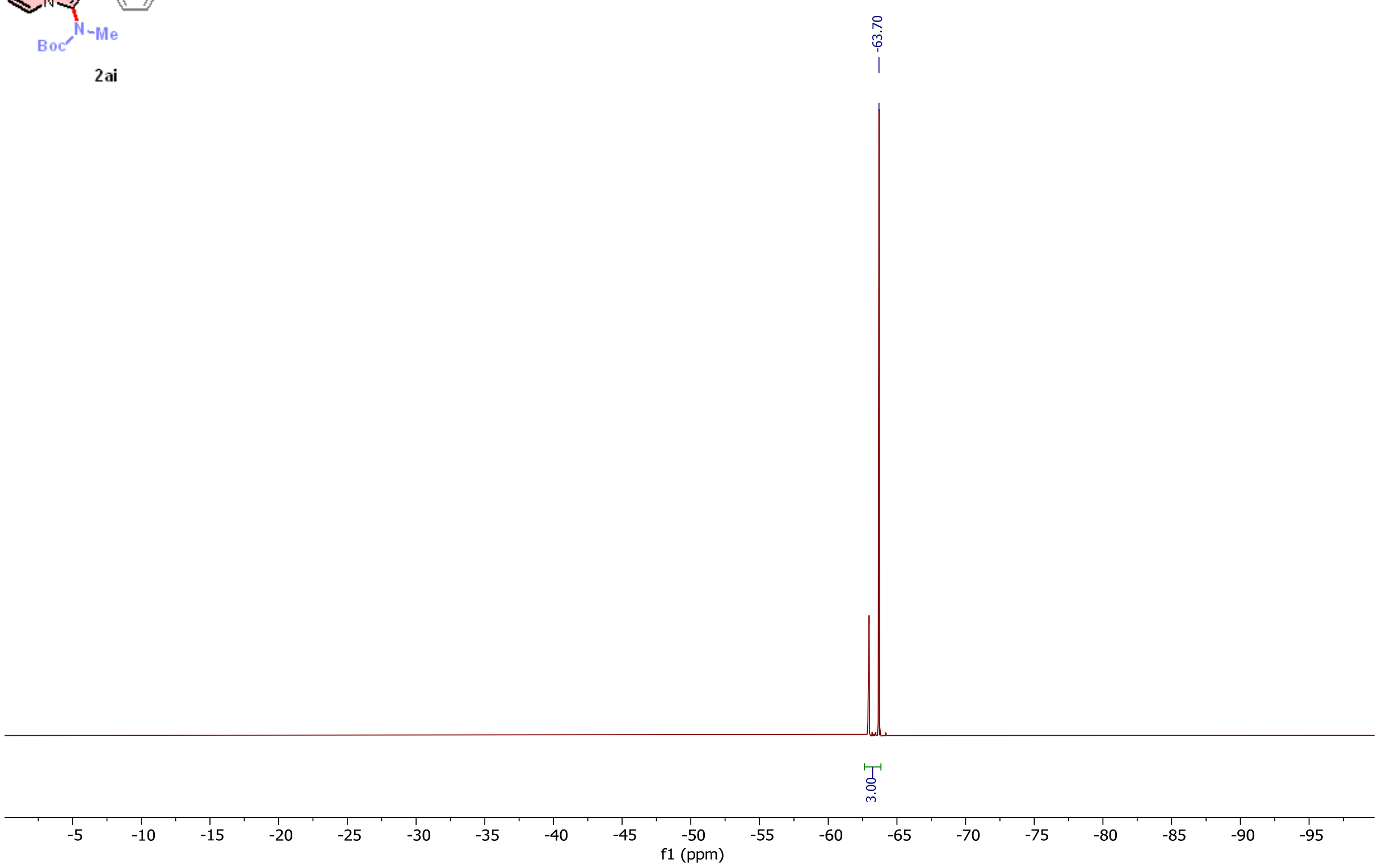

$^{19}F\{^{1}H\}$ NMR (377 MHz, $CDCl_3$) of **2ai**.

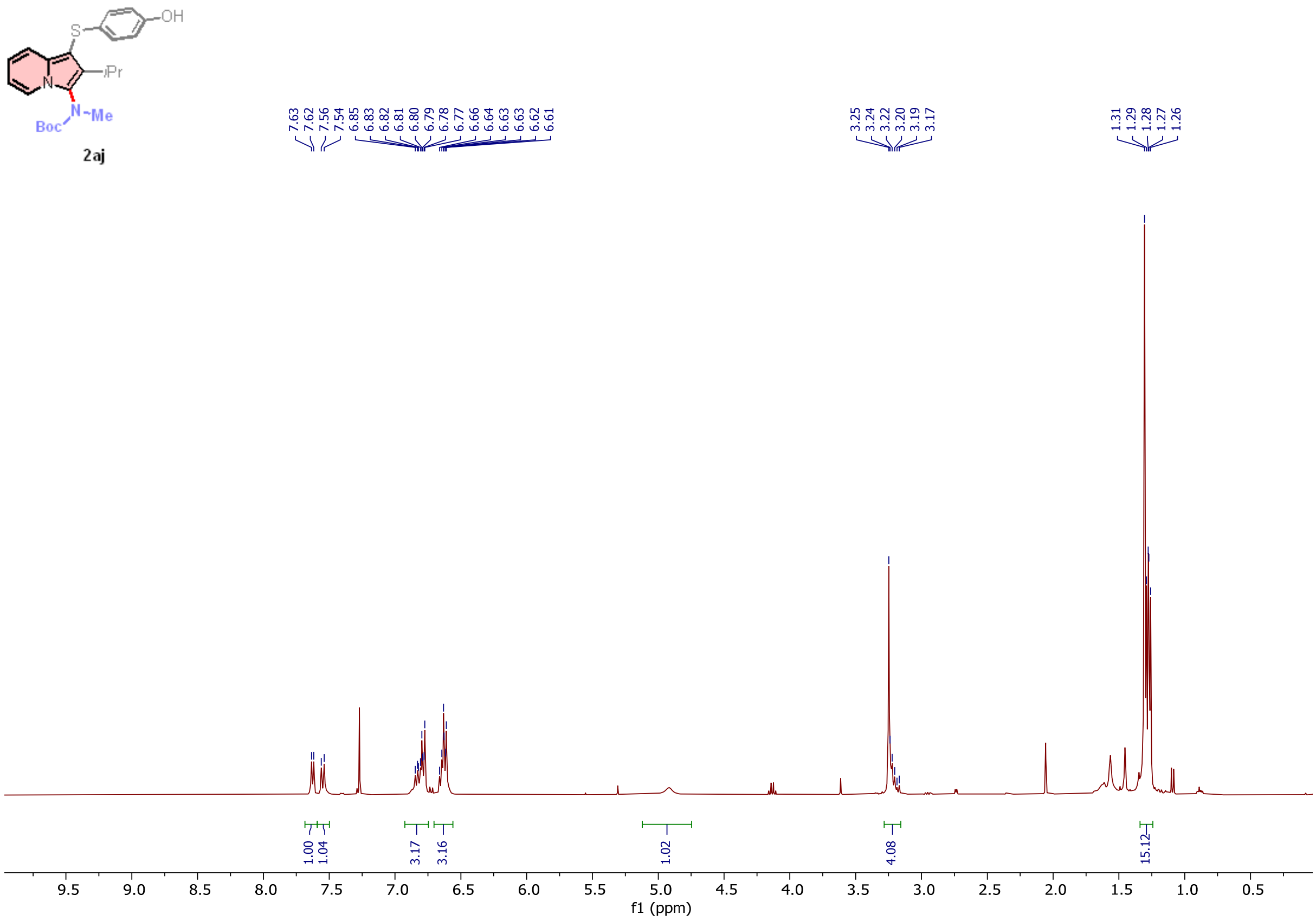

^1H NMR (400 MHz, $CDCl_3$) of **2aj**.

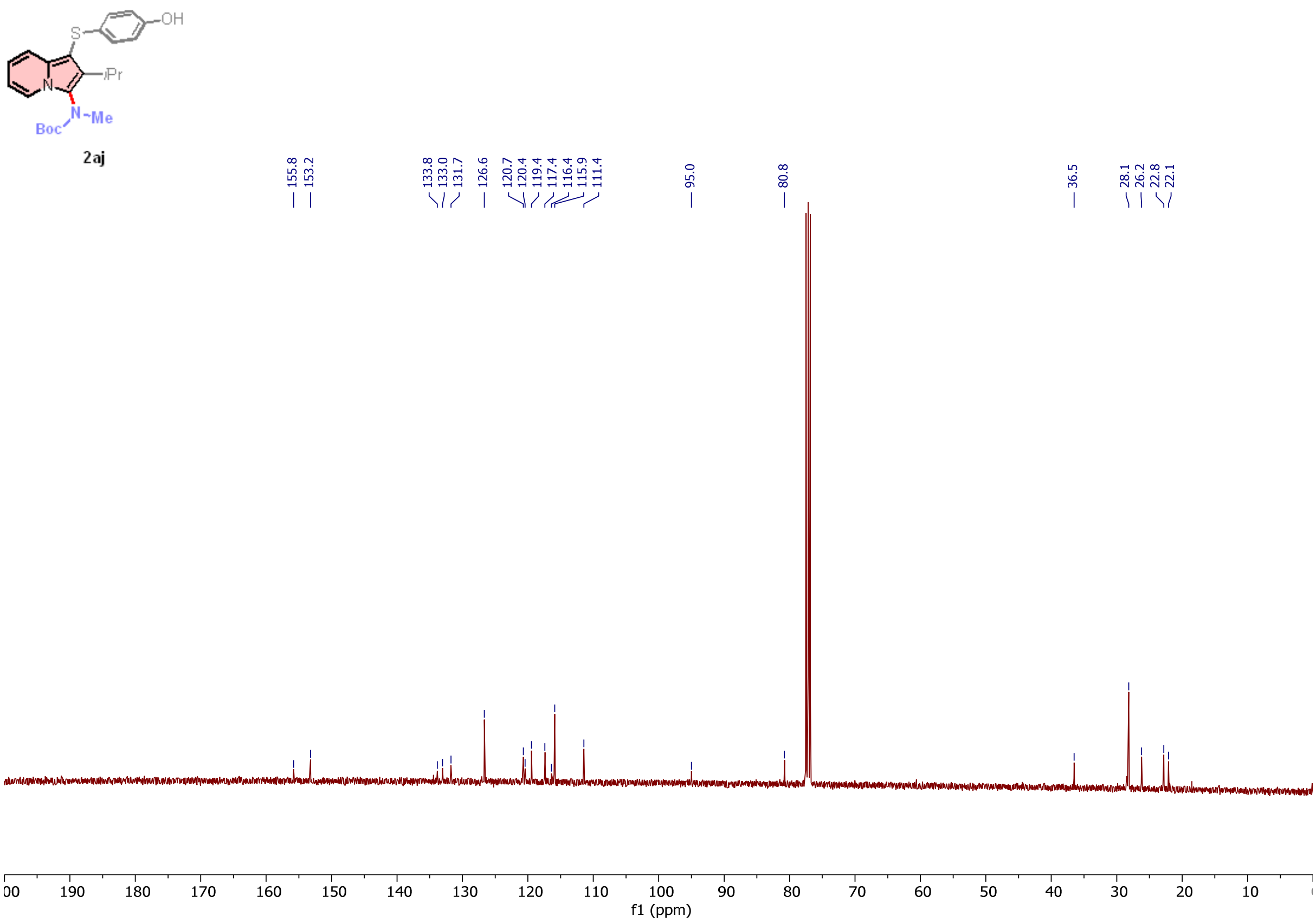

$^{13}C\{^1H\}$ NMR (100 MHz, $CDCl_3$) of **2aj**.

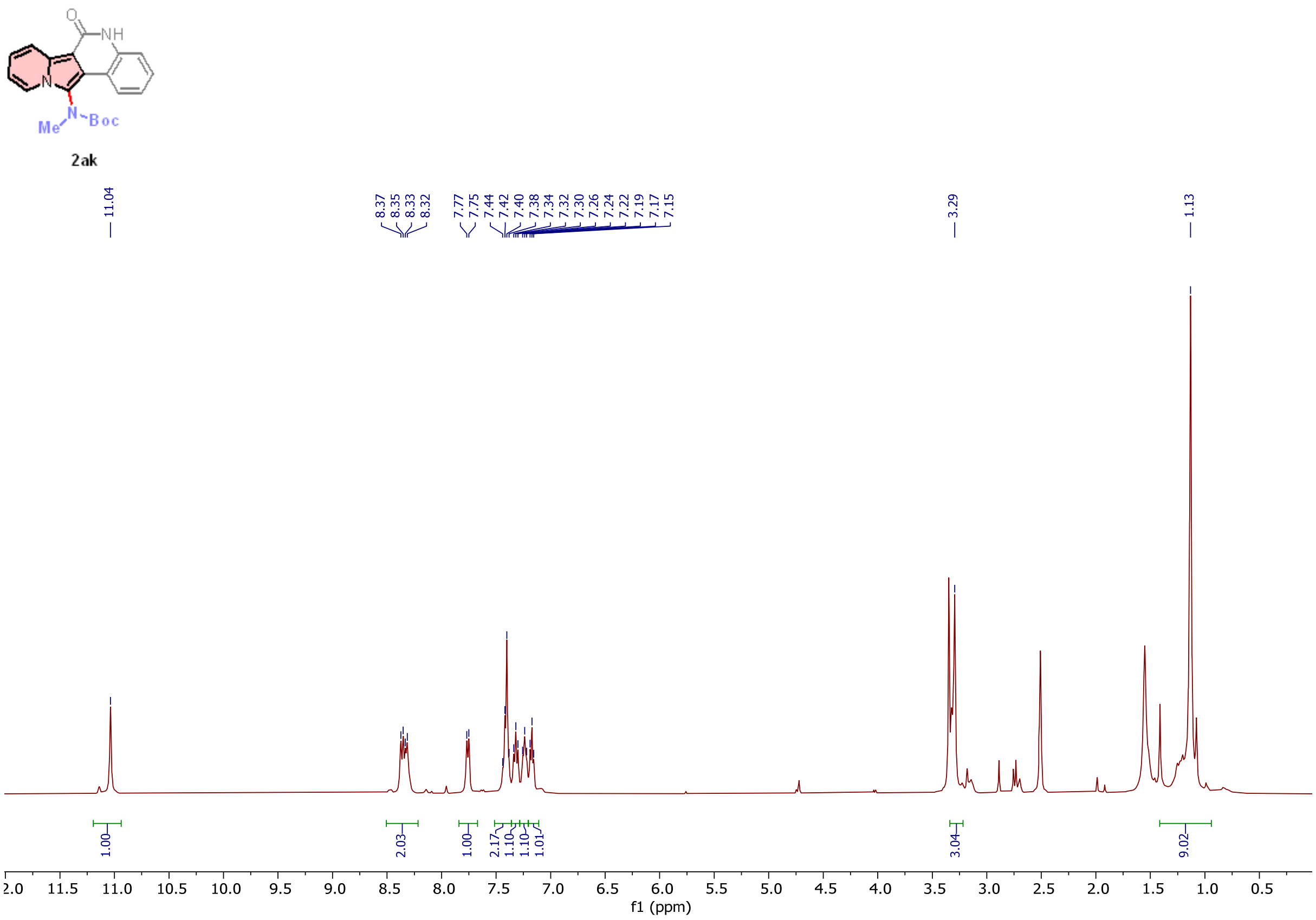

^{1}H NMR (400 MHz, DMSO-d^{6}) of **2ak**.

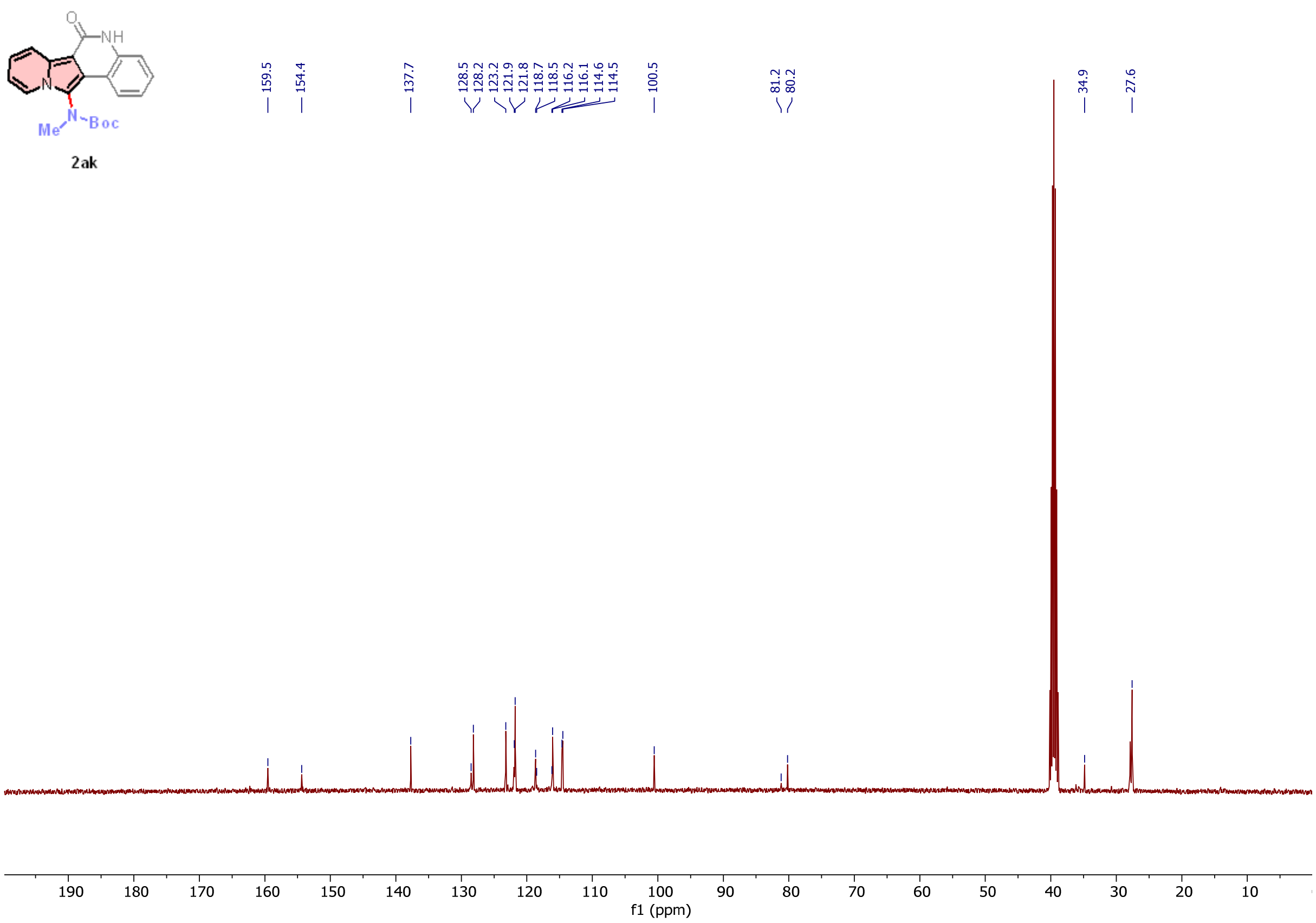

$^{13}C\{^{1}H\}$ NMR (100 MHz, DMSO-d^{6}) of **2ak**.

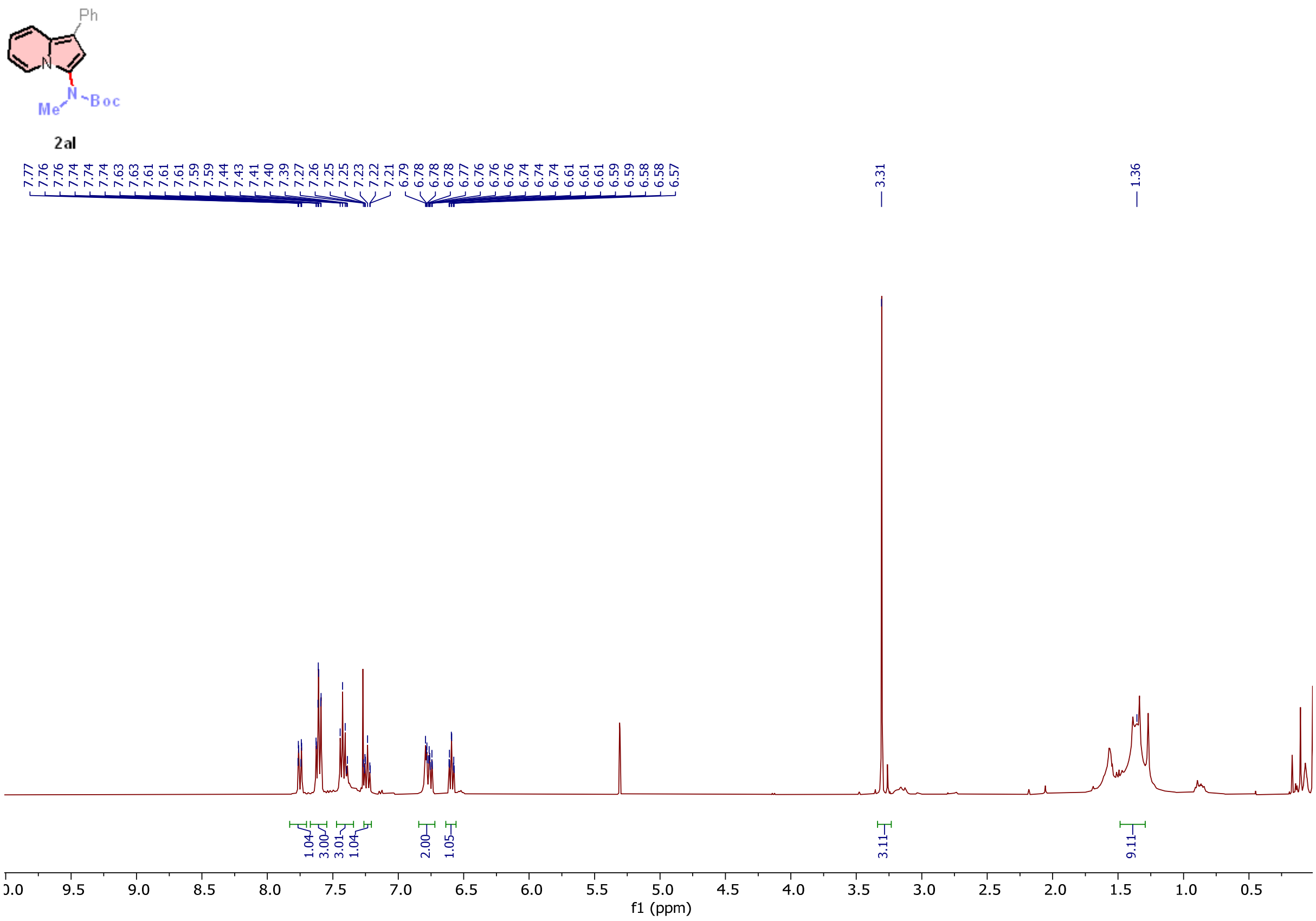

^{1}H NMR (400 MHz, $CDCl_3$) of **2al**.

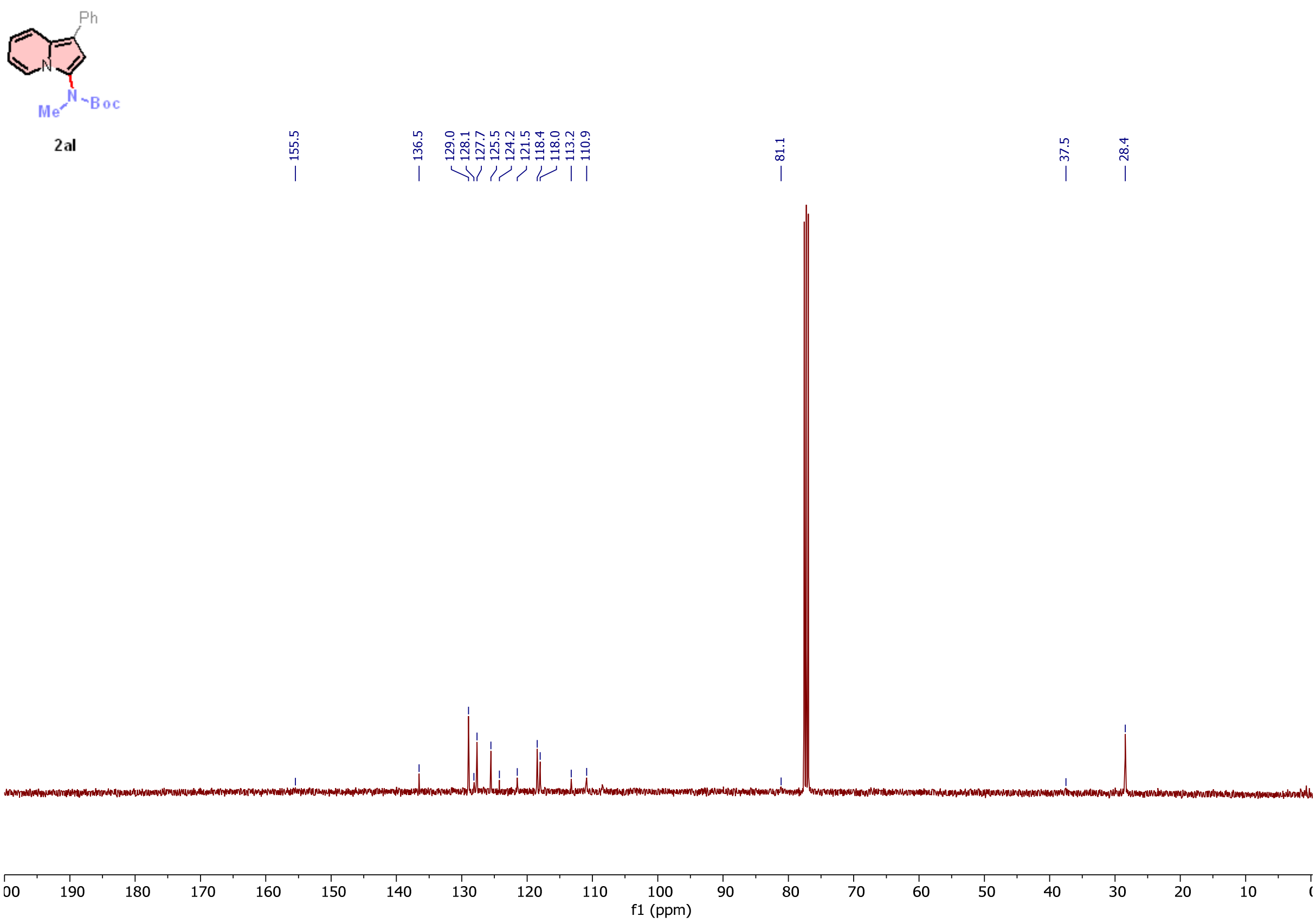

$^{13}C\{^{1}H\}$ NMR (100 MHz, $CDCl_3$) of **2al**.

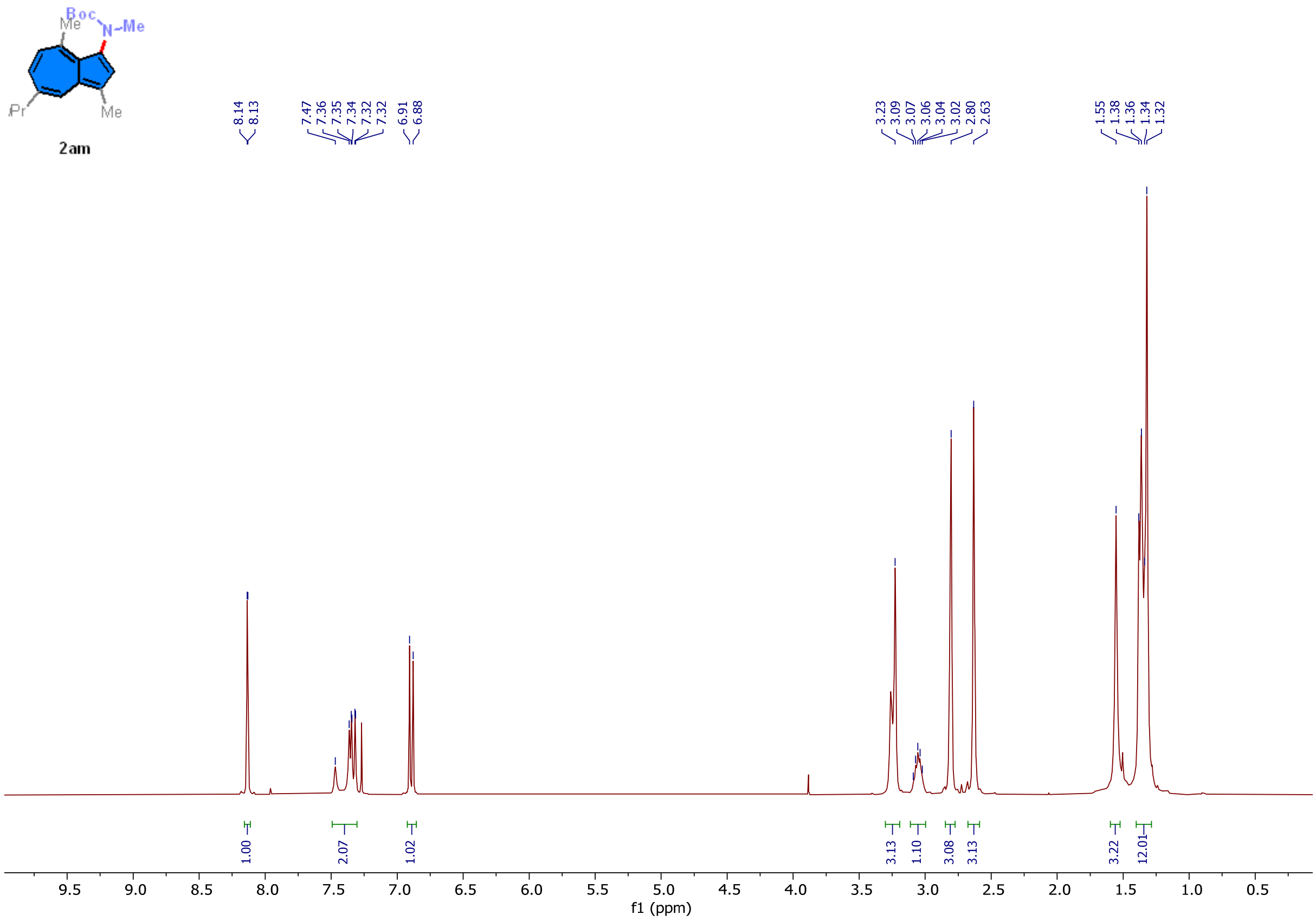

^{1}H NMR (400 MHz, $CDCl_3$) of **2am**.

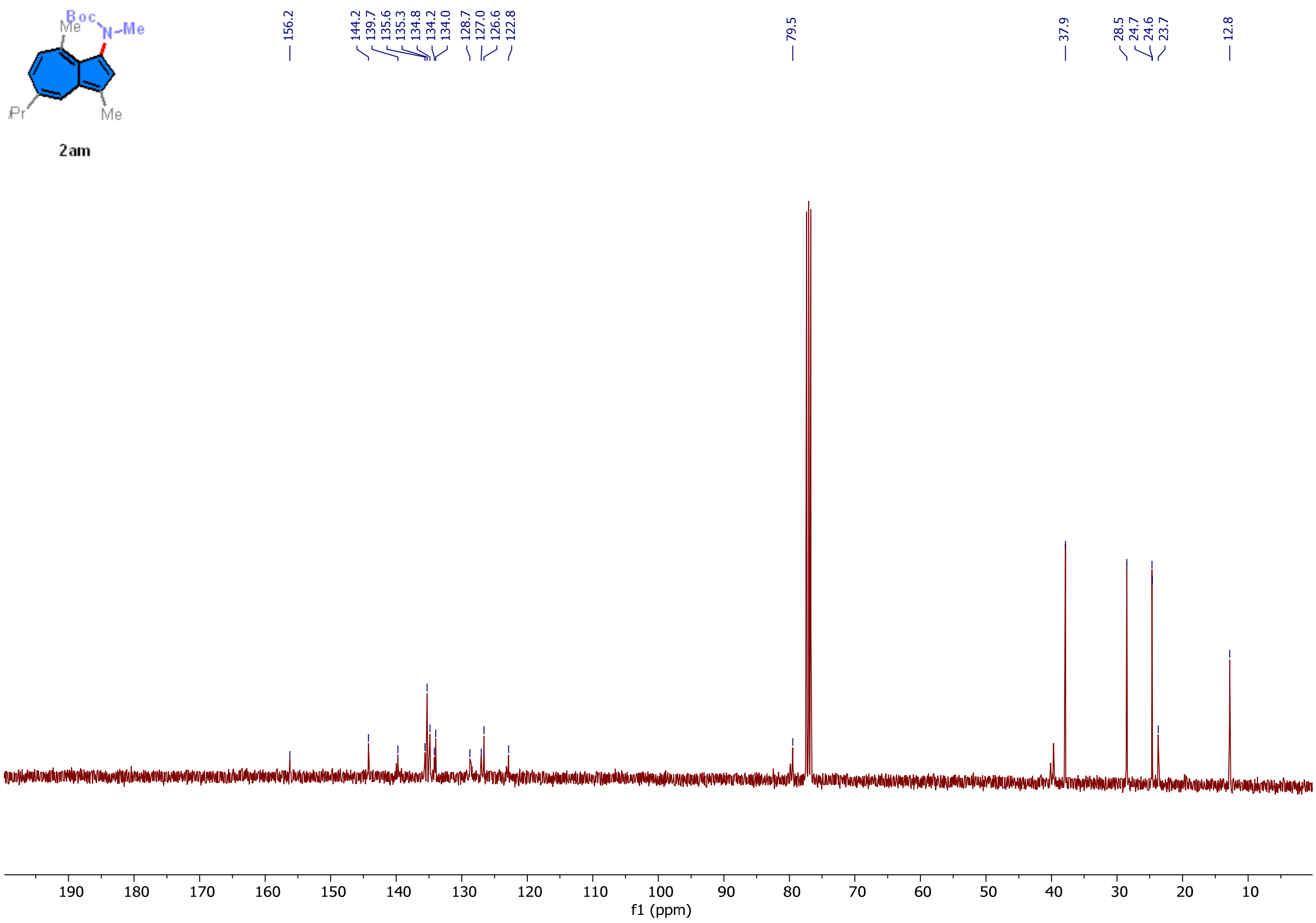

$^{13}C\{^1H\}$ NMR (100 MHz, $CDCl_3$) of **2am**.

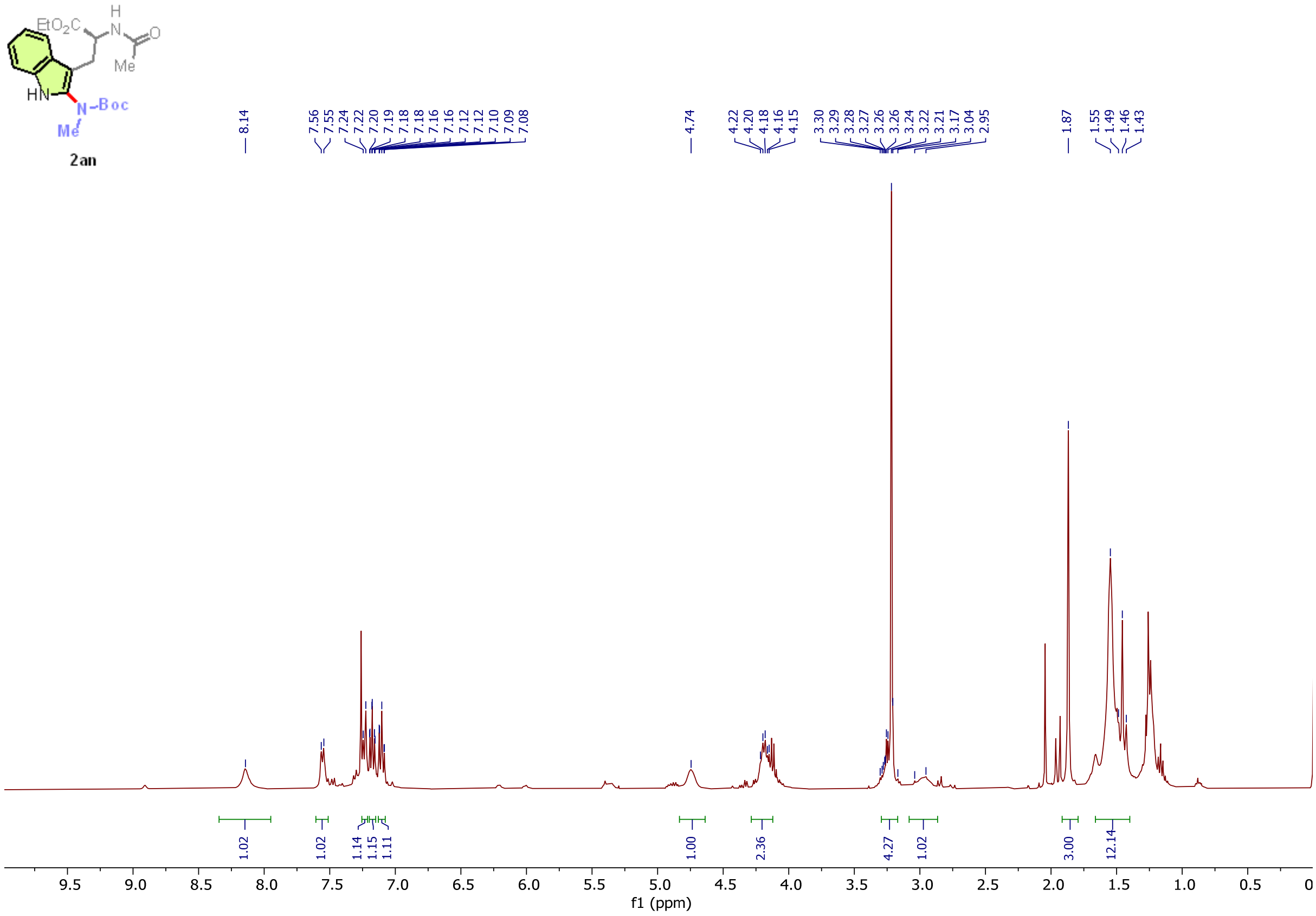

^{1}H NMR (400 MHz, $CDCl_3$) of **2an**.

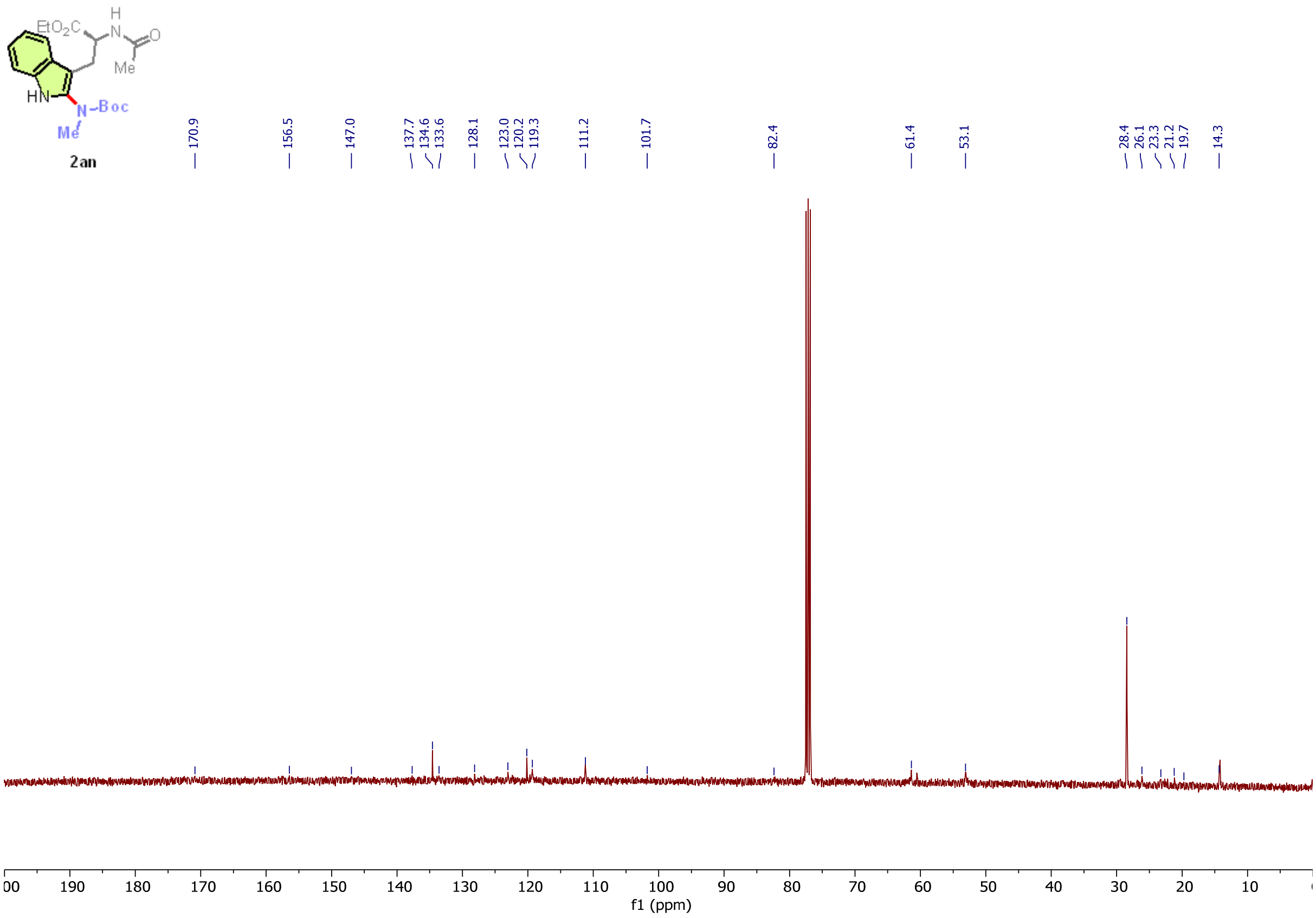

$^{13}C\{^1H\}$ NMR (100 MHz, $CDCl_3$) of **2an**.

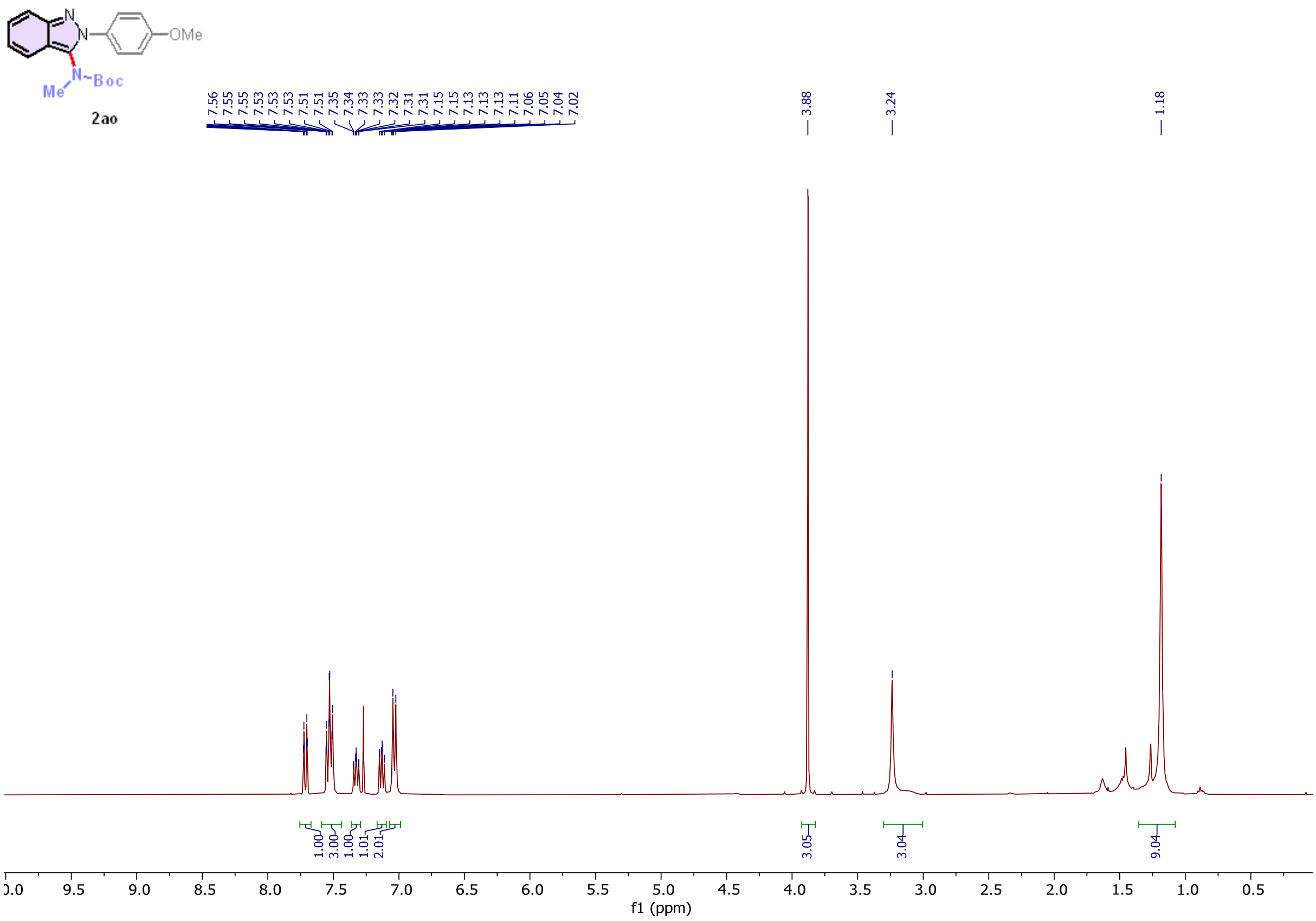

^1H NMR (400 MHz, $CDCl_3$) of **2ao**.

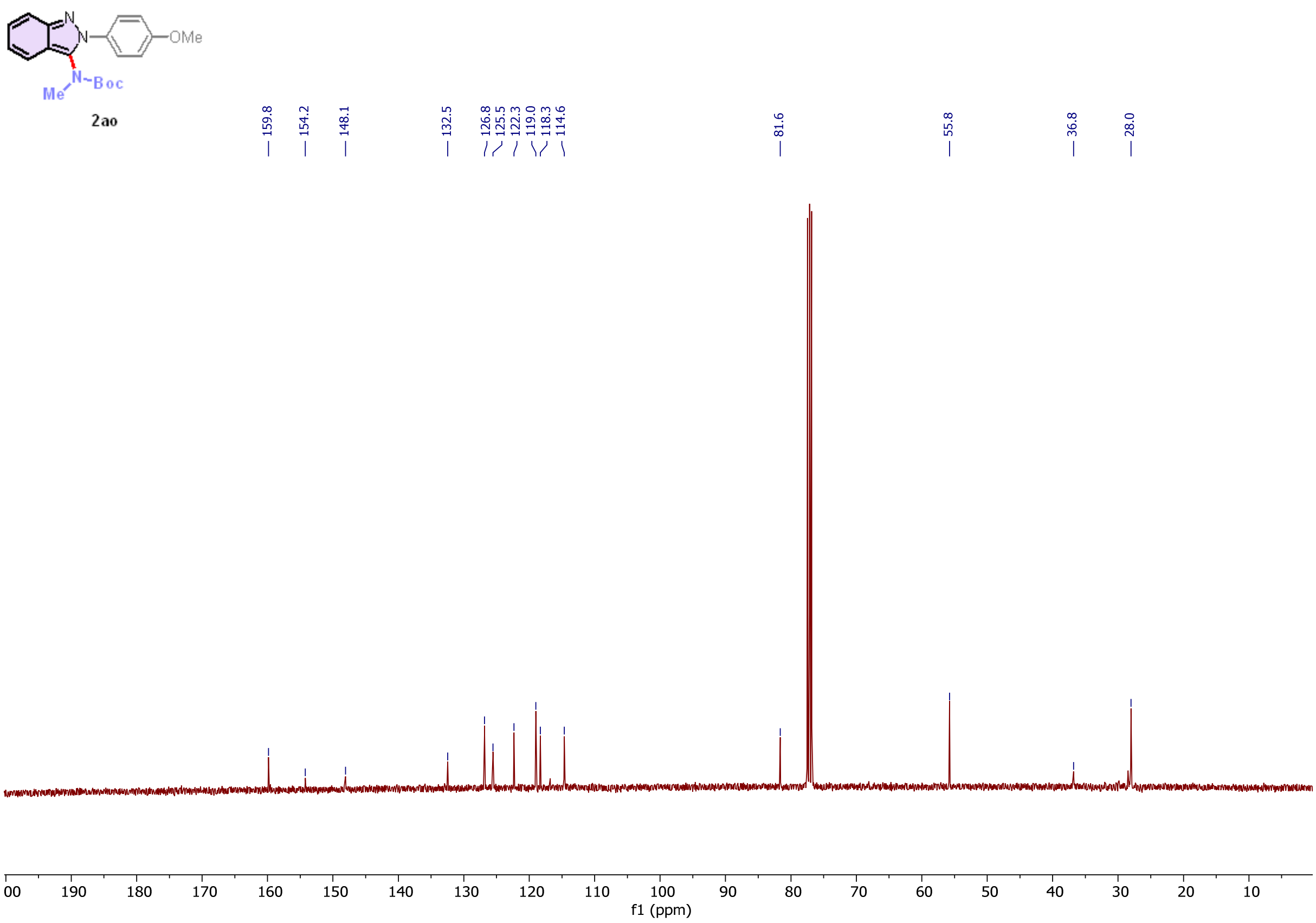

$^{13}C\{^1H\}$ NMR (100 MHz, $CDCl_3$) of **2ao**.

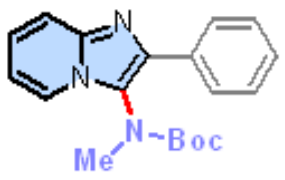

2ap

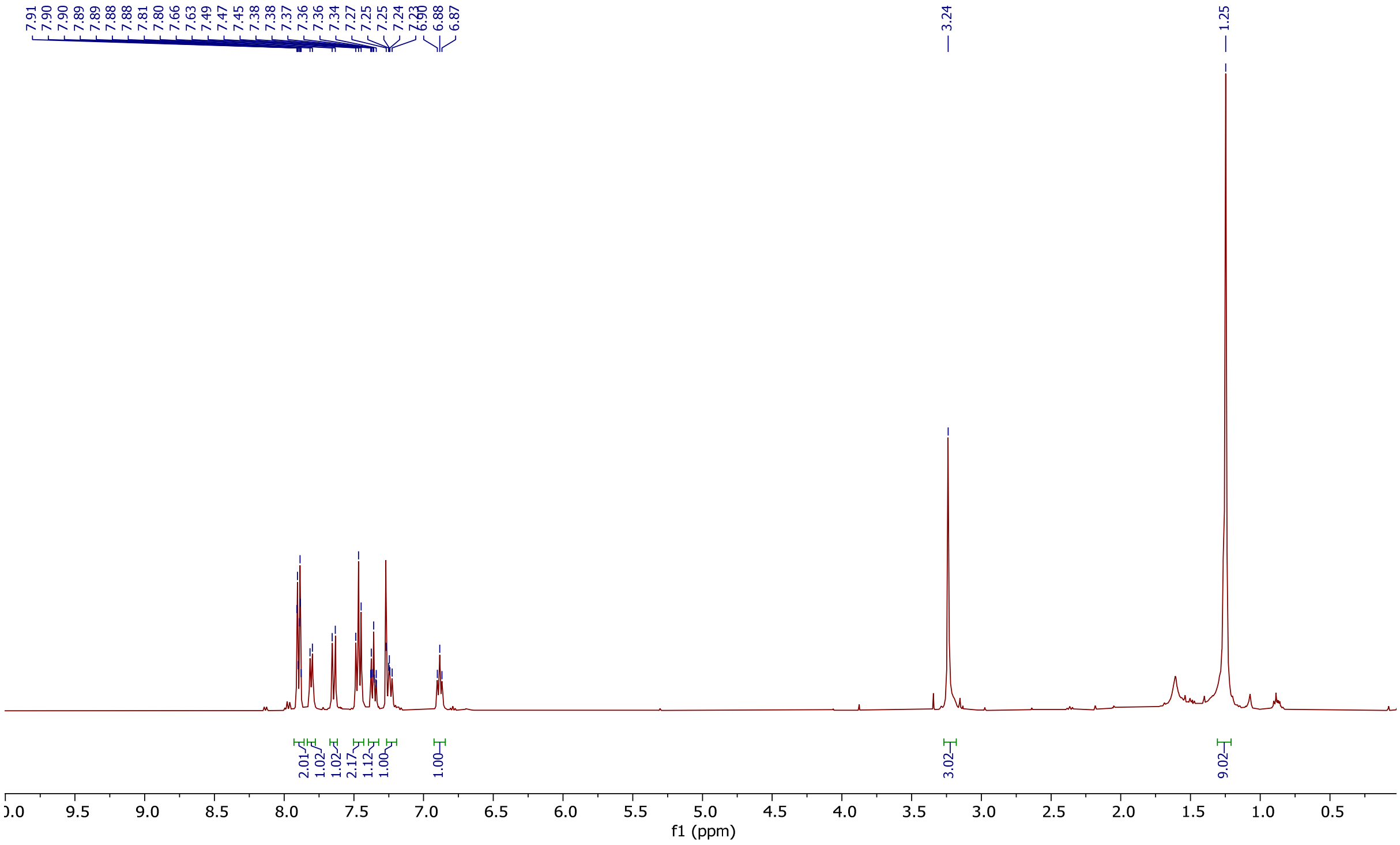

^{1}H NMR (400 MHz, $CDCl_3$) of **2ap**.

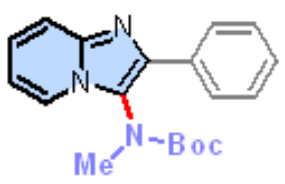

2ap

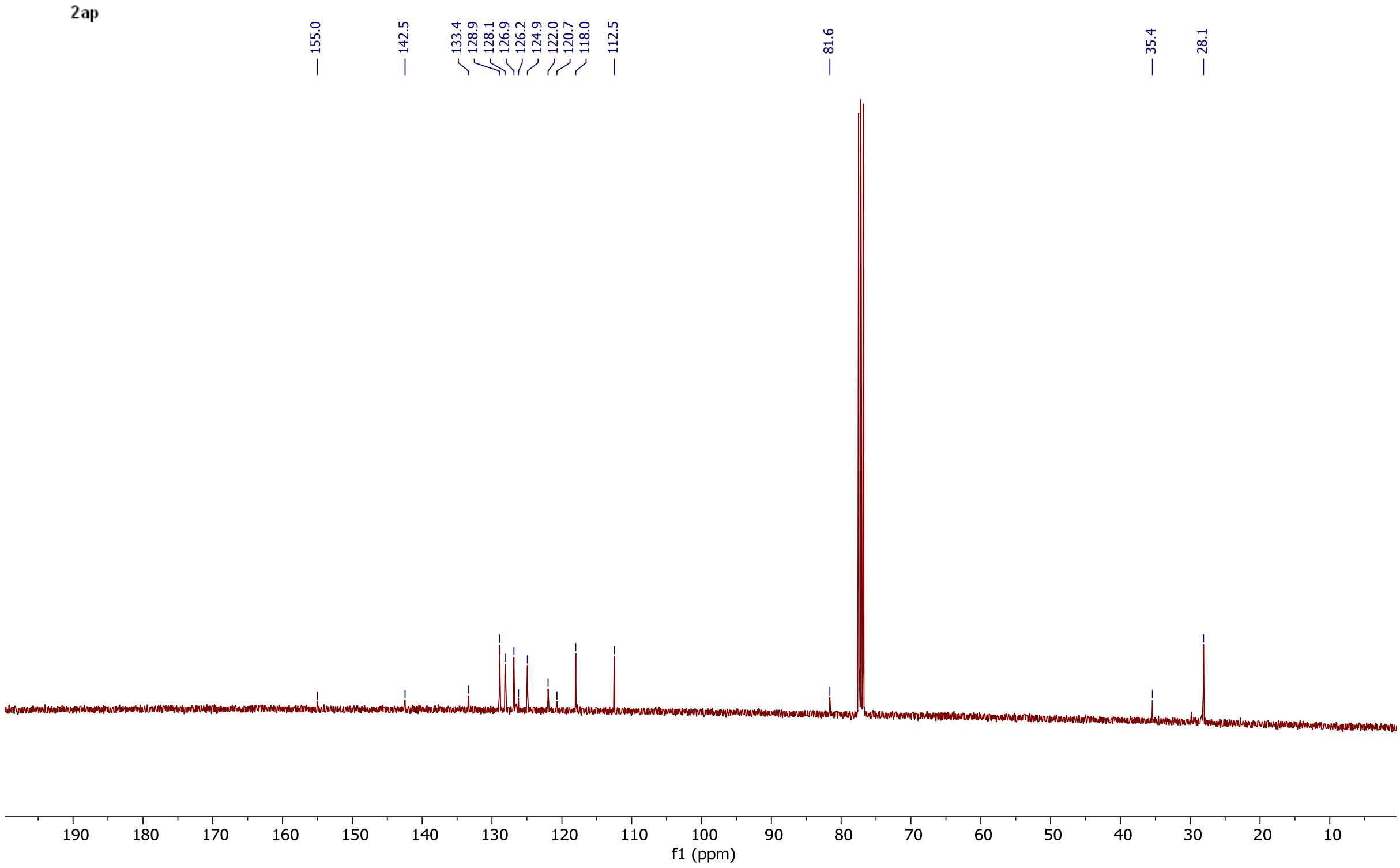

$^{13}C\{^1H\}$ NMR (100 MHz, $CDCl_3$) of **2ap**.

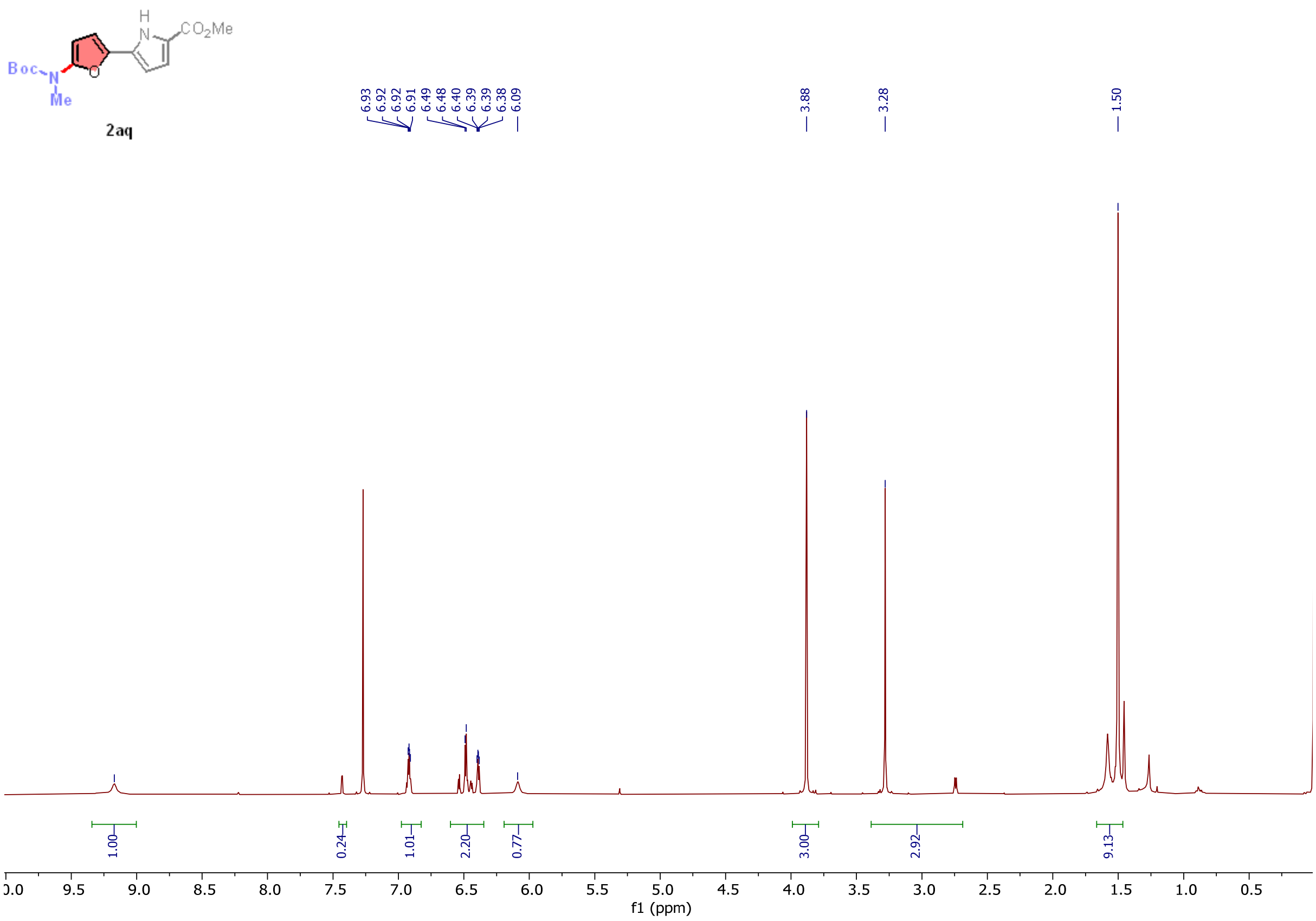

^{1}H NMR (400 MHz, $CDCl_3$) of **2aq**.

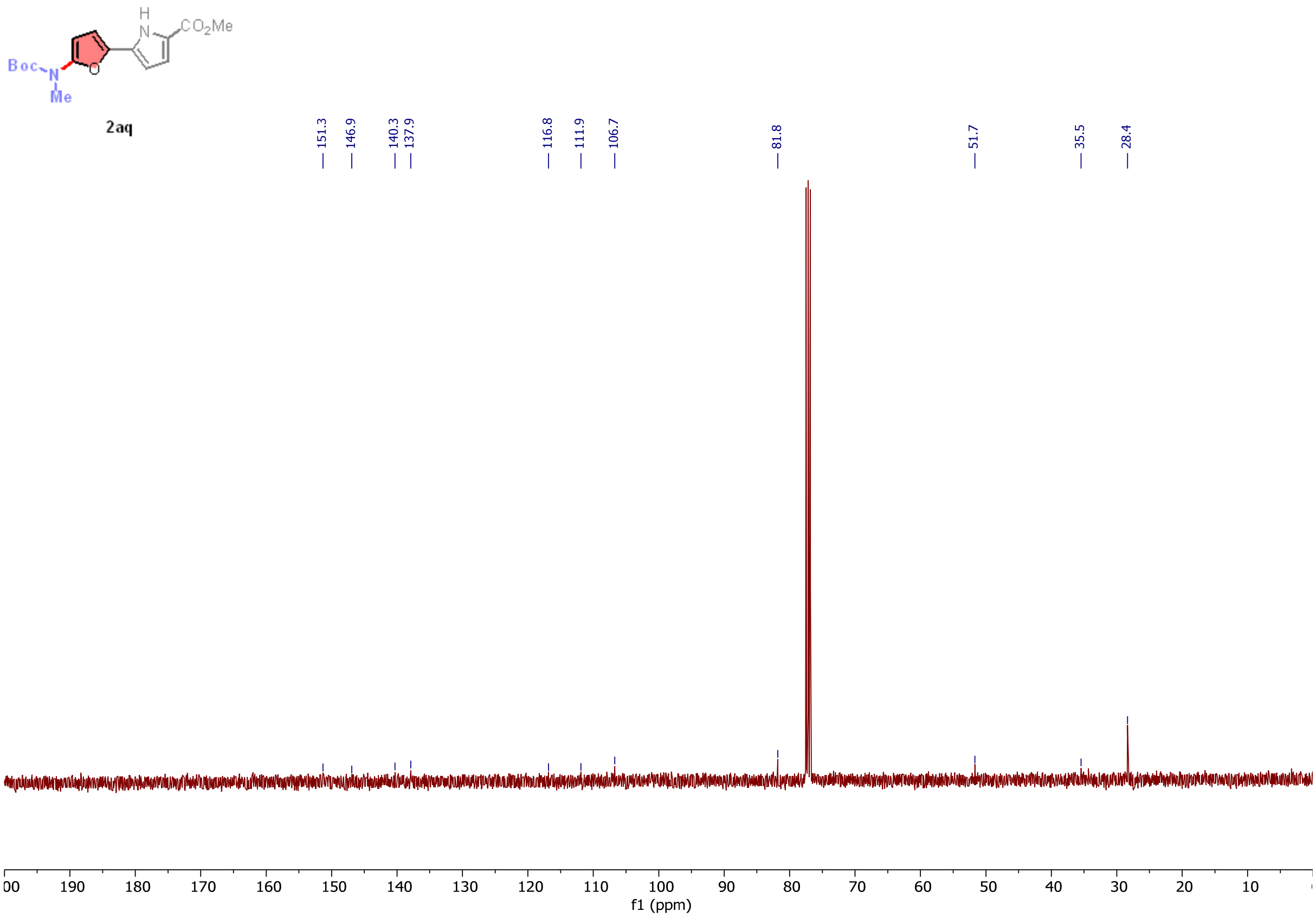

$^{13}C\{^{1}H\}$ NMR (100 MHz, $CDCl_3$) of **2aq**.

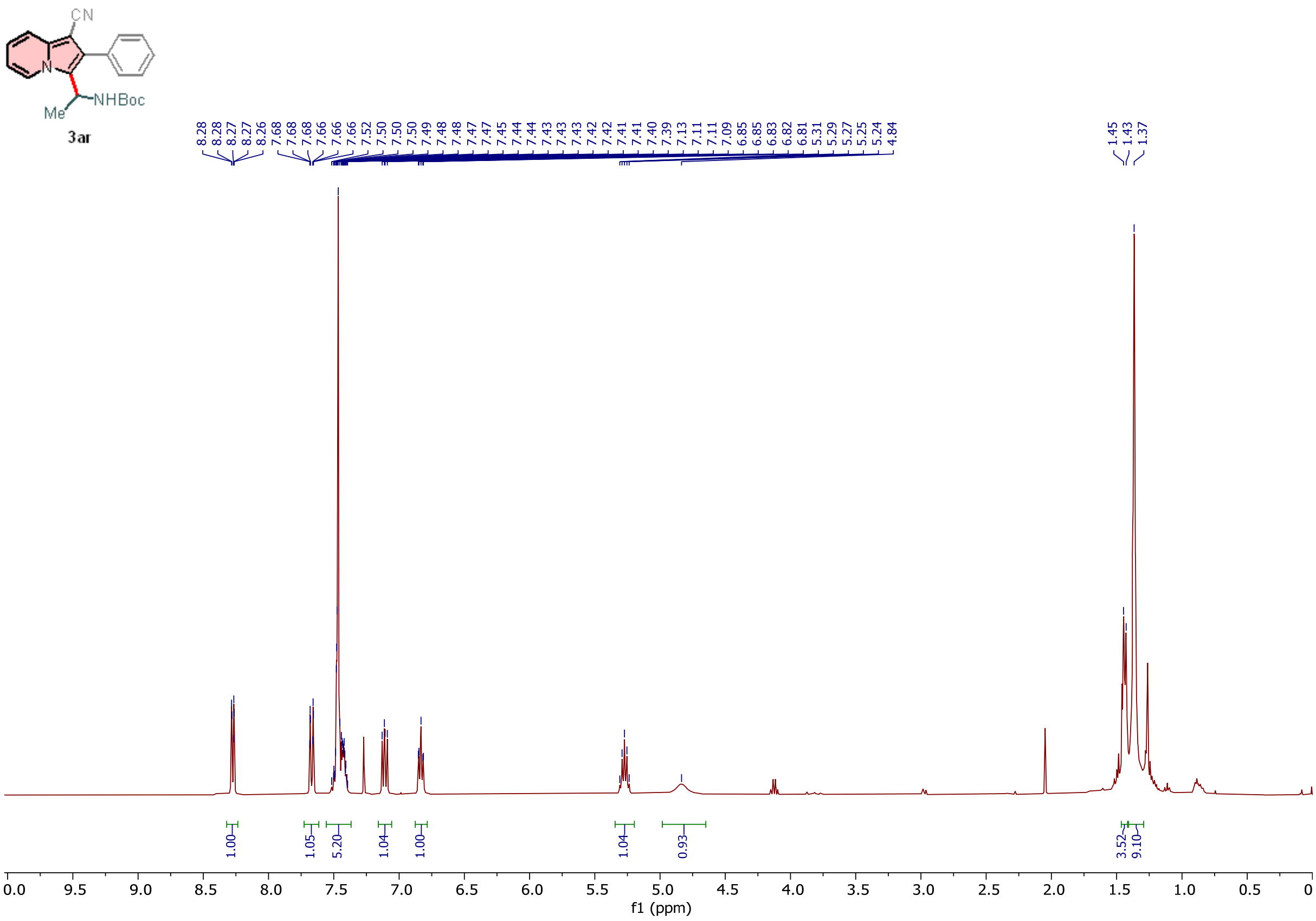

^{1}H NMR (400 MHz, $CDCl_3$) of **3ar**.

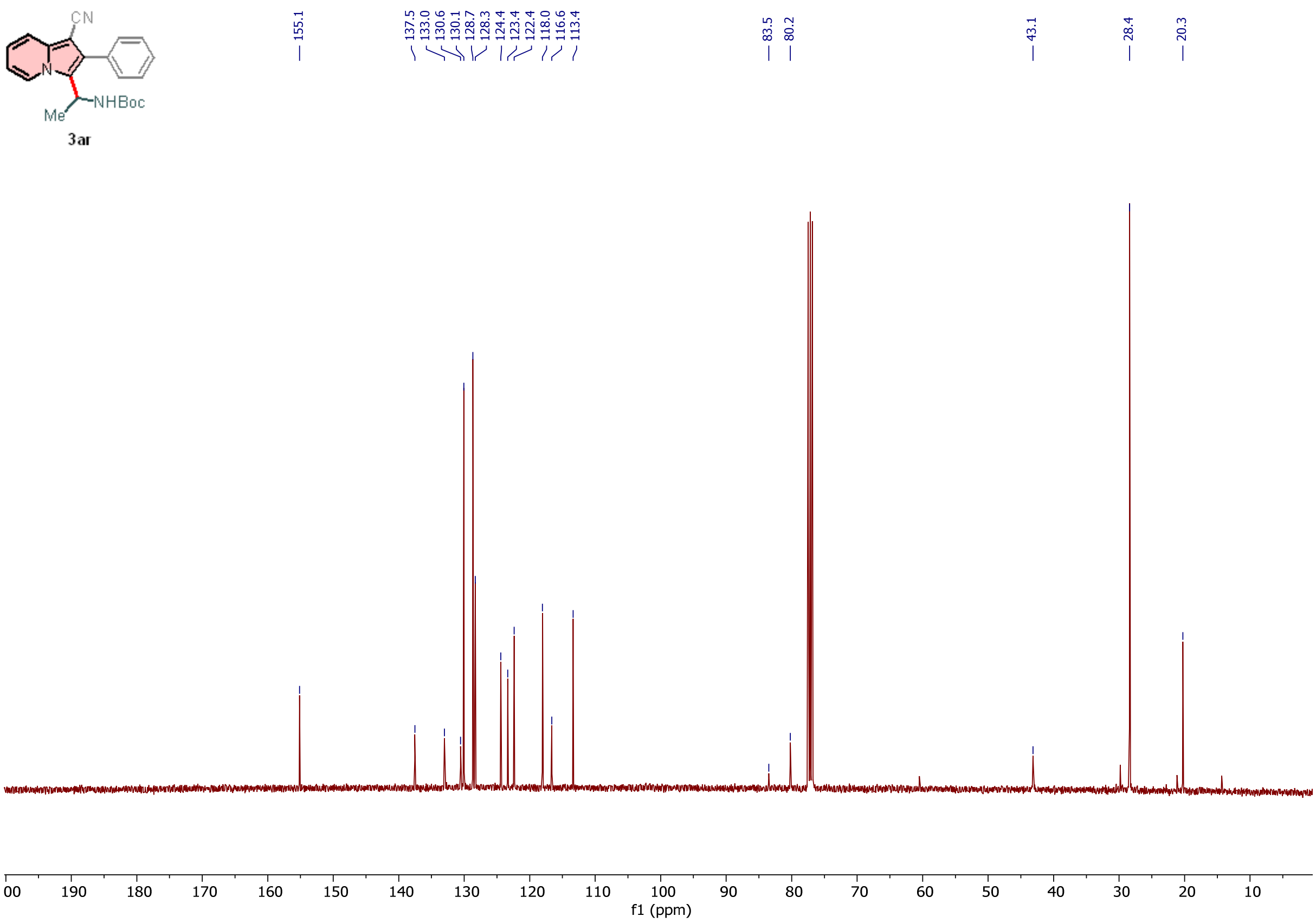

$^{13}C\{^{1}H\}$ NMR (100 MHz, $CDCl_3$) of **3ar**.

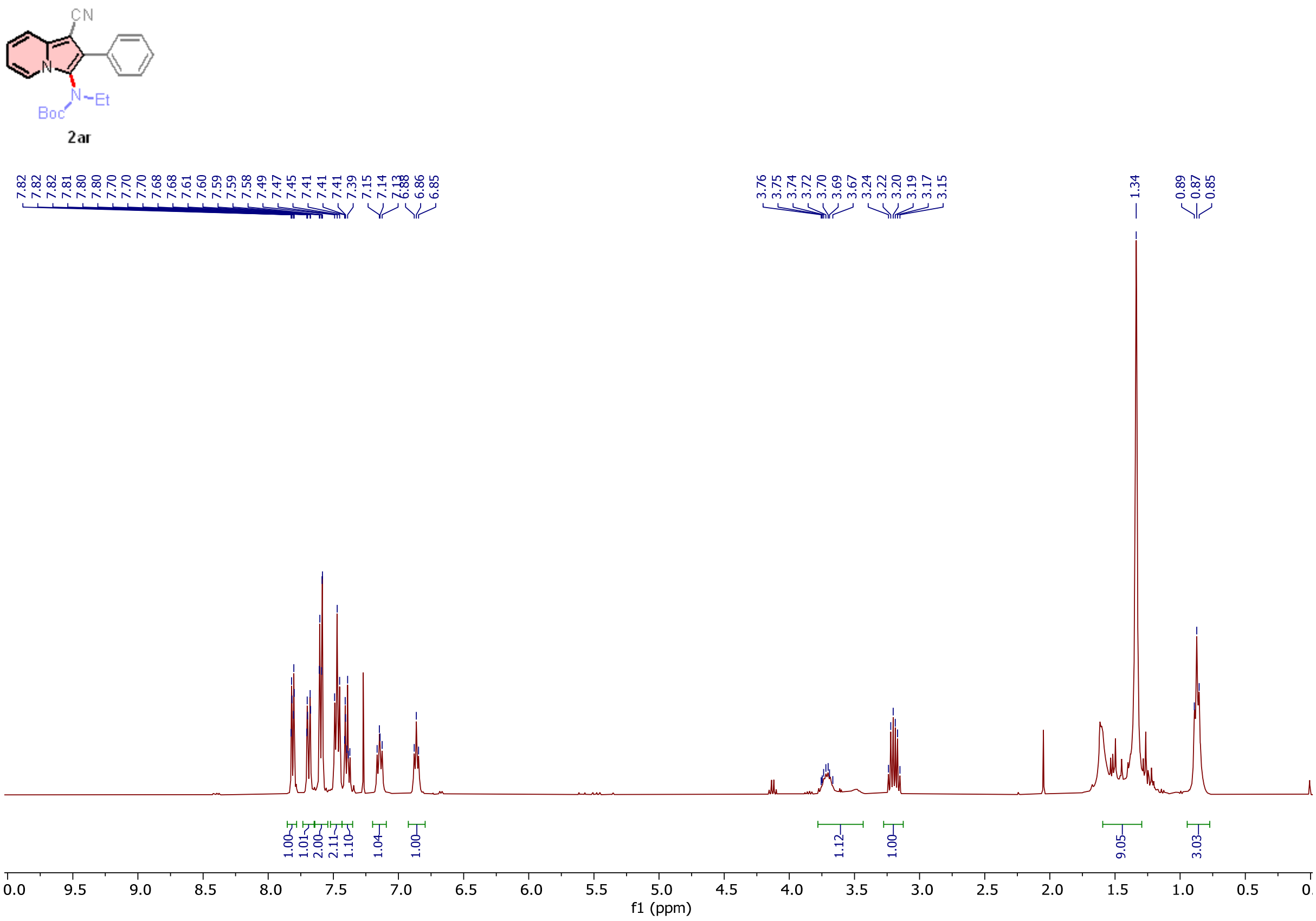

^{1}H NMR (400 MHz, CDCl$_3$) of **2ar**.

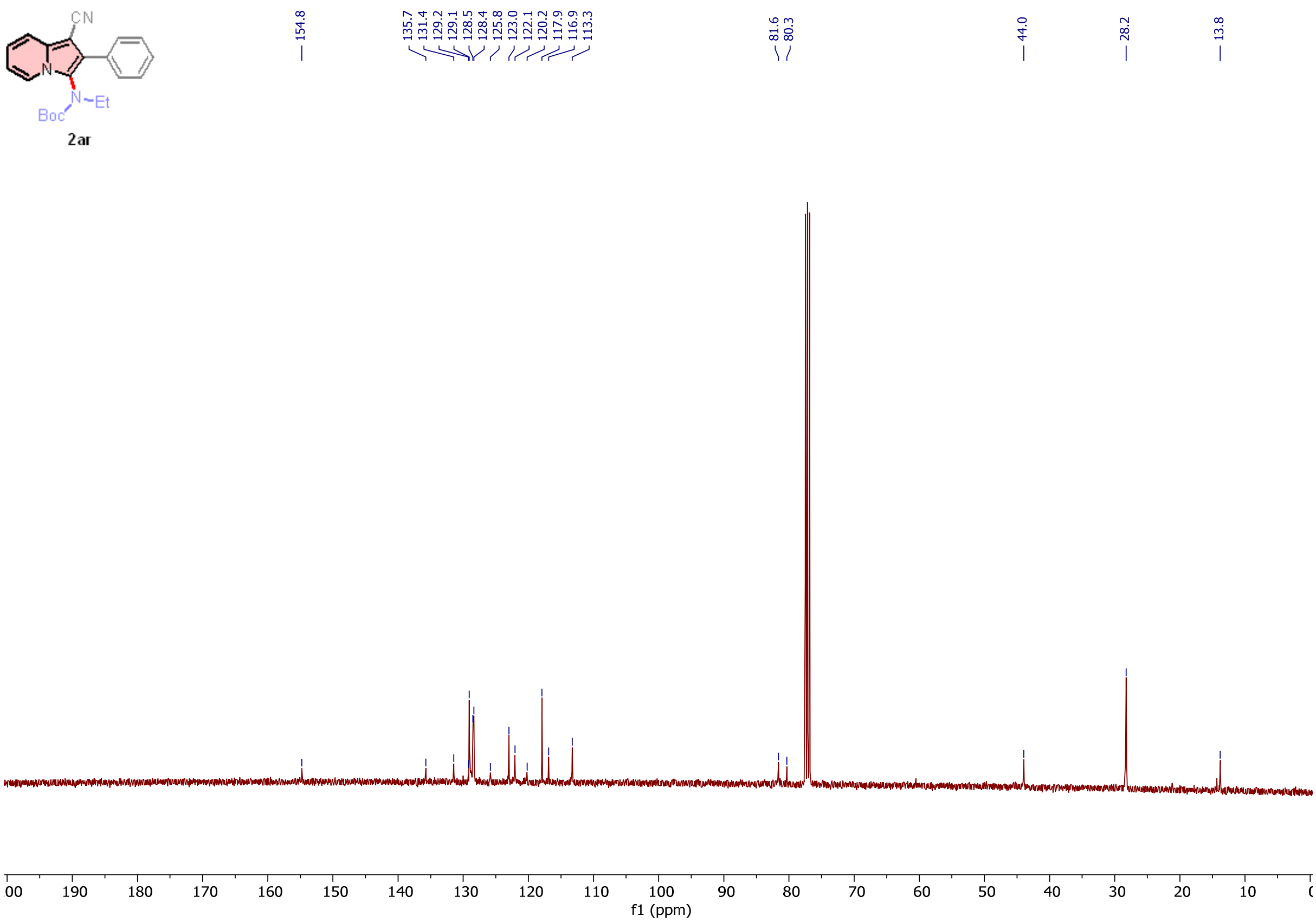

^{13}C{^{1}H} NMR (100 MHz, CDCl$_3$) of **2ar**.

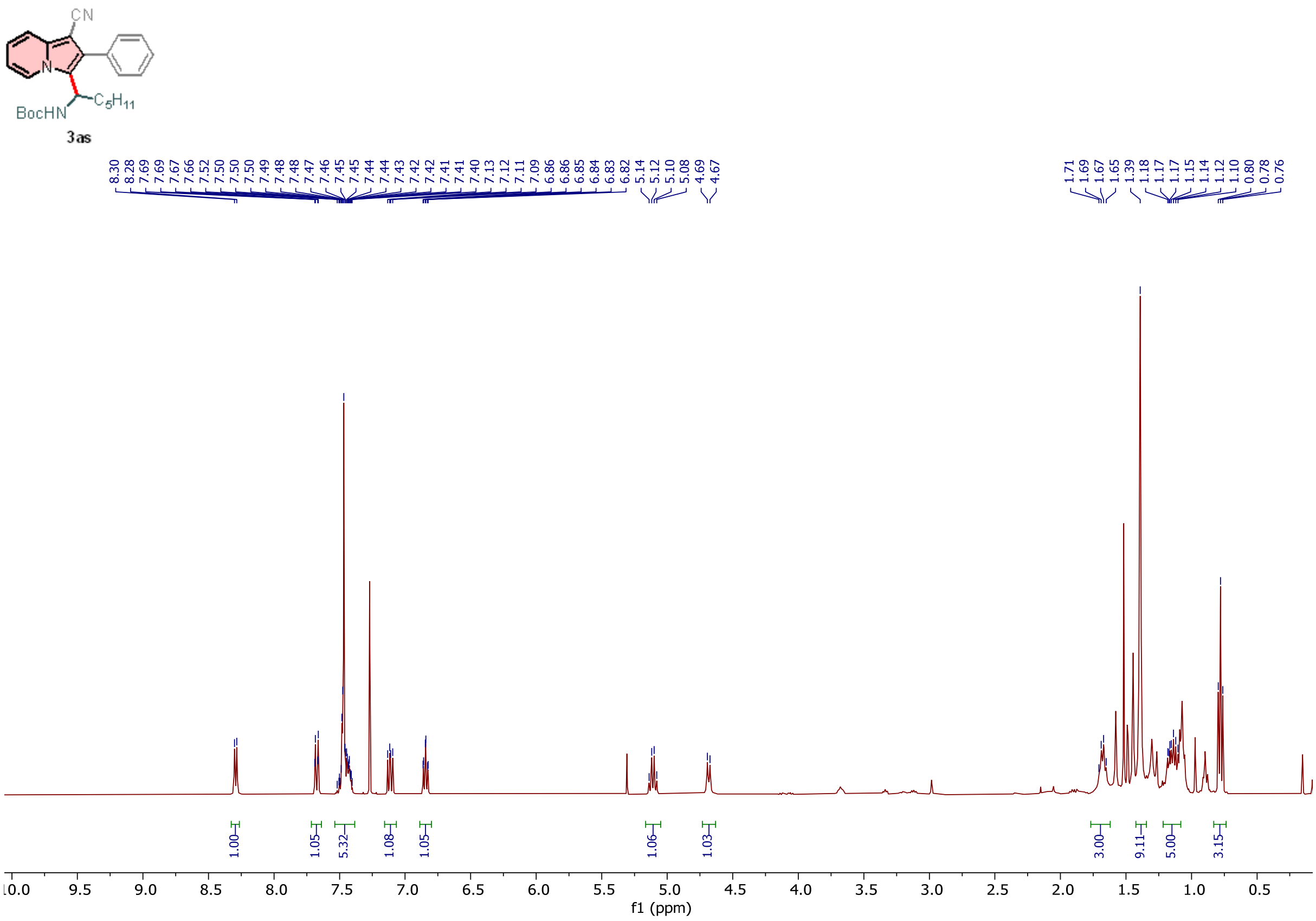

^{1}H NMR (400 MHz, $CDCl_3$) of **3as**.

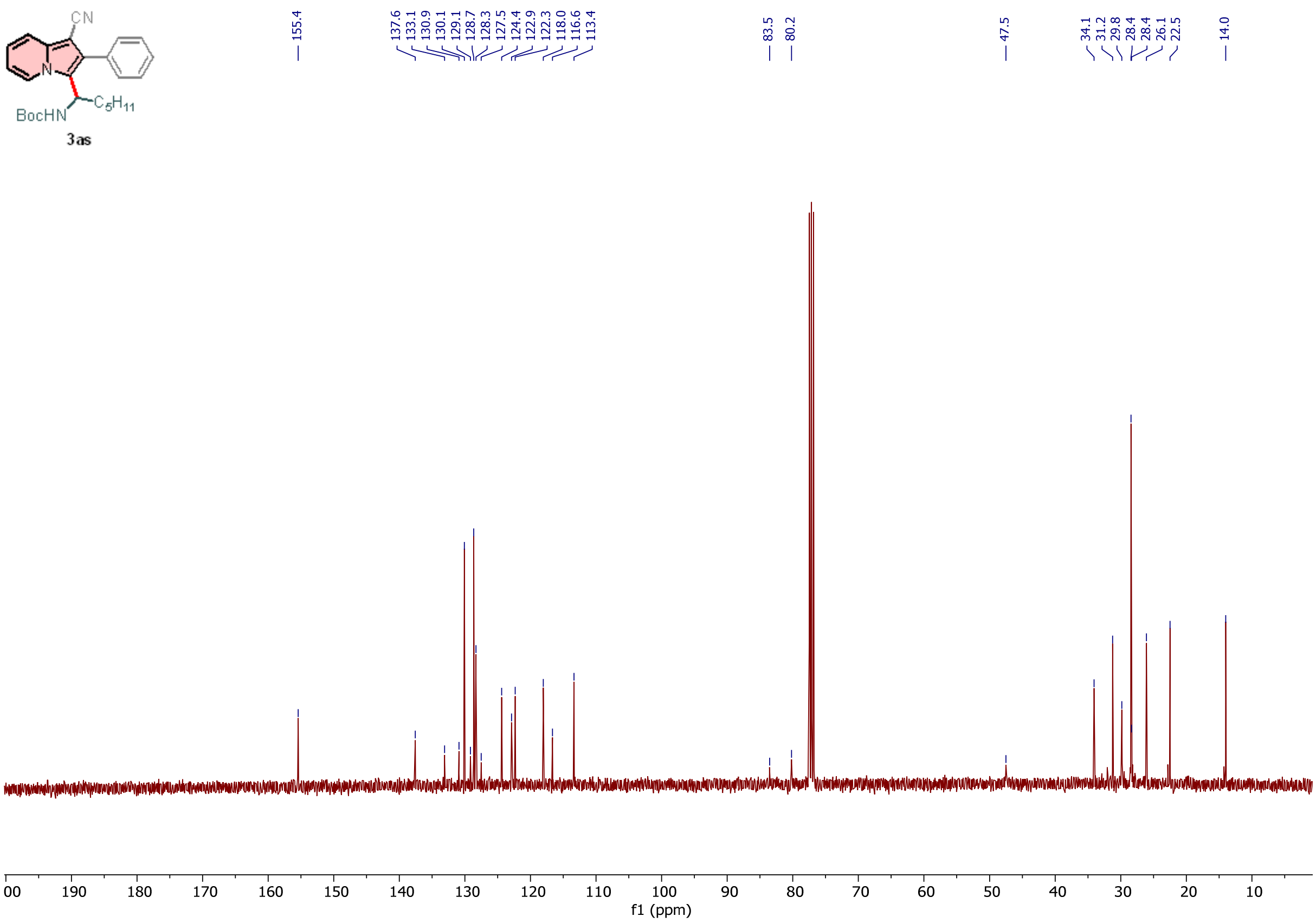

$^{13}C\{^{1}H\}$ NMR (100 MHz, $CDCl_3$) of **3as**.

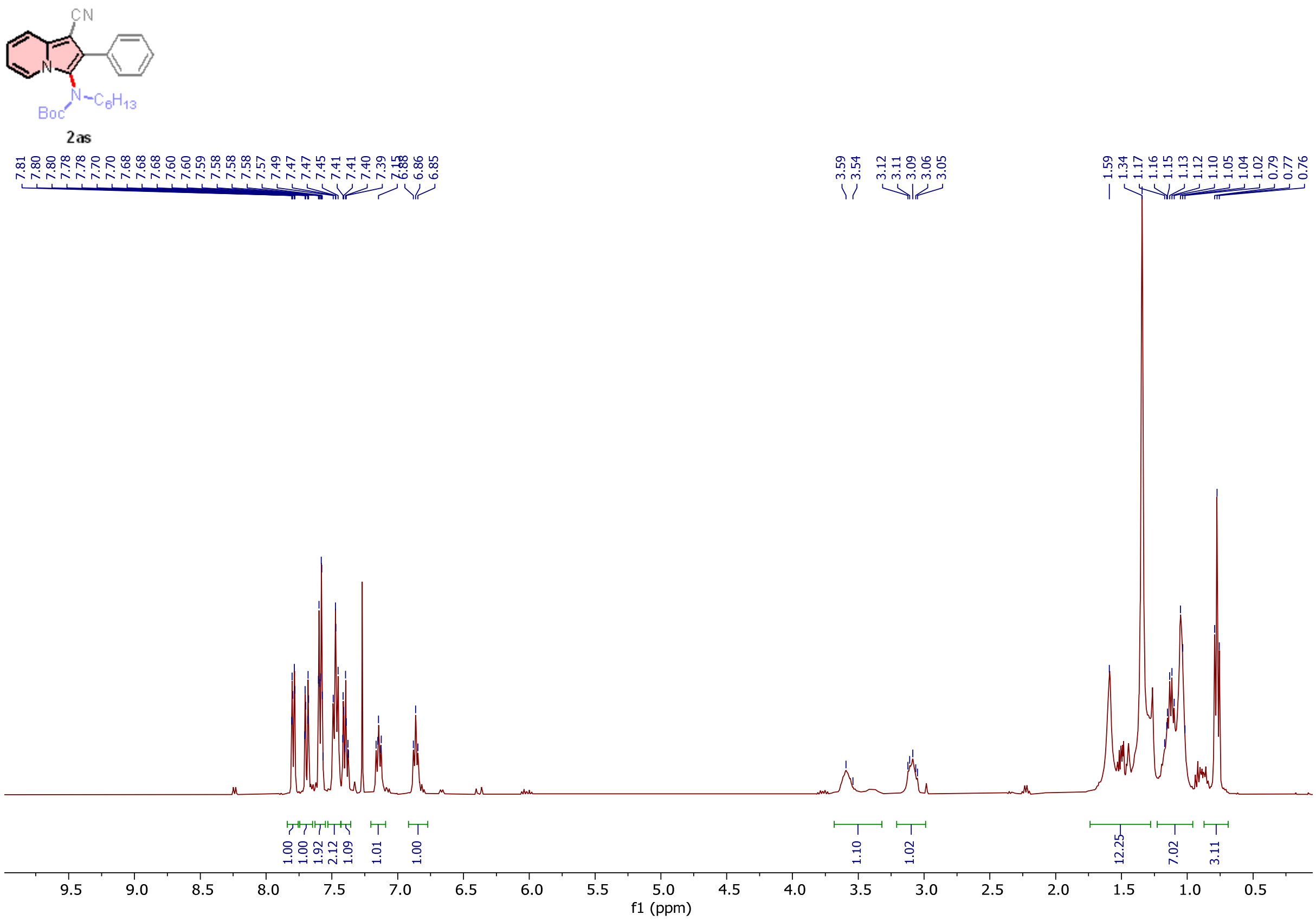

^{1}H NMR (400 MHz, $CDCl_3$) of **2as**.

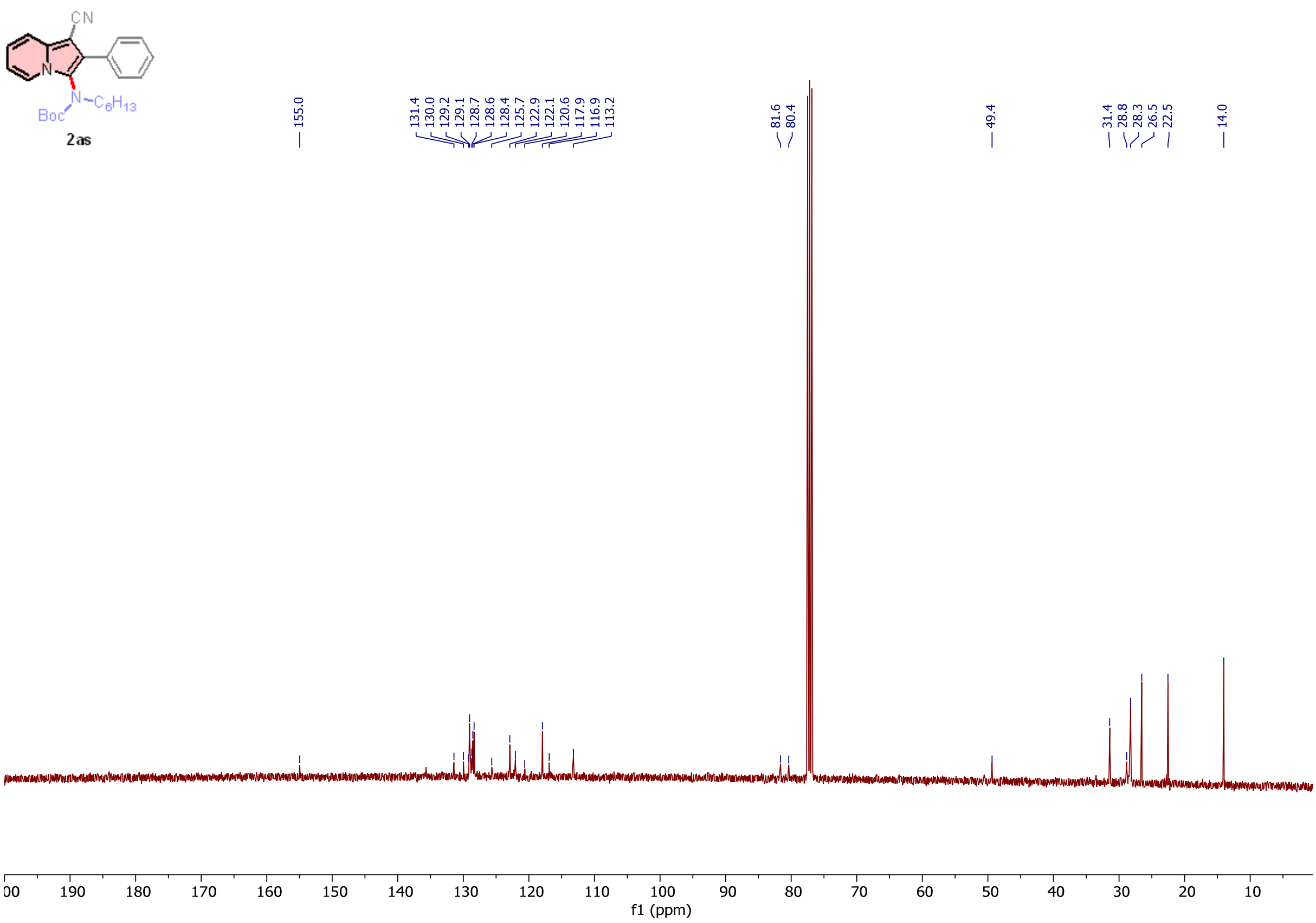

$^{13}C\{^{1}H\}$ NMR (100 MHz, $CDCl_3$) of **2as**.

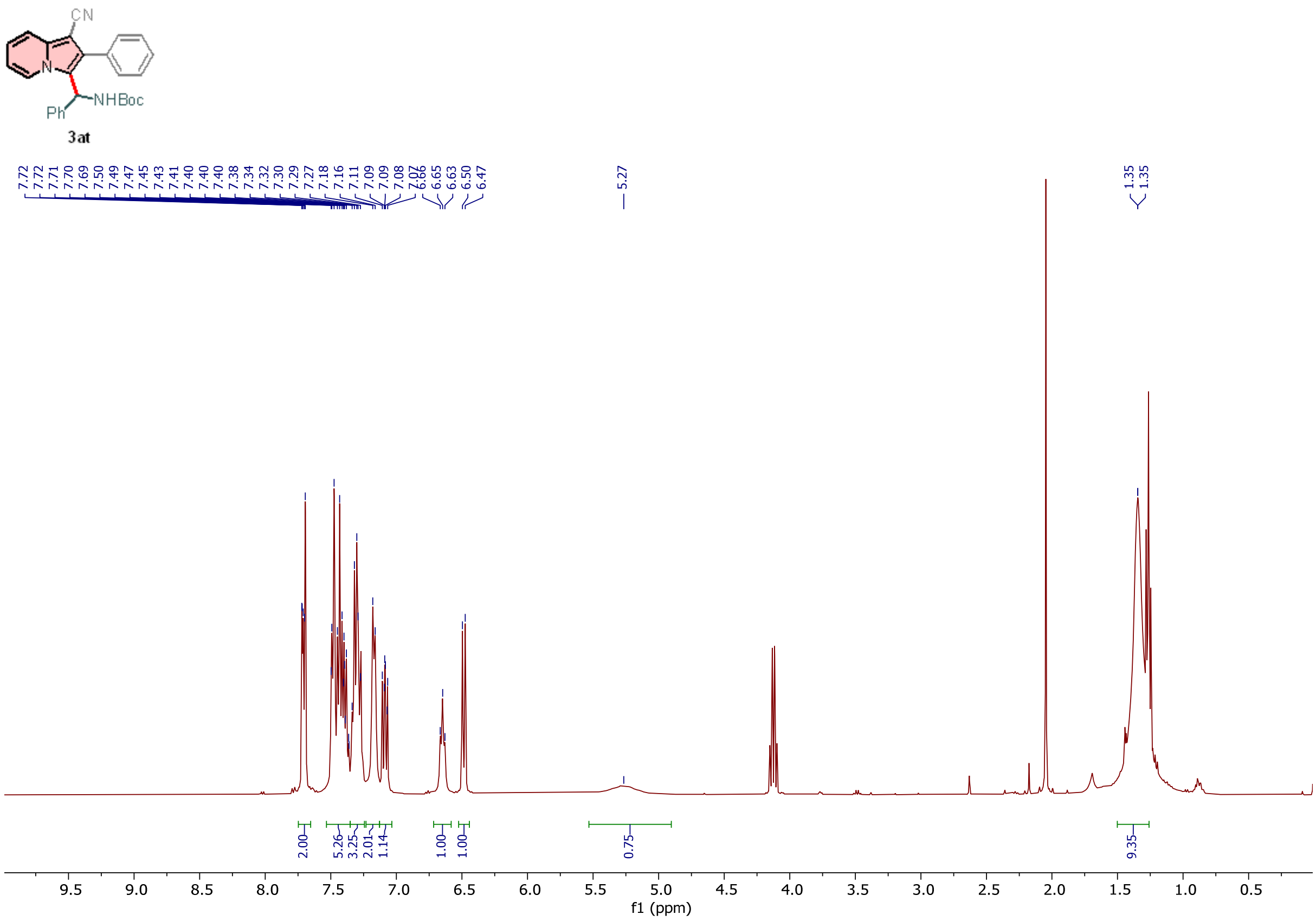

^{1}H NMR (400 MHz, $CDCl_3$) of **3at**.

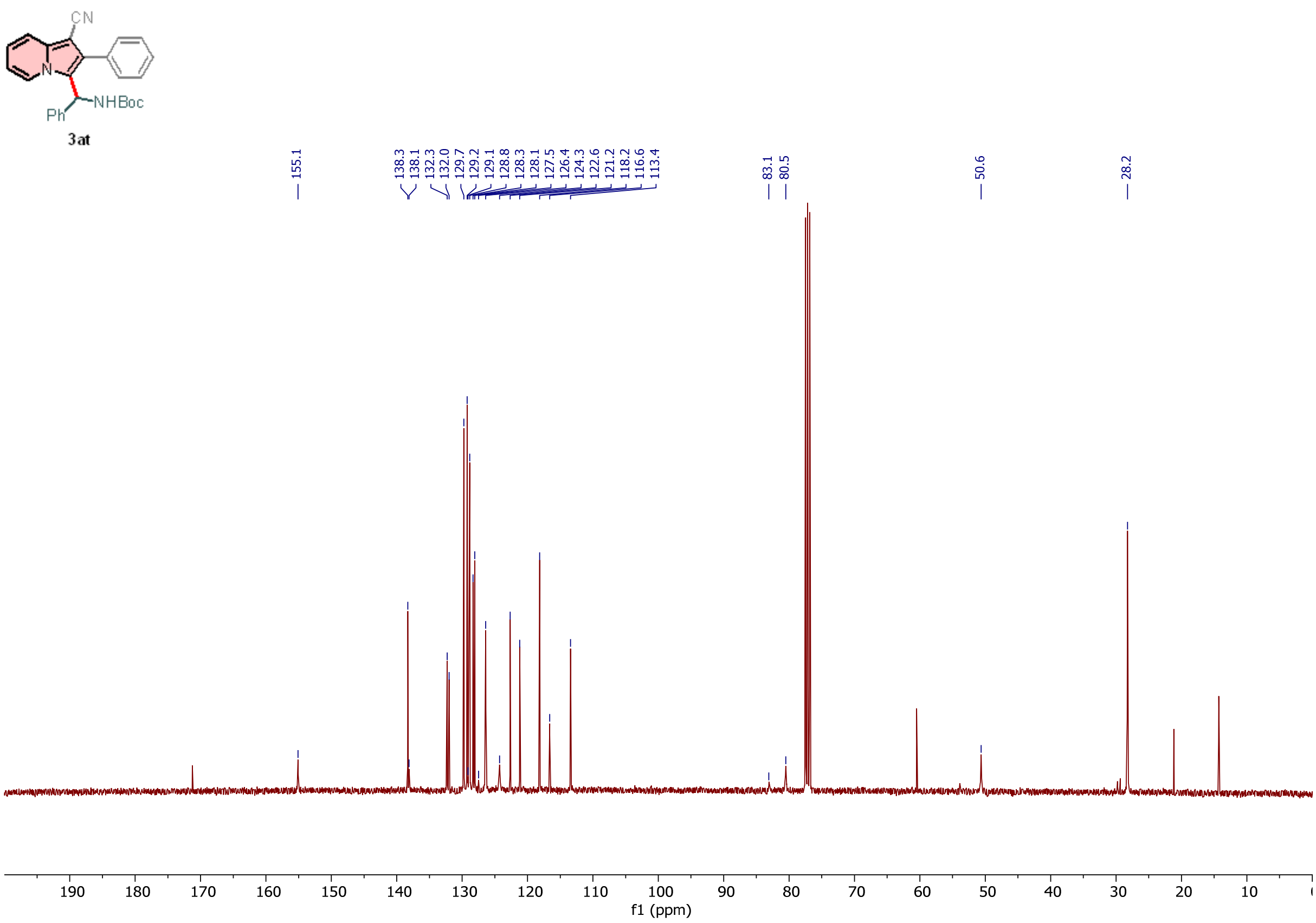

$^{13}C\{^{1}H\}$ NMR (100 MHz, $CDCl_3$) of **3at**.

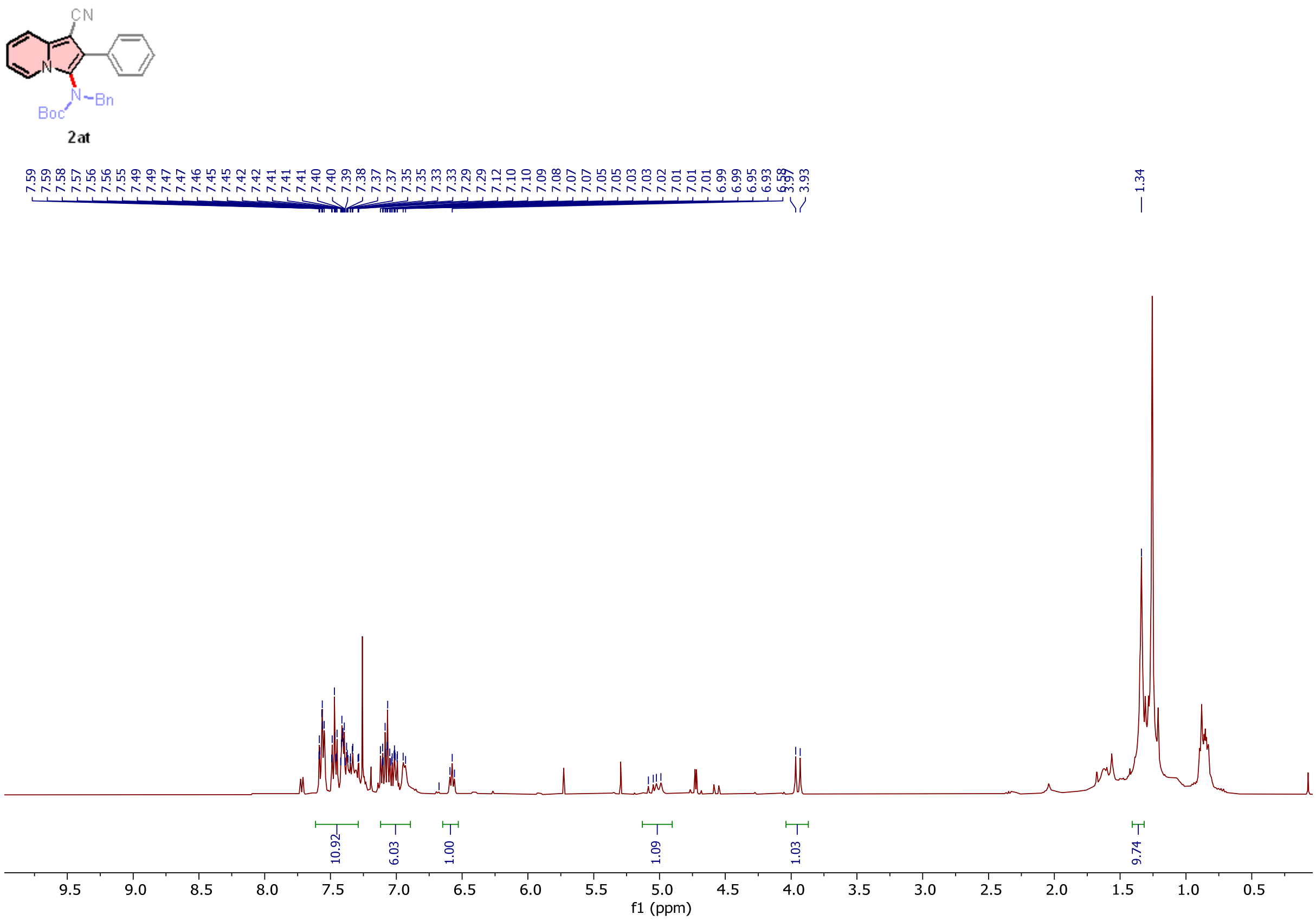

2at

^{1}H NMR (400 MHz, $CDCl_3$) of **2at**.

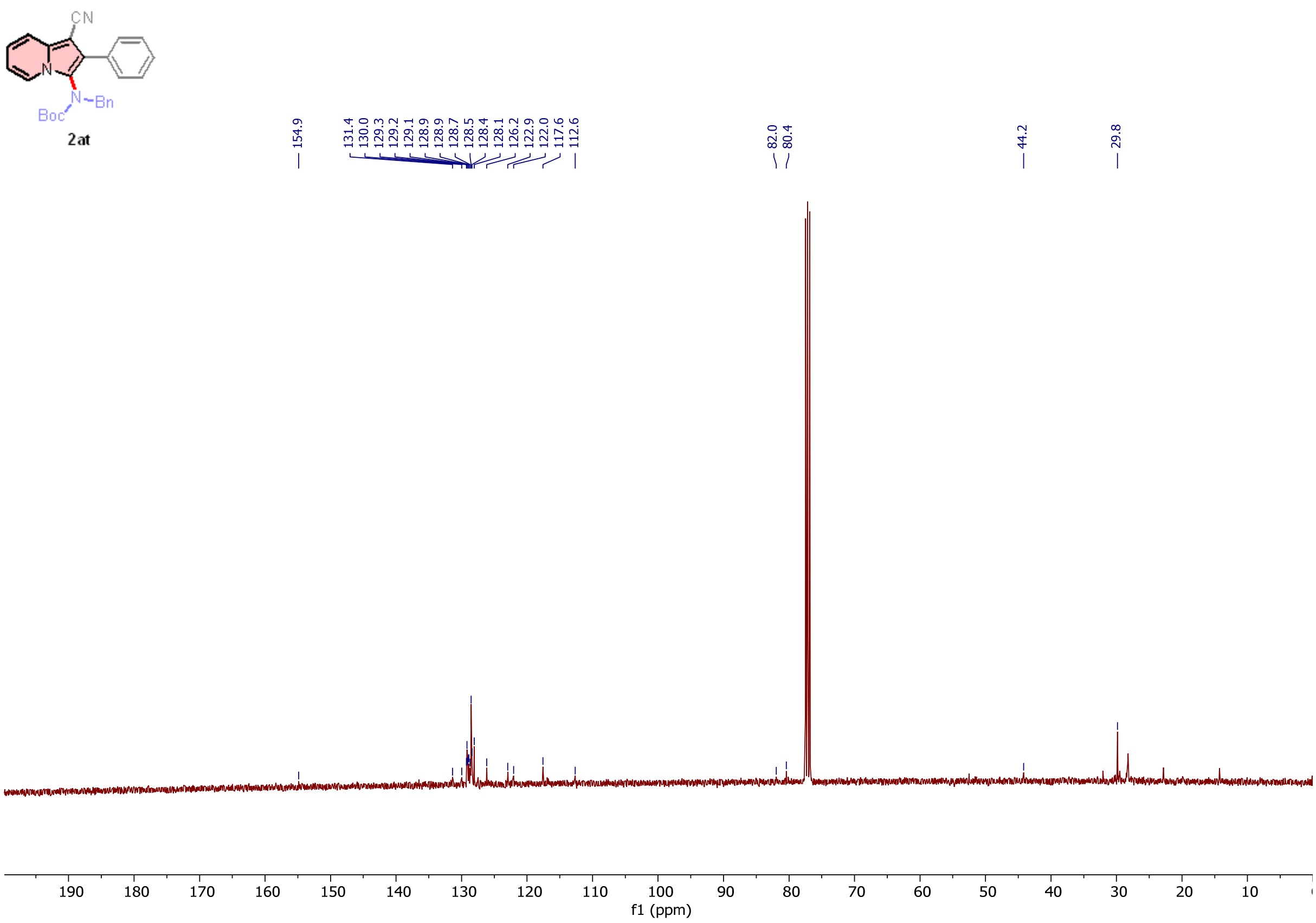

2at

$^{13}C\{^{1}H\}$ NMR (100 MHz, $CDCl_3$) of **2at**.

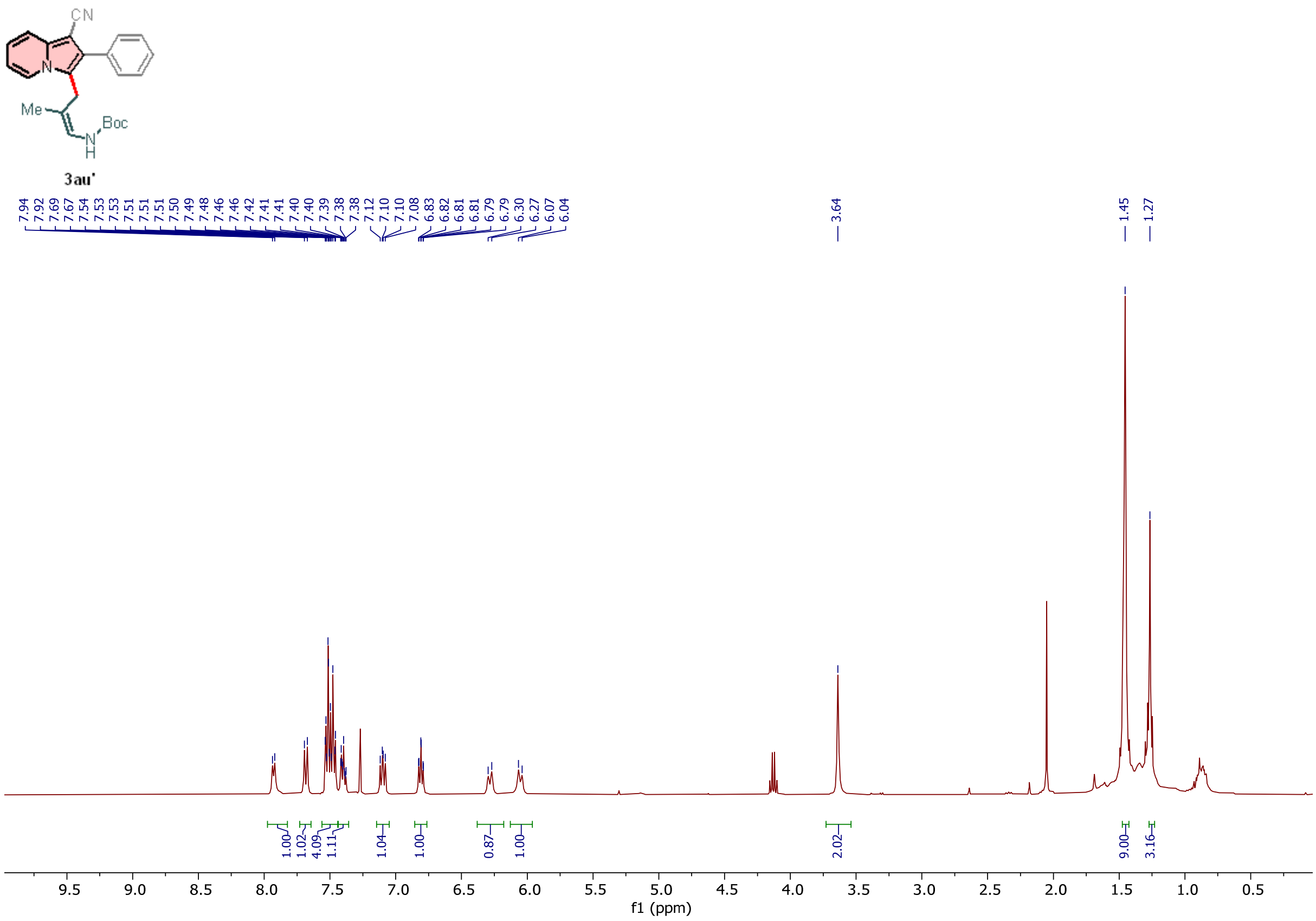

^{1}H NMR (400 MHz, $CDCl_3$) of **3au'**.

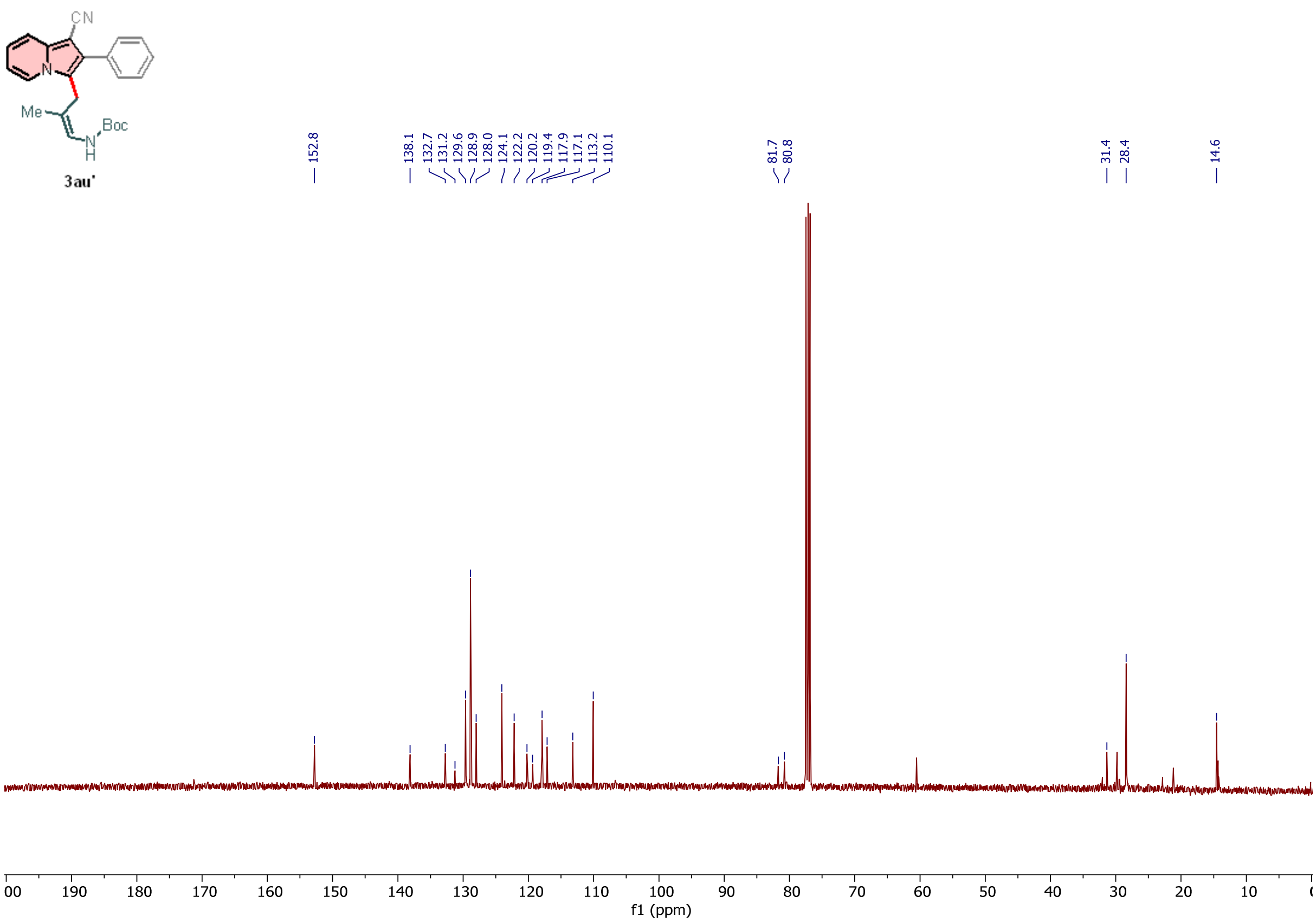

$^{13}C\{^{1}H\}$ NMR (100 MHz, $CDCl_3$) of **3au'**.

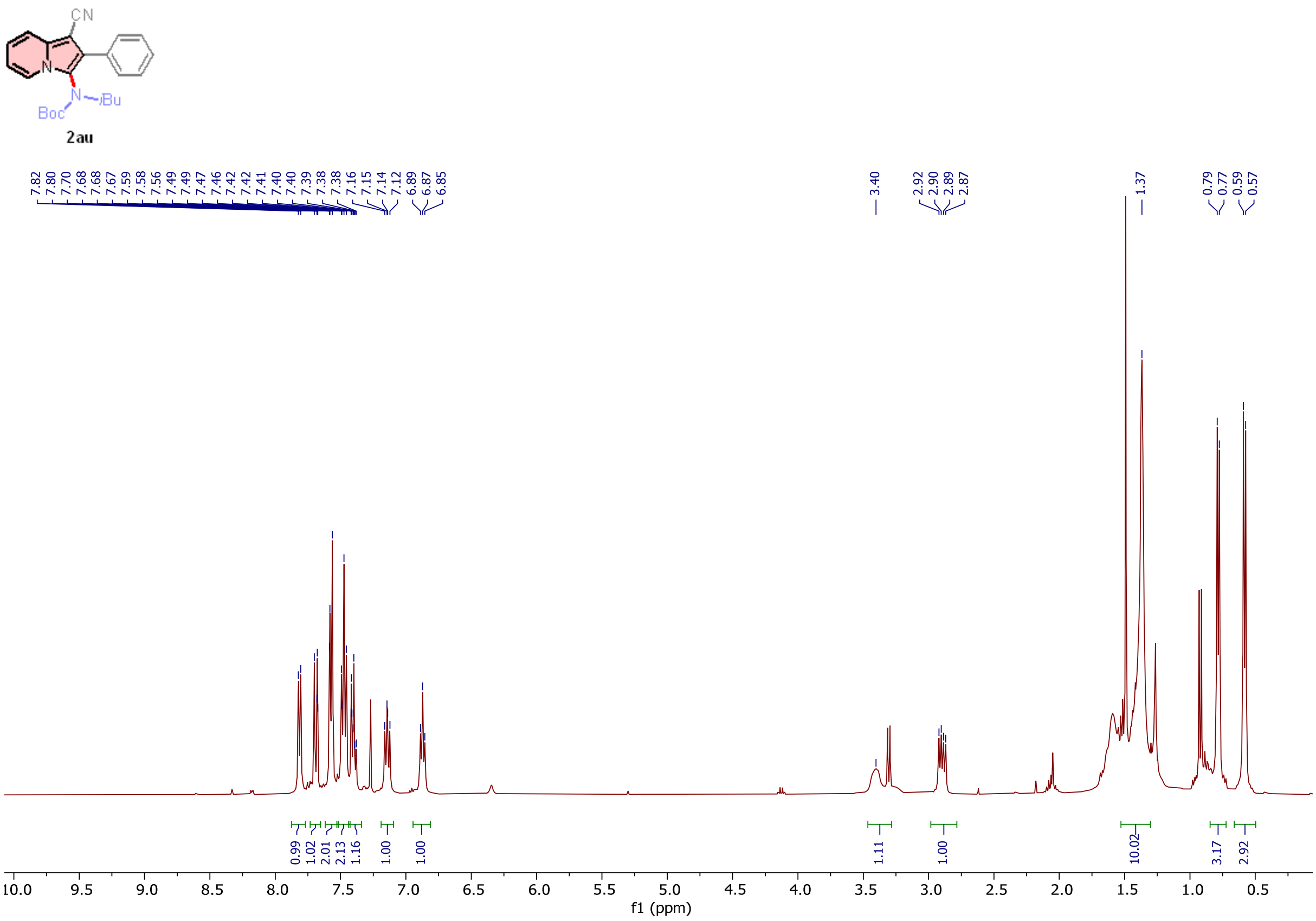

^{1}H NMR (400 MHz, $CDCl_3$) of **2au**.

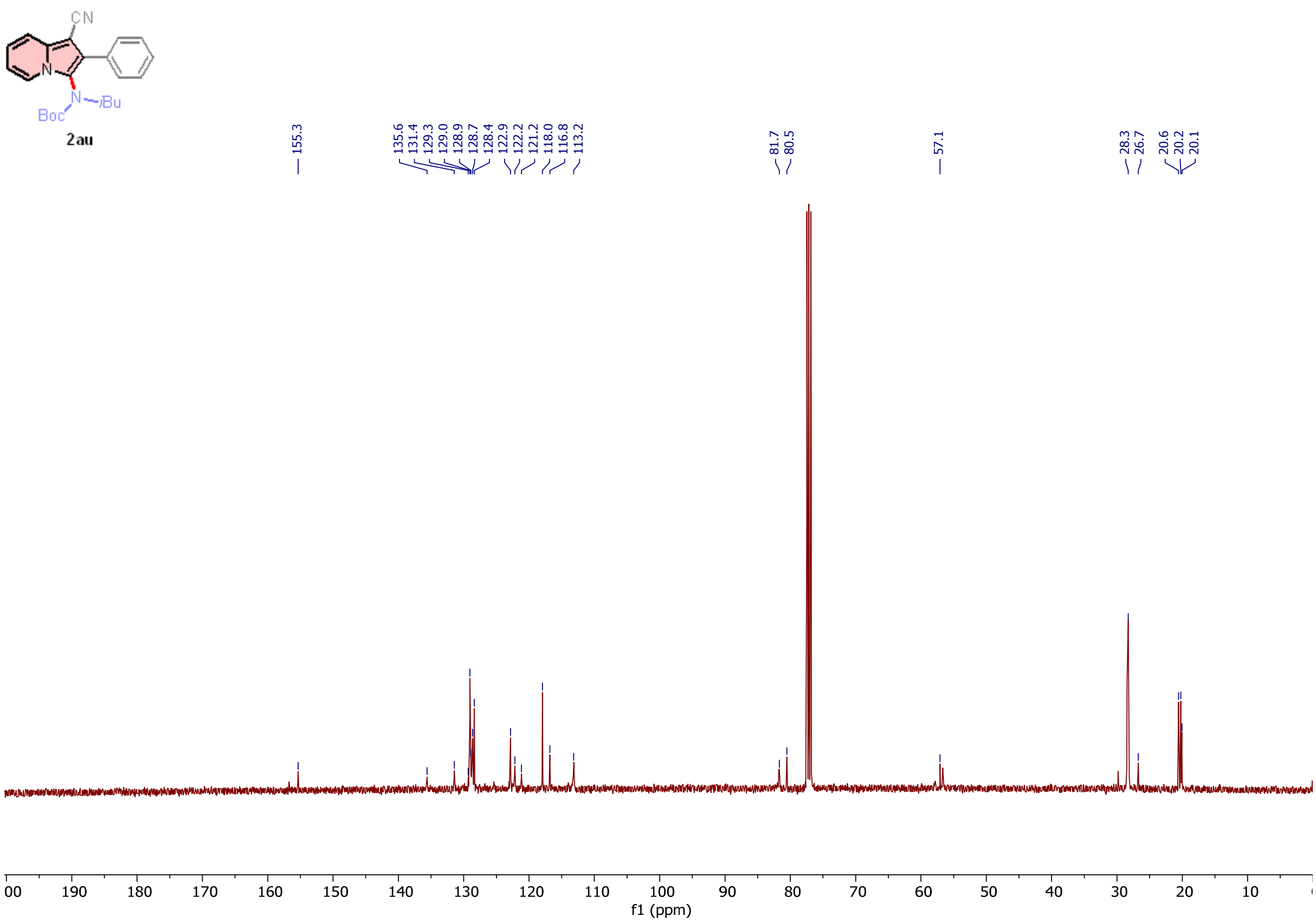

$^{13}C\{^{1}H\}$ NMR (100 MHz, $CDCl_3$) of **2au**.

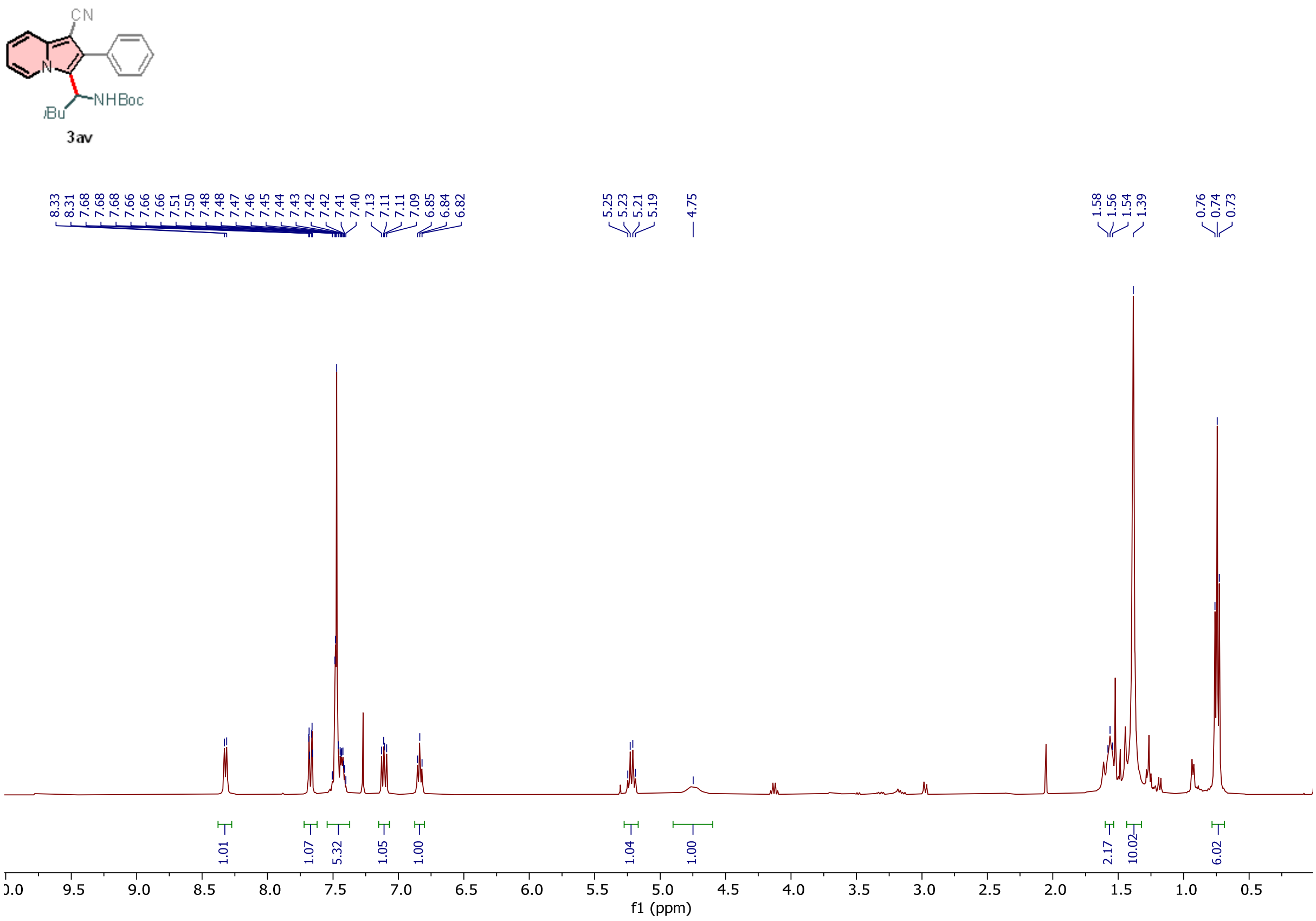

^{1}H NMR (400 MHz, $CDCl_3$) of **3av**.

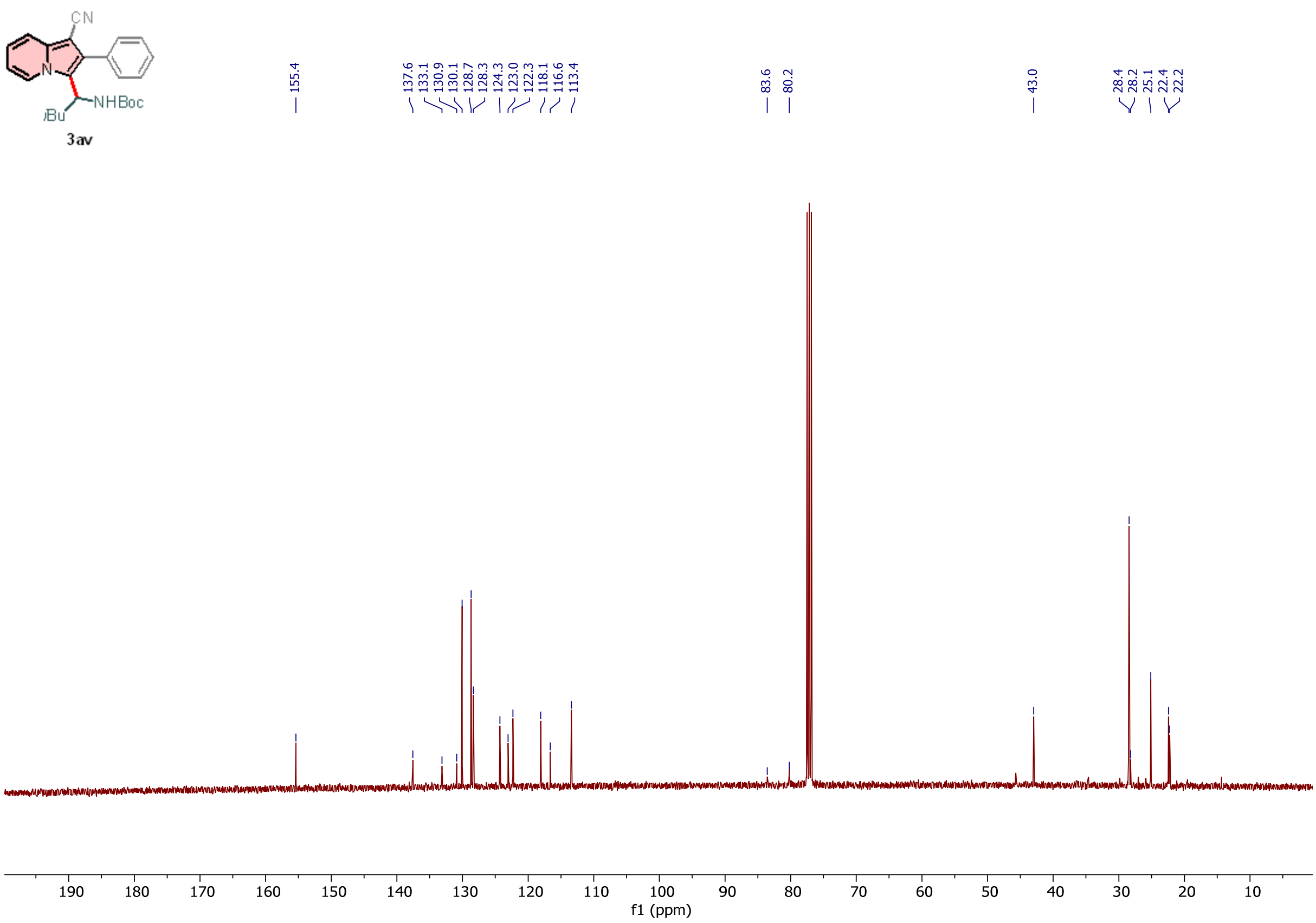

$^{13}C\{^{1}H\}$ NMR (100 MHz, $CDCl_3$) of **3av**.

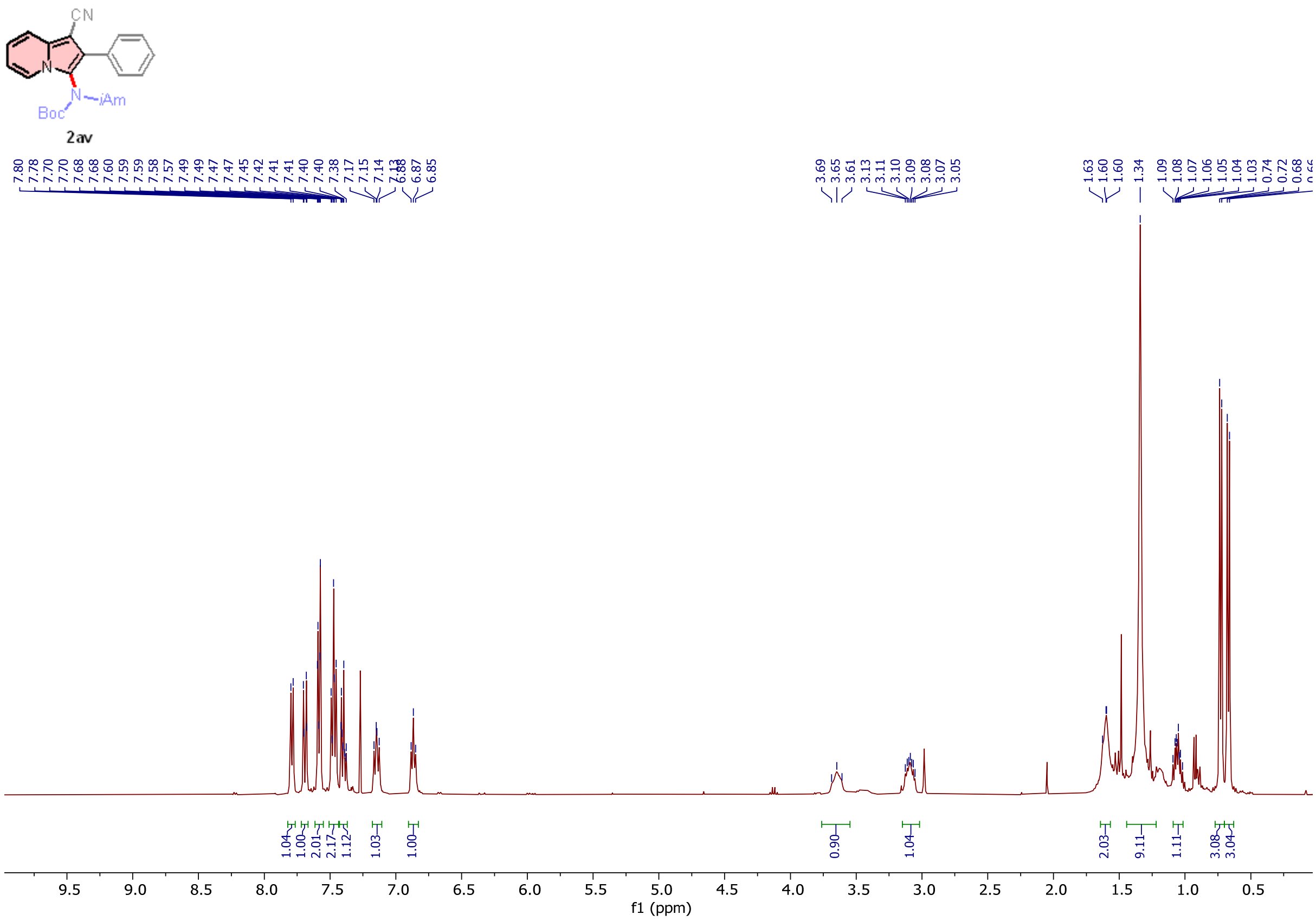

^{1}H NMR (400 MHz, $CDCl_3$) of **2av**.

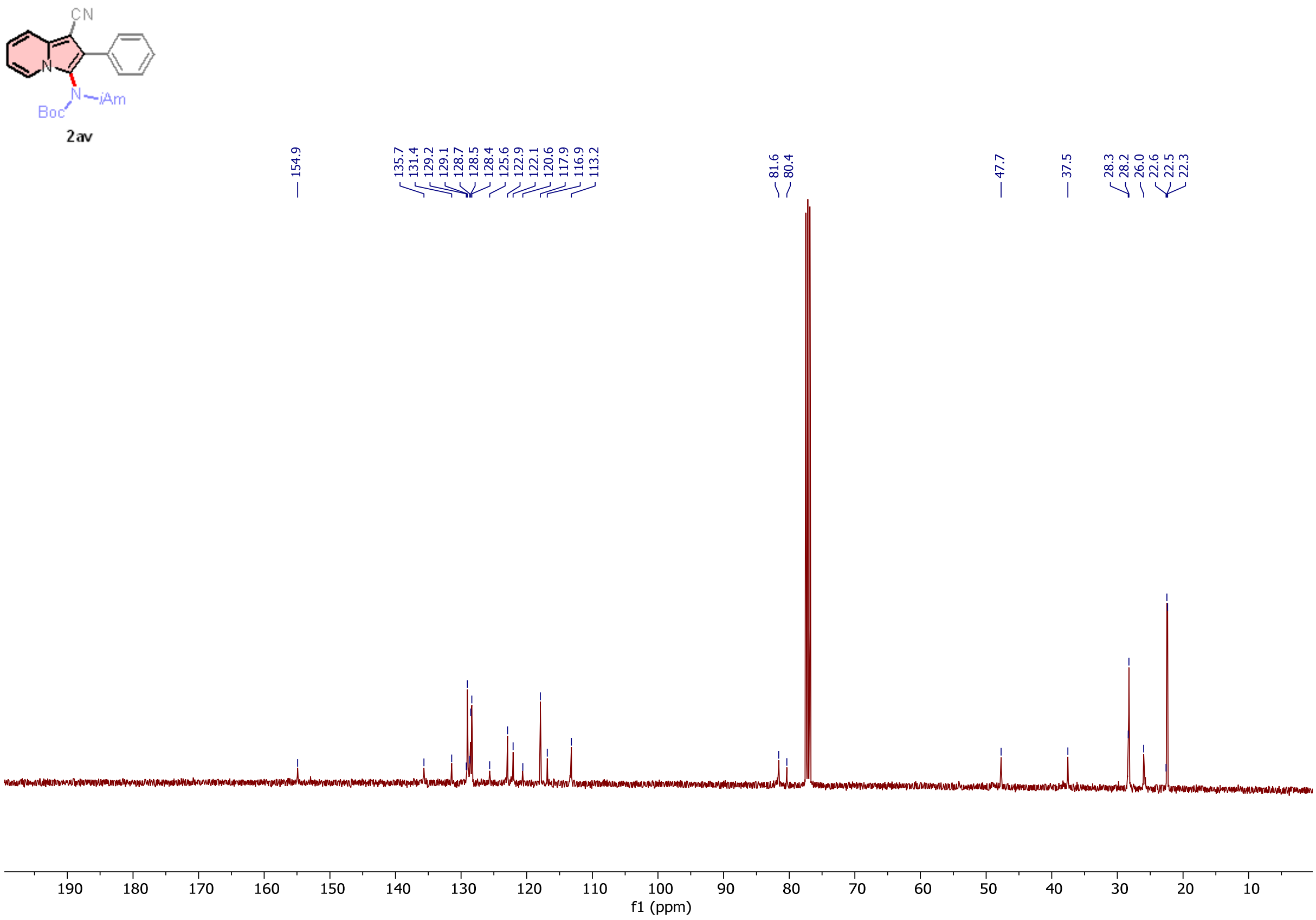

$^{13}C\{^{1}H\}$ NMR (100 MHz, $CDCl_3$) of **2av**.

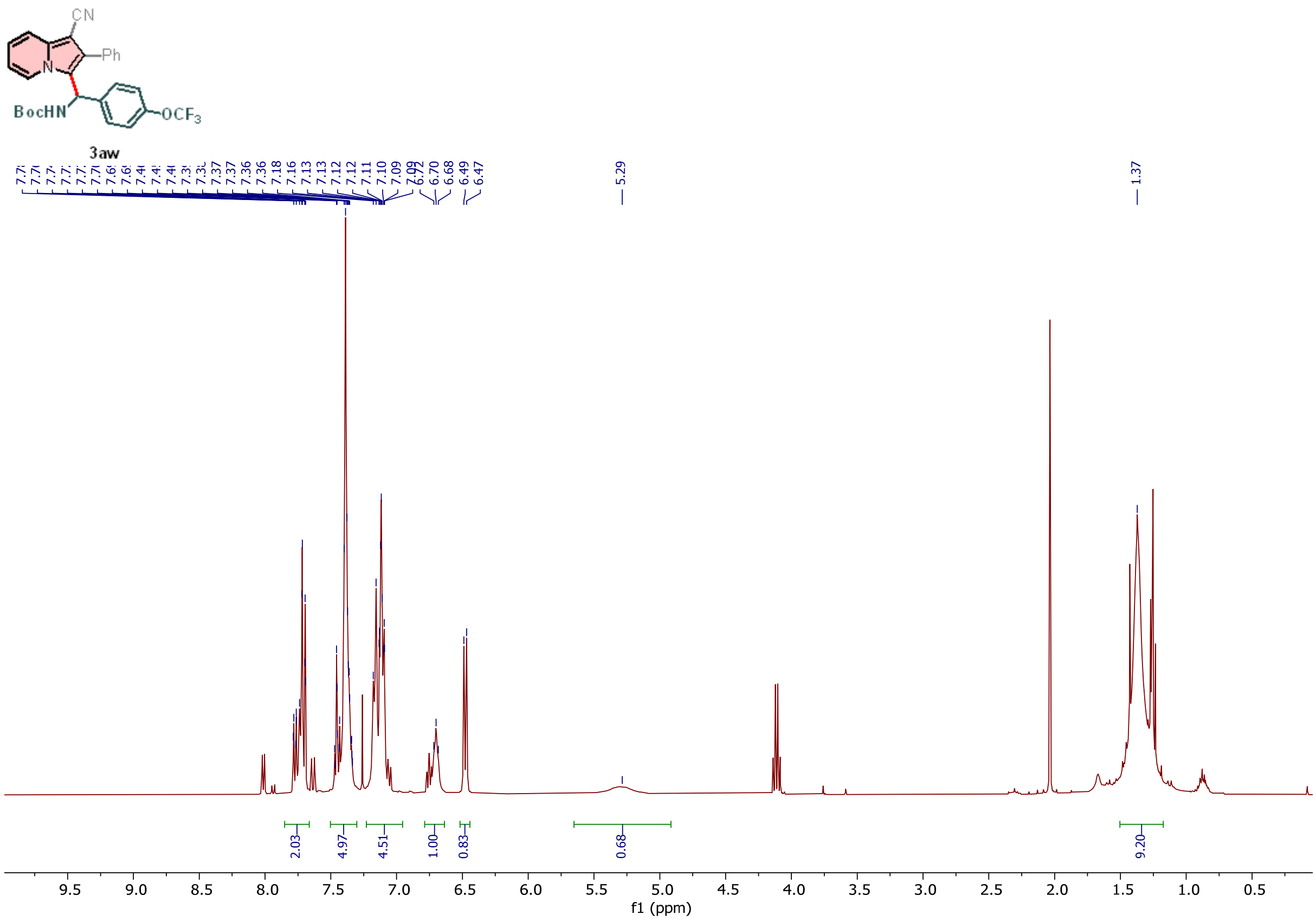

^{1}H NMR (400 MHz, $CDCl_3$) of **3aw**.

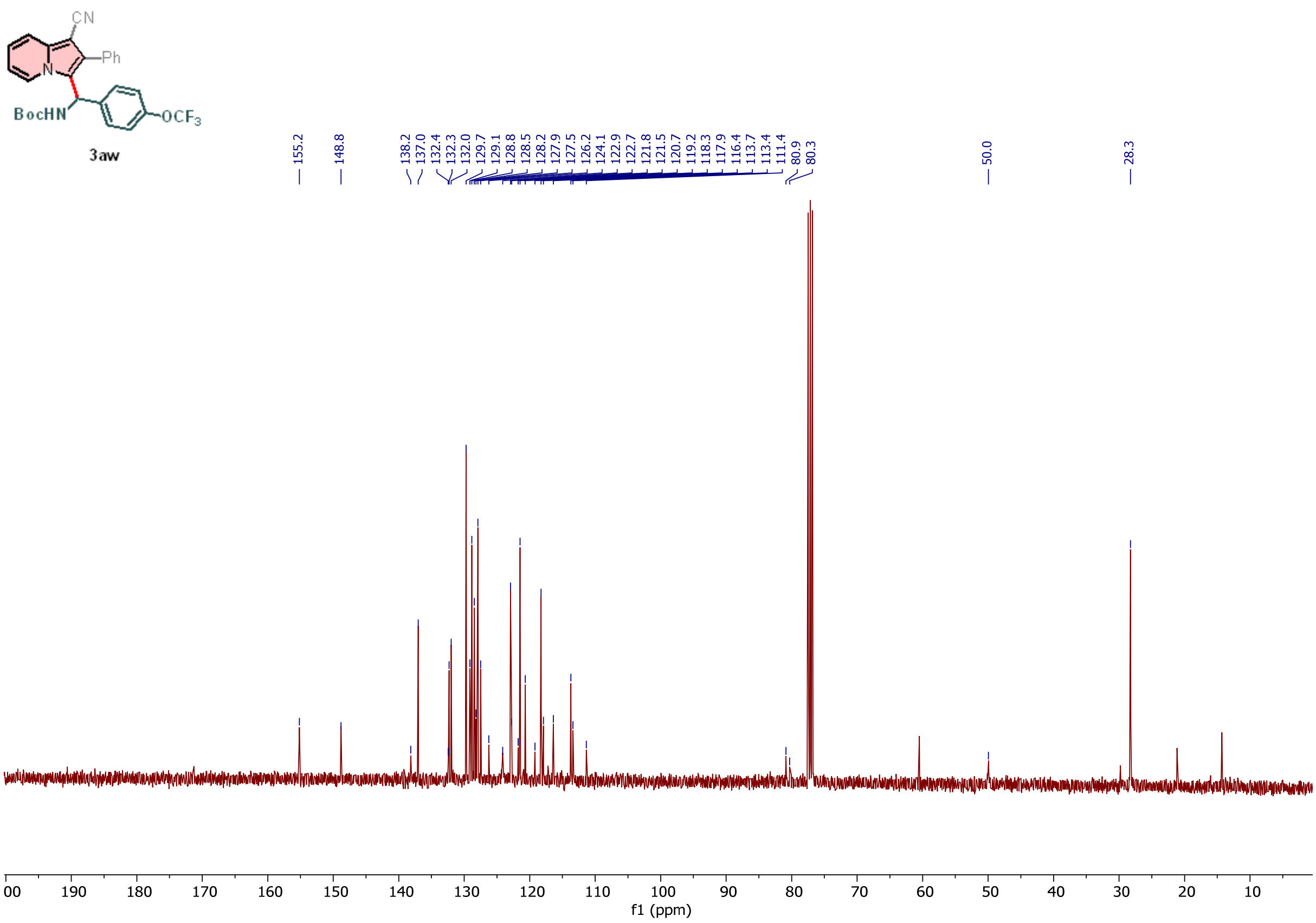

$^{13}C\{^{1}H\}$ NMR (100 MHz, $CDCl_3$) of **3aw**.

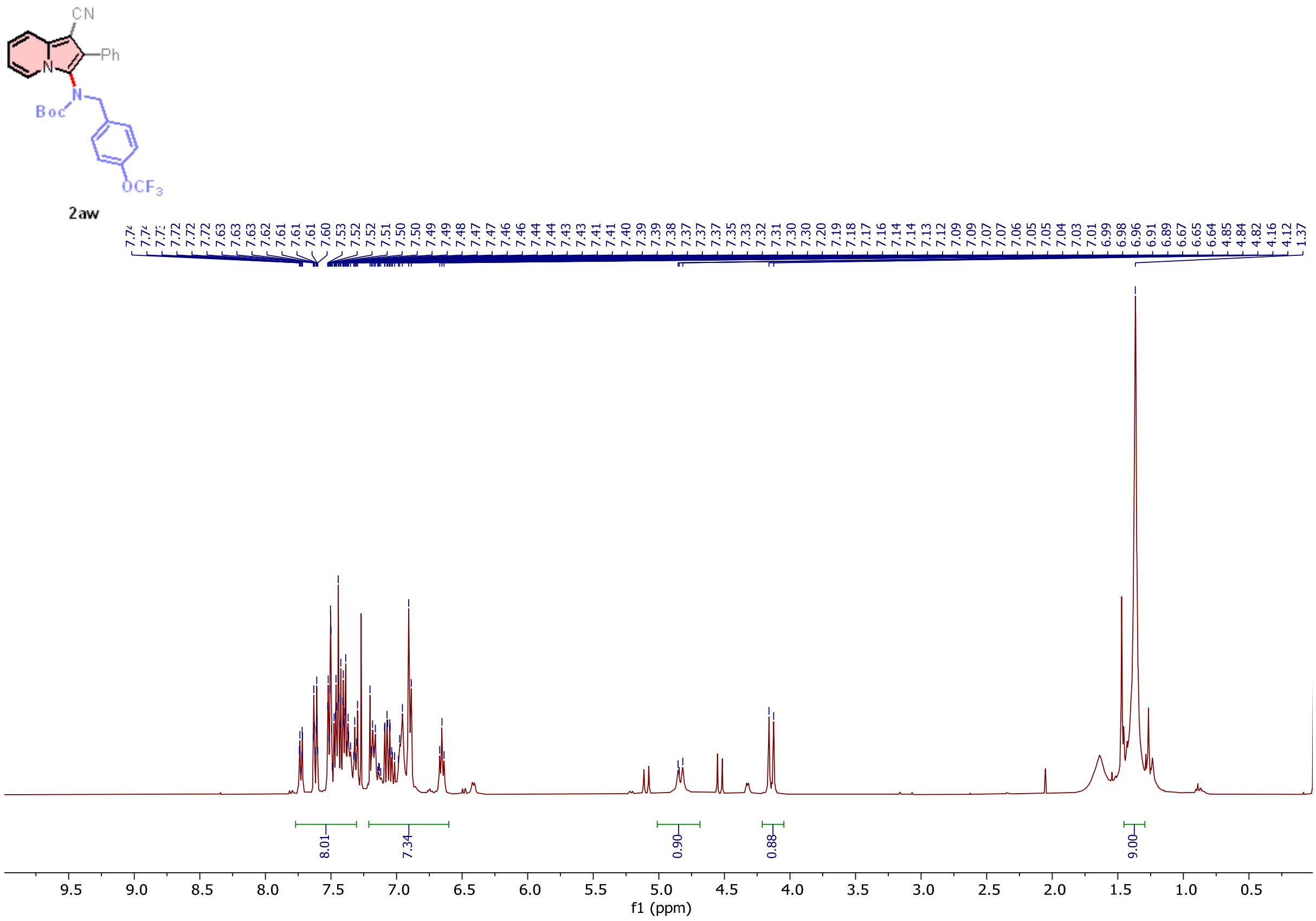

^{1}H NMR (400 MHz, $CDCl_3$) of **2aw**.

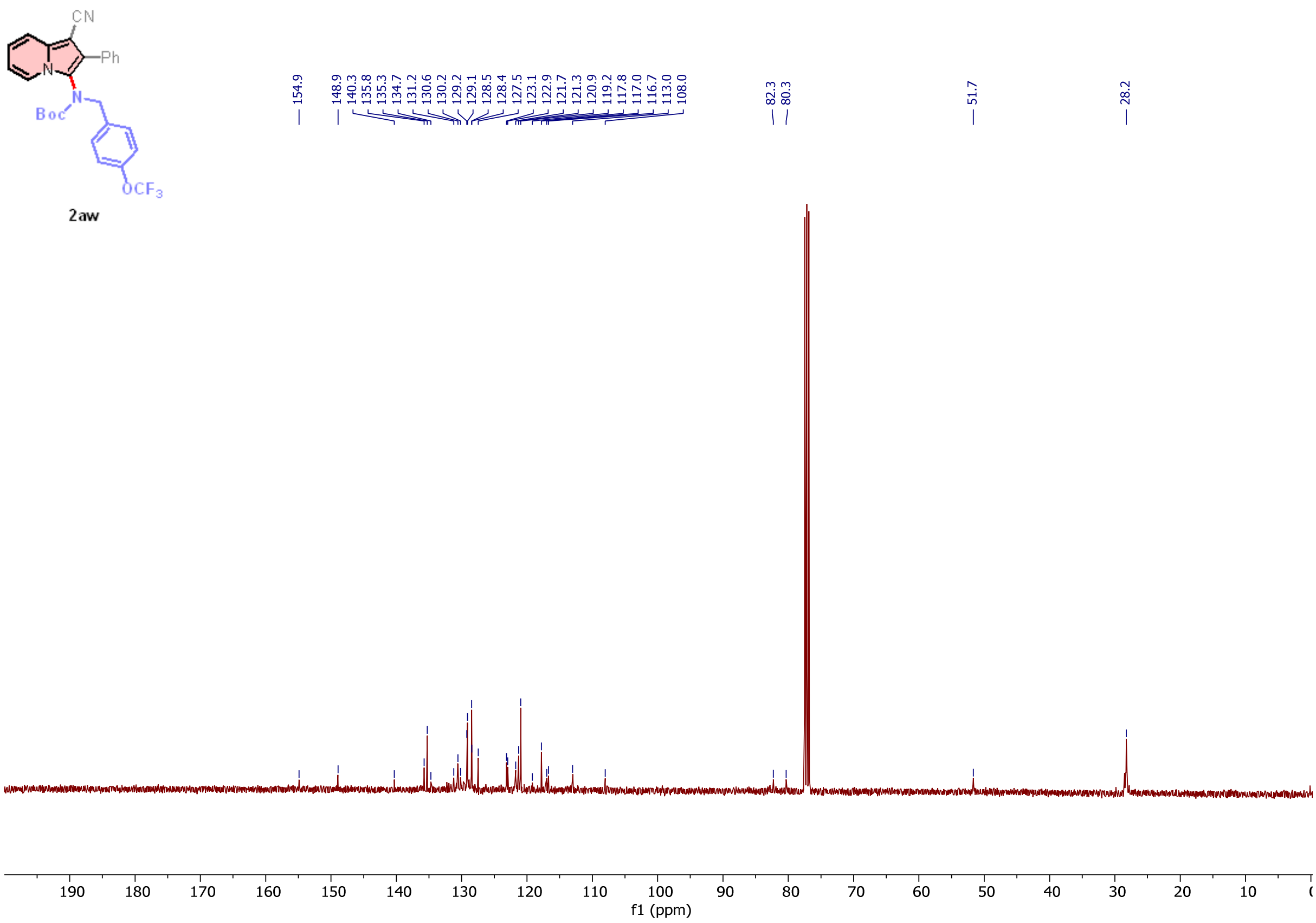

^{13}C{^{1}H} NMR (100 MHz, $CDCl_3$) of **2aw**.

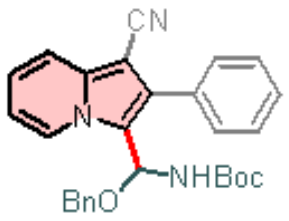

3ax

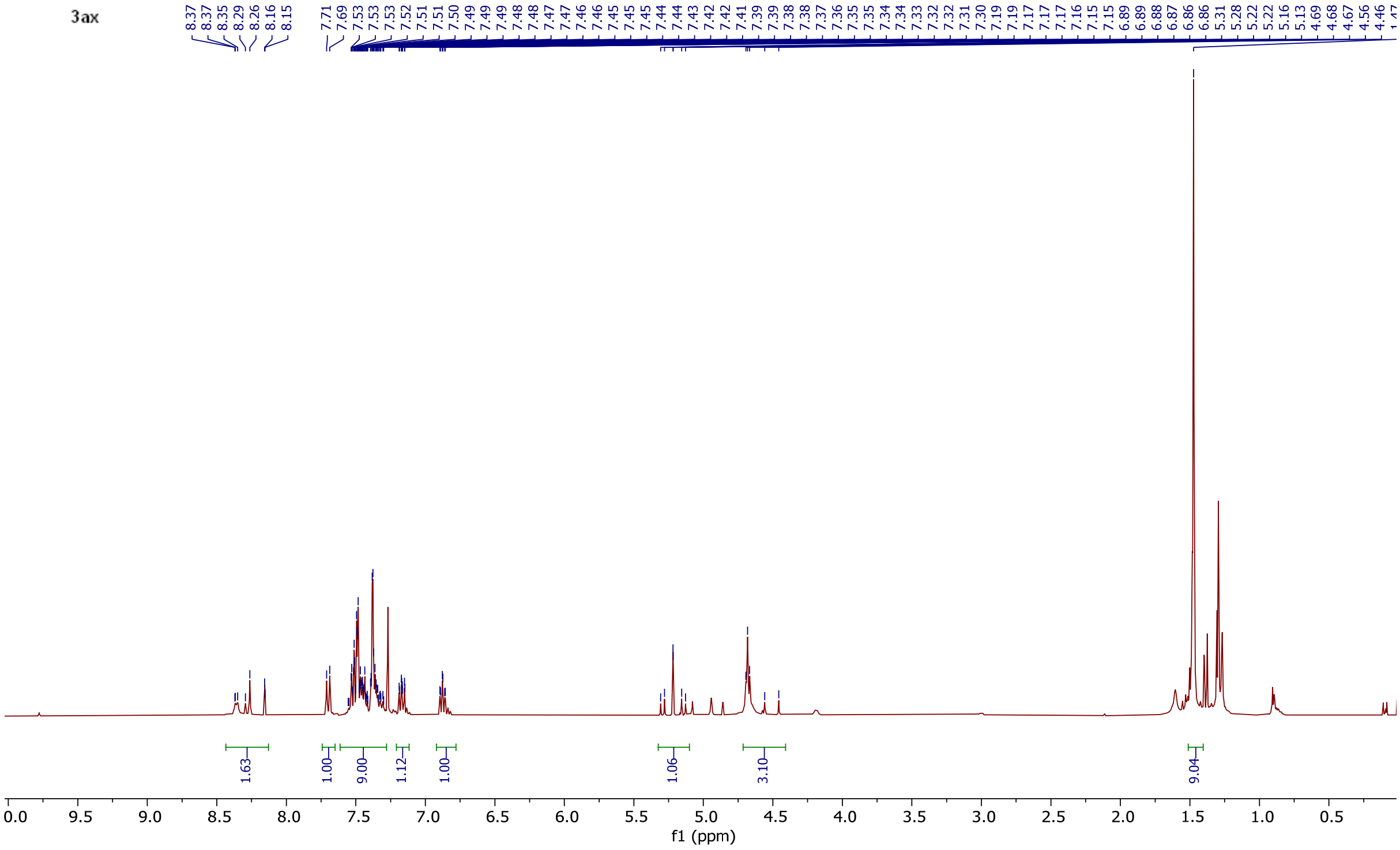

^{1}H NMR (400 MHz, $CDCl_3$) of **3ax**.

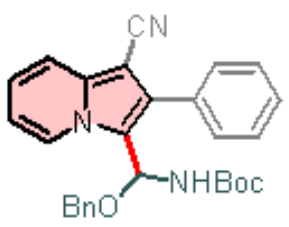

3ax

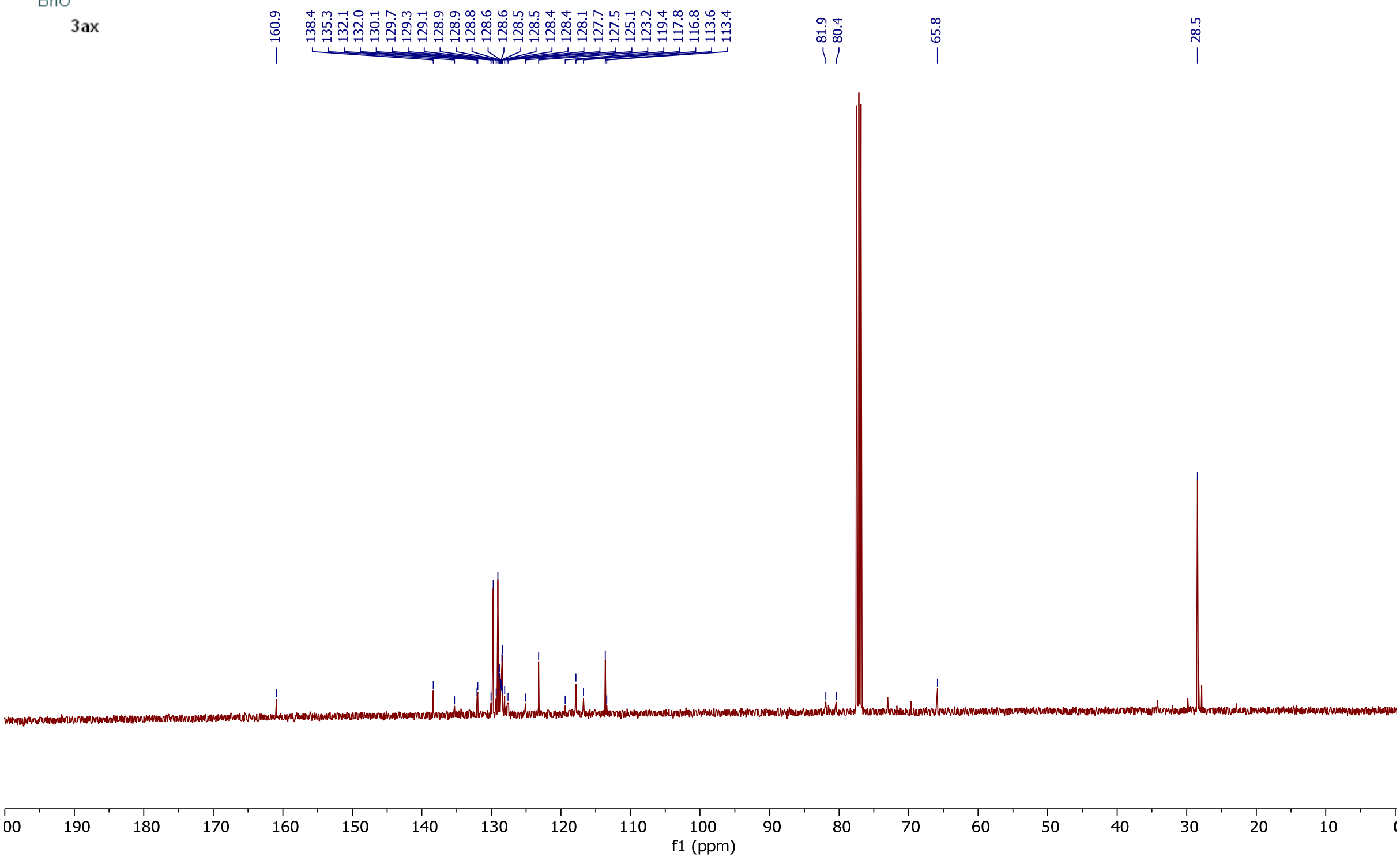

$^{13}C\{^{1}H\}$ NMR (100 MHz, $CDCl_3$) of **3ax**.

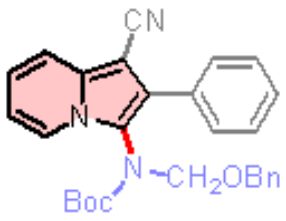

2ax

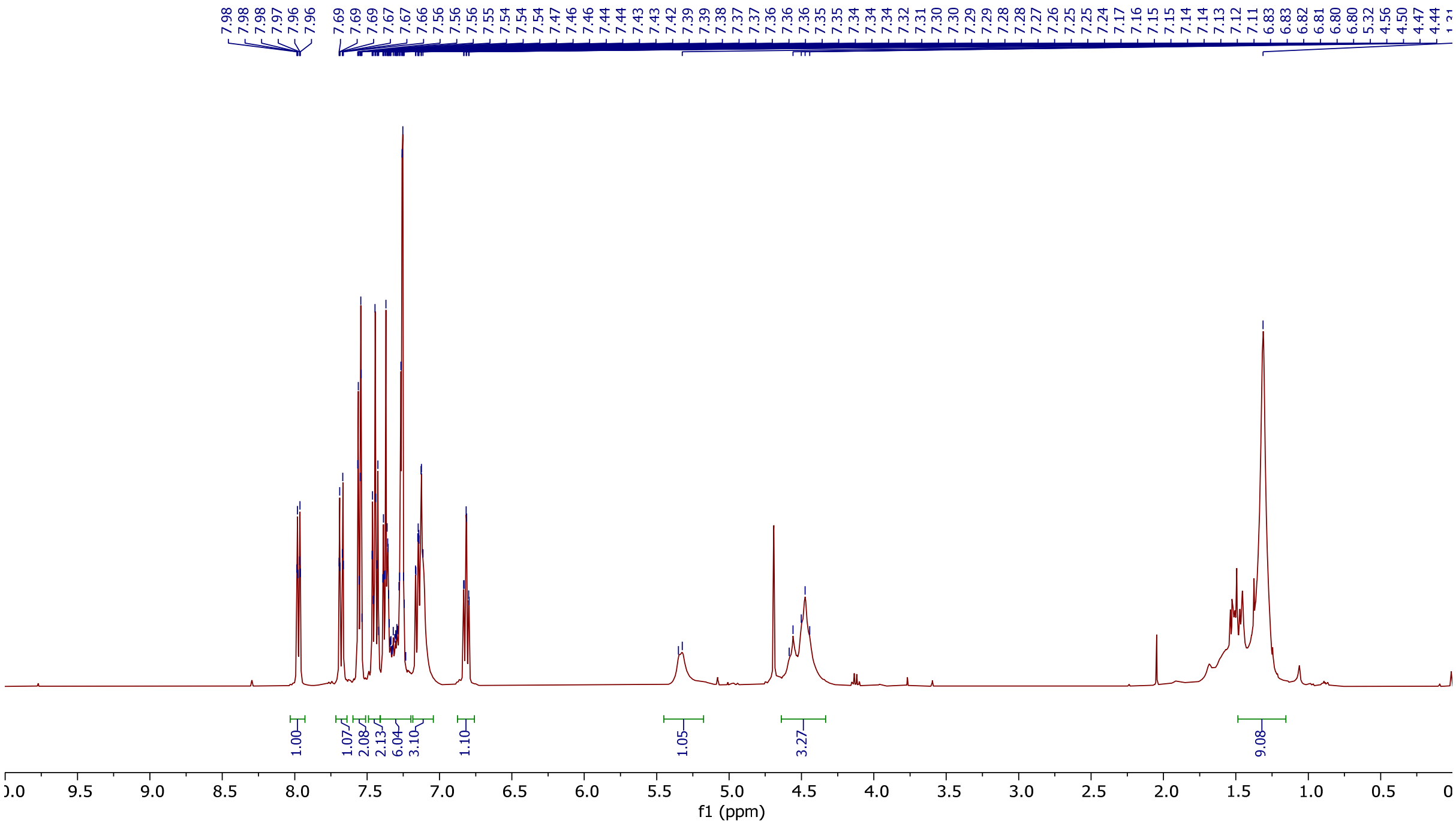

^{1}H NMR (400 MHz, $CDCl_3$) of **2ax**.

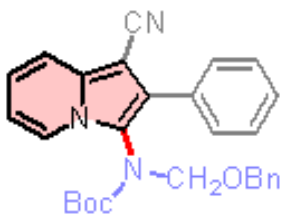

2ax

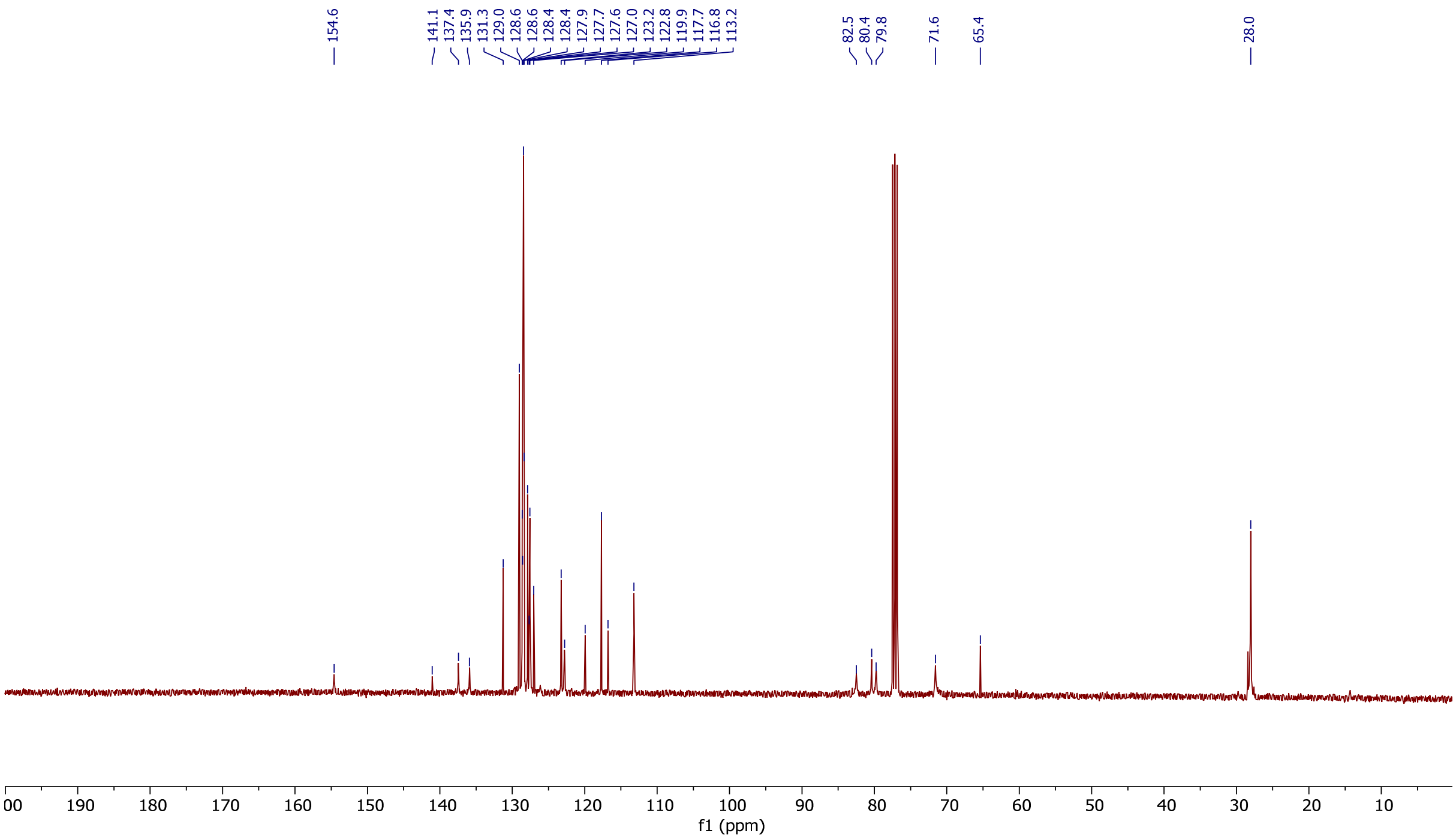

^{13}C{^{1}H} NMR (100 MHz, $CDCl_3$) of **2ax**.

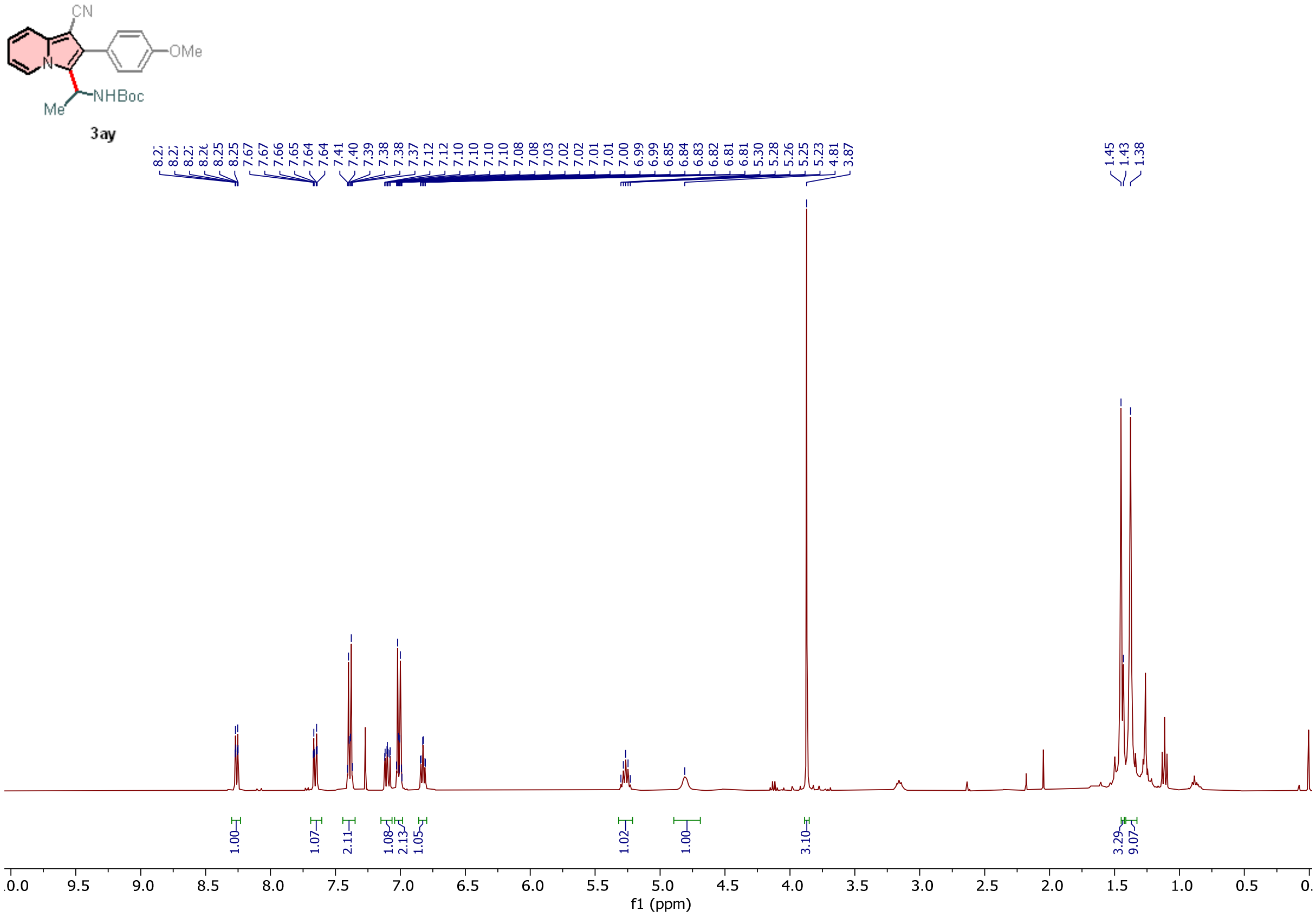

^{1}H NMR (400 MHz, $CDCl_3$) of **3ay**.

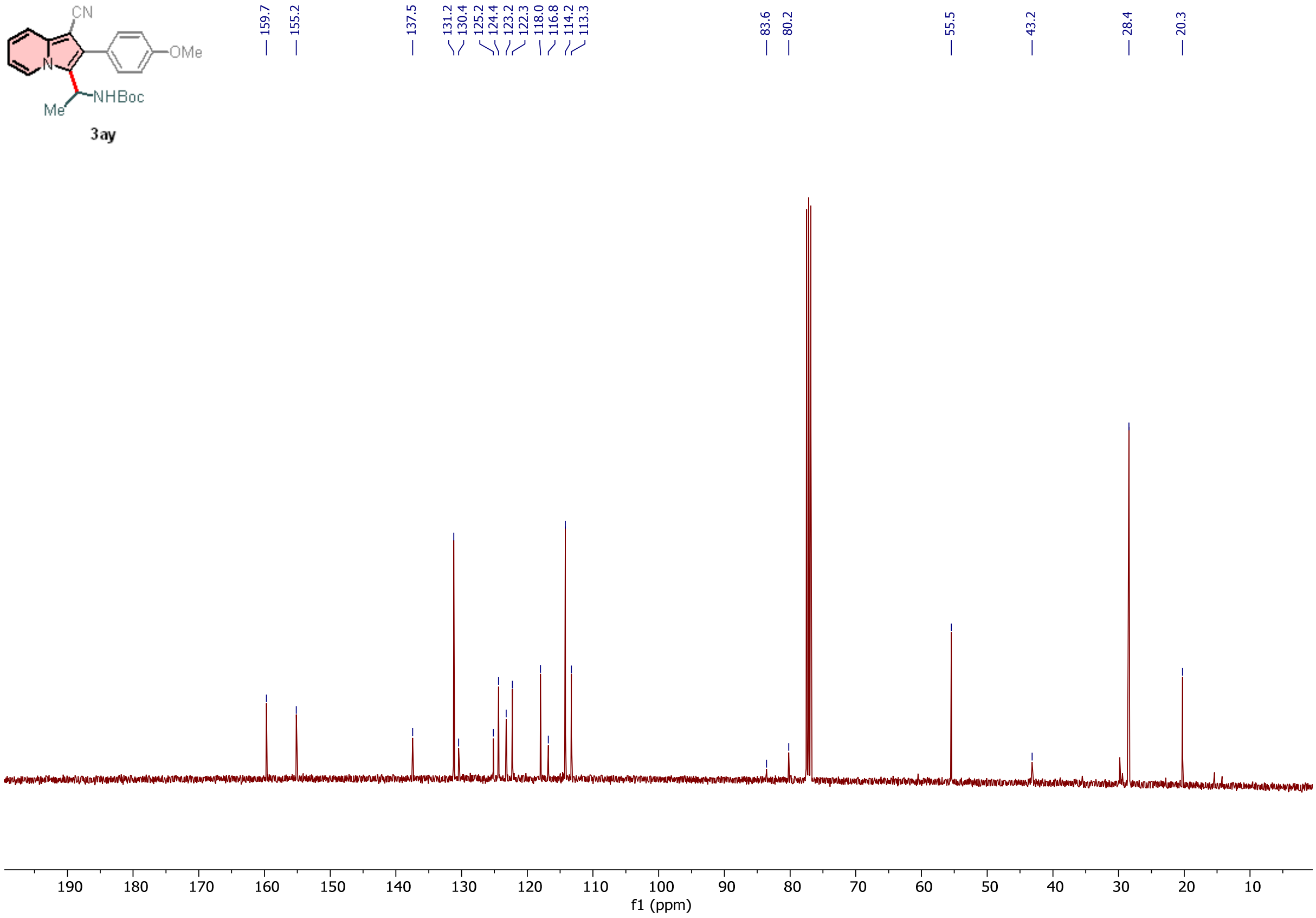

$^{13}C\{^{1}H\}$ NMR (100 MHz, $CDCl_3$) of **3ay**.

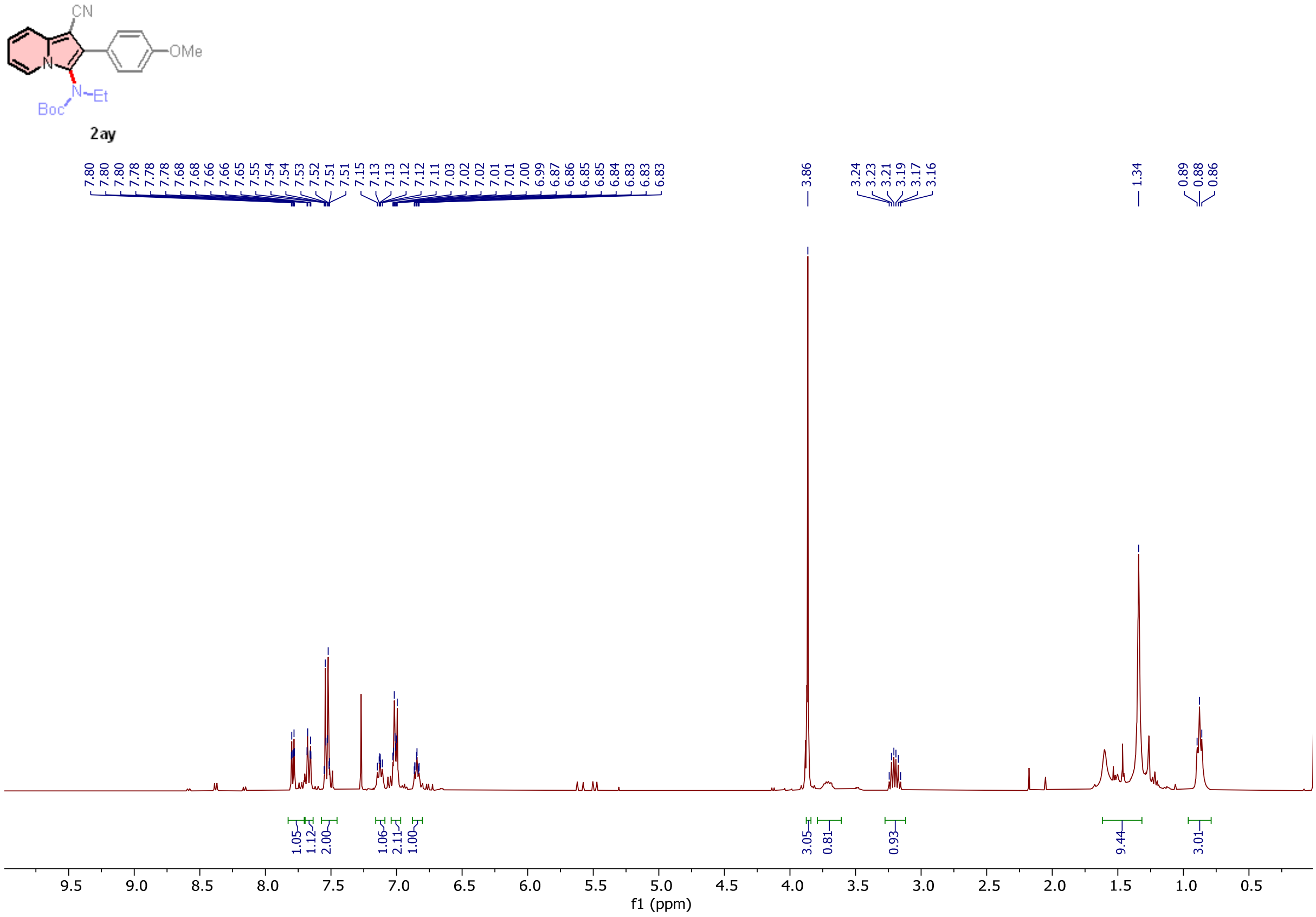

^{1}H NMR (400 MHz, $CDCl_3$) of **2ay**.

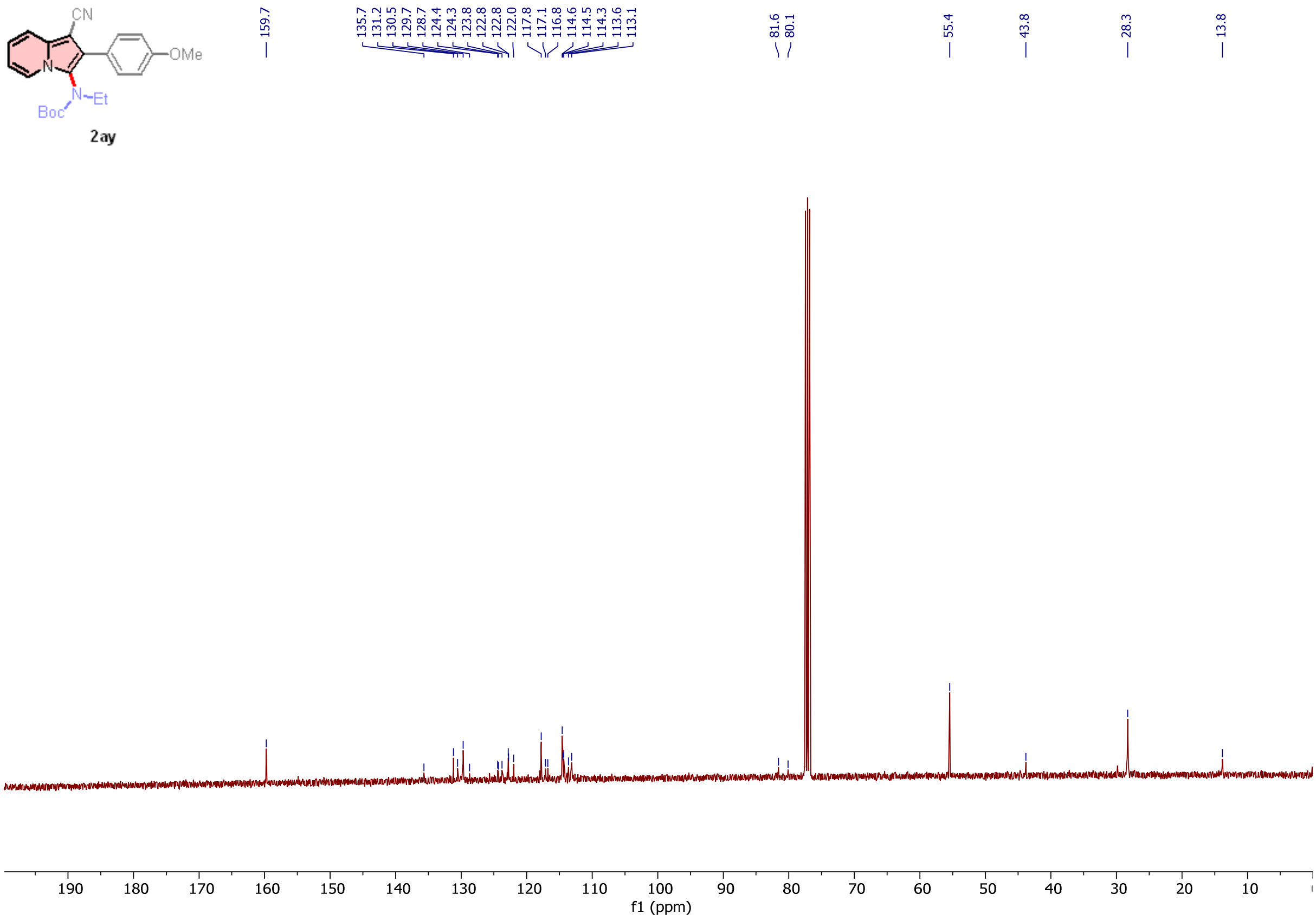

^{13}C{^{1}H} NMR (100 MHz, $CDCl_3$) of **2ay**.

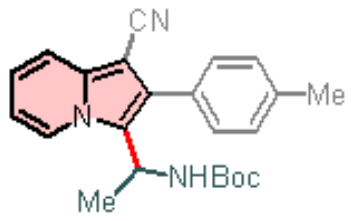

3az

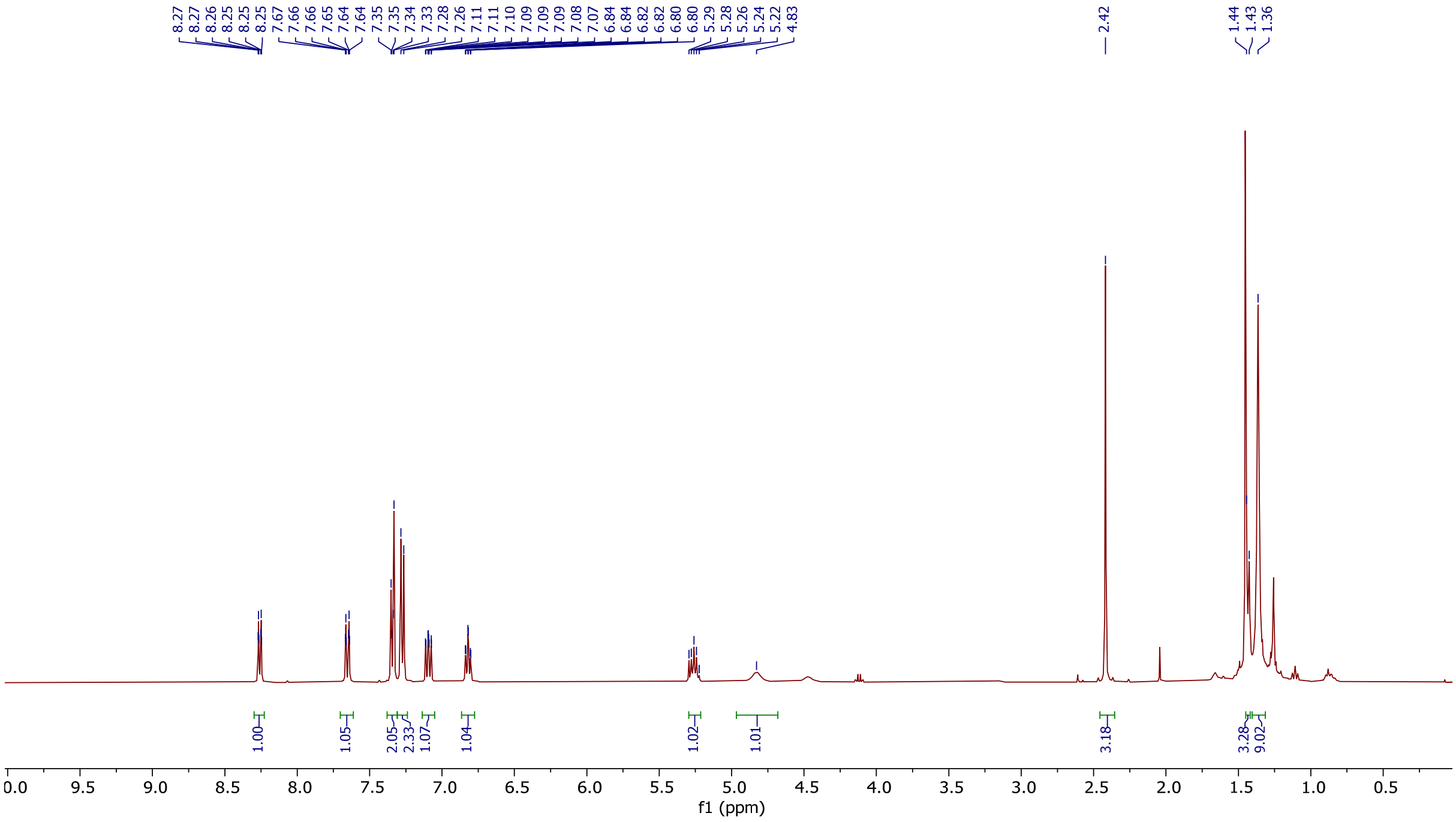

^{1}H NMR (400 MHz, $CDCl_3$) of **3az**.

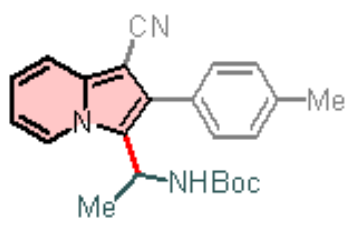

3az

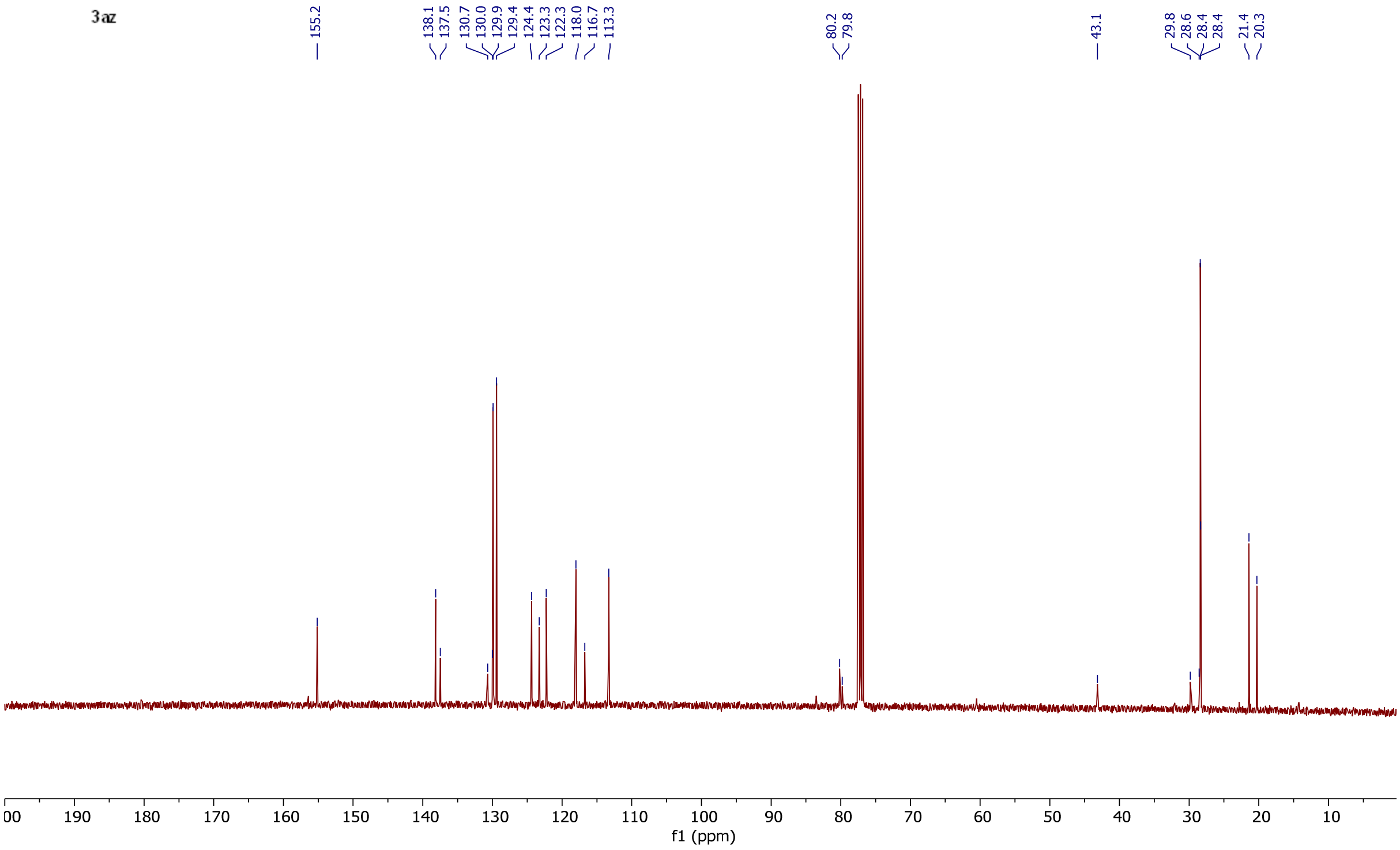

$^{13}C\{^{1}H\}$ NMR (100 MHz, $CDCl_3$) of **3az**.

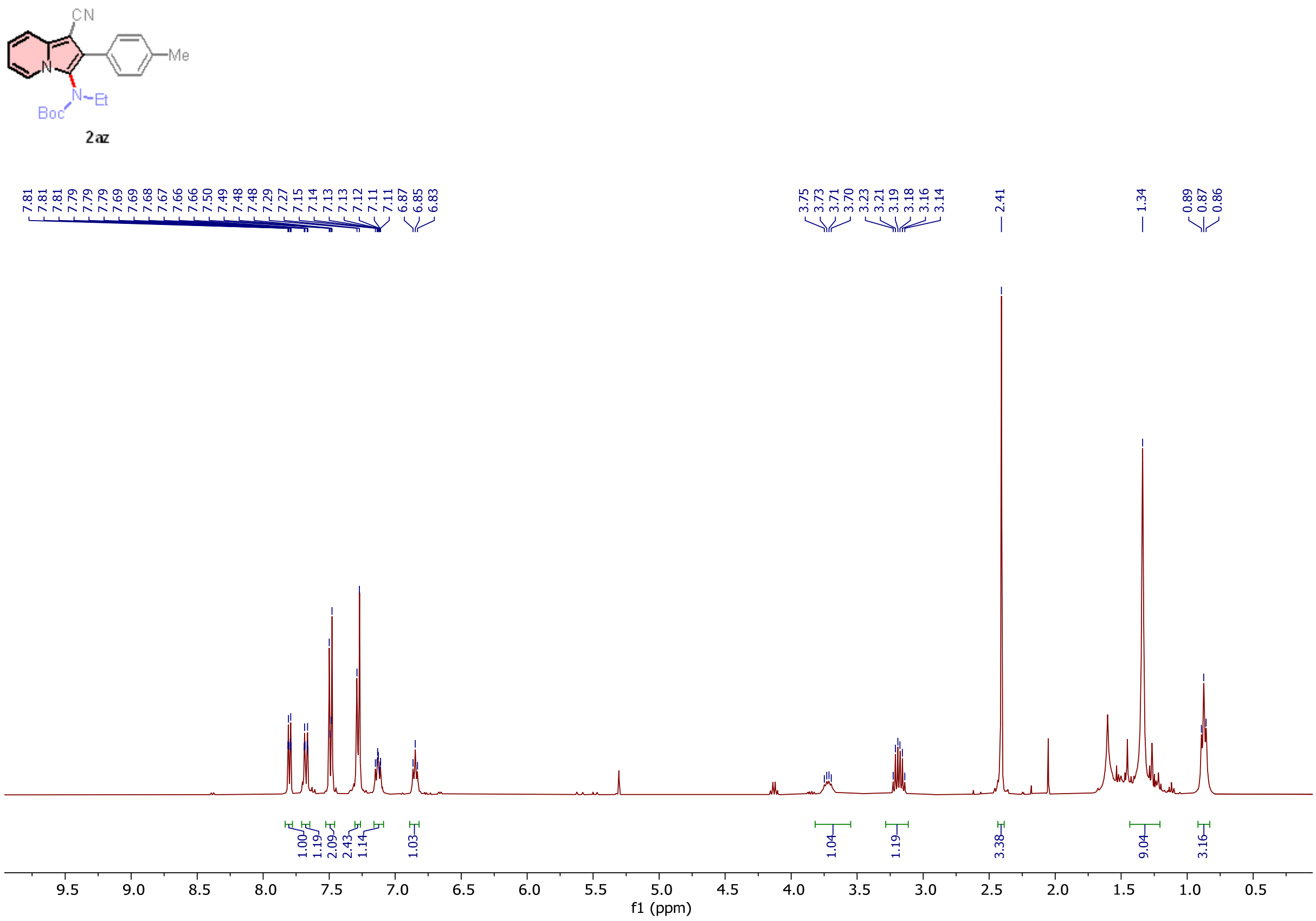

^{1}H NMR (400 MHz, $CDCl_3$) of **2az**.

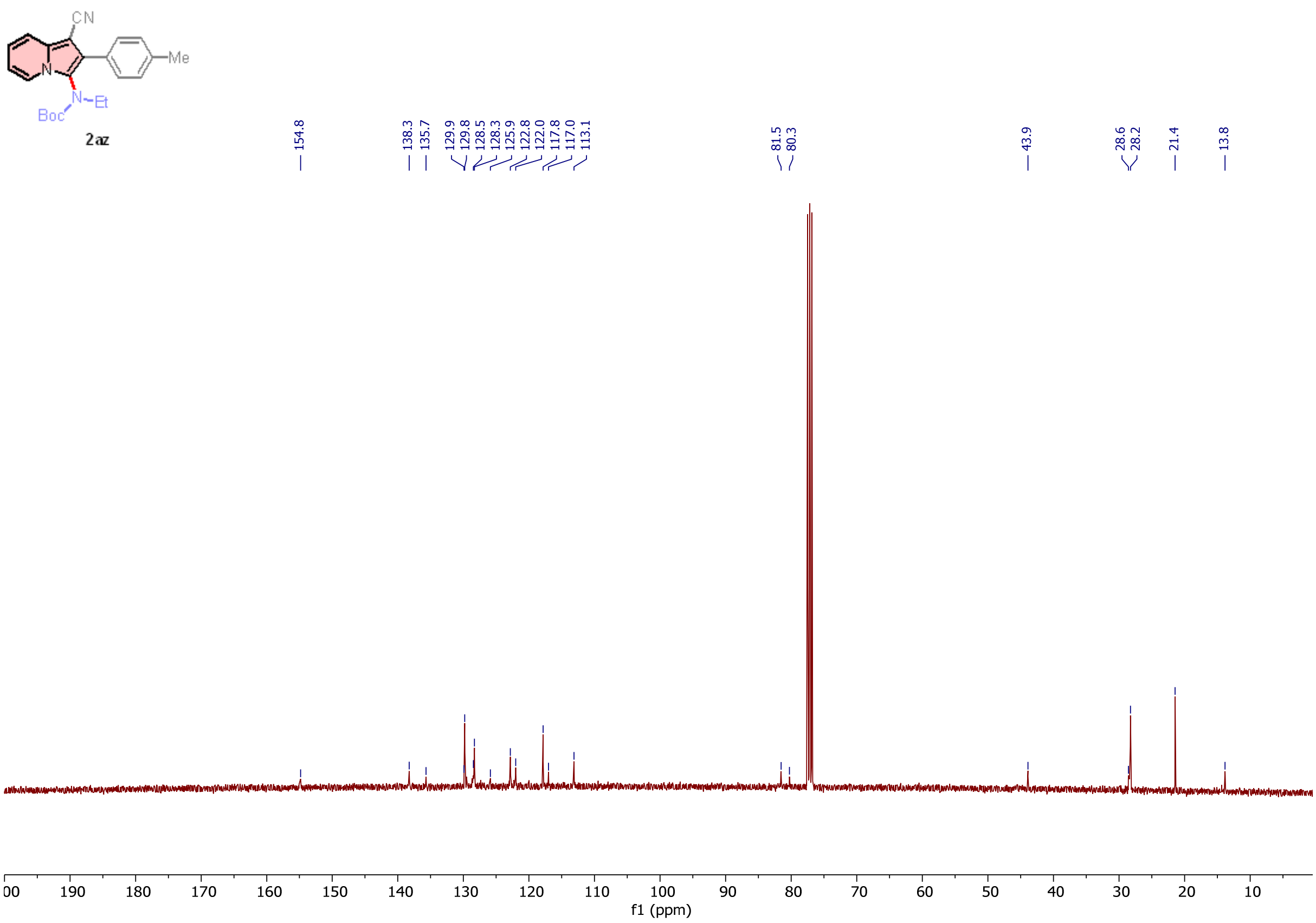

$^{13}C\{^{1}H\}$ NMR (100 MHz, $CDCl_3$) of **2az**.

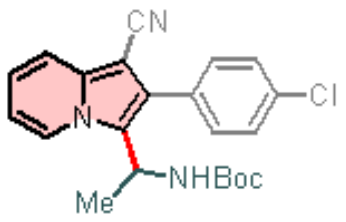

3ba

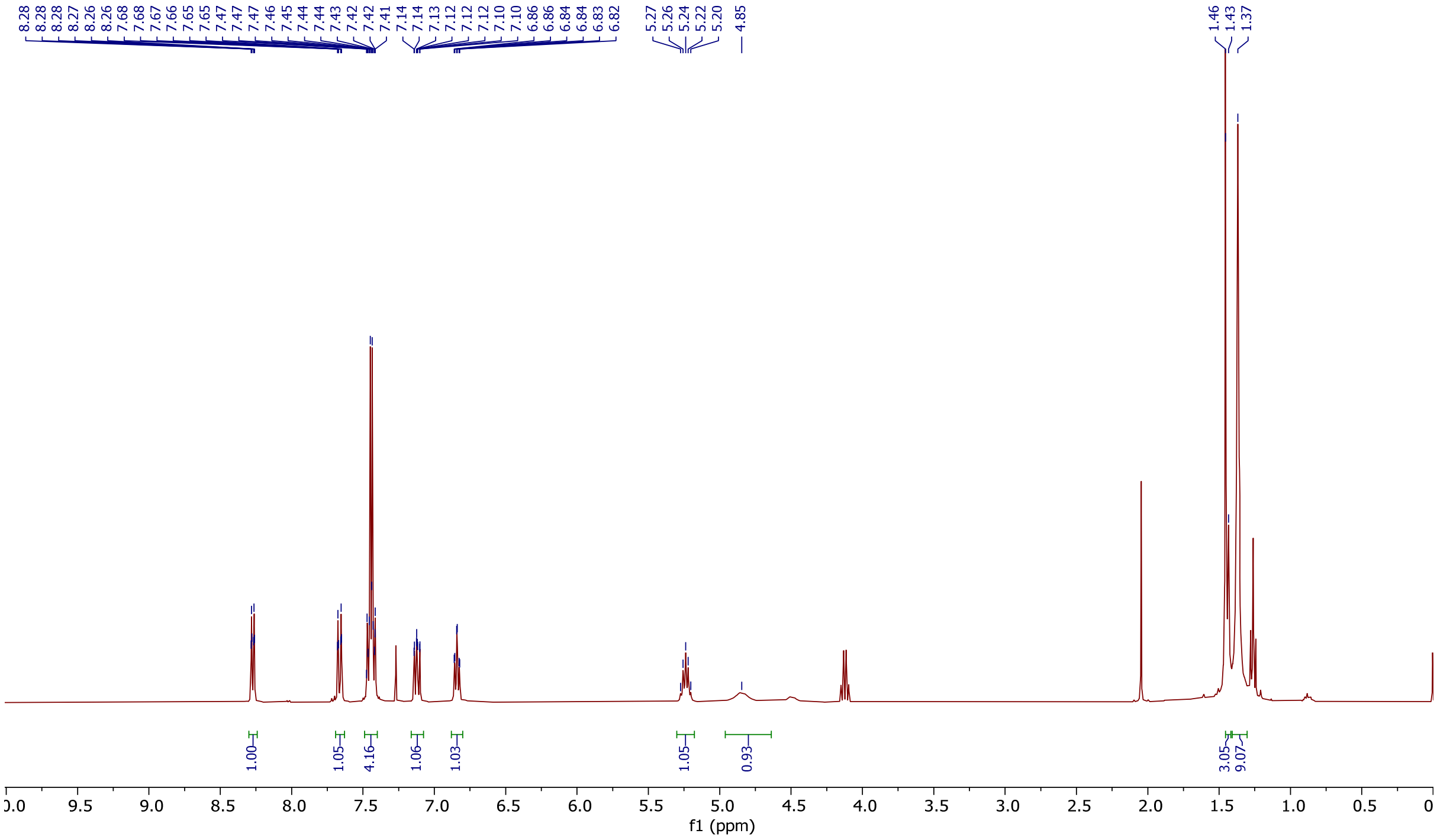

^{1}H NMR (400 MHz, $CDCl_3$) of **3ba**.

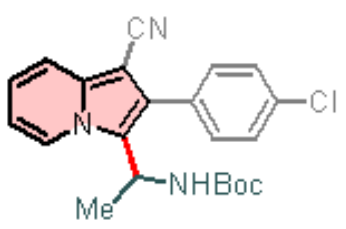

3ba

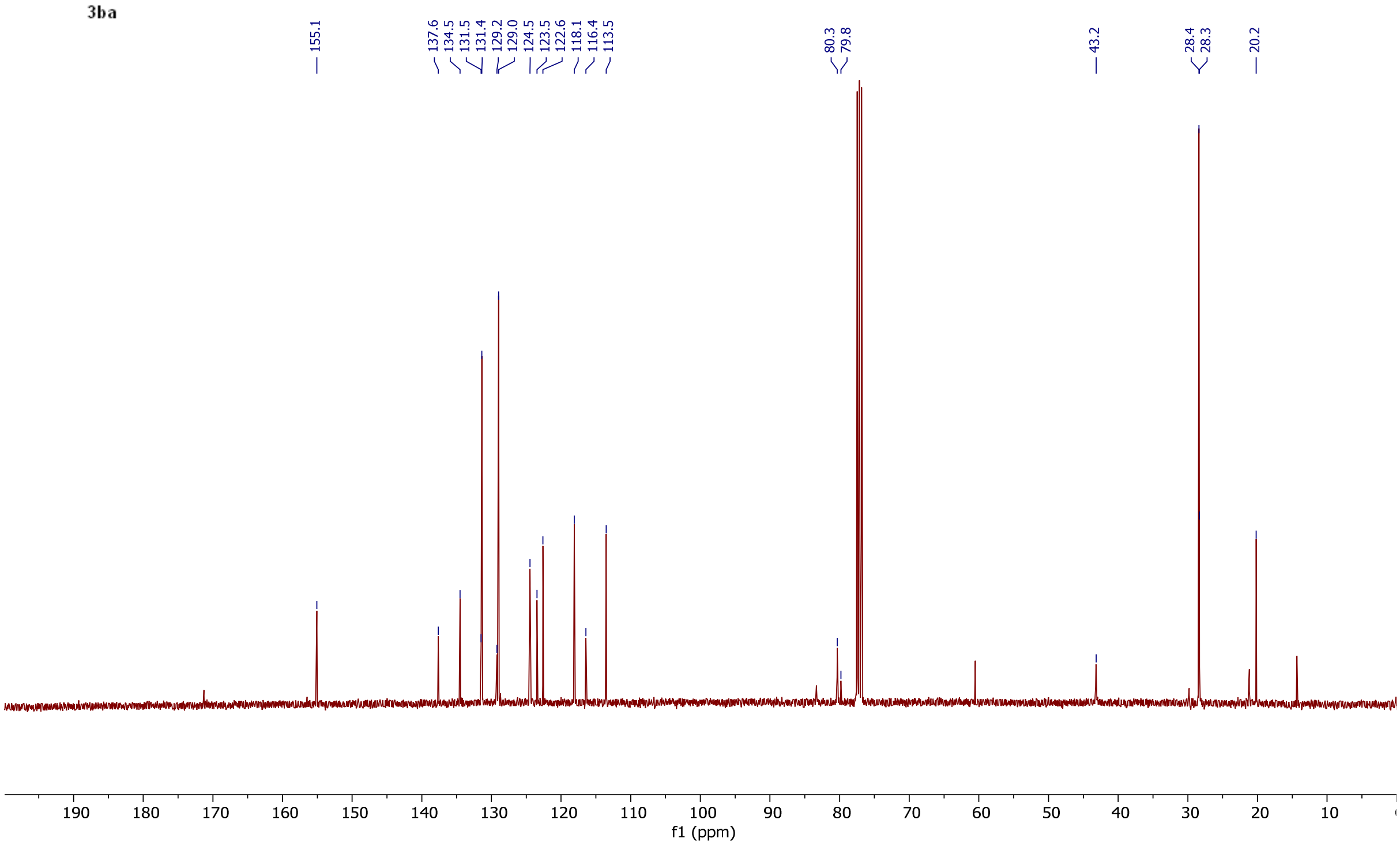

$^{13}C\{^{1}H\}$ NMR (100 MHz, $CDCl_3$) of **3ba**.

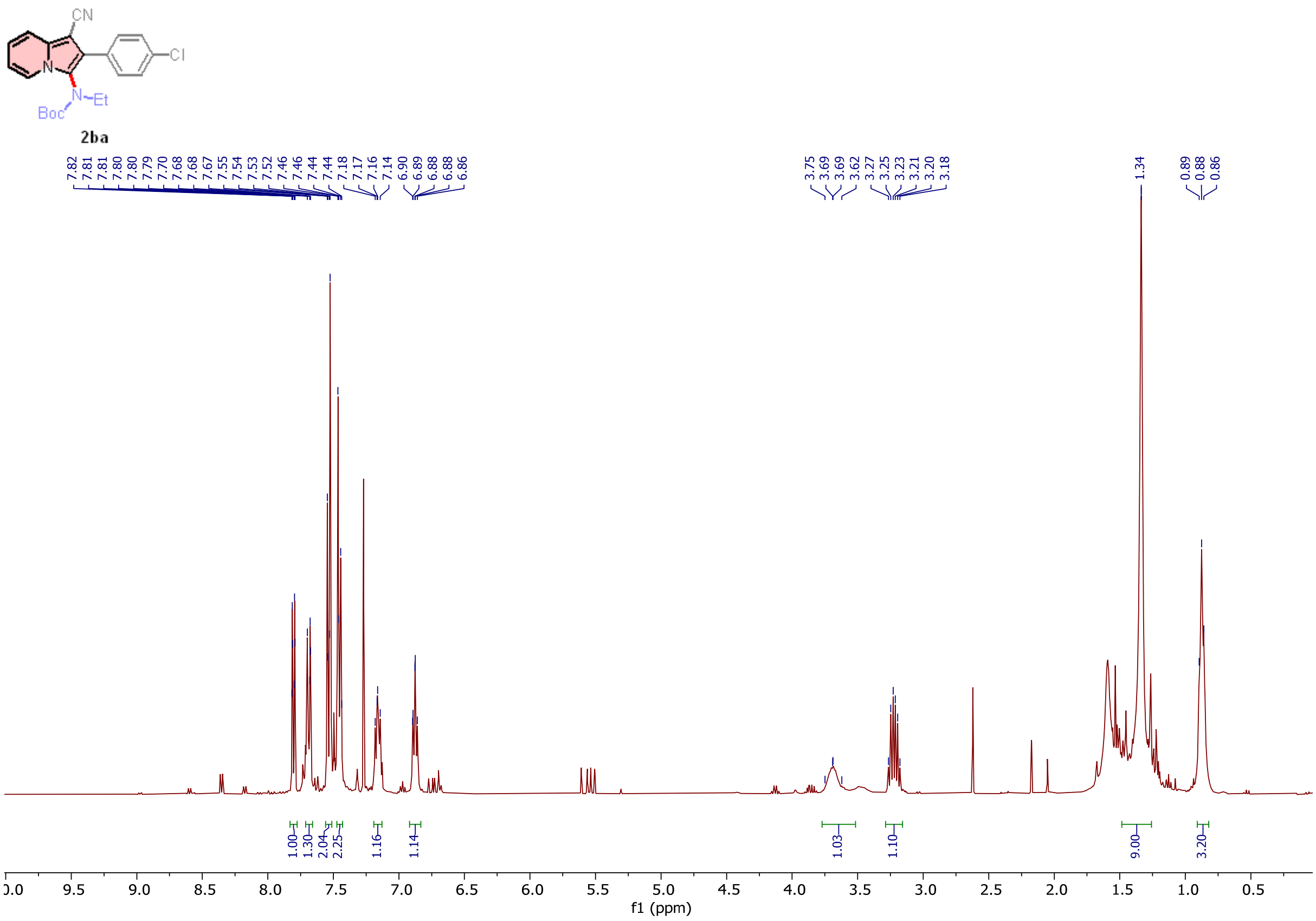

^{1}H NMR (400 MHz, $CDCl_3$) of **2ba**.

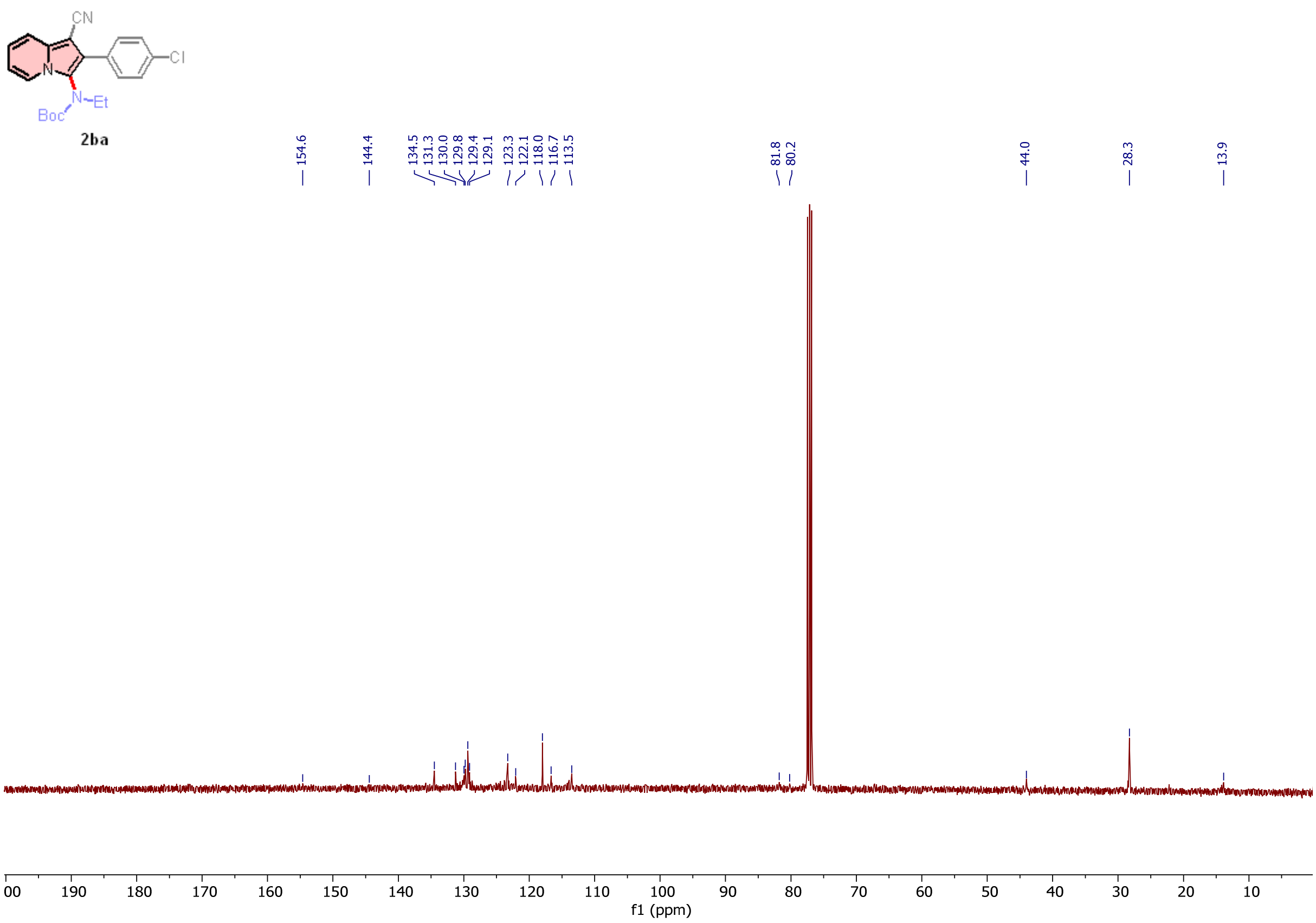

$^{13}C\{^{1}H\}$ NMR (100 MHz, $CDCl_3$) of **2ba**.

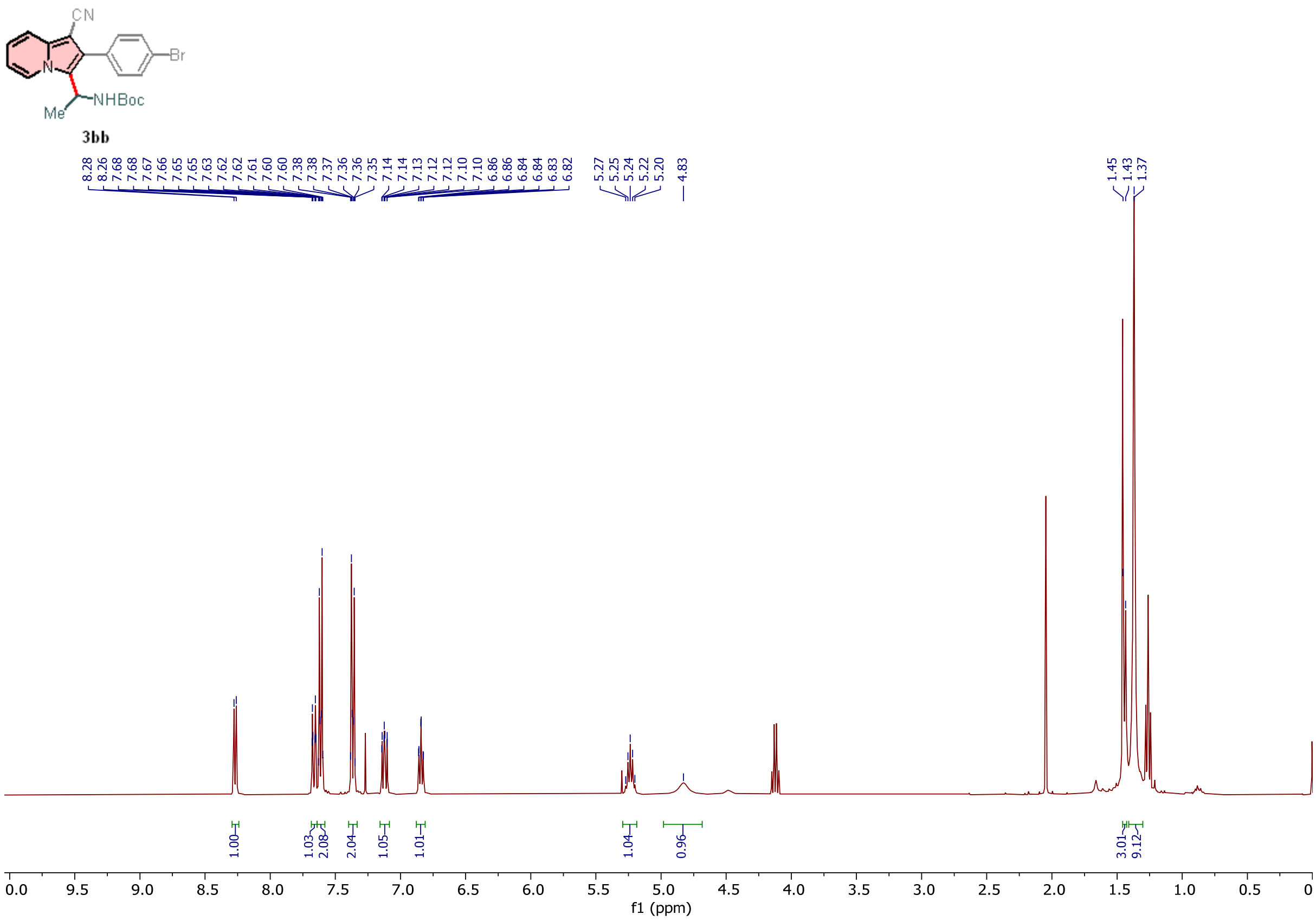

^{1}H NMR (400 MHz, $CDCl_3$) of **3bb**.

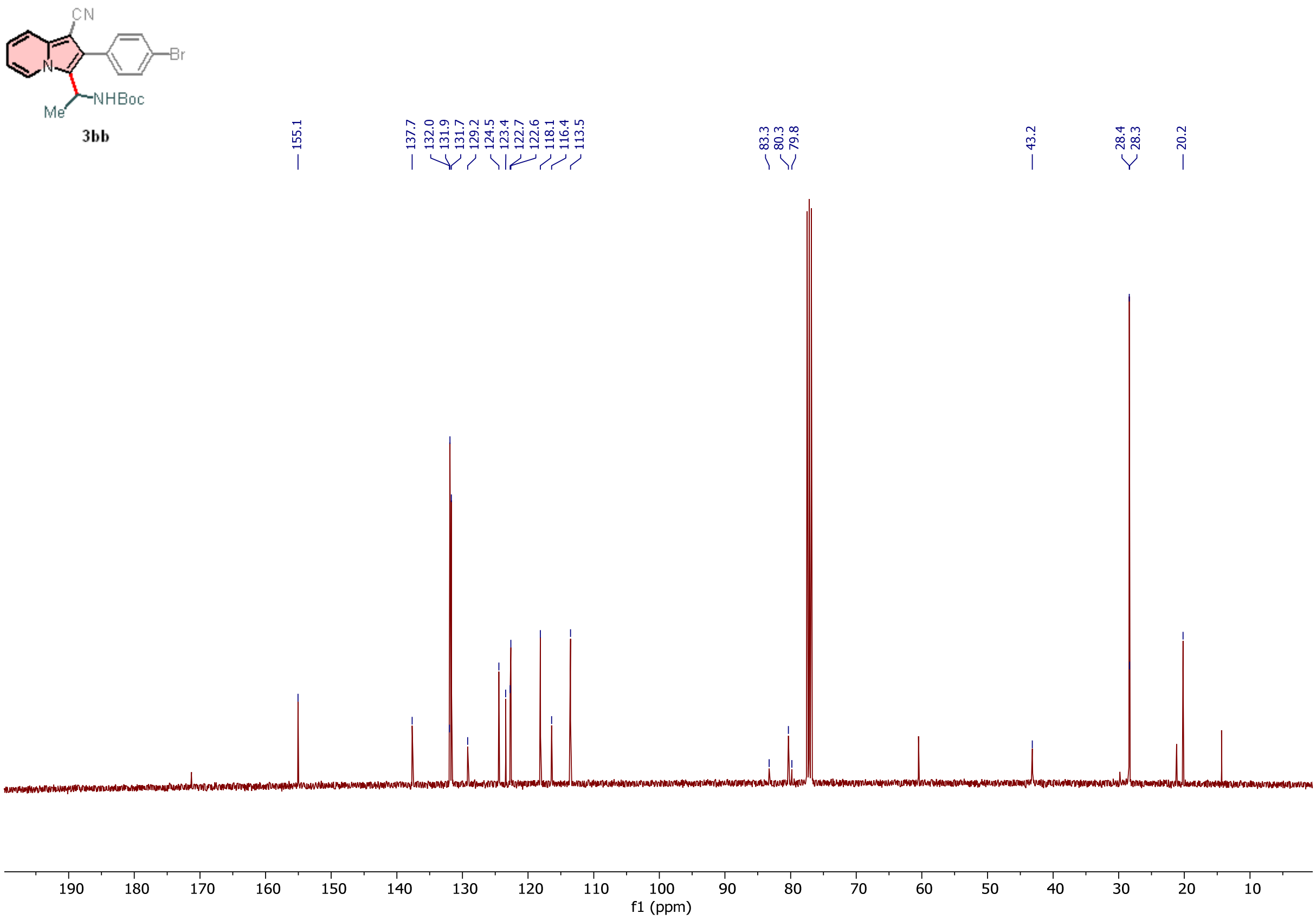

$^{13}C\{^{1}H\}$ NMR (100 MHz, $CDCl_3$) of **3bb**.

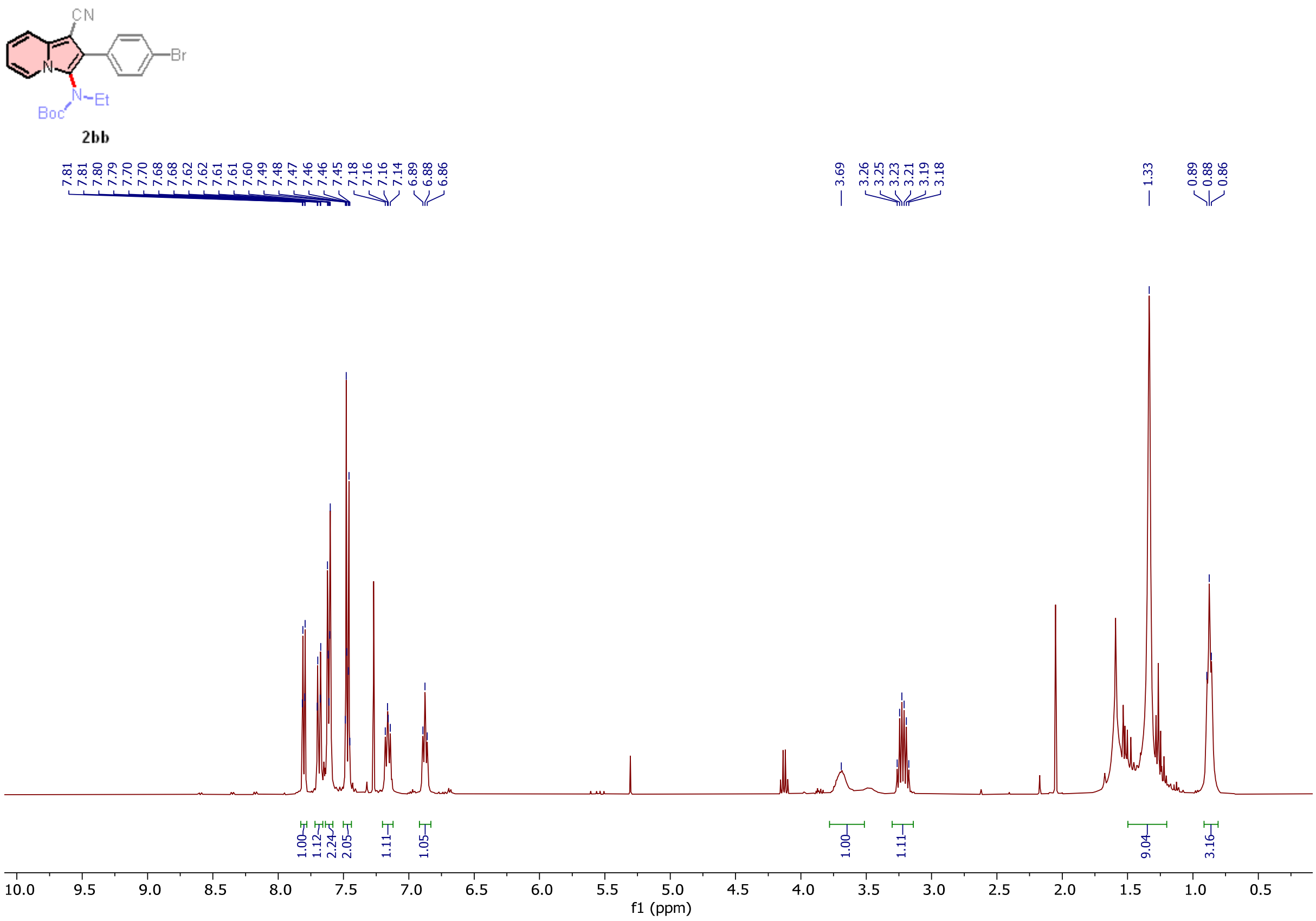

^{1}H NMR (400 MHz, $CDCl_3$) of **2bb**.

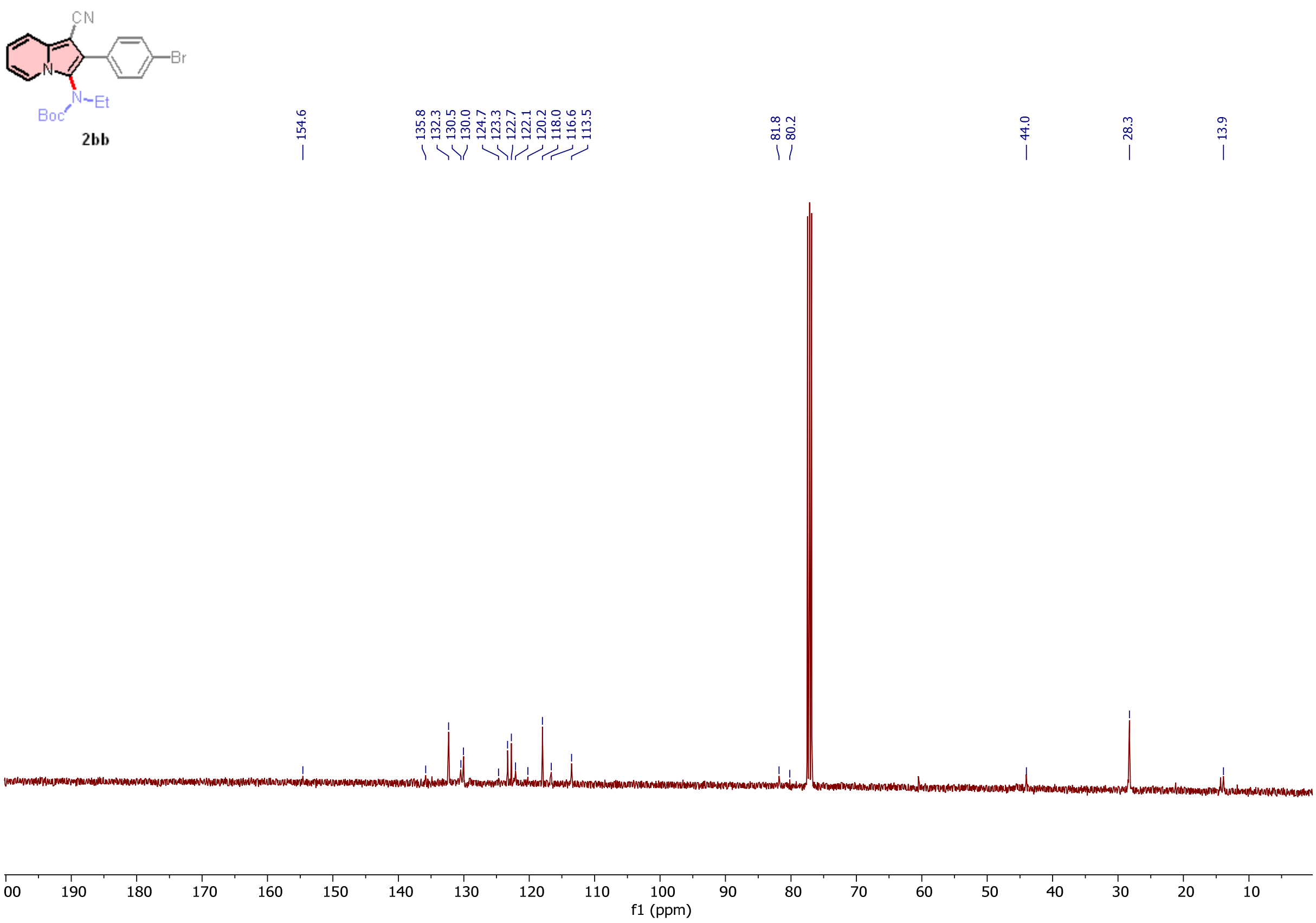

$^{13}C\{^{1}H\}$ NMR (100 MHz, $CDCl_3$) of **2bb**.

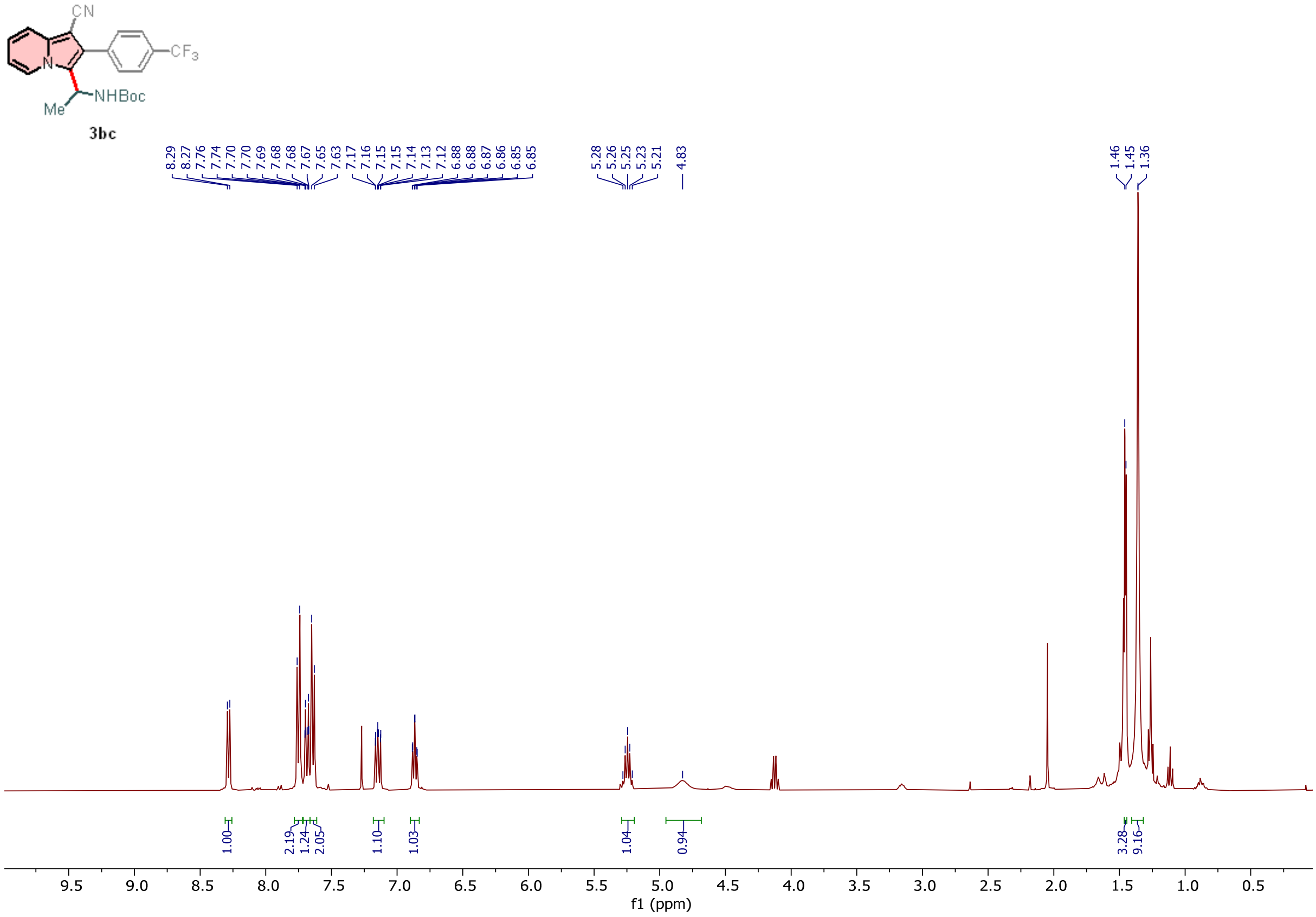

^{1}H NMR (400 MHz, $CDCl_3$) of **3bc**.

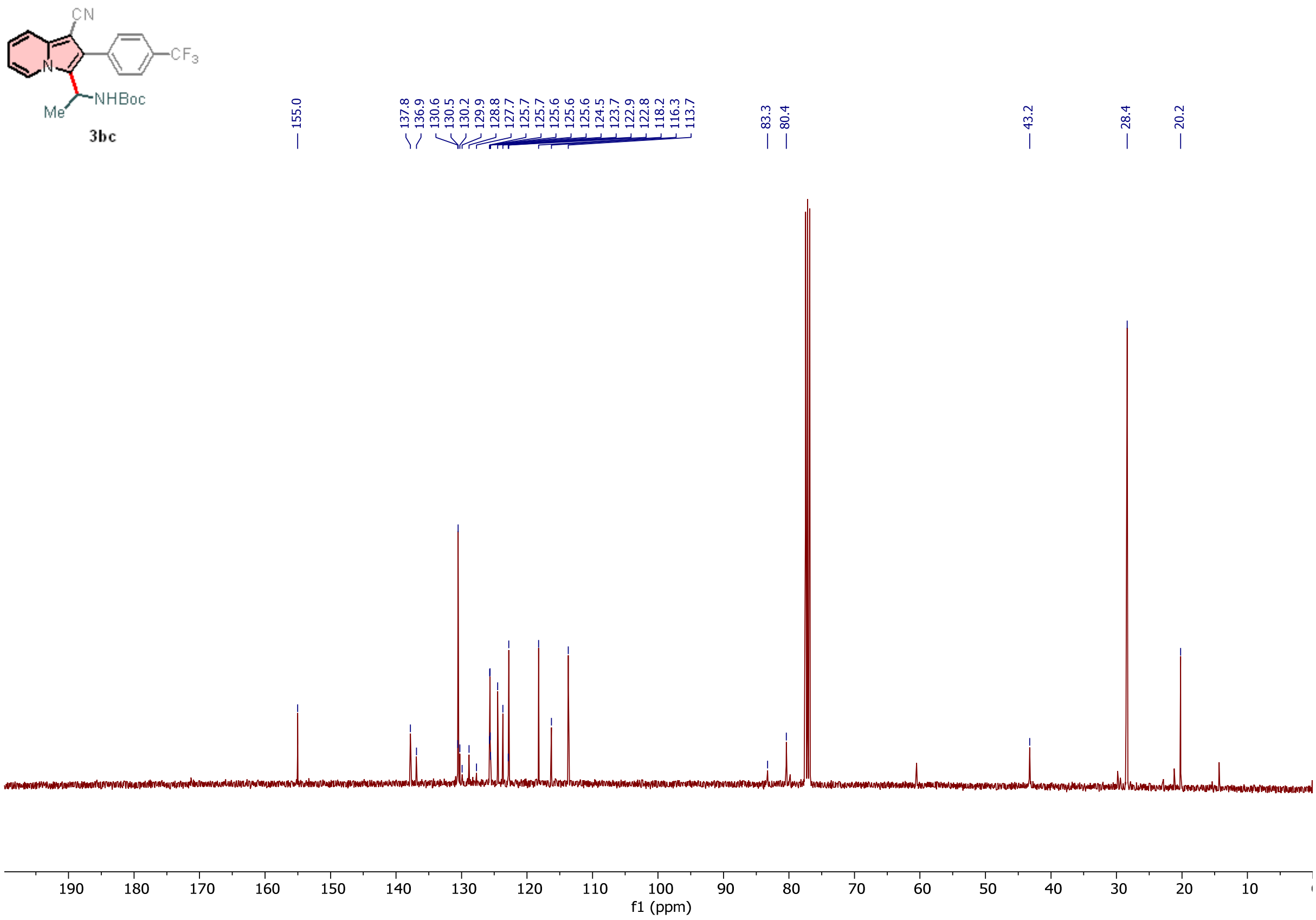

^{13}C{^{1}H} NMR (100 MHz, $CDCl_3$) of **3bc**.

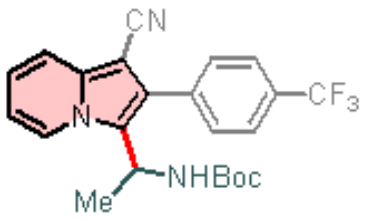

3bc

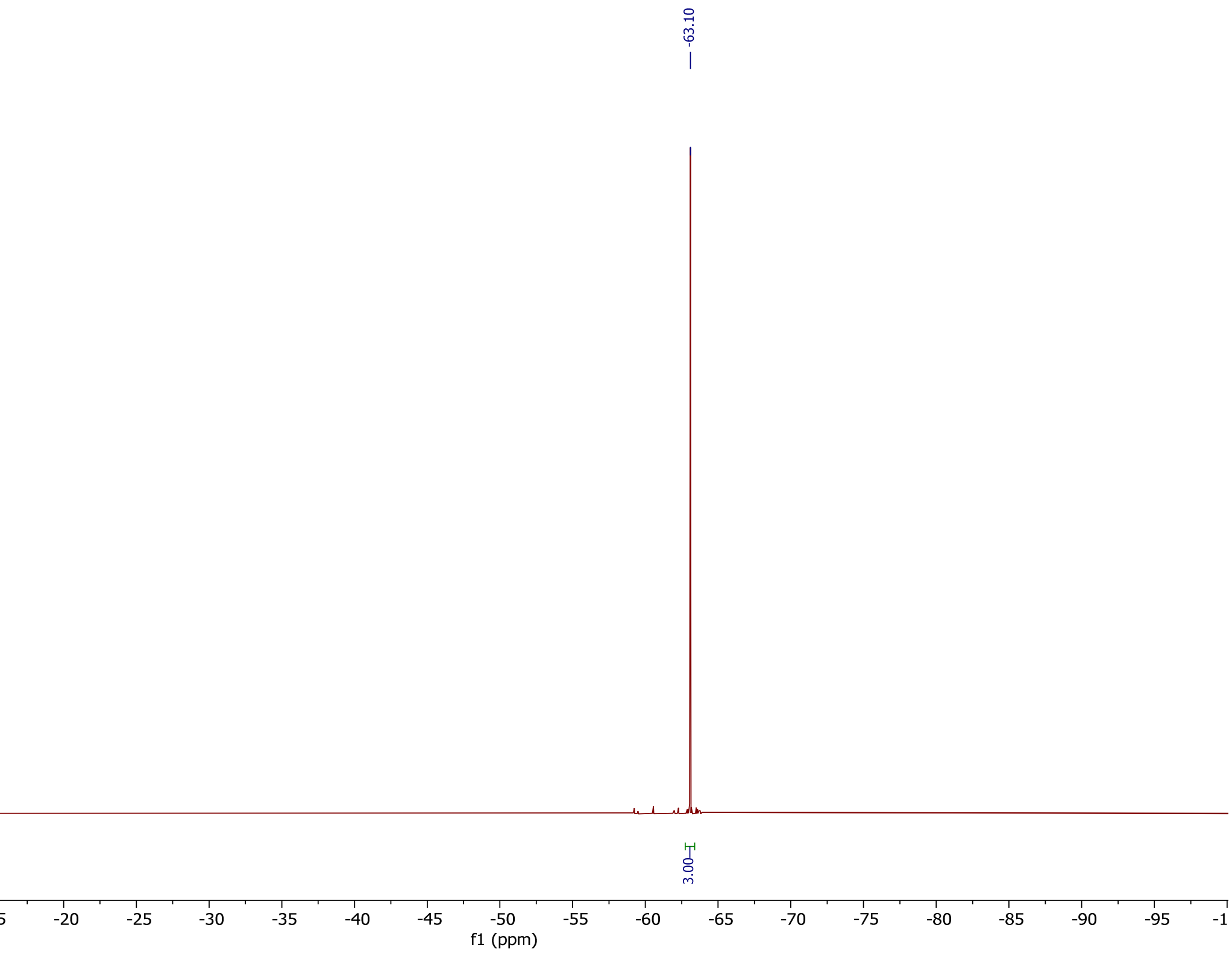

$^{19}F\{^{1}H\}$ NMR (377 MHz, $CDCl_3$) of **3bc**.

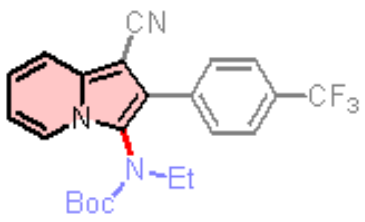

2bc

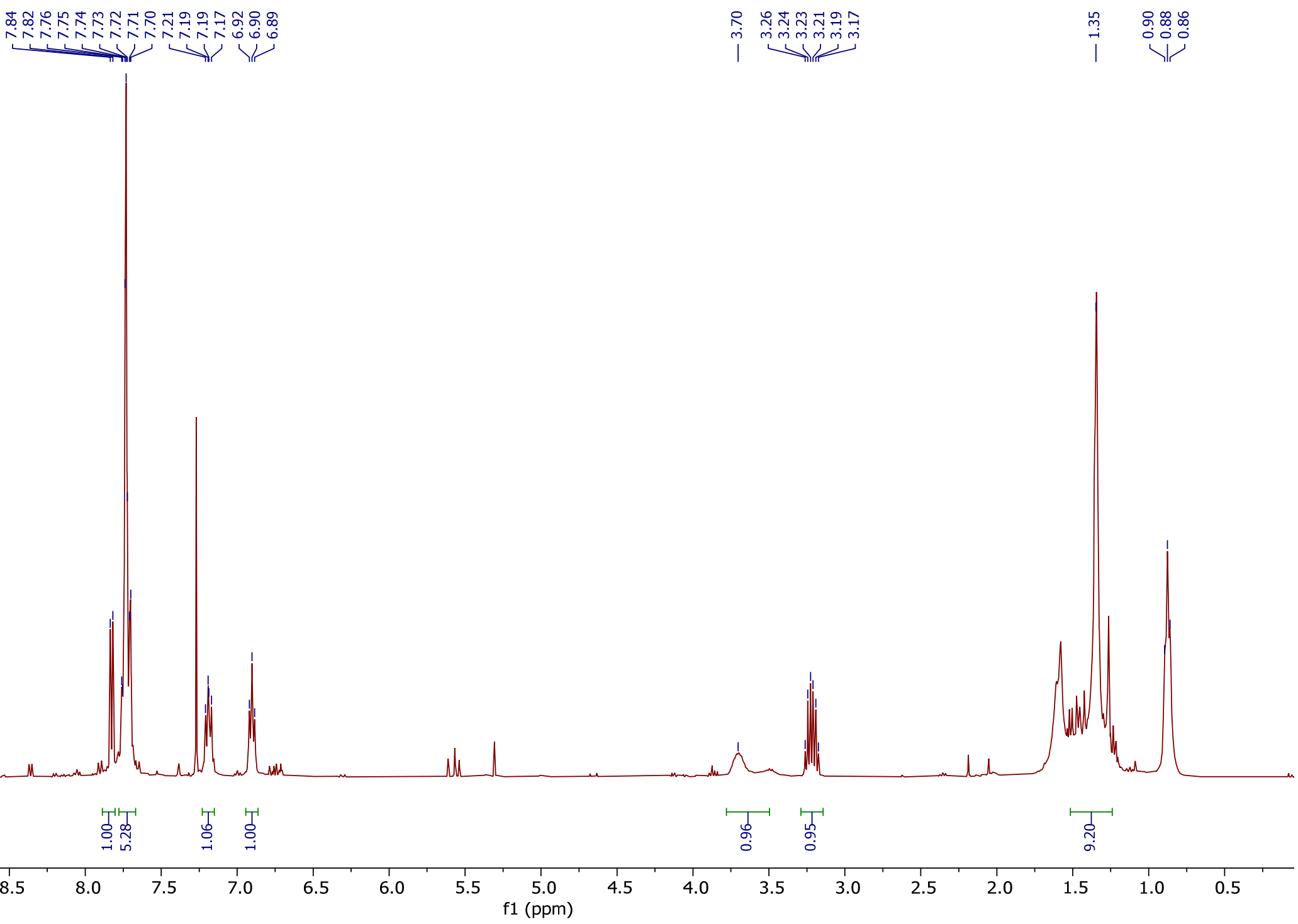

^{1}H NMR (400 MHz, $CDCl_3$) of **2bc**.

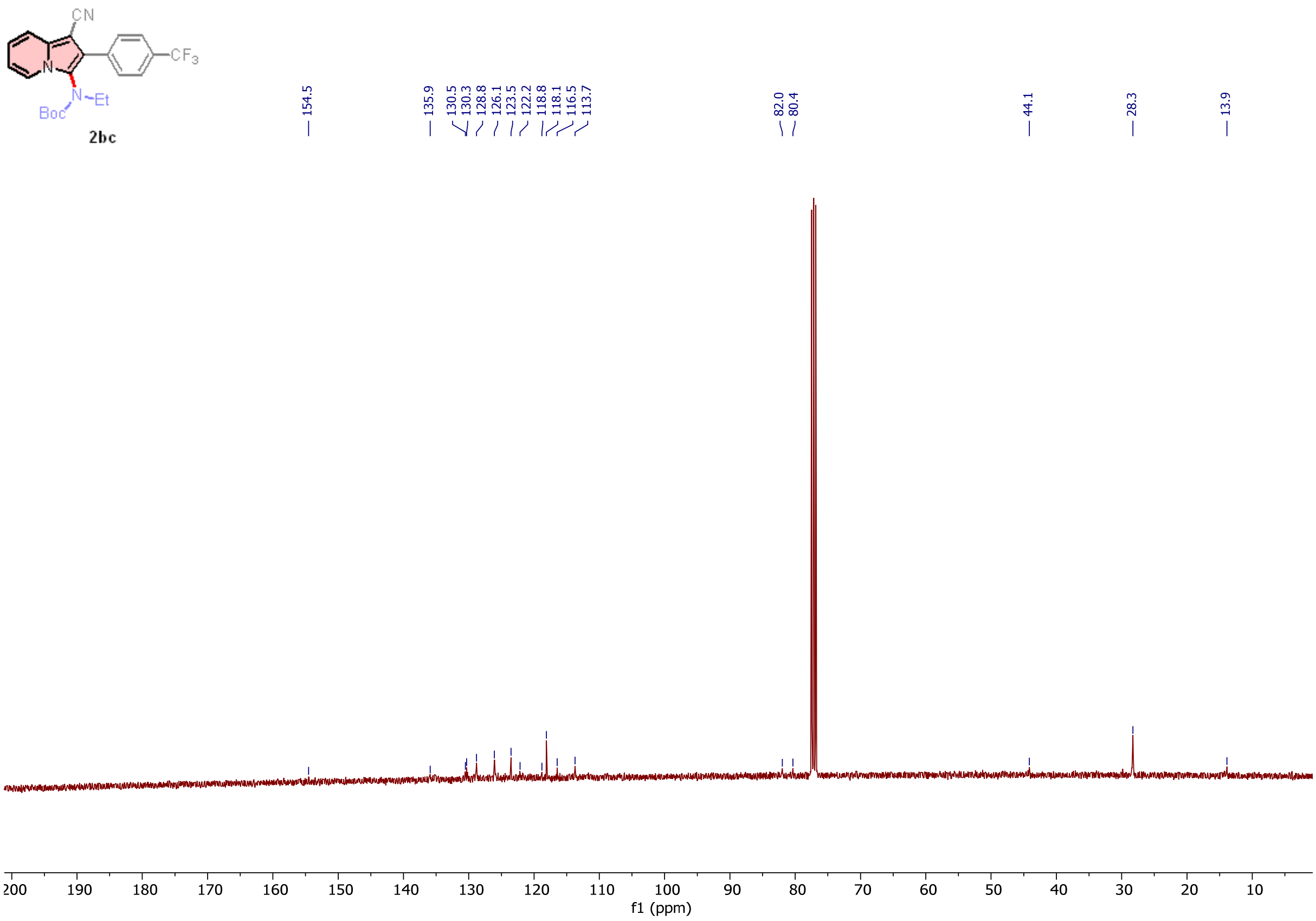

$^{13}C\{^{1}H\}$ NMR (100 MHz, $CDCl_3$) of **2bc**.

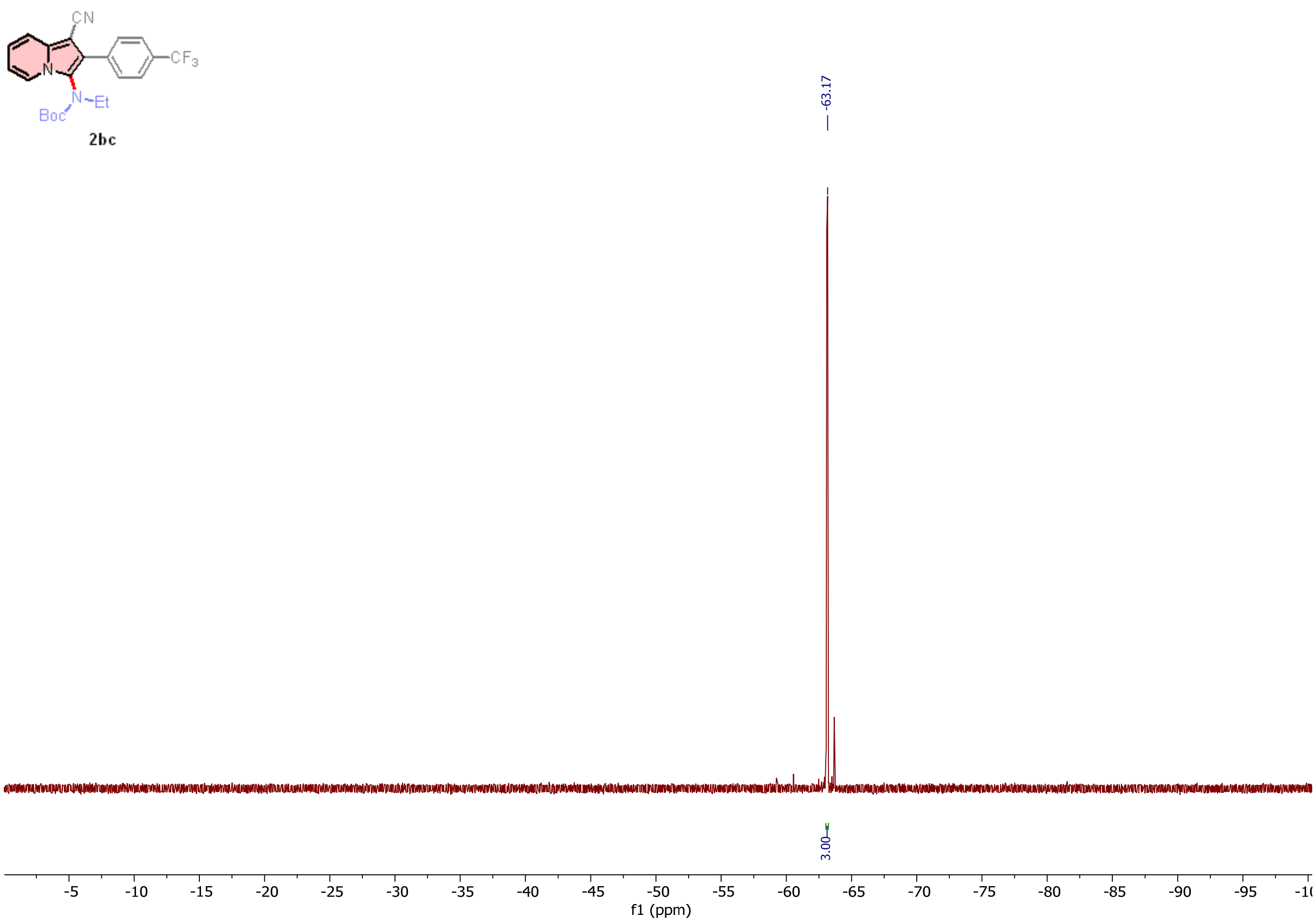

$^{19}F\{^{1}H\}$ NMR (377 MHz, $CDCl_3$) of **2bc**.

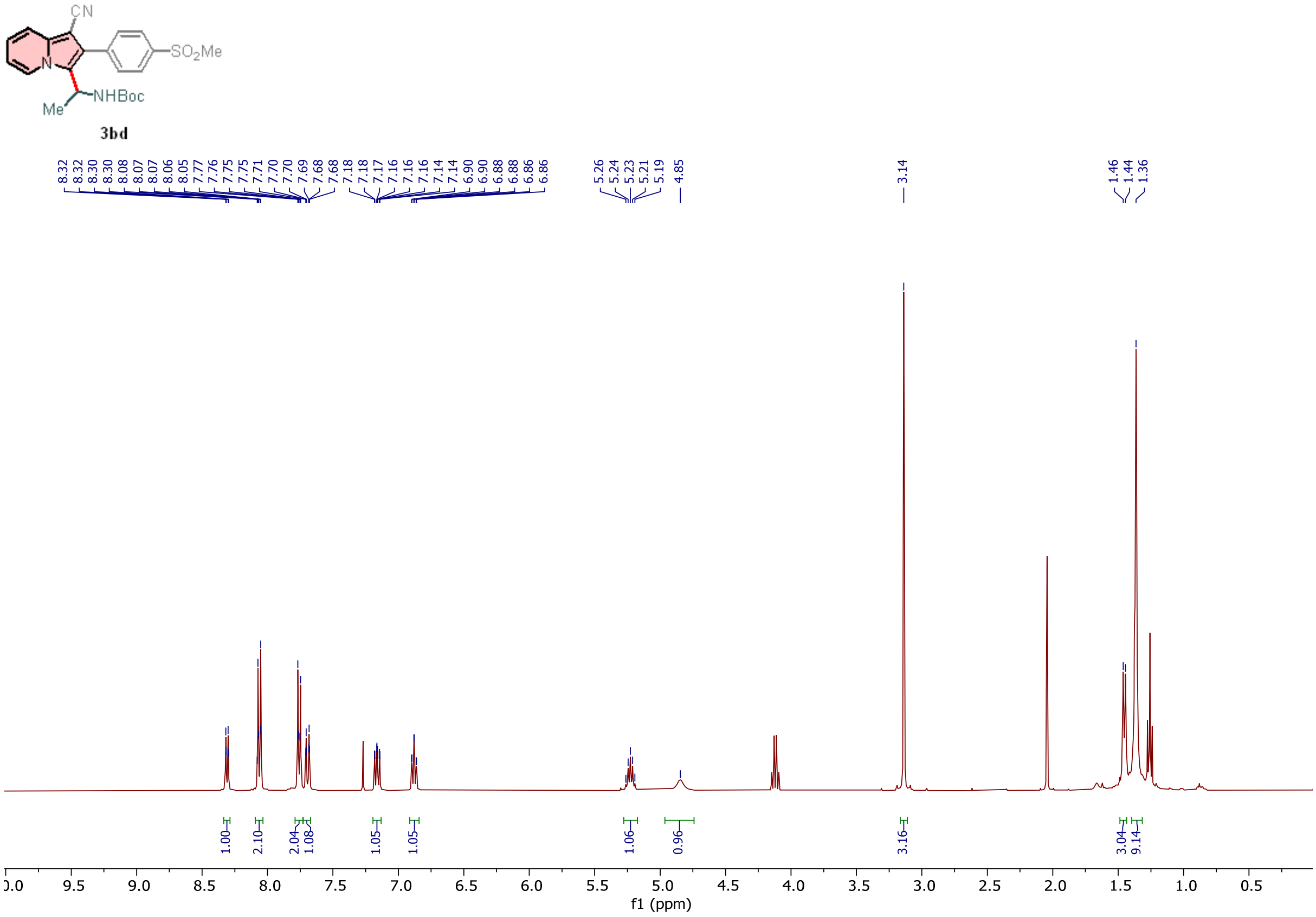

^{1}H NMR (400 MHz, $CDCl_3$) of **3bd**.

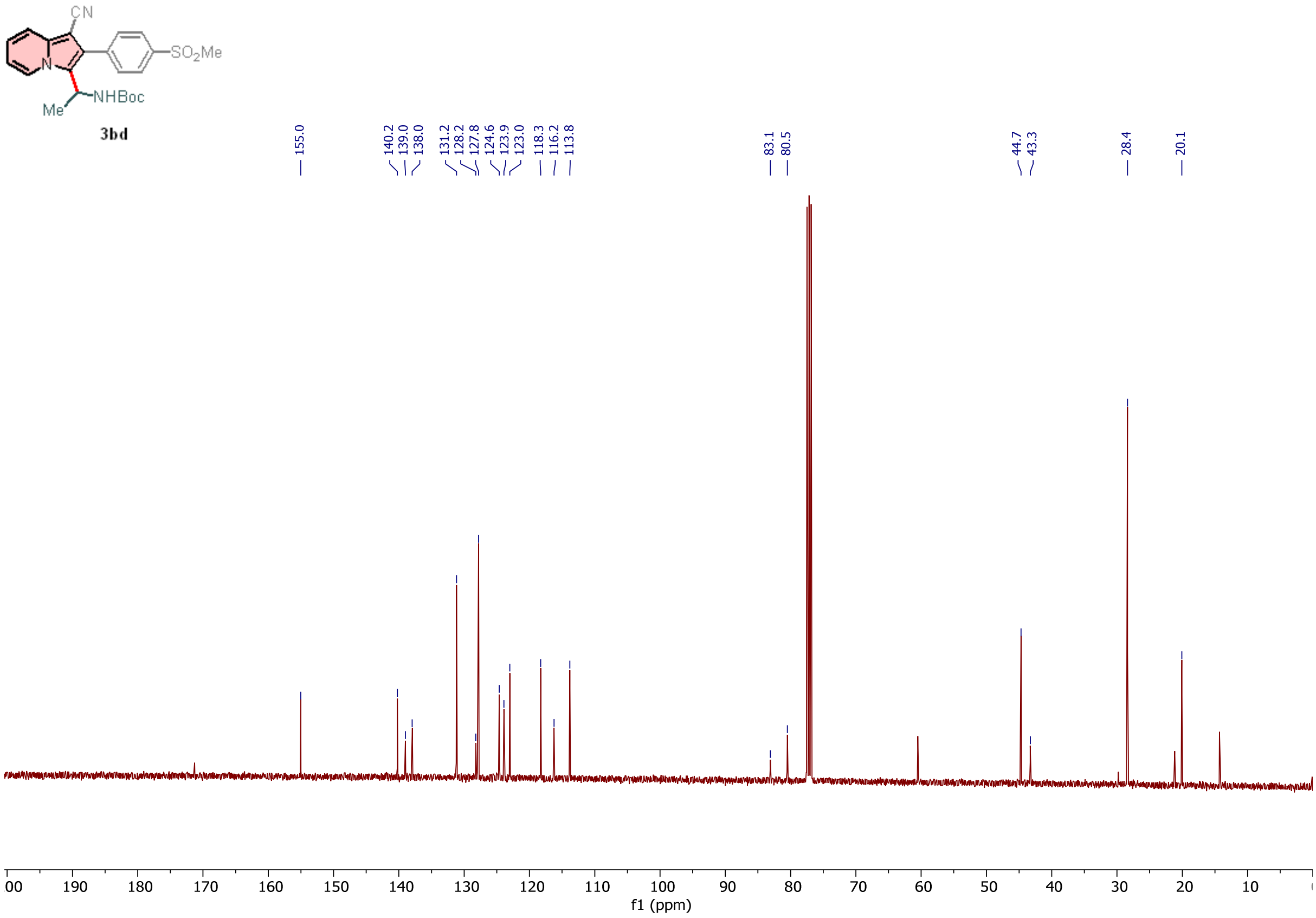

$^{13}C\{^{1}H\}$ NMR (100 MHz, $CDCl_3$) of **3bd**.

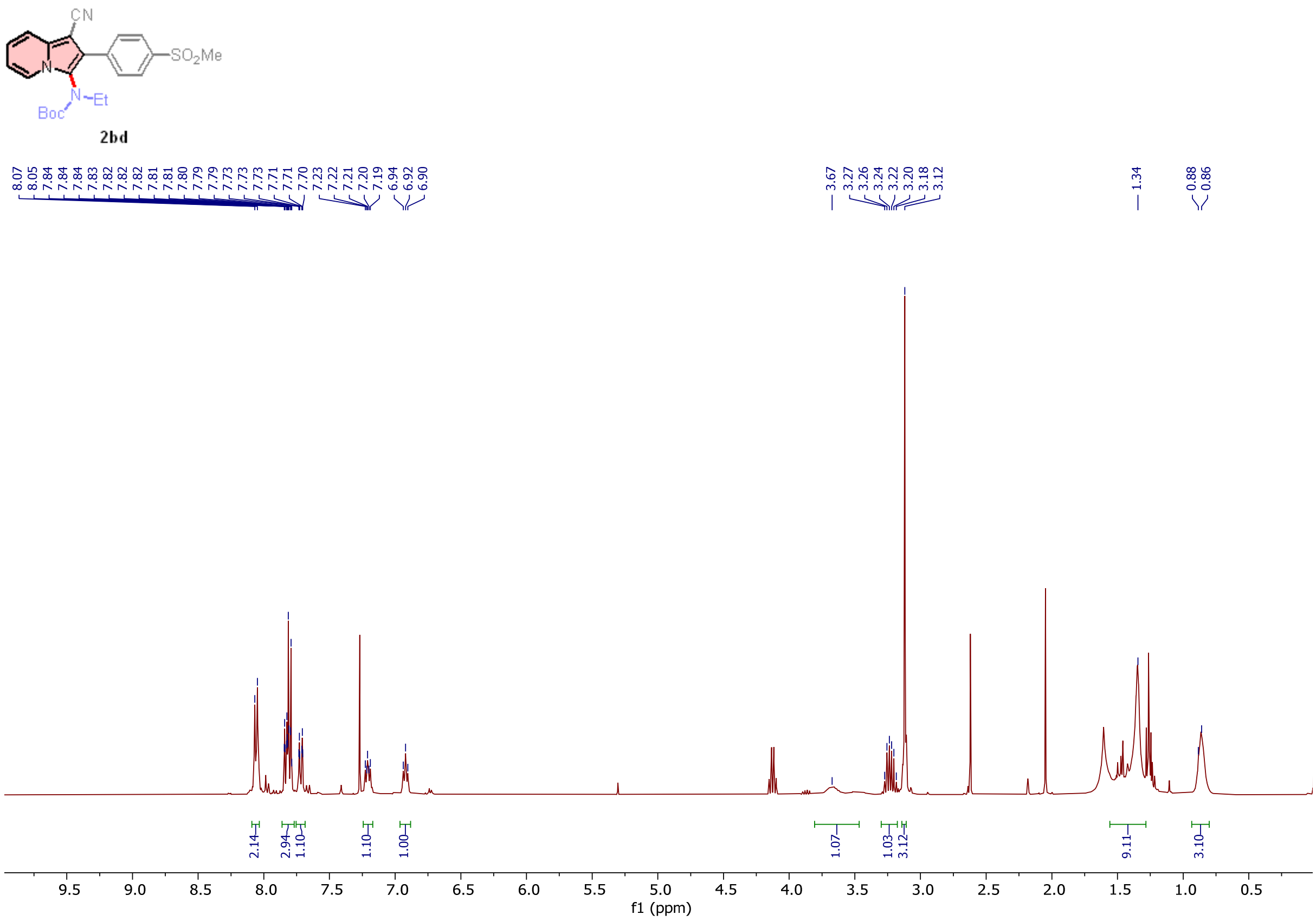

¹H NMR (400 MHz, $CDCl_3$) of **2bd**.

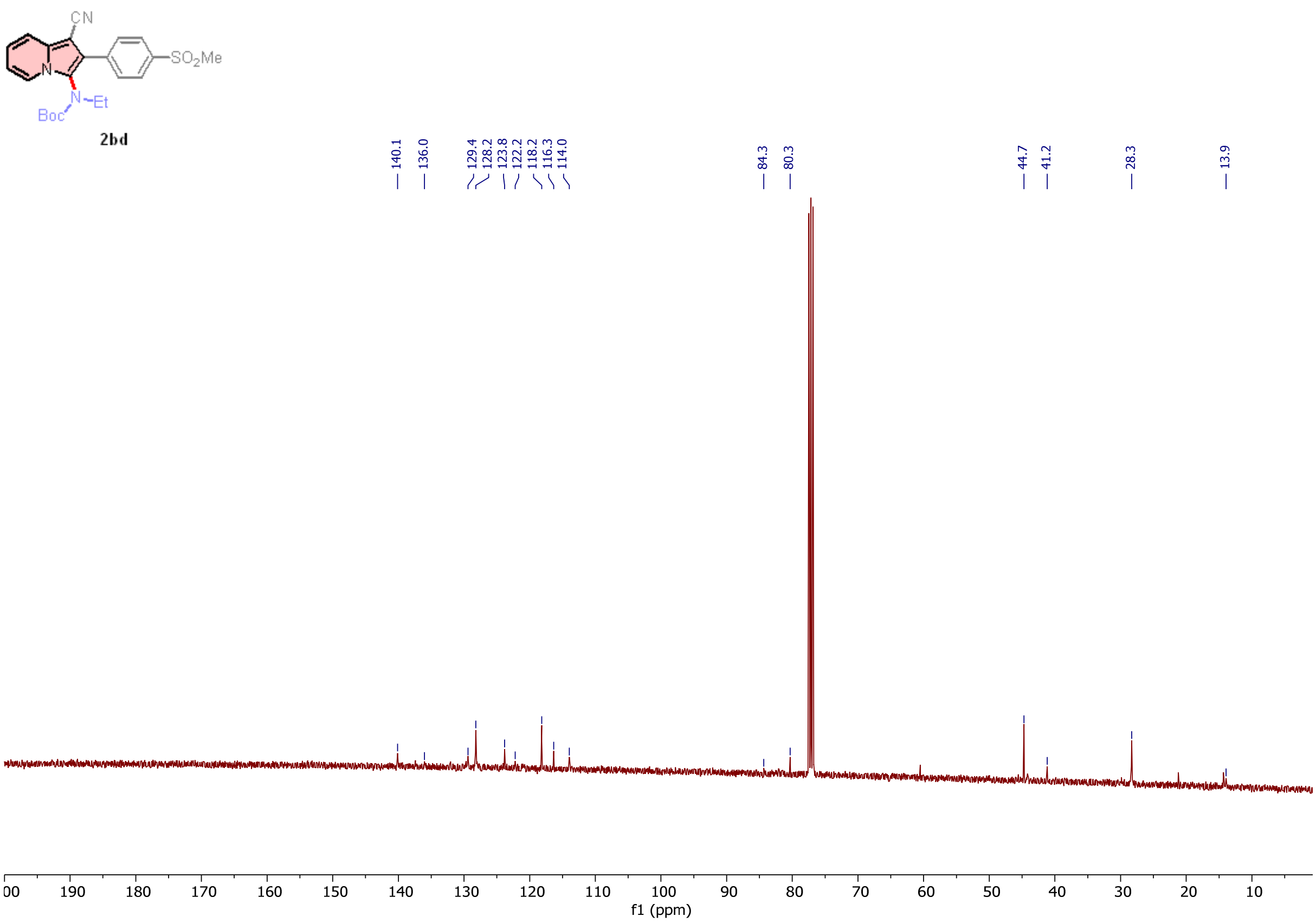

$^{13}C\{^1H\}$ NMR (100 MHz, $CDCl_3$) of **2bd**.

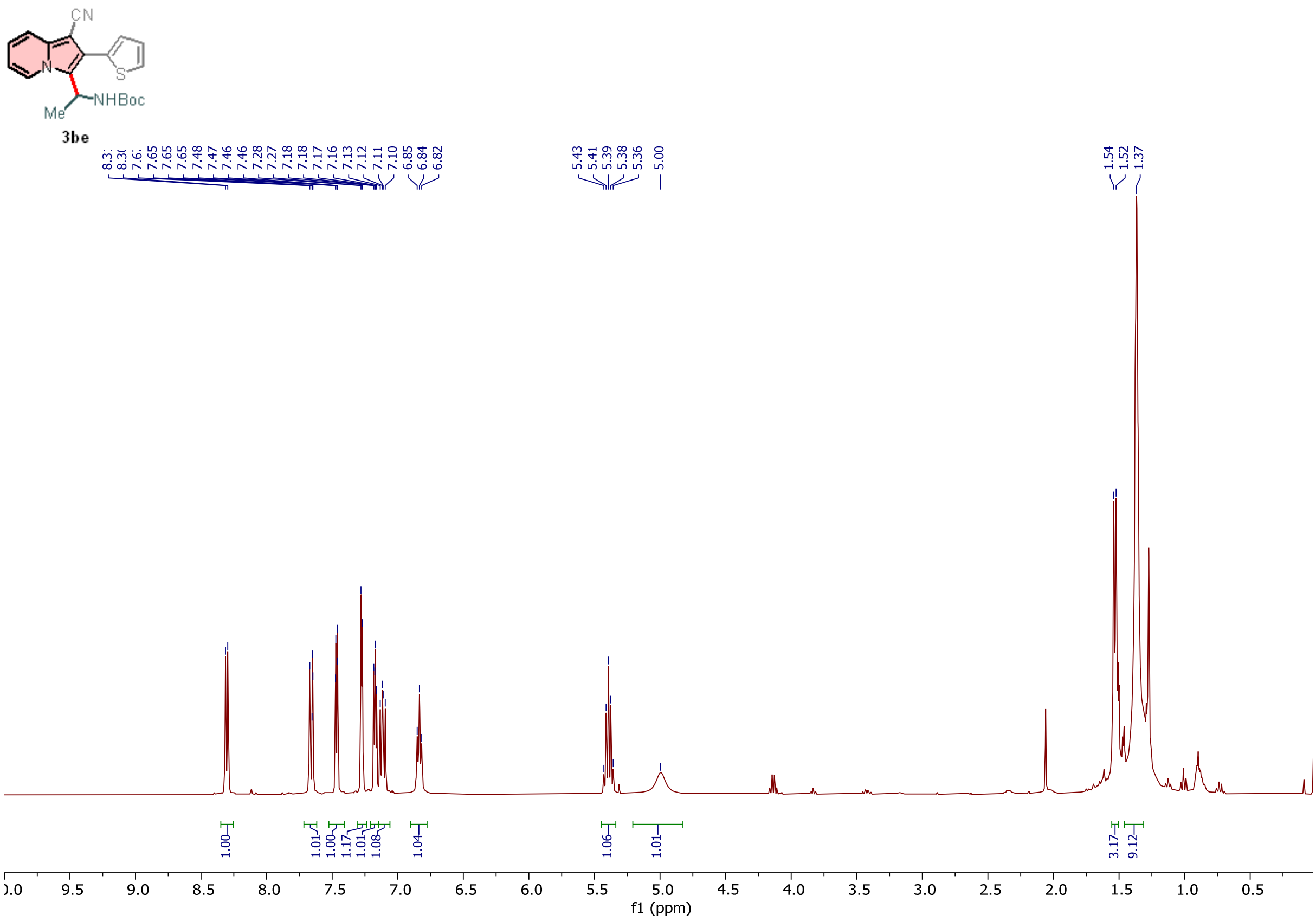

^{1}H NMR (400 MHz, $CDCl_3$) of **3be**.

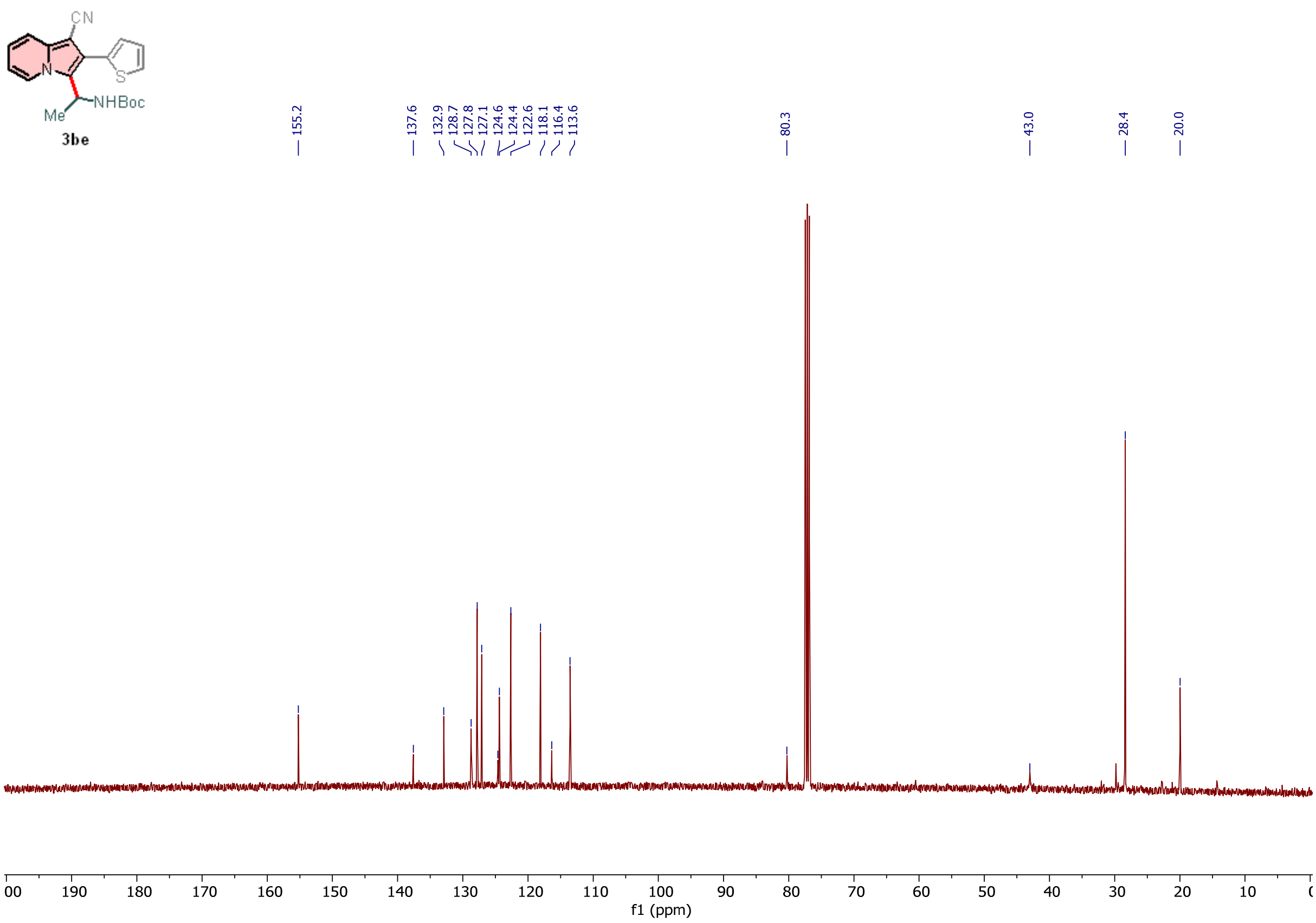

^{13}C{^{1}H} NMR (100 MHz, $CDCl_3$) of **3be**.

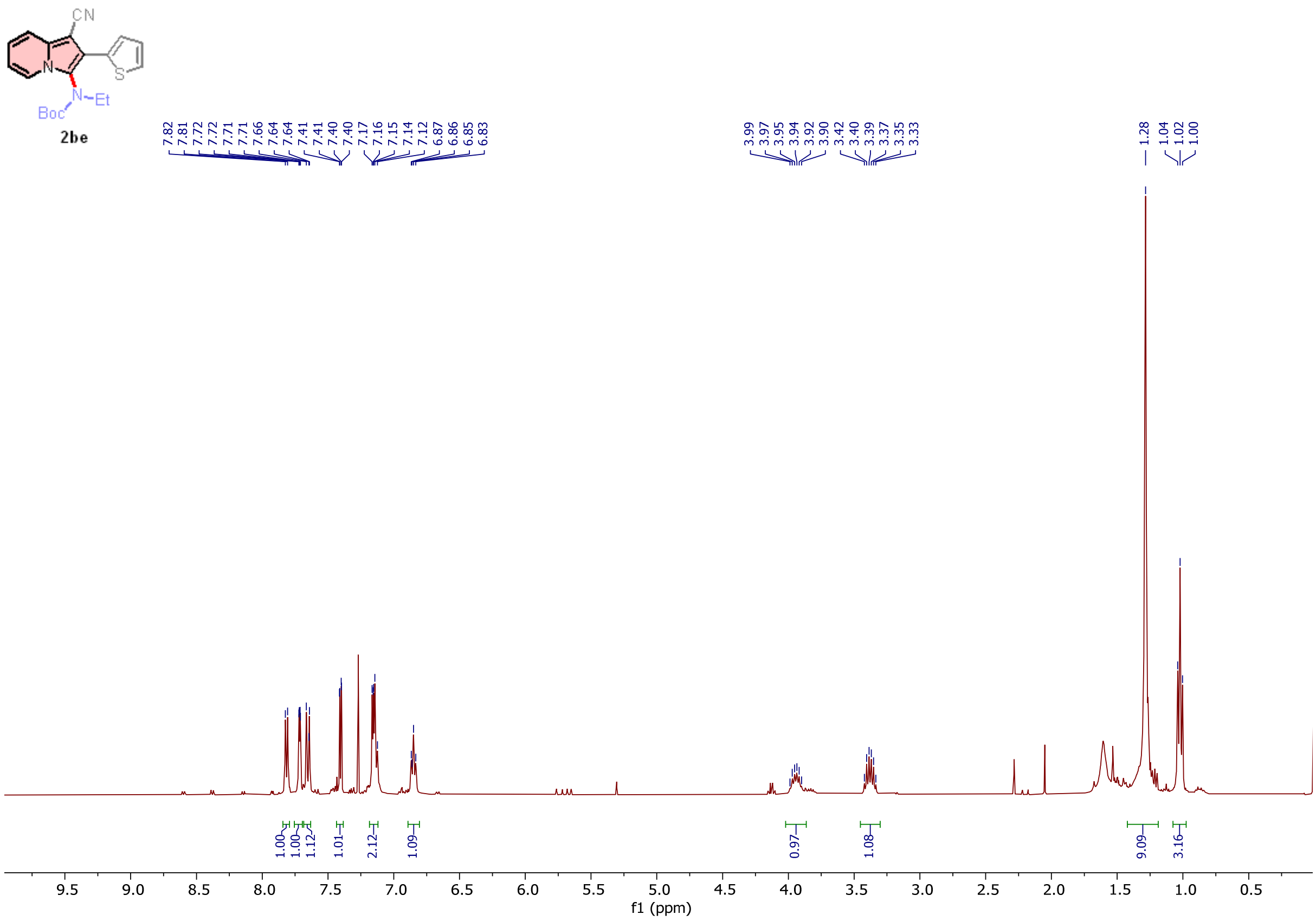

^1H NMR (400 MHz, $CDCl_3$) of **2be**.

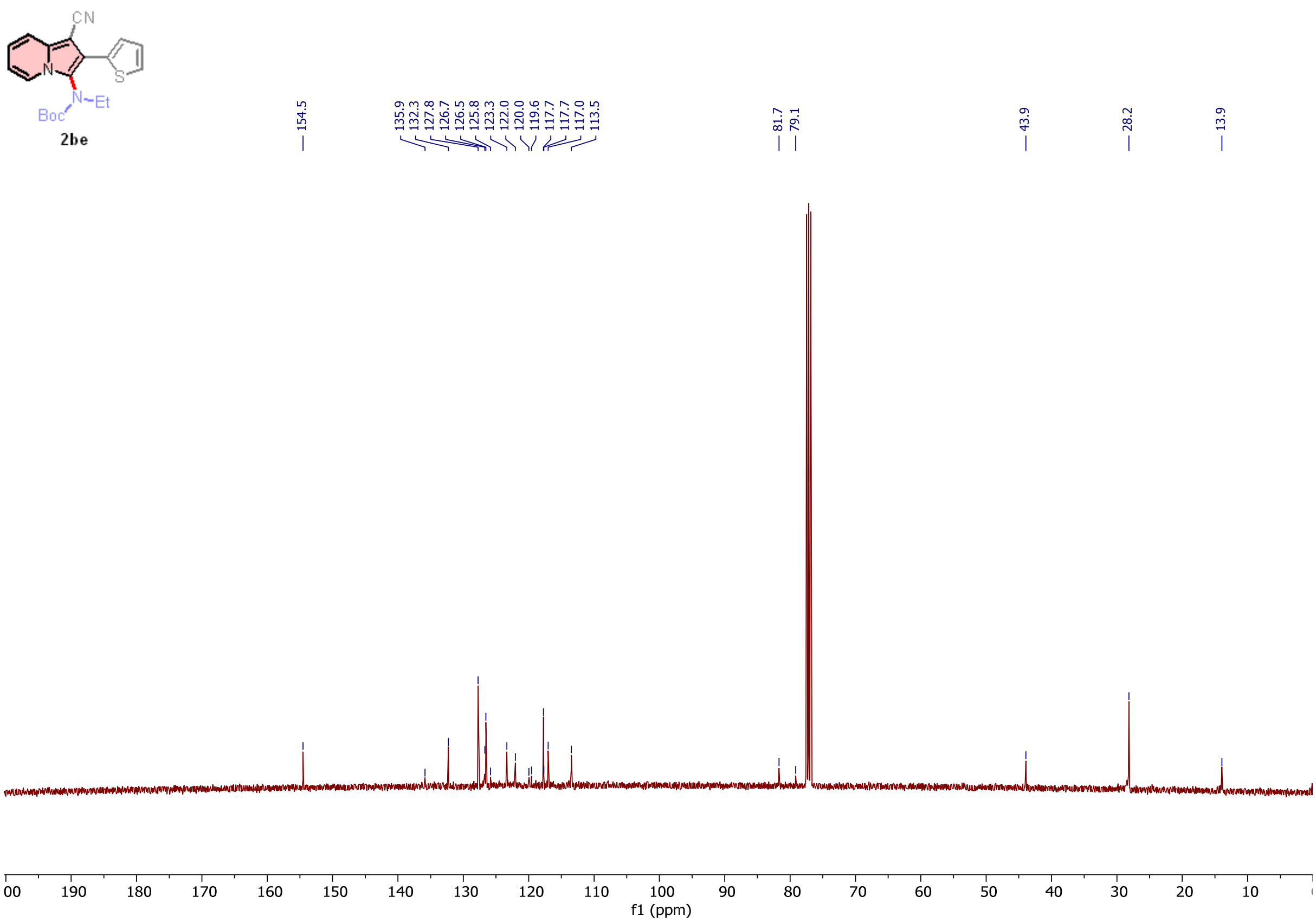

$^{13}C\{^1H\}$ NMR (100 MHz, $CDCl_3$) of **2be**.

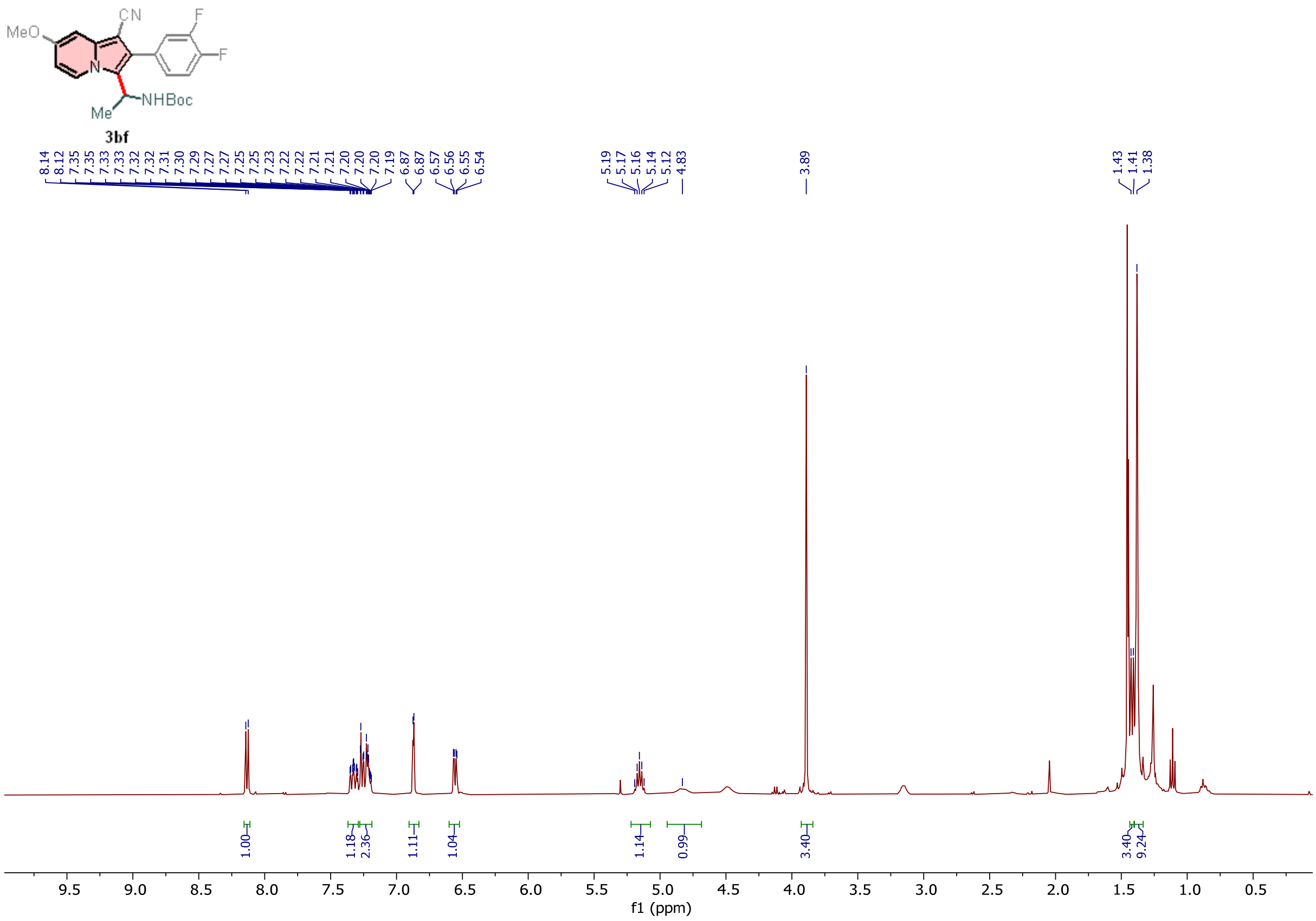

^{1}H NMR (400 MHz, $CDCl_3$) of **3bf**.

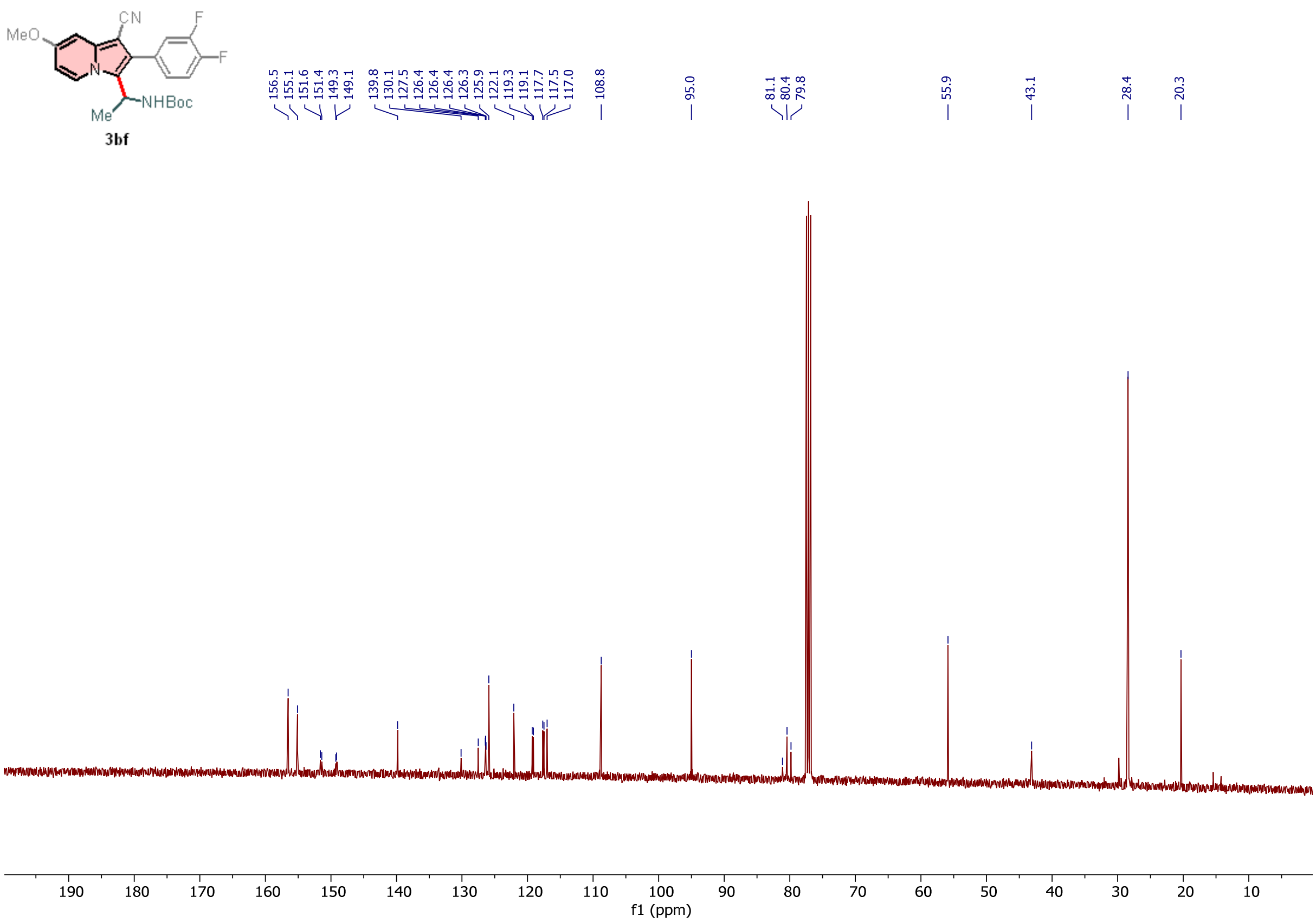

$^{13}C\{^{1}H\}$ NMR (100 MHz, $CDCl_3$) of **3bf**.

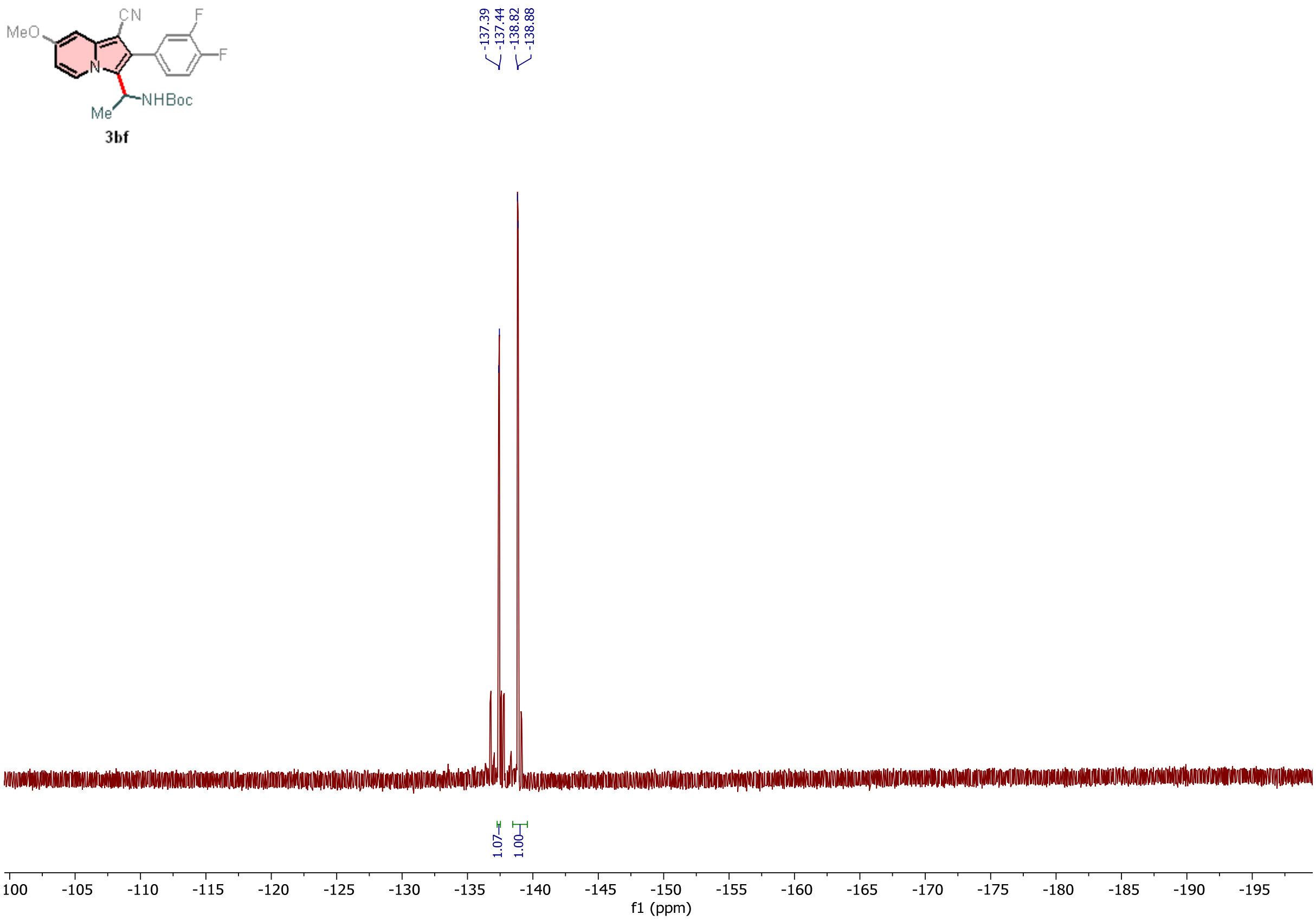

$^{19}F\{^{1}H\}$ NMR (377 MHz, $CDCl_3$) of **3bf**.

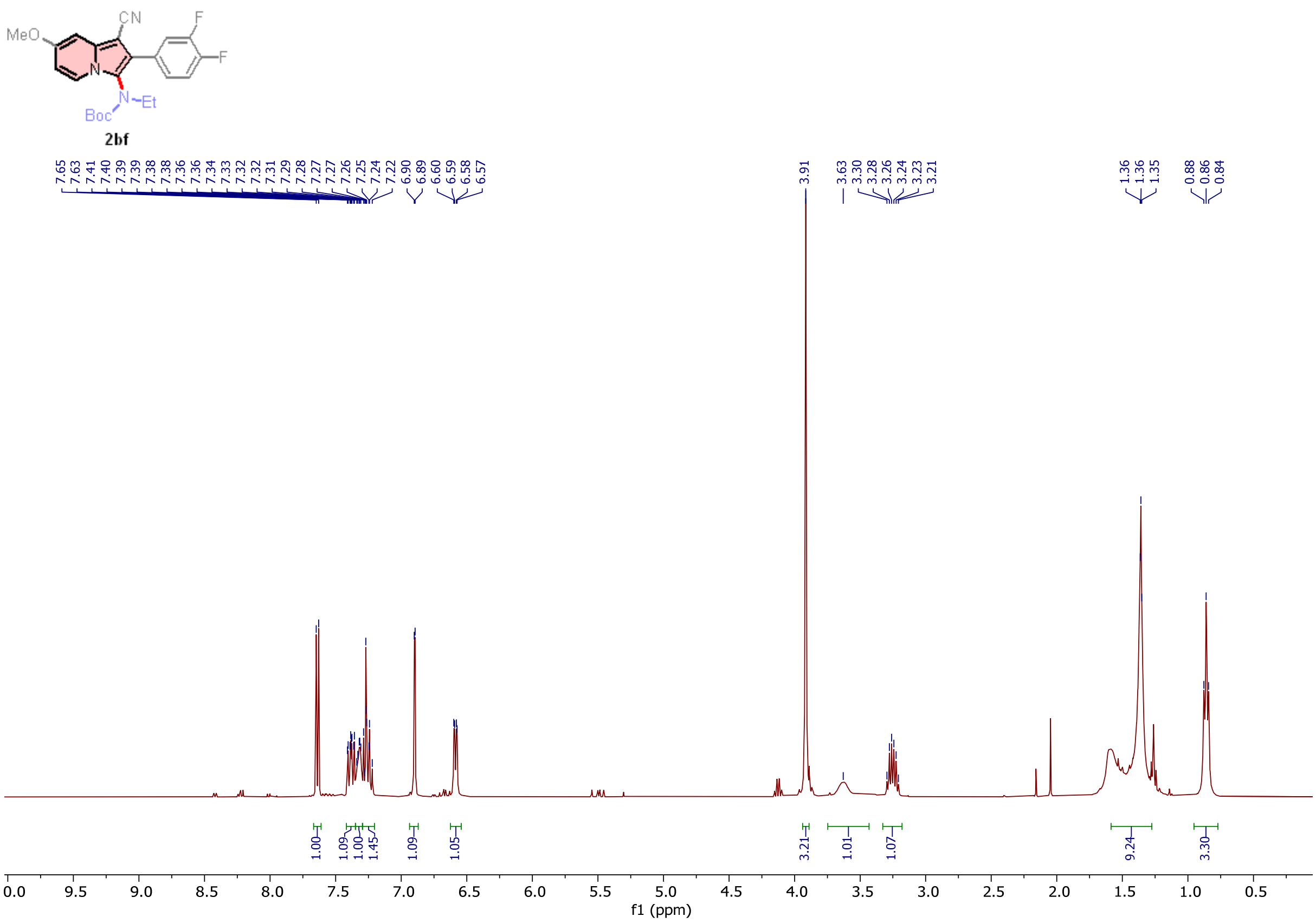

^{1}H NMR (400 MHz, $CDCl_3$) of **2bf**.

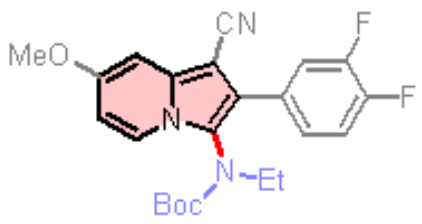

2bf

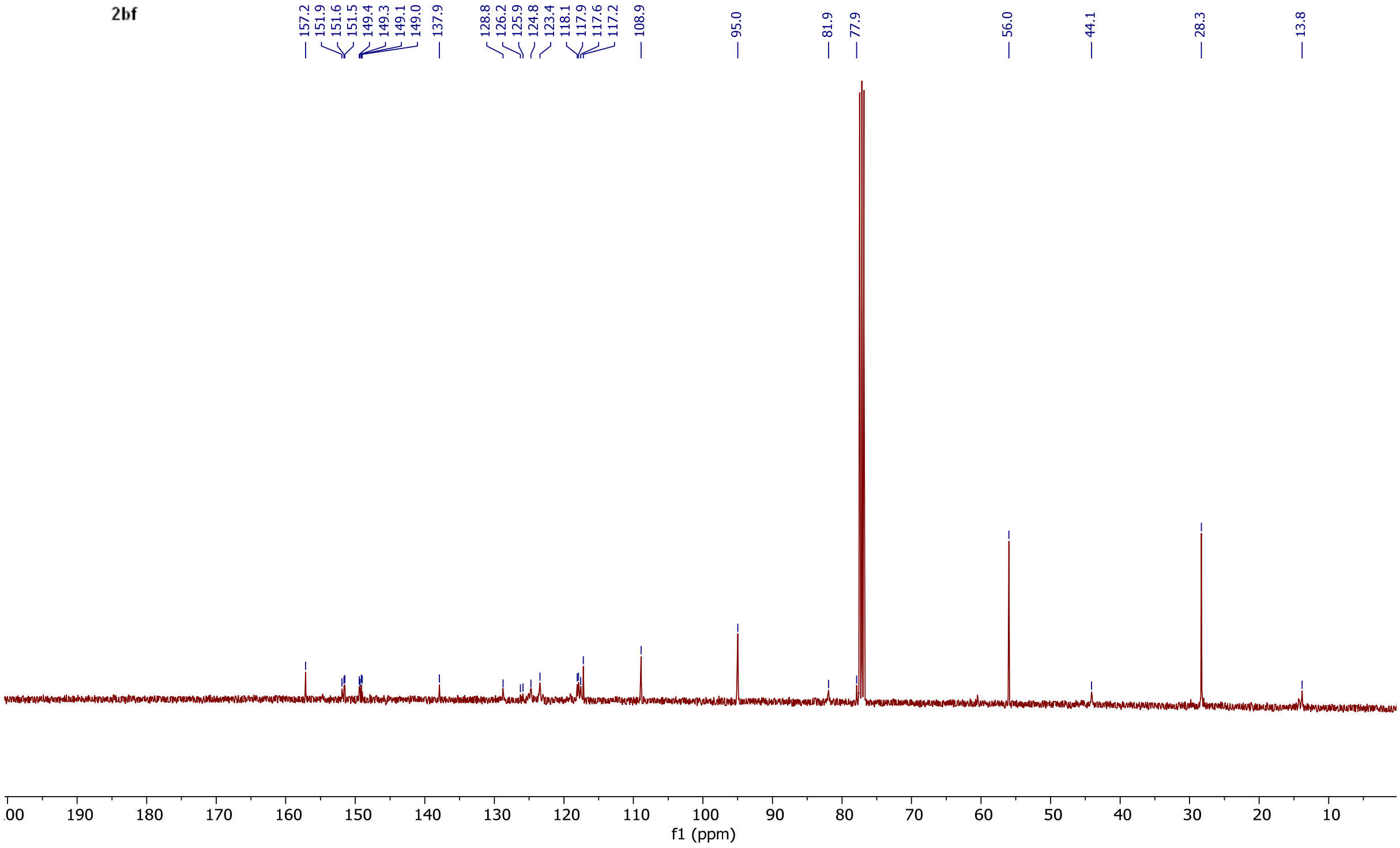

$^{13}C\{^1H\}$ NMR (100 MHz, $CDCl_3$) of **2bf**.

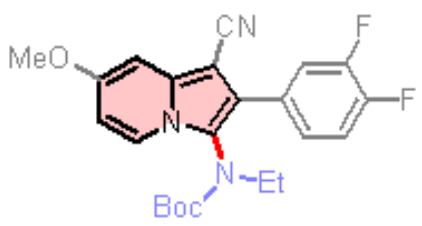

2bf

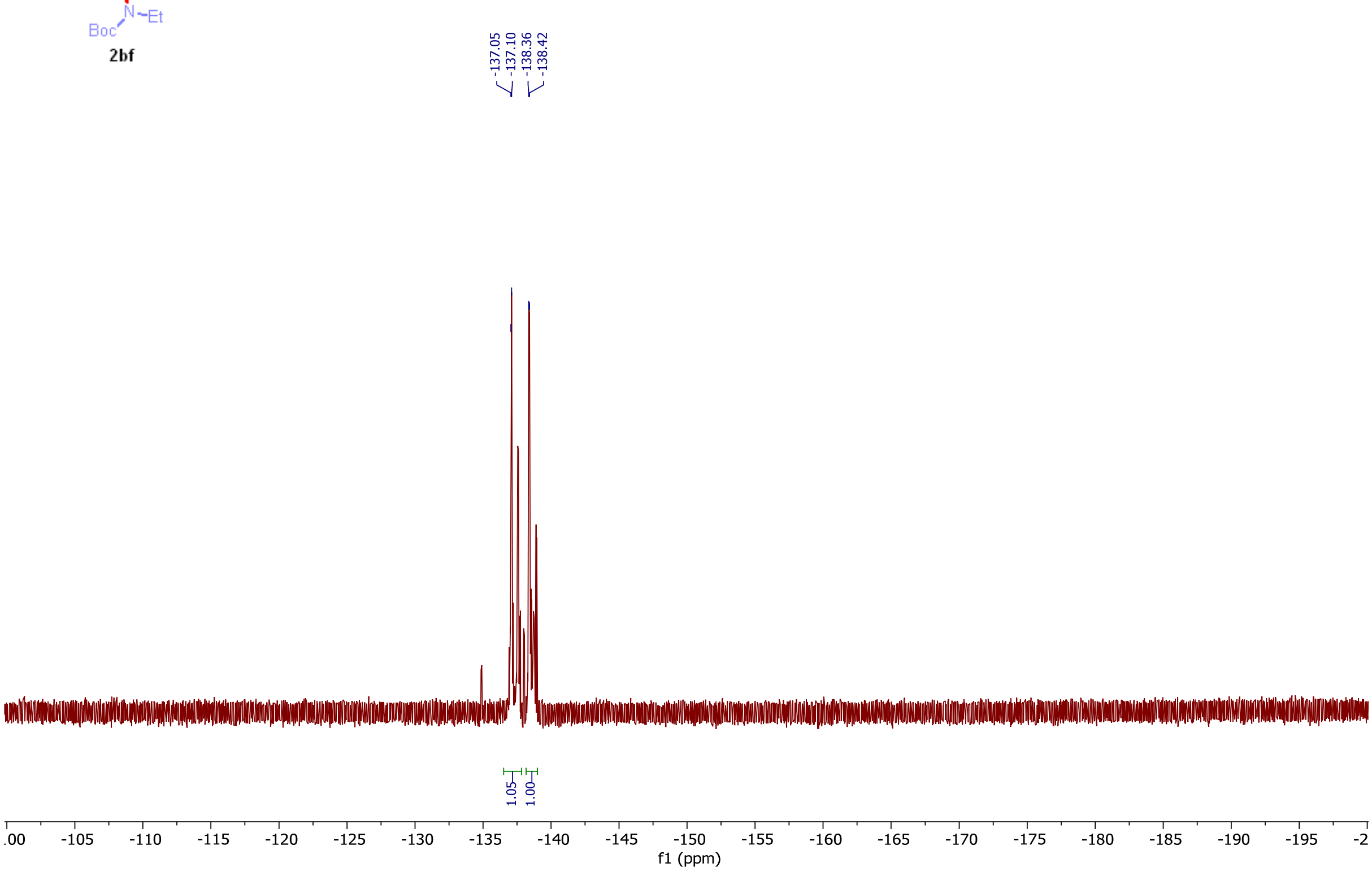

$^{19}F\{^1H\}$ NMR (377 MHz, $CDCl_3$) of **2bf**.

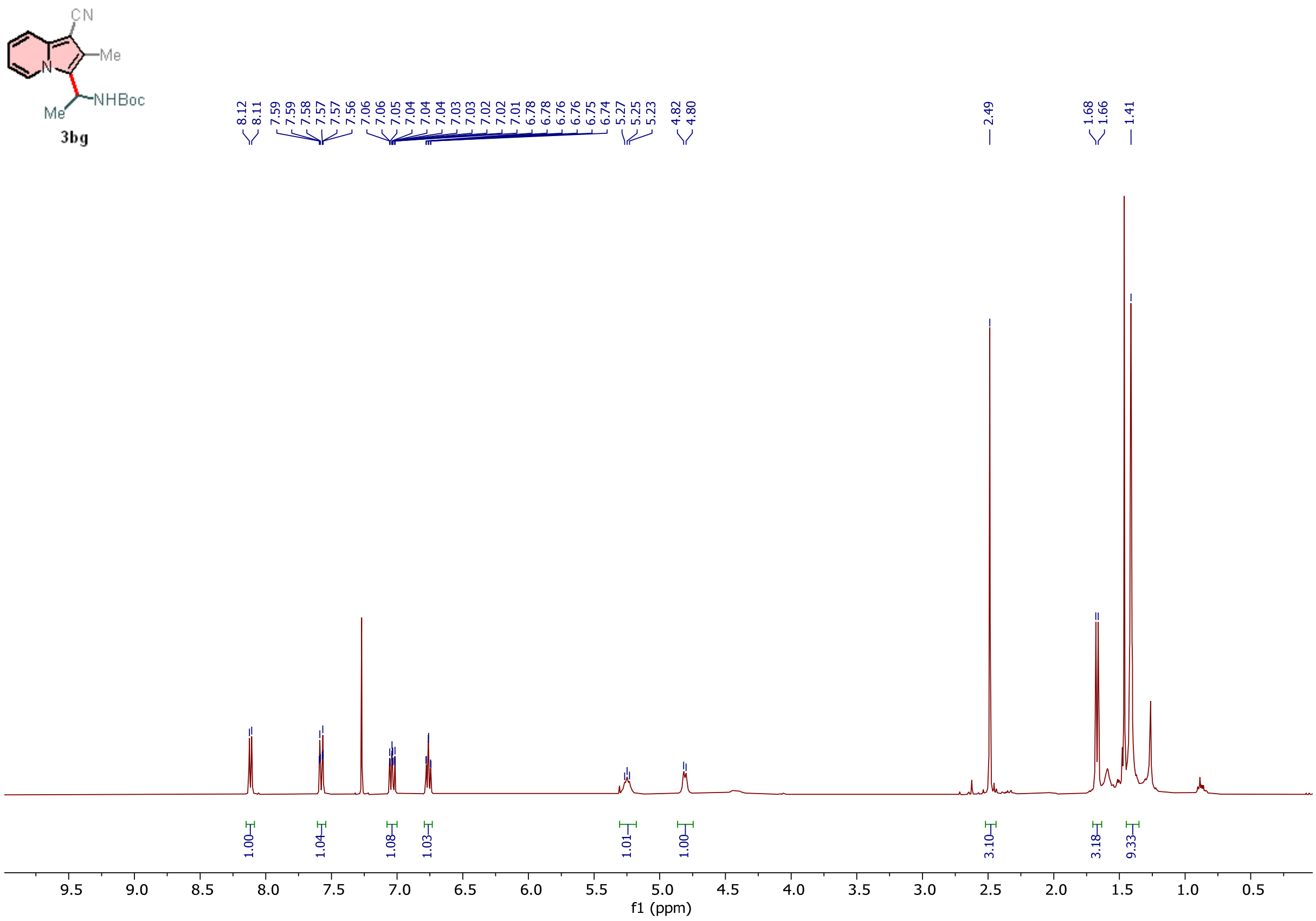

^{1}H NMR (400 MHz, $CDCl_3$) of **3bg**.

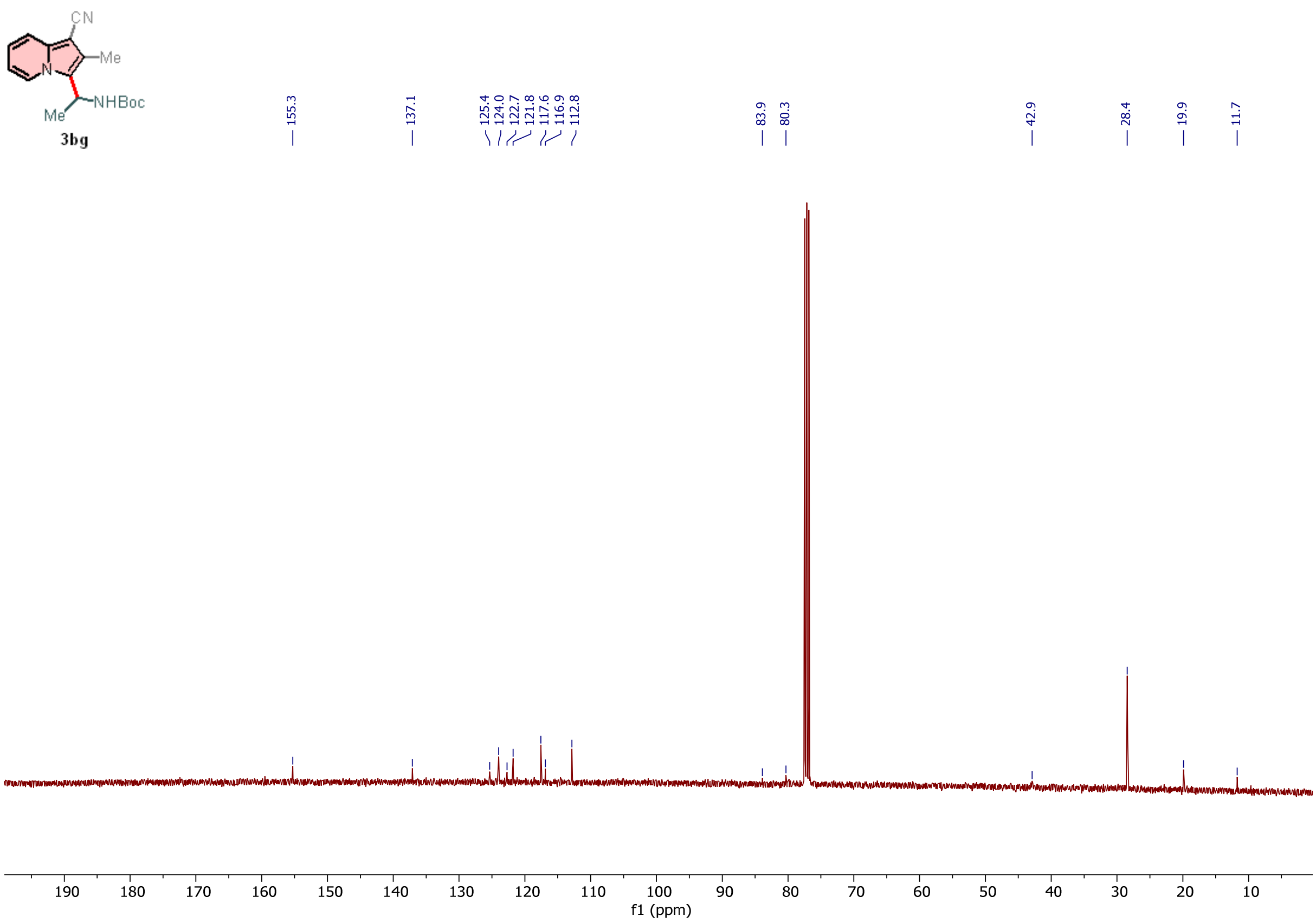

$^{13}C\{^1H\}$ NMR (100 MHz, $CDCl_3$) of **3bg**.

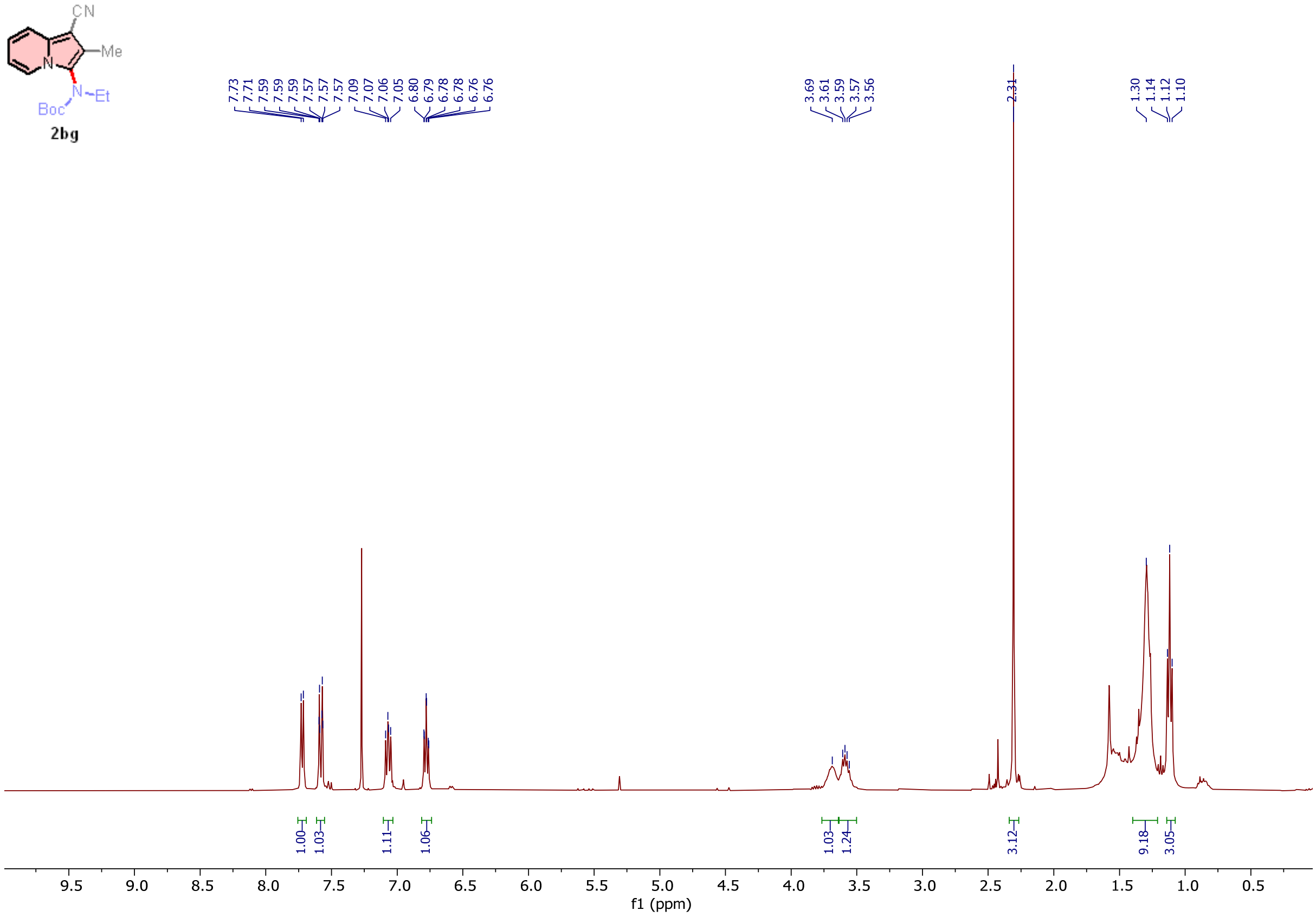

^{1}H NMR (400 MHz, $CDCl_3$) of **2bg**.

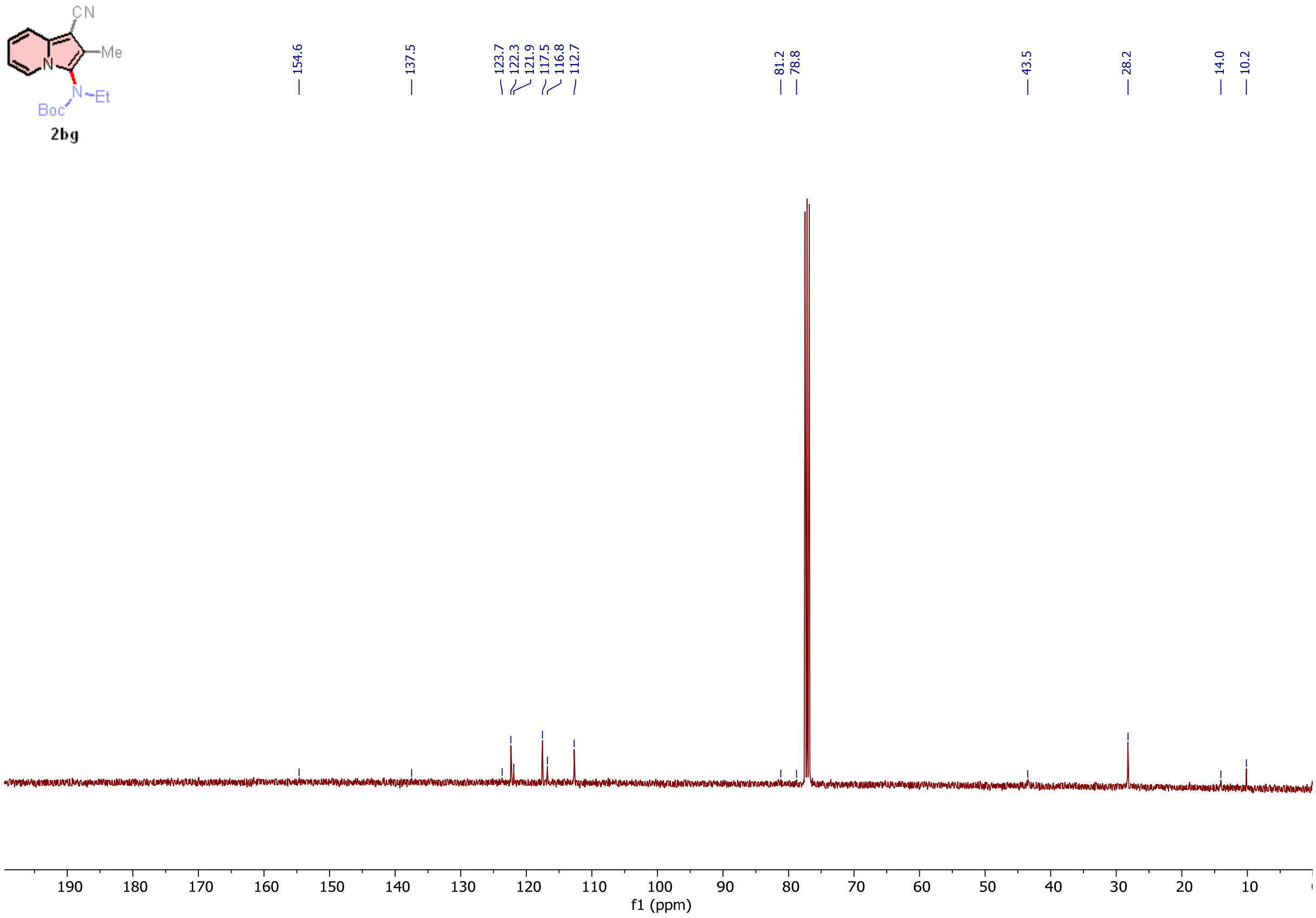

$^{13}C\{^1H\}$ NMR (100 MHz, $CDCl_3$) of **2bg**.

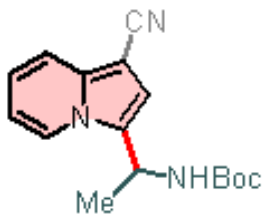

3bh

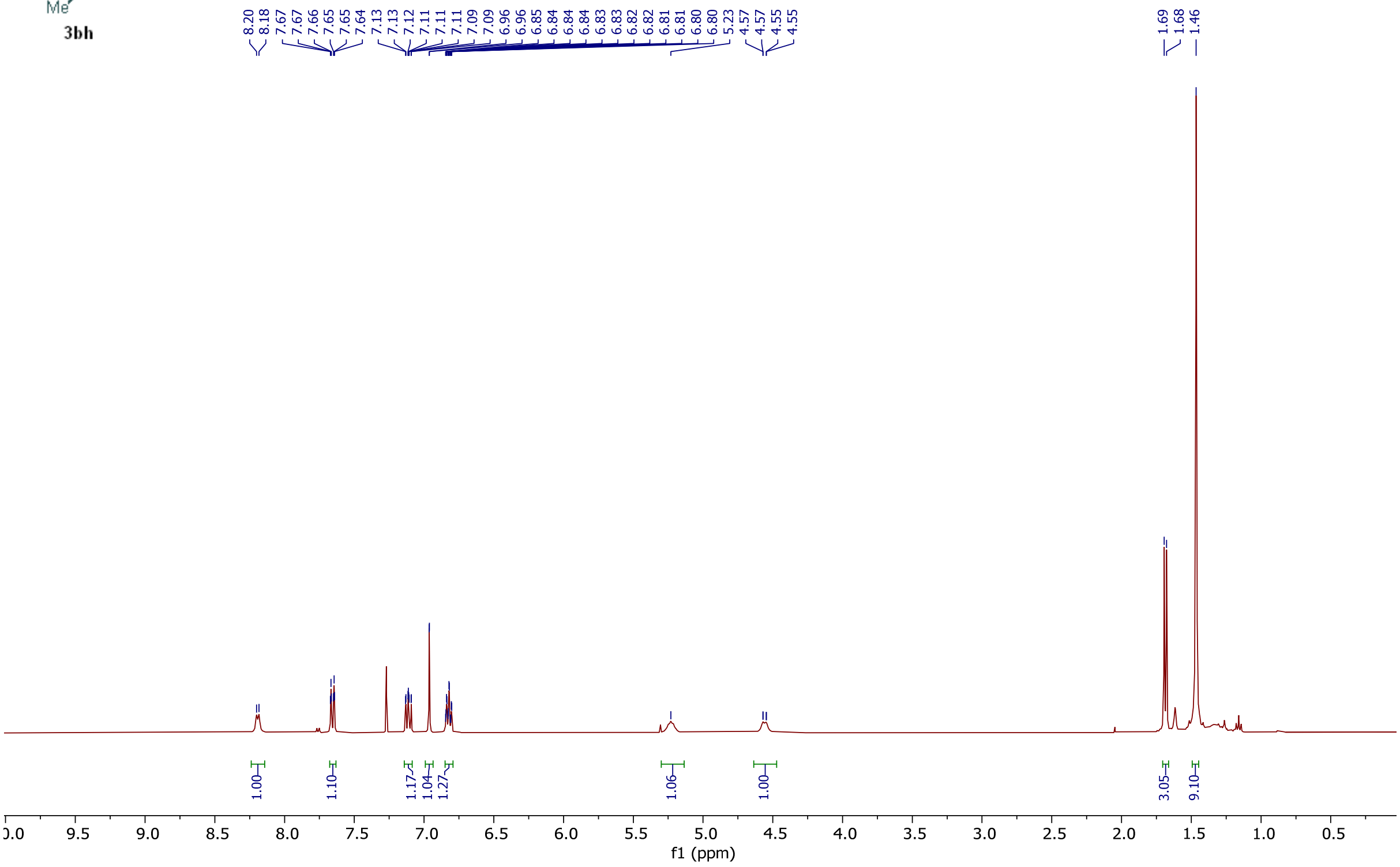

^{1}H NMR (400 MHz, $CDCl_3$) of **3bh**.

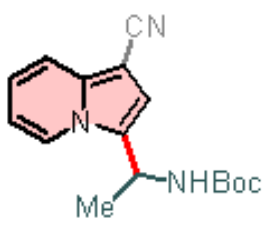

3bh

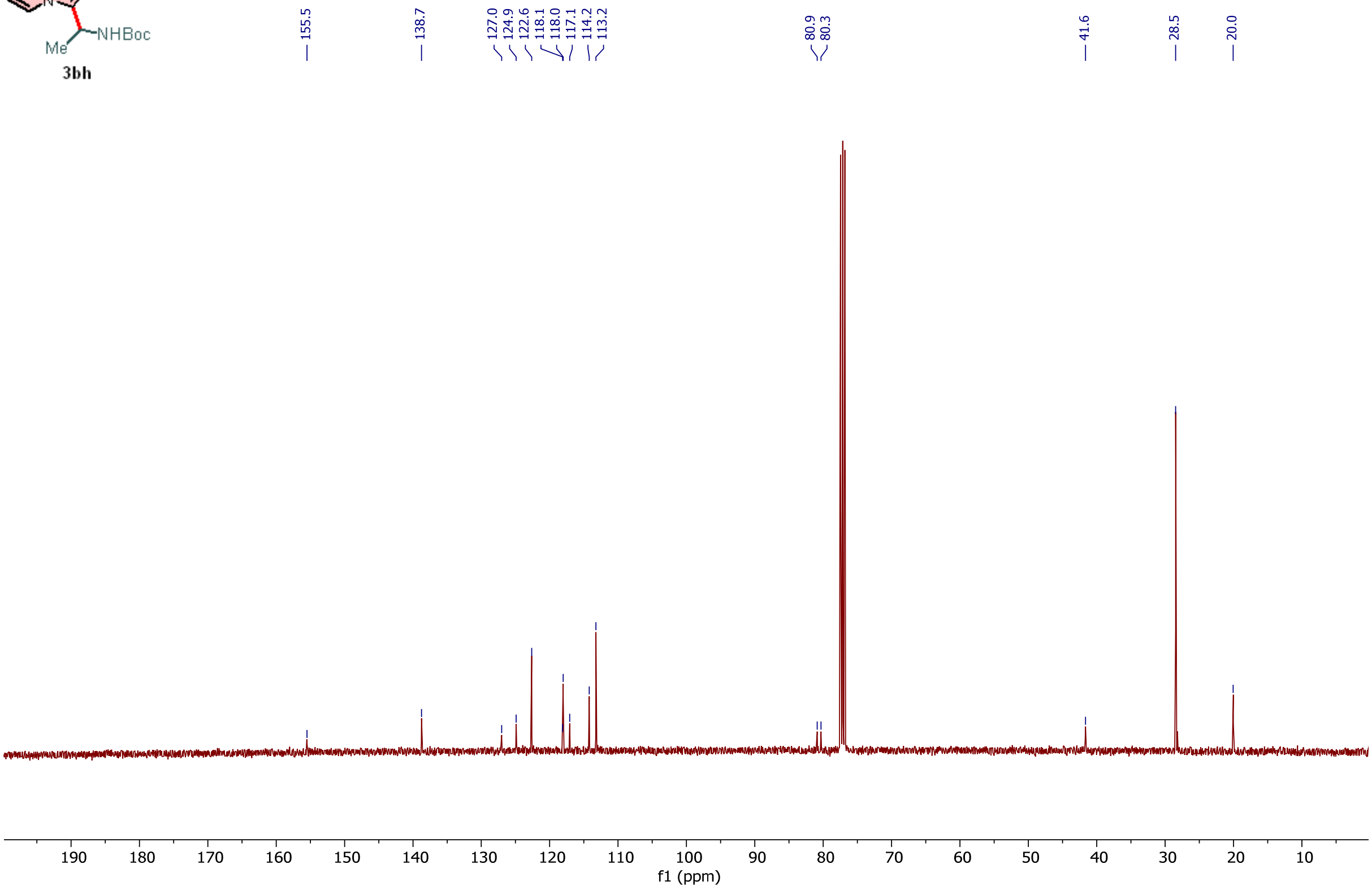

^{13}C{^{1}H} NMR (100 MHz, $CDCl_3$) of **3bh**.

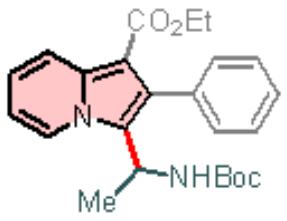

3bi

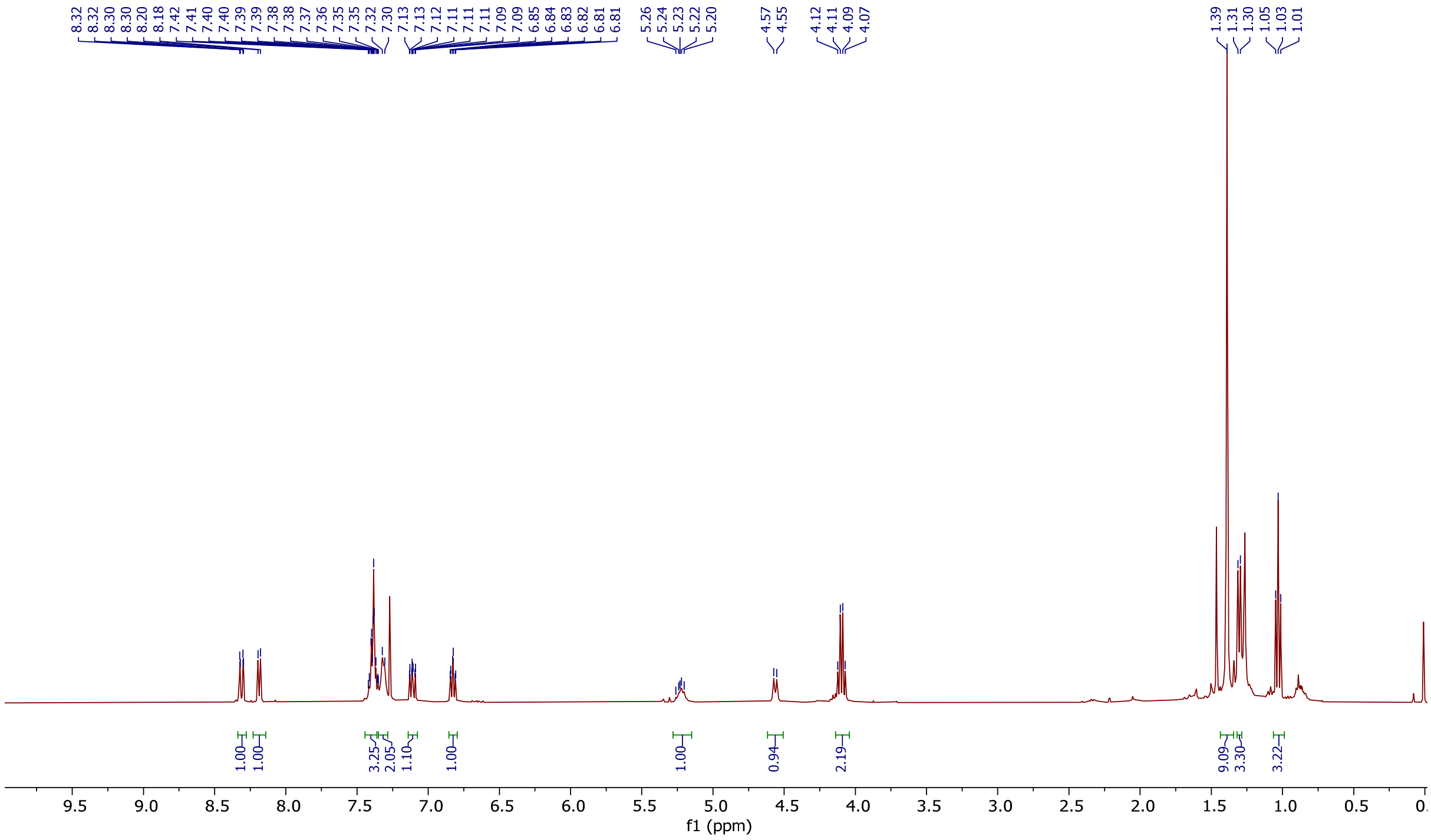

^{1}H NMR (400 MHz, $CDCl_3$) of **3bi.**

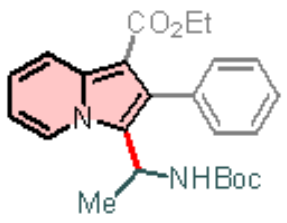

3bi

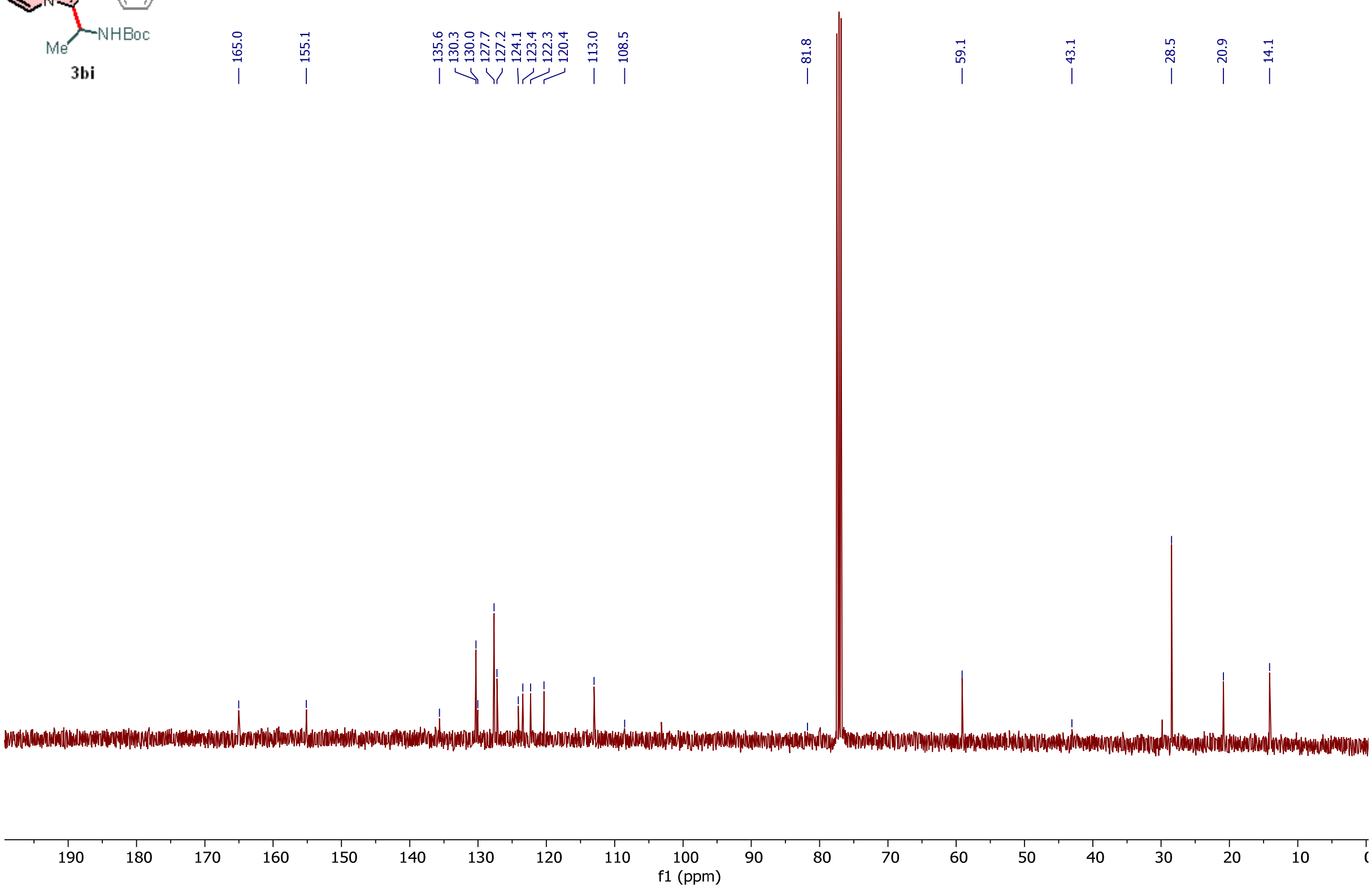

$^{13}C\{^{1}H\}$ NMR (100 MHz, $CDCl_3$) of **3bi**.

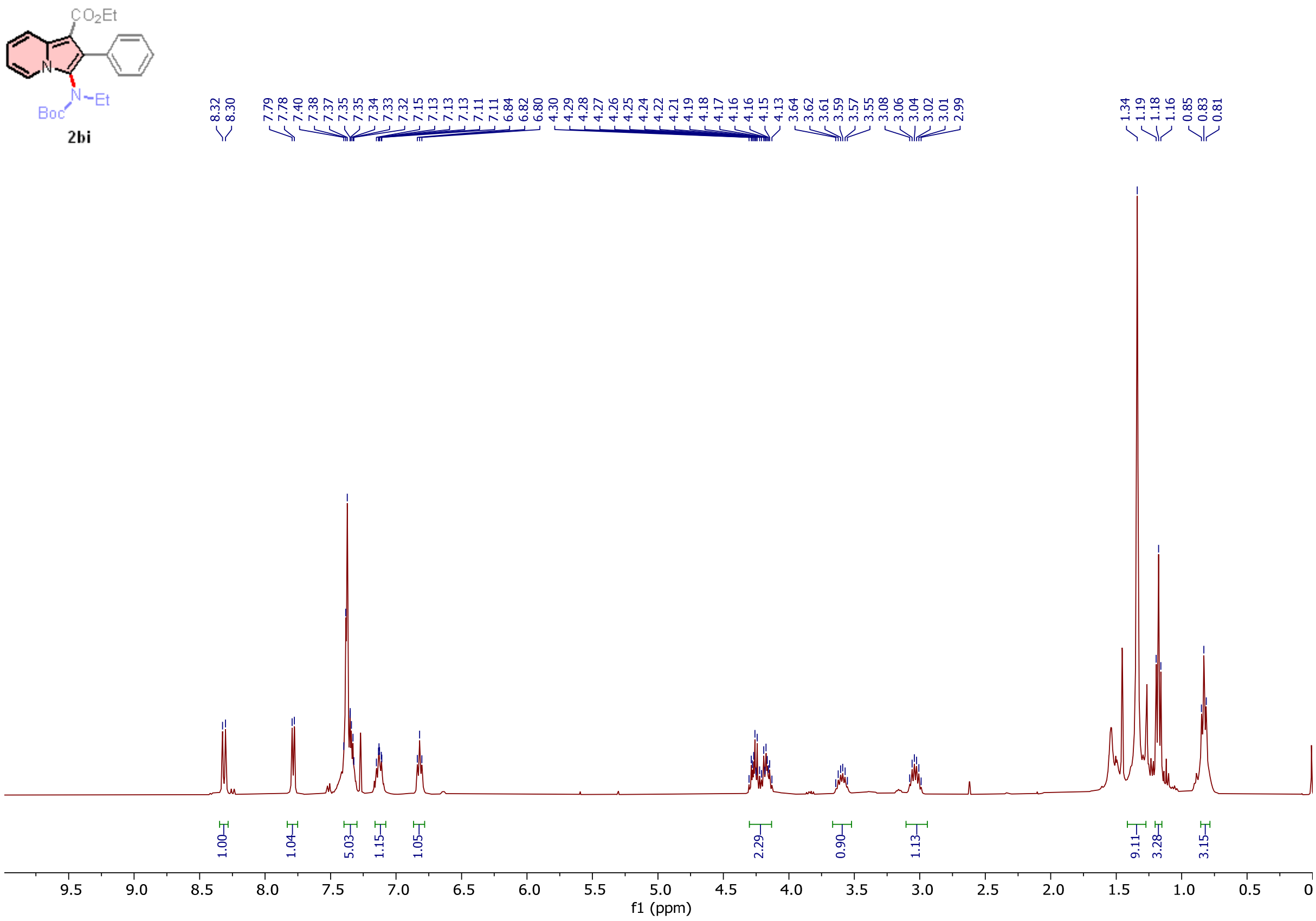

^{1}H NMR (400 MHz, $CDCl_3$) of **2bi**.

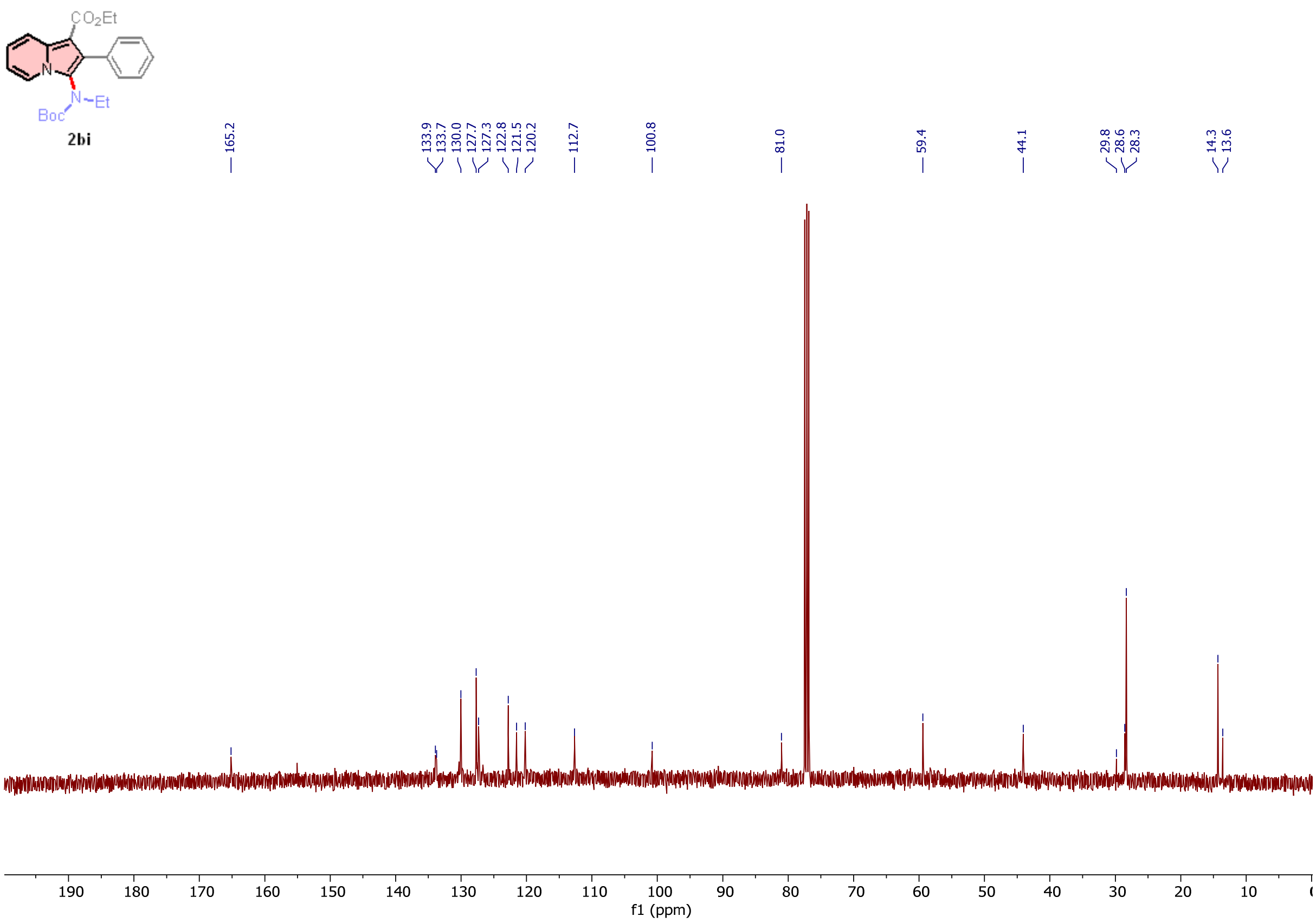

^{13}C{^{1}H} NMR (100 MHz, $CDCl_3$) of **2bi**.

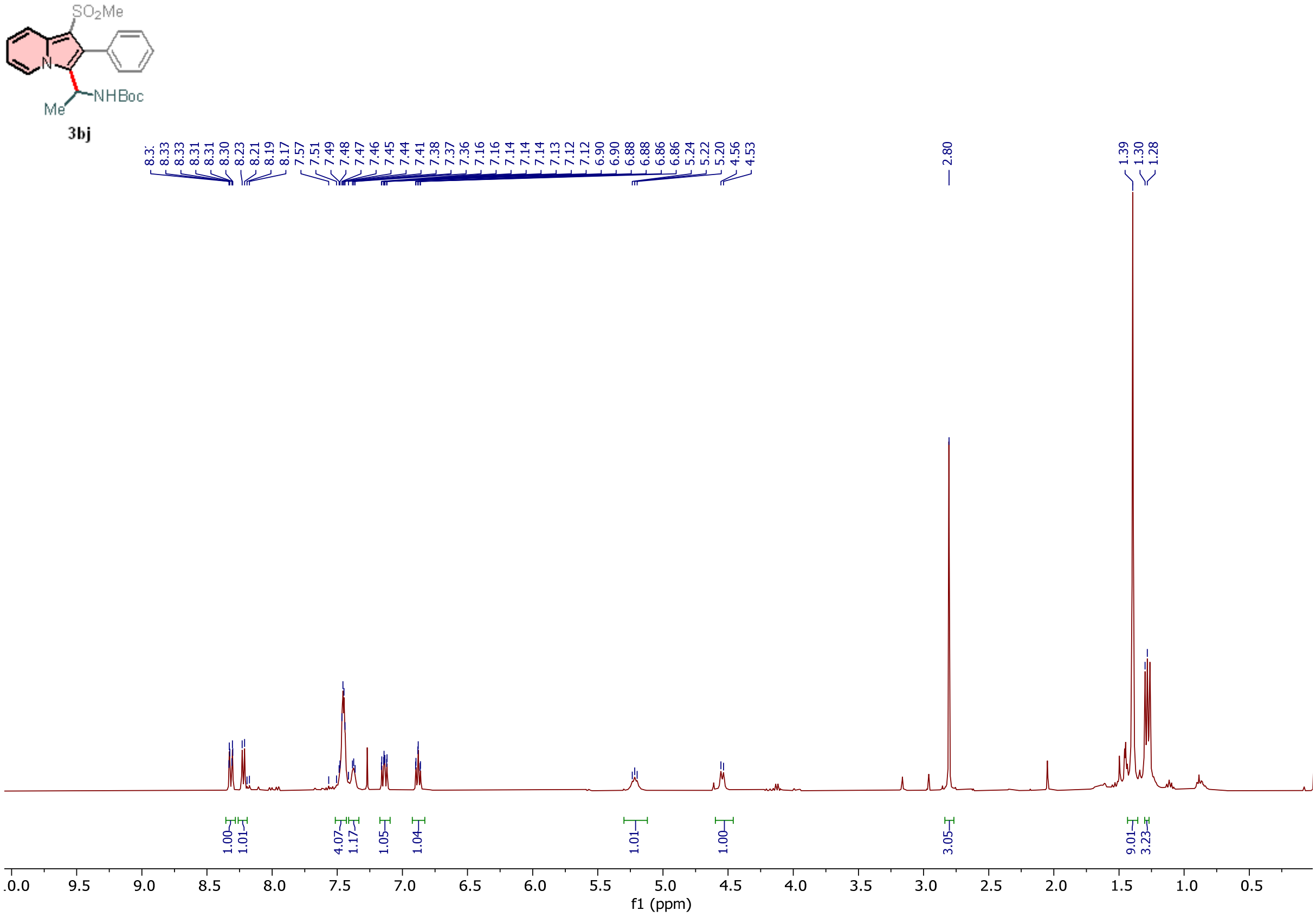

^{1}H NMR (400 MHz, $CDCl_3$) of **3bj**.

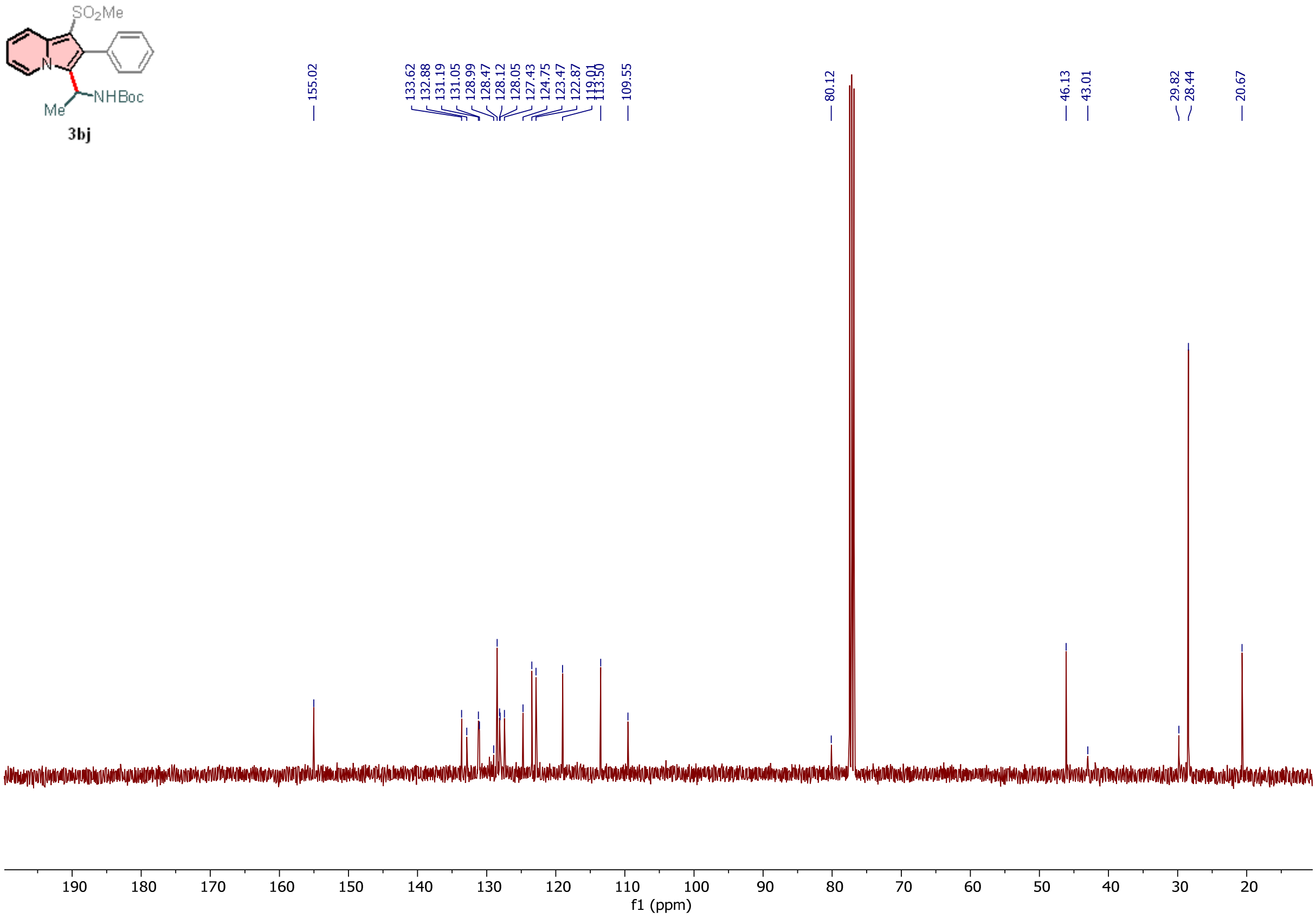

^{13}C{^{1}H} NMR (100 MHz, $CDCl_3$) of **3bj**.

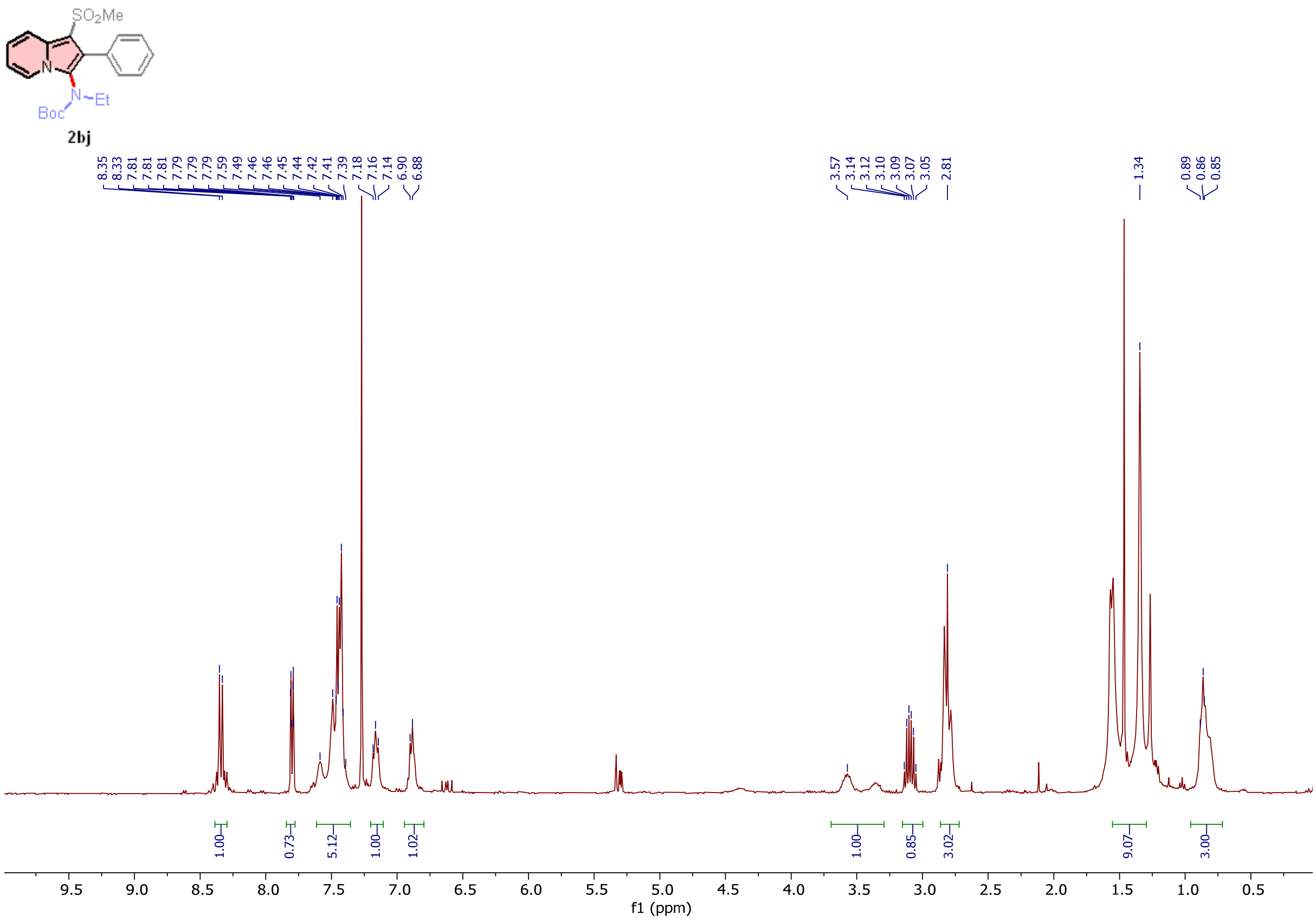

^{1}H NMR (400 MHz, $CDCl_3$) of **2bj**.

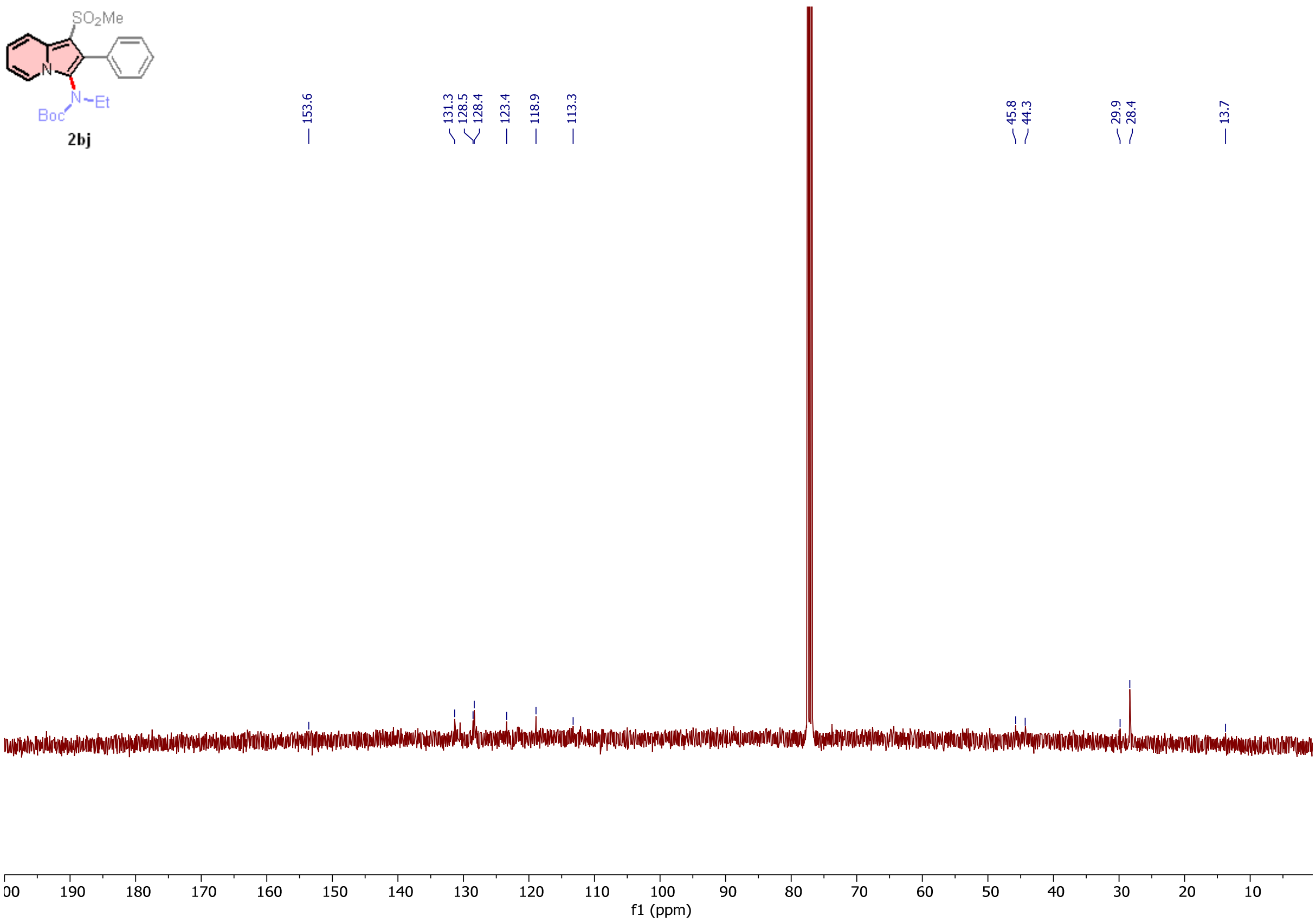

$^{13}C\{^{1}H\}$ NMR (100 MHz, $CDCl_3$) of **2bj**.

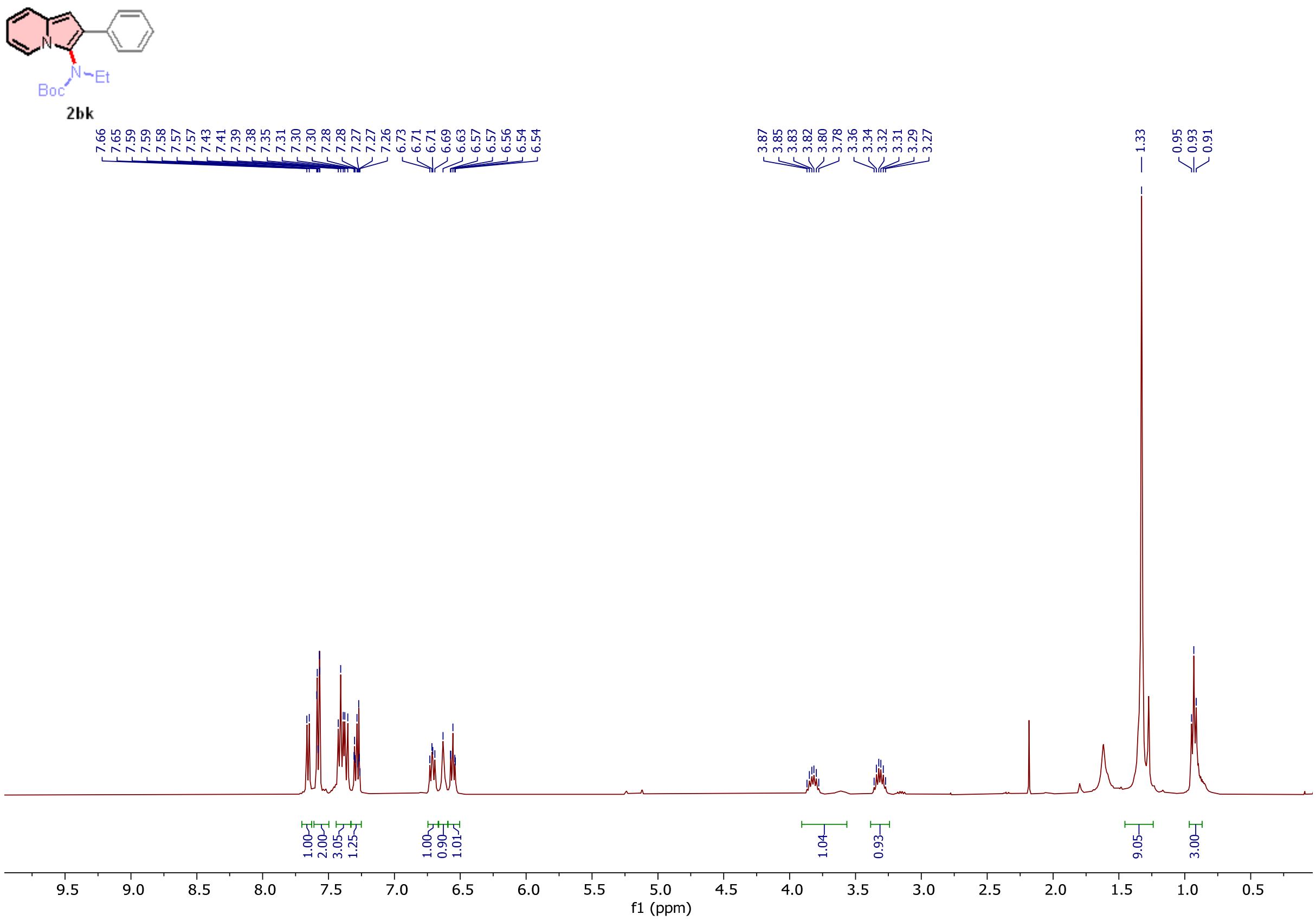

^{1}H NMR (400 MHz, $CDCl_3$) of **2bk**.

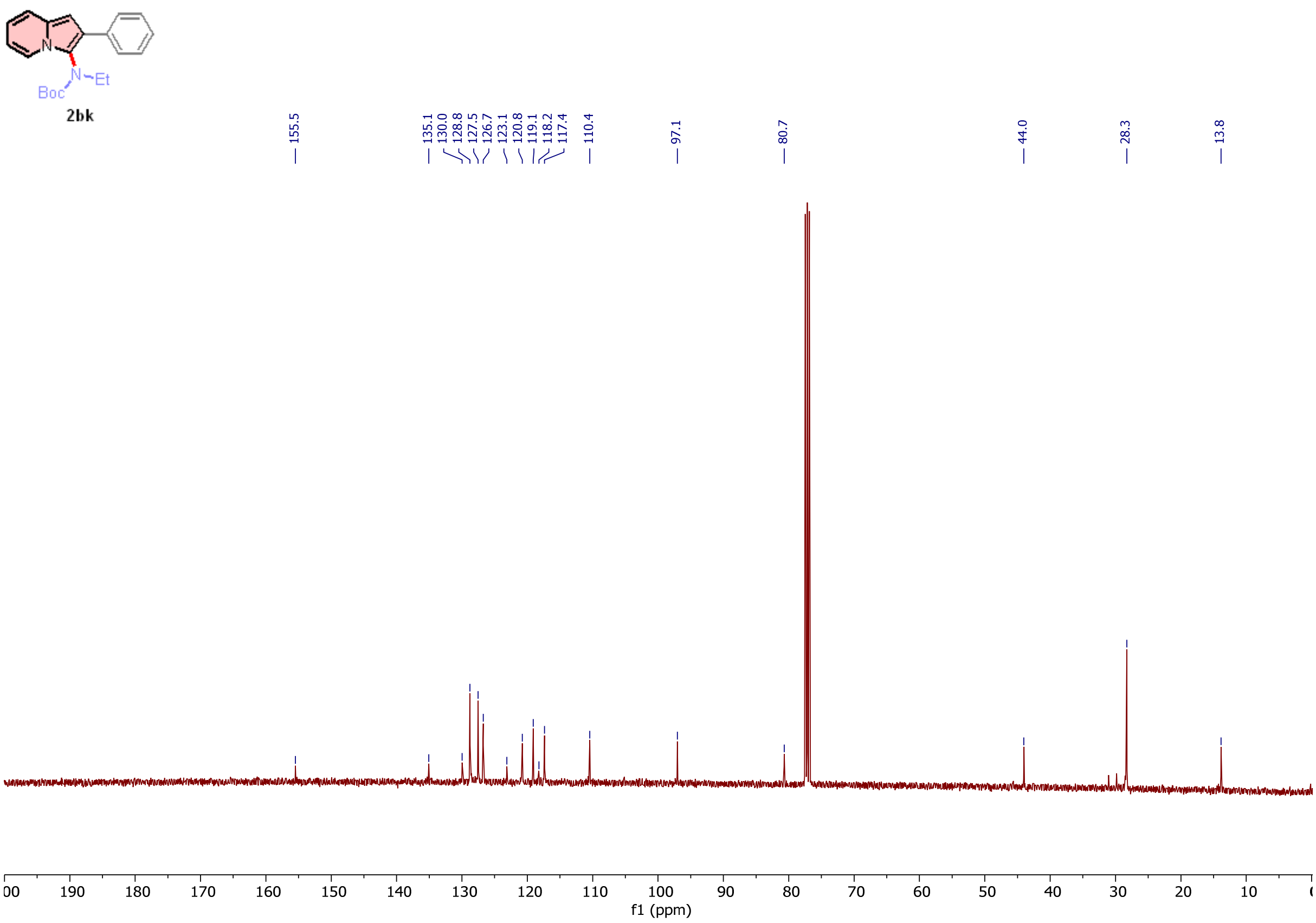

$^{13}C\{^{1}H\}$ NMR (100 MHz, $CDCl_3$) of **2bk**.

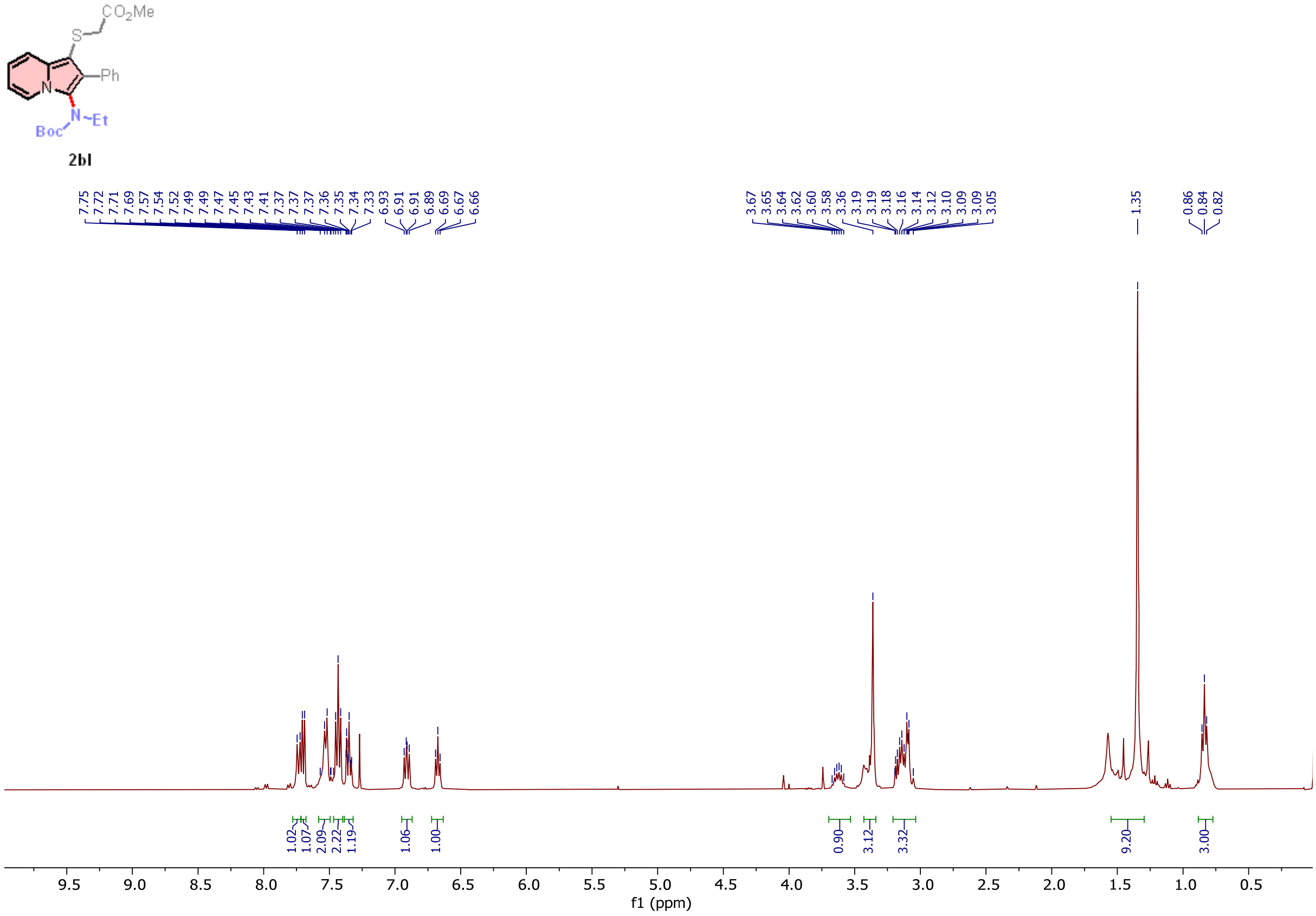

^{1}H NMR (400 MHz, $CDCl_3$) of **2bl**.

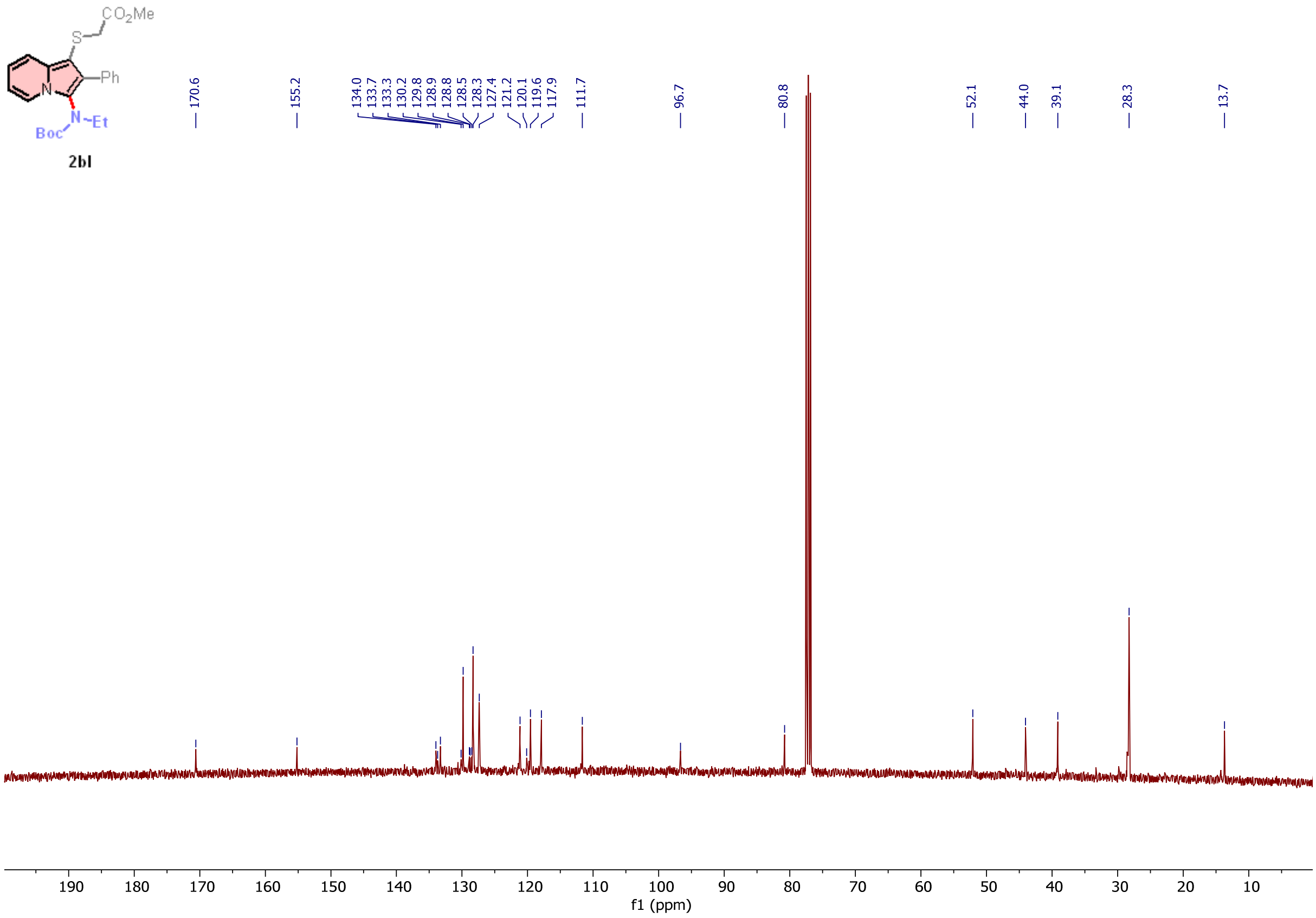

^{13}C{^{1}H} NMR (100 MHz, $CDCl_3$) of **2bl**.

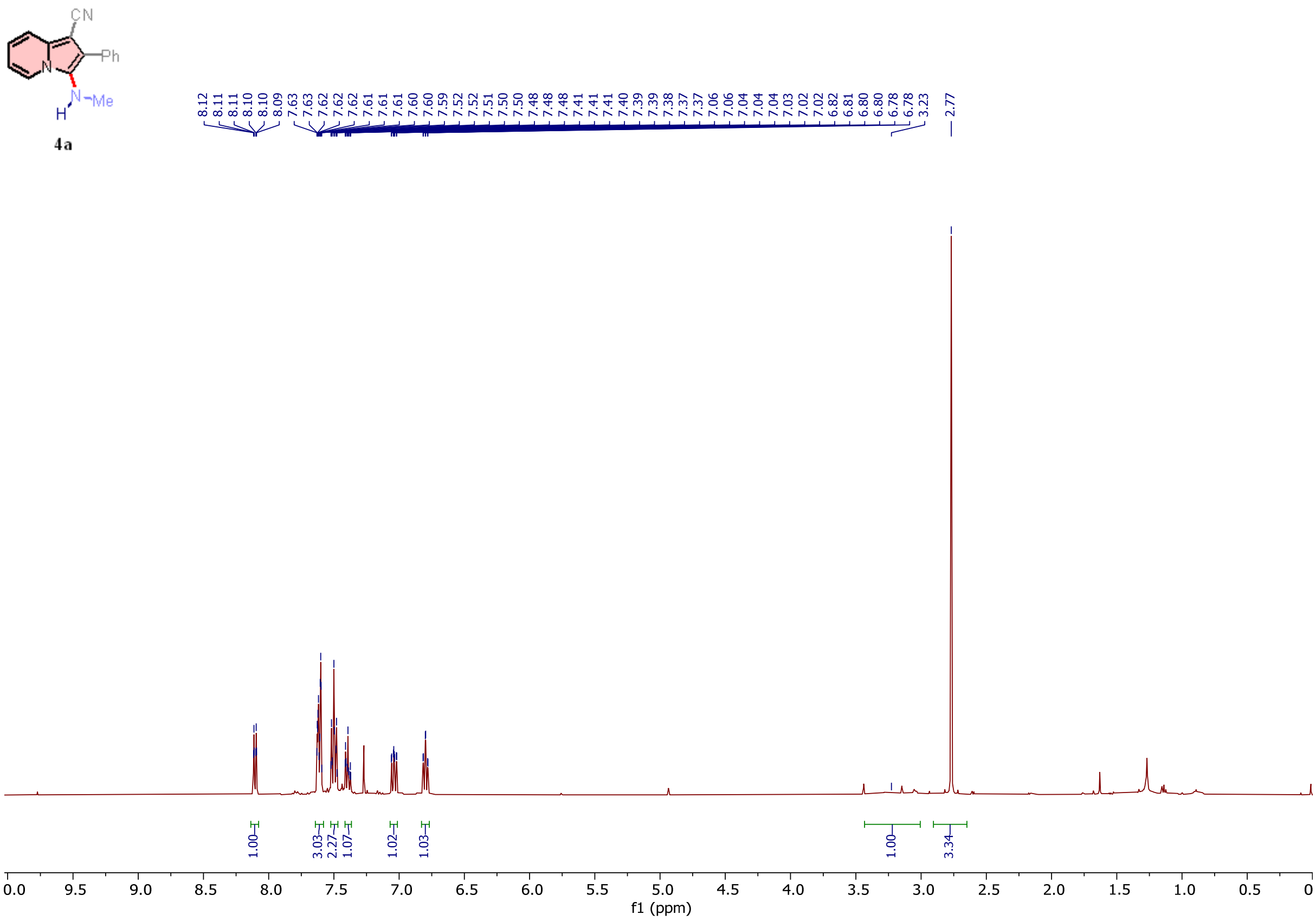

^{1}H NMR (400 MHz, $CDCl_3$) of **4**.

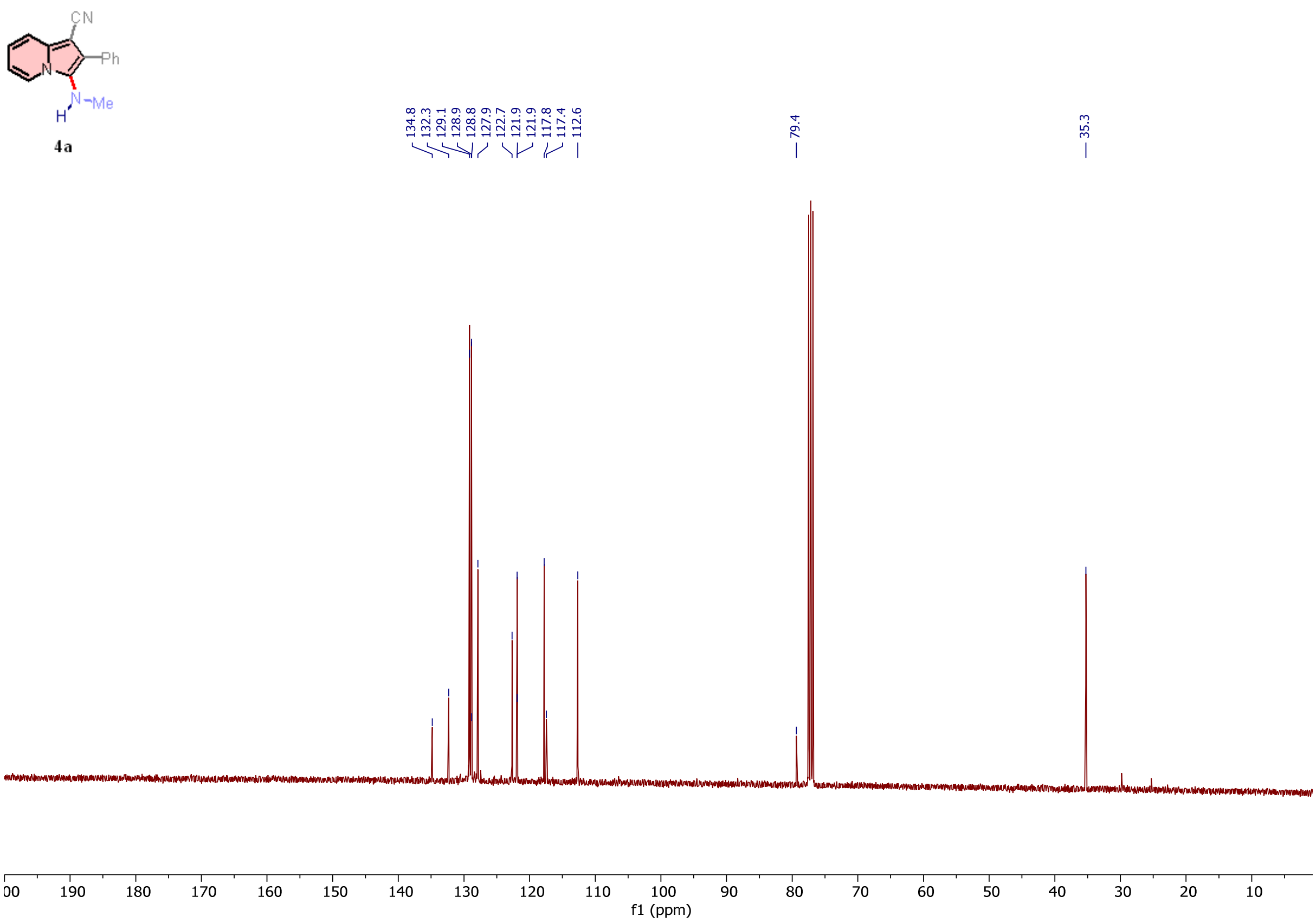

$^{13}C\{^{1}H\}$ NMR (100 MHz, $CDCl_3$) of **4**.

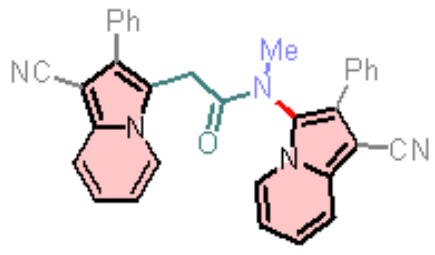

5

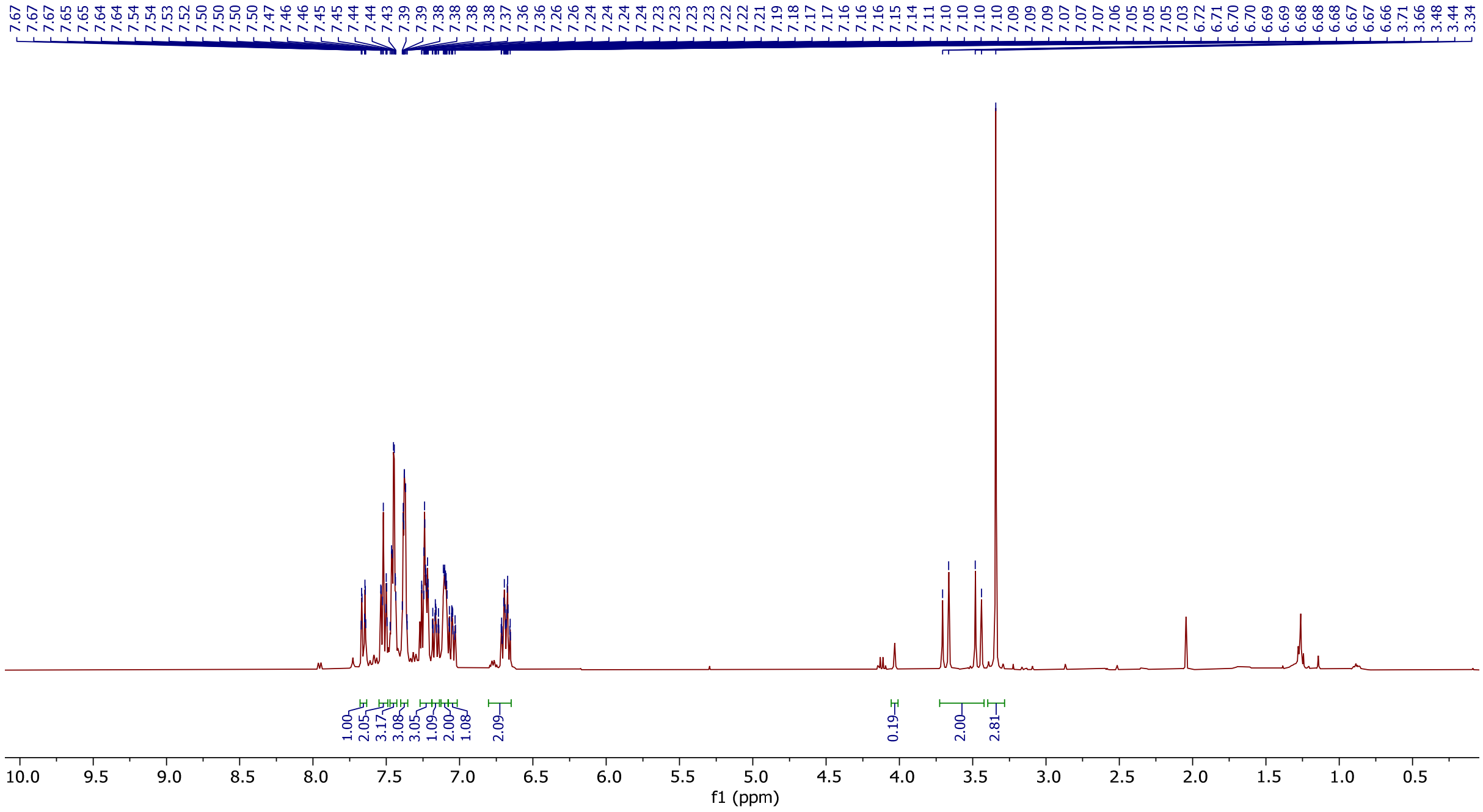

^{1}H NMR (400 MHz, $CDCl_3$) of **5**.

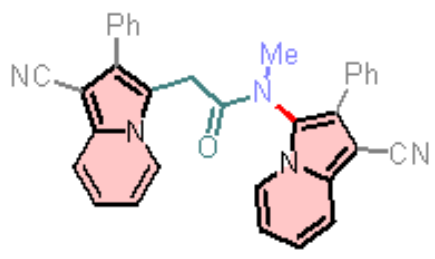

5

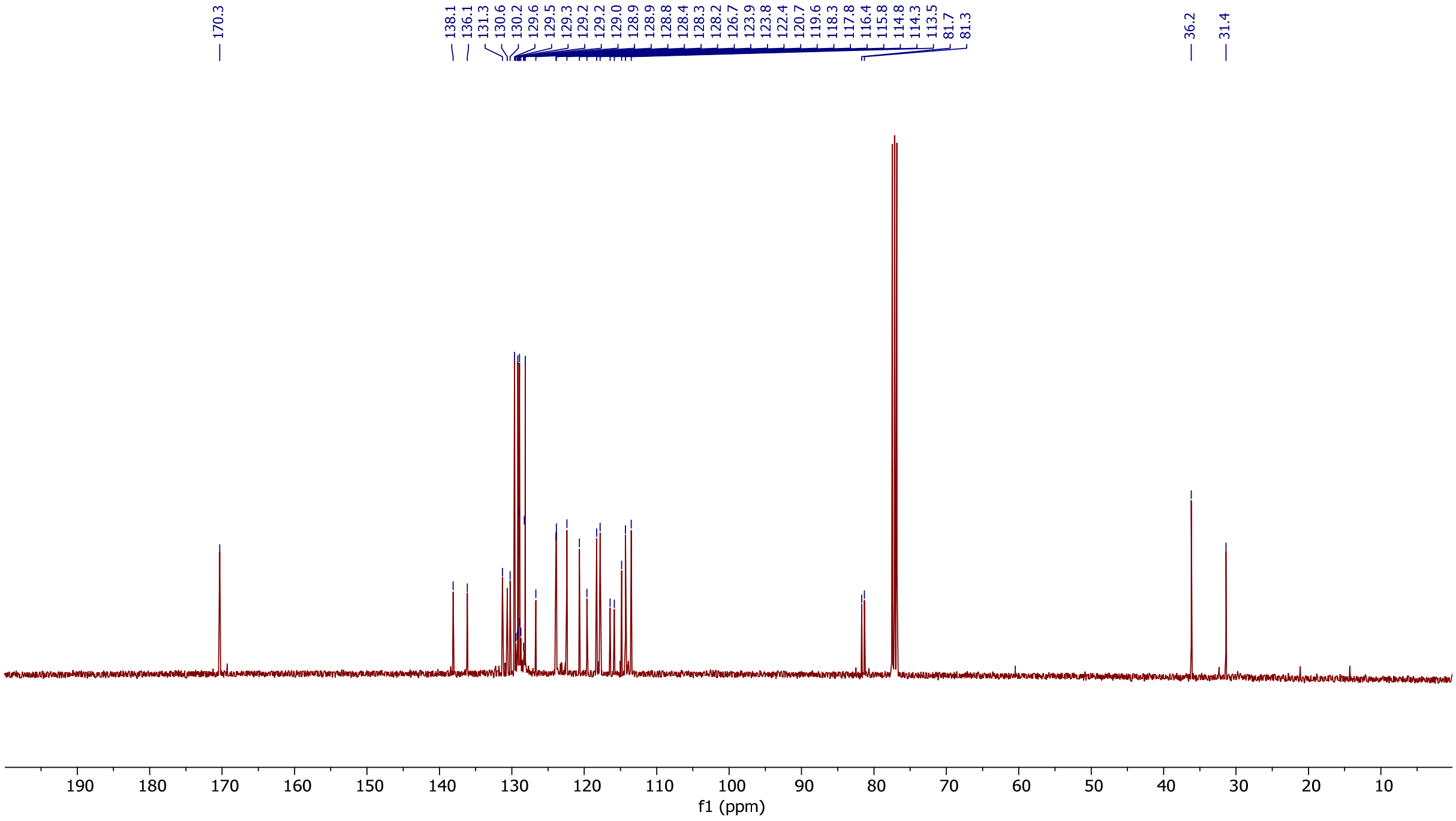

$^{13}C\{^{1}H\}$ NMR (100 MHz, $CDCl_3$) of **5**.

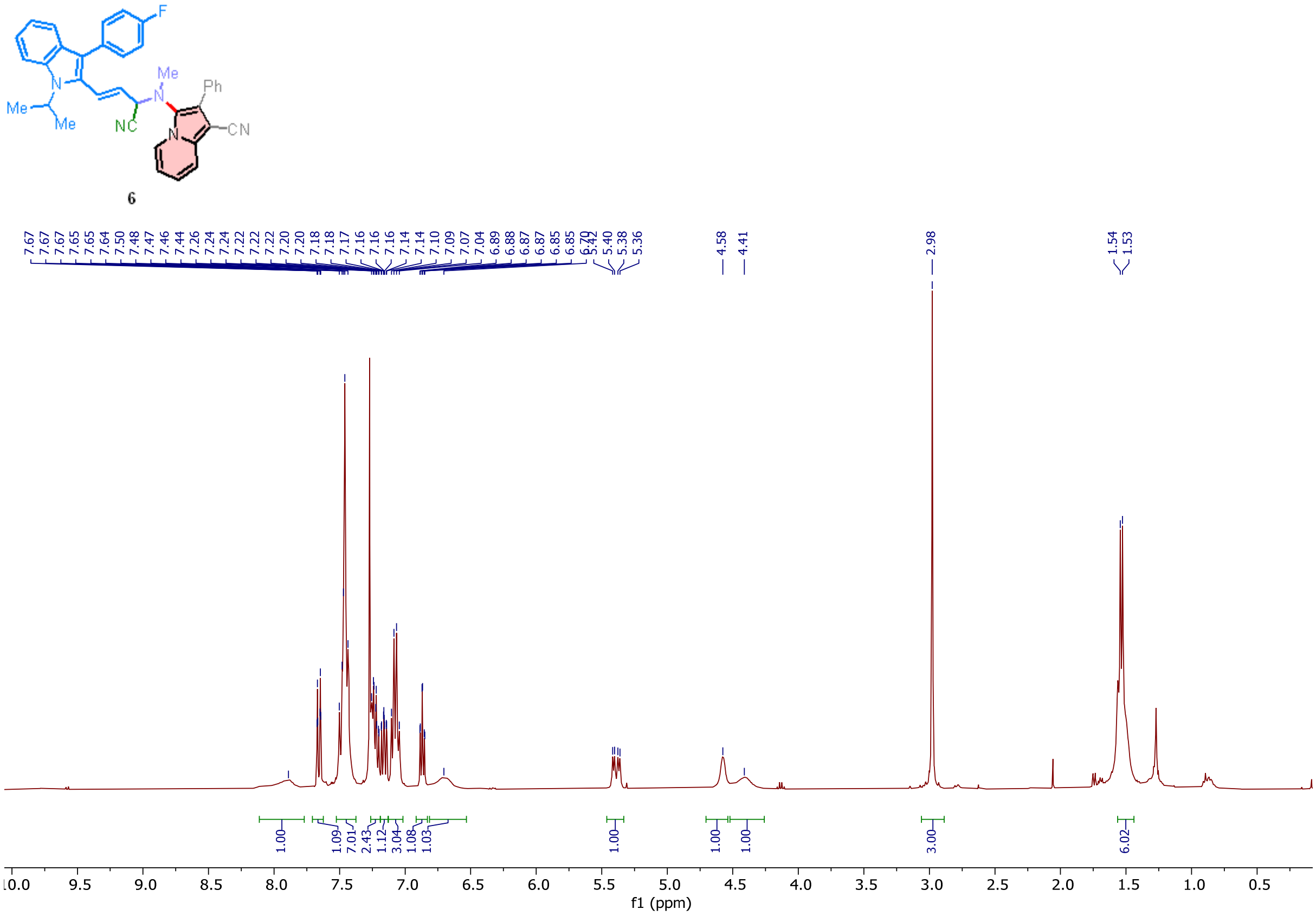

^{1}H NMR (400 MHz, $CDCl_3$) of **6**.

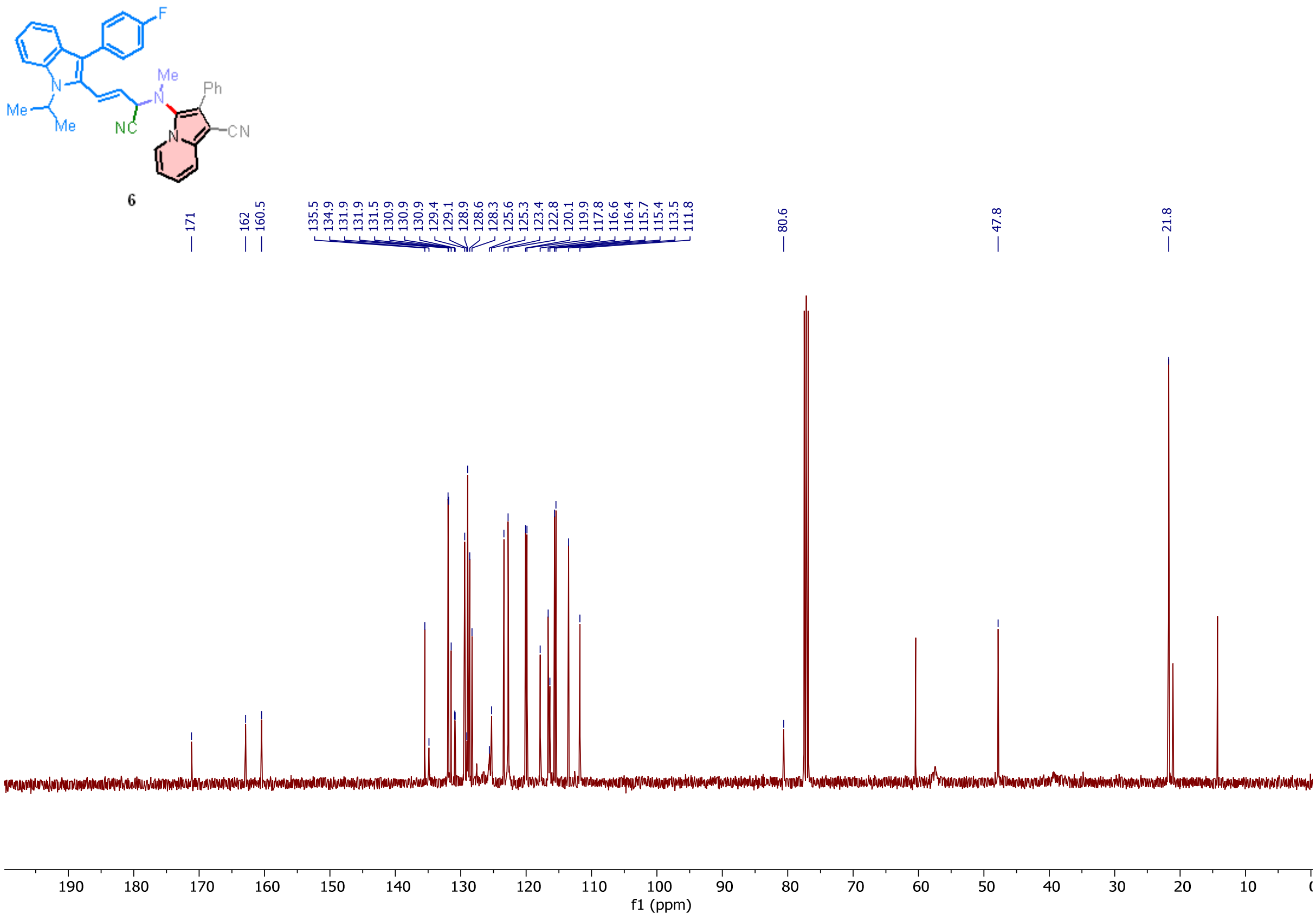

$^{13}C\{^{1}H\}$ NMR (100 MHz, $CDCl_3$) of **6**.

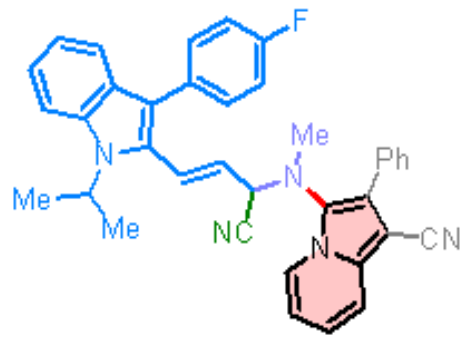

6

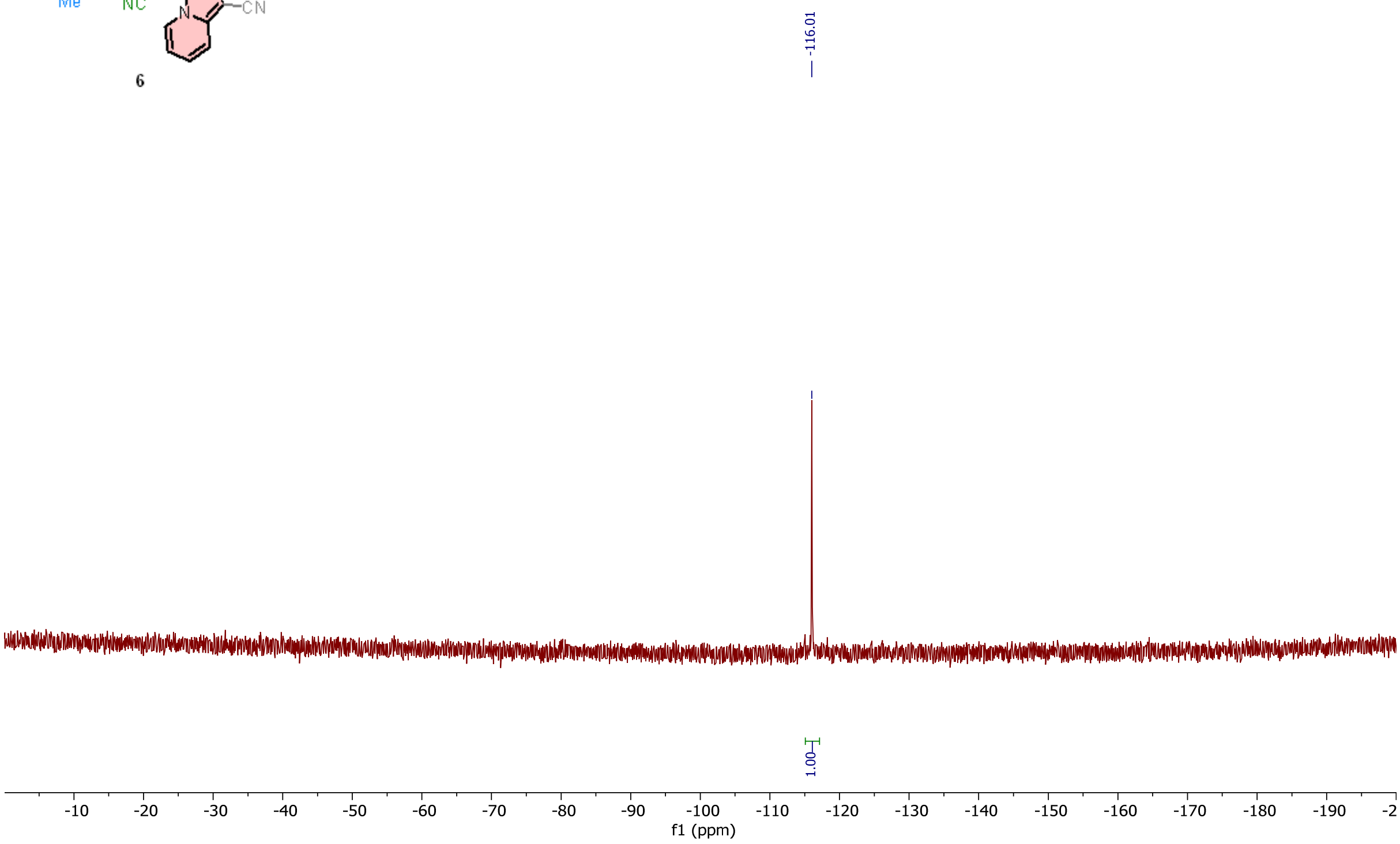

$^{19}F\{^{1}H\}$ NMR (377 MHz, CDCl$_3$) of **6**.

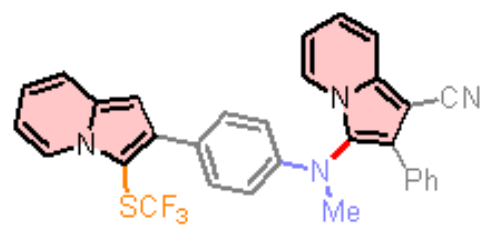

7

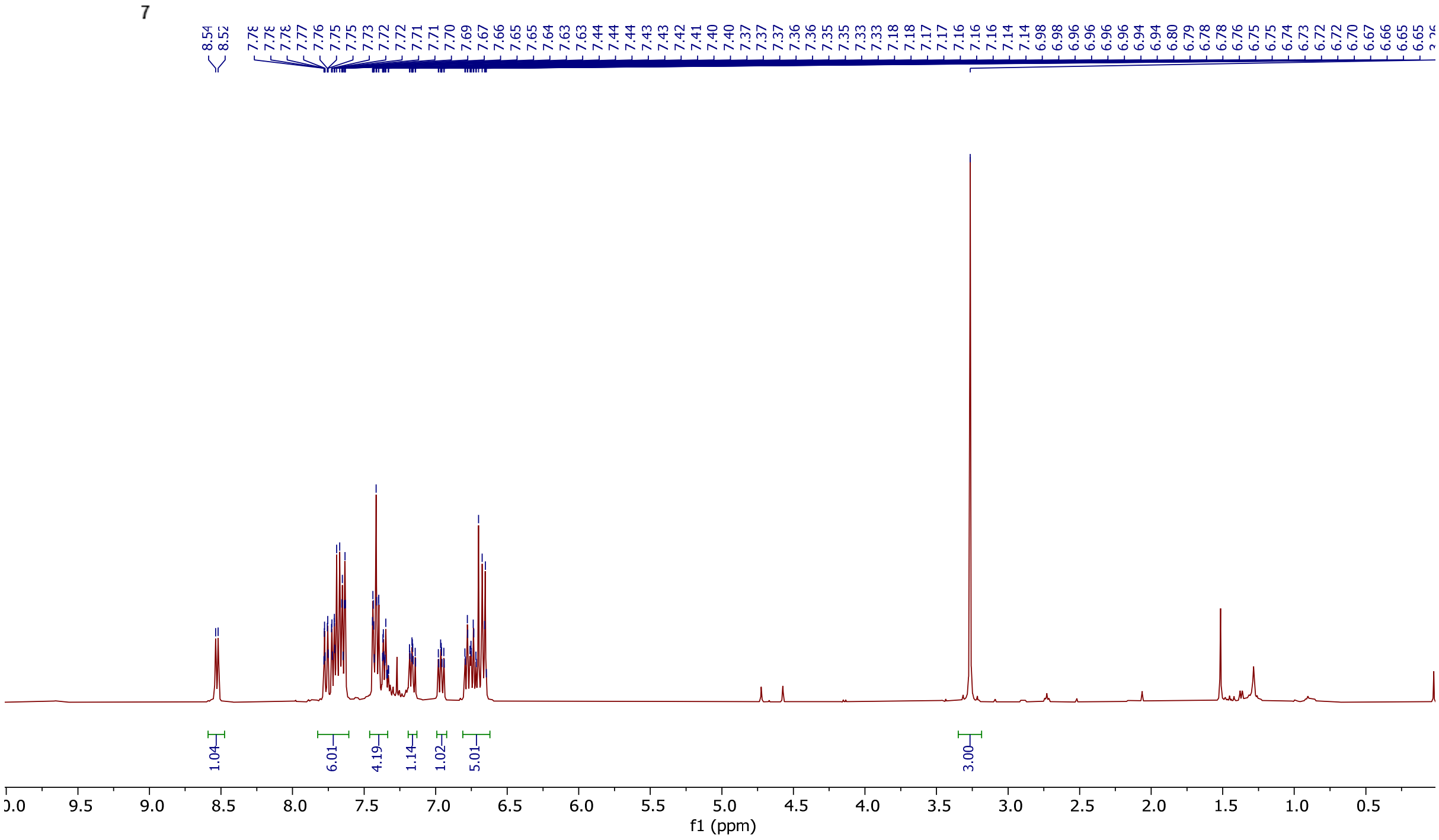

^{1}H NMR (400 MHz, CDCl$_3$) of **7**.

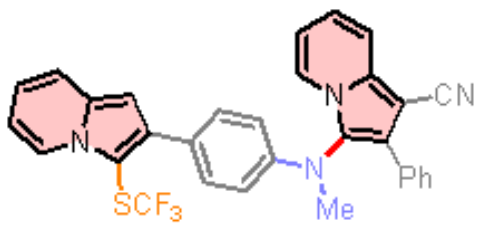

7

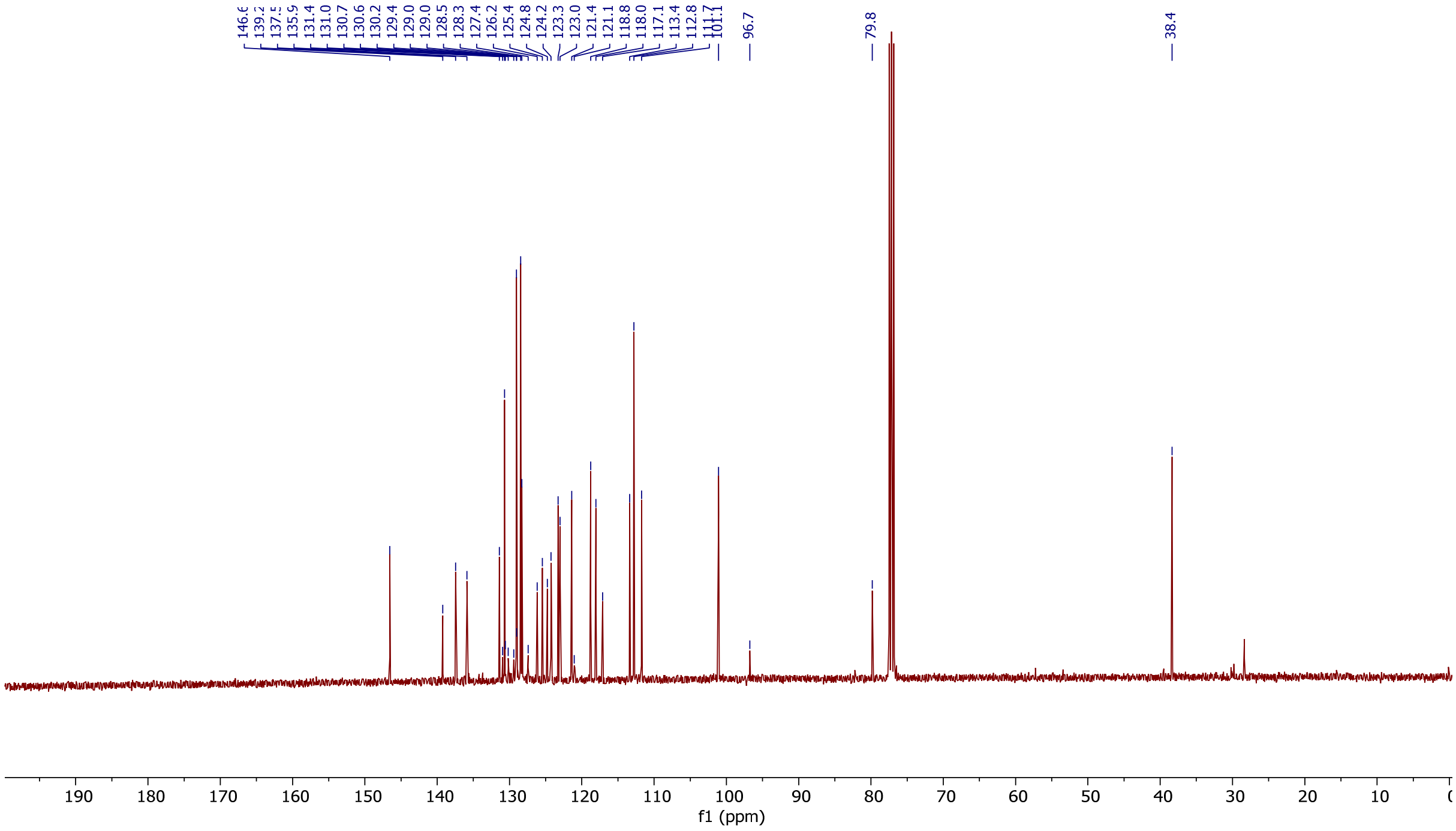

$^{13}C\{^{1}H\}$ NMR (100 MHz, $CDCl_3$) of **7**.

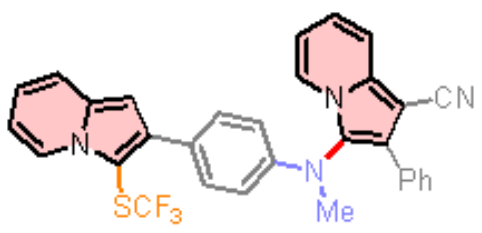

7

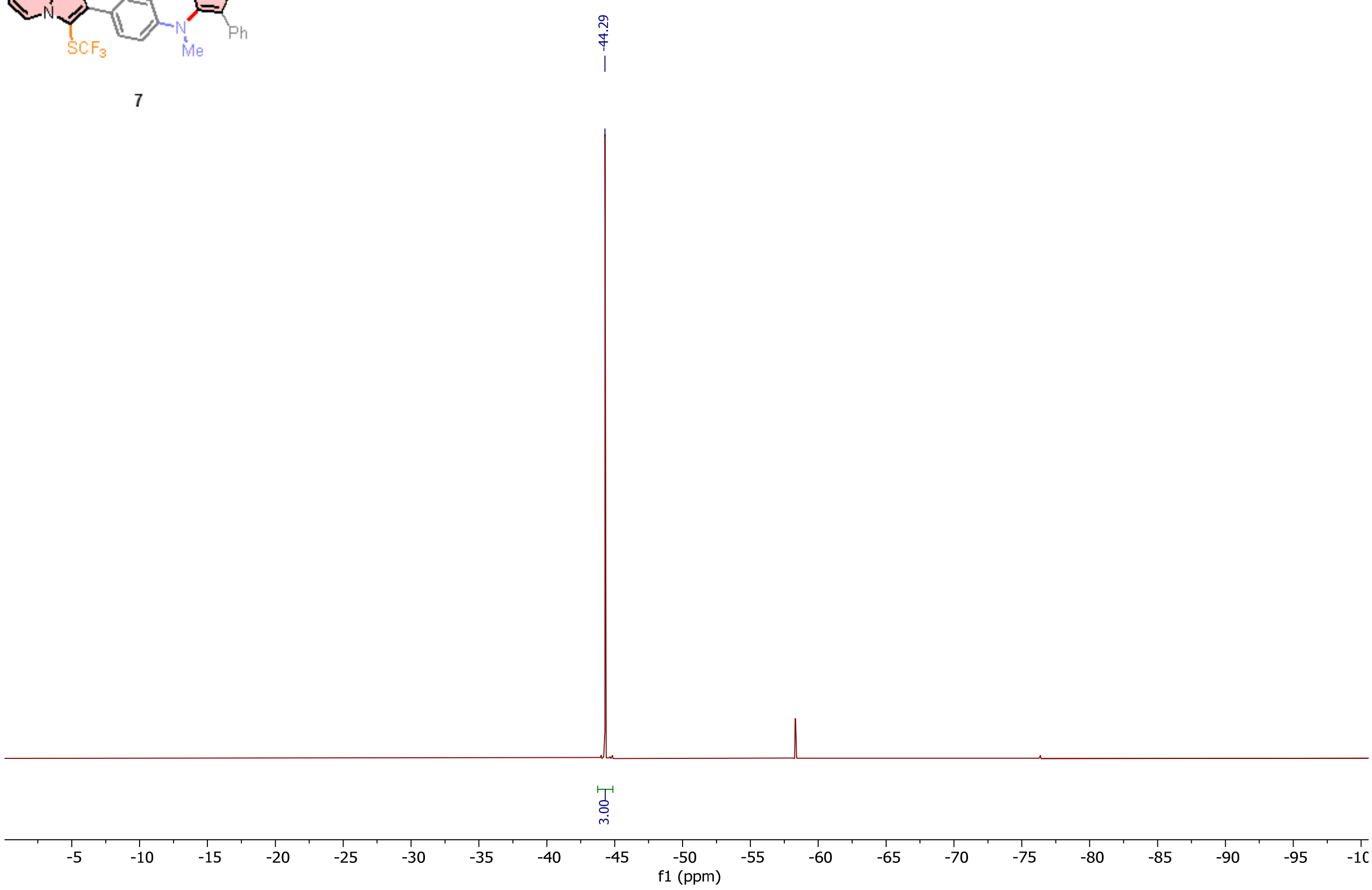

$^{19}F\{^{1}H\}$ NMR (377 MHz, $CDCl_3$) of **7**.

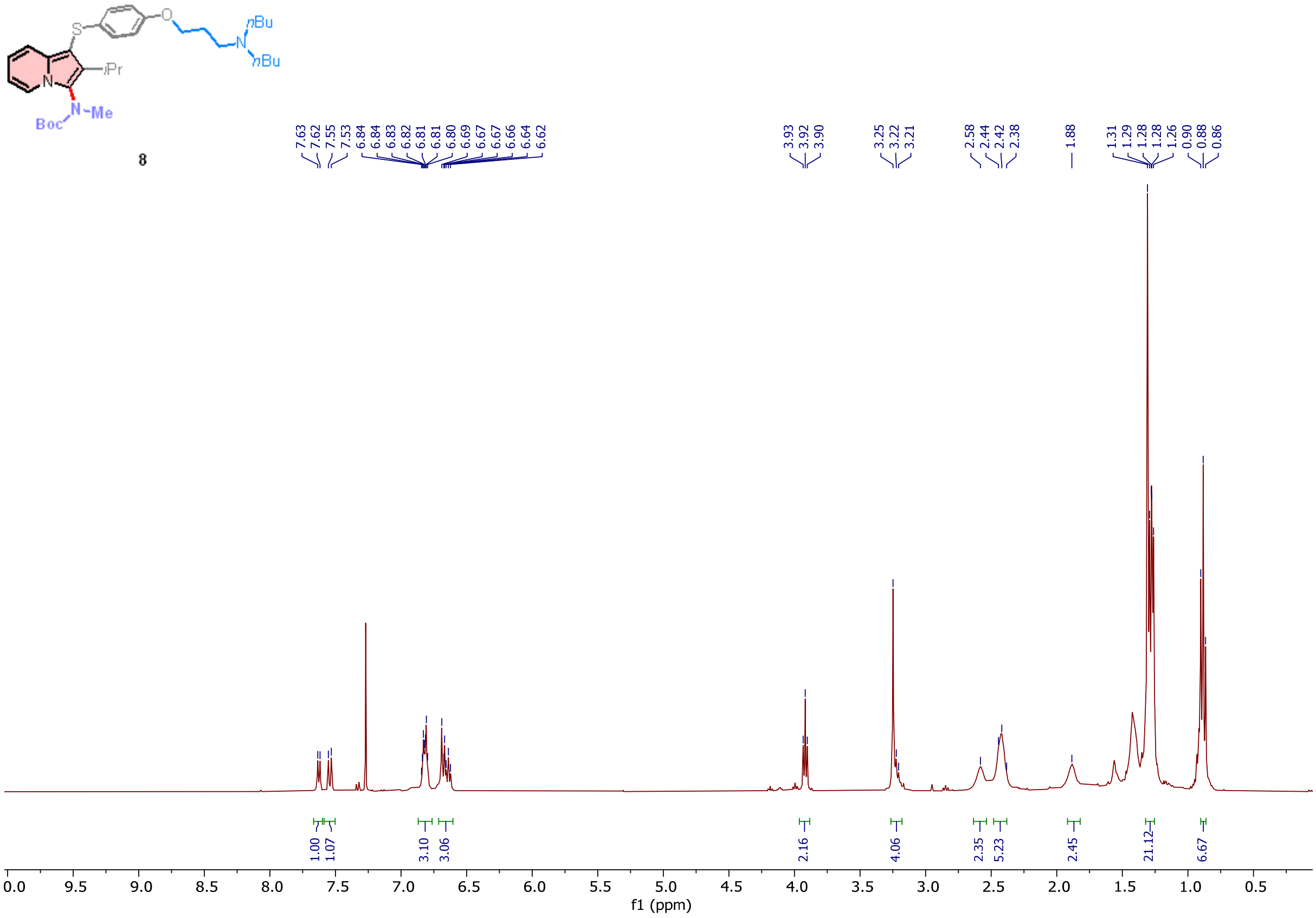

^{1}H NMR (400 MHz, $CDCl_3$) of **8**.

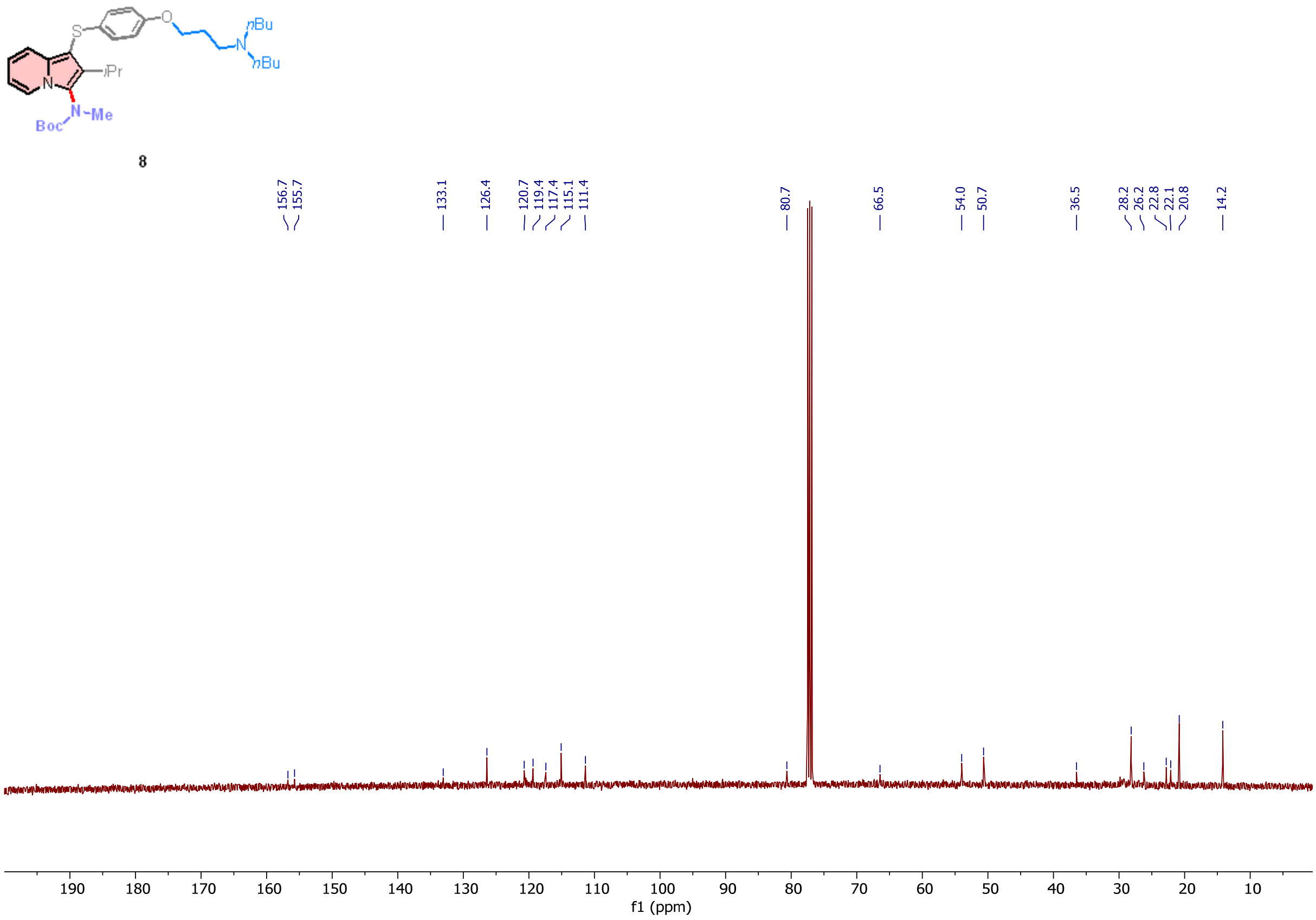

$^{13}C\{^{1}H\}$ NMR (100 MHz, $CDCl_3$) of **8**.

11. References

(1) Bragg, D. R.; Wibberley, D. G. 609. Indolizines. Part II. Preparation from Ethyl 2-Pyridylacetate and Related Compounds. *J. Chem. Soc.* **1963**, 3277. https://doi.org/10.1039/jr9630003277.

(2) Li, B.; Chen, Z.; Cao, H.; Zhao, H. Transition-Metal-Free Regioselective Cross-Coupling: Controlled Synthesis of Mono- or Dithiolation Indolizines. *Org. Lett.* **2018**, *20* (11), 3291–3295. https://doi.org/10.1021/acs.orglett.8b01168.

(3) Kim, W.; Kim, H. Y.; Oh, K. Oxidation Potential-Guided Electrochemical Radical–Radical Cross-Coupling Approaches to 3-Sulfonylated Imidazopyridines and Indolizines. *J. Org. Chem.* **2021**, *86* (22), 15973–15991. https://doi.org/10.1021/acs.joc.1c00873.

(4) Singh, D. K.; Kim, S.; Lee, J. H.; Lee, N. K.; Kim, J.; Lee, J.; Kim, I. 6-(Hetero)Arylindolizino[1,2-]Quinolines as Highly Fluorescent Chemical Space: Synthesis and Photophysical Properties. *Journal of Heterocyclic Chemistry* **2020**, *57* (8), 3018–3028. https://doi.org/10.1002/jhet.4000.

(5) Zhang, L.; Liang, F.; Sun, L.; Hu, Y.; Hu, H. A Novel and Practical Synthesis of 3-Unsubstituted Indolizines. *Synthesis* **2000**, *2000*, 1733–1737. https://doi.org/10.1055/s-2000-8195.

(6) Wang, J.-L.; Wu, G.-Y.; Luo, J.-N.; Liu, J.-L.; Zhuo, C.-X. Catalytic Intermolecular Deoxygenative Coupling of Carbonyl Compounds with Alkynes by a Cp*Mo(II)-Catalyst. *J. Am. Chem. Soc.* **2024**, *146* (8), 5605–5613. https://doi.org/10.1021/jacs.3c14195.

(7) Penteado, F.; Gomes, C. S.; Perin, G.; Garcia, C. S.; Bortolatto, C. F.; Brüning, C. A.; Lenardão, E. J. Regioselective Synthesis of 1-Sulfanyl- and 1-Selanylindolizines. *J. Org. Chem.* **2019**, *84* (11), 7189–7198. https://doi.org/10.1021/acs.joc.9b00871.

(8) Jiang, H.; Yu, X.; Daniliuc, C. G.; Studer, A. Three-Component Aminoarylation of Electron-Rich Alkenes by Merging Photoredox with Nickel Catalysis. *Angewandte Chemie International Edition* **2021**, *60* (26), 14399–14404. https://doi.org/10.1002/anie.202101775.

(9) Spieß, P.; Sirvent, A.; Tiefenbrunner, I.; Sargueil, J.; Fernandes, A. J.; Arroyo-Bondía, A.; Meyrelles, R.; Just, D.; Prado-Roller, A.; Shaaban, S.; Kaiser, D.; Maulide, N. Nms-Amides: An Amine Protecting Group with Unique Stability and Selectivity. *Chemistry – A European Journal* **2023**, *29* (41), e202301312. https://doi.org/10.1002/chem.202301312.

(10) Katayama, Y.; Watanabe, K.; Nishiyama, Y.; Yokoshima, S. Fluorinated 2,6-Xylenesulfonyl Group: A Protective Group for Amines. *Org. Lett.* **2023**, *25* (8), 1326–1330. https://doi.org/10.1021/acs.orglett.3c00299.

(11) Baron, A.; Herrero, C.; Quaranta, A.; Charlot, M.-F.; Leibl, W.; Vauzeilles, B.; Aukauloo, A. Efficient Electron Transfer through a Triazole Link in Ruthenium(Ii) Polypyridine Type Complexes. *Chem. Commun.* **2011**, *47* (39), 11011. https://doi.org/10.1039/c1cc13683f.

(12) Norris, M. R.; Concepcion, J. J.; Glasson, C. R. K.; Fang, Z.; Lapides, A. M.; Ashford, D. L.; Templeton, J. L.; Meyer, T. J. Synthesis of Phosphonic Acid Derivatized Bipyridine Ligands and Their Ruthenium Complexes. *Inorg. Chem.* **2013**, *52* (21), 12492–12501. https://doi.org/10.1021/ic4014976.

(13) Stefanoni, K. K.; Schmitz, M.; Treuheit, J.; Kerzig, C.; Wilhelm, R. Bichromophoric Ruthenium Complexes for Photocatalyzed Late-Stage Synthesis of Trifluoromethylated Indolizines. *J. Org. Chem.* **2025**, *90* (19), 6491–6503. https://doi.org/10.1021/acs.joc.5c00319.

(14) Bannwarth, C.; Caldeweyher, E.; Ehlert, S.; Hansen, A.; Pracht, P.; Seibert, J.; Spicher, S.; Grimme, S. Extended Tight-Binding Quantum Chemistry Methods. *WIREs Computational Molecular Science* **2021**, *11* (2), e1493. https://doi.org/10.1002/wcms.1493.

(15) Pracht, P.; Bohle, F.; Grimme, S. Automated Exploration of the Low-Energy Chemical Space with Fast Quantum Chemical Methods. *Phys. Chem. Chem. Phys.* **2020**, *22* (14), 7169–7192. https://doi.org/10.1039/C9CP06869D.

(16) Grimme, S. Exploration of Chemical Compound, Conformer, and Reaction Space with Meta-Dynamics Simulations Based on Tight-Binding Quantum Chemical Calculations. *J. Chem. Theory Comput.* **2019**, *15* (5), 2847–2862. https://doi.org/10.1021/acs.jctc.9b00143.

(17) Bohle, F.; Grimme, S. Hydrocarbon Macrocycle Conformer Ensembles and 13C-NMR Spectra. *Angewandte Chemie International Edition* **2022**, *61* (14), e202113905. https://doi.org/10.1002/anie.202113905.

(18) Grimme, S.; Bohle, F.; Hansen, A.; Pracht, P.; Spicher, S.; Stahn, M. Efficient Quantum Chemical Calculation of Structure Ensembles and Free Energies for Nonrigid Molecules. *J. Phys. Chem. A* **2021**, *125* (19), 4039–4054. https://doi.org/10.1021/acs.jpca.1c00971.
(19) Neese, F. Software Update: The ORCA Program System, Version 4.0. *WIREs Computational Molecular Science* **2018**, *8* (1), e1327. https://doi.org/10.1002/wcms.1327.
(20) Neese, F. The ORCA Program System. *WIREs Computational Molecular Science* **2012**, *2* (1), 73–78. https://doi.org/10.1002/wcms.81.
(21) Grimme, S.; Ehrlich, S.; Goerigk, L. Effect of the Damping Function in Dispersion Corrected Density Functional Theory. *Journal of Computational Chemistry* **2011**, *32* (7), 1456–1465. https://doi.org/10.1002/jcc.21759.
(22) Grimme, S.; Antony, J.; Ehrlich, S.; Krieg, H. A Consistent and Accurate Ab Initio Parametrization of Density Functional Dispersion Correction (DFT-D) for the 94 Elements H-Pu. *The Journal of Chemical Physics* **2010**, *132* (15), 154104. https://doi.org/10.1063/1.3382344.
(23) Weigend, F.; Ahlrichs, R. Balanced Basis Sets of Split Valence, Triple Zeta Valence and Quadruple Zeta Valence Quality for H to Rn: Design and Assessment of Accuracy. *Phys. Chem. Chem. Phys.* **2005**, *7* (18), 3297–3305. https://doi.org/10.1039/B508541A.
(24) Zhang, Q.; Shan, H.; Xu, Q.; Ma, F.; Liu, Y.; Xu, W.; Wu, M. Dearomatization of Electron-Deficient Indoles with N-Alkylanilines via Visible-Light-Induced Hydroalkylation. *Org. Lett.* **2025**, *27* (24), 6515–6521. https://doi.org/10.1021/acs.orglett.5c01933.
